# Iridium-Catalyzed Enantioselective Propargylic C–H Trifluoromethylthiolation and Related Processes

**DOI:** 10.1021/jacs.4c12093

**Published:** 2024-10-01

**Authors:** Jiao Yu, Yue Xia, Shalini Dey, Jin Zhu, Kiu Sui Cheung, Steven J. Geib, Yi-Ming Wang

**Affiliations:** † Department of Chemistry, University of Pittsburgh, Pittsburgh, Pennsylvania 15213, United States

## Abstract

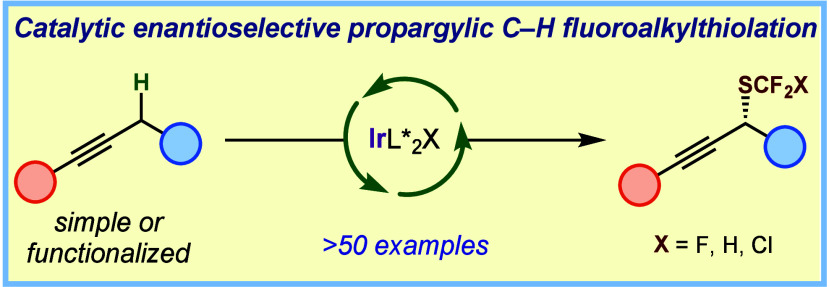

The trifluoromethylthio group (SCF_3_) has gained increasing prominence in the field of drug design and development due to its unique electronic properties, remarkable stability, and high lipophilicity, but its derivatives remain challenging to access, especially in an enantioselective manner. In this Communication, we present an enantioselective iridium-catalyzed trifluoromethylthiolation of the propargylic C(sp^3^)–H bonds of alkynes. This protocol demonstrates its efficacy across a diverse array of alkyne substrates, including B- and Si-protected terminal alkynes as well as those derived from natural products and pharmaceuticals, to give trifluoromethyl thioethers with good to excellent yield and stereoselectivity. Moreover, this protocol could be modified to access enantioenriched difluoromethyl and chlorodifluoromethyl thioethers (SCF_2_H and SCF_2_Cl derivatives), significantly expanding the space of synthetically accessible enantioenriched fluoroorganic compounds.

The development of selective and general protocols for the installation of fluorinated functional groups is a long-standing target of synthetic organic chemistry research. These efforts are largely driven by the unique physicochemical properties of fluoroorganic compounds that make them valuable targets in drug and agrochemical discovery efforts. In addition to its metabolic stability and electron-withdrawing properties (σ_p_ = 0.50), the trifluoromethyl thioether (SCF_3_ group) has garnered particular attention due to its outstanding lipophilicity (π), which surpasses those of sterically and/or electronically similar analogues (Scheme 1A).^1^ As a result, the trifluoromethylthio group and closely related analogues, like the (difluoromethyl)thio (SCF_2_H) and (chlorodifluoromethyl)thio (SCF_2_Cl) groups, have appeared in a number of investigational and currently approved pharmaceuticals and pesticides.^2^

**Scheme 1:**
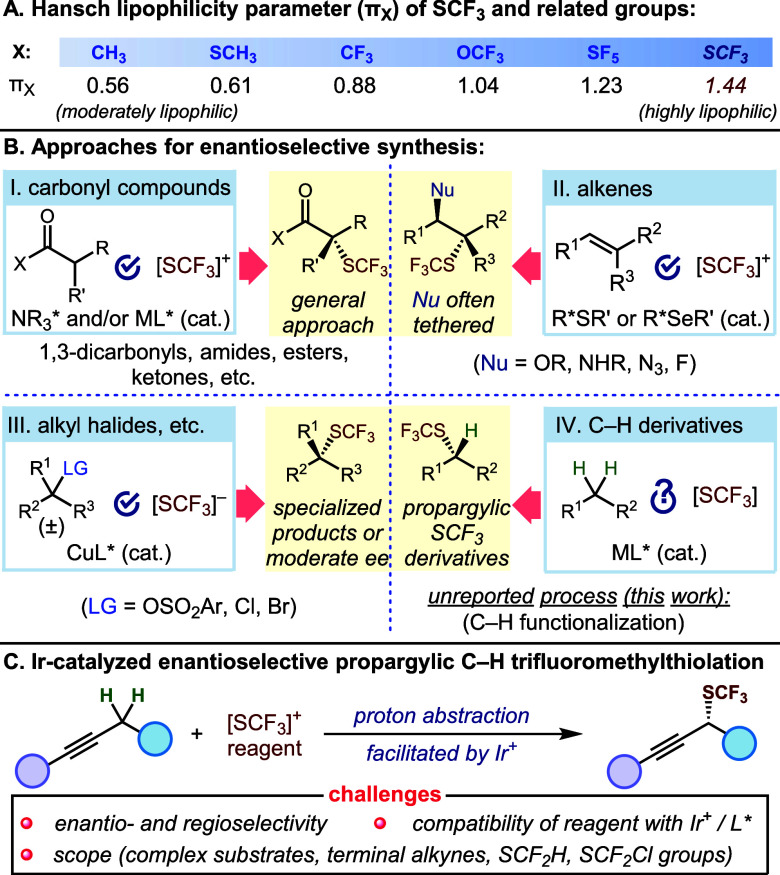
Trifluoromethyl Thioethers: Properties and Enantioselective Synthesis

Given the significance of this functional group, numerous strategies for its incorporation have recently been developed, including protocols that directly transfer an SCF_3_ group to the substrate, as well as indirect approaches that generate trifluoromethyl thioethers from other functional groups.^3^ While these techniques now allow for efficient access to a diverse range of trifluoromethyl thioethers, methods for enantioselective trifluoromethylthiolation remain scarce, severely limiting the structural diversity of α-stereogenic derivatives that are accessible in enantioenriched form.^4^

The focus of asymmetric trifluoromethylthiolation chemistry has been directed primarily toward the α-functionalization of carbonyl compounds using electrophilic reagents. This strategy was first applied to the trifluoromethylthiolation of β-ketoesters using cinchona alkaloid-based catalysts.^5^ Subsequently, a number of other protocols, including those employing chiral Lewis acids, organocatalysts, or reagents, have allowed the successful extension of this reactivity pattern to other carbonyl substrates (Scheme 1B^I^).^6^ A number of strategies, including the use of chiral chalcogenides for SCF_3_ transfer,^7^ rhodium(II) catalyst for carbene transfer,^8^ copper catalysts for nucleophilic substitution of propargylic sulfonates,^9^ and copper or nickel catalysts for coupling with benzylic radicals,^10^ have allowed a limited range of alkenes, diazoalkanes, and benzylic halides to be used as substrates for enantioselective trifluoromethylthiolation (Scheme 1B^II^, B^III^).

Despite these achievements, enantioselective trifluoromethylthiolation remains restricted in scope, and aside from carbonyl derivatives, the process is limited to substrates featuring specialized substitution patterns, directing groups, or pendent nucleophiles for intramolecular cyclization. Moreover, excluding the α-functionalization of carbonyls via enolate intermediates, methods for the enantioselective introduction of SCF_3_ generally employ polyfunctional substrates that require nontrivial synthetic sequences to access. In contrast, a broadly applicable C–H functionalization strategy would ideally allow for the use of simple and accessible starting materials while also enabling the late-stage modification of advanced intermediates.^11^ However, achieving this is challenging due to the limited repertoire of available reagents for SCF_3_ transfer and the inherent challenge of selectively functionalizing one of the enantiotopic C–H bonds of a methylene carbon.^12^

Based on our group’s progress in the α-functionalization of alkynes and alkenes using cationic complexes of carbophilic metals for π-complexation-assisted C–H deprotonation, we have found that catalysts based on Fe, Ir, and Bi demonstrate good efficiency in the functionalization of propargylic and allylic C–H bonds in the presence of a functional group-tolerant amine base.^13^ Given the availability of selective and potentially modifiable reagents for electrophilic SCF_3_ transfer,^14^ we felt that an enantioselective trifluoromethylthiolation could be achieved using this approach. However, several significant obstacles would need to be addressed. Firstly, the electrophilic and oxidizing nature of the reagent may prove incompatible with the alkyne substrate, the stoichiometric base, or the ligand system previously employed for the enantioselective allylation or silylation reactions.^15^ Secondly, the reactivity of the electrophilic sulfur may require fine-tuning so that the allenylmetal intermediate selectively undergoes C–S bond formation in the presence of other electrophilic moieties found in the reagents, additives, or byproducts.^13a,13f^ Overcoming these obstacles allowed for the eventual development of protocols for the enantioselective introduction of the SCF_3_, SCF_2_H, and SCF_2_Cl groups. To the best of our knowledge, no direct approaches for the catalytic enantioselective introduction of SCF_2_H or SCF_2_Cl had been reported.^16^

Drawing on our previous work, we began with [Ir(cod)Cl]_2_ and Carreira’s ligand^17^ as the starting point for studying this proposed process. As anticipated, highly oxidizing and electrophilic reagents were unsuitable for the transformation. For example, use of Shen’s *N*-(trifluoromethylthio)saccharin (**R1**) as the reagent resulted in formation of the N-SCF_3_ product derived from the amine base (TMPH), as well as C(sp^2^)-SCF_3_ signals indicative of direct reaction with the π bonds of the substrate but no desired product (Table 1, entry 1).^15a^ Conversely, less electrophilic reagents like Billard’s reagents **R4** also failed to yield the desired product while leaving the starting materials largely intact (entry 4).^18^ Nonetheless, modest yet promising results were obtained with Munavalli’s reagent (**R2**) and Lu and Shen’s reagent (**R3**) (entry 2, 3).^19^ Replacement of boron trifluoride with silyl Lewis acids led to dramatically improved yields and excellent levels of enantioselectivity when **R2** was employed as the SCF_3_ source (Table 1, entries 5–8). While formation of the propargylic silanes **2a′** could be suppressed by using the bulky TIPSOTf in place of TMSOTf or TESOTf, *O*-silyl hemiaminal **2a″** was unexpectedly formed from coupling of the organoiridium nucleophile with phthalimide when either TIPSOTf or ^i^PrEt_2_SiOTf were employed (entries 7,8). Aiming to improve the selectivity profile by increasing the rate of trifluoromethylthiolation relative to this deleterious processes, electron-deficient analogues of **R2** were prepared. Among them, novel 4-NO_2_-substituted phthalimide-SCF_3_ reagent **R5** and the previously reported 5-NO_2_-substituted phthalimide-SCF_3_ reagent **R6** (Table 1, entries 9, 10),^20^ proved to be the most effective, delivering the desired product with exclusive selectivity for propargylic C–S bond formation in near quantitative yields and excellent enantiomeric excesses. Notably, these reactions could be performed at room temperature, achieving a 93% yield and 97% ee at a reduced catalyst loading of 2 mol % [Ir(cod)Cl]_2_ (4 mol % Ir) and 8 mol % ligand (Table 1, entry 11). Control experiments reinforced the essential role of the catalyst, ligand, Lewis acid, and base in this reaction (Supporting Information).

**Table 1:**
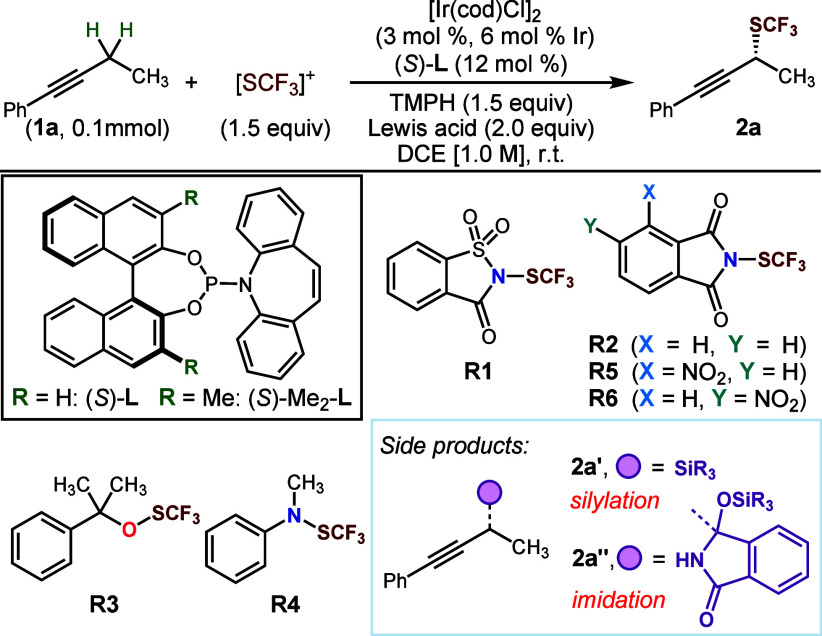
Screening of Reaction Conditions^a^

			Yield (%)			
Entry	[SCF_3_]^+^	Lewis acid	**2a**	**2a′**	**2a″**	ee (%)
1	**R1**	BF_3_·Et_2_O	0	-	-	-
2	**R2**	BF_3_·Et_2_O	7	-	-	-
3	**R3**	BF_3_·Et_2_O	1	-	-	-
4	**R4**	BF_3_·Et_2_O	0	-	-	-
5^b^	**R2**	Me_3_SiOTf	67	32	0	98
6^b^	**R2**	Et_3_SiOTf	78	20	0	98
7^b^	**R2**	^i^Pr_3_SiOTf	48	0	43	96
8^b^	**R2**	^i^Pr_3_Et_2_SiOTf	48	7	13	-
9^b^	**R5**	Et_3_SiOTf	95	0	0	96
10^b^	**R6**	Et_3_SiOTf	98	0	0	97
11^c^	**R6**	Et_3_SiOTf	93	0	0	97

a Yields were determined by ^1^H NMR spectroscopy using 1,3,5-trimethoxybenzene as the internal standard, TMPH = 2,2,6,6-tetramethylpiperidine.

b 35 °C.

c Standard conditions (see the Supporting Information).

With optimized reaction conditions established (Table 1, entries 11), we investigated the scope of the alkynes (Table 2). A series of aryl alkyl acetylenes reacted smoothly, yielding the desired products (**2a**-**2m**) in moderate to good yield and with uniformly high enantiomeric excess (ee). Substrates bearing heterocyclic aryl substituents, including those based on oxygen (**2n**-**2p**), sulfur (**2q**), and nitrogen (**2r**-**2u**) were likewise successful. We also explored conjugated enynes as substrates, which gave moderate yields and excellent enantioselectivities (**2v**, **2w**). The absolute configuration of **2f** were determined to be (*R*) by X-ray crystallographic analysis, and other products were assigned by analogy accordingly.

**Table 2:**
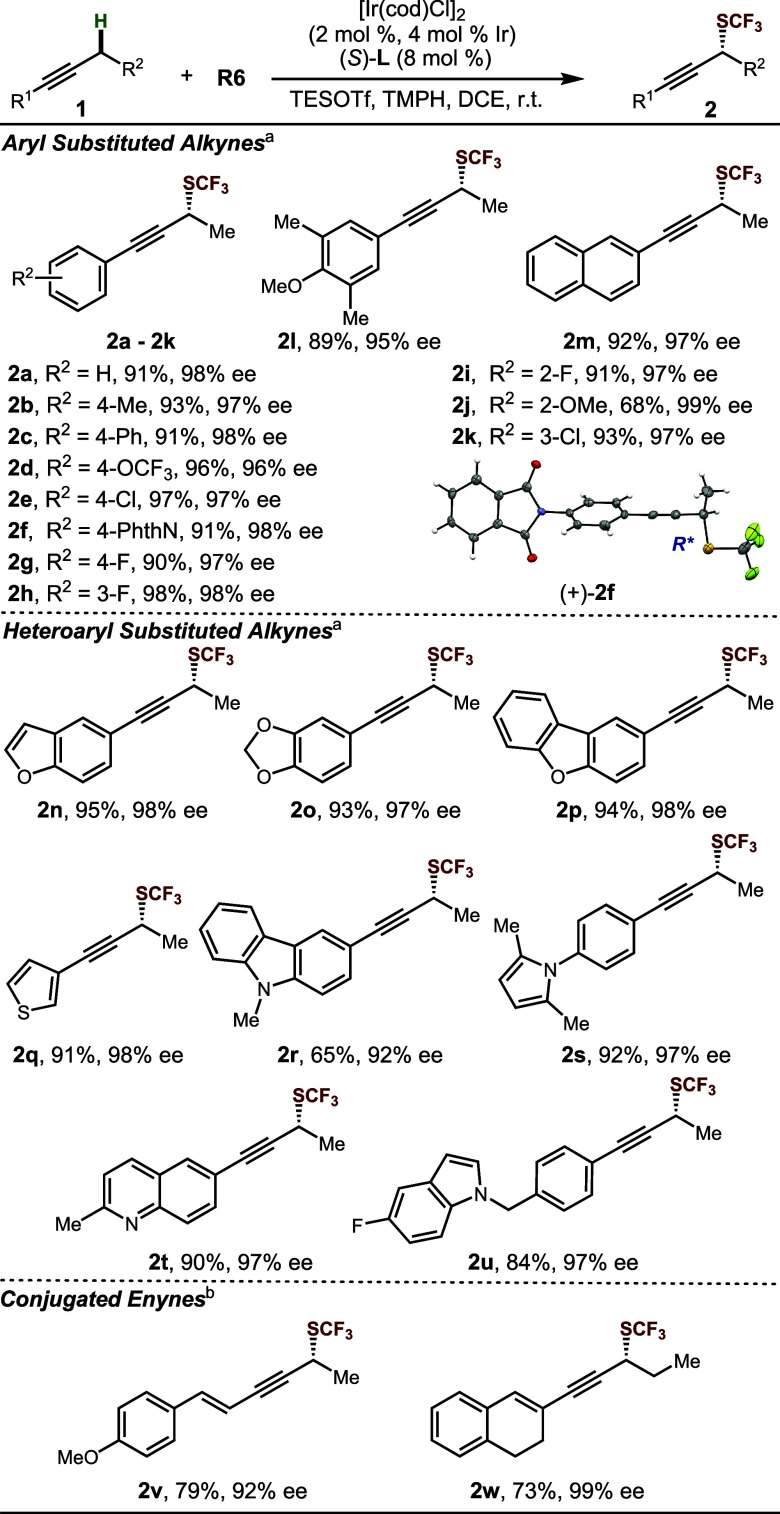
Substrate Scope of Aryl, Heteroaryl, and Conjugated Alkynes

a Standard conditions.

b 35 °C.

Furthermore, substrates carrying longer chain alkynes, dialkyl alkynes, and B- or Si-protected terminal alkynes could also be used (Table 3). Although excellent enantioselectivities were obtained in all cases, yields tend to be better groups for substrates bearing unencumbered alkyl groups (**4a**-**4d**, **4g**–**4j**), while those with bulky groups α or β to the propargylic carbon (**4e**, **4f**) gave poorer yields (<50%). Dialkylacetylenes (**4k**, **4l**) could also be used, although yields were only moderate, and somewhat lower levels of enantioselectivity were observed. Additionally, we examined protected terminal alkynes (**4m**-**4p**). We found that Bpin and Si(OMe)_3_ protected alkynes could undergo the desired trifluoromethylthiolation with moderately high ee. For example, the Bpin protected substrate **3m** achieved 81% ee, while **3n** gave a lower ee of 76%. To enhance the enantioselectivity, we explored modified ligands, finding that (*S*)-Me_2_-**L** (Table 1) increased the ee to 84% for the desired product **4n**. Additionally, the Si(OMe)_3_ protected alkynes produced similar yields and ee to the Bpin protected alkynes to give the desired products **4o** and **4p**.^21^

**Table 3:**
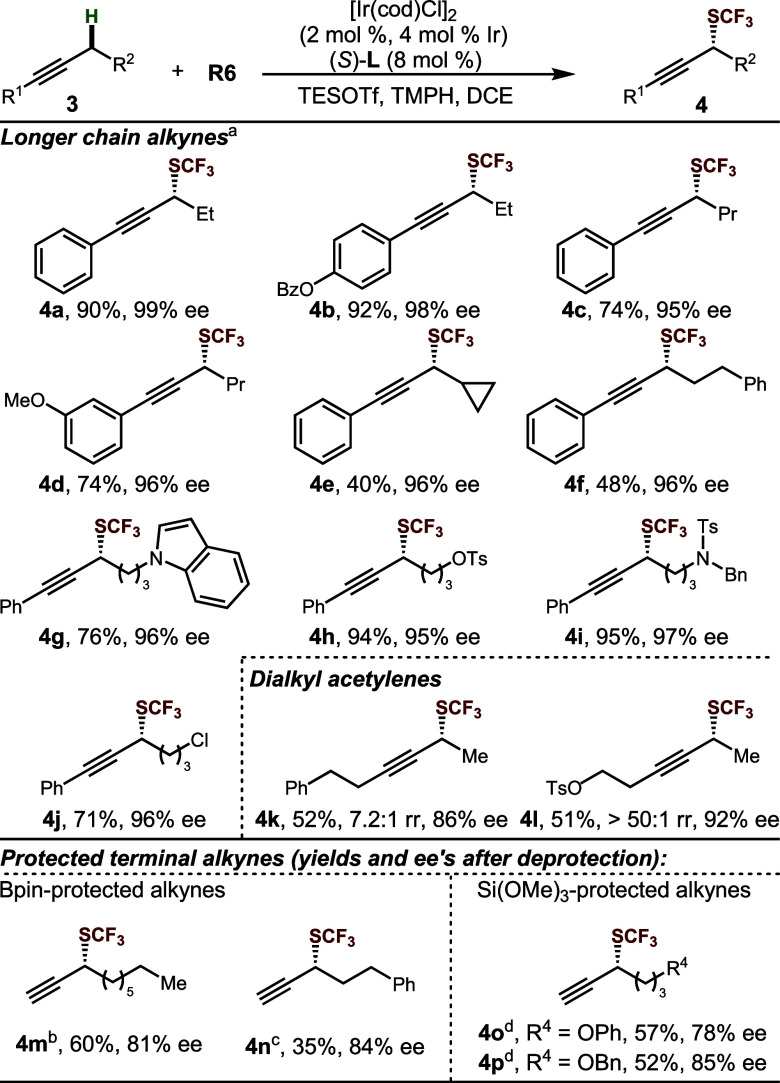
Substrate Scope of Long Chain and Protected Terminal Alkynes

a Standard conditions: 35 °C.

b-d See the Supporting Information.

We further explored the practicality and synthetic applicability of this chemistry (Scheme 2). The protocol was found to be scalable. Scaling from 0.3 to 5 mmol scale, **1c** reacted with nearly identical efficiency and enantioselectivity to give **2c** on gram-scale (Scheme 2A). We next examined a collection of substrates derived from pharmaceutical building blocks and natural products (**4q**–**4v**). Employing the current protocol, all of these complex starting materials gave good yields and excellent enantiomeric or diastereomeric excess (Scheme 2B). We note that these substrates all contain C–H bonds α to allylic or benzylic carbons or α to heteroatomic substituents, structural features that would pose regioselectivity challenges for traditional C–H functionalization strategies. Finally, to demonstrate the synthetic utility of the propargylic trifluoromethyl thioethers products, we performed several transformations to derivatize the triple bond, affording enantioenriched vinyl (pseudo)halides **2c′** and **2c′′** and triazole **4m′** without loss of stereochemical integrity (Scheme 2C).

**Scheme 2:**
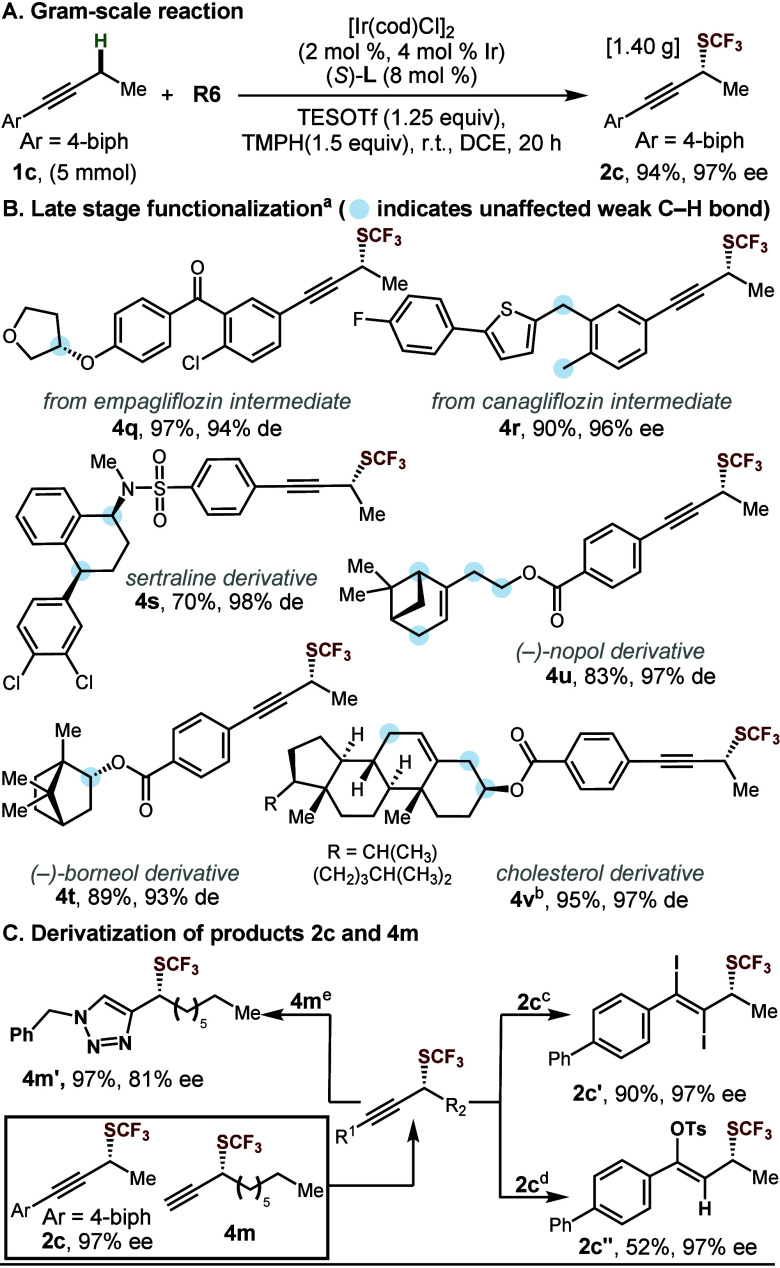
Synthetic Utility Standard conditions. DCE [0.5 M]. See the Supporting Information.

We next turned our attention to the enantioselective introduction of analogues of the SCF_3_ group. Only a handful of processes give rise to stereodefined SCF_2_H groups, and a method for the synthesis of propargylic difluoromethyl thioethers has not been reported. To introduce the SCF_2_H group, we used the reported phthalimide-SCF_2_H reagent **R7** with TIPSOTf as the Lewis acid to achieve the synthesis of enantioenriched difluoromethyl thioethers in moderate to good yield and excellent levels of enantioselectivity (Table 4, **6a**-**6c**, 95–97% ee). Furthermore, we developed the novel 4-NO_2_-substituted phthalimide-SCF_2_Cl reagent **R8** and applied it to enantioselective chlorodifluoromethylthiolation, giving desired products in moderate yield and good though somewhat diminished levels of enantioselectivity (**6d**-**6k**, 87–91% ee). To the best of our knowledge, this current protocol represents the first report of the synthesis of enantioenriched α-stereogenic chlorodifluoromethyl thioethers of any kind.

**Table 4:**
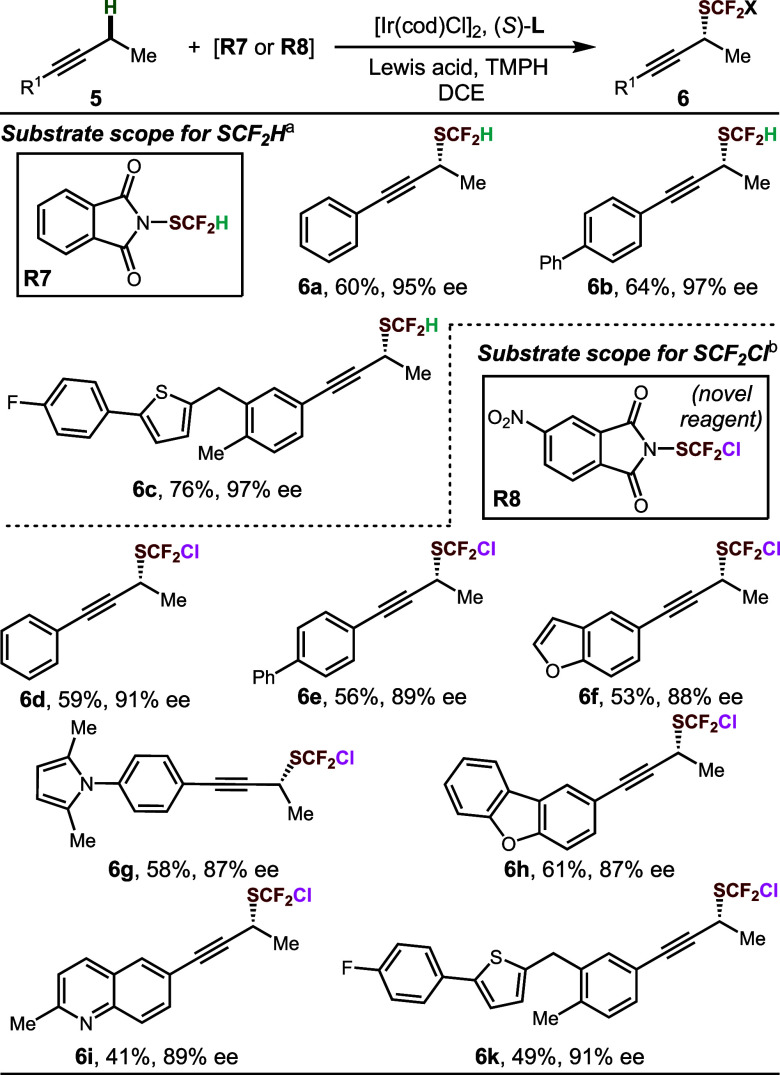
Substrate Scope for SCF_2_H and SCF_2_Cl Installation

a **R7** and TIPSOTf were used.

b **R8** and TESOTf were used.

To investigate the mechanism and probe the potential involvement of a radical pathway in our reaction, we conducted experiments using **R6** as the reagent. To test whether the reaction could be inhibited by radical scavengers, we added 9,10-dihydroanthracene, BHT, and 1,1-diphenylethylene to the reaction mixture. The yields of the desired product in the presence of these scavengers were only slightly diminished (Scheme 3A^I^). Furthermore, we performed a radical clock reaction using substrate **3e** (Scheme 3A^II^). The reaction yielded only the cyclized product, while the ring-opening product was not detected. These results suggest that our reaction is unlikely to proceed through a radical pathway. Next, we investigated the nature of the deprotonation step in our reaction using kinetic isotope effect (KIE) studies. The KIE found in independent rate measurement experiments (8.61 ± 0.47) and competition experiments (7.61 ± 0.16) indicated that the C–H bond cleavage is likely turnover-limiting (Scheme 3B^I^, 3B^II^). In addition, the observation of a strong nonlinear effect suggested that an IrL_2_^+^ complex is present in the enantiodetermining step (or steps) (see the Supporting Information).

**Scheme 3:**
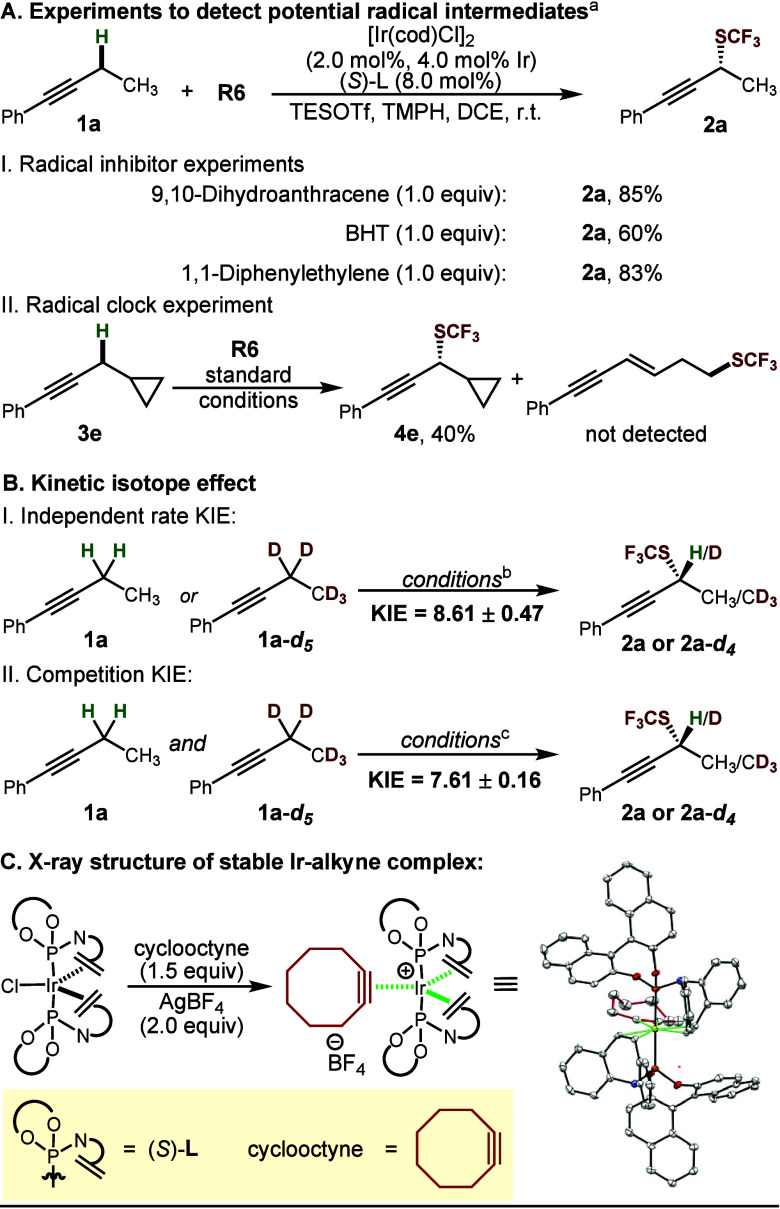
Mechanistic Studies Standard conditions. See the Supporting Information.

Lastly, we conducted experiments with different alkynes to detect the presence of a cationic Ir–alkyne complex. The ^31^P NMR spectra showed similar spectroscopic signals across various alkynes (see the Supporting Information). In order to more definitively confirm our assignment, we employed a strained alkyne to obtain a stable, isolable alkyne complex (Scheme 3C). Using cyclooctyne, we prepared and fully characterized [Ir(κ^2^-(*S*)-**L**)_2_(η^2^-C_8_H_12_)]^+^BF_4_^–^, whose structure was further confirmed by single-crystal X-ray diffraction. The similarity between the ^31^P NMR chemical shift of this complex and those previously observed for 3-hexyne and other unstrained alkynes suggests that structurally analogous (but more labile) Ir–alkyne complexes were formed as potential catalytic intermediates.

Based on these mechanistic studies and our previous in-depth study of propargylic silylation,^13f^ we propose a plausible catalytic cycle (Scheme 4). Initially, the ligand coordinates to the iridium center to form iridium complex **I**. Complex **I** can undergo a loss of chloride in the presence of TESOTf to generate cationic iridium complex **II**. Next, the alkyne substrate coordinates with **II** to form π-complex **III**. Activated by metal coordination, the α-hydrogen of the alkyne is deprotonated by TMPH, leading to the formation of allenyliridium complex **IV**. The SCF_3_ reagent, likely activated by TESOTf, then reacts with **IV** to form the product, still coordinated to iridium (**V**). Finally, the desired product is released by alkyne exchange of **V** with the starting material, regenerating **II** and closing the catalytic cycle.

**Scheme 4:**
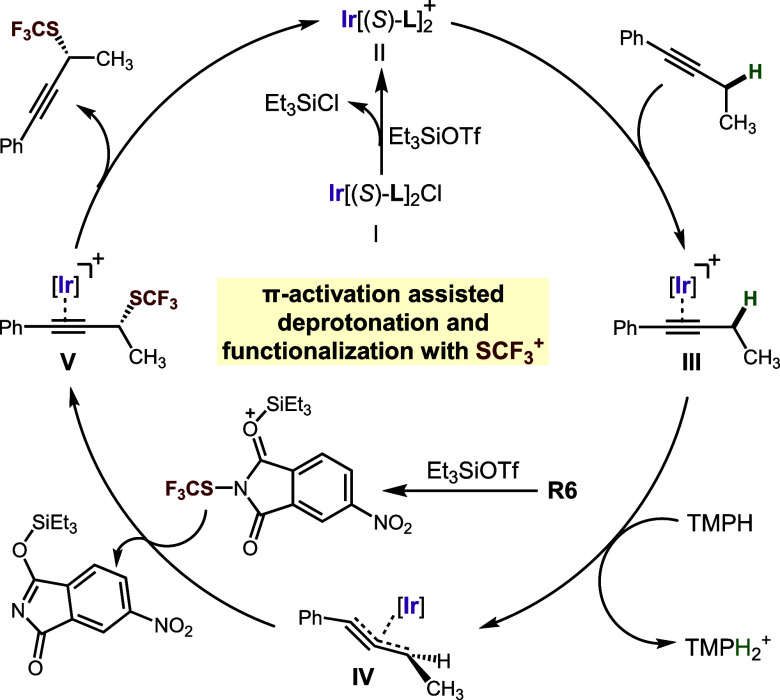
Proposed Mechanism

In summary, a direct, enantioselective propargylic C–H trifluoromethylthiolation and related fluoroalkylthiolation reactions are reported. This reaction features a broad substrate scope with high functional group tolerance, encompassing aryl- and alkyl-substituted internal alkynes and protected terminal alkynes. Furthermore, this method has proven effective for the late-stage modification of drug-like molecules and natural product derivatives, highlighting its potential utility in the modication of bioactive molecules for pharmaceutical research. Further efforts in our laboratories to employ this strategy in the preparation of enantioenriched fluoroorganics is ongoing and will be reported in due course.

## Supporting Information Available

Experimental procedures, characterization of new compounds, X-ray crystallographic data, copies of NMR spectra, and copies of HPLC traces (PDF)
