# Correction to “Iridium-Catalyzed Enantioselective Propargylic C–H Trifluoromethylthiolation and Related Processes”

**DOI:** 10.1021/jacs.4c13920

**Published:** 2024-11-05

**Authors:** Jiao Yu, Yue Xia, Shalini Dey, Jin Zhu, Kiu Sui Cheung, Steven J. Geib, Yi-Ming Wang

Due to errors in data transcription, incorrect reaction conditions and yields were displayed in Table 1 and Scheme 2 of the manuscript and in the Supporting Information. In addition, the procedure for a 5 mmol scale reaction was inadvertently left out of the Supporting Information. The corrected Table and Scheme are displayed below, and the Supporting Information has been updated with a revised version. We regret any inconvenience caused by these errors and omissions.

**Table 1:**
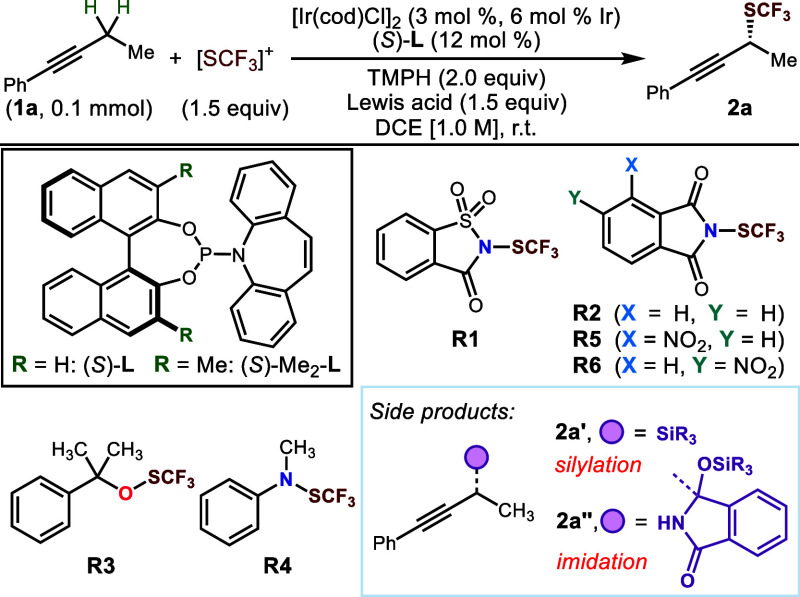
Screening of Reaction Conditions^a^

			Yield (%)			
Entry	[SCF_3_]^+^	Lewis acid	**2a**	**2a′**	**2a″**	ee (%)
1	**R1**	BF_3_·Et_2_O	0			
2	**R2**	BF_3_·Et_2_O	7			
3	**R3**	BF_3_·Et_2_O	1			
4	**R4**	BF_3_·Et_2_O	0			
5^b^	**R2**	Me_3_SiOTf	67	32	0	98
6^b^	**R2**	Et_3_SiOTf	78	20	0	98
7^b^	**R2**	^i^Pr_3_SiOTf	58	0	41	96
8^b^	**R2**	^i^PrEt_2_SiOTf	31	7	13	
9^b^	**R5**	Et_3_SiOTf	95	0	0	96
10^b^	**R6**	Et_3_SiOTf	98	0	0	97
11^c^	**R6**	Et_3_SiOTf	93	0	0	97

a Yields were determined by ^1^H NMR spectroscopy using 1,3,5-trimethoxybenzene as the internal standard, TMPH = 2,2,6,6-tetramethylpiperidine.

b 35 °C.

c [Ir(cod)Cl]_2_ (2 mol %, 4 mol % Ir), (*S*)-**L** (8 mol %), **1a** (1.0 equiv), **R6** (1.5 equiv), TMPH (2.0 equiv) and TESOTf (1.25 equiv), DCE [1.0 M], r.t., overnight.

**Scheme 2:**
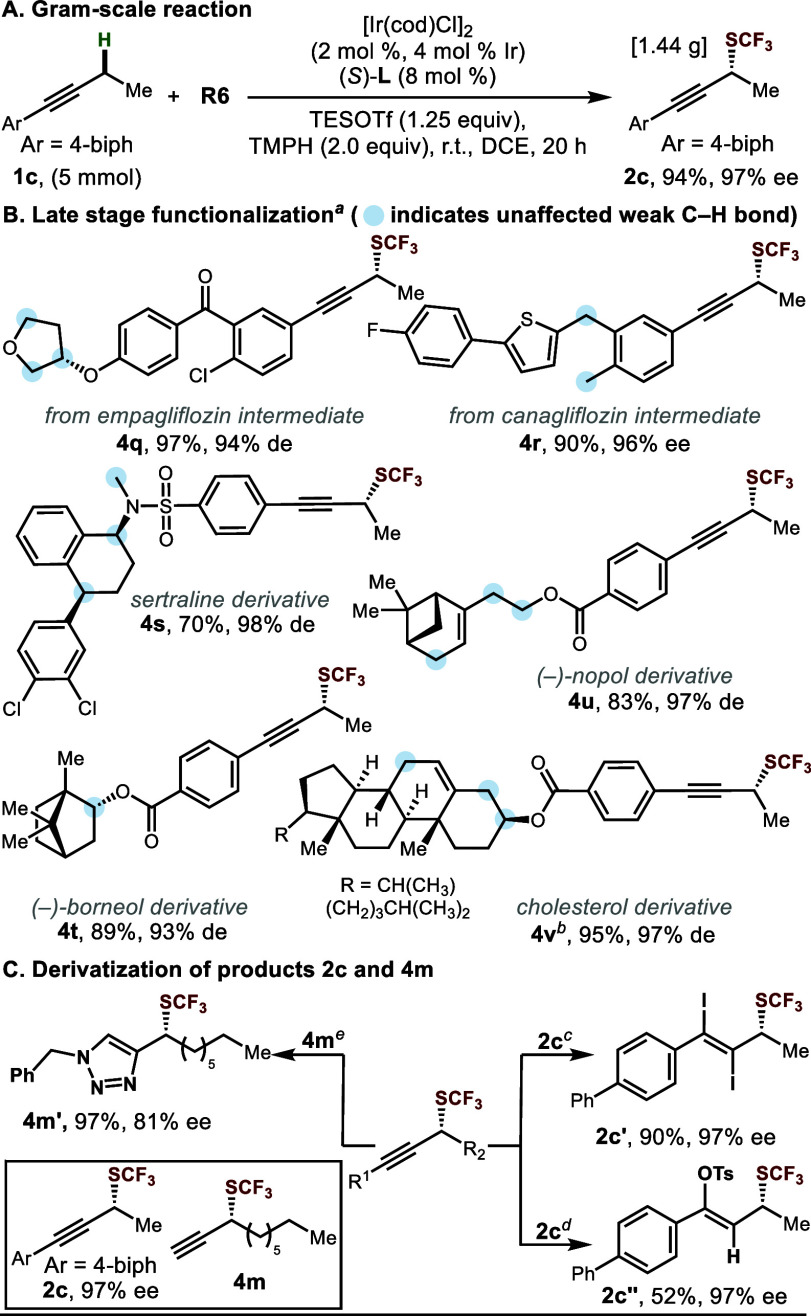
Synthetic Utility Standard conditions. DCE [0.5 M]. See the Supporting Information.

## Supporting Information Available

Experimental procedures, characterization of new compounds, X-ray crystallographic data, copies of NMR spectra, and copies of HPLC traces (PDF)

