# Supplementary material for: Correction to “Iridium-Catalyzed Enantioselective Propargylic C–H Trifluoromethylthiolation and Related Processes”
Source: J Am Chem Soc. 2024 Nov 5;146(46):32158–9. doi: 10.1021/jacs.4c13920 (PMC11583333; doi:10.1021/jacs.4c13920)
Supplement: Supplementary file 1 — ja4c13920_si_001.pdf [file ja4c13920_si_001.pdf]

Supporting Information for

Iridium-Catalyzed Enantioselective Propargylic C-H Trifluoromethylthiolation and Related Processes

Jiao Yu, Yue Xia, Shalini Dey, Jin Zhu, Kiu Sui Cheung, Steven J. Geib, and Yi-Ming Wang*
Department of Chemistry, University of Pittsburgh, Pittsburgh, Pennsylvania 15260, United States
E-mail: ym.wang@pitt.edu

Contents

General Information 2
1. General procedures for catalytic reactions 3
2. Reaction optimization 5
3. Characterization data for products 8
4. Synthetic applications of products 36
5. Mechanistic studies 39
5.1 Kinetic isotope effect experiments 39
5.2 Non-linear effect experiments 42
5.3 Control experiments and tests for potential radical intermediates 45
5.4 Efforts to detect potential intermediate species 46
6. Procedures for substrate synthesis and characterization data 52
6.1 Synthesis of non-Si-substituted alkyne substrates 52
6.2 Synthesis of $Si(OMe)_3$-protected alkyne substrates **3o**, **3p** 54
7. Procedure for **R8** synthesis and characterization data 56
8. X-ray structure of **2f** and iridium cyclooctyne complex 59
9. Copies of NMR spectra of products 61
10. Copies of HPLC traces 177
11. References 234

General Information

General Reagent Information:

Anhydrous solvents were purchased from Acros (AcroSeal packaging) or Sigma Aldrich (Sure/Seal packaging) and were transferred into an argon-filled glovebox and used as received. Bis(1,5-cyclooctadiene) diiridium (I) dichloride was purchased from Sigma Aldrich and was used as received. 2,2,6,6-Tetramethylpiperidine (TMPH) was purchased from Sigma Aldrich and vacuum distilled from CaH_2. (*Note:* Distilled TMPH must be stored under inert atmosphere.) All other reagents were purchased from TCI, Strem, Oakwood, Acros, Alfa Aesar, or Sigma Aldrich and used as received. Compounds were purified by flash column chromatography using SiliCycle *SiliaFlash® F60* silica gel, unless otherwise indicated. Compounds were visualized by irradiation with UV light, or by staining with iodine/silica gel, ceric ammonium molybdate, or potassium permanganate. Yields refer to isolated compounds, unless otherwise indicated.

General Analytical Information:

All new compounds (starting materials and products) were characterized by ^{1}H NMR, ^{13}C NMR, and high-resolution mass spectrometry, as well as by ^{19}F NMR where appropriate. All ^{1}H NMR data are reported in δ units, parts per million (ppm), and were measured relative to the residual proton signal in the deuterated solvent at 7.26 ppm ($CDCl_3$), 5.32 ppm (CD_2Cl_2). Data for ^{1}H and ^{19}F NMR are reported as follows: chemical shift (δ ppm), multiplicity (s = singlet, d = doublet, t = triplet, q = quartet, quint = quintet, sept = septet, m = multiplet), integration, and coupling constant (Hz). All ^{13}C NMR spectra are ^{1}H decoupled and reported in ppm relative to the solvent signal at 77.16 ppm ($CDCl_3$), 53.84 ppm (CD_2Cl_2). Enantiomeric excesses (ee) were determined by HPLC (high performance liquid chromatography) analysis using a chiral stationary phase. Specific columns and analytical methods are provided in the experimental details for individual compounds; the wavelengths of light used for chiral analyses are provided with the associated chromatograms. High Resolution Mass spectra were obtained on a Bruker Daltonics, Inc. APEXIII 7.0 TESLA FTMS instrument (ESI) or Waters Micromass GCT Premier instrument (EI). Specific optical rotation was collected on Jacso P-2000 Digital Polarimeter.

1. General procedures for catalytic reactions

General procedure A

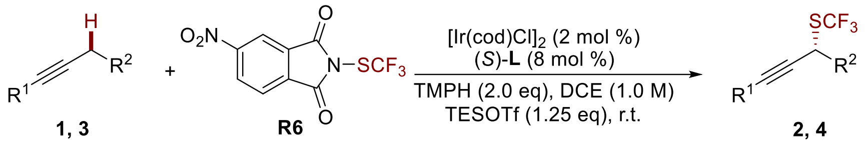

Preparation of the Ir complex solution: In an argon-filled glovebox, an oven-dried reaction vial equipped with a magnetic stir bar was charged with $[Ir(cod)Cl]_2$ (2.7 mg, 0.004 mmol, 2.0 mol %) and phosphoramidite (*S*)-**L** (8.1 mg, 0.016 mmol, 8 mol %), followed by the addition of 1,2-dichloroethane (0.2 mL) via syringe. The resultant solution was stirred at room temperature (r.t.) for 1 hour to give a dark red solution.

Ir-catalyzed trifluoromethylthiolation reaction: To the solution were added **R6** (0.3 mmol, 1.5 equiv), alkyne substrate **1** (0.2 mmol, 1.0 equiv), distilled TMPH (70 µL, 0.4 mmol, 2.0 equiv) and triethylsilyl trifluoromethanesulfonate (58 µL, 0.25 mmol, 1.25 equiv). The reaction tube was then capped, removed from the glovebox, and the reaction mixture was stirred at r.t. overnight. The crude mixture was filtered through a short pad of silica to remove insoluble materials, washed with CH_2Cl_2 or EtOAc, and concentrated *in vacuo*. The crude material was purified by flash column chromatography on silica gel to obtain the desired product.

For HPLC analysis, racemic samples were prepared following general procedure **A** using achiral phosphoramidite ligand 5-(dibenzo[*d*,*f*][1,3,2]dioxaphosphepin-6-yl)-5*H*-dibenzo[*b*,*f*]azepine (**L**$_{rac}$).

General procedure B

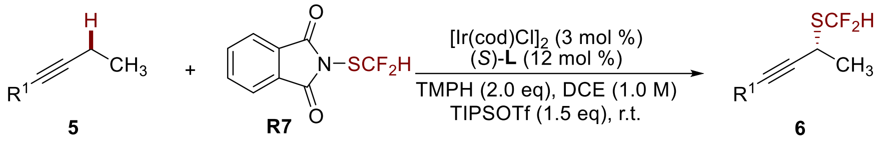

Preparation of the Ir complex solution: In an argon-filled glovebox, an oven-dried reaction vial equipped with a magnetic stir bar was charged with $[Ir(cod)Cl]_2$ (2.0 mg, 0.003 mmol, 3.0 mol %) and phosphoramidite (*S*)-**L** (6.1 mg, 0.012 mmol, 12 mol %), followed by the addition of 1,2-dichloroethane (0.1 mL) via syringe. The resultant solution was stirred at room temperature (r.t.) for 1 hour to give a dark red solution.

Ir-catalyzed difluoromethylthiolation reaction: To the solution were added **R7** (0.15 mmol, 1.5 equiv), alkyne substrate **1** (0.1 mmol, 1.0 equiv), distilled TMPH (35 µL, 0.2 mmol, 2.0 equiv) and triisopropylsilyl trifluoromethanesulfonate (41 µL, 0.15 mmol, 1.5 equiv). The reaction tube was then capped, removed from the glovebox, and the reaction mixture was stirred at r.t. overnight. The crude mixture was filtered through a short pad of silica to remove insoluble materials, washed with CH_2Cl_2 or EtOAc, and concentrated *in vacuo*. The crude material was purified by flash column chromatography on silica gel to obtain the desired product.

For HPLC analysis, racemic samples were prepared following general procedure **A** using achiral phosphoramidite ligand 5-(dibenzo[*d*,*f*][1,3,2]dioxaphosphepin-6-yl)-5*H*-dibenzo[*b*,*f*]azepine (**L**$_{rac}$).

General procedure C

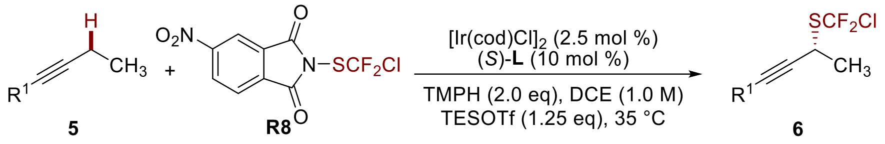

Preparation of the Ir complex solution: In an argon-filled glovebox, an oven-dried reaction vial equipped with a magnetic stir bar was charged with $[Ir(cod)Cl]_2$ (3.4 mg, 0.005 mmol, 2.5 mol %) and phosphoramidite (*S*)-**L** (10.1 mg, 0.020 mmol, 10 mol %), followed by the addition of 1,2-dichloroethane (0.2 mL) via syringe. The resultant solution was stirred at room temperature (r.t.) for 1 hour to give a dark red solution.

Ir-catalyzed chlorodifluoromethylthiolation reaction: To the solution were added **R8** (0.3 mmol, 1.5 equiv), alkyne substrate **5** (0.2 mmol, 1.0 equiv), distilled TMPH (70 µL, 0.4 mmol, 2.0 equiv) and triethylsilyl trifluoromethanesulfonate (58 µL, 0.25 mmol, 1.25 equiv). The reaction tube was then capped, removed from the glovebox, and the reaction mixture was stirred at 35 °C overnight. The crude mixture was filtered through a short pad of silica to remove insoluble materials, washed with CH_2Cl_2 or EtOAc, and concentrated *in vacuo*. The crude material was purified by flash column chromatography on silica gel to obtain the desired product.

For HPLC analysis, racemic samples were prepared following general procedure **A** using achiral phosphoramidite ligand 5-(dibenzo[*d*,*f*][1,3,2]dioxaphosphepin-6-yl)-5*H*-dibenzo[*b*,*f*]azepine (**L**$_{\mathbf{rac}}$).

General procedure D

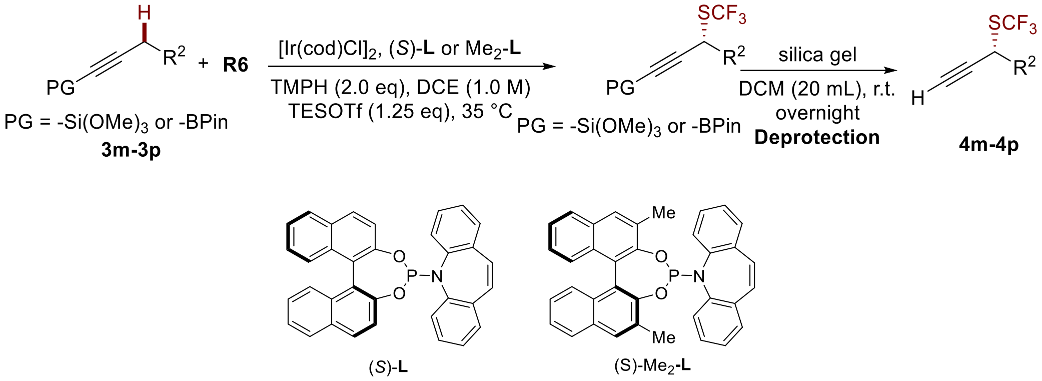

Preparation of the Ir complex solution: In an argon-filled glovebox, an oven-dried reaction vial equipped with a magnetic stir bar was charged with $[Ir(cod)Cl]_2$ (X mol %) and phosphoramidite (*S*)-**L** (4X mol %) or (*S*)-**L**(2-Me)$_2$ (4X mol %) depending on different substrate, followed by the addition of 1,2-dichloroethane (0.2 mL) via syringe. The resultant solution was stirred at room temperature (r.t.) for 1 hour to give a dark red solution.

Ir-catalyzed trifluoromethylthiolation reaction: To the solution were added **R6** (0.3 mmol, 1.5 equiv), alkyne substrate **3** (0.2 mmol, 1.0 equiv), distilled TMPH (70 µL, 0.4 mmol, 2.0 equiv) and triethylsilyl trifluoromethanesulfonate (58 µL, 0.25 mmol, 1.25 equiv). The reaction tube was then capped, removed from the glovebox, and the reaction mixture was stirred at 35 °C overnight.

Deprotection of -Bpin or -Si(OMe)$_3$: The reaction mixture was diluted with 20 mL of CH_2Cl_2, silica gel (~15 g) was added thereto, and the mixture was then stirred at r.t. overnight. The crude material was purified by flash column chromatography on silica gel to obtain the desired product.

For HPLC analysis, racemic samples were prepared following general procedure **A** using achiral phosphoramidite ligand 5-(dibenzo[*d*,*f*][1,3,2]dioxaphosphepin-6-yl)-5*H*-dibenzo[*b*,*f*]azepine (**L**$_{\mathbf{rac}}$).

2. Reaction optimization

Initial investigations

In an argon-filled glovebox, an oven-dried reaction vial equipped with a magnetic stir bar was charged with $[Ir(cod)Cl]_2$ (2.0 mg, 0.003 mmol, 3.0 mol %) and phosphoramidite (*S*)-**L** (6.8 mg, 0.012 mmol, 12 mol %), followed by the addition of 1,2-dichloroethane (0.1 mL) via syringe. The resultant solution was stirred at room temperature (r.t.) for 1 hour to give a dark red solution. To the solution were added $[SCF_3]^+$ reagent (0.15 mmol, 1.5 equiv), alkyne substrate **1** (0.1 mmol, 1.0 equiv), distilled TMPH (2.0 equiv) and Lewis acid (1.5 equiv). The reaction tube was then capped, removed from the glovebox, and the reaction mixture was stirred at r.t. overnight. Yields were determined by ^1H NMR spectroscopy using 1,3,5-trimethoxybenzene as the internal standard.

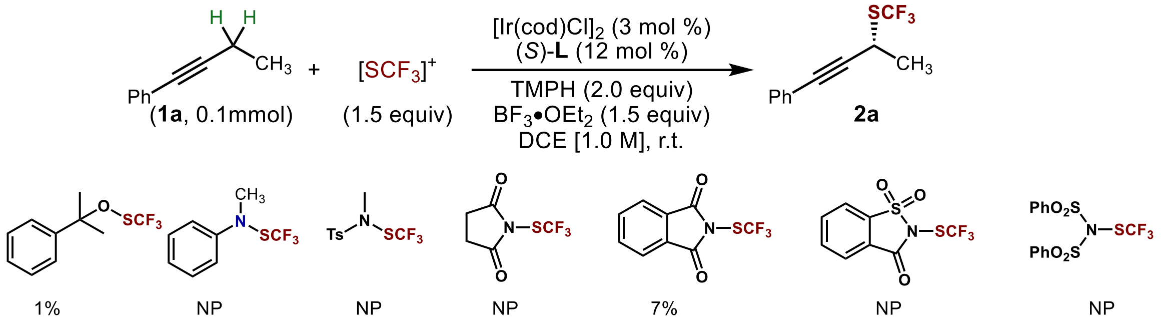

Lewis acid evaluation:

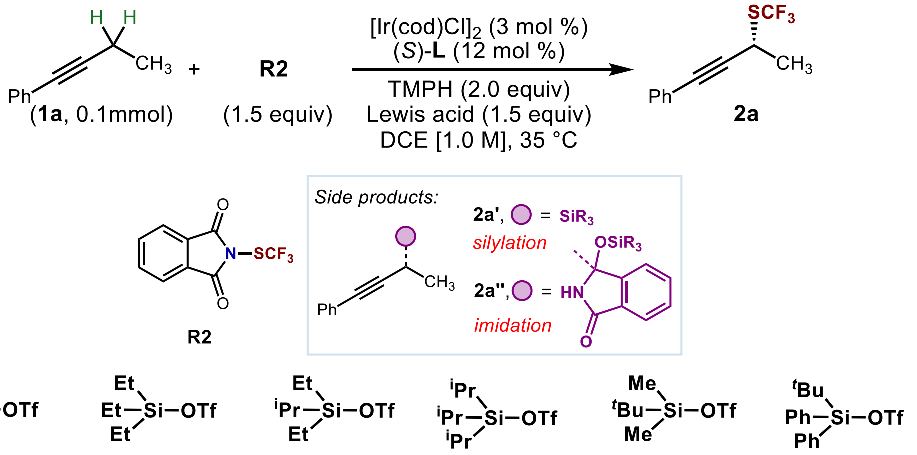

Entry	[+SCF3]	Lewis acid	Yield(%)			ee (%)
			**2a**	**2a'**	**2a"**	
1	**R2**	TMSOTf	67	32	0	98
2	**R2**	TESOTf	78	20	0	98
3	**R2**	$Et_2{}^iPrSiOTf$	31	7	13	-
4	**R2**	TIPSOTf	58	0	41	96
5	**R2**	TBDMSOTf	56	0	38	94
6	**R2**	$Ph_2{}^tBuSiOTf$	28	0	12	-

Solvent evaluation:

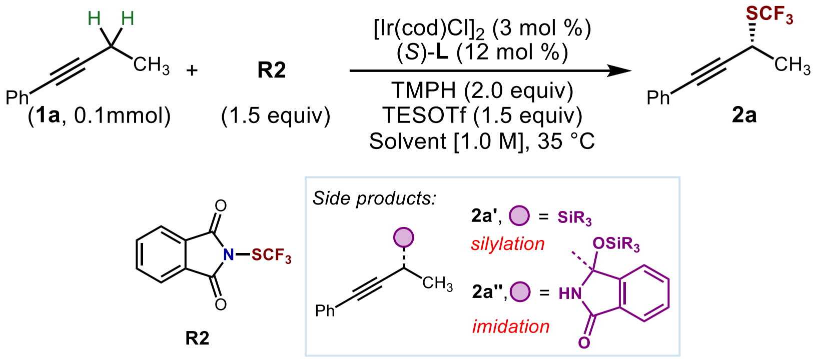

Entry	[+SCF3]	Solvent	Yield(%)			ee (%)
			**2a**	**2a’**	**2a”**	
1	**R2**	DCE	78	20	0	98
2	**R2**	DCM	40	44	0	96
3	**R2**	Toluene	77	17	0	95
4	**R2**	$PhCF_3$	69	27	0	95

Modification of Munavalli's reagent:

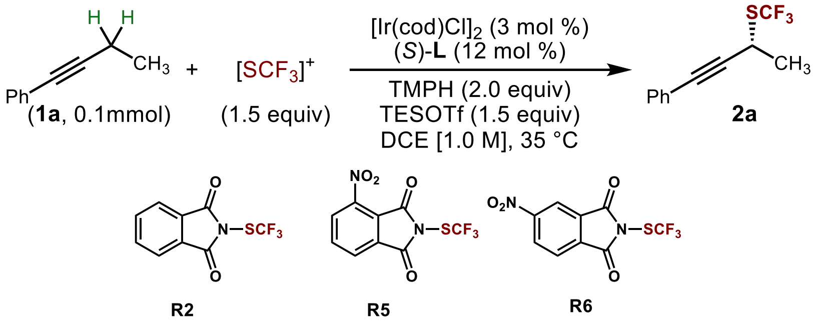

Entry	[+SCF3]	solvent	Yield(%)			ee (%)
			**2a**	**2a’**	**2a”**	
1	**R2**	DCE	78	20	0	98
2	**R5**	DCE	95	0	0	96
3	**R6**	DCE	98	0	0	97

Stoichiometry of reactants:

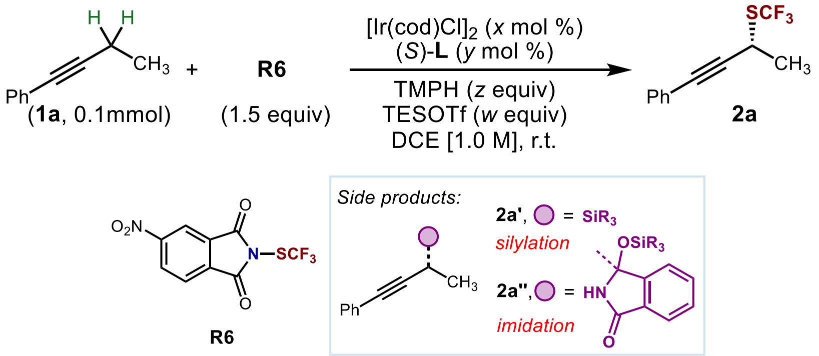

Entry	[+SCF_3]	$[Ir(cod)Cl]_2$: L : Acid : Base	Yield(%)			ee (%)
			**2a**	**2a'**	**2a"**	
1	**R6**	3.0% : 12% : 1.5 : 2	98	0	0	97
2	**R6**	2.5% : 10% : 1.5 : 2	91	2	0	97
3	**R6**	2.0% : 8% : 1.5 : 2	91	2	0	97
4	**R6**	1.5% : 6% : 1.5 : 2	83	4	0	97
5	**R6**	2.0% : 8% : 2.0: 2	90	0	0	97
6	**R6**	2.0% : 8% : 1.25 : 2	93	0	0	97
7	**R6**	2.0% : 8% : 1.0 : 2	89	0	0	97
8	**R6**	2.0% : 8% : 0.5 : 2	44	0	0	-
9	**R6**	2.0% : 8% : 1.25 : 1.5	92	2	0	97
10	**R6**	2.0% : 8% : 1.25 : 1.25	86	2	0	97

3. Characterization data for products

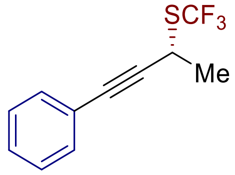

2a

(*R*)-(4-Phenylbut-3-yn-2-yl)(trifluoromethyl)sulfane:

Prepared according to **General Procedure A**. The reaction mixture was purified by column chromatography on silica gel (eluent: hexanes, R_f = 0.55) to yield the product **2a** as a colorless oil (42.1 mg, 91% yield, 98% ee).
$[\alpha]_D^{25}$ = +236.1 (c 0.23, $CHCl_3$)
HPLC analysis: Chiralcel OD-3 (hexanes/*i*-PrOH = 100/0, flow rate 1.0 mL/min, λ = 243 nm), t_R (major) = 6.98 min, t_R (minor) = 8.09 min.
^{1}H NMR (400 MHz, $CDCl_3$) δ 7.47 – 7.40 (m, 2H), 7.36 – 7.29 (m, 3H), 4.35 (q, *J* = 7.0 Hz, 1H), 1.73 (d, *J* = 7.0 Hz, 3H).
^{19}F NMR (376 MHz, $CDCl_3$) δ -40.12 (s, 3F).
^{13}C NMR (101 MHz, $CDCl_3$) δ 131.7, 130.3 (q, *J* = 307.7 Hz), 128.6, 128.3, 122.3, 87.3, 84.6, 30.9 (q, *J* = 2.6 Hz), 22.6.
HRMS (ESI) m/z calcd. for $C_{11}H_{10}F_3S$ $[M+H]^+$ 231.0450, found 231.0444.

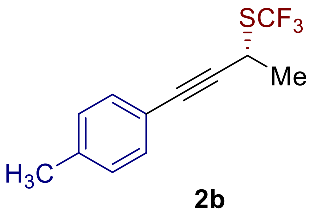

2b

(*R*)-(4-(*p*-Tolyl)but-3-yn-2-yl)(trifluoromethyl)sulfane:

Prepared according to **General Procedure A**. The reaction mixture was purified by column chromatography on silica gel (eluent: hexanes, R_f = 0.45) to yield the product **2b** as a colorless oil (45.6 mg, 93% yield, 97% ee).
$[\alpha]_D^{25}$ = +253.6 (c 0.32, $CHCl_3$)
HPLC analysis: Chiralcel OD-3 (hexanes/*i*-PrOH = 100/0, flow rate 1.0 mL/min, λ = 248 nm), t_R (major) = 8.04 min, t_R (minor) = 8.47 min.
^{1}H NMR (400 MHz, $CDCl_3$) δ 7.32 (d, *J* = 8.1 Hz, 2H), 7.12 (d, *J* = 8.1 Hz, 2H), 4.35 (q, *J* = 7.0 Hz, 1H), 2.35 (s, 3H), 1.72 (d, *J* = 7.0 Hz, 3H).
^{19}F NMR (376 MHz, $CDCl_3$) δ -40.12 (s, 3F).
^{13}C NMR (101 MHz, $CDCl_3$) δ 138.8, 131.6, 130.4 (q, *J* = 307.8 Hz), 129.1, 119.2, 86.6, 84.7, 31.0 (q, *J* = 2.6 Hz), 22.7, 21.5.
HRMS (ESI) m/z calcd. for $C_{12}H_{12}F_3S$ $[M+H]^+$ 245.0606, found 245.0607.

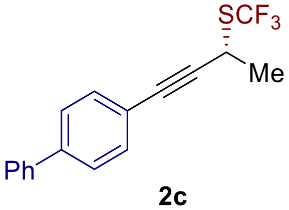

2c

(*R*)-(4-([1,1'-Biphenyl]-4-yl)but-3-yn-2-yl)(trifluoromethyl)sulfane:

Prepared according to **General Procedure A**. The reaction mixture was purified by column chromatography on silica gel (eluent: 10:1 hexanes/CH_2Cl_2, R_f = 0.50) to yield the product **2c** as a light yellow solid (55.5 mg, 91% yield, 98% ee).

m.p. 48.0 °C – 49.0 °C

$[\alpha]_D^{25}$ = +131.8 (c 0.63, $CHCl_3$)

HPLC analysis: Chiralcel OD-3 (hexanes/*i*-PrOH = 100/0, flow rate 1.0 mL/min, λ = 277 nm), t_R (major) = 16.21 min, t_R (minor) = 24.01 min.

^{1}H NMR (400 MHz, $CDCl_3$) δ 7.57 – 7.45 (m, 4H), 7.44 – 7.34 (m, 4H), 7.32 – 7.25 (m, 1H), 4.30 (q, *J* = 7.0 Hz, 1H), 1.67 (d, *J* = 7.0 Hz, 3H).

^{19}F NMR (376 MHz, $CDCl_3$) δ -40.08 (s, 3F).

^{13}C NMR (101 MHz, $CDCl_3$) δ 141.4, 140.3, 132.2, 130.4 (q, *J* = 307.8 Hz), 128.9, 127.7, 127.1, 127.0, 121.2, 87.9, 84.4, 30.9 (q, *J* = 2.6 Hz), 22.6.

HRMS (ESI) m/z calcd. for $C_{17}H_{14}F_3S$ $[M+H]^+$ 307.0763, found 307.0768.

Protocol for reaction on 5.0 mmol scale:

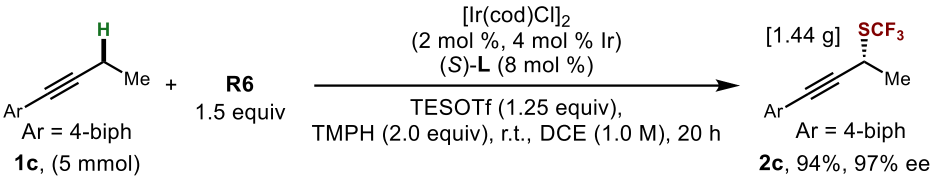

Preparation of the Ir complex solution: In an argon-filled glovebox, a 25 mL oven-dried Schlenk tube equipped with a magnetic stir bar was charged with $[Ir(cod)Cl]_2$ (67.2 mg, 0.1 mmol, 2.0 mol %) and phosphoramidite (*S*)-**L** (202.8 mg, 0.4 mmol, 8 mol %), followed by the addition of 1,2-dichloroethane (5.0 mL) via syringe. The resultant solution was stirred at room temperature (r.t.) for 1 hour to give a dark red solution.

Ir-catalyzed trifluoromethylthiolation reaction: To the solution were added **R6** (2.19 g, 7.5 mmol, 1.5 equiv), alkyne substrate **1c** (1.03 g, 5.0 mmol, 1.0 equiv), distilled TMPH (1.75 mL, 10.0 mmol, 2.0 equiv) and triethylsilyl trifluoromethanesulfonate (1.45 mL, 6.25 mmol, 1.25 equiv). The reaction tube was then capped, removed from the glovebox, and the reaction mixture was stirred at r.t. for 20 h. The crude mixture was filtered through a short pad of silica to remove insoluble materials, washed with CH_2Cl_2, and concentrated *in vacuo*. The reaction mixture was purified by column chromatography on silica gel (eluent: 10:1 hexanes/CH_2Cl_2, R_f = 0.50) to yield the product **2c** as a light yellow solid (1.44 g, 94% yield, 97% ee).

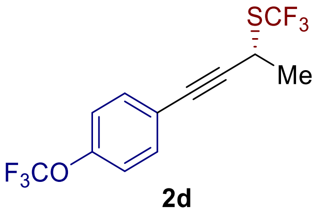

2d

(*R*)-(4-(4-(Trifluoromethoxy)phenyl)but-3-yn-2-yl)(trifluoromethyl)sulfane:

Prepared according to **General Procedure A**. The reaction mixture was purified by column chromatography on silica gel (eluent: hexanes, R_f = 0.40) to yield the product **2d** as a colorless oil (60.2 mg, 96% yield, 96% ee).
[α]$_D^{25}$ = +104.9 (c 0.44, $CHCl_3$)
HPLC analysis: Chiralcel OJ-3 (hexanes/*i*-PrOH = 100/0, flow rate 0.6 mL/min, λ = 245 nm), t_R (major) = 7.45 min, t_R (minor) = 7.57 min.
^{1}H NMR (400 MHz, $CDCl_3$) δ 7.45 (d, *J* = 8.7 Hz, 2H), 7.16 (d, *J* = 8.7 Hz, 2H), 4.33 (q, *J* = 7.1 Hz, 1H), 1.72 (d, *J* = 7.1 Hz, 3H).
^{19}F NMR (376 MHz, $CDCl_3$) δ -40.15 (s, 3F), -57.84 (s, 3F).
^{13}C NMR (101 MHz, $CDCl_3$) δ 149.2 (q, *J* = 1.9 Hz), 133.3, 130.3 (q, *J* = 307.7 Hz), 121.1, 120.8, 120.4 (q, *J* = 257.9 Hz), 88.3, 83.1, 30.7 (q, *J* = 2.6 Hz), 22.3.
HRMS (APCI) m/z calcd. for $C_{12}H_9OF_6S$ $[M+H]^+$ 315.0273, found 315.0273.

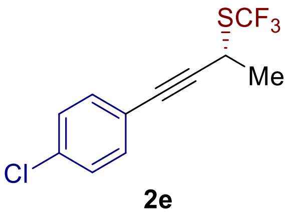

2e

(*R*)-(4-(4-Chlorophenyl)but-3-yn-2-yl)(trifluoromethyl)sulfane:

Prepared according to **General Procedure A**. The reaction mixture was purified by column chromatography on silica gel (eluent: hexanes, R_f = 0.55) to yield the product **2e** as a colorless oil (51.1 mg, 97% yield, 97% ee).
[α]$_D^{25}$ = +217.6 (c 0.25, $CHCl_3$)
HPLC analysis: Chiralcel OD-3 (hexanes/*i*-PrOH = 100/0, flow rate 1.0 mL/min, λ = 252 nm), t_R (major) = 5.18 min, t_R (minor) = 5.49 min.
^{1}H NMR (400 MHz, $CDCl_3$) δ 7.35 (d, *J* = 8.6 Hz, 2H), 7.29 (d, *J* = 8.6 Hz, 2H), 4.33 (q, *J* = 7.1 Hz, 1H), 1.72 (d, *J* = 7.1 Hz, 2H).
^{19}F NMR (376 MHz, $CDCl_3$) δ -40.13 (s, 3F).
^{13}C NMR (101 MHz, $CDCl_3$) δ 132.9, 130.3 (q, *J* = 307.7 Hz), 128.7, 120.8, 88.3, 83.4, 30.7 (q, *J* = 2.7 Hz), 22.4.
HRMS (ESI) m/z calcd. for $C_{11}H_9ClF_3S$ $[M+H]^+$ 265.0060, found 265.0063.

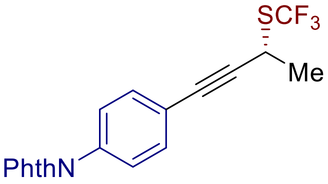

2f

(*R*)-2-(4-(3-((Trifluoromethyl)thio)but-1-yn-1-yl)phenyl)isoindoline-1,3-dione:

Prepared according to **General Procedure A**. The reaction mixture was purified by column chromatography on silica gel (eluent: 2:1 hexanes/CH_2Cl_2, R_f = 0.50) to yield the product **2e** as a white solid (63.9 mg, 85% yield, 96% ee).
m.p. 123.0 °C – 124.0 °C
$[\alpha]_D^{25}$ = +106.3 (c 0.28, $CHCl_3$)
HPLC analysis: Chiralcel AD-3 (hexanes/*i*-PrOH = 98/2, flow rate 0.8 mL/min, λ = 267 nm), t_R (major) = 23.76 min, t_R (minor) = 24.64 min.
^{1}H NMR (400 MHz, $CDCl_3$) δ 7.95 (s, 2H), 7.80 (s, 2H), 7.55 (d, *J* = 8.2 Hz, 2H), 7.44 (d, *J* = 8.2 Hz, 2H), 4.36 (q, *J* = 7.0 Hz, 1H), 1.73 (d, *J* = 7.0 Hz, 3H).
^{19}F NMR (376 MHz, $CDCl_3$) δ -40.08 (s, 3F).
^{13}C NMR (101 MHz, $CDCl_3$) δ 166.9, 132.4, 131.9, 131.7, 130.3 (q, *J* = 307.7 Hz), 126.2, 123.9, 122.0, 88.3, 83.8, 30.8 (q, *J* = 2.8 Hz), 22.4.
HRMS (ESI) m/z calcd. for $C_{19}H_{13}O_2NF_3S$ $[M+H]^+$ 376.0614, found 376.0614.

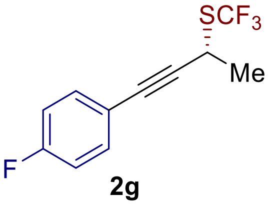

2g

(*R*)-(4-(4-Fluorophenyl)but-3-yn-2-yl)(trifluoromethyl)sulfane:

Prepared according to **General Procedure A**. The reaction mixture was purified by column chromatography on silica gel (eluent: hexanes, R_f = 0.55) to yield the product **2g** as a colorless oil (44.6 mg, 90% yield, 97% ee).
$[\alpha]_D^{25}$ = +464.0 (c 0.10, $CHCl_3$)
HPLC analysis: Chiralcel OD-3 (hexanes/*i*-PrOH = 100/0, flow rate 1.0 mL/min, λ = 243 nm), t_R (major) = 4.64 min, t_R (minor) = 4.84 min.
^{1}H NMR (400 MHz, $CDCl_3$) δ 7.44 – 7.34 (m, 2H), 7.01 (t, *J* = 8.7 Hz, 2H), 4.33 (q, *J* = 7.0 Hz, 1H), 1.71 (d, *J* = 7.0 Hz, 3H).
^{19}F NMR (376 MHz, $CDCl_3$) δ -40.14 (s, 3F), -110.38 (m, 1F).
^{13}C NMR (101 MHz, $CDCl_3$) δ 162.7 (d, *J* = 250.0 Hz), 133.7 (d, *J* = 8.4 Hz), 130.3 (q, *J* = 307.7 Hz), 118.4 (d, *J* = 3.5 Hz), 115.6 (d, *J* = 22.0 Hz), 87.1, 83.5, 30.8 (q, *J* = 2.7 Hz), 22.5.
HRMS (APCI) m/z calcd. for $C_{11}H_9F_4S$ $[M+H]^+$ 249.0356, found 249.0356.

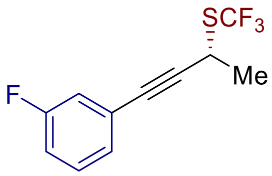

2h

(*R*)-(4-(3-Fluorophenyl)but-3-yn-2-yl)(trifluoromethyl)sulfane:

Prepared according to **General Procedure A**. The reaction mixture was purified by column chromatography on silica gel (eluent: hexanes, R_f = 0.45) to yield the product **2h** as a colorless oil (48.8 mg, 98% yield, 98% ee).
[α]$_D^{25}$ = +254.9 (c 0.32, $CHCl_3$)
HPLC analysis: Chiralcel OD-3 (hexanes/*i*-PrOH = 100/0, flow rate 1.0 mL/min, λ = 250 nm), t_R (major) = 4.93 min, t_R (minor) = 5.37 min.
^{1}H NMR (400 MHz, $CDCl_3$) δ 7.36 – 7.27 (m, 1H), 7.22 (d, *J* = 7.7 Hz, 1H), 7.17 – 7.11 (m, 1H), 7.09 – 7.02 (m, 1H), 4.35 (q, *J* = 7.1 Hz, 1H), 1.74 (d, *J* = 7.1 Hz, 3H).
^{19}F NMR (376 MHz, $CDCl_3$) δ -40.15 (s, 3F), -113.03 (m, 1F).
^{13}C NMR (101 MHz, $CDCl_3$) δ 162.3 (d, *J* = 246.7 Hz), 130.3 (q, *J* = 307.8 Hz), 129.9 (d, *J* = 8.7 Hz), 127.6 (d, *J* = 3.2 Hz), 124.1 (d, *J* = 9.5 Hz), 118.6 (d, *J* = 22.9 Hz), 116.0 (d, *J* = 21.2 Hz), 88.3, 83.3 (d, *J* = 3.4 Hz), 30.6 (q, *J* = 2.7 Hz), 22.3.
HRMS (ESI) m/z calcd. for $C_{11}H_9F_4S$ $[M+H]^+$ 249.0356, found 249.0359.

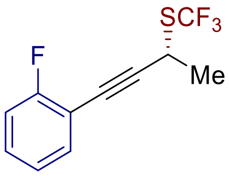

2i

(*R*)-(4-(2-Fluorophenyl)but-3-yn-2-yl)(trifluoromethyl)sulfane:

Prepared according to **General Procedure A**. The reaction mixture was purified by column chromatography on silica gel (eluent: hexanes, R_f = 0.40) to yield the product **2i** as a colorless oil (45.0 mg, 91% yield, 97% ee).
[α]$_D^{25}$ = +275.4 (c 0.28, $CHCl_3$)
HPLC analysis: Chiralcel OD-3 (hexanes/*i*-PrOH = 100/0, flow rate 1.0 mL/min, λ = 241 nm), t_R (major) = 6.10 min, t_R (minor) = 6.23 min.
^{1}H NMR (400 MHz, $CDCl_3$) δ 7.49 – 7.37 (m, 1H), 7.36 – 7.28 (m, 1H), 7.14 – 6.98 (m, 2H), 4.37 (q, *J* = 7.1 Hz, 1H), 1.74 (d, *J* = 7.1 Hz, 3H).
^{19}F NMR (376 MHz, $CDCl_3$) δ -40.14 (s, 3F), -110.03 (m, 1F).
^{13}C NMR (101 MHz, $CDCl_3$) δ 160.6 (d, *J* = 252.2 Hz), 131.4 (d, *J* = 1.3 Hz), 128.1 (d, *J* = 8.0 Hz), 128.0 (q, *J* = 307.8 Hz), 121.7 (d, *J* = 3.7 Hz), 113.3 (d, *J* = 20.9 Hz), 108.7 (d, *J* = 15.7 Hz), 90.2 (d, *J* = 3.3 Hz), 75.2, 28.5 (q, *J* = 2.7 Hz), 20.1.
HRMS (ESI) m/z calcd. for $C_{11}H_9F_4S$ $[M+H]^+$ 249.0356, found 249.0360.

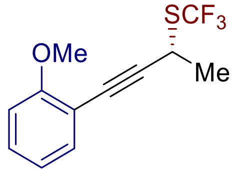

2j

(*R*)-(4-(2-Methoxyphenyl)but-3-yn-2-yl)(trifluoromethyl)sulfane:

Prepared according to **General Procedure A**. The reaction mixture was purified by column chromatography on silica gel (eluent: 20:1 hexanes/EtOAc, R_f = 0.40) to yield the product **2j** as a light yellow oil (35.5 mg, 68% yield, 99% ee).
[α]$_D$^{25} = +235.6 (c 0.33, $CHCl_3$)
HPLC analysis: Chiralcel OD-3 (hexanes/*i*-PrOH = 98/2, flow rate 1.0 mL/min, λ = 252 nm), t_R (major) = 4.84 min, t_R (minor) = 5.38 min.
^{1}H NMR (400 MHz, $CDCl_3$) δ 7.39 (dd, *J* = 7.5, 1.8 Hz, 1H), 7.29 (ddd, *J* = 8.3, 7.5, 1.8 Hz, 1H), 6.90 (td, *J* = 7.5, 1.0 Hz, 1H), 6.86 (dd, *J* = 8.3, 1.0 Hz, 1H), 4.41 (q, *J* = 7.0 Hz, 1H), 3.87 (s, 3H), 1.75 (d, *J* = 7.0 Hz, 3H).
^{19}F NMR (376 MHz, $CDCl_3$) δ -40.07 (s, 3F).
^{13}C NMR (101 MHz, $CDCl_3$) δ 157.9, 131.5, 128.1 (q, *J* = 307.8 Hz), 127.8, 118.1, 109.2, 108.5, 88.9, 78.7, 53.5, 28.9 (q, *J* = 2.6 Hz), 20.5.
HRMS (ESI) m/z calcd. for $C_{12}H_{12}OF_3S$ $[M+H]^+$ 261.0556, found 261.0550.

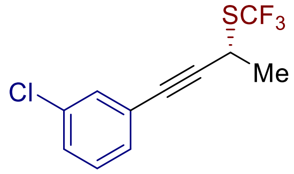

2k

(*R*)-(4-(3-Chlorophenyl)but-3-yn-2-yl)(trifluoromethyl)sulfane:

Prepared according to **General Procedure A**. The reaction mixture was purified by column chromatography on silica gel (eluent: hexanes, R_f = 0.55) to yield the product **2k** as a light yellow oil (49.1 mg, 93% yield, 97% ee).
[α]$_D$^{25} = +347.0 (c 0.12, $CHCl_3$)
HPLC analysis: Chiralcel OD-3 (hexanes/*i*-PrOH = 100/0, flow rate 1.0 mL/min, λ = 211 nm), t_R (major) = 5.34 min, t_R (minor) = 5.71 min.
^{1}H NMR (400 MHz, $CDCl_3$) δ 7.44 (t, *J* = 1.8 Hz, 1H), 7.37 – 7.31 (m, 2H), 7.29 – 7.25 (m, 1H), 4.35 (q, *J* = 7.0 Hz, 1H), 1.74 (d, *J* = 7.0 Hz, 3H).
^{19}F NMR (376 MHz, $CDCl_3$) δ -40.13 (s, 3F).
^{13}C NMR (101 MHz, $CDCl_3$) δ 134.2, 131.6, 130.3 (q, *J* = 307.8 Hz), 129.8, 129.6, 128.9, 124.0, 88.6, 83.1, 30.6 (q, *J* = 2.6 Hz), 22.3.
HRMS (APCI) m/z calcd. for $C_{11}H_9ClF_3S$ $[M+H]^+$ 265.0060, found 265.0057.

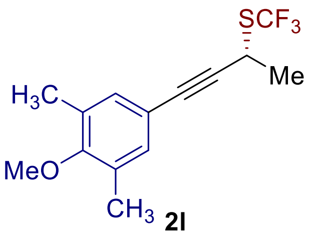

2l

(*R*)-(4-(4-Methoxy-3,5-dimethylphenyl)but-3-yn-2-yl)(trifluoromethyl)sulfane:

Prepared according to **General Procedure A**. The reaction mixture was purified by column chromatography on silica gel (eluent: 3:1 hexanes/CH_2Cl_2, R_f = 0.35) to yield the product **2l** as a colorless oil (51.1 mg, 89% yield, 95% ee).

[α]D^{25} = +168.9 (c 0.42, $CHCl_3$)

HPLC analysis: Chiralcel OD-3 (hexanes/*i*-PrOH = 99/1, flow rate 1.0 mL/min, λ = 253 nm), t_R (major) = 4.14 min, t_R (minor) = 4.45 min.

^{1}H NMR (400 MHz, $CDCl_3$) δ 7.10 (s, 2H), 4.33 (q, *J* = 7.0 Hz, 1H), 3.71 (s, 3H), 2.25 (s, 6H), 1.71 (d, *J* = 7.0 Hz, 3H).

^{19}F NMR (376 MHz, $CDCl_3$) δ -40.12 (s, 3F).

^{13}C NMR (101 MHz, $CDCl_3$) δ 157.6, 132.3, 131.1, 130.4 (q, *J* = 307.8 Hz), 117.5, 86.1, 84.5, 59.7, 31.0 (q, *J* = 2.6 Hz), 22.7, 15.9.

HRMS (ESI) m/z calcd. for $C_{14}H_{16}OF_3S$ $[M+H]^+$ 289.0869, found 289.0871.

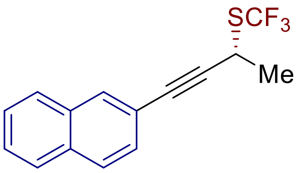

2m

(*R*)-(4-(Naphthalen-2-yl)but-3-yn-2-yl)(trifluoromethyl)sulfane:

Prepared according to **General Procedure A**. The reaction mixture was purified by column chromatography on silica gel (eluent: 20:1 hexanes/CH_2Cl_2, R_f = 0.50) to yield the product **2m** as a colorless oil (51.6 mg, 92% yield, 97% ee).

[α]D^{25} = +177.1 (c 0.54, $CHCl_3$)

HPLC analysis: Chiralcel OD-3 (hexanes/*i*-PrOH = 99/1, flow rate 1.0 mL/min, λ = 247 nm), t_R (major) = 6.31 min, t_R (minor) = 7.63 min.

^{1}H NMR (400 MHz, $CDCl_3$) δ 7.97 (s, 1H), 7.86 – 7.73 (m, 3H), 7.57 – 7.44 (m, 3H), 4.41 (qd, *J* = 7.0, 2.1 Hz, 1H), 1.78 (d, *J* = 7.0 Hz, 3H).

^{19}F NMR (376 MHz, $CDCl_3$) δ -40.04 (d, *J* = 4.0 Hz, 3F).

^{13}C NMR (101 MHz, $CDCl_3$) δ 130.6, 130.5, 129.4, 128.0 (q, *J* = 307.9 Hz), 125.9, 125.7, 125.4, 124.5, 124.2, 117.2, 85.2, 82.6, 28.6 (q, *J* = 2.7 Hz), 20.3.

HRMS (ESI) m/z calcd. for $C_{15}H_{12}F_3S$ $[M+H]^+$ 281.0606, found 281.0608.

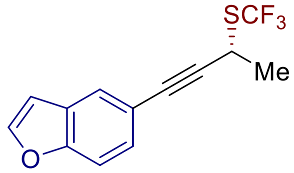

2n

(*R*)-5-(3-((Trifluoromethyl)thio)but-1-yn-1-yl)benzofuran:

Prepared according to **General Procedure A**. The reaction mixture was purified by column chromatography on silica gel (eluent: 2:1 hexanes/CH_2Cl_2, R_f = 0.60) to yield the product **2n** as a colorless oil (51.3 mg, 95% yield, 98% ee).

[α]$_D^{25}$ = +127.8 (c 0.21, $CHCl_3$)

HPLC analysis: Chiralcel OJ-3 (hexanes/*i*-PrOH = 99/1, flow rate 1.0 mL/min, λ = 230 nm), t_R (major) = 9.09 min, t_R (minor) = 11.10 min.

^{1}H NMR (400 MHz, $CDCl_3$) δ 7.70 (d, J = 1.7 Hz, 1H), 7.64 (d, J = 2.2 Hz, 1H), 7.44 (d, J = 8.5 Hz, 1H), 7.36 (dd, J = 8.5, 1.7 Hz, 1H), 6.78 – 6.70 (m, 1H), 4.37 (q, J = 7.0 Hz, 1H), 1.74 (d, J = 7.0 Hz, 3H).

^{19}F NMR (376 MHz, $CDCl_3$) δ -40.08 (s, 3F).

^{13}C NMR (101 MHz, $CDCl_3$) δ 154.7, 145.9, 130.4 (q, J = 307.8 Hz), 128.1, 127.6, 124.9, 116.8, 111.5, 106.5, 85.9, 84.9, 31.0 (q, J = 2.7 Hz), 22.7.

HRMS (ESI) m/z calcd. for $C_{13}H_{11}OF_3S$ $[M+H]^+$ 271.0399, found 271.0401.

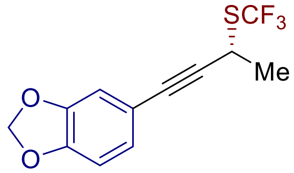

2o

(*R*)-5-(3-((Trifluoromethyl)thio)but-1-yn-1-yl)benzo[d][1,3]dioxole:

Prepared according to **General Procedure A**. The reaction mixture was purified by column chromatography on silica gel (eluent: 20:1 hexanes/EtOAc, R_f = 0.50) to yield the product **2o** as a colorless oil (51.1 mg, 93% yield, 97% ee).

[α]$_D^{25}$ = +221.0 (c 0.49, $CHCl_3$)

HPLC analysis: Chiralcel OD-3 (hexanes/*i*-PrOH = 99/1, flow rate 1.0 mL/min, λ = 265 nm), t_R (major) = 5.11 min, t_R (minor) = 5.43 min.

^{1}H NMR (400 MHz, $CDCl_3$) δ 6.95 (dd, J = 8.0, 1.6 Hz, 1H), 6.86 (d, J = 1.6 Hz, 1H), 6.74 (d, J = 8.0 Hz, 1H), 5.97 (s, 2H), 4.32 (q, J = 7.0 Hz, 1H), 1.70 (d, J = 7.0 Hz, 3H).

^{19}F NMR (376 MHz, $CDCl_3$) δ -40.12 (s, 3F).

^{13}C NMR (101 MHz, $CDCl_3$) δ 148.2, 147.4, 130.3 (q, J = 307.7 Hz), 126.4, 115.5, 111.7, 108.4, 101.4, 85.6, 84.5, 30.9 (q, J = 2.7 Hz), 22.6.

HRMS (ESI) m/z calcd. for $C_{12}H_{10}O_2F_3S$ $[M+H]^+$ 275.0348, found 275.0352.

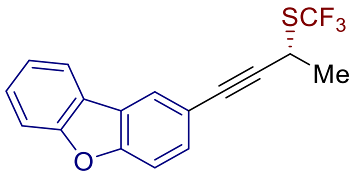

2p

(*R*)-2-(3-((Trifluoromethyl)thio)but-1-yn-1-yl)dibenzo[b,d]furan:

Prepared according to **General Procedure A**. The reaction mixture was purified by column chromatography on silica gel (eluent: 20:1 hexanes/EtOAc, R_f = 0.65) to yield the product **2p** as a colorless oil (60.0 mg, 94% yield, 98% ee).

$[\alpha]_D^{25}$ = +52.0 (c 0.32, $CHCl_3$)

HPLC analysis: Chiralcel OD-3 (hexanes/*i*-PrOH = 99/1, flow rate 1.0 mL/min, λ = 242 nm), t_R (minor) = 6.81 min, t_R (major) = 8.66 min.

^{1}H NMR (400 MHz, $CDCl_3$) δ 8.03 (s, 1H), 7.93 (d, *J* = 7.7 Hz, 1H), 7.57 (d, *J* = 8.2 Hz, 1H), 7.55 – 7.44 (m, 3H), 7.36 (t, *J* = 7.7 Hz, 1H), 4.41 (q, *J* = 7.0 Hz, 1H), 1.77 (d, *J* = 7.0 Hz, 3H).

^{19}F NMR (376 MHz, $CDCl_3$) δ -40.04 (s, 3F).

^{13}C NMR (101 MHz, $CDCl_3$) δ 156.6, 155.9, 130.9, 130.4 (q, *J* = 307.8 Hz), 127.7, 124.5, 124.3, 123.5, 123.1, 120.8, 116.8, 111.82, 111.79, 86.4, 84.7, 31.0 (q, *J* = 2.6 Hz), 22.7.

HRMS (ESI) m/z calcd. for $C_{17}H_{12}OF_3S$ $[M+H]^+$ 321.0556, found 321.0557.

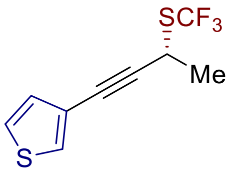

2q

(*R*)-3-(3-((Trifluoromethyl)thio)but-1-yn-1-yl)thiophene:

Prepared according to **General Procedure A**. The reaction mixture was purified by column chromatography on silica gel (eluent: hexanes, R_f = 0.35) to yield the product **2q** as a colorless oil (43.2 mg, 91% yield, 96% ee).

$[\alpha]_D^{25}$ = +302.6 (c 0.08, $CHCl_3$)

HPLC analysis: Chiralcel OD-3 (hexanes/*i*-PrOH = 100/0, flow rate 1.0 mL/min, λ = 253 nm), t_R (major) = 7.02 min, t_R (minor) = 7.48 min.

^{1}H NMR (400 MHz, $CDCl_3$) δ 7.45 (dd, *J* = 3.0, 1.2 Hz, 1H), 7.29 – 7.24 (m, 1H), 7.09 (dd, *J* = 5.0, 1.2 Hz, 1H), 4.33 (q, *J* = 7.0 Hz, 1H), 1.71 (d, *J* = 7.0 Hz, 3H).

^{19}F NMR (376 MHz, $CDCl_3$) δ -40.14 (s, 3F).

^{13}C NMR (101 MHz, $CDCl_3$) δ 130.3 (q, J = 307.8 Hz), 129.8, 129.3, 125.4, 121.3, 86.9, 79.8, 30.9 (q, *J* = 2.6 Hz), 22.6.

HRMS (ESI) m/z calcd. for $C_9H_8F_3S_2$ $[M+H]^+$ 237.0014, found 237.0019.

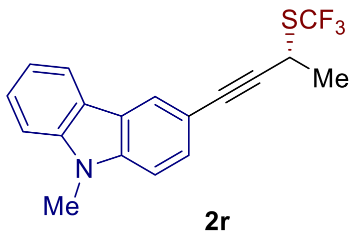

2r

(*R*)-9-Methyl-3-(3-((trifluoromethyl)thio)but-1-yn-1-yl)-9*H*-carbazole:

Prepared according to **General Procedure A**. The reaction mixture was purified by column chromatography on silica gel (eluent: 2:1 hexanes/CH_2Cl_2, R_f = 0.25) to yield the product **2r** as a colorless oil (43.6 mg, 65% yield, 92% ee).

[α]D²⁵ = +38.5 (c 0.12, $CHCl_3$)

HPLC analysis: Chiralcel OD-3 (hexanes/*i*-PrOH = 98/2, flow rate 1.0 mL/min, λ = 280 nm), t_R (minor) = 13.36 min, t_R (major) = 21.09 min.

^{1}H NMR (400 MHz, $CDCl_3$) δ 8.18 (s, 1H), 8.07 (d, *J* = 7.8 Hz, 1H), 7.57 – 7.47 (m, 2H), 7.40 (d, *J* = 8.2 Hz, 1H), 7.32 (d, *J* = 8.5 Hz, 1H), 7.29 – 7.23 (m, 1H), 4.43 (q, *J* = 7.0 Hz, 1H), 3.84 (s, 3H), 1.78 (d, *J* = 7.0 Hz, 3H).

^{19}F NMR (376 MHz, $CDCl_3$) δ -40.03 (s, 3F).

^{13}C NMR (101 MHz, $CDCl_3$) δ 141.4, 140.8, 130.5 (q, *J* = 307.8 Hz),129.4, 126.2, 124.2, 122.7, 122.3, 120.5, 119.5, 112.3, 108.7, 108.4, 85.9, 85.2, 31.4 (q, *J* = 2.6 Hz), 29.2, 22.9.

HRMS (ESI) m/z calcd. for $C_{18}H_{15}NF_3S$ $[M+H]^+$ 334.0872, found 334.0875.

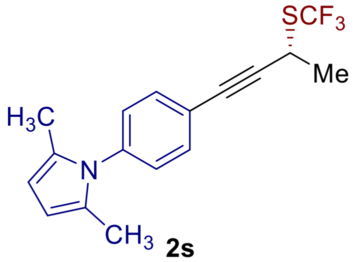

2s

(*R*)-2,5-Dimethyl-1-(4-(3-((trifluoromethyl)thio)but-1-yn-1-yl)phenyl)-1*H*-pyrrole:

Prepared according to **General Procedure A**. The reaction mixture was purified by column chromatography on silica gel (eluent: 20:1 hexanes/EtOAc, R_f = 0.50) to yield the product **2s** as a light yellow solid (59.7 mg, 92% yield, 99% ee).

m.p. 61.0 °C – 62.0 °C

[α]D²⁵ = +145.2 (c 0.48, $CHCl_3$)

HPLC analysis: Chiralcel OD-3 (hexanes/*i*-PrOH = 99/1, flow rate 1.0 mL/min, λ = 268 nm), t_R (major) = 6.57 min, t_R (minor) = 6.91 min.

^{1}H NMR (400 MHz, $CDCl_3$) δ 7.52 (d, *J* = 8.4 Hz, 2H), 7.17 (d, *J* = 8.4 Hz, 2H), 5.91 (s, 2H), 4.37 (q, *J* = 7.1 Hz, 1H), 2.03 (s, 6H), 1.74 (d, *J* = 7.1 Hz, 3H).

^{19}F NMR (376 MHz, $CDCl_3$) δ -40.07 (s, 3F).

^{13}C NMR (101 MHz, $CDCl_3$) δ 139.2, 132.5, 130.3 (q, *J* = 307.8 Hz), 128.7, 128.2, 121.7, 106.2, 88.4, 83.7, 30.8 (q, *J* = 2.7 Hz), 22.4, 13.0.

HRMS (ESI) m/z calcd. for $C_{17}H_{17}NF_3S$ $[M+H]^+$ 324.1028, found 324.1032.

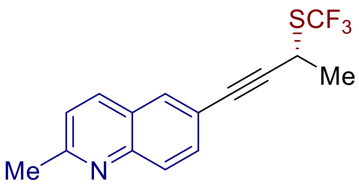

2t

(*R*)-2-Methyl-6-(3-((trifluoromethyl)thio)but-1-yn-1-yl)quinoline:

Prepared according to **General Procedure A**. The reaction mixture was purified by column chromatography on silica gel (eluent: 20:1 CH_2Cl_2/MeOH, R_f = 0.50) to yield the product **2t** as a yellow oil (53.3 mg, 90% yield, 97% ee).

$[\alpha]_D^{25}$ = +189.8 (c 0.25, $CHCl_3$)

HPLC analysis: Chiralcel AD-3 (hexanes/*i*-PrOH = 98/2, flow rate 0.8 mL/min, λ = 246 nm), t_R (major) = 8.03 min, t_R (minor) = 8.45 min.

^{1}H NMR (400 MHz, $CDCl_3$) δ 8.02 – 7.90 (m, 2H), 7.86 (d, *J* = 1.9 Hz, 1H), 7.65 (dd, *J* = 8.4, 1.9 Hz, 1H), 7.28 (d, *J* = 8.4 Hz, 1H), 4.38 (q, *J* = 7.0 Hz, 1H), 2.73 (s, 3H), 1.75 (d, *J* = 7.0 Hz, 3H).

^{19}F NMR (376 MHz, $CDCl_3$) δ -40.07 (s, 3F).

^{13}C NMR (101 MHz, $CDCl_3$) δ 160.0, 147.4, 135.8, 132.0, 131.2, 130.3 (q, *J* = 307.8 Hz), 128.8, 126.1, 122.7, 119.6, 88.1, 84.3, 30.9 (q, *J* = 2.7 Hz), 25.5, 22.5.

HRMS (ESI) m/z calcd. for $C_{15}H_{13}NF_3S$ $[M+H]^+$ 296.0715, found 296.0720

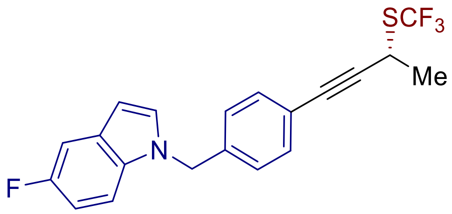

2u

(*R*)-5-Fluoro-1-(4-(3-((trifluoromethyl)thio)but-1-yn-1-yl)benzyl)-1H-indole:

Prepared according to **General Procedure A**. The reaction mixture was purified by column chromatography on silica gel (eluent: 20:1 hexanes/EtOAc, R_f = 0.25) to yield the product **2u** as a light yellow oil (63.3 mg, 84% yield, 97% ee).

$[\alpha]_D^{25}$ = +173.4 (c 0.45, $CHCl_3$)

HPLC analysis: Chiralcel OD-3 (hexanes/*i*-PrOH = 99/1, flow rate 1.0 mL/min, λ = 250 nm), t_R (major) = 19.38 min, t_R (minor) = 20.41 min.

^{1}H NMR (400 MHz, $CDCl_3$) δ 7.36 (d, *J* = 7.9 Hz, 2H), 7.30 (dt, *J* = 9.5, 2.1 Hz, 1H), 7.16 (d, *J* = 3.2 Hz, 1H), 7.11 (dd, *J* = 9.5, 4.3 Hz, 1H), 7.02 (d, *J* = 7.9 Hz, 2H), 6.91 (tt, *J* = 9.0, 2.1 Hz, 1H), 6.52 (d, *J* = 3.2 Hz, 1H), 5.29 (s, 2H), 4.33 (q, *J* = 7.1 Hz, 1H), 1.71 (d, *J* = 7.1 Hz, 3H).

^{19}F NMR (376 MHz, $CDCl_3$) δ -40.09 (d, *J* = 4.2 Hz, 3F), -125.08 (m, 1F).

^{13}C NMR (101 MHz, $CDCl_3$) δ 157.9 (d, *J* = 234.5 Hz), 137.8, 132.8, 132.2, 130.3 (q, *J* = 307.8 Hz), 129.8, 129.0 (d, *J* = 10.3 Hz), 126.6, 121.8, 110.3 (d, *J* = 9.8 Hz), 110.2 (d, *J* = 26.4 Hz), 105.8 (d, *J* = 23.4 Hz), 101.9 (d, *J* = 4.8 Hz), 87.8, 84.0, 50.2, 30.8 (q, *J* = 2.8 Hz), 22.5.

HRMS (ESI) m/z calcd. for $C_{20}H_{16}NF_4S$ $[M+H]^+$ 378.0934, found 378.0924.

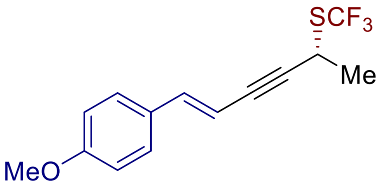

2v

(*R*, *E*)-(6-(4-Methoxyphenyl)hex-5-en-3-yn-2-yl)(trifluoromethyl)sulfane:

Prepared according to **General Procedure A** (35 °C instead of r.t.). The reaction mixture was purified by column chromatography on silica gel (eluent: 20:1 hexanes/EtOAc, R_f = 0.45) to yield the product **2v** as a colorless oil (45.2 mg, 79% yield, 92% ee).

[α]$_D^{25}$ = +106.1 (c 0.48, $CHCl_3$)

HPLC analysis: Chiralcel IB (hexanes/*i*-PrOH = 99/1, flow rate 0.6 mL/min, λ = 294 nm), t_R (major) = 7.92 min, t_R (minor) = 9.39 min.

^{1}H NMR (400 MHz, $CDCl_3$) δ 7.32 (d, J = 8.7 Hz, 2H), 6.91 (d, *J* = 16.3 Hz, 1H), 6.86 (d, *J* = 8.7 Hz, 2H), 6.01 (dd, *J* = 16.3, 2.1 Hz, 1H), 4.31 (qd, *J* = 7.0, 2.1 Hz, 1H), 3.82 (s, 3H), 1.69 (d, *J* = 7.0 Hz, 3H).

^{19}F NMR (376 MHz, $CDCl_3$) δ -40.15 (s, 3F).

^{13}C NMR (101 MHz, $CDCl_3$) δ 160.2, 141.9, 130.4 (q, *J* = 307.8 Hz), 128.8, 127.7, 114.2, 104.7, 88.5, 84.2, 55.3, 31.2 (q, *J* = 2.7 Hz), 22.8.

HRMS (ESI) m/z calcd. for $C_{14}H_{14}OF_3S$ $[M+H]^+$ 287.0712, found 287.0713.

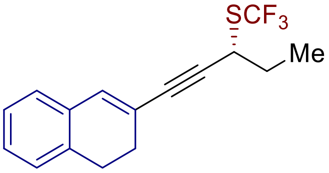

2w

(*R*)-(1-(3,4-Dihydronaphthalen-2-yl)pent-1-yn-3-yl)(trifluoromethyl)sulfane:

Prepared according to **General Procedure A** (35 °C instead of r.t.). The reaction mixture was purified by column chromatography on silica gel (eluent: hexanes, R_f = 0.45) to yield the product **2w** as a colorless oil (43.4 mg, 73% yield, 99% ee).

[α]$_D^{25}$ = +215.2 (c 0.63, $CHCl_3$)

HPLC analysis: Chiralcel IB (hexanes/*i*-PrOH = 100/0, flow rate 0.6 mL/min, λ = 228 nm), t_R (major) = 7.86 min, t_R (minor) = 9.89 min.

^{1}H NMR (400 MHz, $CDCl_3$) δ 7.20 – 7.09 (m, 3H), 7.07 – 7.02 (m, 1H), 6.78 (s, 1H), 4.20 (dd, *J* = 7.7, 5.5 Hz, 1H), 2.85 (t, *J* = 8.2 Hz, 2H), 2.43 (td, *J* = 8.2, 1.6 Hz, 2H), 2.04 – 1.83 (m, 2H), 1.17 (t, *J* = 7.3 Hz, 3H).

^{19}F NMR (376 MHz, $CDCl_3$) δ -39.88 (s, 3F).

^{13}C NMR (101 MHz, $CDCl_3$) δ 134.9, 133.9, 133.5, 130.5 (q, *J* = 307.8 Hz), 127.9, 127.5, 126.7, 126.5, 120.3, 88.0, 86.8, 37.7 (q, *J* = 2.3 Hz), 29.1, 27.6, 27.5, 11.2.

HRMS (ESI) m/z calcd. for $C_{16}H_{16}F_3S$ $[M+H]^+$ 297.0919, found 297.0921.

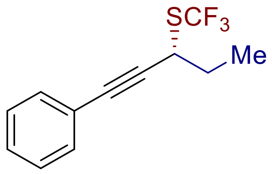

4a

(*R*)-(1-Phenylpent-1-yn-3-yl)(trifluoromethyl)sulfane:

Prepared according to **General Procedure A** (35 °C instead of r.t.). The reaction mixture was purified by column chromatography on silica gel (eluent: hexanes, R_f = 0.60) to yield the product **4a** as a colorless oil (43.9 mg, 90% yield, 99% ee).
$[\alpha]_D^{25}$ = +317.9 (c 0.34, $CHCl_3$)
HPLC analysis: Chiralcel OD-3 (hexanes/*i*-PrOH = 100/0, flow rate 1.0 mL/min, λ = 243 nm), t_R (major) = 6.04 min, t_R (minor) = 6.72 min.
^{1}H NMR (400 MHz, $CDCl_3$) δ 7.48 – 7.40 (m, 2H), 7.38 – 7.28 (m, 3H), 4.22 (dd, *J* = 7.4, 5.6 Hz, 1H), 2.16 – 1.80 (m, 2H), 1.20 (t, *J* = 7.4 Hz, 3H).
^{19}F NMR (376 MHz, $CDCl_3$) δ -39.91 (s, 3F).
^{13}C NMR (101 MHz, $CDCl_3$) δ 131.7, 130.5 (q, *J* = 307.5 Hz), 128.6, 128.3, 122.5, 86.2, 85.5, 37.4 (q, *J* = 2.3 Hz), 29.1, 11.2.
HRMS (ESI) m/z calcd. for $C_{12}H_{12}F_3S$ $[M+H]^+$ 245.0606, found 245.0603.

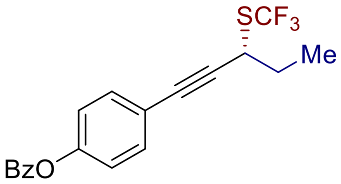

4b

(*R*)-4-(3-((Trifluoromethyl)thio)pent-1-yn-1-yl)phenyl benzoate:

Prepared according to **General Procedure A** (35 °C instead of r.t.). The reaction mixture was purified by column chromatography on silica gel (eluent: 2:1 hexanes/CH_2Cl_2, R_f = 0.35) to yield the product **4b** as a colorless oil (66.8 mg, 92% yield, 98% ee).
$[\alpha]_D^{25}$ = +95.3 (c 1.06, $CHCl_3$)
HPLC analysis: Chiralcel AD-3 (hexanes/*i*-PrOH = 99/1, flow rate 0.8 mL/min, λ = 246 nm), t_R (major) = 10.05 min, t_R (minor) = 10.27 min.
^{1}H NMR (400 MHz, $CDCl_3$) δ 8.20 (d, *J* = 7.5 Hz, 2H), 7.65 (t, *J* = 7.5 Hz, 1H), 7.56 – 7.46 (m, 4H), 7.19 (d, *J* = 8.6 Hz, 2H), 4.22 (dd, *J* = 7.6, 5.6 Hz, 1H), 2.12 – 1.87 (m, 2H), 1.20 (t, *J* = 7.3 Hz, 3H).
^{19}F NMR (376 MHz, $CDCl_3$) δ -39.88 (s, 3F).
^{13}C NMR (101 MHz, $CDCl_3$) δ 164.9, 151.1, 133.8, 132.9, 130.5 (q, *J* = 307.5 Hz), 130.2, 129.3, 128.6, 121.9, 120.2, 86.4, 84.7, 37.4 (q, *J* = 2.4 Hz), 29.0, 11.3.
HRMS (ESI) m/z calcd. for $C_{19}H_{16}O_2F_3S$ $[M+H]^+$ 365.0818, found 365.0810.

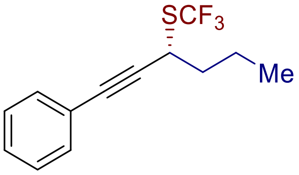

4c

(*R*)-(1-Phenylhex-1-yn-3-yl)(trifluoromethyl)sulfane:

Prepared according to **General Procedure A** (35 °C instead of r.t.). The reaction mixture was purified by column chromatography on silica gel (eluent: hexanes, R_f = 0.60) to yield the product **4c** as a colorless oil (38.4 mg, 74% yield, 95% ee).
$[\alpha]_D^{25}$ = +123.2 (c 0.42, $CHCl_3$)
HPLC analysis: Chiralcel OD-3 (hexanes/*i*-PrOH = 100/0, flow rate 1.0 mL/min, λ = 243 nm), t_R (major) = 5.48 min, t_R (minor) = 6.46 min.
^{1}H NMR (400 MHz, $CDCl_3$) δ 7.49 – 7.39 (m, 2H), 7.37 – 7.29 (m, 3H), 4.25 (t, *J* = 6.9 Hz, 1H), 1.92 (q, *J* = 7.4 Hz, 2H), 1.77 – 1.56 (m, 2H), 1.01 (t, *J* = 7.4 Hz, 3H).
^{19}F NMR (376 MHz, $CDCl_3$) δ -39.92 (s, 3F).
^{13}C NMR (101 MHz, $CDCl_3$) δ 131.7, 130.5 (q, *J* = 307.5 Hz), 128.6, 128.3, 122.5, 86.6, 85.3, 37.6, 35.7 (q, *J* = 2.3 Hz), 20.2, 13.4.
HRMS (ESI) m/z calcd. for $C_{13}H_{14}F_3S$ $[M+H]^+$ 259.0763, found 259.0759.

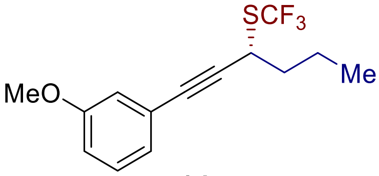

4d

(*R*)-(1-(3-Methoxyphenyl)hex-1-yn-3-yl)(trifluoromethyl)sulfane:

Prepared according to **General Procedure A** (35 °C instead of r.t.). The reaction mixture was purified by column chromatography on silica gel (eluent: 2:1 hexanes/CH_2Cl_2, R_f = 0.55) to yield the product **4d** as a colorless oil (42.4 mg, 74% yield, 96% ee).
$[\alpha]_D^{25}$ = +93.5 (c 0.60, $CHCl_3$)
HPLC analysis: Chiralcel OD-3 (hexanes/*i*-PrOH = 99/1, flow rate 1.0 mL/min, λ = 245 nm), t_R (major) = 3.83 min, t_R (minor) = 4.03 min.
^{1}H NMR (400 MHz, $CDCl_3$) δ 7.22 (t, *J* = 7.9 Hz, 1H), 7.03 (d, *J* = 7.6 Hz, 1H), 6.95 (s, 1H), 6.91 – 6.85 (m, 1H), 4.24 (t, *J* = 7.0 Hz, 1H), 3.80 (s, 3H), 1.91 (q, *J* = 7.0 Hz, 2H), 1.76 – 1.57 (m, 2H), 1.01 (t, *J* = 7.3 Hz, 3H).
^{19}F NMR (376 MHz, $CDCl_3$) δ -39.92 (s, 3F).
^{13}C NMR (101 MHz, $CDCl_3$) δ 159.3, 130.5 (q, *J* = 307.5 Hz), 129.4, 124.3, 123.5, 116.6, 115.1, 86.4, 85.2, 55.3, 37.6, 35.6 (q, *J* = 2.3 Hz), 20.2, 13.4.
HRMS (ESI) m/z calcd. for $C_{14}H_{16}OF_3S$ $[M+H]^+$ 289.0869, found 289.0861.

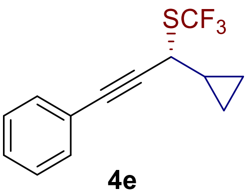

4e

(*R*)-(1-Cyclopropyl-3-phenylprop-2-yn-1-yl)(trifluoromethyl)sulfane:

Prepared according to **General Procedure A** (35 °C instead of r.t.). The reaction mixture was purified by column chromatography on silica gel (eluent: hexanes, R_f = 0.45) to yield the product **4e** as a colorless oil (20.7 mg, 40% yield, 99% ee).
$[\alpha]_D^{25}$ = +502.0 (c 0.06, $CHCl_3$)
HPLC analysis: Chiralcel OD-3 (hexanes/*i*-PrOH = 100/0, flow rate 1.0 mL/min, λ = 245 nm), t_R (minor) = 5.92 min, t_R (major) = 6.35 min.
^{1}H NMR (400 MHz, $CDCl_3$) δ 7.48 – 7.38 (m, 2H), 7.36 – 7.28 (m, 3H), 4.26 (d, *J* = 6.4 Hz, 1H), 1.40 – 1.24 (m, 1H), 0.79 – 0.57 (m, 4H).
^{19}F NMR (376 MHz, $CDCl_3$) δ -39.65 (s, 3F).
^{13}C NMR (101 MHz, $CDCl_3$) δ 131.8, 130.4 (q, *J* = 307.9 Hz), 128.7, 128.3, 122.3, 85.8, 83.9, 39.9 (q, *J* = 2.3 Hz), 14.7, 5.3, 3.9.
HRMS (APCI) m/z calcd. for $C_{13}H_{12}F_3S$ $[M+H]^+$ 257.0606, found 257.0599.

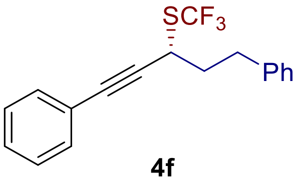

4f

(*R*)-(1,5-Diphenylpent-1-yn-3-yl)(trifluoromethyl)sulfane:

Prepared according to **General Procedure A** (35 °C instead of r.t.). The reaction mixture was purified by column chromatography on silica gel (eluent: hexanes, R_f = 0.30) to yield the product **4f** as a colorless oil (31.0 mg, 48% yield, 96% ee).
$[\alpha]_D^{25}$ = +130.0 (c 0.34, $CHCl_3$)
HPLC analysis: Chiralcel OD-3 (hexanes/*i*-PrOH = 100/0, flow rate 1.0 mL/min, λ = 244 nm), t_R (minor) = 18.75 min, t_R (major) = 22.10 min.
^{1}H NMR (400 MHz, $CDCl_3$) δ 7.53 – 7.45 (m, 2H), 7.42 – 7.33 (m, 5H), 7.29 – 7.18 (m, 3H), 4.23 (t, *J* = 7.0 Hz, 1H), 3.06 – 2.87 (m, 2H), 2.37 – 2.21 (m, 2H).
^{19}F NMR (376 MHz, $CDCl_3$) δ -39.67 (s, 3F).
^{13}C NMR (101 MHz, $CDCl_3$) δ 140.1, 131.8, 130.4 (q, *J* = 307.9 Hz), 128.69, 128.65, 128.5, 128.4, 126.4, 122.4, 86.1, 85.9, 37.2, 35.3 (q, *J* = 2.4 Hz), 32.9.
HRMS (ESI) m/z calcd. for $C_{18}H_{16}F_3S$ $[M+H]^+$ 321.0919, found 321.0913.

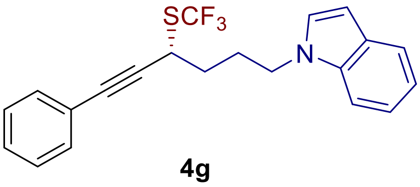

4g

(*R*)-1-(6-Phenyl-4-((trifluoromethyl)thio)hex-5-yn-1-yl)-1H-indole:

Prepared according to **General Procedure A** (35 °C instead of r.t.). The reaction mixture was purified by column chromatography on silica gel (eluent: 2:1 hexanes/CH_2Cl_2, R_f = 0.55) to yield the product **4g** as a light yellow oil (56.4 mg, 76% yield, 96% ee).
$[\alpha]_D^{25}$ = +47.3 (c 0.71, $CHCl_3$)
HPLC analysis: Chiralcel IB (hexanes/*i*-PrOH = 80/20, flow rate 0.8 mL/min, λ = 222 nm), t_R (major) = 8.23 min, t_R (minor) = 16.64 min.
^{1}H NMR (400 MHz, $CDCl_3$) δ 7.66 (d, *J* = 7.8 Hz, 1H), 7.42 – 7.28 (m, 6H), 7.22 (t, *J* = 7.6 Hz, 1H), 7.16 – 7.05 (m, 2H), 6.53 (d, *J* = 3.1 Hz, 1H), 4.31 – 4.14 (m, 3H), 2.33 – 2.09 (m, 2H), 2.05 – 1.80 (m, 2H).
^{19}F NMR (376 MHz, $CDCl_3$) δ -39.83 (s, 3F).
^{13}C NMR (101 MHz, $CDCl_3$) δ 135.9, 131.8, 130.3 (q, *J* = 307.9 Hz), 128.8, 128.7, 128.4, 127.6, 122.1, 121.7, 121.1, 119.5, 109.2, 101.5, 86.0, 85.6, 45.6, 35.5 (q, *J* = 2.5 Hz), 32.9, 27.5.
HRMS (ESI) m/z calcd. for $C_{21}H_{19}NF_3S$ $[M+H]^+$ 374.1185, found 374.1174.

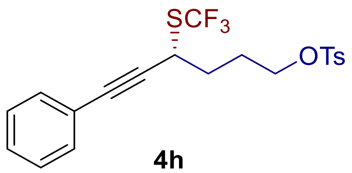

4h

(*R*)-6-Phenyl-4-((trifluoromethyl)thio)hex-5-yn-1-yl 4-methylbenzenesulfonate:

Prepared according to **General Procedure A** (35 °C instead of r.t.). The reaction mixture was purified by column chromatography on silica gel (eluent: 1:2 hexanes/CH_2Cl_2, R_f = 0.50) to yield the product **4h** as a colorless oil (80.3 mg, 94% yield, 95% ee).
$[\alpha]_D^{25}$ = +99.0 (c 1.01, $CHCl_3$)
HPLC analysis: Chiralcel OD-3 (hexanes/*i*-PrOH = 90/10, flow rate 1.0 mL/min, λ = 230 nm), t_R (major) = 8.02 min, t_R (minor) = 9.85 min.
^{1}H NMR (400 MHz, $CDCl_3$) δ 7.80 (d, *J* = 8.2 Hz, 2H), 7.43 – 7.38 (m, 2H), 7.37 – 7.28 (m, 5H), 4.26 – 4.16 (m, 1H), 4.14 – 4.02 (m, 2H), 2.44 (s, 3H), 2.08 – 1.88 (m, 4H).
^{19}F NMR (376 MHz, $CDCl_3$) δ -39.83 (s, 3F).
^{13}C NMR (101 MHz, $CDCl_3$) δ 144.9, 132.9, 131.7, 130.2 (q, *J* = 307.9 Hz), 129.9, 128.8, 128.4, 127.9, 122.0, 86.1, 85.3, 69.3, 35.2 (q, *J* = 2.4 Hz), 31.7, 26.3, 21.6.
HRMS (ESI) m/z calcd. for $C_{20}H_{20}O_3F_3S_2$ $[M+H]^+$ 429.0801, found 429.0789.

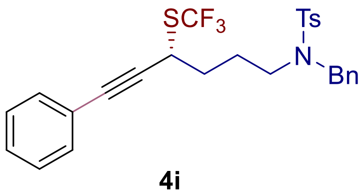

4i

(*R*)-*N*-Benzyl-4-methyl-*N*-(6-phenyl-4-((trifluoromethyl)thio)hex-5-yn-1-yl)benzenesulfonamide:

According to **General Procedure A** (35 °C instead of r.t.). The reaction mixture was purified by column chromatography on silica gel (eluent: 1:2 hexanes/CH_2Cl_2, R_f = 0.30) to yield the product **4i** as a yellow solid (98.5 mg, 95% yield, 97% ee).
m.p. 82.0 °C – 83.0 °C
$[\alpha]_D^{25}$ = +50.6 (c 0.58, $CHCl_3$)
HPLC analysis: Chiralcel OD-3 (hexanes/*i*-PrOH = 90/10, flow rate 1.0 mL/min, λ = 240 nm), t_R (major) = 11.62 min, t_R (minor) = 14.32 min.
^{1}H NMR (400 MHz, $CDCl_3$) δ 7.73 (d, *J* = 8.1 Hz, 2H), 7.41 – 7.16 (m, 12H), 4.42 – 4.19 (m, 2H), 4.03 (dd, *J* = 7.8, 5.6 Hz, 1H), 3.17 (t, *J* = 7.1 Hz, 2H), 2.42 (s, 3H), 1.82 – 1.57 (m, 4H).
^{19}F NMR (376 MHz, $CDCl_3$) δ -39.84 (s, 3F).
^{13}C NMR (101 MHz, $CDCl_3$) δ 143.4, 136.7, 136.2, 131.7, 130.3 (q, *J* = 307.8 Hz), 129.8, 128.7, 128.4, 128.3, 127.9, 127.2, 122.2, 85.8, 85.6, 52.4, 47.4, 35.3 (q, *J* = 2.3 Hz), 32.5, 25.5, 21.5.
HRMS (ESI) m/z calcd. for $C_{27}H_{27}O_2NF_3S_2$ $[M+H]^+$ 518.1430, found 518.1420.

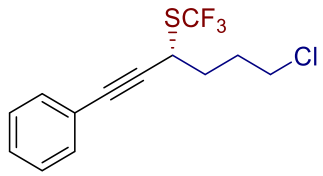

4j

(*R*)-(6-Chloro-1-phenylhex-1-yn-3-yl)(trifluoromethyl)sulfane:

Prepared according to **General Procedure A** (35 °C instead of r.t.). The reaction mixture was purified by column chromatography on silica gel (eluent: hexanes, R_f = 0.35) to yield the product **4j** as a light yellow oil (41.8 mg, 71% yield, 96% ee).
$[\alpha]_D^{25}$ = +137.5 (c 0.35, $CHCl_3$)
HPLC analysis: Chiralcel OJ-3 (hexanes/*i*-PrOH = 100/0, flow rate 1.0 mL/min, λ = 242 nm), t_R (minor) = 10.46 min, t_R (major) = 11.56 min.
^{1}H NMR (400 MHz, $CDCl_3$) δ 7.47 – 7.40 (m, 2H), 7.37 – 7.28 (m, 3H), 4.39 – 4.19 (m, 1H), 3.72 – 3.49 (m, 2H), 2.44 – 1.94 (m, 4H).
^{19}F NMR (376 MHz, $CDCl_3$) δ -39.85 (s, 3F).
^{13}C NMR (101 MHz, $CDCl_3$) δ 131.8, 130.3 (q, *J* = 307.8 Hz), 128.8, 128.4, 122.1, 85.9, 85.6, 43.9, 35.3 (q, *J* = 2.4 Hz), 32.9, 29.7.
HRMS (ESI) m/z calcd. for $C_{13}H_{13}ClF_3S$ $[M+H]^+$ 293.0373, found 293.0367.

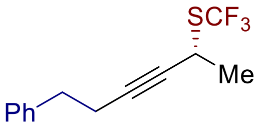

4k

(*R*)-(6-Phenylhex-3-yn-2-yl)(trifluoromethyl)sulfane:

Prepared according to **General Procedure A** (35 °C instead of r.t.). The reaction mixture was purified by column chromatography on silica gel (eluent: hexanes, R_f = 0.30) to yield the product **4k** as a colorless oil (26.9 mg, 52% yield, 86% ee).

[α]$_D^{25}$ = +185.1 (c 0.20, $CHCl_3$)
HPLC analysis: Chiralcel OD-3 (hexanes/*i*-PrOH = 100/0, flow rate 1.0 mL/min, λ = 211 nm), t_R (major) = 7.42 min, t_R (minor) = 10.36 min.
^{1}H NMR (400 MHz, $CDCl_3$) δ 7.34 – 7.27 (m, 2H), 7.25 – 7.18 (m, 3H), 4.10 (qt, *J* = 7.0, 2.3 Hz, 1H), 2.82 (t, *J* = 7.5 Hz, 2H), 2.49 (td, *J* = 7.5, 2.3 Hz, 2H), 1.59 (d, *J* = 7.0 Hz, 3H).
^{19}F NMR (376 MHz, $CDCl_3$) δ -40.21 (s, 3F).
^{13}C NMR (101 MHz, $CDCl_3$) δ 140.5, 130.4 (q, *J* = 307.6 Hz), 128.5, 128.4, 126.3, 84.7, 79.1, 34.8, 30.7 (q, *J* = 2.6 Hz), 23.1, 20.9.
HRMS (APCI) m/z calcd. for $C_{13}H_{14}F_3S$ $[M+H]^+$ 259.0763, found 259.0760.

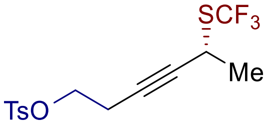

4l

(*R*)-5-((Trifluoromethyl)thio)hex-3-yn-1-yl 4-methylbenzenesulfonate:

Prepared according to **General Procedure A** (35 °C instead of r.t.). The reaction mixture was purified by column chromatography on silica gel (eluent: 2:1 hexanes/CH_2Cl_2, R_f = 0.30) to yield the product **4l** as a colorless oil (36.0 mg, 51% yield, 92% ee).
[α]$_D^{25}$ = +255.0 (c 0.18, $CHCl_3$)
HPLC analysis: Chiralcel OD-3 (hexanes/*i*-PrOH = 95/5, flow rate 1.0 mL/min, λ = 254 nm), t_R (major) = 6.79 min, t_R (minor) = 7.28 min.
^{1}H NMR (400 MHz, $CDCl_3$) δ 7.80 (d, *J* = 7.9 Hz, 2H), 7.36 (d, *J* = 7.9 Hz, 2H), 4.07 (t, *J* = 7.0 Hz, 2H), 4.04 – 3.97 (m, 1H), 2.58 (td, *J* = 7.0, 2.2 Hz, 2H), 2.45 (s, 3H), 1.56 (dd, *J* = 7.0, 0.8 Hz, 3H).
^{19}F NMR (376 MHz, $CDCl_3$) δ -40.27 (s, 3F).
^{13}C NMR (101 MHz, $CDCl_3$) δ 145.0, 132.9, 130.2 (q, *J* = 307.6 Hz), 129.9, 127.9, 80.8, 79.2, 67.4, 30.2 (q, *J* = 2.6 Hz), 22.6, 21.7, 19.7.
HRMS (ESI) m/z calcd. for $C_{14}H_{16}O_3F_3S_2$ $[M+H]^+$ 353.0488, found 353.0489.

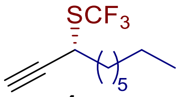

4m

(*R*)-Dec-1-yn-3-yl(trifluoromethyl)sulfane:

Prepared according to **General Procedure D** ($[Ir(cod)Cl]_2$ (2.0 mol %) and (*S*)-**L** (8.0 mol %)) on 0.6 mmol scale. The reaction mixture was purified by column chromatography on silica gel (eluent: hexanes, R_f = 0.65) to yield the product **4m** as a colorless oil (86.2 mg, 60% yield, 81% ee was determined after derivatization to obtain compound **4m'**)
^{1}H NMR (400 MHz, $CDCl_3$) δ 3.97 (td, *J* = 7.0, 2.4 Hz, 1H), 2.44 (d, *J* = 2.4 Hz, 1H), 1.84 (q, *J* = 7.0 Hz, 2H), 1.61 – 1.44 (m, J = 6.6 Hz, 2H), 1.36 – 1.18 (m, 8H), 0.92 – 0.86 (m, 3H).
^{19}F NMR (376 MHz, $CDCl_3$) δ -40.17 (s, 3F).
^{13}C NMR (101 MHz, $CDCl_3$) δ 130.3 (q, *J* = 307.9 Hz), 81.5, 73.3, 35.3, 34.7 (q, *J* = 2.4 Hz), 31.7, 28.9, 28.8, 26.7, 22.6, 14.1.

HRMS (ESI) m/z calcd. for $C_{11}H_{18}F_3S$ $[M+H]^+$ 239.1076, found 239.1079.

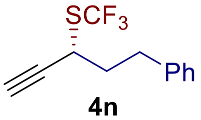

4n

(*R*)-(5-Phenylpent-1-yn-3-yl)(trifluoromethyl)sulfane:

Prepared according to **General Procedure D** ($[Ir(cod)Cl]_2$ (4.0 mol %) and (*S*)-**L** (16 mol %)). The reaction mixture was purified by column chromatography on silica gel (eluent: hexanes, R_f = 0.30) to yield the product **4n** as a colorless oil (25.9 mg, 52% yield, 76% ee). Under identical conditions using (*S*)-Me_2-**L** (17.1 mg, 16 mol %) as the ligand, the product was obtained in 35% yield (16.9 mg) and 84% ee.
$[\alpha]_D^{25}$ = +190.1 (c 0.16, $CHCl_3$)
HPLC analysis: Chiralcel OD-3 (hexanes/*i*-PrOH = 100/0, flow rate 1.0 mL/min, λ = 208 nm), t_R (major) = 7.95 min, t_R (minor) = 10.17 min.
^{1}H NMR (400 MHz, $CDCl_3$) δ 7.38 – 7.30 (m, 2H), 7.27 – 7.21 (m, 3H), 3.97 (td, *J* = 7.0, 2.4 Hz, 1H), 3.21 – 2.78 (m, 2H), 2.54 (d, *J* = 2.4 Hz, 1H), 2.19 (q, *J* = 7.0 Hz, 2H).
^{19}F NMR (376 MHz, $CDCl_3$) δ -39.92 (s, 3F).
^{13}C NMR (101 MHz, $CDCl_3$) δ 139.9, 130.2 (q, *J* = 307.9 Hz), 128.7, 128.5, 126.5, 80.9, 73.9, 36.9, 34.0 (q, *J* = 2.5 Hz), 32.7.
HRMS (APCI) m/z calcd. for $C_{12}H_{12}F_3S$ $[M+H]^+$ 245.0606, found 245.0605.

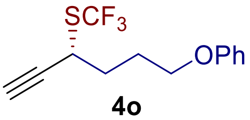

4o

(*R*)-(6-Phenoxyhex-1-yn-3-yl)(trifluoromethyl)sulfane:

Prepared according to **General Procedure D** ($[Ir(cod)Cl]_2$ (3.0 mol %) and (*S*)-**L** (12 mol %)). The reaction mixture was purified by column chromatography on silica gel (eluent: 20:1 hexanes/EtOAc, R_f = 0.45) to yield the product **4o** as a colorless oil (31.1 mg, 57% yield, 79% ee).
$[\alpha]_D^{25}$ = +108.5 (c 0.11, $CHCl_3$)
HPLC analysis: Chiralcel OD-3 (hexanes/*i*-PrOH = 99/1, flow rate 1.0 mL/min, λ = 213 nm), t_R (major) = 6.18 min, t_R (minor) = 7.33 min.
^{1}H NMR (400 MHz, $CDCl_3$) δ 7.34 – 7.26 (m, 2H), 6.96 (t, *J* = 7.3 Hz, 1H), 6.90 (d, *J* = 7.6 Hz, 2H), 4.14 – 4.07 (m, 1H), 4.05 – 3.94 (m, 2H), 2.49 (d, *J* = 2.4 Hz, 1H), 2.22 – 1.82 (m, 4H).
^{19}F NMR (376 MHz, $CDCl_3$) δ -40.04 (s, 3F).
^{13}C NMR (101 MHz, $CDCl_3$) δ 158.8, 130.2 (q, *J* = 307.7 Hz), 129.5, 120.9, 114.5, 80.9, 73.8, 66.7, 34.5 (q, *J* = 2.5 Hz), 32.2, 26.5.
HRMS (APCI) m/z calcd. for $C_{13}H_{14}OF_3S$ $[M+H]^+$ 275.0712, found 275.0701.

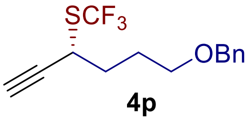

4p

(*R*)-(6-(Benzyloxy)hex-1-yn-3-yl)(trifluoromethyl)sulfane:

Prepared according to **General Procedure D** ($[Ir(cod)Cl]_2$ (3.0 mol %) and (*S*)-**L** (12 mol %)). The reaction mixture was purified by column chromatography on silica gel (eluent: 1:1 hexanes/CH_2Cl_2, R_f = 0.35) to yield the product **4p** as a colorless oil (30.1 mg, 52% yield, 85% ee).
$[\alpha]_D^{25}$ = -118.1 (c 0.16, $CHCl_3$)
HPLC analysis: Chiralcel OD-3 (hexanes/*i*-PrOH = 99/1, flow rate 1.0 mL/min, λ = 209 nm), t_R (minor) = 4.23 min, t_R (major) = 5.34 min.
^{1}H NMR (400 MHz, $CDCl_3$) δ 7.41 – 7.25 (m, 5H), 4.52 (s, 2H), 4.08 – 3.98 (m, 1H), 3.57 – 3.48 (m, 2H), 2.45 (d, *J* = 2.4 Hz, 1H), 2.17 – 1.78 (m, 4H).
^{19}F NMR (376 MHz, $CDCl_3$) δ -40.07 (s, 3F).
^{13}C NMR (101 MHz, $CDCl_3$) δ 138.3, 130.3 (q, *J* = 307.6 Hz), 128.4, 127.7, 127.6, 81.2, 73.6, 72.9, 69.1, 34.5 (q, *J* = 2.4 Hz), 32.3, 26.9.
HRMS (ESI) m/z calcd. for $C_{14}H_{16}OF_3S$ $[M+H]^+$ 289.0869, found 289.0873.

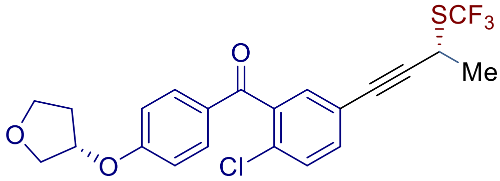

4q

(2-Chloro-5-((*R*)-3-((trifluoromethyl)thio)but-1-yn-1-yl)phenyl)(4-(((*S*)-tetrahydrofuran-3-yl)oxy)phenyl)methanone:

Prepared according to **General Procedure A**. The reaction mixture was purified by column chromatography on silica gel (eluent: 5:1 hexanes/EtOAc, R_f = 0.20) to yield the product **4q** as a light yellow oil (88.1 mg, 97% yield, 94% de)
$[\alpha]_D^{25}$ = +142.1 (c 0.92, $CHCl_3$)
HPLC analysis: Chiralcel OD-3 (hexanes/*i*-PrOH = 98/2, flow rate 1.0 mL/min, λ = 265 nm), t_R (major) = 17.11 min, t_R (minor) = 19.04 min.
^{1}H NMR (400 MHz, $CDCl_3$) δ 7.76 (d, *J* = 8.9 Hz, 2H), 7.48 – 7.43 (m, 1H), 7.41 – 7.35 (m, 2H), 6.90 (d, *J* = 8.9 Hz, 2H), 5.07 – 4.89 (m, 1H), 4.31 (q, *J* = 7.1 Hz, 1H), 4.06 – 3.95 (m, 3H), 3.95 – 3.84 (m, 1H), 2.32 – 2.10 (m, 2H), 1.69 (d, *J* = 7.0 Hz, 3H).
^{19}F NMR (376 MHz, $CDCl_3$) δ -40.11 (s, 3F).
^{13}C NMR (101 MHz, $CDCl_3$) δ 192.6, 162.2, 139.2, 133.7, 132.6, 131.8, 131.4, 130.2 (q, *J* = 307.6 Hz), 130.1, 129.2, 121.3, 115.2, 89.4, 82.6, 77.8, 72.9, 67.2, 33.0, 30.6 (q, *J* = 2.7 Hz), 22.3.
HRMS (ESI) m/z calcd. for $C_{22}H_{19}O_3ClF_3S$ $[M+H]^+$ 455.0690, found 455.0672.

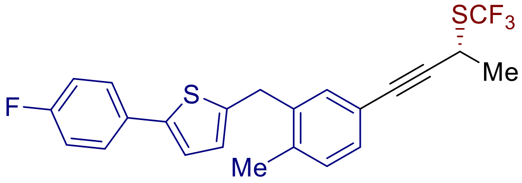

4r

(*R*)-2-(4-Fluorophenyl)-5-(2-methyl-5-(3-((trifluoromethyl)thio)but-1-yn-1-yl)benzyl)thiophene:

Prepared according to **General Procedure A**. The reaction mixture was purified by column chromatography on silica gel (eluent: 20:1 hexanes/EtOAc, R_f = 0.60) to yield the product **4r** as a light yellow oil (88.1 mg, 97% yield, 94% ee).
[α]$_D$^{25} = +173.4 (c 0.45, $CHCl_3$)
HPLC analysis: Chiralcel OD-3 (hexanes/*i*-PrOH = 99/1, flow rate 1.0 mL/min, λ = 252 nm), t_R (major) = 6.06 min, t_R (minor) = 6.35 min.
^{1}H NMR (400 MHz, $CDCl_3$) δ 7.56 – 7.44 (m, 2H), 7.32 (s, 1H), 7.29 – 7.26 (m, 1H), 7.15 (d, *J* = 7.8 Hz, 1H), 7.09 – 6.99 (m, 3H), 6.68 (d, *J* = 3.4 Hz, 1H), 4.36 (q, *J* = 7.0 Hz, 1H), 4.11 (s, 2H), 2.34 (s, 3H), 1.74 (d, *J* = 7.0 Hz, 3H).
^{19}F NMR (376 MHz, $CDCl_3$) δ -40.09 (s, 3F), -115.06 (m, 1F).
^{13}C NMR (101 MHz, $CDCl_3$) δ 162.1 (d, *J* = 246.8 Hz), 142.7, 141.7, 138.3, 137.4, 132.7, 130.8 (d, *J* = 3.4 Hz), 130.6, 130.4 (q, *J* = 307.9 Hz), 130.4, 127.2 (d, *J* = 8.0 Hz), 126.1, 122.7, 120.1, 115.7 (d, *J* = 21.8 Hz), 86.8, 84.6, 33.9, 31.0 (q, *J* = 2.4 Hz), 22.7, 19.5.
HRMS (ESI) m/z calcd. for $C_{23}H_{19}F_4S_2$ $[M+H]^+$ 435.0859, found 435.0850.

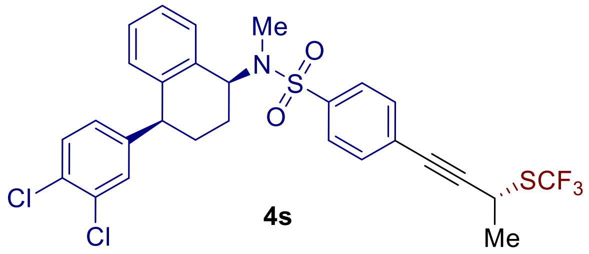

4s

***N*-((1*S*,4*S*)-4-(3,4-Dichlorophenyl)-1,2,3,4-tetrahydronaphthalen-1-yl)-*N*-methyl-4-((*R*)-3-((trifluoromethyl)thio)but-1-yn-1-yl)benzenesulfonamide:**

Prepared according to **General Procedure A**. The reaction mixture was purified by column chromatography on silica gel (eluent: 3:7 hexanes/CH_2Cl_2, R_f = 0.55) to yield the product **4s** as a light yellow oil (117.2 mg, 70% yield, 98% de).
[α]$_D$^{25} = +217.5 (c 0.33, $CHCl_3$)
HPLC analysis: Chiralcel AD-3 (hexanes/*i*-PrOH = 98/2, flow rate 0.8 mL/min, λ = 266 nm), t_R (major) = 21.90 min, t_R (minor) = 23.02 min.
^{1}H NMR (400 MHz, $CDCl_3$) δ 7.76 (d, *J* = 8.4 Hz, 2H), 7.48 (d, *J* = 8.4 Hz, 2H), 7.30 (d, *J* = 7.8 Hz, 1H), 7.25 – 7.15 (m, 2H), 7.12 (t, *J* = 7.0 Hz, 1H), 6.93 (d, *J* = 2.1 Hz, 1H), 6.85 (d, *J* = 7.6 Hz, 1H), 6.67 (dd, *J* = 8.3, 2.1 Hz, 1H), 5.16 (dd, *J* = 10.8, 6.1 Hz, 1H), 4.27 (q, *J* = 7.1 Hz, 1H), 4.11 – 3.94 (m, 1H), 2.58 (s, 3H), 2.23 – 2.03 (m, 1H), 1.89 – 1.78 (m, 1H), 1.65 (d, *J* = 7.1 Hz, 3H), 1.56 – 1.44 (m, 1H), 1.40 – 1.27 (m, 1H).
^{19}F NMR (376 MHz, $CDCl_3$) δ -40.08 (s, 3F).
^{13}C NMR (101 MHz, $CDCl_3$) δ 146.6, 140.1, 138.3, 135.1, 132.4, 132.4, 130.7, 130.5, 130.3, 130.2 (q, J = 307.9 Hz), 130.1, 127.9, 127.8, 126.9, 126.8, 91.0, 82.9, 56.9, 42.8, 30.5 (q, *J* = 2.7 Hz), 29.9, 29.6, 22.1, 21.2.

HRMS (ESI) m/z calcd. for $C_{28}H_{25}O_2NCl_2F_3S_2$ $[M+H]^+$ 598.0650, found 598.0646.

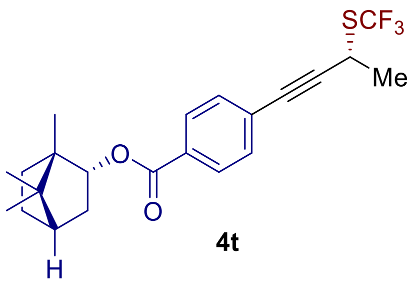

4t

(1*S*,2*R*,4*R*)-1,7,7-Trimethylbicyclo[2.2.1]heptan-2-yl 4-((*R*)-3-((trifluoromethyl)thio)but-1-yn-1-yl)benzoate:

Prepared according to **General Procedure A**. The reaction mixture was purified by column chromatography on silica gel (eluent: 2:1 hexanes/CH_2Cl_2, R_f = 0.35) to yield the product **4t** as a colorless oil (72.8 mg, 89% yield, 93% de).
$[\alpha]_D^{25}$ = -140.7 (c 0.21, $CHCl_3$)
HPLC analysis: Chiralcel AD-3 (hexanes/*i*-PrOH = 99/1, flow rate 0.6 mL/min, λ = 265 nm), t_R (major) = 7.29 min, t_R (minor) = 7.51 min.
^{1}H NMR (400 MHz, $CDCl_3$) δ 7.99 (d, *J* = 8.4 Hz, 2H), 7.48 (d, *J* = 8.4 Hz, 2H), 5.11 (dt, *J* = 9.9, 2.4 Hz, 1H), 4.35 (q, *J* = 7.1 Hz, 1H), 2.56 – 2.42 (m, 1H), 2.19 – 2.03 (m, 1H), 1.86 – 1.77 (m, 1H), 1.76 – 1.70 (m, 4H), 1.47 – 1.36 (m, 1H), 1.35 – 1.22 (m, 1H), 1.11 (dd, *J* = 13.8, 3.5 Hz, 1H), 0.97 (s, 3H), 0.92 (s, 3H), 0.91 (s, 3H).
^{19}F NMR (376 MHz, $CDCl_3$) δ -40.11 (s, 3F).
^{13}C NMR (101 MHz, $CDCl_3$) δ 166.2, 131.6, 130.7, 130.3 (q, *J* = 307.8 Hz), 129.4, 126.8, 90.2, 83.8, 80.9, 49.1, 47.9, 44.9, 36.9, 30.7 (q, *J* = 2.6 Hz), 28.1, 27.4, 22.3, 19.7, 18.9, 13.6.
HRMS (ESI) m/z calcd. for $C_{22}H_{26}O_2F_3S$ $[M+H]^+$ 411.1600 found 411.1602.

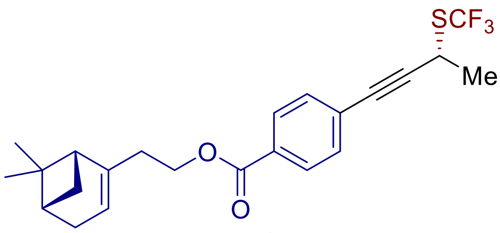

4u

2-((1*R*,5*S*)-6,6-Dimethylbicyclo[3.1.1]hept-2-en-2-yl)ethyl 4-((*R*)-3-((trifluoromethyl)thio)but-1-yn-1-yl)benzoate:

Prepared according to **General Procedure A**. The reaction mixture was purified by column chromatography on silica gel (eluent: 20:1 hexanes/EtOAc, R_f = 0.40) to yield the product **4u** as a colorless oil (70.2 mg, 83% yield, 97% de).
$[\alpha]_D^{25}$ = -53.3 (c 0.16, $CHCl_3$)
HPLC analysis: Chiralcel AD-3 (hexanes/*i*-PrOH = 99/1, flow rate 0.6 mL/min, λ = 265 nm), t_R (major) = 9.60 min, t_R (minor) = 10.57 min.

^{1}**H NMR** (400 MHz, $CDCl_3$) δ 7.97 (d, J = 8.2 Hz, 2H), 7.47 (d, J = 8.2 Hz, 2H), 5.45 – 5.23 (m, 1H), 4.45 – 4.07 (m, 3H), 2.48 – 2.34 (m, 3H), 2.31 – 2.16 (m, 2H), 2.14 – 2.02 (m, 2H), 1.73 (d, J = 7.0 Hz, 3H), 1.27 (s, 3H), 1.16 (d, J = 8.5 Hz, 1H), 0.82 (s, 3H).
^{19}**F NMR** (376 MHz, $CDCl_3$) δ -40.11 (s, 3F).
^{13}**C NMR** (101 MHz, $CDCl_3$) δ 165.9, 144.2, 131.6, 130.3, 130.2 (q, J = 307.9 Hz), 129.4, 126.8, 119.0, 90.2, 83.8, 63.5, 45.8, 40.7, 38.0, 36.0, 31.7, 31.4, 30.7 (q, J = 2.7 Hz), 26.3, 22.3, 21.2.
HRMS (ESI) m/z calcd. for $C_{23}H_{26}O_2F_3S$ $[M+H]^+$ 423.1600, found 423.1606.

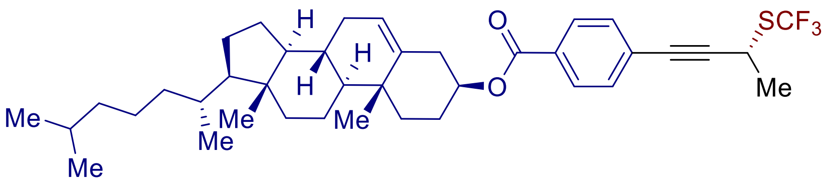

4v

(3*S*,8*S*,9*S*,10*R*,13*R*,14*S*,17*R*)-10,13-Dimethyl-17-((*R*)-6-methylheptan-2-yl)-2,3,4,7,8,9,10,11,12,13,14,15,16,17-tetradecahydro-1*H*-cyclopenta[a]phenanthren-3-yl 4-((*R*)-3-((trifluoromethyl)thio)but-1-yn-1-yl)benzoate:

Prepared according to **General Procedure A** (0.4 mL DCE instead of 0.2 mL DCE). The reaction mixture was purified by column chromatography on silica gel (eluent: 2:1 hexanes/CH_2Cl_2, R_f = 0.25) to yield the product **4v** as a white solid (122.4 mg, 95% yield, 97% de).
m.p. 180.0°C – 182.0 °C
$[\alpha]_D^{25}$ = +18.1 (c 0.16, $CHCl_3$)
HPLC analysis: Chiralcel IB (hexanes/*i*-PrOH = 100/0, flow rate 0.6 mL/min, λ = 265 nm), t_R (major) = 6.45 min, t_R (minor) = 6.63 min.
^{1}**H NMR** (400 MHz, $CDCl_3$) δ 7.98 (d, J = 8.1 Hz, 2H), 7.47 (d, J = 8.1 Hz, 2H), 5.42 (d, J = 5.0 Hz, 1H), 4.99 – 4.76 (m, 1H), 4.35 (q, J = 7.1 Hz, 1H), 2.46 (d, J = 8.3 Hz, 2H), 2.11 – 1.67 (m, 9H), 1.63 – 0.98 (m, 23H), 0.92 (d, J = 6.4 Hz, 3H), 0.88 (s, 3H), 0.86 (s, 3H), 0.69 (s, 3H).
^{19}**F NMR** (376 MHz, $CDCl_3$) δ -40.11 (s, 3F).
^{13}**C NMR** (101 MHz, $CDCl_3$) δ 165.3, 139.6, 131.6, 130.7, 130.3 (q, J = 307.8 Hz), 129.4, 126.7, 122.9, 90.1, 83.8, 74.9, 56.7, 56.2, 50.1, 42.3, 39.8, 39.5, 38.2, 37.0, 36.7, 36.2, 35.8, 32.0, 31.9, 30.7 (q, J = 2.6 Hz), 28.3, 28.0, 27.9, 24.3, 23.9, 22.8, 22.6, 22.3, 21.1, 19.4, 18.7, 11.9.
HRMS (ESI) m/z calcd. for $C_{39}H_{52}O_2F_3S$ $[M+H]^+$ 641.3635, found 641.3630.

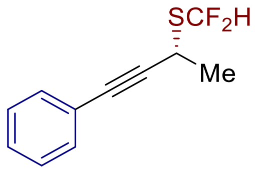

6a

(*R*)-(Difluoromethyl)(4-phenylbut-3-yn-2-yl)sulfane:

Prepared according to **General Procedure B**. The reaction mixture was purified by column

chromatography on silica gel (eluent: hexanes, R_f = 0.35) to yield the product **6a** as a colorless oil (12.8 mg, 60% yield, 95% ee).
[α]$_D$^{25} = +250.9 (c 0.09, $CHCl_3$)
HPLC analysis: Chiralcel OD-3 (hexanes/*i*-PrOH = 99/1, flow rate 1.0 mL/min, λ = 241 nm), t_R (major) = 4.77 min, t_R (minor) = 5.68 min.
^{1}H NMR (400 MHz, $CDCl_3$) δ 7.45 – 7.39 (m, 2H), 7.36 – 7.30 (m, 3H), 7.07 (dd, *J* = 58.2, 55.9 Hz, 1H), 4.22 (q, *J* = 7.1 Hz, 1H), 1.69 (d, *J* = 7.1 Hz, 3H).
^{19}F NMR (376 MHz, $CDCl_3$) δ -92.85 (dd, *J* = 251.7, 58.2 Hz, 1F), -95.05 (dd, *J* = 251.7, 55.9 Hz, 1F).
^{13}C NMR (101 MHz, $CDCl_3$) δ 131.7, 128.6, 128.3, 122.4, 121.1 (dd, *J* = 273.9, 270.5 Hz), 87.9, 84.3, 28.7 (t, *J* = 3.3 Hz), 22.7.
HRMS (ESI) m/z calcd. for $C_{11}H_{11}F_2S$ $[M+H]^+$ 213.0544, found 213.0548.

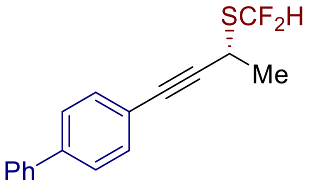

6b

(*R*)-(4-([1,1'-Biphenyl]-4-yl)but-3-yn-2-yl)(difluoromethyl)sulfane:

Prepared according to **General Procedure B**. The reaction mixture was purified by column chromatography on silica gel (eluent: 10:1 hexanes/CH_2Cl_2, R_f = 0.30) to yield the product **6b** as a white foam (18.4 mg, 64% yield, 97% ee).
[α]$_D$^{25} = +281.9 (c 0.14, $CHCl_3$)
HPLC analysis: Chiralcel OD-3 (hexanes/*i*-PrOH = 99/1, flow rate 1.0 mL/min, λ = 275 nm), t_R (minor) = 5.92 min, t_R (major) = 7.45 min.
^{1}H NMR (400 MHz, $CDCl_3$) δ 7.63 – 7.53 (m, 4H), 7.50 (d, *J* = 8.3 Hz, 2H), 7.45 (t, *J* = 7.5 Hz, 2H), 7.40 – 7.33 (m, 1H), 7.09 (dd, *J* = 58.2, 56.0 Hz, 1H), 4.25 (q, *J* = 7.1 Hz, 1H), 1.71 (d, *J* = 7.0 Hz, 3H).
^{19}F NMR (376 MHz, $CDCl_3$) δ -92.80 (dd, *J* = 251.3, 58.0 Hz, 1F), -94.98 (dd, *J* = 251.3, 55.9 Hz, 1F).
^{13}C NMR (101 MHz, $CDCl_3$) δ 141.4, 140.3, 132.2, 128.9, 127.7, 127.04, 127.02, 121.2, 121.1 (dd, J = 274.0, 270.7 Hz), 88.6, 84.2, 28.8 (t, J = 3.1 Hz), 22.7.
HRMS (ESI) m/z calcd. for $C_{17}H_{15}F_2S$ $[M+H]^+$ 289.0857, found 289.0864.

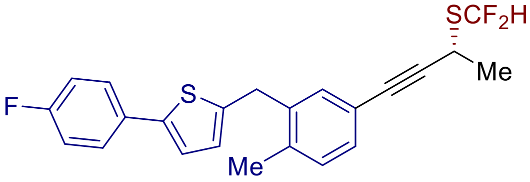

6c

(*R*)-2-(5-(3-((Difluoromethyl)thio)but-1-yn-1-yl)-2-methylbenzyl)-5-(4-fluorophenyl)thiophene:

Prepared according to **General Procedure B**. The reaction mixture was purified by column

chromatography on silica gel (eluent: 5:1 hexanes/CH_2Cl_2, R_f = 0.30) to yield the product **6c** as a colorless oil (31.7 mg, 76% yield, 97% ee).

[α]$_D$^{25} = +201.2 (c 0.31, $CHCl_3$)

HPLC analysis: Chiralcel OD-3 (hexanes/*i*-PrOH = 99/1, flow rate 1.0 mL/min, λ = 252 nm), t_R (major) = 7.73 min, t_R (minor) = 9.28 min.

^{1}H NMR (400 MHz, $CDCl_3$) δ 7.54 – 7.44 (m, 2H), 7.32 (s, 1H), 7.29 – 7.25 (m, 1H), 7.24 – 6.91 (m, 5H), 6.68 (d, *J* = 3.5 Hz, 1H), 4.23 (q, *J* = 7.1 Hz, 1H), 4.11 (s, 2H), 2.34 (s, 3H), 1.69 (d, *J* = 7.0 Hz, 3H).

^{19}F NMR (376 MHz, $CDCl_3$) δ -92.81 (dd, *J* = 251.7, 58.2 Hz, 1F), -95.01 (dd, *J* = 251.7, 55.9 Hz, 1F), -110.41 – -116.01 (m, 1F).

^{13}C NMR (101 MHz, $CDCl_3$) δ 162.2 (d, *J* = 246.7 Hz), 142.7, 141.7, 138.4, 137.4, 132.7, 130.8 (d, *J* = 3.4 Hz), 130.6, 130.4, 127.2 (d, *J* = 7.9 Hz), 126.1, 122.7 (d, *J* = 1.3 Hz), 121.1 (dd, *J* = 273.9, 270.4 Hz), 120.1, 115.7 (d, *J* = 21.7 Hz), 87.5, 84.4, 33.9, 28.8 (t, *J* = 3.2 Hz), 22.8, 19.5.

HRMS (ESI) m/z calcd. for $C_{23}H_{20}F_3S_2$ $[M+H]^+$ 417.0953, found 417.0962.

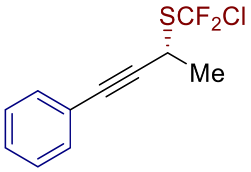

6d

(*R*)-(Chlorodifluoromethyl)(4-phenylbut-3-yn-2-yl)sulfane:

Prepared according to **General Procedure C**. The reaction mixture was purified by column chromatography on silica gel (eluent: hexanes, R_f = 0.40) to yield the product **6d** as a colorless oil (29.1 mg, 59% yield, 91% ee).

[α]$_D$^{25} = +197.1 (c 0.16, $CHCl_3$)

HPLC analysis: Chiralcel OD-3 (hexanes/*i*-PrOH = 100/0, flow rate 1.0 mL/min, λ = 243 nm), t_R (major) = 7.15 min, t_R (minor) = 7.72 min.

^{1}H NMR (400 MHz, $CDCl_3$) δ 7.45 – 7.38 (m, 2H), 7.35 – 7.28 (m, 3H), 4.40 (q, *J* = 7.1 Hz, 1H), 1.72 (d, *J* = 7.1 Hz, 3H).

^{19}F NMR (376 MHz, $CDCl_3$) δ -25.44 – -26.56 (m, 2F).

^{13}C NMR (101 MHz, $CDCl_3$) δ 131.7, 130.9 (t, *J* = 324.0 Hz), 128.6, 128.3, 122.4, 87.4, 84.6, 32.6 (t, *J* = 2.0 Hz), 22.4.

HRMS (APCI) m/z calcd. for $C_{11}H_{11}ClF_2S$ $[M+H]^+$ 247.0154, found 247.0151.

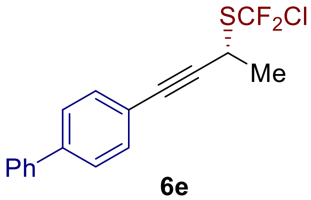

6e

(*R*)-(4-([1,1'-Biphenyl]-4-yl)but-3-yn-2-yl)(chlorodifluoromethyl)sulfane:

Prepared according to **General Procedure C**. The reaction mixture was purified by column

chromatography on silica gel (twice, eluent: 20:1 hexanes/Et_2O, R_f = 0.70, then eluent: 10:1 hexanes/CH_2Cl_2, R_f = 0.40) to yield the product **6e** as a white foam (36.4 mg, 56% yield, 89% ee).
$[\alpha]_D^{25}$ = +591.6 (c 0.07, $CHCl_3$)
HPLC analysis: Chiralcel OD-3 (hexanes/*i*-PrOH = 100/0, flow rate 1.0 mL/min, λ = 278 nm), t_R (major) = 18.92 min, t_R (minor) = 23.79 min.
^{1}H NMR (400 MHz, $CDCl_3$) δ 7.62 – 7.53 (m, 4H), 7.52 – 7.42 (m, 4H), 7.36 (t, *J* = 7.4 Hz, 1H), 4.43 (q, *J* = 7.1 Hz, 1H), 1.74 (d, *J* = 7.1 Hz, 3H).
^{19}F NMR (376 MHz, $CDCl_3$) δ -25.34 – -26.82 (m, 2F).
^{13}C NMR (101 MHz, $CDCl_3$) δ 141.4, 140.3, 132.1, 130.9 (t, *J* = 324.1 Hz), 128.9, 127.7, 127.1, 126.9, 121.2, 88.0, 84.6, 32.7 (t, *J* = 1.9 Hz), 22.4.
HRMS (ESI) m/z calcd. for $C_{17}H_{14}ClF_2S$ $[M+H]^+$ 323.0467, found 323.0477.

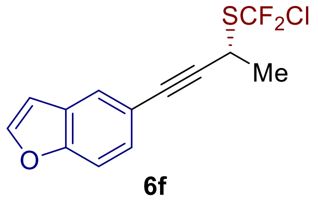

6f

(*R*)-5-(3-((Chlorodifluoromethyl)thio)but-1-yn-1-yl)benzofuran:

Prepared according to **General Procedure C**. The reaction mixture was purified by column chromatography on silica gel (twice, eluent: 10:1 hexanes/Et_2O, R_f = 0.55, then eluent: 3:1 hexanes/CH_2Cl_2, R_f = 0.60) to yield the product **6f** as a colorless oil (30.5 mg, 53% yield, 88% ee).
$[\alpha]_D^{25}$ = +518.7 (c 0.10, $CHCl_3$)
HPLC analysis: Chiralcel OJ-3 (hexanes/*i*-PrOH = 99/1, flow rate 1.0 mL/min, λ = 229 nm), t_R (major) = 9.17 min, t_R (minor) = 10.96 min.
^{1}H NMR (400 MHz, $CDCl_3$) δ 7.69 (s, 1H), 7.64 (d, *J* = 2.2 Hz, 1H), 7.44 (d, *J* = 8.5 Hz, 1H), 7.36 (dd, *J* = 8.5, 1.6 Hz, 1H), 6.74 (d, *J* = 1.3 Hz, 1H), 4.42 (q, *J* = 7.0 Hz, 1H), 1.74 (d, *J* = 7.0 Hz, 3H).
^{19}F NMR (376 MHz, $CDCl_3$) δ -25.43 – -26.59 (m, 2F).
^{13}C NMR (101 MHz, $CDCl_3$) δ 154.7, 145.9, 130.9 (t, *J* = 324.1 Hz), 128.1, 127.5, 124.9, 116.9, 111.5, 106.5, 85.9, 85.0, 32.8 (d, *J* = 2.0 Hz), 22.5.
HRMS (APCI) m/z calcd. for $C_{13}H_{10}OClF_2S$ $[M+H]^+$ 287.0104, found 287.0096.

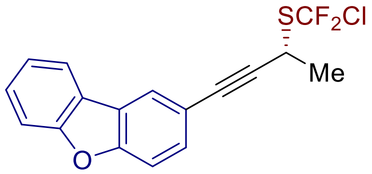

6g

(*R*)-2-(3-((Chlorodifluoromethyl)thio)but-1-yn-1-yl)dibenzo[*b*,*d*]furan:

Prepared according to **General Procedure C**. The reaction mixture was purified by column chromatography on silica gel (twice, eluent: 30:1 hexanes/EtOAc, R_f = 0.65, then eluent: 5:1 hexanes/CH_2Cl_2, R_f = 0.50) to yield the product **6g** as a colorless oil (39.2 mg, 58% yield, 87% ee).
$[\alpha]_D^{25}$ = -486.7 (c 0.03, $CHCl_3$)

HPLC analysis: Chiralcel OD-3 (hexanes/*i*-PrOH = 99/1, flow rate 1.0 mL/min, λ = 241 nm), t_R (minor) = 6.57 min, t_R (major) = 8.22 min.
^{1}H NMR (400 MHz, $CDCl_3$) δ 8.03 (s, 1H), 7.93 (d, *J* = 7.7 Hz, 1H), 7.64 – 7.45 (m, 4H), 7.36 (t, *J* = 7.5 Hz, 1H), 4.45 (q, *J* = 7.1 Hz, 1H), 1.76 (d, *J* = 7.1 Hz, 3H).
^{19}F NMR (376 MHz, $CDCl_3$) δ -25.39 – -26.41 (m, 2F).
^{13}C NMR (101 MHz, $CDCl_3$) δ 156.6, 155.9, 130.9 (t, *J* = 324.1 Hz), 130.8, 124.5, 124.3, 123.5, 123.1, 120.8, 116.8, 111.8, 111.7, 86.5, 84.8, 32.8 (t, *J* = 2.1 Hz), 22.5.
HRMS (ESI) m/z calcd. for $C_{17}H_{12}OClF_2S$ $[M+H]^+$ 337.0260, found 337.0268.

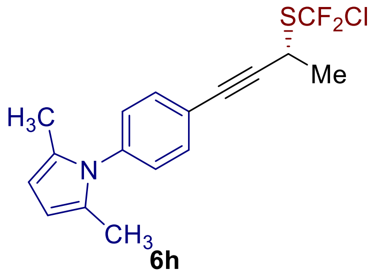

6h

(*R*)-1-(4-(3-((Chlorodifluoromethyl)thio)but-1-yn-1-yl)phenyl)-2,5-dimethyl-1*H*-pyrrole:

Prepared according to **General Procedure C**. the reaction mixture was purified by column chromatography on silica gel (twice, eluent: 20:1 hexanes/EtOAc, R_f = 0.40, then eluent: 2:1 hexanes/CH_2Cl_2, R_f = 0.60) to yield the product **6h** as a pale yellow oil (41.3 mg, 61% yield, 89% ee).
$[\alpha]_D^{25}$ = +267.0 (c 0.26, $CHCl_3$)
HPLC analysis: Chiralcel OD-3 (hexanes/*i*-PrOH = 99/1, flow rate 0.5 mL/min, λ = 267 nm), t_R (major) = 11.68 min, t_R (minor) = 11.84 min.
^{1}H NMR (400 MHz, $CDCl_3$) δ 7.44 (d, *J* = 8.4 Hz, 2H), 7.08 (d, *J* = 8.4 Hz, 2H), 5.83 (s, 2H), 4.34 (q, *J* = 7.1 Hz, 1H), 1.95 (s, 6H), 1.66 (d, *J* = 7.1 Hz, 3H).
^{19}F NMR (376 MHz, $CDCl_3$) δ -25.63 – -26.40 (m, 2F).
^{13}C NMR (101 MHz, $CDCl_3$) δ 139.2, 132.5, 130.8 (t, *J* = 324.2 Hz), 128.7, 128.2, 121.8, 106.2, 88.5, 83.8, 32.5 (t, *J* = 1.9 Hz), 22.3, 12.9.
HRMS (ESI) m/z calcd. for $C_{17}H_{17}NClF_2S$ $[M+H]^+$ 340.0733, found 340.0741.

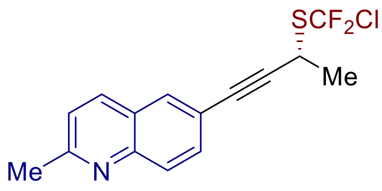

6i

(*R*)-6-(3-((Chlorodifluoromethyl)thio)but-1-yn-1-yl)-2-methylquinoline:

Prepared according to **General Procedure C**. The reaction mixture was purified by column chromatography on silica gel (twice, eluent: 2:1 hexanes/EtOAc, R_f = 0.50, then eluent: 20:1 CH_2Cl_2/MeOH, R_f = 0.55) to yield the product **6i** as a pale yellow oil (25.6 mg, 41% yield, 89% ee).
$[\alpha]_D^{25}$ = +375.8 (c 0.04, $CHCl_3$)
HPLC analysis: Chiralcel AD-3 (hexanes/*i*-PrOH = 99/1, flow rate 0.8 mL/min, λ = 246 nm), t_R

(major) = 12.63 min, t_R (minor) = 13.48 min.
^{1}H NMR (400 MHz, $CDCl_3$) δ 7.96 (dd, *J* = 16.5, 8.6 Hz, 2H), 7.86 (s, 1H), 7.65 (dd, *J* = 8.7, 1.9 Hz, 1H), 7.29 (d, *J* = 8.5 Hz, 1H), 4.43 (q, *J* = 7.1 Hz, 1H), 2.74 (s, 3H), 1.75 (d, *J* = 7.1 Hz, 3H).
^{19}F NMR (376 MHz, $CDCl_3$)δ -25.49 – -26.63 (m, 2F).
^{13}C NMR (101 MHz, $CDCl_3$) δ 160.0, 147.4, 135.9, 132.1, 131.2, 130.9 (t, *J* = 324.1 Hz), 128.8, 126.1, 122.7, 119.7, 88.3, 84.4, 32.6 (t, *J* = 2.1 Hz), 25.4, 22.3.
HRMS (ESI) m/z calcd. for $C_{15}H_{13}NClF_2S$ $[M+H]^+$ 312.0420, found 312.0427.

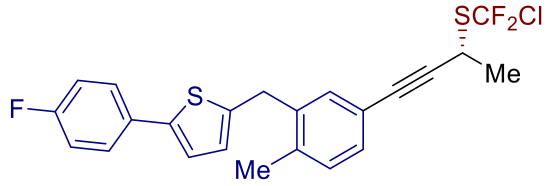

6k

(*R*)-2-(5-(3-((Chlorodifluoromethyl)thio)but-1-yn-1-yl)-2-methylbenzyl)-5-(4-fluorophenyl)thiophene:

Prepared according to **General Procedure C**. The reaction mixture was purified by column chromatography on silica gel (twice, eluent: 5:1 hexanes/CH_2Cl_2, R_f = 0.40, then eluent: 5:1 hexanes/Et_2O, R_f = 0.75) to yield the product **6k** as a colorless oil (44.4 mg, 49% yield, 91% ee).
$[\alpha]_D^{25}$ = +912.8 (c 0.04, $CHCl_3$)
HPLC analysis: Chiralcel OD-3 (hexanes/*i*-PrOH = 99/1, flow rate 0.5 mL/min, λ = 290 nm), t_R (major) = 11.19 min, t_R (minor) = 11.43 min.
^{1}H NMR (400 MHz, $CDCl_3$) δ 7.52 – 7.41 (m, 2H), 7.29 (s, 1H), 7.25 (d, *J* = 6.8 Hz, 1H), 7.13 (d, *J* = 7.8 Hz, 1H), 7.06 – 6.97 (m, 3H), 6.66 (d, *J* = 3.6 Hz, 1H), 4.39 (q, *J* = 7.0 Hz, 1H), 4.09 (s, 2H), 2.32 (s, 3H), 1.71 (d, *J* = 7.0 Hz, 3H).
^{19}F NMR (376 MHz, $CDCl_3$) δ -25.42 – -26.62 (m, 2F), -114.89 – -115.30 (m, 1F).
^{13}C NMR (101 MHz, $CDCl_3$) δ 142.7, 141.7, 138.3, 137.4, 132.7, 130.8 (d, *J* = 3.3 Hz), 130.6, 130.34, 127.2, 127.1, 126.1, 122.7, 120.1, 115.7 (d, *J* = 21.8 Hz), 86.9, 84.7, 33.9, 32.8 (t, *J* = 1.9 Hz), 22.6, 19.5.
HRMS (ESI) m/z calcd. for $C_{23}H_{19}ClF_3S_2$ $[M+H]^+$ 451.0563, found 451.0575.

4. Synthetic applications of products

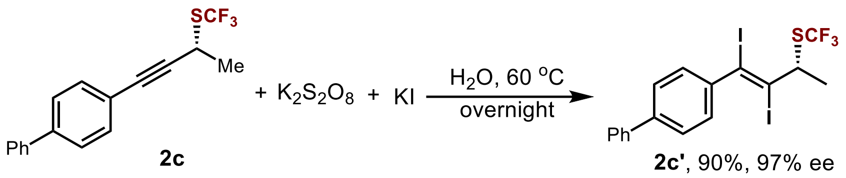

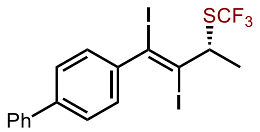

2c'

(*R*,*E*)-(4-([1,1'-Biphenyl]-4-yl)-3,4-diiodobut-3-en-2-yl)(trifluoromethyl)sulfane:

To a 10 mL vial were added $K_2S_2O_8$ (3.0 equiv), KI (2.5 equiv), **2c** (0.1 mmol), and H_2O (0.4 mL). The reaction mixture was warmed to 60 °C and stirred overnight. The mixture was cooled to r.t., and EtOAc (10 mL) and saturated Na_2SO_3 solution (10 mL) were added. The organic layer was separated, and the aqueous phase was extracted with EtOAc (2 × 10 mL). The combined organic layers were dried over anhydrous $MgSO_4$, filtered, and concentrated. The residue was purified by column chromatography (eluent: 20:1 hexanes/CH_2Cl_2, R_f = 0.30) to give the pure product **2c'** as a pale yellow solid (50.3 mg, 90% yield, 97% ee).

m.p. 69.0°C – 70.0 °C

$[\alpha]_D^{25}$ = +120.2 (c 0.33, $CHCl_3$)

HPLC analysis: Chiralcel OD-3 (hexanes/*i*-PrOH = 99/1, flow rate 0.8 mL/min, λ = 228 nm), t_R (major) = 5.83 min, t_R (minor) = 6.54 min.

^{1}H NMR (400 MHz, $CDCl_3$) δ 7.58 – 7.49 (m, 4H), 7.42 – 7.35 (m, 2H), 7.34 – 7.27 (m, 1H), 7.23 – 7.15 (m, 2H), 4.60 (q, J = 6.8 Hz, 1H), 1.48 (d, J = 6.8 Hz, 3H).

^{19}F NMR (376 MHz, $CDCl_3$) δ -39.06 (s, 3F).

^{13}C NMR (101 MHz, $CDCl_3$) δ 146.3, 141.4, 140.2, 130.3 (q, J = 307.8 Hz), 128.9, 128.6, 127.7, 127.2, 127.1, 110.7, 95.9, 56.1 (q, J = 2.1 Hz), 22.0.

HRMS (ESI) m/z calcd. for $C_{17}H_{13}F_3I_2S$ $[M]^+$ 559.8774, found 559.8788.

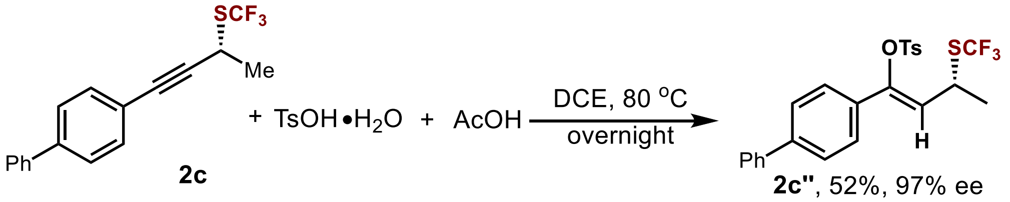

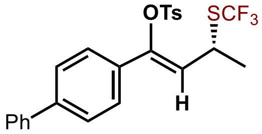

2c''

(*R*,*Z*)-1-([1,1'-Biphenyl]-4-yl)-3-((trifluoromethyl)thio)but-1-en-1-yl 4-methylbenzenesulfonate:
Alkyne **2c** (0.2 mmol) was added to a solution of *p*-toluenesulfonic acid monohydrate (0.2 mmol, 38.0 mg) and acetic acid (0.1 mL) in DCE (0.4 mL). The reaction vessel was then sealed and stirred overnight at 60 °C. After completion, saturated aqueous $NaHCO_3$ (10 mL) was added to quench the

reaction, the organic layer was separated, and the aqueous phase was extracted with CH_2Cl_2 (3 × 10 mL). The combined organic layers were dried over $MgSO_4$ and concentrated *in vacuo*. The residue was purified by column chromatography (eluent: 10:1 hexanes/EtOAc, R_f = 0.45) to give the product **2c"** as a pale yellow oil (49.9 mg, 52% yield, 97% ee).

[α]$_D^{25}$ = -103.9 (c 0.53, $CHCl_3$)

HPLC analysis: Chiralcel AD-3 (hexanes/*i*-PrOH = 98/2, flow rate 0.8 mL/min, λ = 270 nm), t_R (minor) = 15.61 min, t_R (major) = 19.66 min.

^{1}H NMR (400 MHz, $CDCl_3$) δ 7.65 (d, *J* = 8.4 Hz, 2H), 7.58 – 7.54 (m, 2H), 7.52 – 7.43 (m, 4H), 7.42 – 7.35 (m, 1H), 7.33 – 7.29 (m, 2H), 7.21 (d, *J* = 8.1 Hz, 2H), 5.58 (d, *J* = 11.2 Hz, 1H), 4.12 (dq, *J* = 11.2, 6.8 Hz, 1H), 2.39 (s, 3H), 1.48 (d, *J* = 6.8 Hz, 3H).

^{19}F NMR (376 MHz, $CDCl_3$) δ -38.84 (s, 3F).

^{13}C NMR (101 MHz, $CDCl_3$) δ 147.6, 145.1, 142.5, 140.1, 133.1, 130.8, 130.2 (q, *J* = 307.7 Hz), 129.6, 128.9, 128.4, 127.9, 127.1, 126.9, 123.2, 38.6 (q, *J* = 1.9 Hz), 21.9, 21.6.

HRMS (ESI) m/z calcd. for $C_{24}H_{22}O_3F_3S_2$ $[M+H]^+$ 479.0957, found 479.0967.

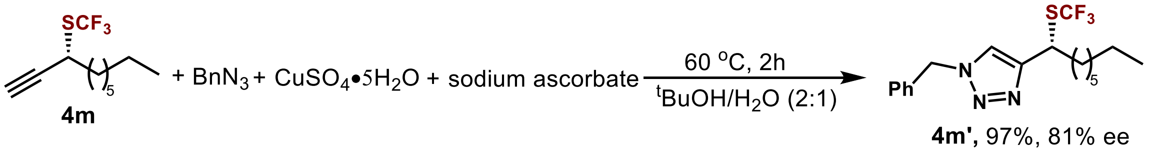

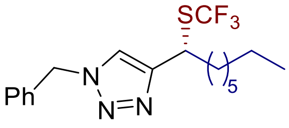

4m'

(*R*)-1-Benzyl-4-(1-((trifluoromethyl)thio)octyl)-1*H*-1,2,3-triazole:

Derivatization of compound **4m** was achieved using a copper-catalyzed alkyne–azide click reaction: A solution of alkyne **4m** (86 mg, 0.36 mmol, 1.0 equiv) (obtained from the iridium-catalyzed reaction and subjected to deprotection) and benzyl azide (96.0 mg, 0.72 mmol, 2.0 equiv) in *t*-BuOH/H_2O (2:1, 9.0 mL) was degassed by means of two freeze-pump-thaw cycles. Sodium L-ascorbate (59.4 mg, 0.30 mmol, 0.8 equiv) and $CuSO_4 \cdot 5H_2O$ (30 mg, 0.12 mmol, 0.33 equiv) were added and the resulting mixture was stirred at 60 °C. After 2 h, H_2O (15 mL) was added, the organic layer was separated, and the aqueous phase was extracted with CH_2Cl_2 (3 × 30 mL). The combined organic layers were dried over $MgSO_4$, and the solvent was removed under reduced pressure. Purification by column chromatography (eluent: 5:1 hexanes/EtOAc, R_f = 0.40) gave triazole **4m'** as a white solid (129.3 mg, 97% yield, 81% ee).

m.p. 45.0 °C – 46.0 °C

[α]$_D^{25}$ = +156.1 (c 0.16, $CHCl_3$)

HPLC analysis: Chiralcel IB (hexanes/*i*-PrOH = 99/1, flow rate 0.45 mL/min, λ = 205 nm), t_R (minor) = 28.99 min, t_R (major) = 29.85 min.

^{1}H NMR (400 MHz, $CDCl_3$) δ 7.42 – 7.33 (m, 4H), 7.25 – 7.20 (m, 2H), 5.53 (s, 2H), 4.48 (t, *J* = 7.4 Hz, 1H), 2.16 – 2.05 (m, 1H), 2.04 – 1.90 (m, 1H), 1.48 – 1.12 (m, 10H), 0.86 (t, *J* = 6.8 Hz, 3H).

^{19}F NMR (376 MHz, $CDCl_3$) δ -39.90 (s, 3F).

^{13}C NMR (101 MHz, $CDCl_3$) δ 148.5, 134.5, 130.6 (q, *J* = 307.9 Hz), 129.2, 128.8, 127.9, 121.5, 54.2, 40.8 (q, *J* = 2.2 Hz), 35.2, 31.7, 29.0, 28.9, 26.9, 22.6, 14.1.

HRMS (ESI) m/z calcd. for $C_{18}H_{25}N_3F_3S$ $[M+H]^+$ 372.1716, found 372.1710.

5. Mechanistic studies

5.1 Kinetic isotope effect experiments

(1) Independent rate KIE results

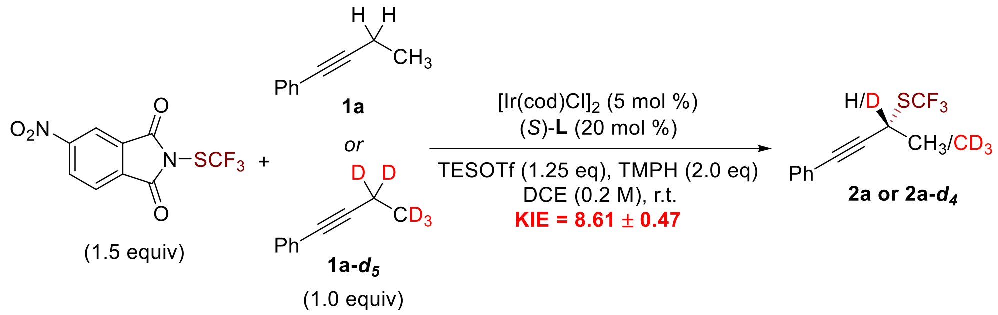

In an argon-filled glove box, an oven-dried reaction tube equipped with magnetic stir bar was charged with $[Ir(cod)Cl]_2$ (3.4 mg, 0.005 mmol, 5 mol %) and phosphoramidite (*S*)-**L** (10.1 mg, 0.02 mmol, 20 mol %), followed by the addition of 1,2-dichloroethane (0.5 mL) via syringe. The resultant solution was stirred at room temperature for 1 hour to give a dark red solution. The solution was transferred into an NMR tube charge with $PhCF_3$ (0.1 mmol, 14.6 mg) as the internal standard. To the solution was added **R6** (44 mg, 0.15 mmol, 1.5 equiv), **1a** (13 mg, 0.10 mmol) or **1a**-d_5 (13.5 mg, 0.10 mmol), distilled TMPH (35 μL, 0.20 mmol, 2.0 equiv) and triethylsilyl trifluoromethanesulfonate (TESOTf, 29 μL, 0.125 mmol, 1.25 equiv).

The NMR tube was then shaken by hand until a color change from dark red to light orange was observed. The reaction was monitored by ^{19}F NMR during the initial period of reaction (conversion < 20%). Observed initial rates were extracted from the slopes of [**2a or 2a**-d_4] vs time. Each reaction was repeated three times.

Table S1. Original data for independent kinetic experiments (% yield, 1st run)

	6 min	10 min	14 min	18 min	22 min	26 min	30 min
**2a**	3	5	7	9	12	14	16
	18 min	36 min	55 min	76 min	97 min	115 min	133 min
**2a-d_4**	1	2	3	4	6	7	8

Chart S1. Initial rates with **2a**.

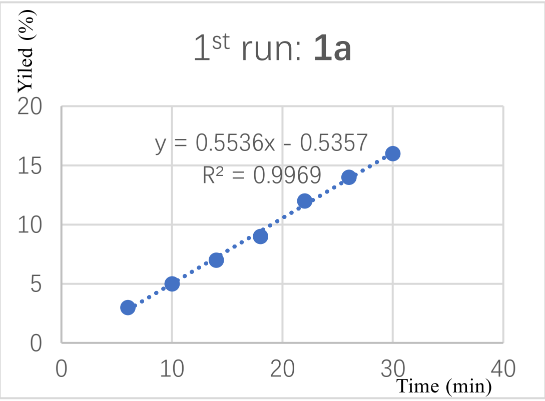

Chart S1-d. Initial rates with **2a-d_4**.

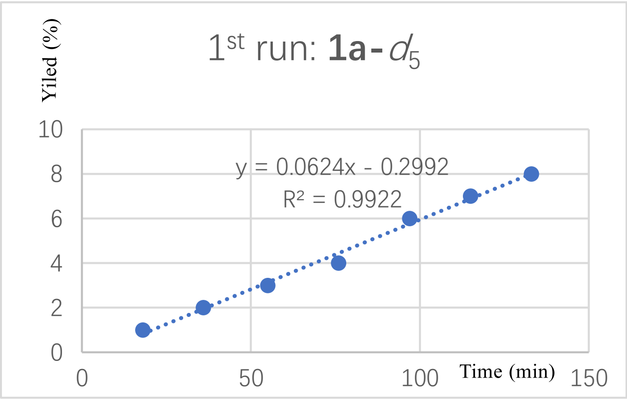

k_H/k_D = 0.5536/0.0624 = 8.87

Table S2. Original data for independent kinetic experiments (% yield, 2nd run)

	0 min	14 min	18 min	22 min	26 min	30 min	34 min
**2a**	0	7	9	11	13	15	17
	39 min	57 min	76 min	97 min	118 min	136 min	154 min
**2a-*d*4**	2	3	5	6	7	8	9

Chart S2. Initial rates with **2a**.

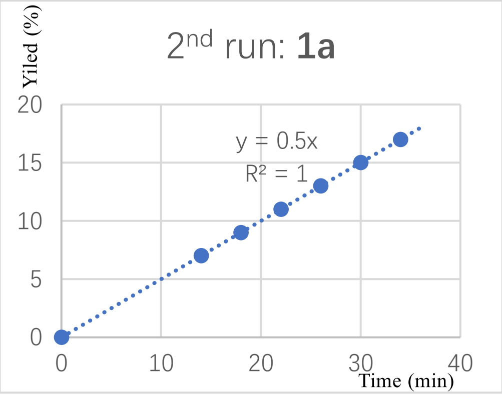

Chart S2-d. Initial rates with **2a-*d*4**.

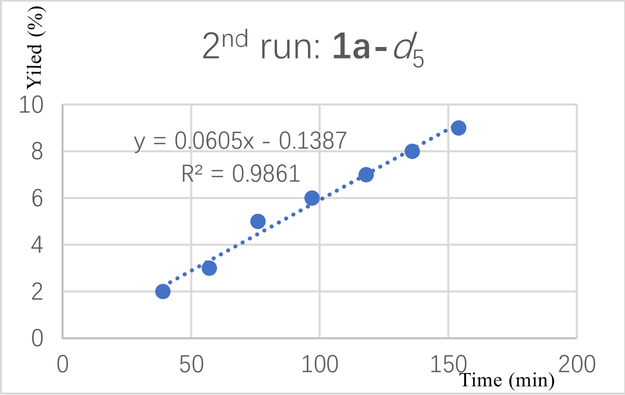

$\boldsymbol{k_H/k_D}$ = 0.5000/0.0605 = 8.26

Table S3. Original data for independent kinetic experiments (% yield, 3rd run)

	8 min	12 min	16 min	20 min	24 min	28 min	32 min
**2a**	3	6	8	10	12	14	16
	15 min	69 min	90 min	107 min	128 min	149 min	167 min
**2a-*d*4**	1	4	5	6	8	9	10

Chart S3. Initial rates with **2a**.

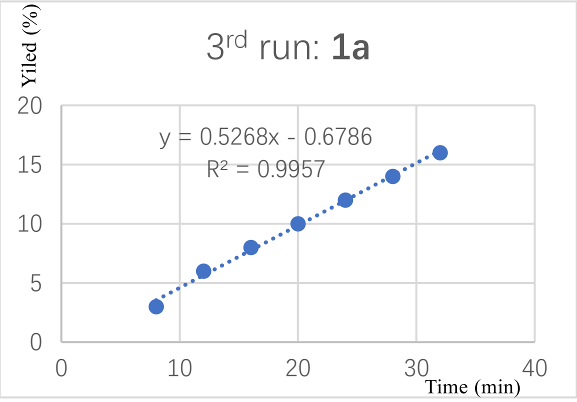

Chart S3-d. Initial rates with **2a-*d*4**.

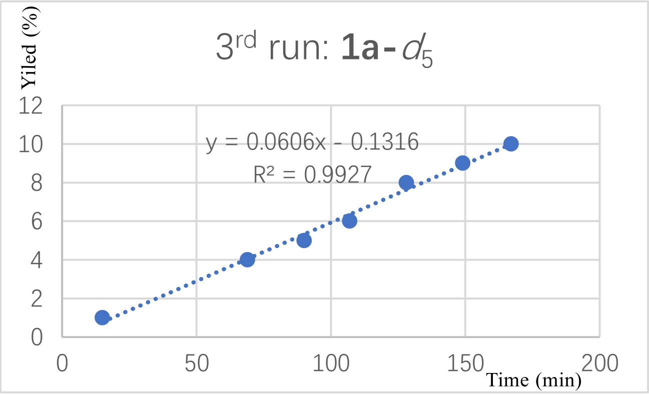

$\boldsymbol{k_H/k_D}$ = 0.5268/0.0606 = 8.69

The uncertainty was calculated using the formula for propagation of errors for KIE = k_H/k_D: ΔKIE = KIE•$((\Delta k_H / k_H)^2 + (\Delta k_D / k_D)^2)^{1/2}$. Average and uncertainty: $\boldsymbol{k_H/k_D}$ **= 8.61 ± 0.47**.

(2) Competitive KIE results

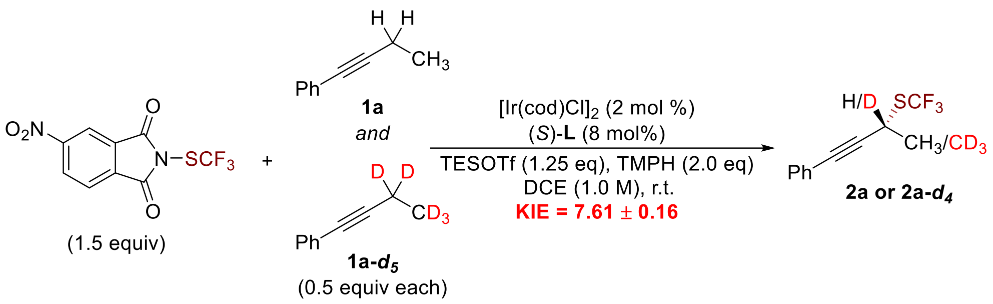

In an argon-filled glove box, an oven-dried reaction tube equipped with magnetic stir bar was charged with $[Ir(cod)Cl]_2$ (2.7 mg, 0.004 mmol, 2.0 mol %) and phosphoramidite (*S*)-**L** (8.1 mg, 0.016 mmol, 8.0 mol %), followed by the addition of 1,2-dichloroethane (0.2 mL) via syringe. The resultant solution was stirred at room temperature for 1 hour to give a dark red solution.

The solution was added **R6** (88 mg, 0.30 mmol, 1.5 equiv), **1a** (13 mg, 0.10 mmol, 0.5 equiv) and **1a**-*d*$_5$ (13.5 mg, 0.10 mmol, 0.5 equiv), distilled TMPH (70 μL, 0.40 mmol, 2.0 equiv) and triethylsilyl trifluoromethanesulfonate (TESOTf, 58 μL, 0.25 mmol, 1.25 equiv). The reaction tube was then capped and the reaction was stirred at room temperature for 40 min. The conversion was determined by crude ^{19}F NMR with $PhCF_3$ (0.1 mmol, 14.6 mg) as the internal standard (conversion < 15%). Each reaction was repeated three times.

Table S4. Original data for competition experiments (% yield, Repeat three times)

	1st run	2nd run	3rd run
**Total Yield (2a and 2a-*d*$_4$)**	14	14	14
**P_H/P_D (area ratio from NMR)**	7.65	7.44	7.75

Average and uncertainty (std. dev.): k_H/k_D = **7.61 ± 0.16**

5.2 Non-linear effect experiments

(1) Mixture of [(*S*)-L]$_2$IrCl and [(*R*)-L]$_2$IrCl complexes

Preparation of the (S)-Ir complex stock solution: In an argon-filled glove box, a vial equipped with a stir bar were charged with [Ir(cod)Cl]$_2$ (6.7 mg, 0.01 mmol) and phosphoramidite (*S*)-**L** (20.3 mg, 0.04 mmol) and followed by the addition of 1,2-dichloroethane (450 µL) via syringe. The resultant solution was stirred at room temperature for one hour to give a dark red solution.

Preparation of the (R)-Ir complex stock solution: In an argon-filled glove box, a vial equipped with a stir bar were charged with [Ir(cod)Cl]$_2$ (2.7 mg, 0.004 mmol) and phosphoramidite (*R*)-**L** (8.1 mg, 0.016 mmol) and followed by the addition of 1,2-dichloroethane (150 µL) via syringe. The resultant solution was stirred at room temperature for one hour to give a dark red solution.

Ir-catalyzed alkyne trifluoromethylthiolation reaction: To an oven-dried reaction tube was added 1-phenyl-1-butyne (**1a**, 13 mg, 0.1 mmol, 1.0 equiv) and **R6** (44 mg, 0.15 mmol, 1.5 equiv), followed by two prepared Ir complex solutions in the amount as indicated in the following table. Distilled TMPH (35 µL, 0.2 mmol, 2.0 equiv) and TESOTf (29 µL, 0.125 mmol, 1.25 equiv) were added in sequence. The reactions were stirred at r.t. for 17 h. The reaction was diluted with CH_2Cl_2 and filtered through a short plug of silica gel (ca. 1 g) eluting with CH_2Cl_2. The yield was determined by ^{1}H NMR spectroscopy of the crude reaction mixture, using 1,3,5-trimethoxybenzene as the internal standard. The enantiomeric ratio (ee) was determined by HPLC analysis of the purified product.

Entry	Ir/(*S*)-**L** (µL)	Ir/(*R*)-**L** (µL)	ee (%) of complex	yield of **2a** (%)	ee of **2a** (%)
1	50	50	0	95	0
2	60	40	20	92	21
3	70	30	40	99	44
4	80	20	60	90	63
5	90	10	80	96	81
6	100	0	100	92	97

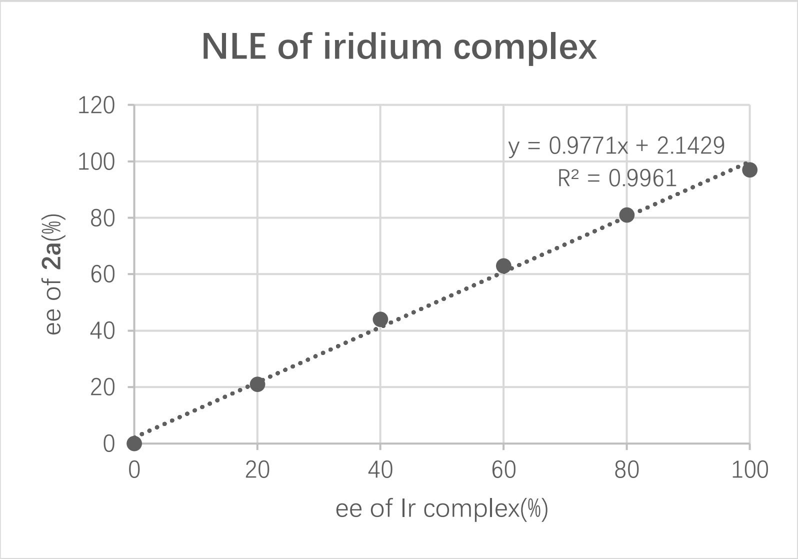

(2) Mixture of (*S*)-L and (*R*)-L ligands

*Preparation of the (S)-**L** ligand stock solution:* In an argon-filled glove box, a vial equipped with a stir bar were charged with phosphoramidite (*S*)-**L** (20.3 mg, 0.04 mmol) and followed by the addition of 1,2-dichloroethane (450 μL) via syringe. The resultant solution was stirred at room temperature for 5 min to give a colorless solution.

*Preparation of the (R)-**L** ligand stock solution:* In an argon-filled glove box, a vial equipped with a stir bar were charged with phosphoramidite (*R*)-**L** (8.1 mg, 0.016 mmol) and followed by the addition of 1,2-dichloroethane (150 μL) via syringe. The resultant solution was stirred at room temperature for 5 min to give a colorless solution.

Ir-catalyzed alkyne trifluoromethylthiolation reaction: To an oven-dried reaction tube was added [Ir(cod)Cl]$_2$ (1.34 mg, 0.002 mmol) followed by two prepared ligand solutions in the amount as indicated in the following table. The resultant solution was stirred at room temperature for one hour. Into the reaction tube were added 1-phenyl-1-butyne (**1a**, 13 mg, 0.1 mmol, 1.0 equiv) and **R6** (44 mg, 0.15 mmol, 1.5 equiv), Distilled TMPH (35 μL, 0.2 mmol, 2.0 equiv) and TESOTf (29 μL, 0.125 mmol, 1.25 equiv). The reactions were stirred at r.t. for 17 h. The reaction was diluted with CH_2Cl_2 and filtered through a short plug of silica gel (ca. 1 g) eluting with CH_2Cl_2. The yield was determined by ^{1}H NMR spectroscopy of the crude reaction mixture, using 1,3,5-trimethoxybenzene as the internal standard. The enantiomeric ratio (ee) was determined by HPLC analysis of the purified product.

Entry	(*S*)-**L** (μL)	(*R*)-**L** (μL)	ee (%) of ligand	yield of **2a** (%)	ee of **2a** (%)
1	50	50	0	77	2
2	60	40	20	81	61
3	70	30	40	87	83
4	80	20	60	86	94
5	90	10	80	78	95
6	100	0	100	92	97

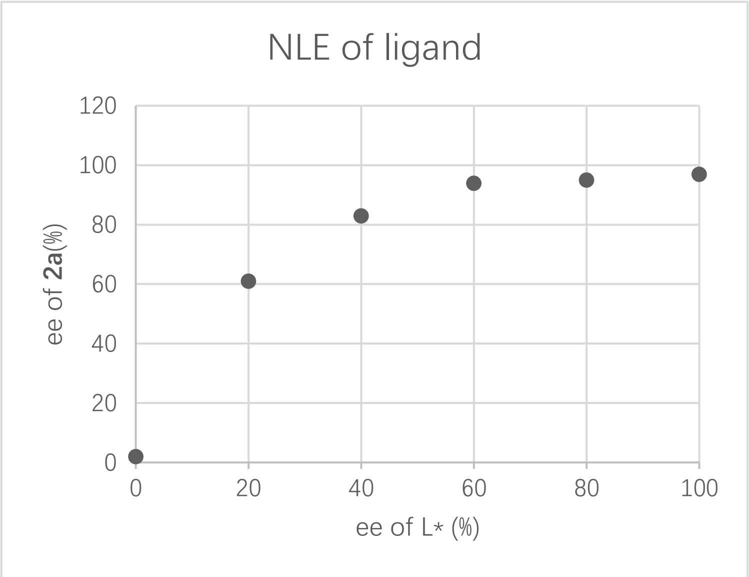

(3) Conclusion

When we mixed the ligands together before formation of the Ir complex, we observed a nonlinear

effect. However, when we first synthesized the (*R*) and (*S*) Ir-complexes first and then mixed them, we observed a linear relationship between the enantiomeric excess (ee) of the product and the ee of the Ir complex. These results suggest that each iridium center coordinates with two ligand molecules and that, once bound, exchange of phosphoramidite ligands between these complexes does not readily occur when stereoisomeric complexes are mixed together.

Nonlinear effect study results

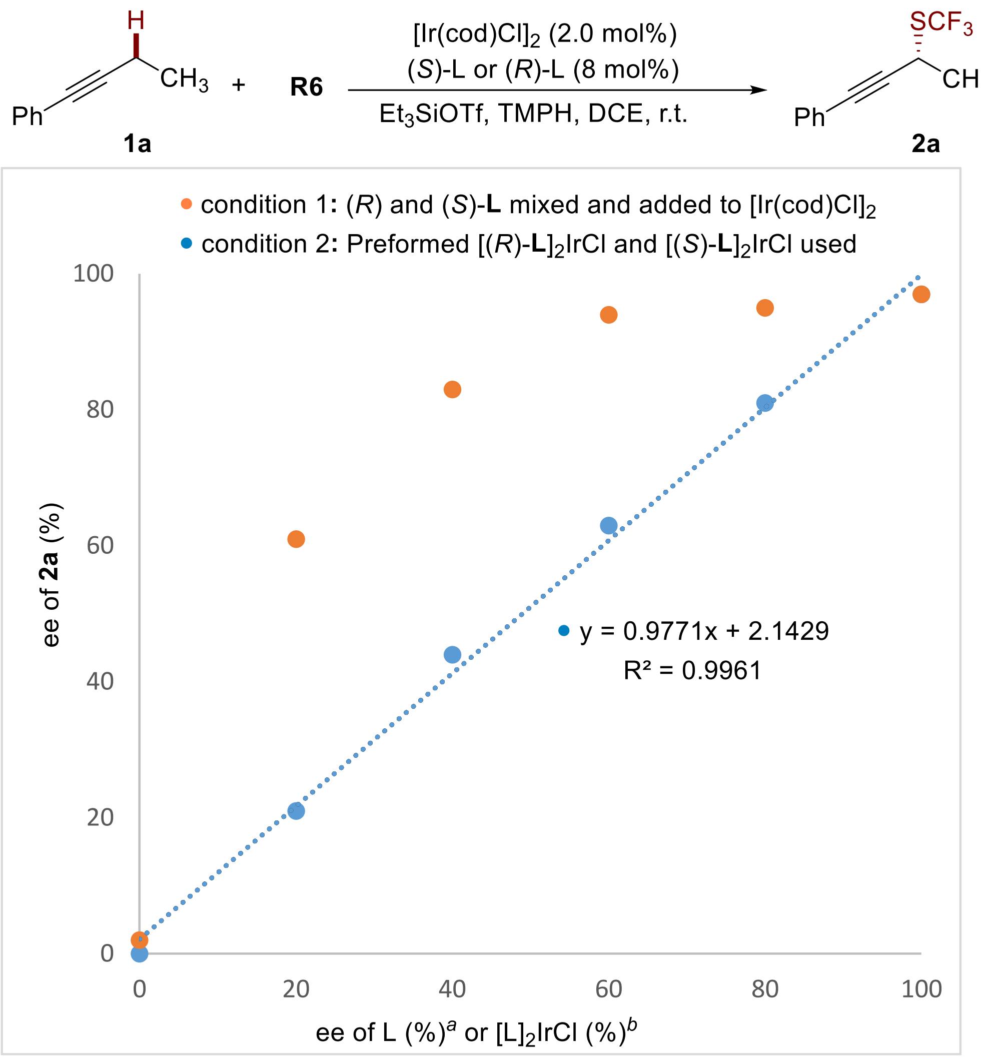

5.3 Control experiments and tests for potential radical intermediates

(1) Control experiments: According to **General Procedure A**

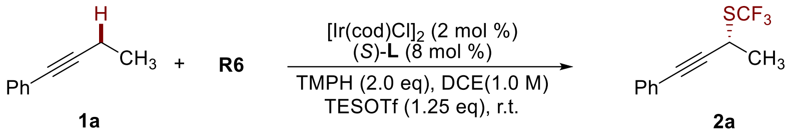

Entry	w/o	Yield (%) **2a**[a,b]	ee of **2a** (%)
1	[Ir(cod)Cl]2	NP	ND
2	(*S*)-L	NP	ND
3	TMPH	NP	ND
4	TESOTf	NP	ND

[a]On 0.2 mmol scale. 1a (0.2 mmol) 1.0 equiv. **R6** 1.5 equiv. [Ir(cod)Cl]2 2.0 mol %, (*S*)-L 8 mol %, TMPH 2.0 equiv, TESOTf 1.25 equiv. and 0.2 mL DCE were used. [b]NP: no desired product observed, ND: not determined.

(2) Tests for potential radical intermediates: According to **General Procedure A**

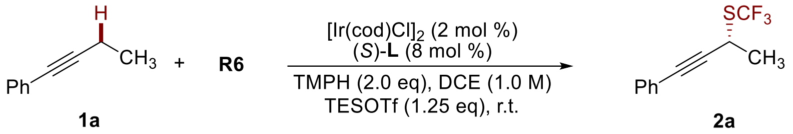

Entry	Radical Scavenger	Yield (%) **2a**[a,b]
1	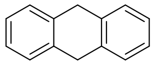	85
2	BHT	60
3	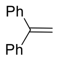	83

[a]On 0.2 mmol scale. 1a (0.2 mmol) 1.0 equiv. **R6** 1.5 equiv. [Ir(cod)Cl]2 2.0 mol %, (*S*)-L 8 mol %, Radical Scavenger 1.0 equiv. TMPH 2.0 equiv, TESOTf 1.25 equiv. and 0.2 mL DCE were used. [b]The yield was determined by ^{1}H NMR spectroscopy of the crude reaction mixture, using 1,3,5-trimethoxybenzene as the internal standard.

5.4 Efforts to detect potential intermediate species

(1) Comparison of ^{31}P NMR spectra during the alkyne coordination step

(1) According to the literature,[1] $[Ir(cod)Cl]_2$ can react with the ligand to form the Cl-complex very easily, observed at 141.60 ppm in the ^{31}P NMR spectrum.

(2) Adding the alkyne (10 equiv) to the Cl-complex does not result in coordination between the alkyne and the Cl-complex, as indicated by the ^{31}P NMR signal remaining at 141.60 ppm.

(3) When TESOTf (20 equiv) is added as a Cl^- trapping reagent, the signal of the alkyne complex is observed at 127.90 and 128.46 ppm in the ^{31}P NMR spectrum. However, this alkyne complex is unstable and difficult to isolate, making it challenging to identify the structure. Therefore, a more strongly coordinating alkyne was used for isolation and characterization of an Ir–alkyne complex.

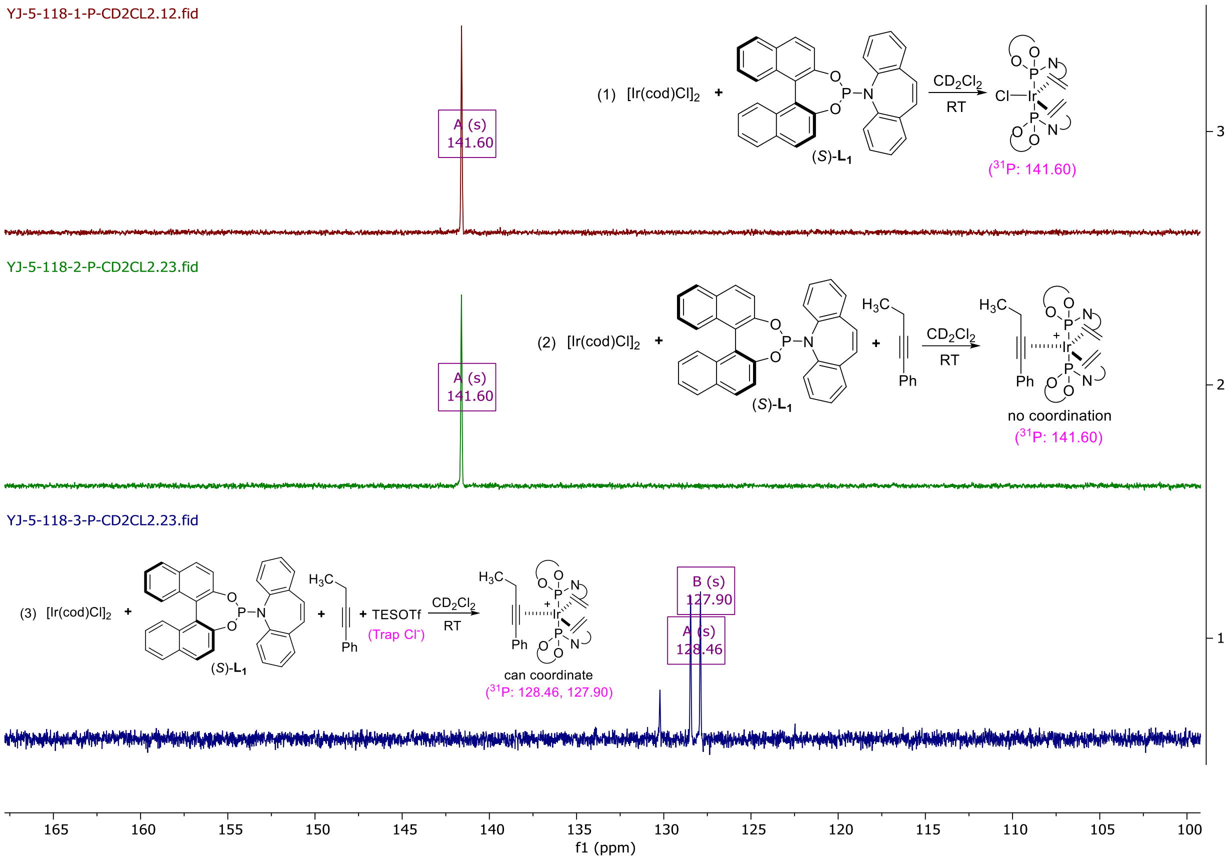

(2) Observations from stoichiometric experiments to obtain stable alkyne complexes

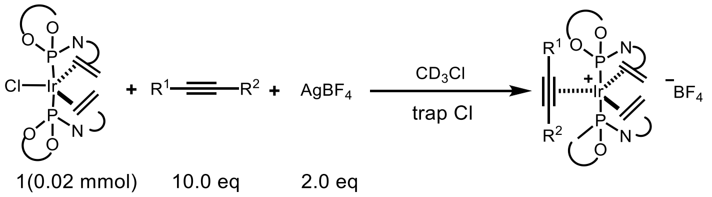

(1) The model alkyne, 1-phenyl-1-butyne, formed an alkyne complex observed at 127.90 and 128.46 ppm in the ^{31}P NMR spectrum.

(2) Another diarylacetylene, 1-phenyl-1-propyne, also formed a complex, with signals at 127.97 and 127.32 ppm in the ^{31}P NMR spectrum.

(3) A dialkylacetylene, 3-hexyne, resulted in a single signal at 128.41 ppm in the ^{31}P NMR spectrum.

(4) The strained alkyne, cyclooctyne (only 1.5 equiv needed), produced a single signal at 128.55 ppm

in the ^{31}P NMR spectrum. Notably, the cyclooctyne Ir-complex was relatively stable, allowing for isolation and identification of its structure spectroscopically and crystallographically.

We have not definitively identified the complex that gives rise to the more downfield signal at ~130 ppm in the first three cases.

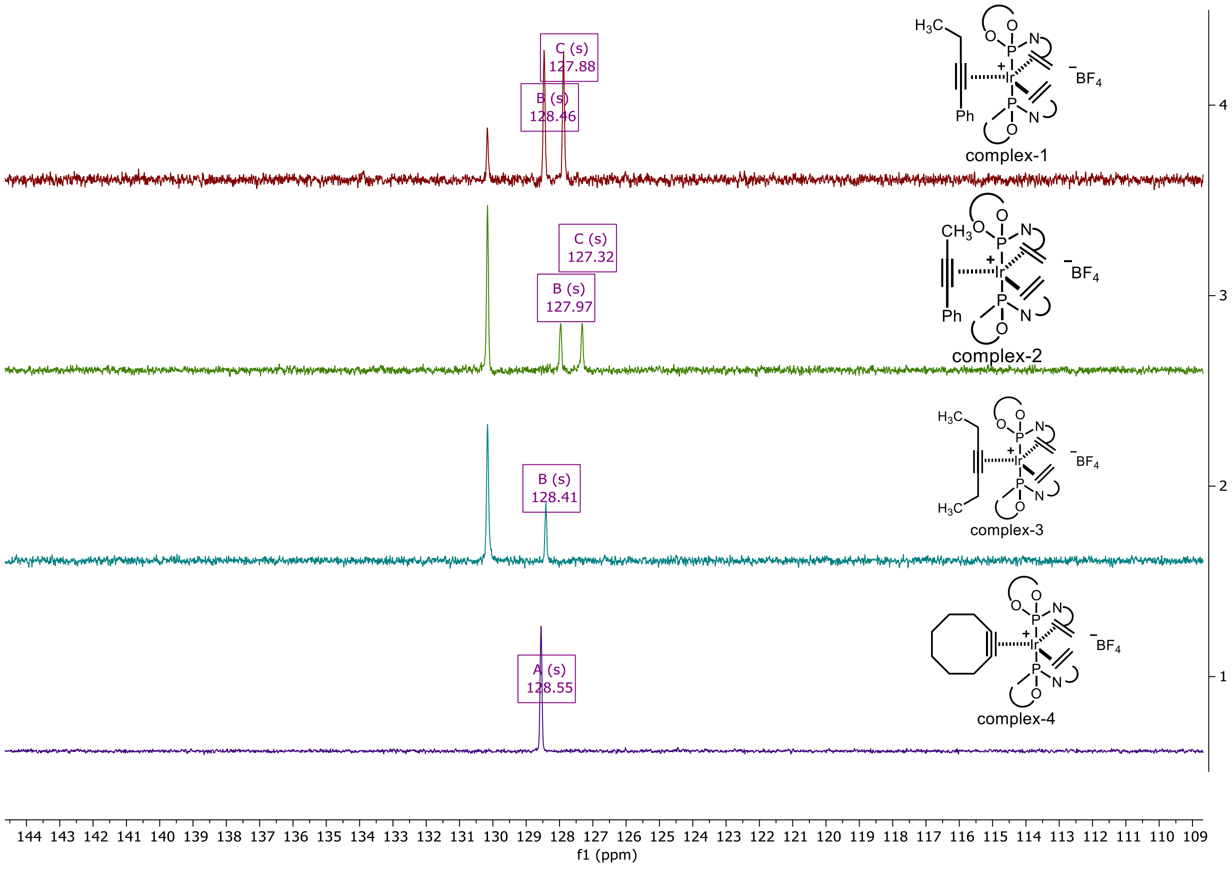

(3) Synthesis and characterization of cyclooctyne-derived complex-4

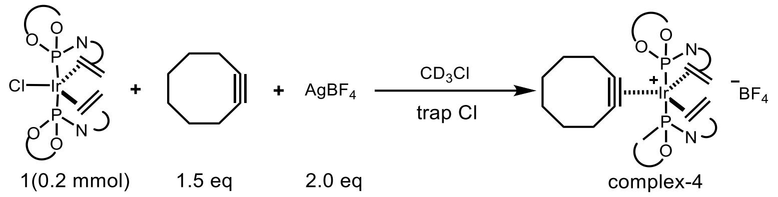

In an argon-filled glovebox, an oven-dried 25 mL round-bottom flask was charged with [((*S*)-L)$_2$Ir]Cl (249.0 mg, 0.20 mmol, 1.0 equiv) and $AgBF_4$ (77.9 mg, 0.40 mmol, 2.0 equiv), followed by the addition of $CDCl_3$ (5.0 mL) via syringe. The resultant solution was stirred at r.t. in the glove box for 5 minutes to give a dark solution. To the solution were added cyclooctyne (32.5 mg, 0.3 mmol, 1.5 equiv). The flask was then capped, and the reaction was stirred at r.t. for 30 minutes. The reaction mixture was concentrated to 1/3 of the initial volume and resulting solution filtered through a syringe filter into and flask containing Et_2O (20 mL). The resulting precipitate was isolated by filtration, and the filter cake was washed with Et_2O (3 × 5 mL) and dried under high vacuum to yield [Ir((*S*)-**L**)$_2$(cyclooctyne)]$^+$$BF_4^-$ (260 mg, 93%). Single crystals suitable for X-ray diffraction were obtained via $PhCF_3$/Et_2O vapor diffusion at –20 °C.

^{1}H NMR (400 MHz, CD_2Cl_2) δ 8.19 (d, *J* = 8.9 Hz, 2H), 8.13 (d, *J* = 8.3 Hz, 2H), 8.01 (t, *J* = 9.0 Hz, 4H), 7.67 – 7.49 (m, 8H), 7.47 – 7.35 (m, 8H), 7.30 (tdd, *J* = 9.1, 7.3, 3.7 Hz, 4H), 7.24 (d, *J* = 7.9 Hz, 2H), 7.19 (d, *J* = 7.6 Hz, 2H), 7.14 – 7.09 (m, 4H), 7.02 (d, *J* = 8.6 Hz, 2H), 6.64 (d, *J* = 8.9 Hz, 2H), 5.87 (dt, *J* = 9.5, 3.5 Hz, 2H), 4.83 (dt, *J* = 9.4, 3.2 Hz, 2H), 1.93 – 1.72 (m, 2H), 1.41 – 1.20 (m, 8H), 1.04 – 0.89 (m, 2H).
^{19}F NMR (376 MHz, CD_2Cl_2) δ -151.35 ($^{10}BF_4^-$), -151.41 ($^{11}BF_4^-$).
^{31}P NMR (162 MHz, CD_2Cl_2) δ 128.55.
^{13}C NMR (101 MHz, CD_2Cl_2) δ 149.1, 148.4, 143.0, 142.4, 138.2, 137.7, 134.7, 134.5, 133.9, 133.8, 133.6, 132.9, 132.7, 132.5, 132.1, 131.9, 130.9, 130.7, 130.4, 130.4, 129.9, 129.4, 128.9, 128.9, 128.7, 128.47, 128.1, 123.9, 122.9, 122.7, 122.4, 65.2, 60.3, 30.7, 29.1, 24.8.
HRMS (ESI) m/z calcd. for $C_{76}H_{56}IrN_2O_4P_2$ $[M]^+$ 1315.3339, found 1315.3346.

^{1}H NMR:

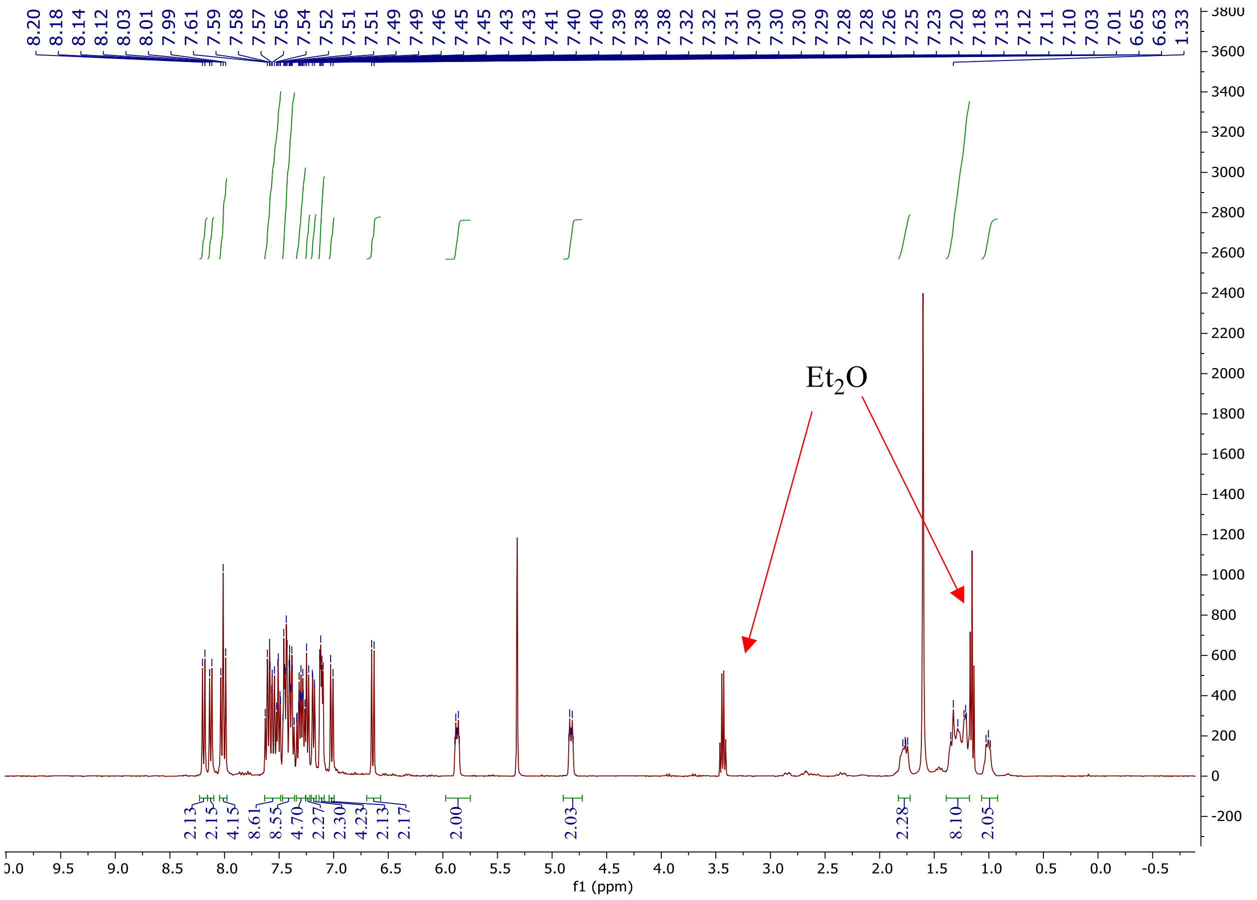

^{19}F NMR:

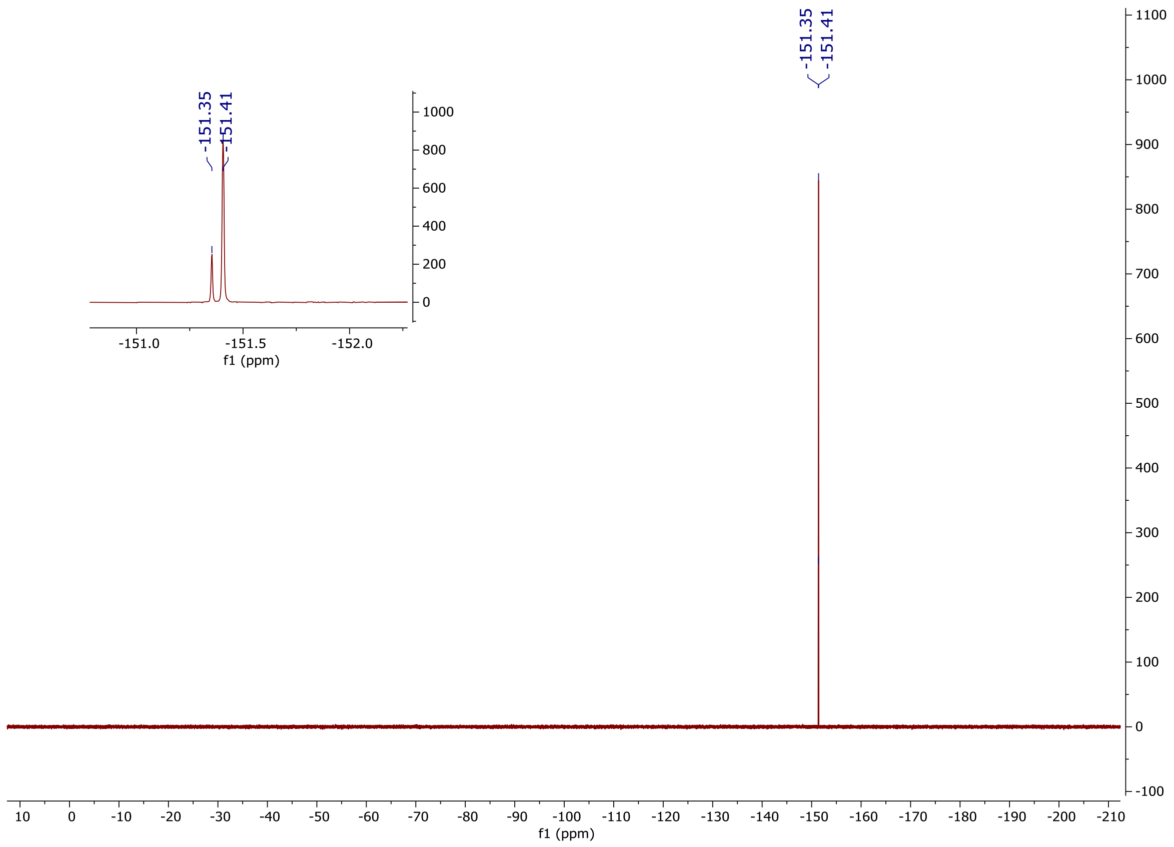

^{31}P NMR:

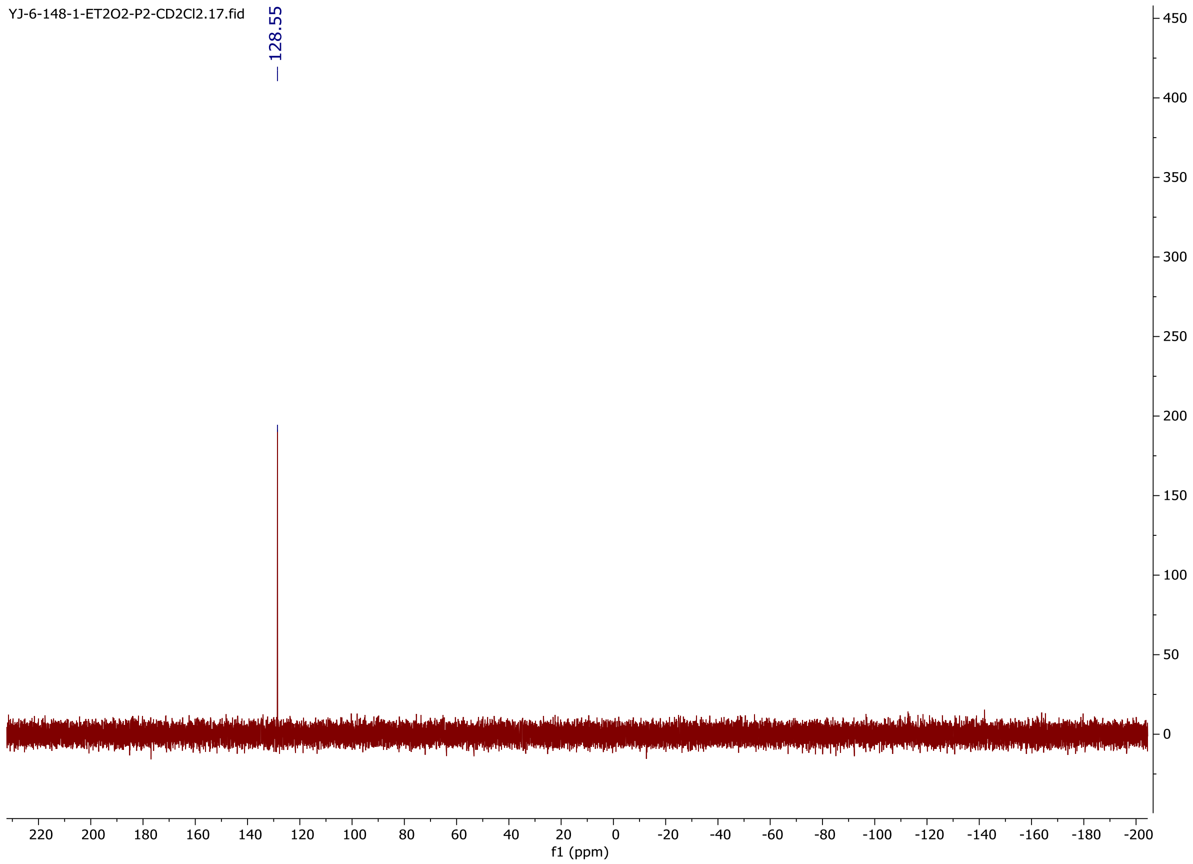

^{13}C NMR:

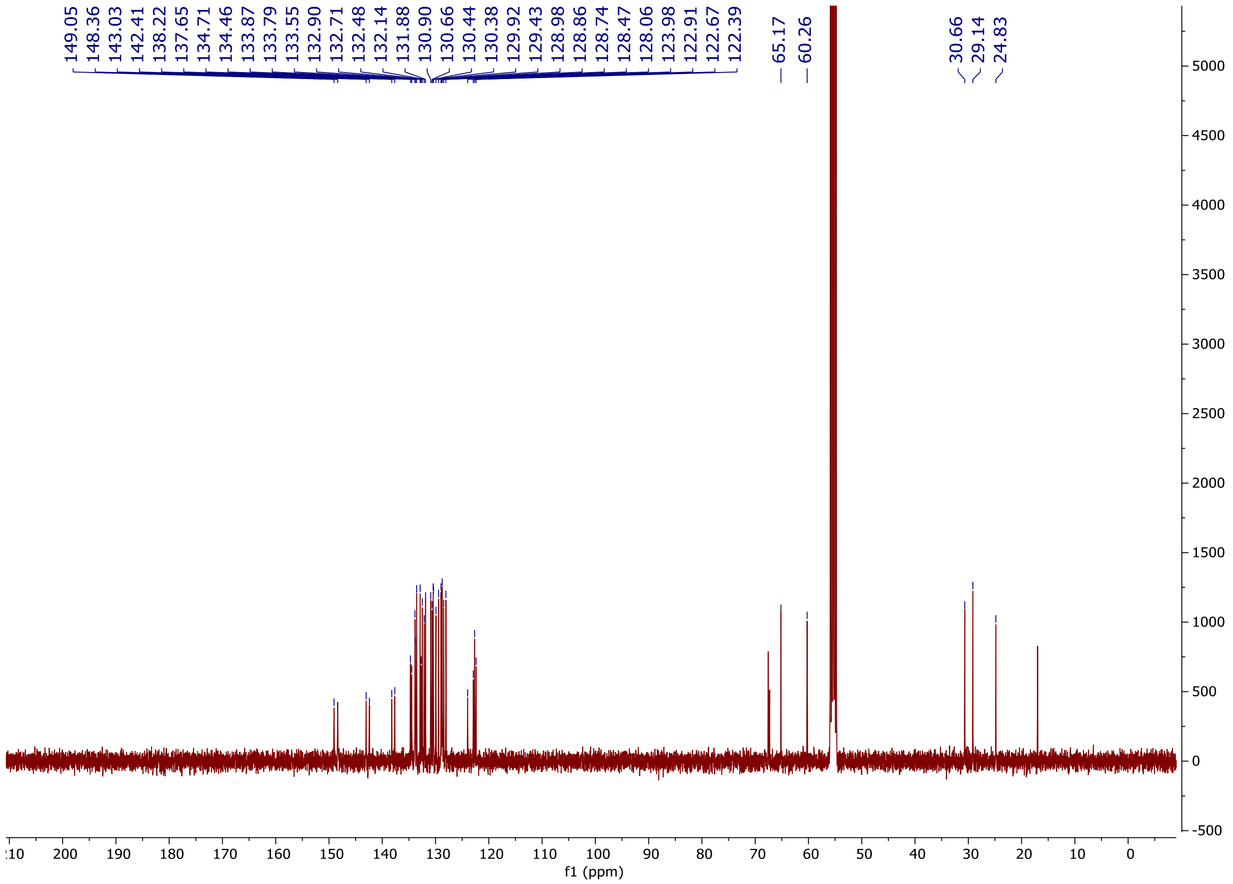

^1H–^1H COSY:

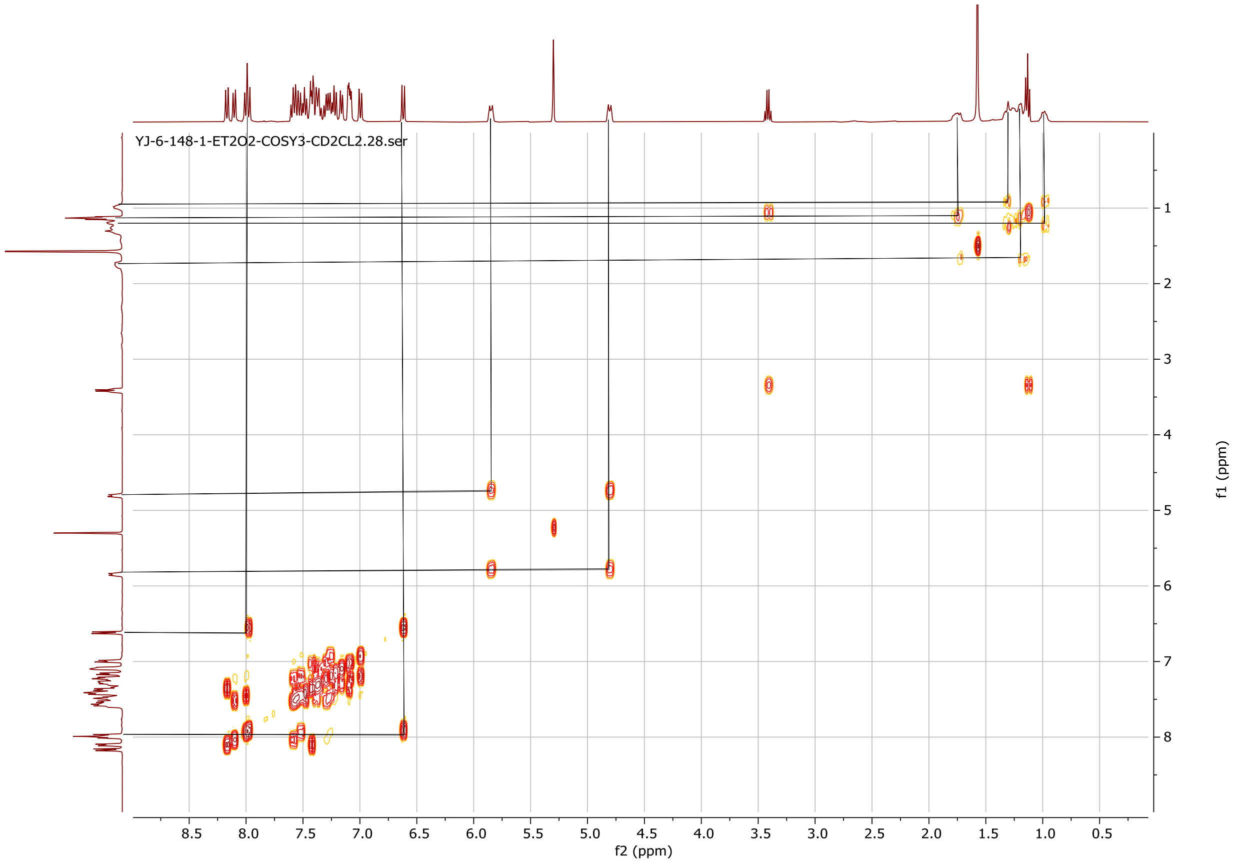

^1H–^{13}C HSQC

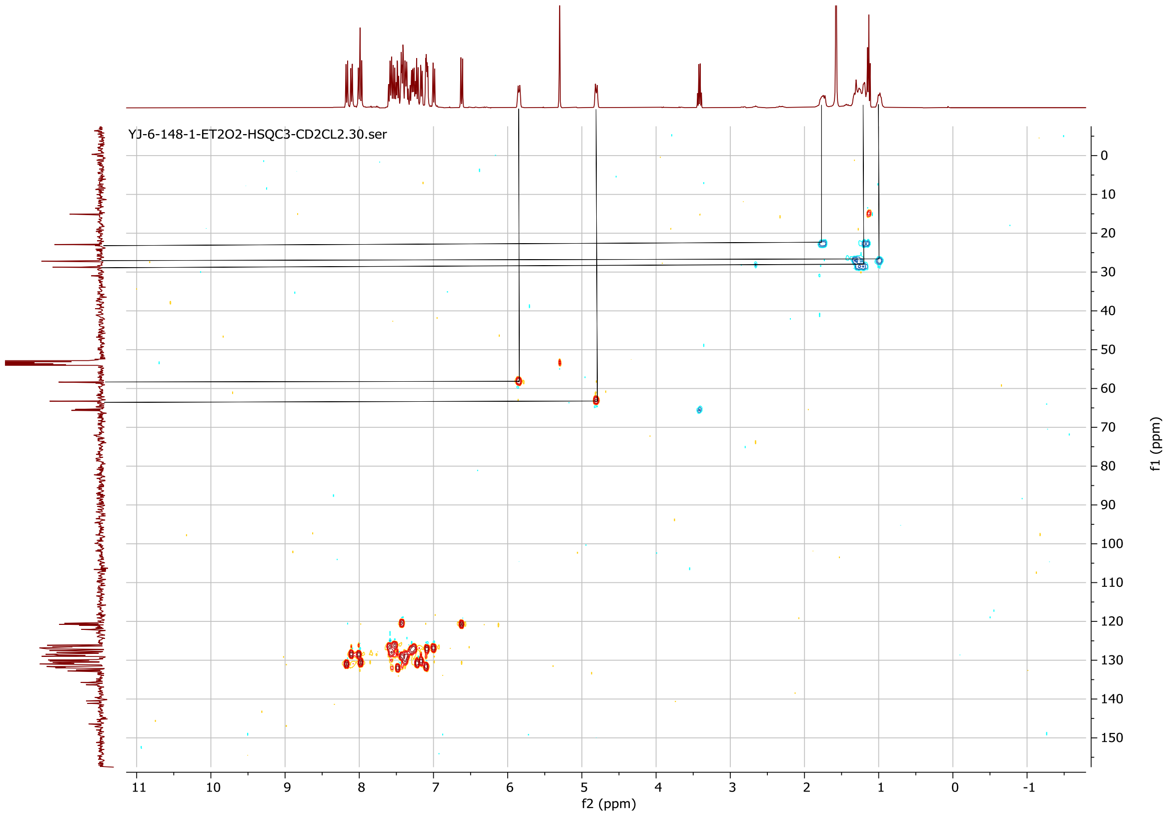

6. Procedures for substrate synthesis and characterization data

6.1 Synthesis of non-Si-substituted alkyne substrates

(**1b–1w**, **3b**, **3d**, **3g**, **3i**, **3j**, **3k**),[2-4] **3e**,[5] **3f**,[6] **3h**,[7] **3l**,[8] **3m**,[9] and **3n**[10] are known compounds and were synthesized according to literature procedures.

The procedures for the synthesis of **3t**, **3u** and **3v** are given below:

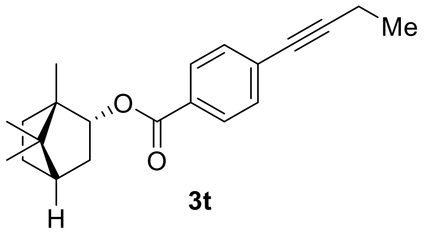

3t

(1*S*,2*R*,4*R*)-1,7,7-Trimethylbicyclo[2.2.1]heptan-2-yl 4-(but-1-yn-1-yl)benzoate (3t): The compound was prepared according to the following procedure.

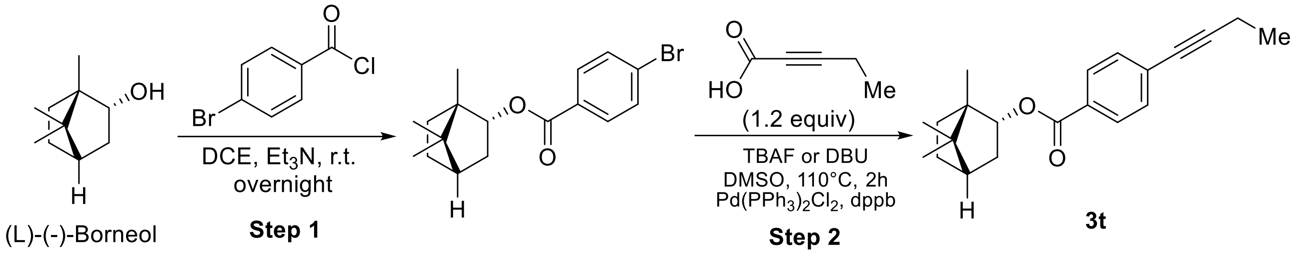

Step 1: Following a literature report,[10] to a solution of (L)-(–)-borneol (1.7 g, 11 mmol, 1.1 equiv) in 1,2-dichloroethane (100 mL) was added 4-bromobenzoyl chloride (2.2 g, 10 mmol, 1.0 equiv) and Et_3N (4.2 mL, 30 mmol, 3.0 equiv). The resulting mixture was stirred at room temperature overnight, after which the reaction mixture was diluted with H_2O and extracted with EtOAc (3 × 60 mL). The organic layer was dried ($MgSO_4$) and concentrated *in vacuo*. The crude material was purified by column chromatography to afford the aryl bromide as a pale yellow oil (485 mg, 14%).

Step 2: To a round bottom flask were added the aryl bromide (480 mg, 1.4 mmol, 1.0 equiv), 2-pentynoic acid (170 mg, 1.7 mmol, 1.2 equiv), $PdCl_2(PPh_3)_2$ (10 mg, 0.014 mmol, 1 mol %), 1,4-bis(diphenylphosphino)butane (dppb, 12 mg, 0.028 mmol, 2 mol %). The flask was then evacuated and refilled with N_2. Anhydrous DMSO (0.5 M) was then added, followed by the addition of tetra-*n*-butylammonium fluoride (TBAF, 1 M in THF, 3 mL, 3.0 mmol, 2.0 equiv). The reaction mixture was stirred at 110 °C for 5 h. It was then cooled to room temperature and quenched by addition of saturated NH_4Cl solution (10 mL). The product was extracted by Et_2O (3 × 10 mL), and the combined organic layers were washed with brine. The organic phase was further dried over $MgSO_4$ and concentrated. The crude mixture was purified by vacuum distillation or flash column chromatography to afford the title compound **3v** (382 mg, 82%) as a pale yellow oil.

^{1}H NMR (400 MHz, $CDCl_3$) δ 7.96 (d, J = 8.4 Hz, 2H), 7.45 (d, J = 8.4 Hz, 2H), 5.18 – 4.98 (m, 1H), 2.54 – 2.39 (m, 3H), 2.17 – 2.07 (m, 1H), 1.86 – 1.76 (m, 1H), 1.73 (t, J = 4.5 Hz, 1H), 1.47 – 1.36 (m, 1H), 1.35 – 1.28 (m, 1H), 1.25 (t, J = 7.5 Hz, 3H), 1.11 (dd, J = 13.8, 3.5 Hz, 1H), 0.96 (s, 3H), 0.91 (d, J = 3.0 Hz, 6H).

^{13}C NMR (100 MHz, $CDCl_3$) δ 166.4, 131.4, 129.6, 129.3, 128.7, 94.9, 80.7, 79.5, 49.1, 47.9, 45.0, 36.9, 28.1, 27.4, 19.7, 18.9, 13.7, 13.6, 13.2.

HRMS (ESI) calcd for $C_{21}H_{27}O_2$ $[M+H]^+$: 311.2006, found: 311.2010.

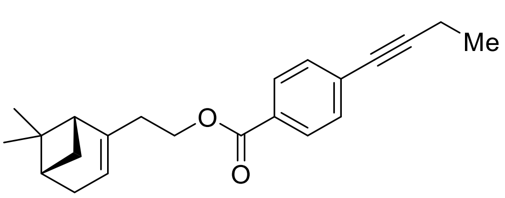
3u

2-((1*R*,5*S*)-6,6-Dimethylbicyclo[3.1.1]hept-2-en-2-yl)ethyl 4-(but-1-yn-1-yl)benzoate (**3u**): The compound was prepared according to the following procedure.

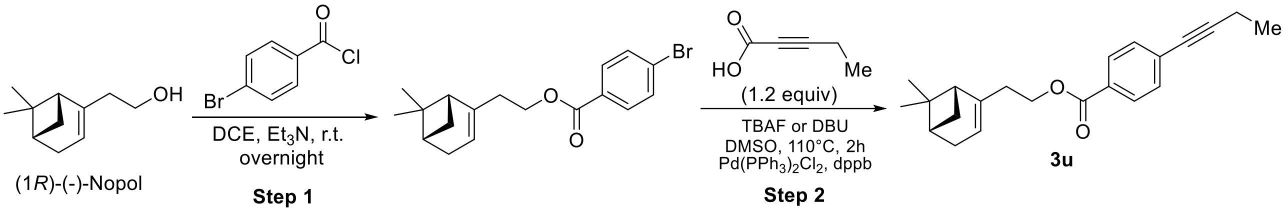

Step 1**:** Following a literature report,[11] to a solution of (1*R*)-(-)-nopol (1.8 g, 11 mmol, 1.1 equiv) in 1,2-dichloroethane (100 mL) was added 4-bromobenzoyl chloride (2.2 g, 10 mmol, 1.0 equiv) and Et_3N (4.2 mL, 30 mmol, 3.0 equiv). The resulting mixture was stirred at room temperature overnight, after which the reaction mixture was diluted with H_2O and extracted with EtOAc (3 × 60 mL). The organic layer was dried ($MgSO_4$) and concentrated *in vacuo*. The crude material was purified by column chromatography to afford the aryl bromide as a colorless oil (3.2 g, 91%).
Step 2**:** To a round bottom flask were added the aryl bromide (1.40 g, 4.0 mmol, 1.0 equiv), 2-pentynoic acid (470 mg, 4.8 mmol, 1.2 equiv), $PdCl_2(PPh_3)_2$ (28 mg, 0.04 mmol, 1 mol %), 1,4-bis(diphenylphosphino)butane (dppb, 34 mg, 0.08 mmol, 2 mol %). The flask was then evacuated and refilled with N_2. Anhydrous DMSO (0.5 M) was then added, followed by the addition of tetra-*n*-butylammonium fluoride (TBAF, 1 M in THF, 3 mL, 8.0 mmol, 2.0 equiv). The reaction mixture was stirred at 110 °C for 5 h. It was then cooled to room temperature and quenched by addition of saturated NH_4Cl solution (10 mL). The product was extracted by Et_2O (3 × 30 mL), and the combined organic layers were washed with brine. The organic phase was further dried over $MgSO_4$ and concentrated. The crude mixture was purified by vacuum distillation or flash column chromatography to afford the title compound **3u** (999 mg, 78%) as a pale yellow oil.
^{1}H NMR (400 MHz, $CDCl_3$) δ 7.93 (d, J = 8.4 Hz, 2H), 7.43 (d, J = 8.4 Hz, 2H), 5.50 – 5.11 (m, 1H), 4.48 – 4.07 (m, 2H), 2.55 – 2.33 (m, 5H), 2.32 – 2.14 (m, 2H), 2.15 – 2.00 (m, 2H), 1.29 – 1.21 (m, 6H), 1.16 (d, J = 8.5 Hz, 1H), 0.82 (s, 3H).
^{13}C NMR (100 MHz, $CDCl_3$) δ 166.1, 144.2, 131.4, 129.4, 129.2, 128.8, 118.9, 95.0, 79.5, 63.4, 45.8, 40.8, 38.0, 36.1, 31.7, 31.4, 26.3, 21.1, 13.7, 13.2.
HRMS (ESI) calcd for $C_{22}H_{27}O_2$ $[M+H]^+$: 323.2006, found: 323.2009.

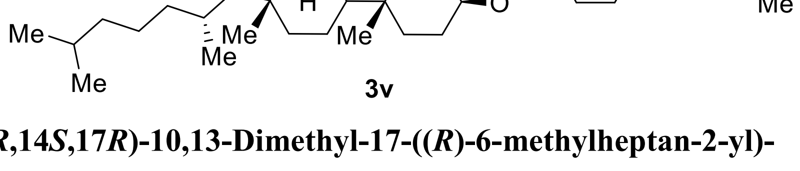
3v

(3*S*,8*S*,9*S*,10*R*,13*R*,14*S*,17*R*)-10,13-Dimethyl-17-((*R*)-6-methylheptan-2-yl)-

2,3,4,7,8,9,10,11,12,13,14,15,16,17-tetradecahydro-1*H*-cyclopenta[a]phenanthren-3-yl 4-(but-1-yn-1-yl)benzoate (3v): The compound was prepared according to the following procedure.

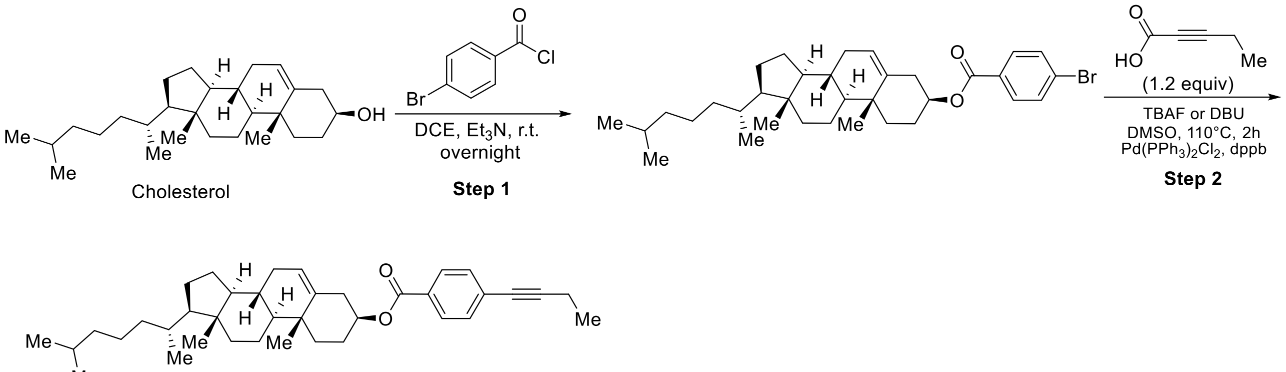

3v

***Step 1*:** Following a literature report,[11] to a solution of cholesterol (2.1 g, 5.5 mmol, 1.1 equiv) in 1,2-dichloroethane (50 mL) was added 4-bromobenzoyl chloride (1.1 g, 5.0 mmol, 1.0 equiv) and Et_3N (2.1 mL, 15 mmol, 3.0 equiv). The resulting mixture was stirred at room temperature overnight, after which time the reaction mixture was diluted with H_2O and extracted with EtOAc (3 × 50 mL). The organic layer was dried ($MgSO_4$) and concentrated in vacuo. The crude material was purified by column chromatography to afford the aryl bromide (1.1 g, 39%) as a white solid.

***Step 2*:** To a round bottom flask were added the aryl bromide (855 mg, 1.5 mmol, 1.0 equiv), 2-pentynoic acid (176 mg, 1.8 mmol, 1.2 equiv), $PdCl_2(PPh_3)_2$ (11 mg, 0.015 mmol, 1 mol %), 1,4-bis(diphenylphosphino)butane (dppb, 13 mg, 0.03 mmol, 2 mol %). The flask was then evacuated and refilled with N_2. Anhydrous DMSO (0.5 M) was then added, followed by the addition of tetra-*n*-butylammonium fluoride (TBAF, 1 M in THF, 3 mL, 3.0 mmol, 2.0 equiv). The reaction mixture was stirred at 110 °C for 5 h. It was then cooled to room temperature and quenched by addition of saturated NH_4Cl solution (10 mL). The product was extracted by Et_2O (3 × 30 mL), and the combined organic layers were washed with brine. The organic phase was further dried over $MgSO_4$ and concentrated. The crude mixture was purified by vacuum distillation or flash column chromatography to afford the title compound **3v** (704 mg, 87%) as a white solid.

^{1}H NMR (400 MHz, $CDCl_3$) δ 7.95 (d, *J* = 8.4 Hz, 2H), 7.43 (d, *J* = 8.4 Hz, 2H), 5.76 – 5.37 (m, 1H), 5.12 – 4.60 (m, 1H), 2.53 – 2.30 (m, 4H), 2.09 – 1.65 (m, 6H), 1.63 – 0.95 (m, 28H), 0.92 (d, *J* = 6.5 Hz, 3H), 0.87 (dd, *J* = 6.5, 1.8 Hz, 6H), 0.69 (s, 3H).

^{13}C NMR (100 MHz, $CDCl_3$) δ 165.6, 139.6, 131.4, 129.6, 129.4, 128.7, 122.8, 94.9, 79.5, 74.7, 56.7, 56.2, 53.4, 50.1, 42.3, 39.8, 39.5, 38.2, 37.0, 36.7, 36.2, 35.8, 31.9, 31.9, 28.2, 28.0, 27.9, 24.3, 23.85, 22.8, 22.6, 21.1, 19.4, 18.7, 13.7, 13.2, 11.9.

HRMS (ESI) calcd for $C_{38}H_{55}O_2$ $[M+H]^+$: 543.4197, found: 543.4213.

m.p.: 141-142 °C

6.2 Synthesis of Si(OMe)3-protected alkyne substrates 3o, 3p

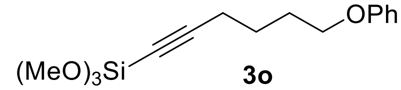

3o

Trimethoxy(6-phenoxyhex-1-yn-1-yl)silane (**3o**):

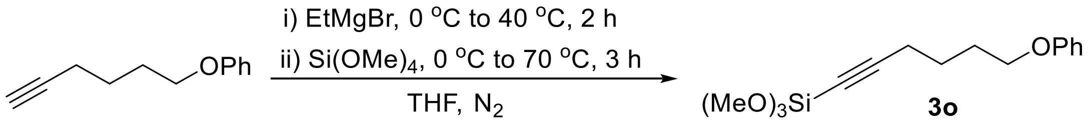

Following a literature report,[12] to a solution of (hex-5-yn-1-yloxy)benzene (1.74 g, 10 mmol, 1.0 equiv) in THF (9 mL) was slowly added ethylmagnesium bromide (3.2 M solution in THF, 3.3 mL, 11 mmol, 1.1 equiv) at 0 °C under Ar, then the mixture was heated up to 40 ºC and stirred for 2 h. After cooling to room temperature, tetramethoxysilane (2.3 mL, 15 mmol, 1.5 equiv) was added into the reaction mixture at 0 °C, and the reaction mixture was heated up to 70 °C and stirred for another 3 h. After cooling to room temperature, THF was removed and dry Et_2O (30 mL) was poured into the residue, and the mixture was then stirred at room temperature for 1 h. The solid material was filtered out and Et_2O was removed, and the product was purified from the residue by distillation under high vacuum, yielding the title compound **3o** as a colorless liquid (420 mg, 14% yield).

^{1}H NMR (400 MHz, $CDCl_3$) δ 7.32 – 7.27 (m, 2H), 6.94 (t, J = 7.3 Hz, 1H), 6.89 (d, J = 7.7 Hz, 2H), 3.99 (t, J = 6.2 Hz, 2H), 3.59 (s, 9H), 2.37 (t, J = 7.0 Hz, 2H), 2.04 – 1.87 (m, 2H), 1.83 – 1.63 (m, 2H).

^{13}C NMR (100 MHz, $CDCl_3$) δ 158.9, 129.5, 120.7, 114.5, 107.6, 74.9, 67.0, 50.8, 28.4, 24.9, 19.4.

HRMS (ESI) calcd for $C_{15}H_{23}O_4Si$ $[M+H]^+$: 295.1360, found: 295.1363.

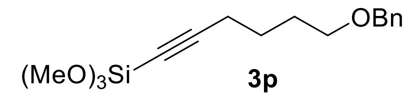

(6-(Benzyloxy)hex-1-yn-1-yl)trimethoxysilane (3p):

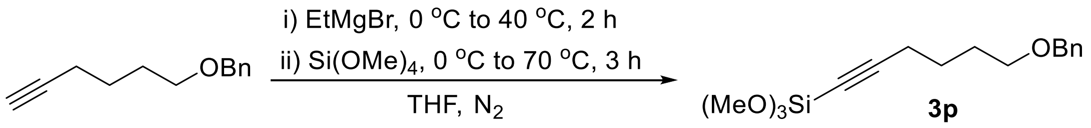

Following a literature report,[12] to a solution of ((hex-5-yn-1-yloxy)methyl)benzene (1.75 g, 9.3 mmol, 1.0 equiv) in THF (9 mL) was slowly added ethylmagnesium bromide (3.2 M solution in THF, 2.9 mL, 9.3 mmol, 1.0 equiv) at 0 °C under Ar, then the mixture was heated up to 40 °C and stirred for 2 h. After cooling to room temperature, tetramethoxysilane (2.1 mL, 14 mmol, 1.5 equiv) was added into the reaction mixture at 0 °C, and the reaction was heated up to 70 ºC and stirred for another 3 h. After cooling to room temperature, THF was removed and dry Et_2O (30 mL) was poured into the residue, the mixture was then stirred at room temperature for 1 h. The solid material was filtered out and Et_2O was removed, and the product was purified from the residue by distillation under high vacuum, yielding the title compound **3p** as a pale yellow liquid (529 mg, 18% yield).

^{1}H NMR (400 MHz, $CDCl_3$) δ 7.46 – 7.27 (m, 5H), 4.50 (s, 2H), 3.58 (s, 9H), 3.49 (t, J = 6.1 Hz, 2H), 2.31 (t, J = 6.9 Hz, 2H), 1.86 – 1.63 (m, 4H).

^{13}C NMR (100 MHz, $CDCl_3$) δ 138.5, 128.4, 127.6, 127.6, 107.9, 74.7, 72.9, 69.6, 50.8, 28.8, 25.0, 19.4.

HRMS (ESI) calcd for $C_{16}H_{25}O_4Si$ $[M+H]^+$: 309.1517, found: 309.1519.

7. Procedure for R8 synthesis and characterization data

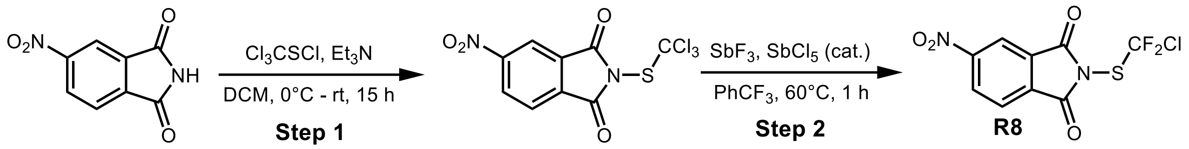

Step 1**: 5-Nitro-2-((trichloromethyl)thio)isoindoline-1,3-dione**: To a stirred mixture of 5-nitroisoindoline-1,3-dione (1.92 g, 10.0 mmol, 1.0 equiv) and triethylamine (2.09 mL, 1.52 g, 15.0 mmol, 1.5 equiv) in CH_2Cl_2 (20 mL) at 0 °C under nitrogen atmosphere was slowly added perchloromethyl mercaptan (1.30 mL, 2.23 g, 12.0 mmol, 1.2 equiv) dropwise. The reaction mixture was allowed to warm to rt over 15 h. Upon completion, the reaction was quenched with water (50 mL), and the aqueous phase was extracted with CH_2Cl_2 (4 × 20 mL). The combined organic phases were washed with saturated $NaHCO_3$ (2 × 20 mL) and brine (50 mL), dried over anhydrous sodium sulfate, and concentrated in *vacuo*. The crude mixture was recrystallized from CH_2Cl_2/hexanes to afford 5-nitro-2-((trichloromethyl)thio)isoindoline-1,3-dione as a pale-yellow solid (3.11 g, 91 % yield).

Step 2**: 2-((Chlorodifluoromethyl)thio)-5-nitroisoindoline-1,3-dione:** To a stirred mixture 5-nitro-2-((trichloromethyl)thio)isoindoline-1,3-dione (3.42 g, 10.0 mmol, 1.0 equiv) and SbF_3 (3.58 g, 20.0 mmol, 2.0 equiv) in $PhCF_3$ (10 mL) at 60 °C under argon atmosphere was slowly added $SbCl_5$ (63.4 μL, 149.5 mg, 0.5 mmol, 5 mol %) dropwise. The reaction mixture was heated at that temperature for 1 h. Upon completion, the reaction was diluted with CH_2Cl_2 (90 mL), filtered through a plug of silica gel, and the filtrate was concentrated in *vacuo*. The crude mixture was purified via column chromatography on silica gel (gradient from 5% EtOAc in hexanes to 30% EtOAc in hexanes) then recrystallized from hot Et_2O to afford the 2-((chlorodifluoromethyl)thio)-5-nitroisoindoline-1,3-dione as a white solid (1.62 g, 52 % yield).

^{1}H NMR (400 MHz, $CDCl_3$) δ 8.82 (dd, *J* = 2.0, 0.6 Hz, 1H), 8.73 (dd, *J* = 8.2, 2.0 Hz, 1H), 8.23 (dd, *J* = 8.2, 0.6 Hz, 1H).

^{19}F NMR (376 MHz, $CDCl_3$) δ -34.74 (d, *J* = 2.1 Hz, 2F).

^{13}C NMR (100 MHz, $CDCl_3$) δ 163.9, 163.7, 152.4, 135.6, 132.8, 130.4, 129.1 (t, *J* = 331.1 Hz), 126.6, 120.1.

HRMS (ESI) calcd for $C_9H_4O_4N_2ClF_2S$ $[M+H]^+$: 308.9543, found: 308.9557.

m.p.: 89-90 °C

^{1}H NMR:

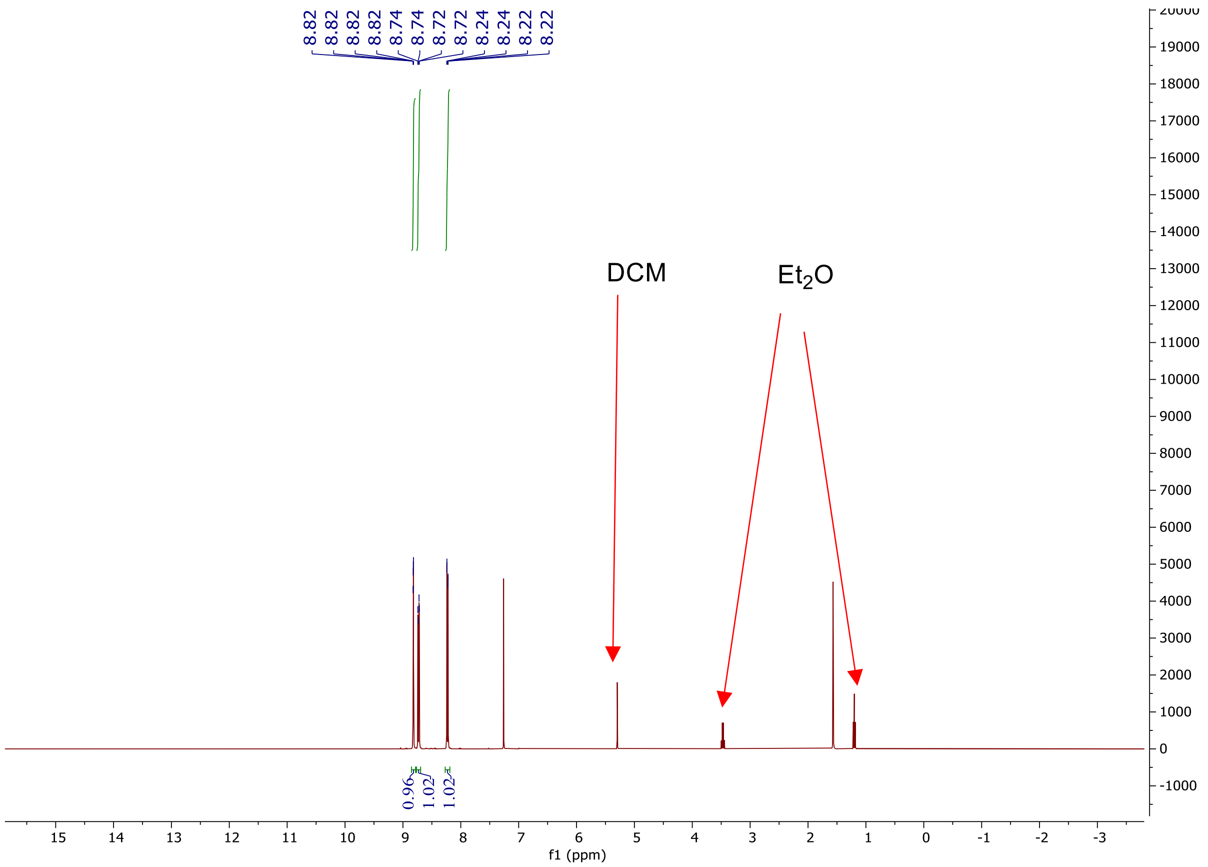

^{19}F NMR:

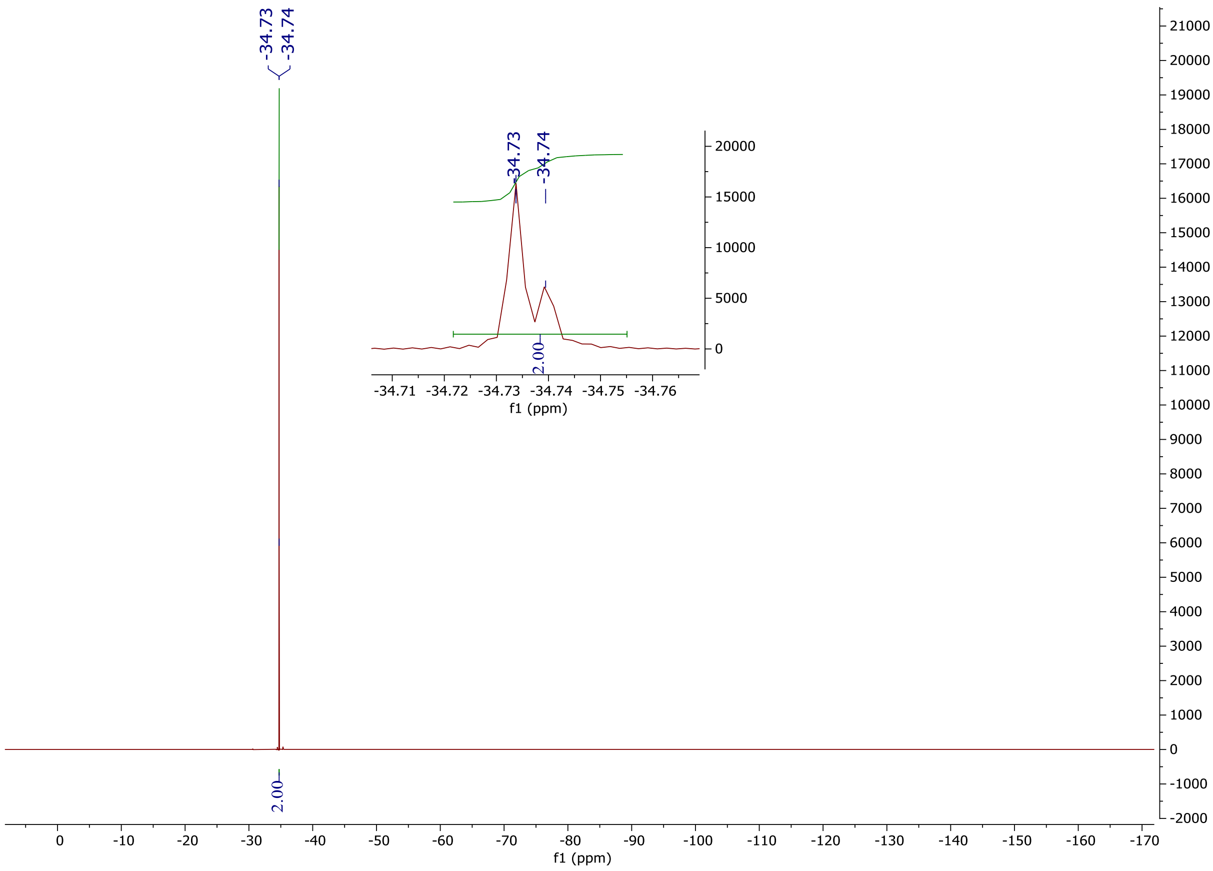

^{13}C NMR:

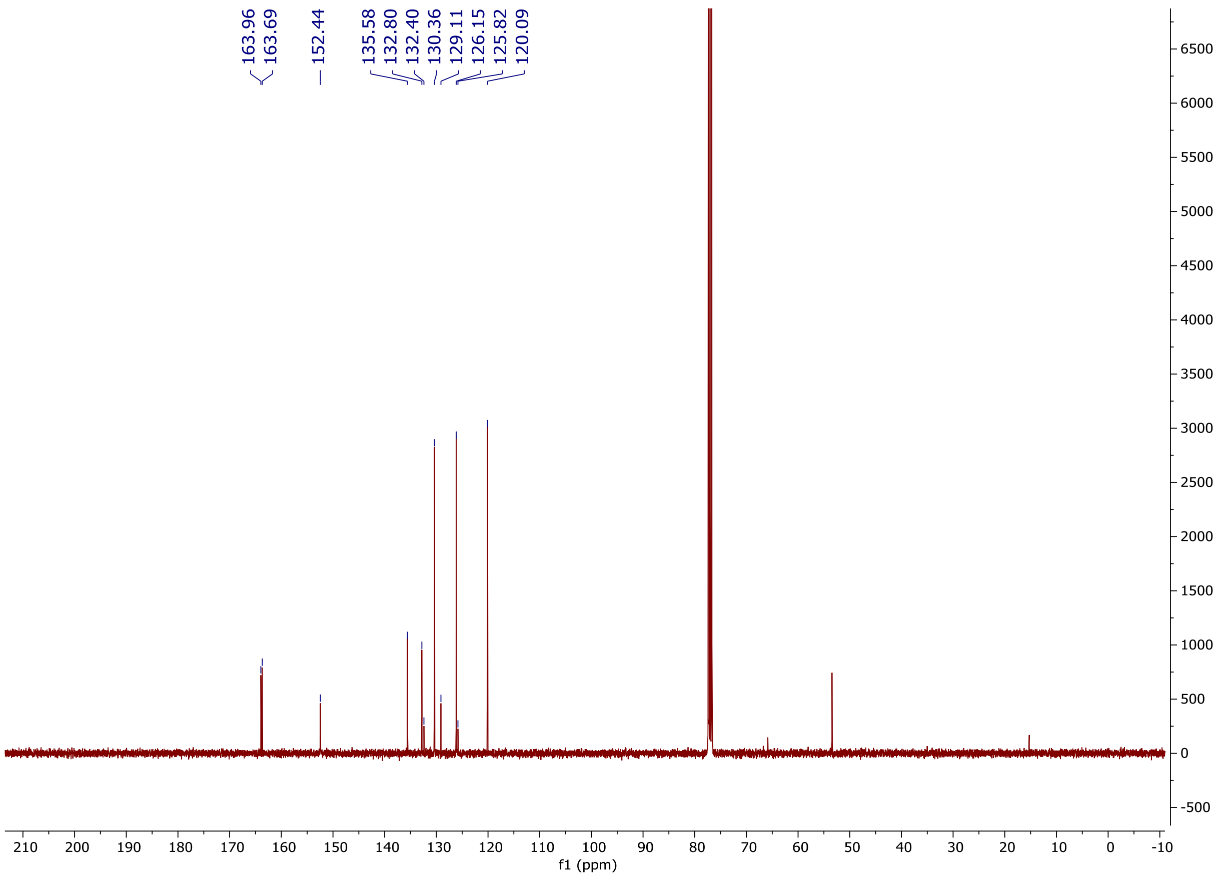
163.96
163.69
152.44
135.58
132.80
132.40
130.36
129.11
126.15
125.82
120.09
f1 (ppm)

8. X-ray structure of 2f and iridium cyclooctyne complex

X-ray structure of **2f**:

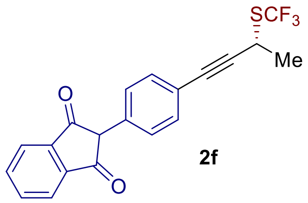

≡

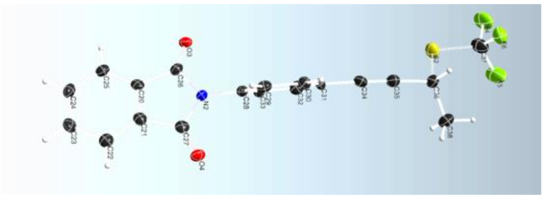

Datablock: mo_JiaoYu2_a

Bond precision: C-C = 0.0109 A Wavelength=0.71073

Cell:	a=5.4692(10)	b=16.595(2)	c=18.705(3)
	alpha=90	beta=92.234(7)	gamma=90
Temperature:	100 K		

	Calculated	Reported
Volume	1696.4(5)	1696.4(5)
Space group	P 21	P 21
Hall group	P 2yb	P 2yb
Moiety formula	C19 H12 F3 N O2 S	?
Sum formula	C19 H12 F3 N O2 S	C19 H12 F3 N O2 S
Mr	375.36	375.36
Dx,g cm-3	1.470	1.470
Z	4	4
Mu (mm-1)	0.235	0.235
F000	768.0	768.0
F000'	768.97	
h,k,lmax	6,20,22	6,20,22
Nref	6488[3363]	6457
Tmin,Tmax	0.994,0.999	0.980,0.990
Tmin'	0.984	

Correction method= # Reported T Limits: Tmin=0.980 Tmax=0.990
AbsCorr = MULTI-SCAN

Data completeness= 1.92/1.00 Theta(max)= 25.735

R(reflections)= 0.0597(4030) wR2(reflections)= 0.1572(6457)

S = 0.868 Npar= 472

X-ray structure of **iridium cyclooctyne complex**:

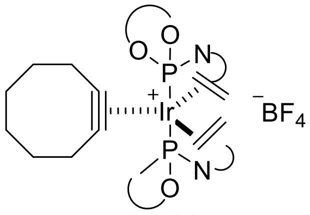

complex-IV

≡

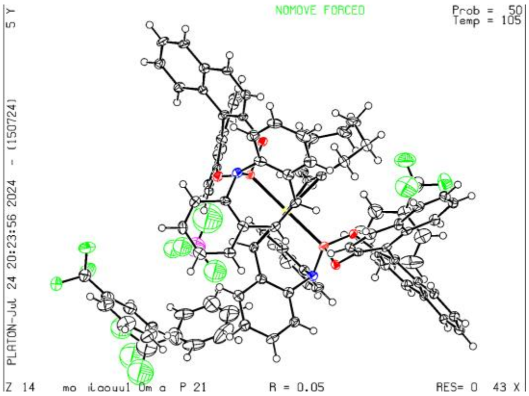

Datablock: mo_jiaoyu1_0m_a

Bond precision:	C-C = 0.0167 A		Wavelength=0.71073
Cell:	a=13.161(3)	b=21.426(5)	c=15.080(3)
	alpha=90	beta=91.744(5)	gamma=90
Temperature:	105 K		

	Calculated	Reported
Volume	4250.4(16)	4250.5(16)
Space group	P 21	P 21
Hall group	P 2yb	P 2yb
Moiety formula	C76 H56 Ir N2 O4 P2, 3(C7 H5 F3), B F4	?
Sum formula	C97 H71 B F13 Ir N2 O4 P2	C97 H71 B F13 Ir N2 O4 P2
Mr	1840.53	1840.50
Dx,g cm-3	1.438	1.438
Z	2	2
Mu (mm-1)	1.690	1.690
F000	1856.0	1856.0
F000’	1854.49	
h,k,lmax	15,25,18	15,25,18
Nref	15651[8052]	15506
Tmin,Tmax	0.904,0.995	0.860,0.990
Tmin’	0.859	

Correction method= # Reported T Limits: Tmin=0.860 Tmax=0.990
AbsCorr = MULTI-SCAN

Data completeness= 1.93/0.99 Theta(max)= 25.402

R(reflections)= 0.0469(14388) wR2(reflections)= 0.1335(15506)

S = 1.044 Npar= 1040

9. Copies of NMR spectra of products

^{1}H NMR:

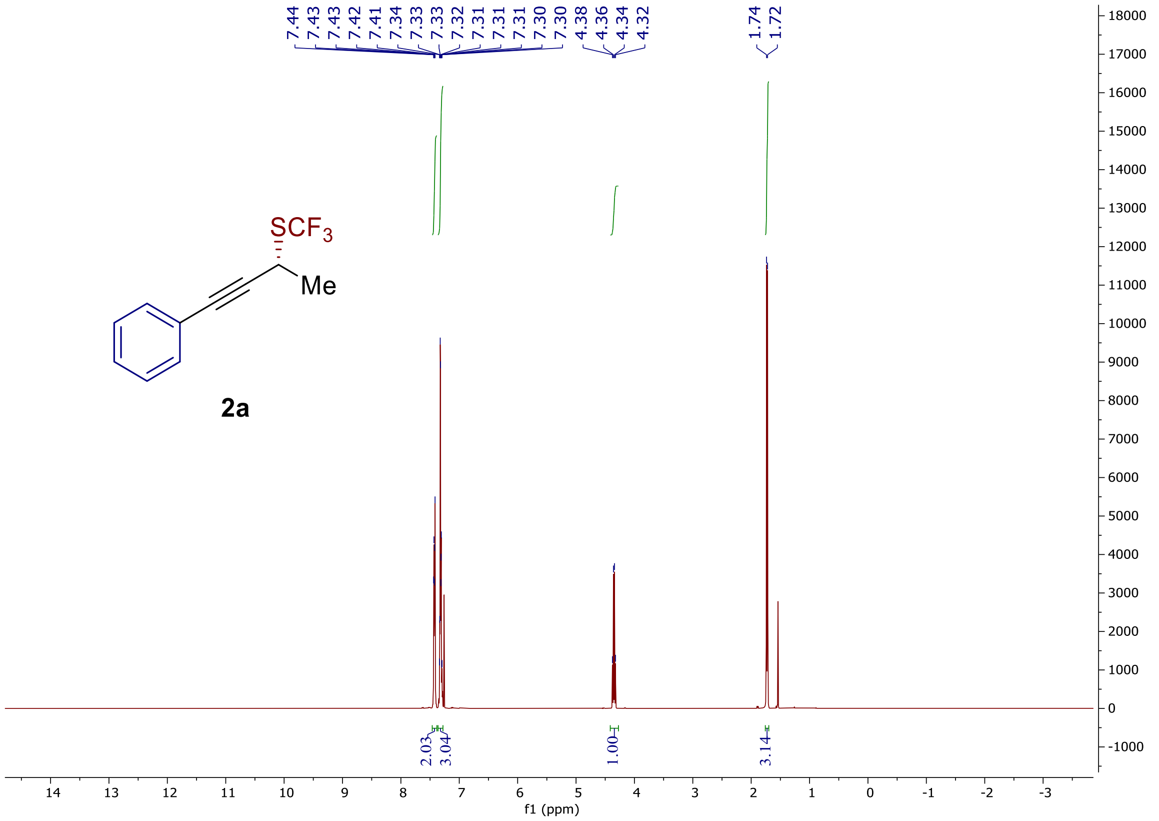

^{19}F NMR:

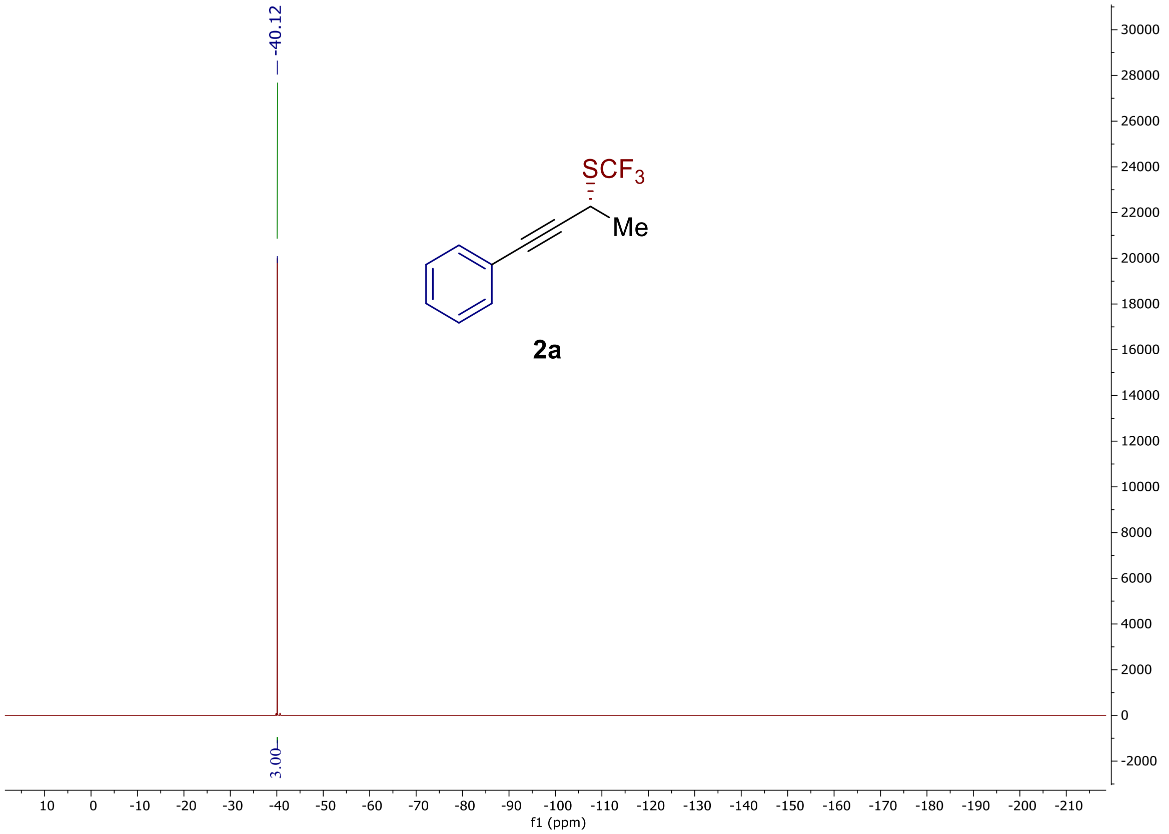

^{13}C NMR:

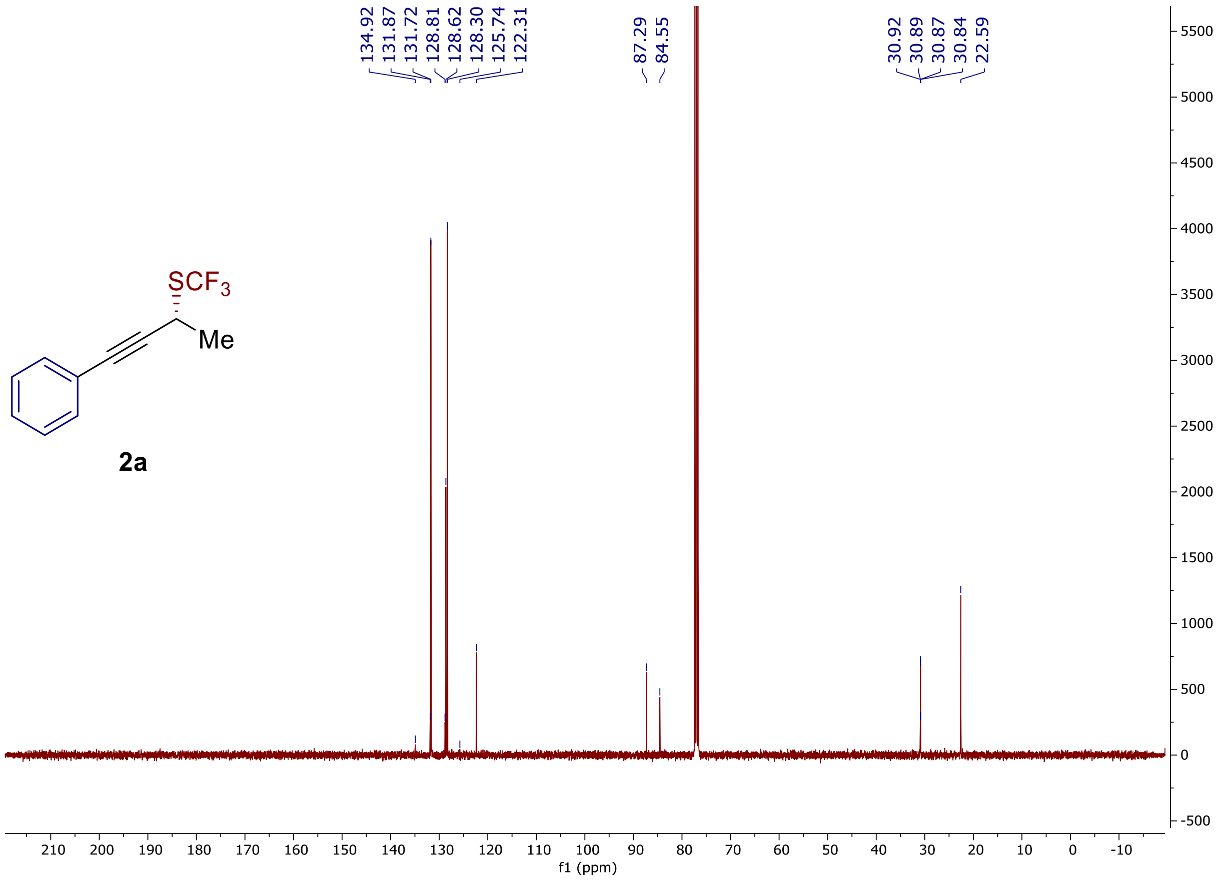
134.92
131.87
131.72
128.81
128.62
128.30
125.74
122.31
87.29
84.55
30.92
30.89
30.87
30.84
22.59
SCF3
Me
2a
f1 (ppm)

^{1}H NMR:

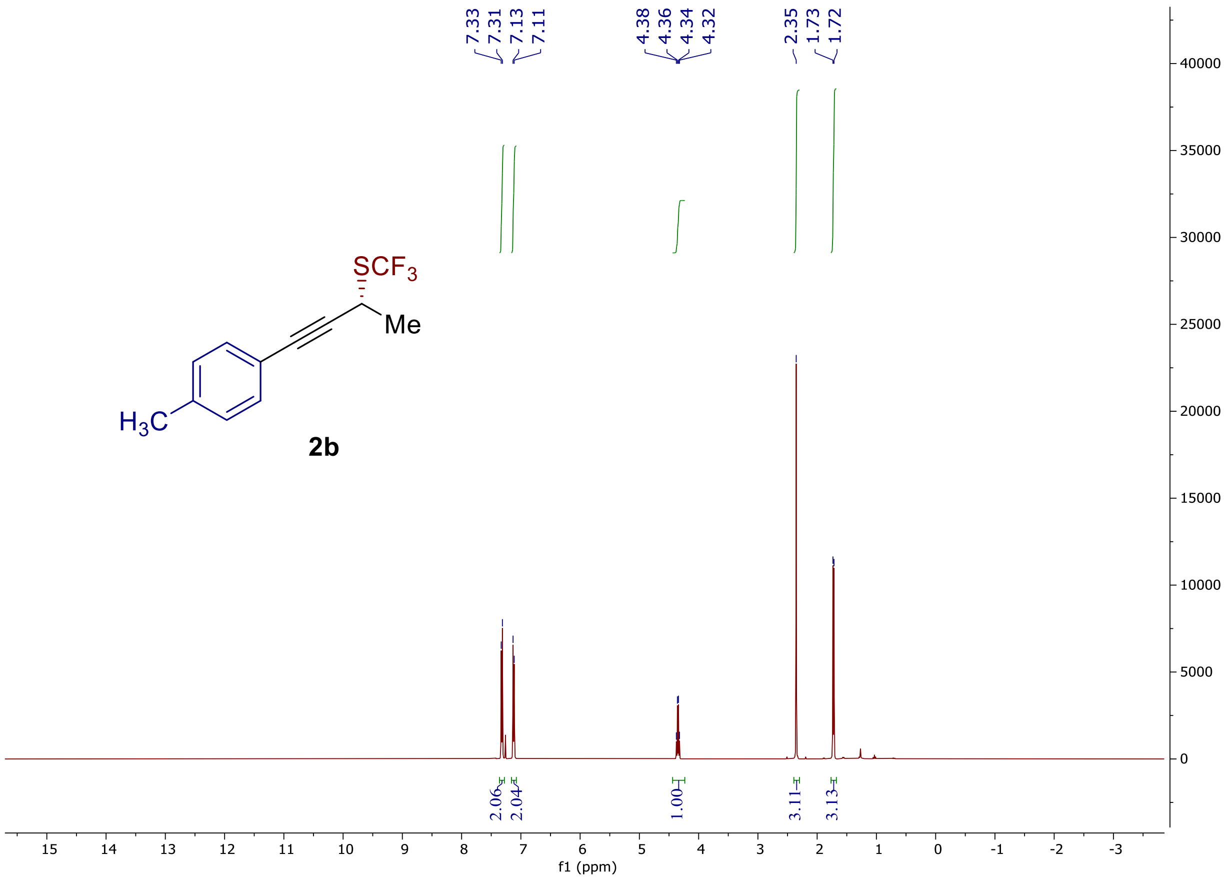

^{19}F NMR:

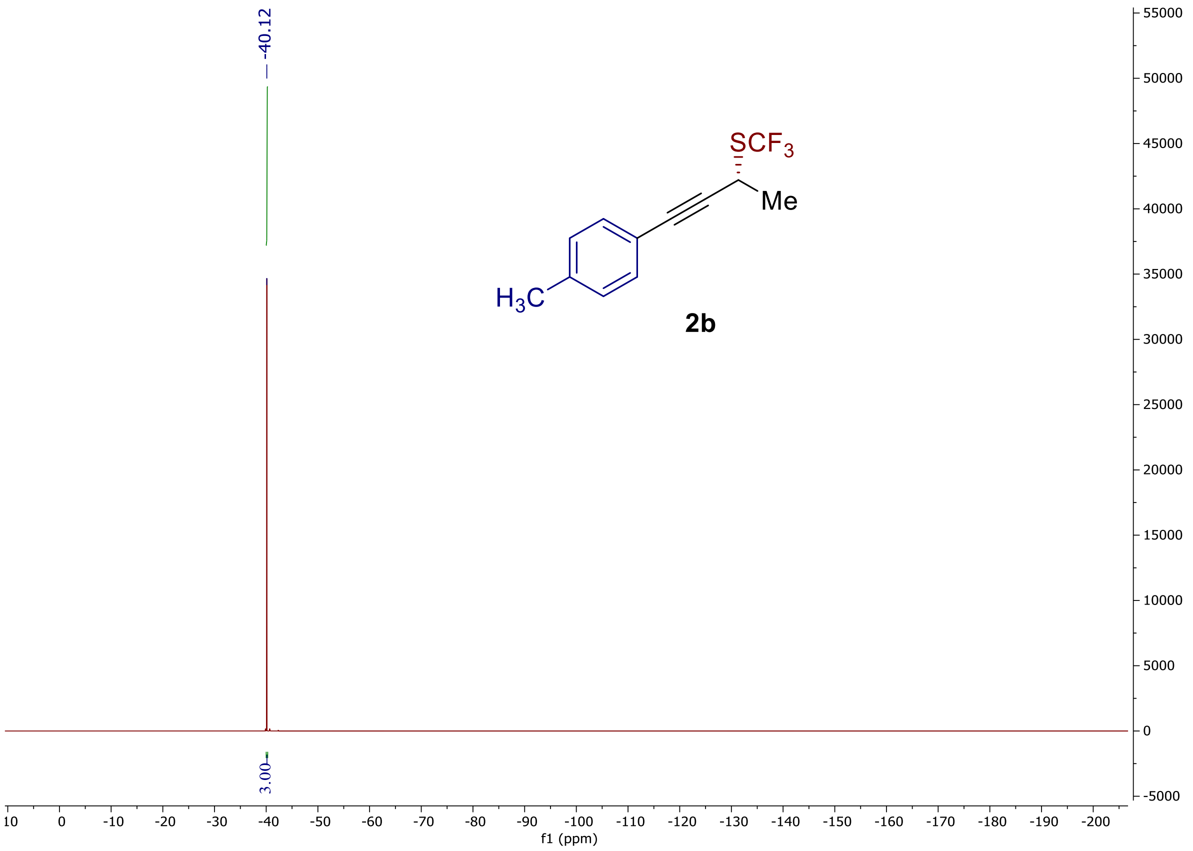

^{13}C NMR:

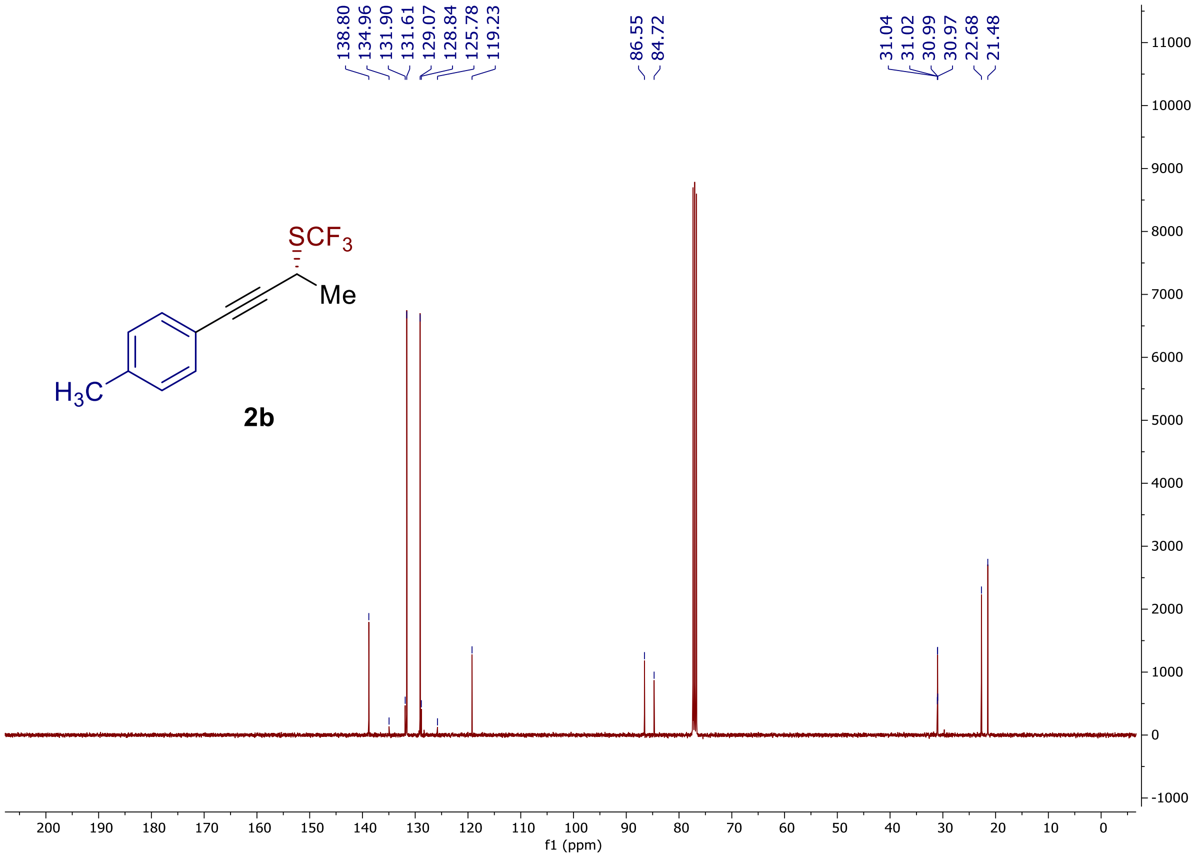
138.80
134.96
131.90
131.61
129.07
128.84
125.78
119.23
86.55
84.72
31.04
31.02
30.99
30.97
22.68
21.48
SCF3
Me
H3C
2b
f1 (ppm)

^{1}H NMR:

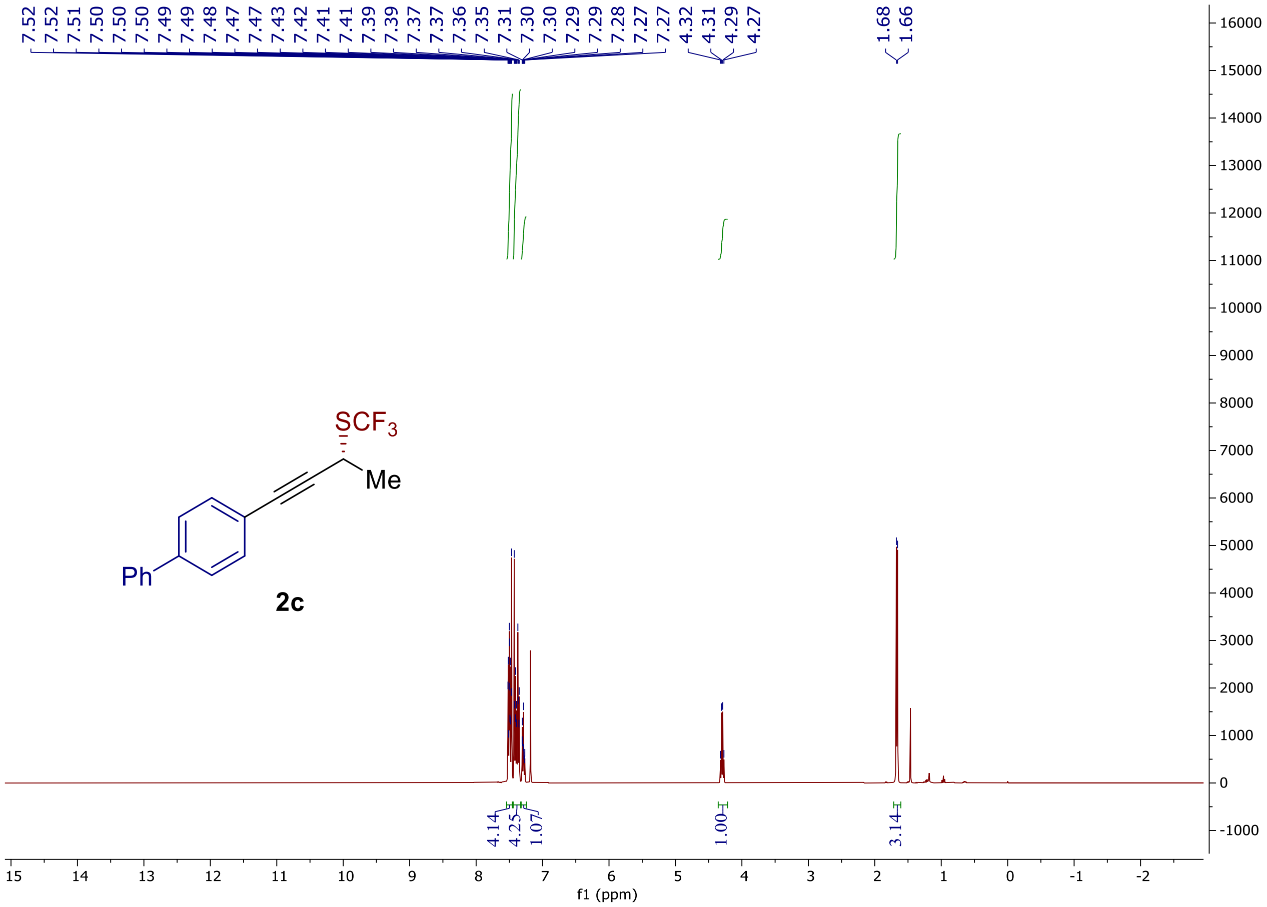

^{19}F NMR:

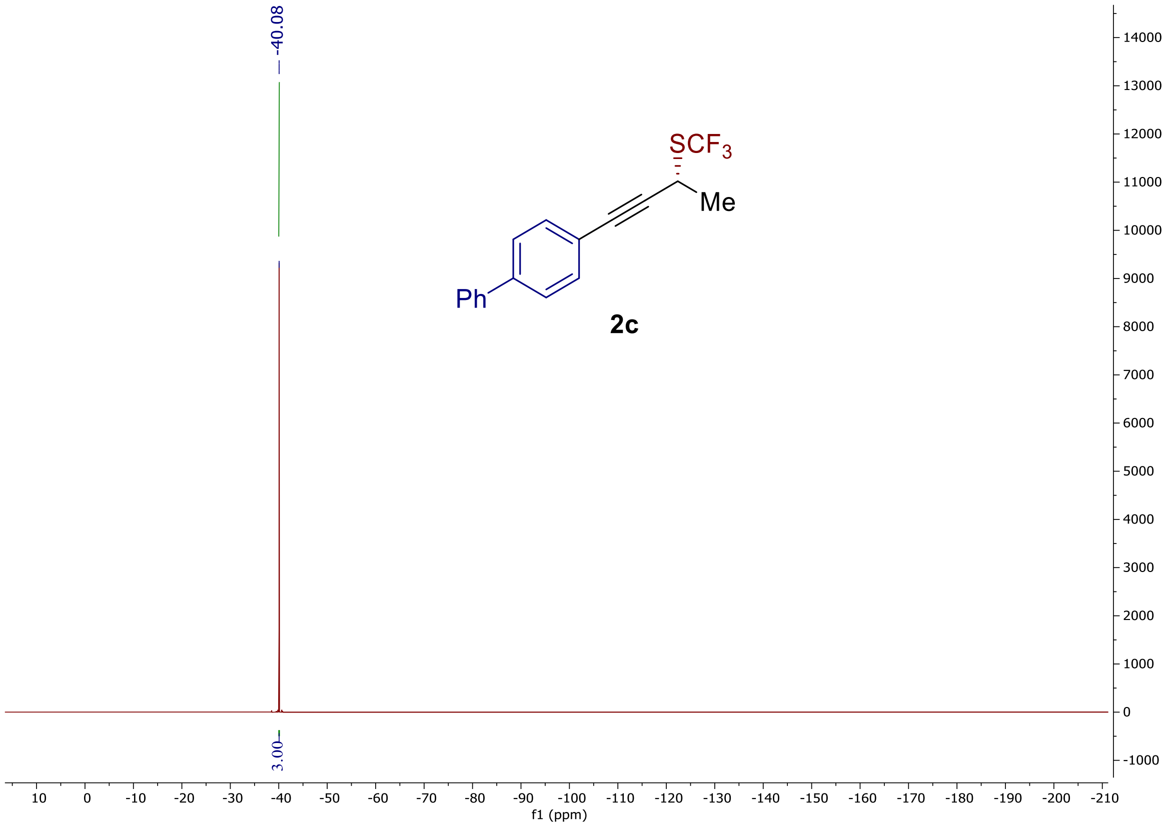

^{13}C NMR:

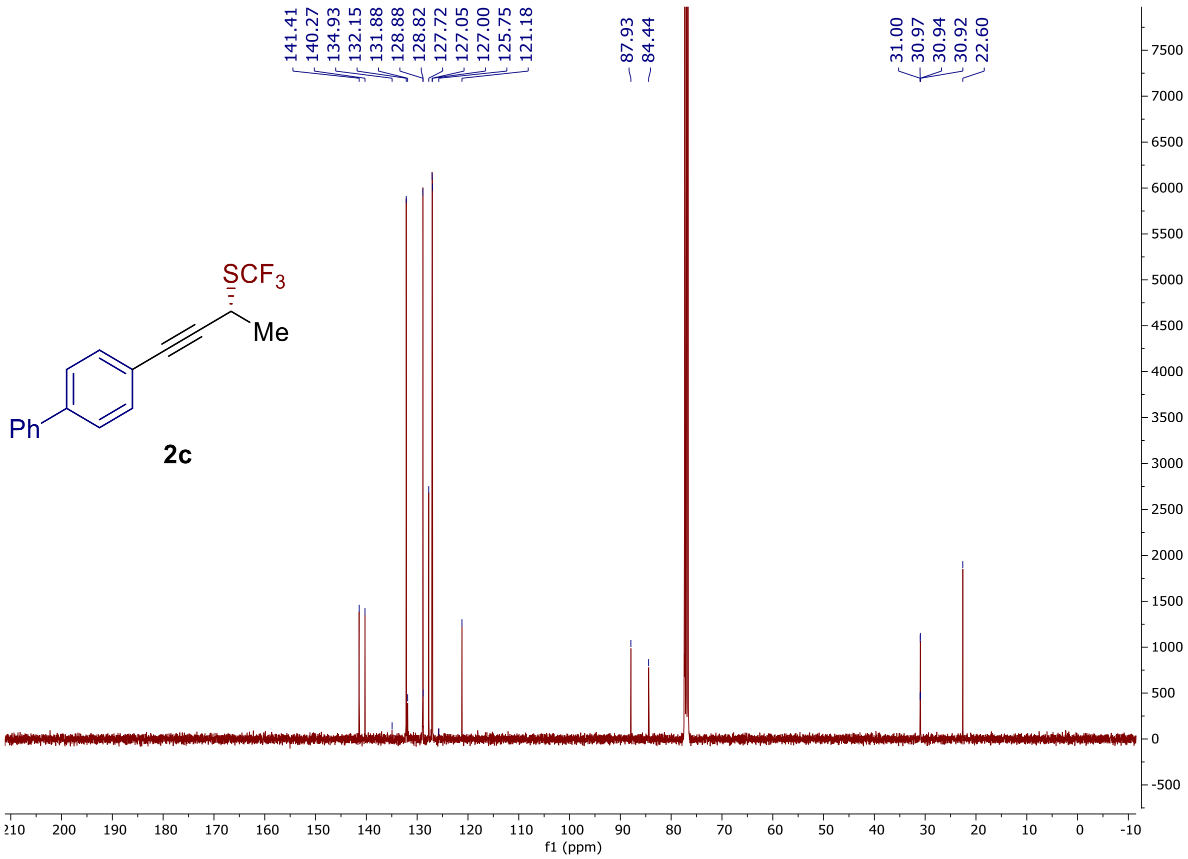

141.41
140.27
134.93
132.15
131.88
128.88
128.82
127.72
127.05
127.00
125.75
121.18
87.93
84.44
31.00
30.97
30.94
30.92
22.60
SCF3
Me
Ph
2c
f1 (ppm)

^{1}H NMR:

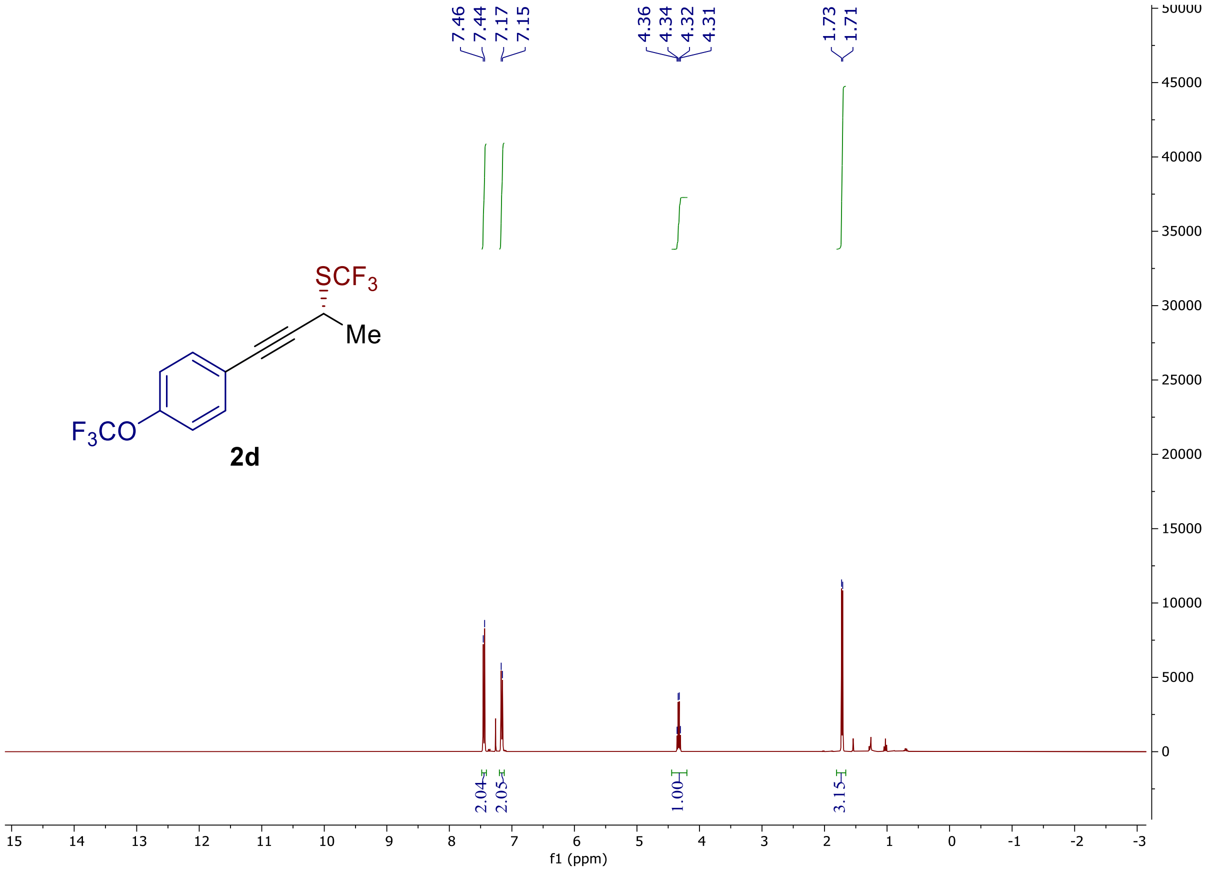

^{19}F NMR:

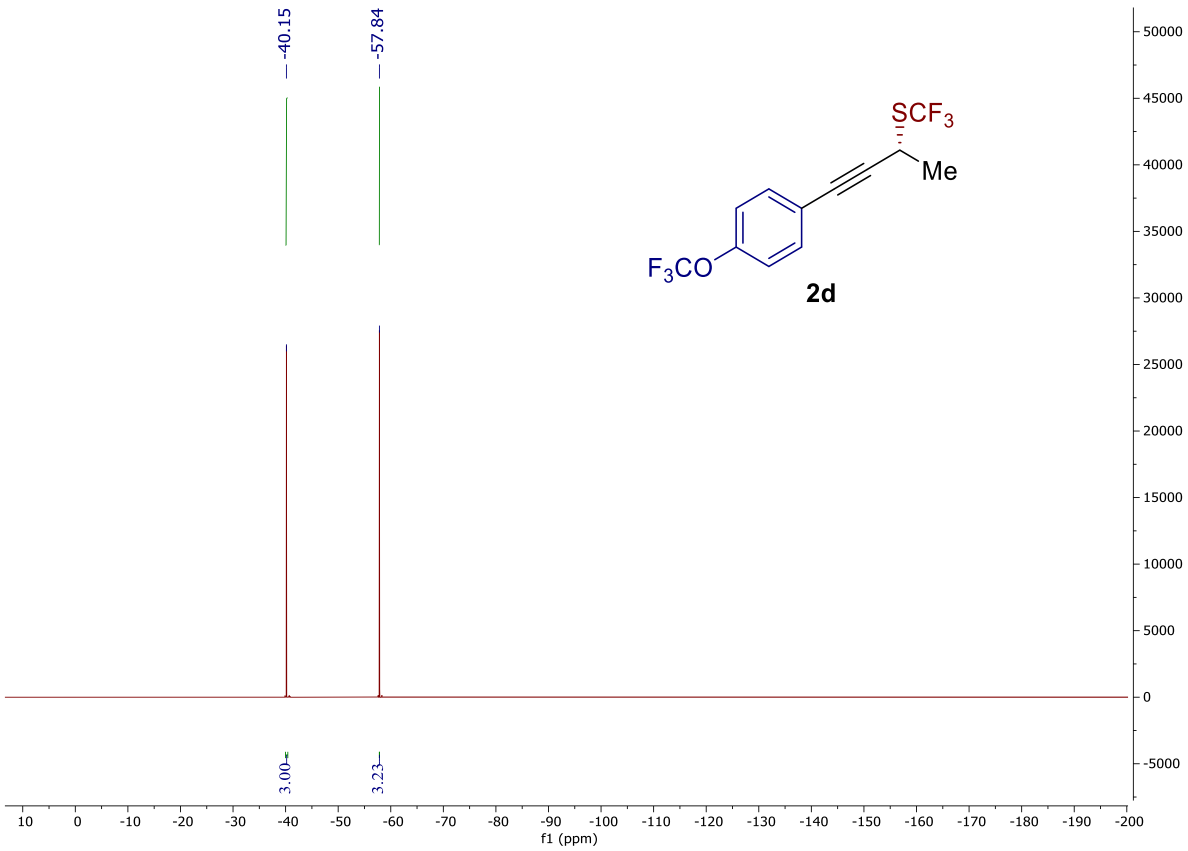

^{13}C NMR:

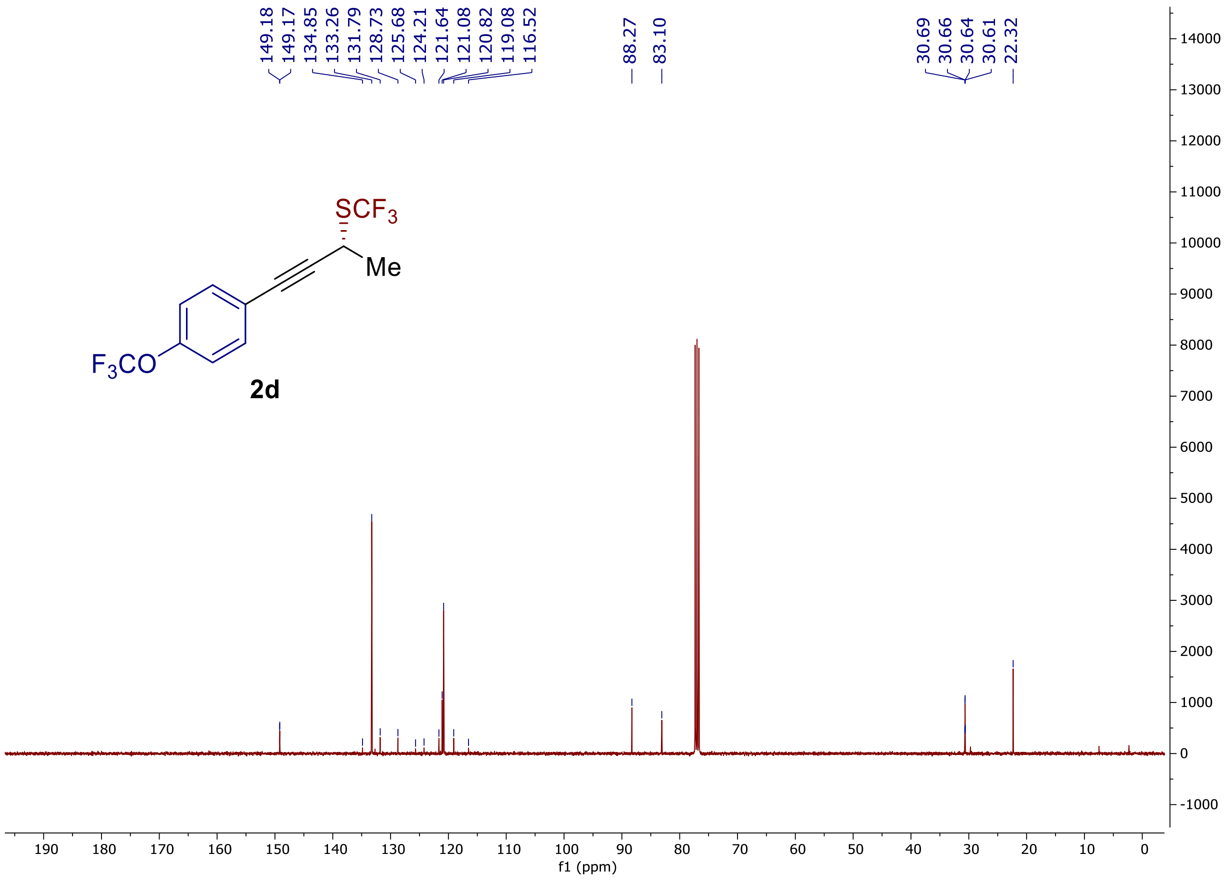
149.18
149.17
134.85
133.26
131.79
128.73
125.68
124.21
121.64
121.08
120.82
119.08
116.52
88.27
83.10
30.69
30.66
30.64
30.61
22.32
SCF3
Me
F3CO
2d
f1 (ppm)

^{1}H NMR:

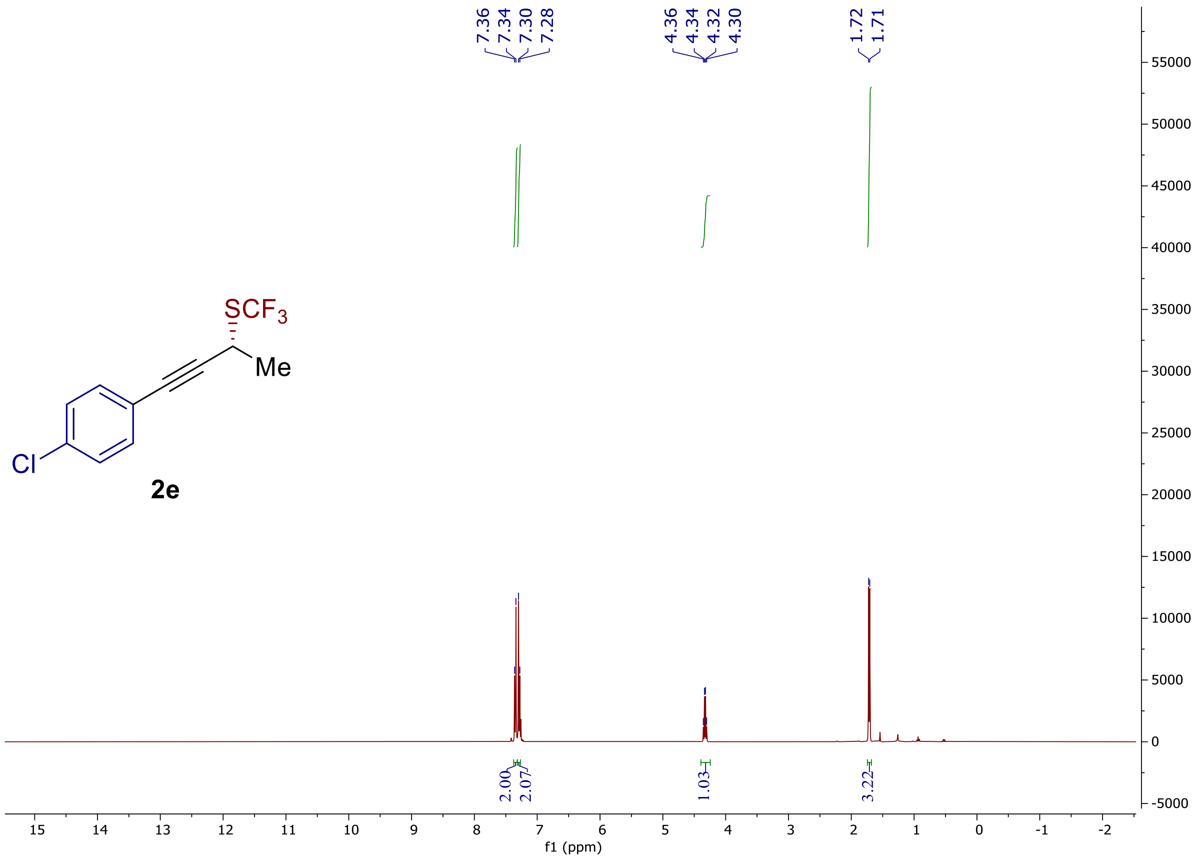

^{19}F NMR:

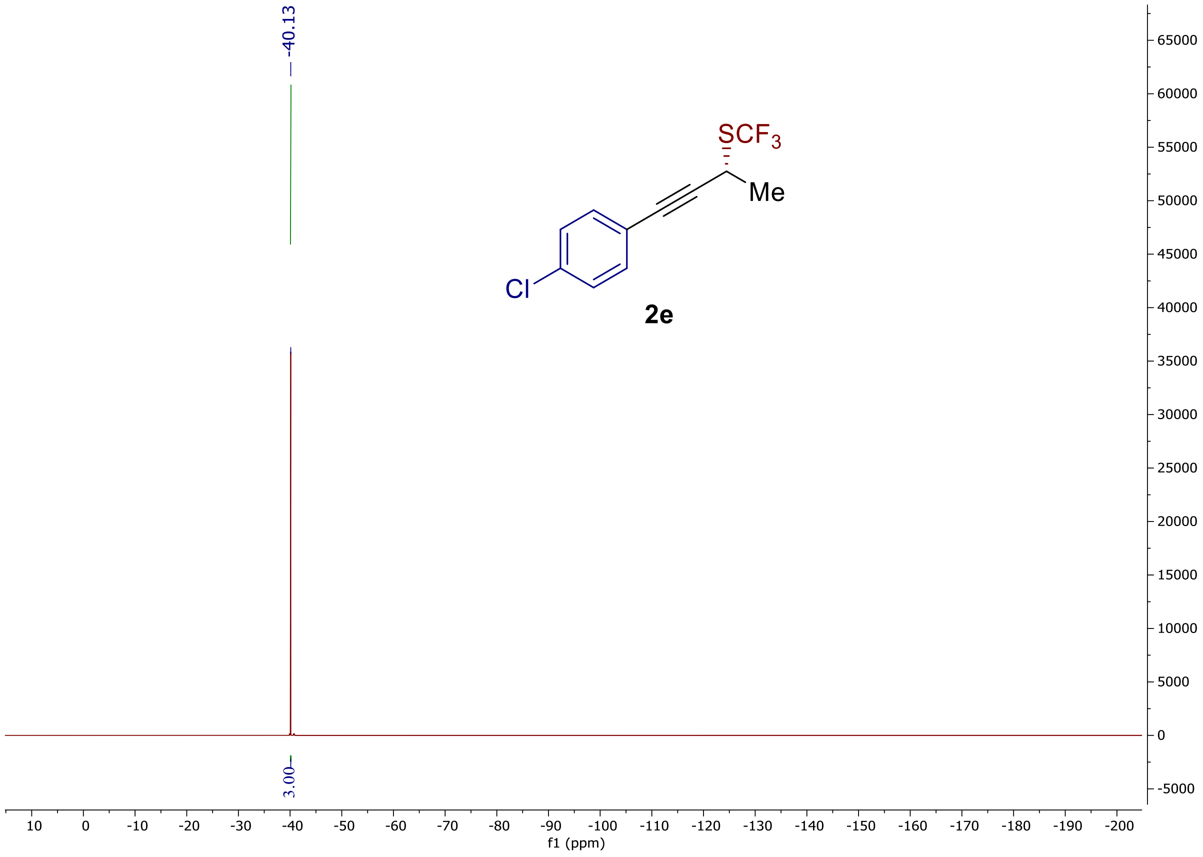

^{13}C NMR:

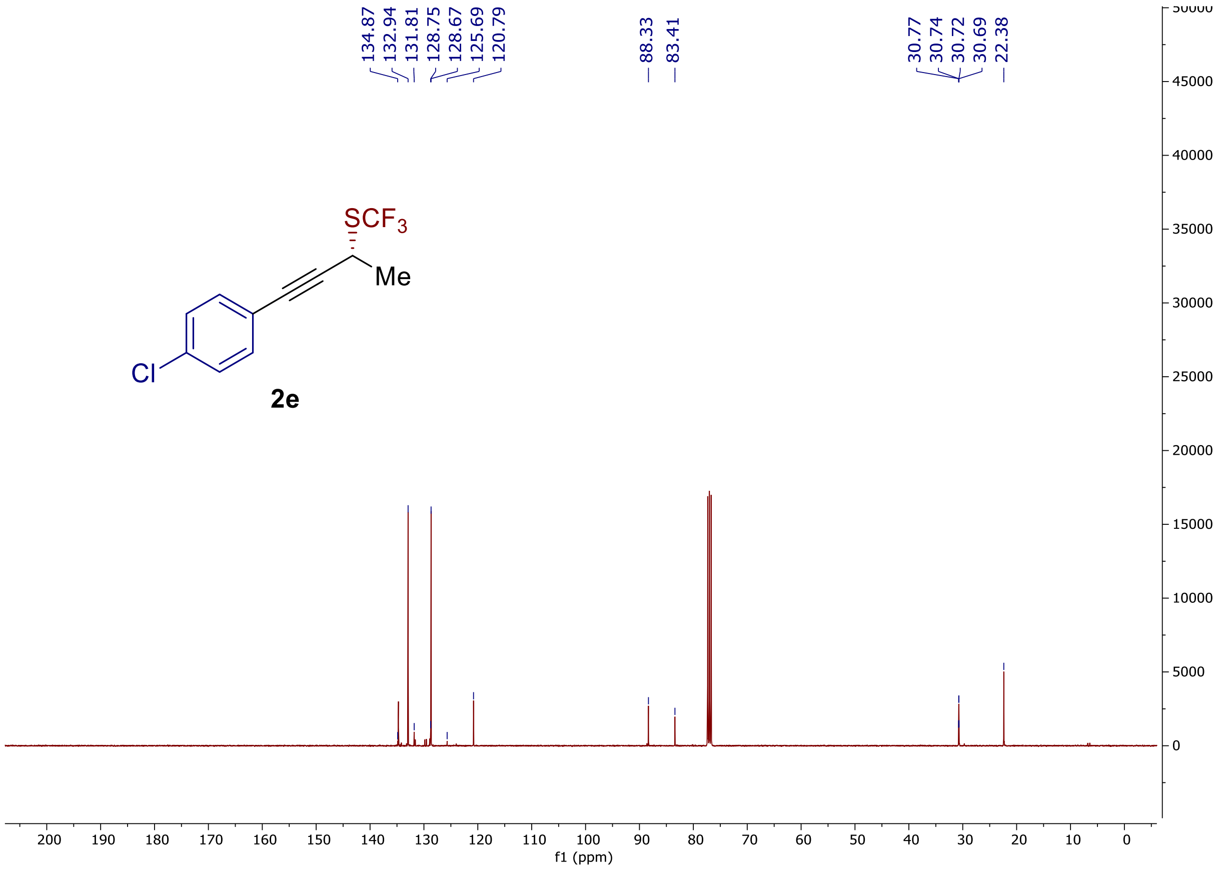

134.87
132.94
131.81
128.75
128.67
125.69
120.79
88.33
83.41
30.77
30.74
30.72
30.69
22.38
SCF3
Me
Cl
2e
f1 (ppm)

^{1}H NMR:

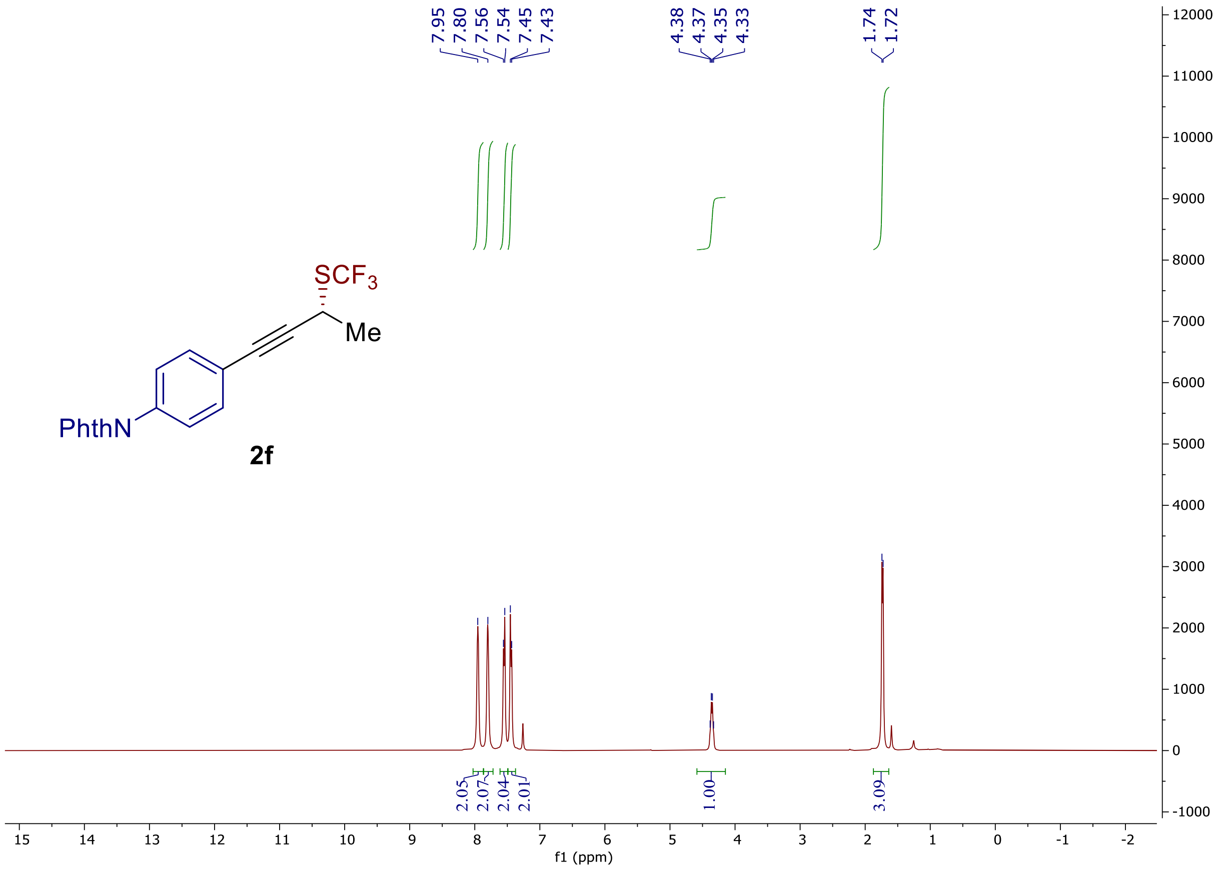

^{19}F NMR:

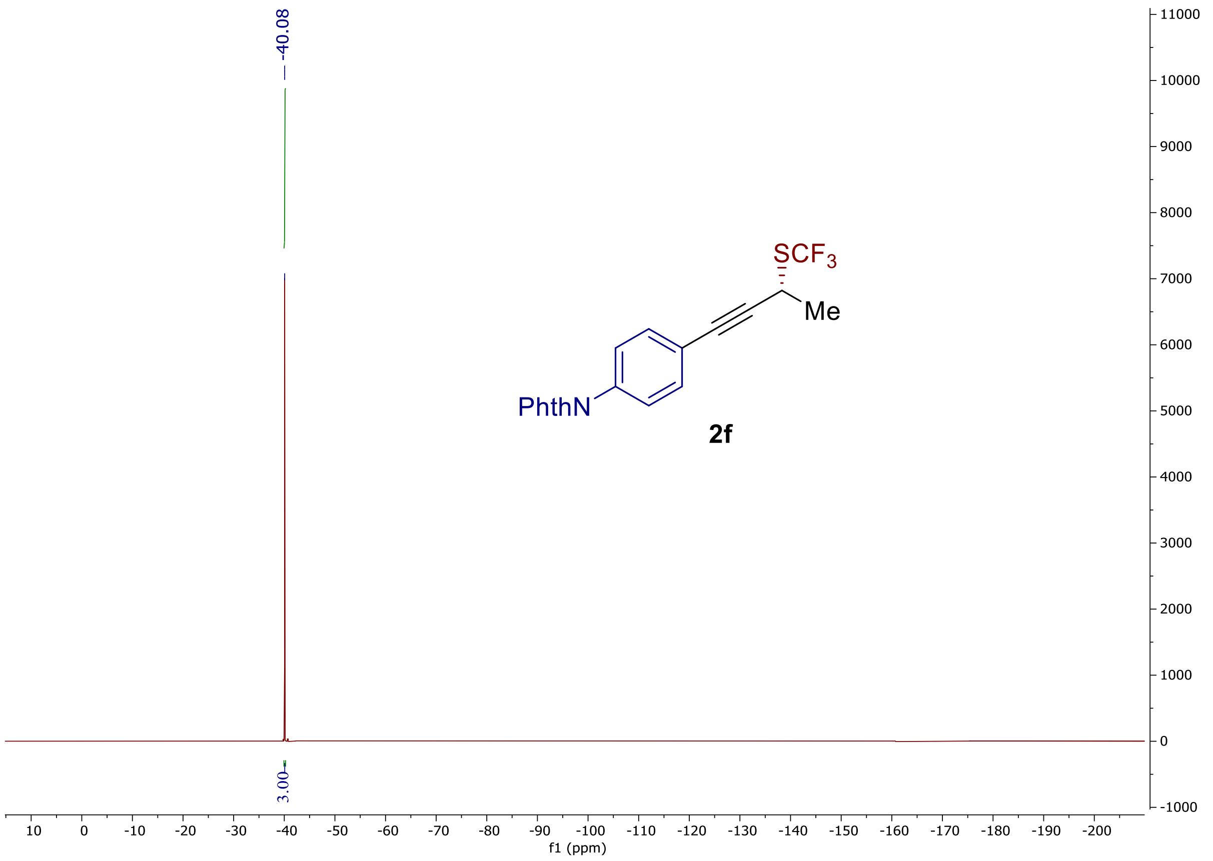

^{13}C NMR:

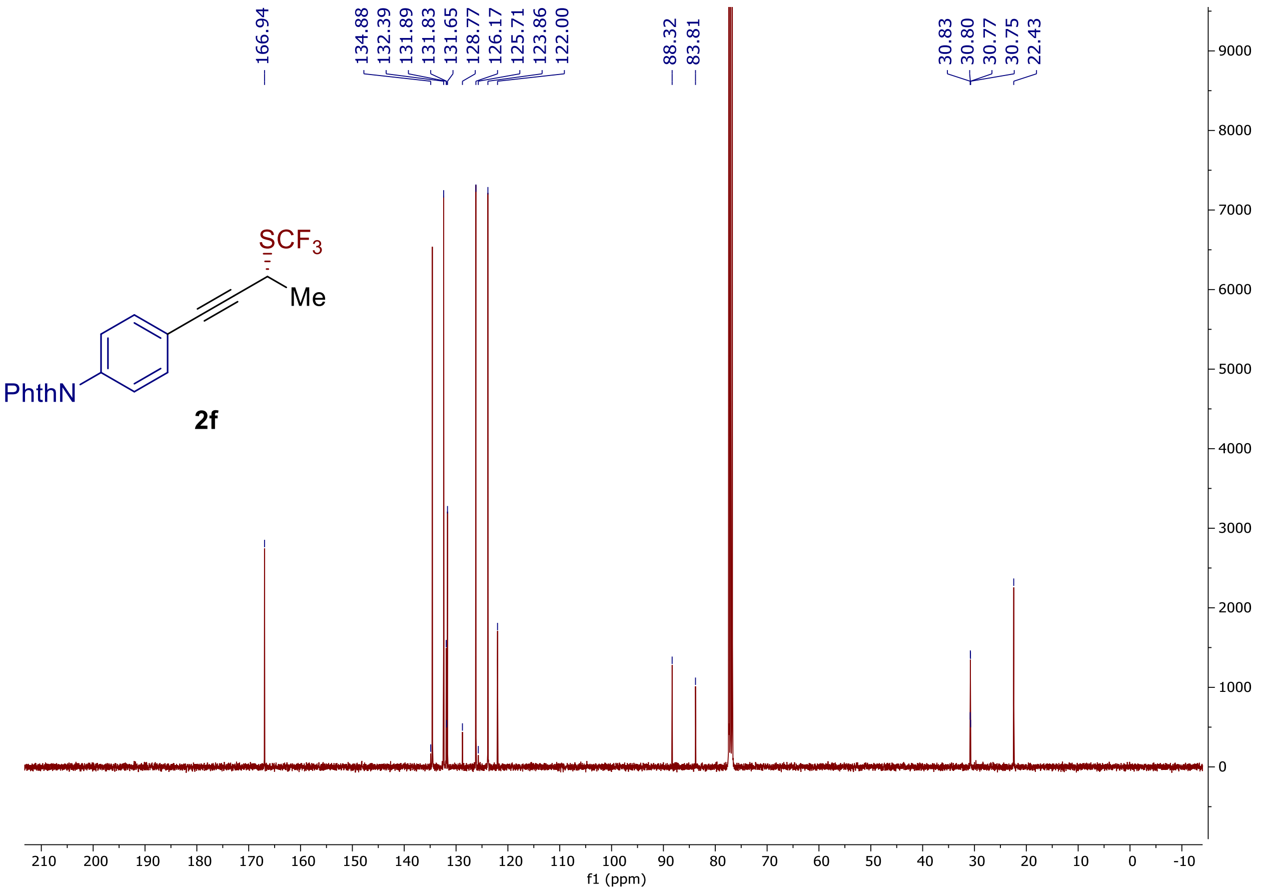

166.94
134.88
132.39
131.89
131.83
131.65
128.77
126.17
125.71
123.86
122.00
88.32
83.81
30.83
30.80
30.77
30.75
22.43
SCF3
Me
PhthN
2f
f1 (ppm)

^{1}H NMR:

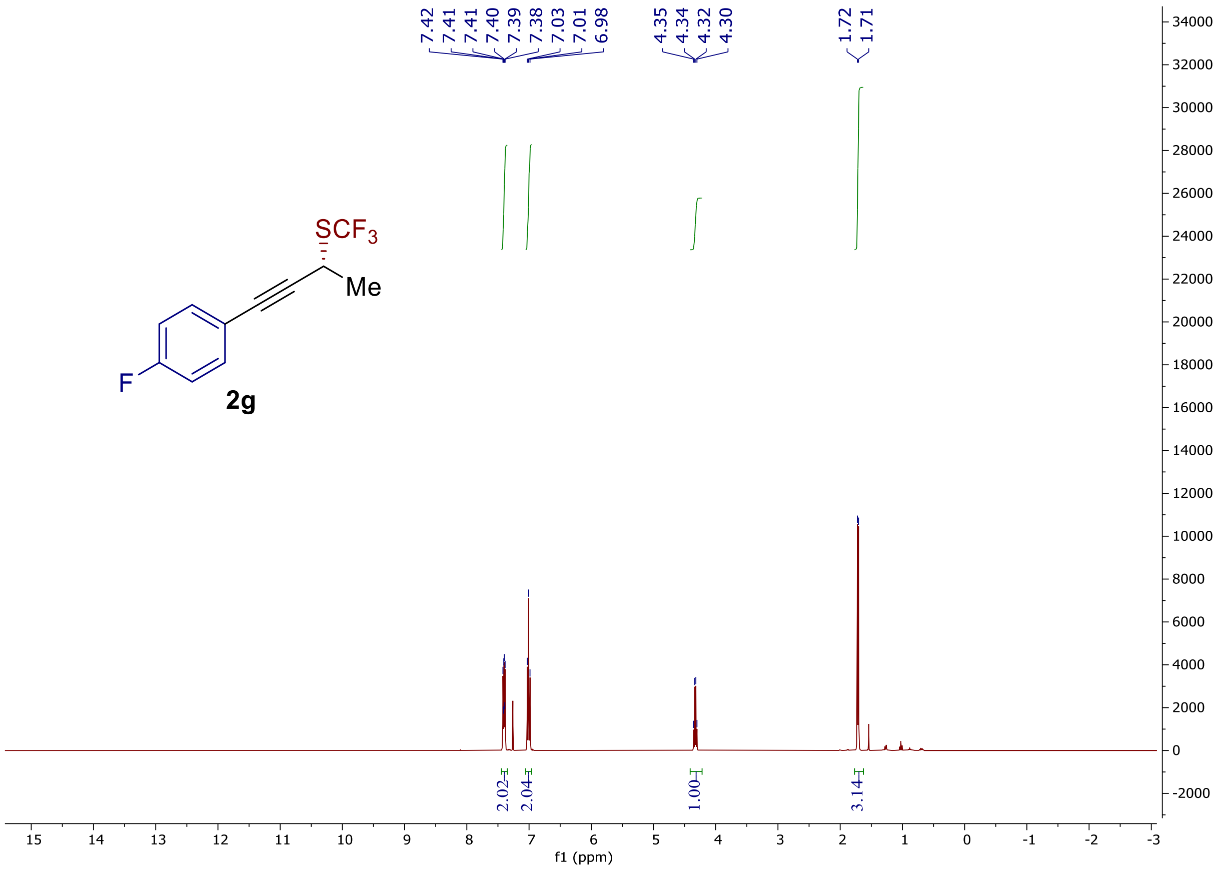

^{19}F NMR:

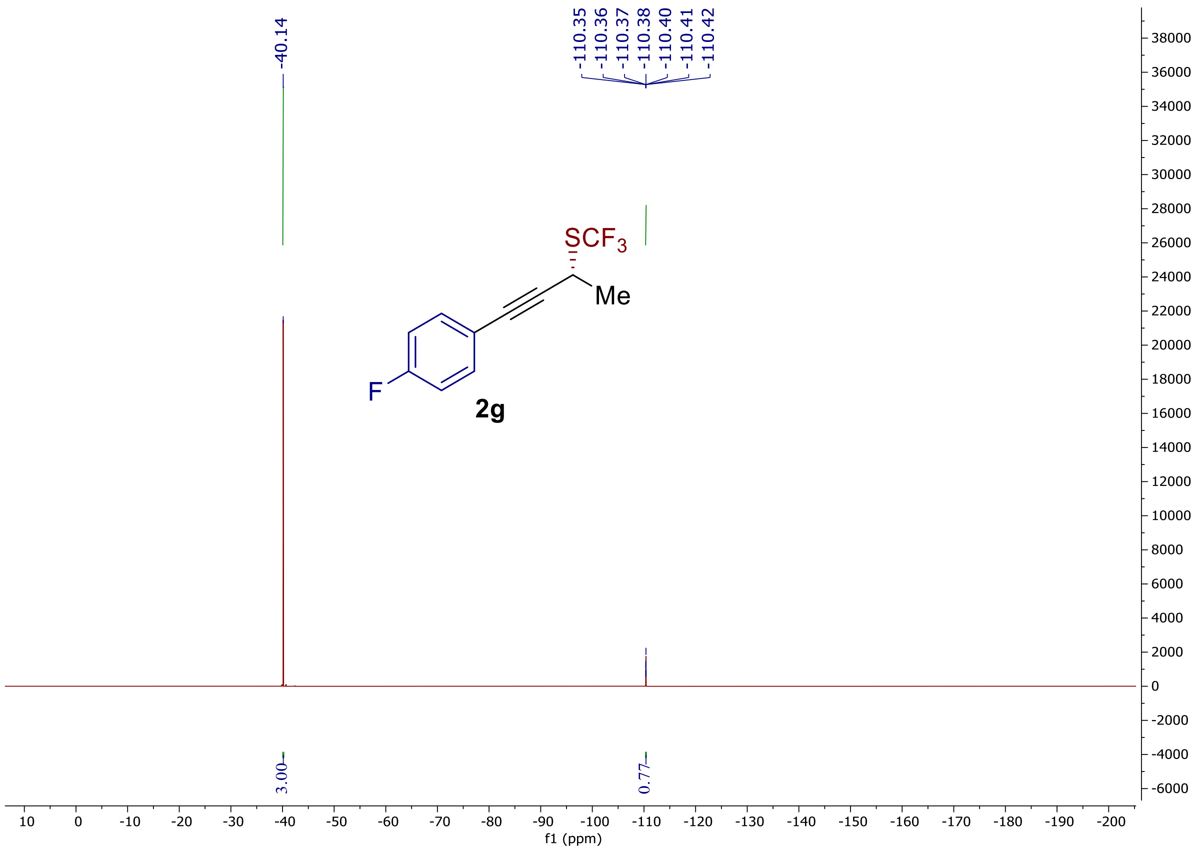

^{13}C NMR:

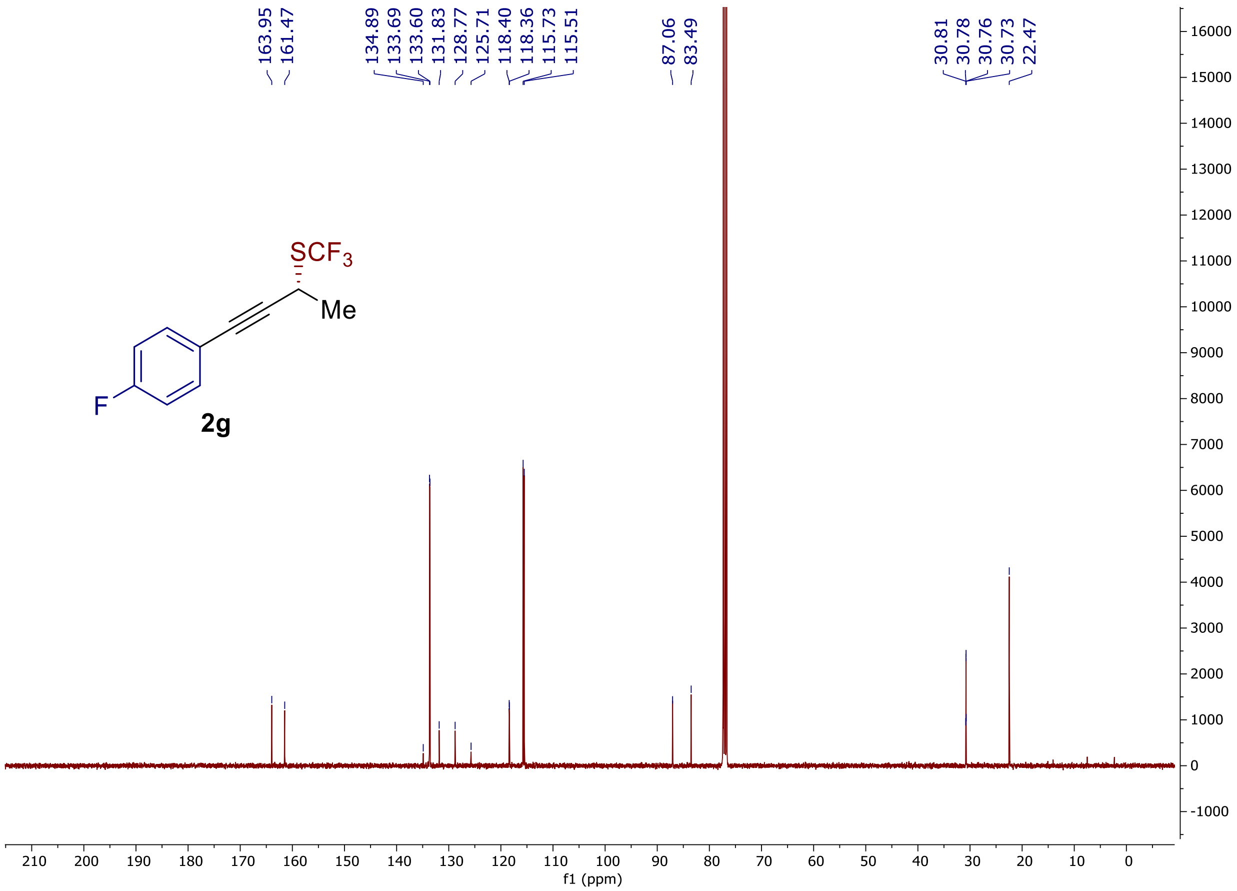

163.95
161.47
134.89
133.69
133.60
131.83
128.77
125.71
118.40
118.36
115.73
115.51
87.06
83.49
30.81
30.78
30.76
30.73
22.47
SCF3
Me
F
2g
f1 (ppm)

^{1}H NMR:

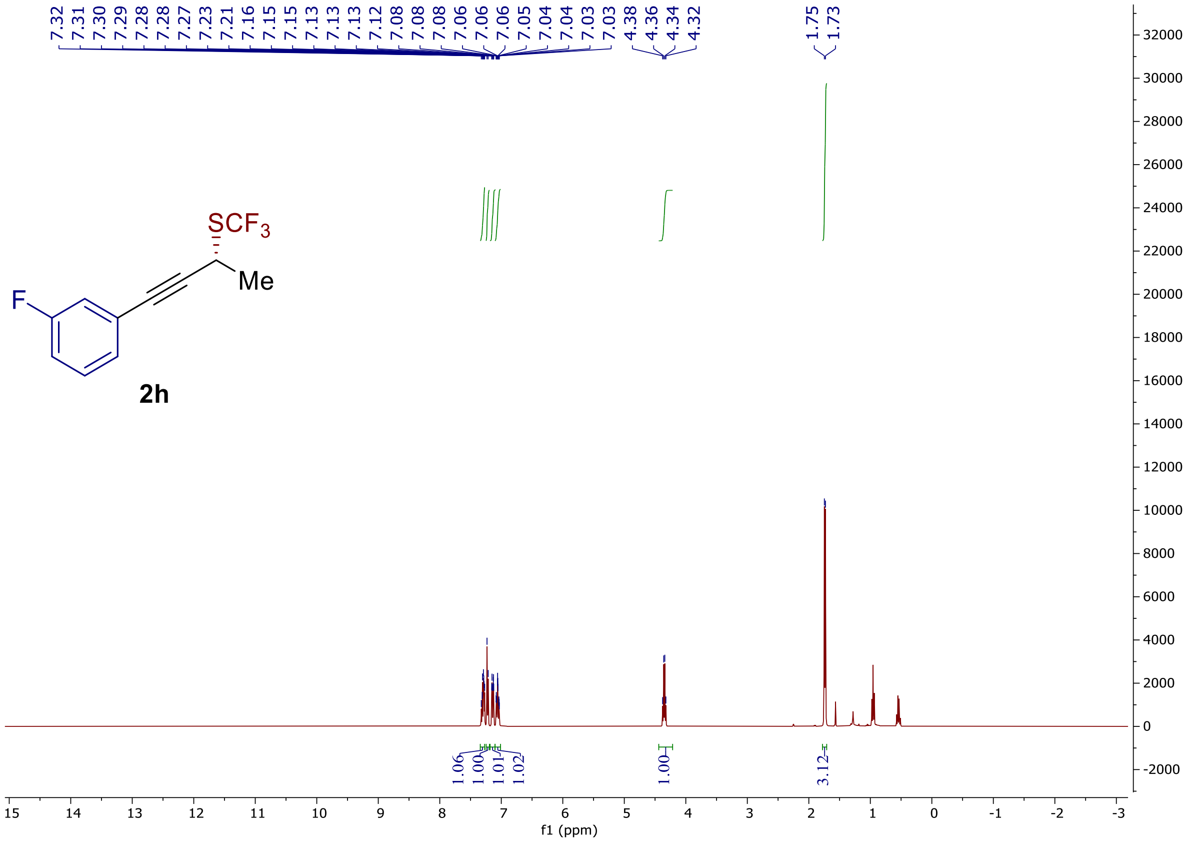

^{19}F NMR:

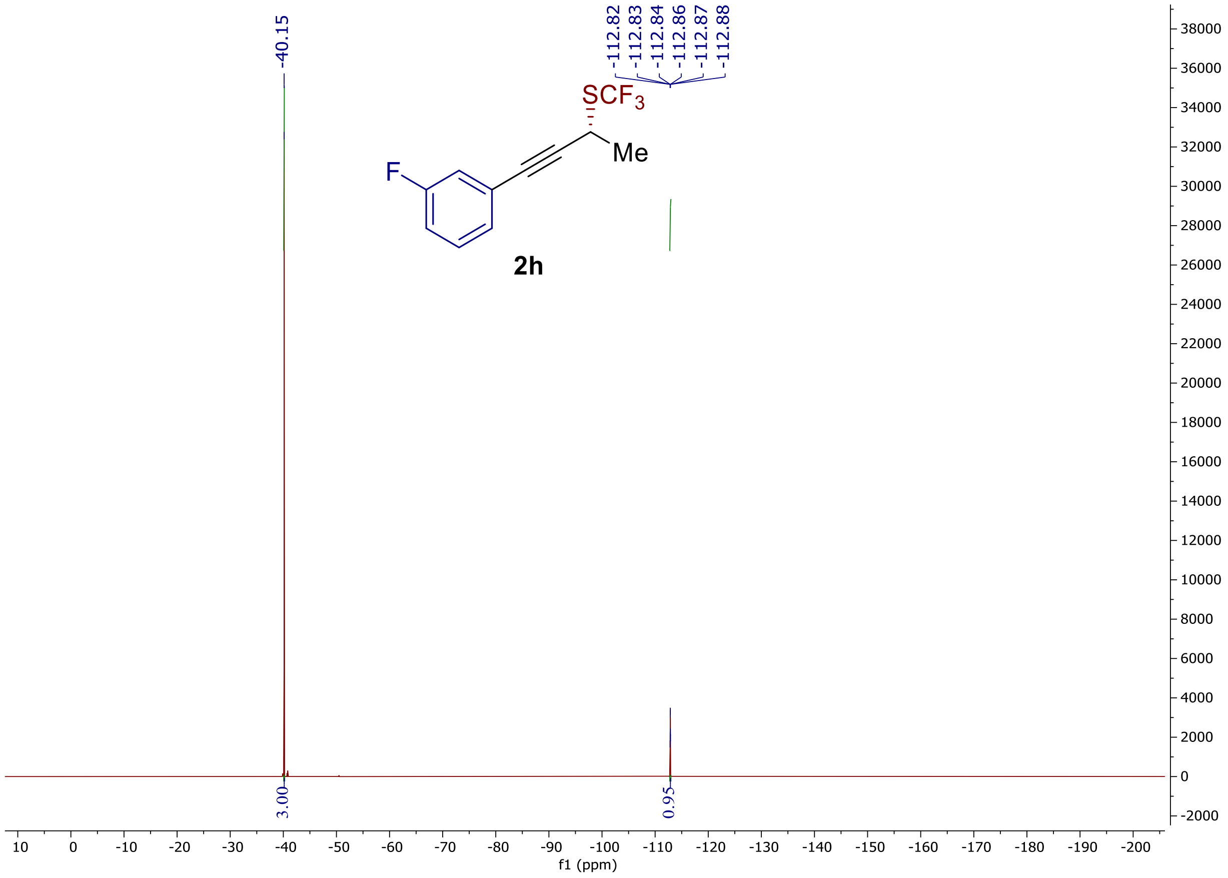

^{13}C NMR:

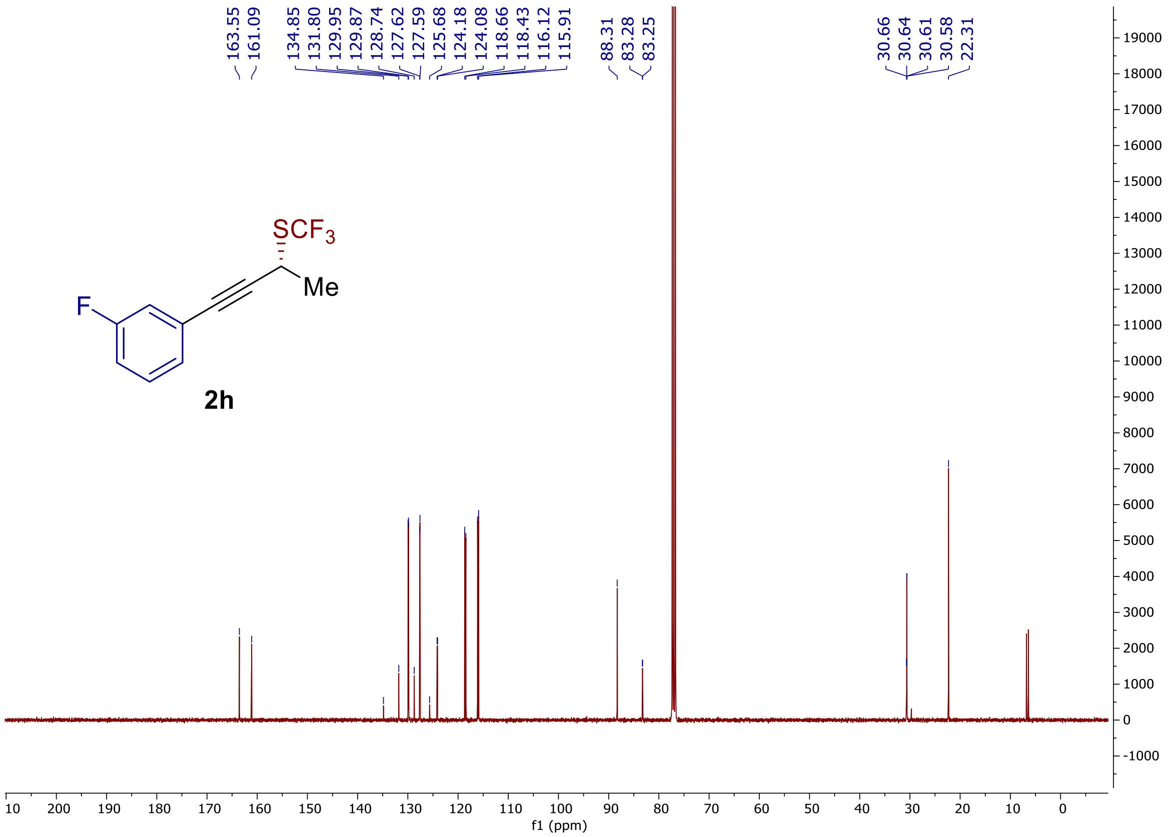

163.55
161.09
134.85
131.80
129.95
129.87
128.74
127.62
127.59
125.68
124.18
124.08
118.66
118.43
116.12
115.91
88.31
83.28
83.25
30.66
30.64
30.61
30.58
22.31
SCF3
Me
F
2h
f1 (ppm)

^{1}H NMR:

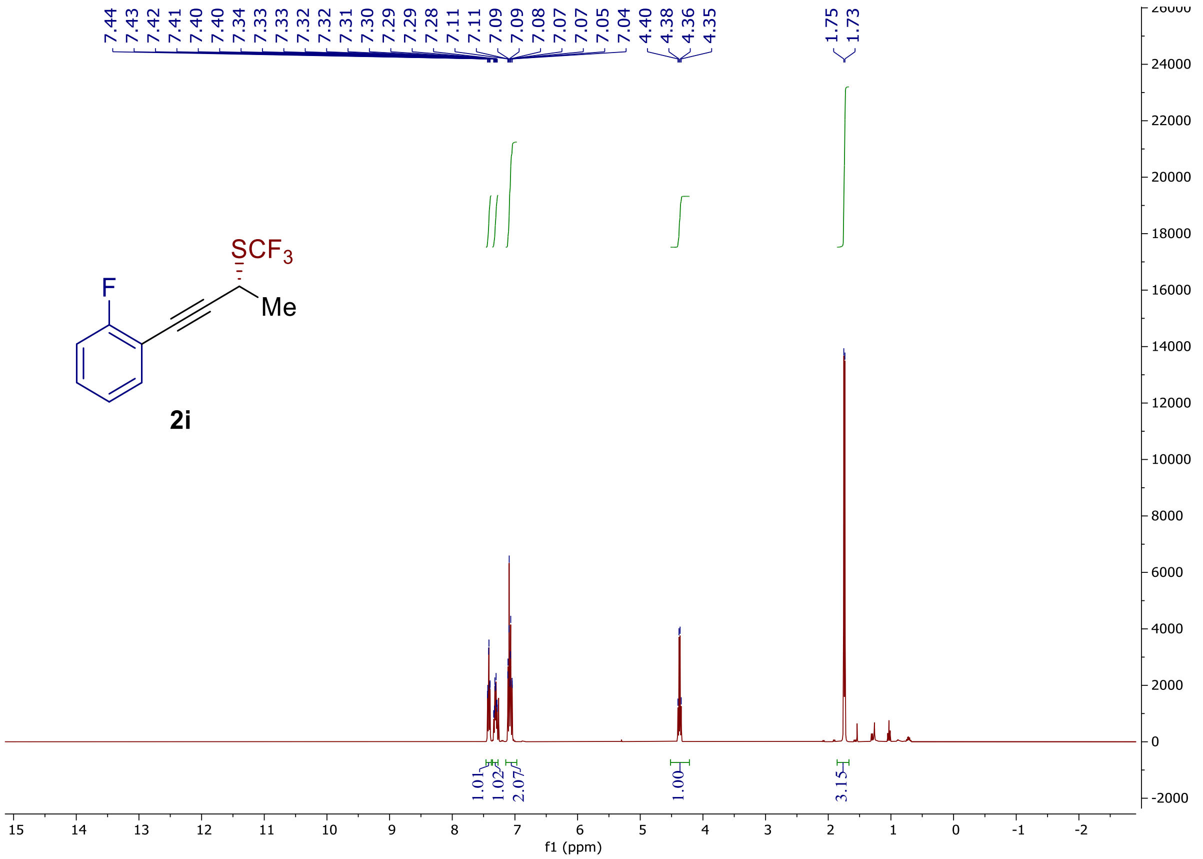

^{19}F NMR:

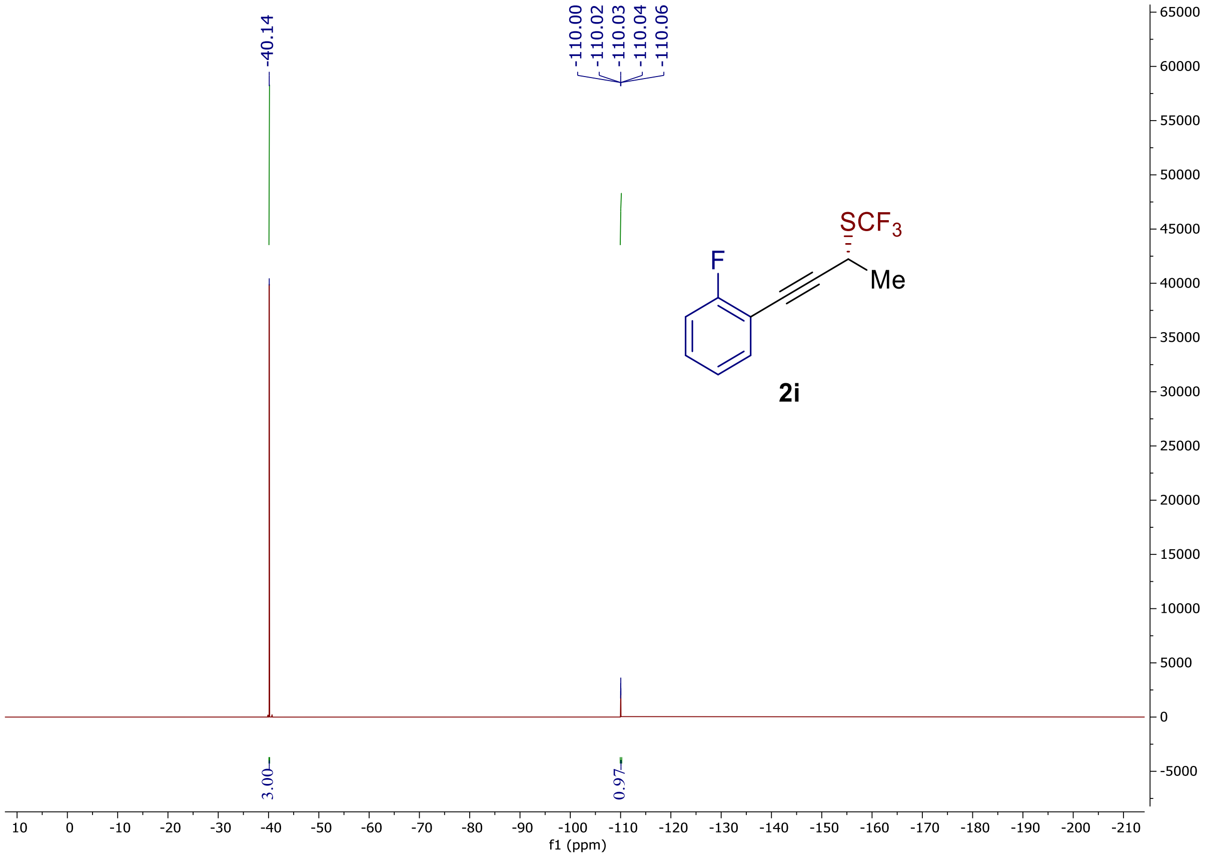

^{13}C NMR:

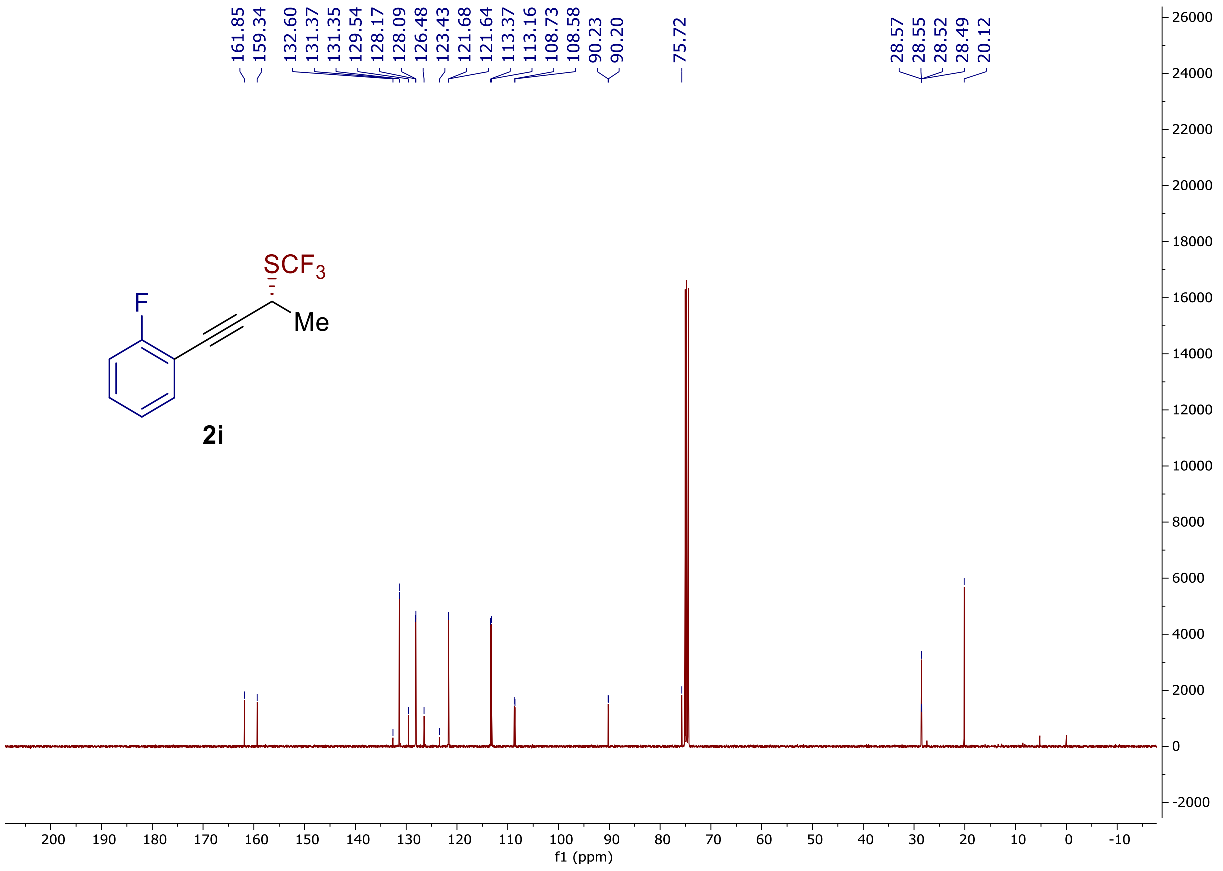

161.85
159.34
132.60
131.37
131.35
129.54
128.17
128.09
126.48
123.43
121.68
121.64
113.37
113.16
108.73
108.58
90.23
90.20
75.72
28.57
28.55
28.52
28.49
20.12
SCF3
F
Me
2i
f1 (ppm)

^{1}H NMR:

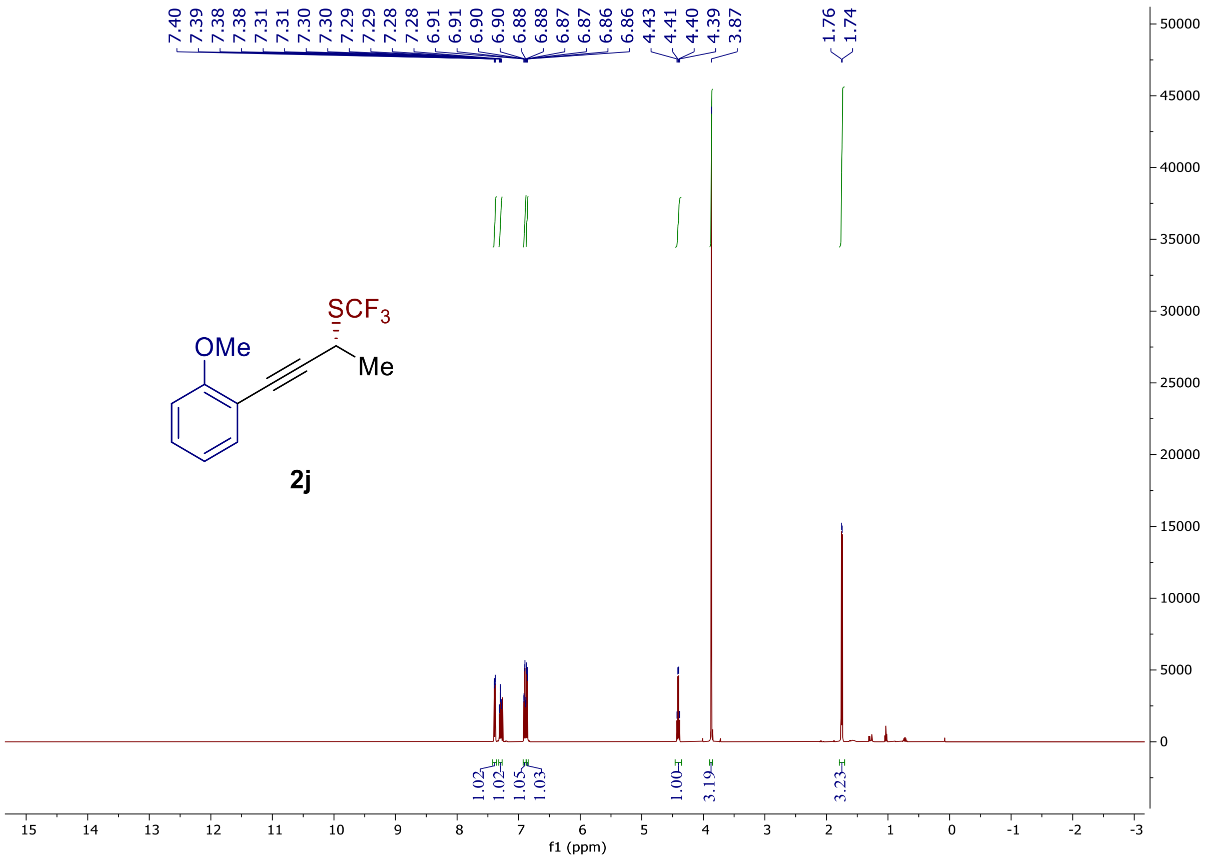

^{19}F NMR:

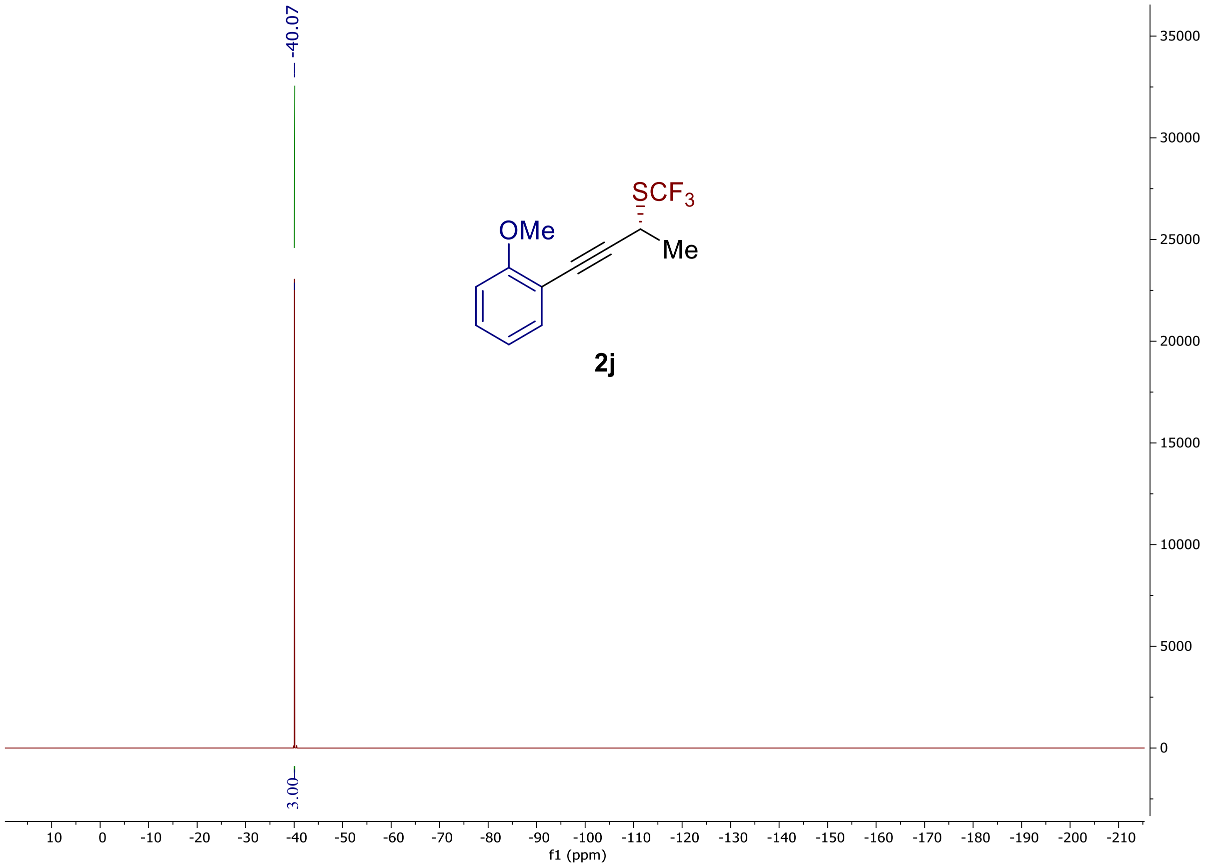

^{13}C NMR:

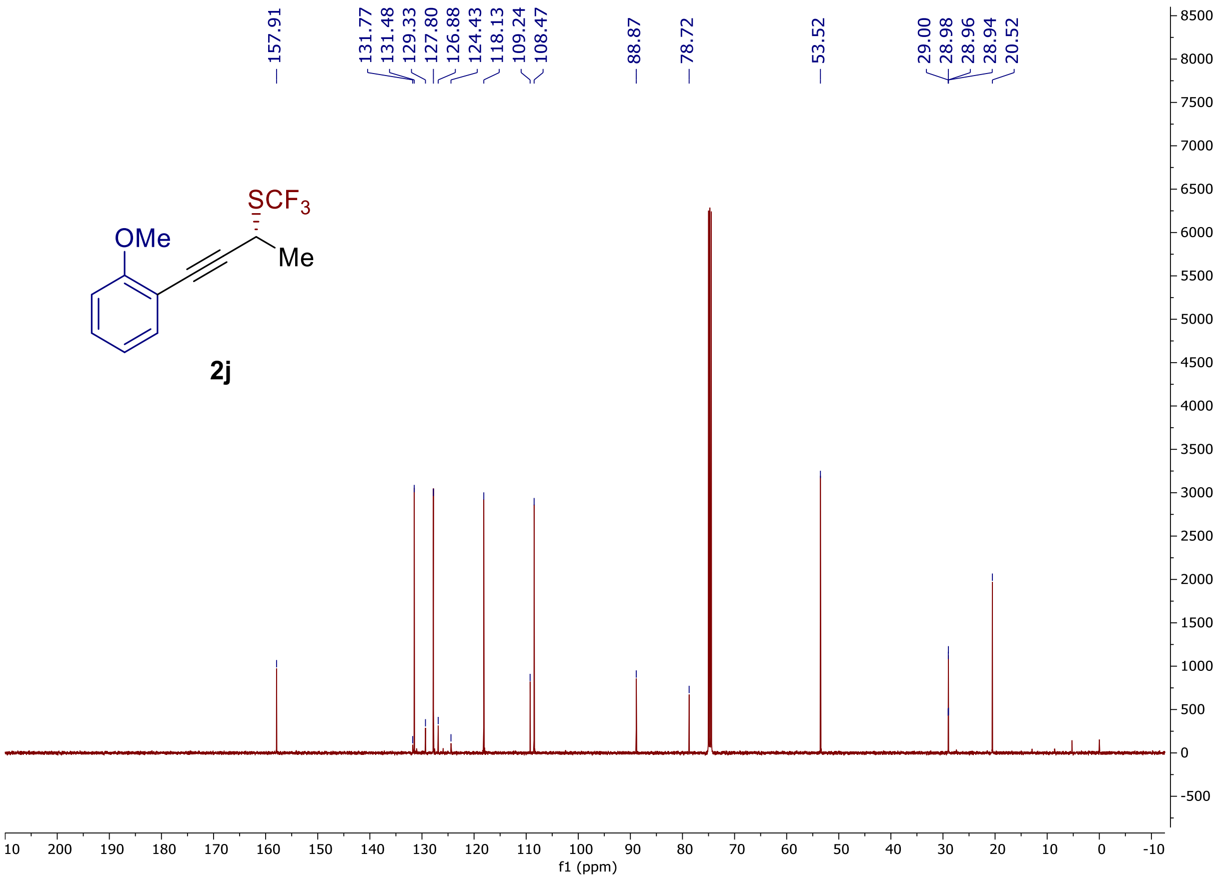

157.91
131.77
131.48
129.33
127.80
126.88
124.43
118.13
109.24
108.47
88.87
78.72
53.52
29.00
28.98
28.96
28.94
20.52
SCF3
OMe
Me
2j
f1 (ppm)

^{1}H NMR:

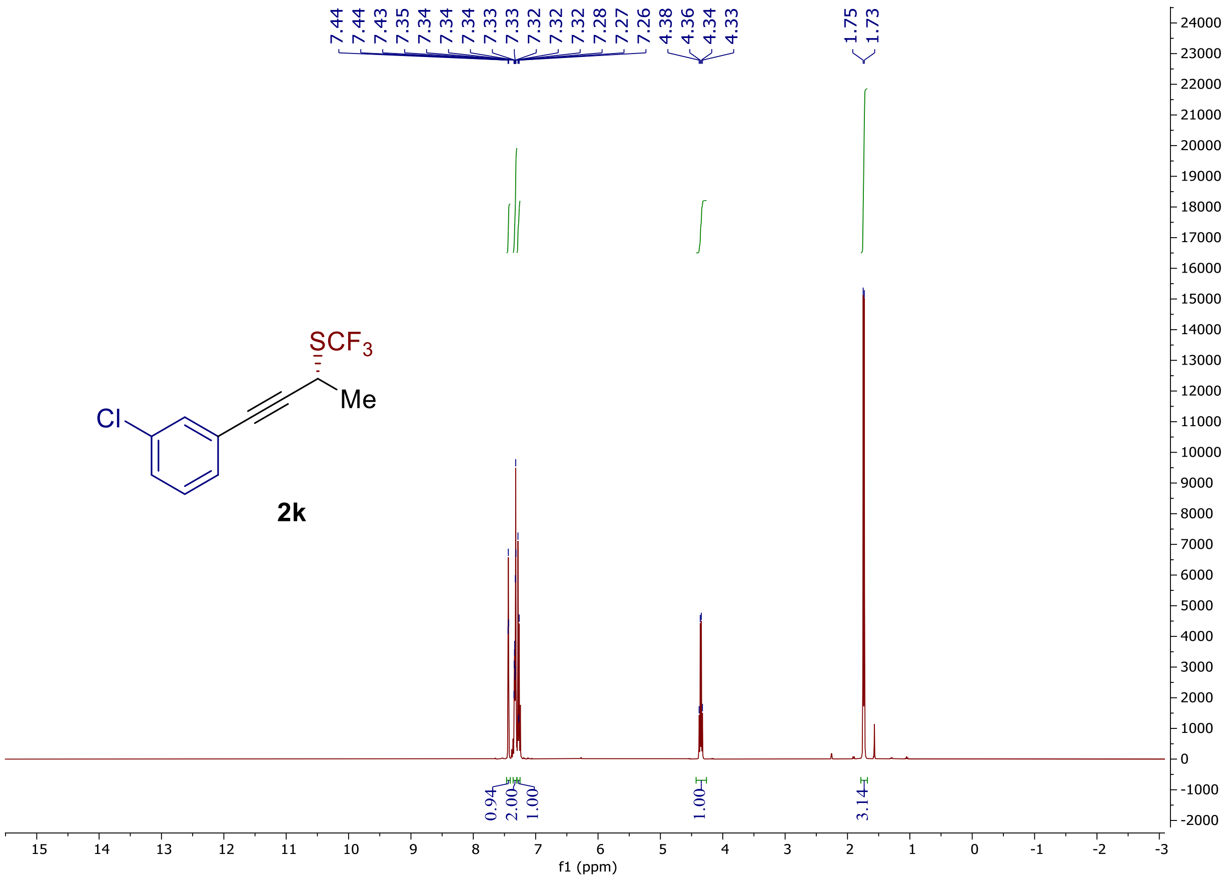

^{19}F NMR:

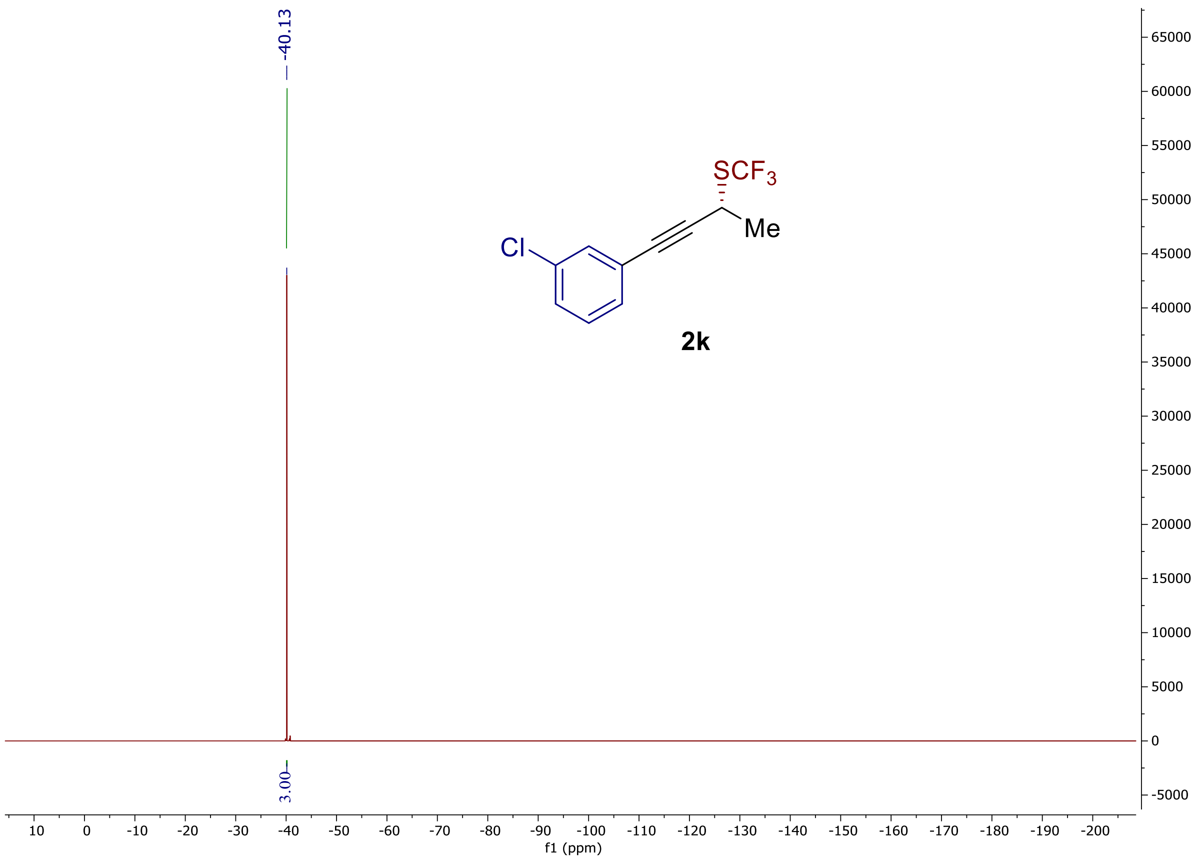

^{13}C NMR:

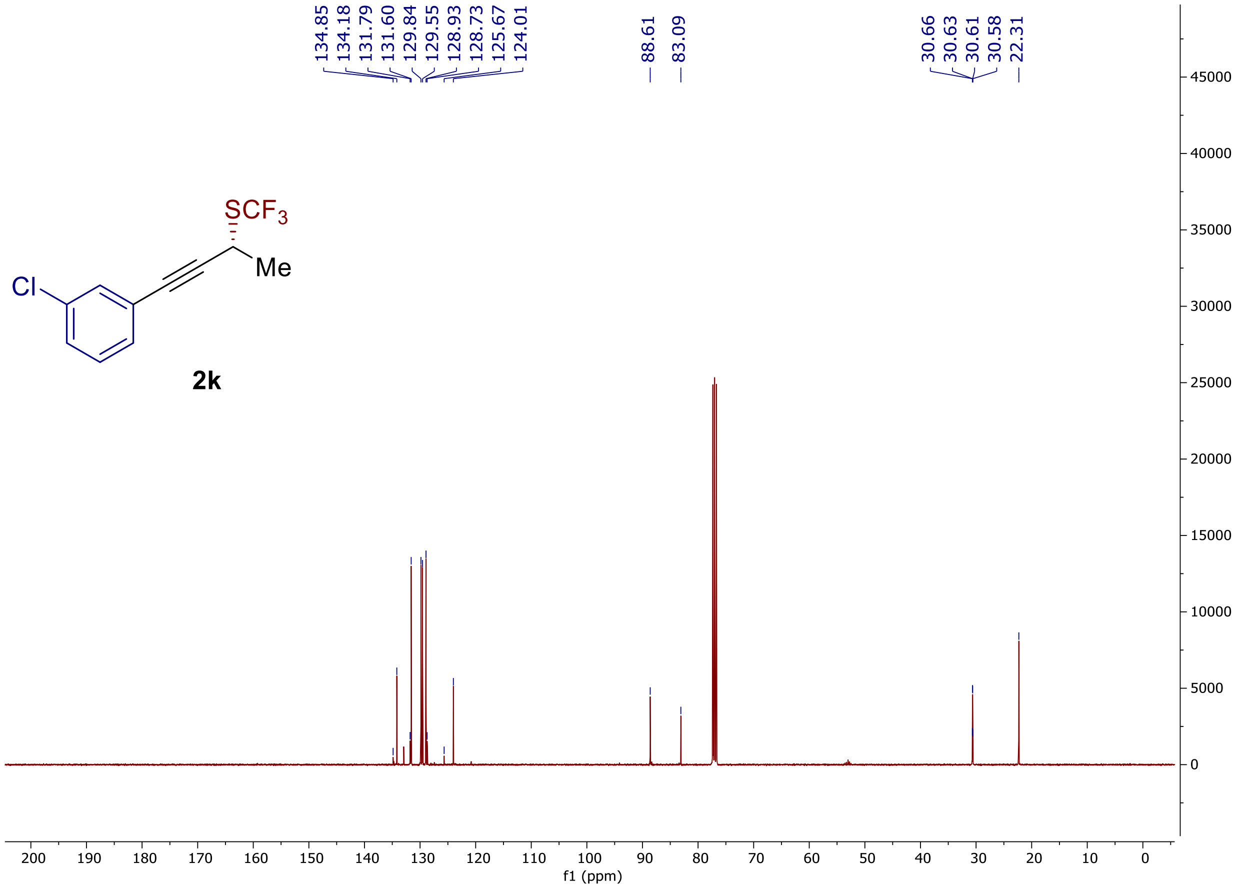

134.85
134.18
131.79
131.60
129.84
129.55
128.93
128.73
125.67
124.01
88.61
83.09
30.66
30.63
30.61
30.58
22.31
SCF3
Me
Cl
2k
45000
40000
35000
30000
25000
20000
15000
10000
5000
0
200
190
180
170
160
150
140
130
120
110
100
90
80
70
60
50
40
30
20
10
0
f1 (ppm)

^{1}H NMR:

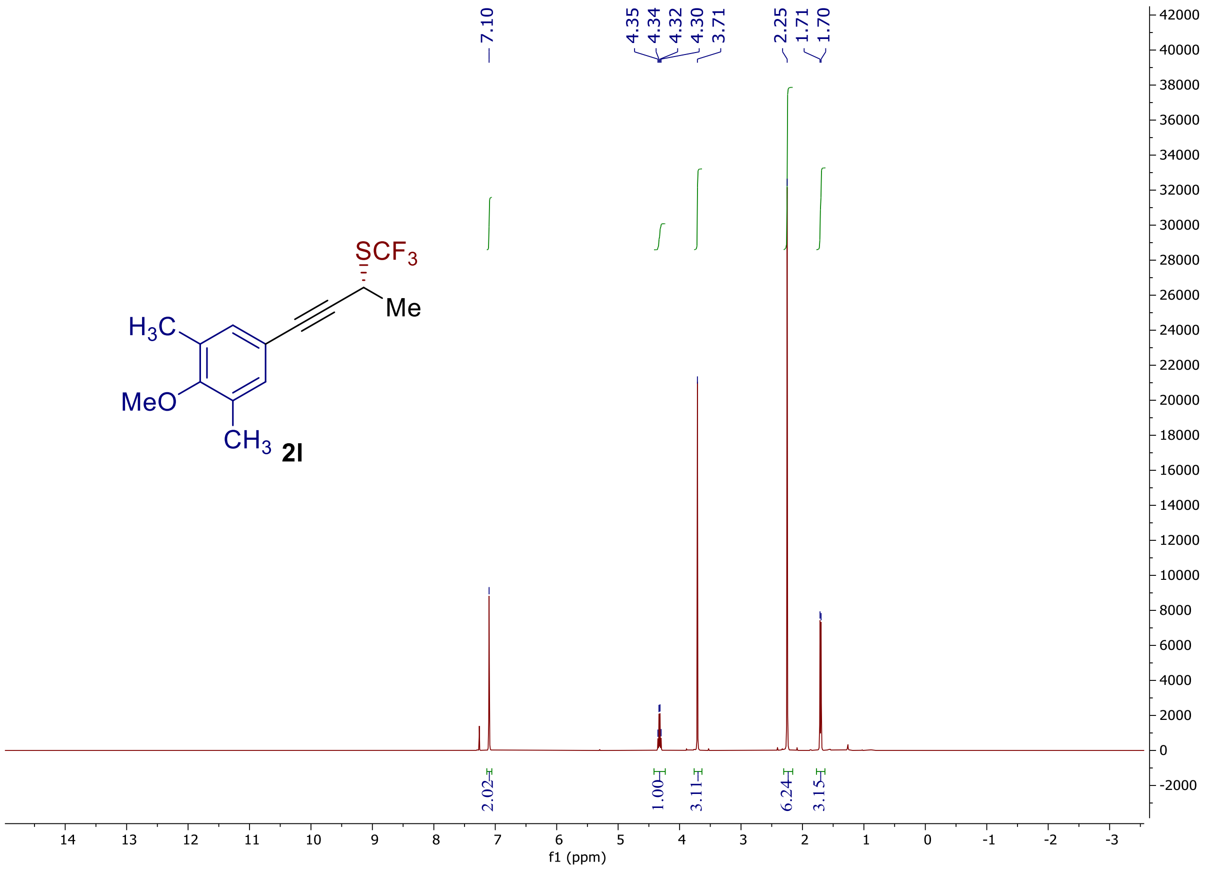

^{19}F NMR:

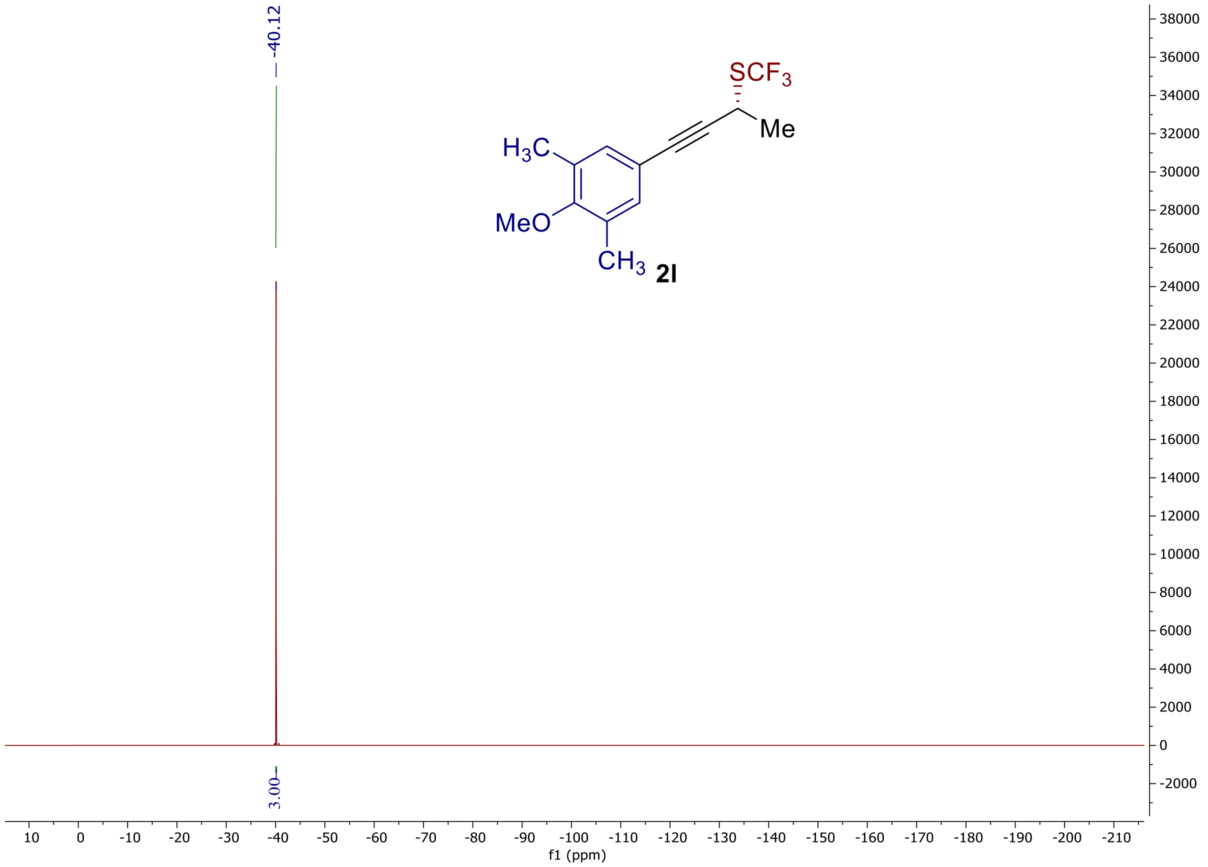

^{13}C NMR:

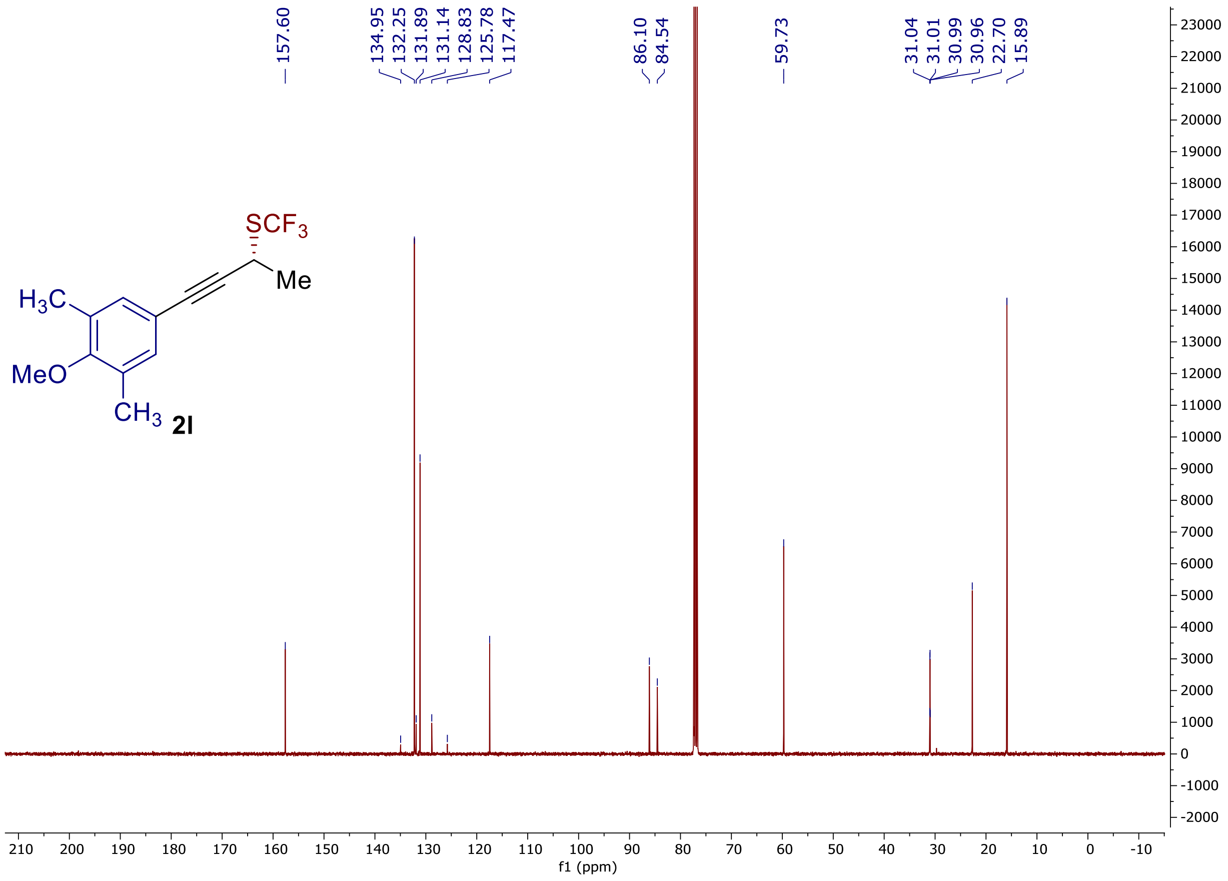
157.60
134.95
132.25
131.89
131.14
128.83
125.78
117.47
86.10
84.54
59.73
31.04
31.01
30.99
30.96
22.70
15.89
SCF3
Me
H3C
MeO
CH3
2l
f1 (ppm)

^{1}H NMR:

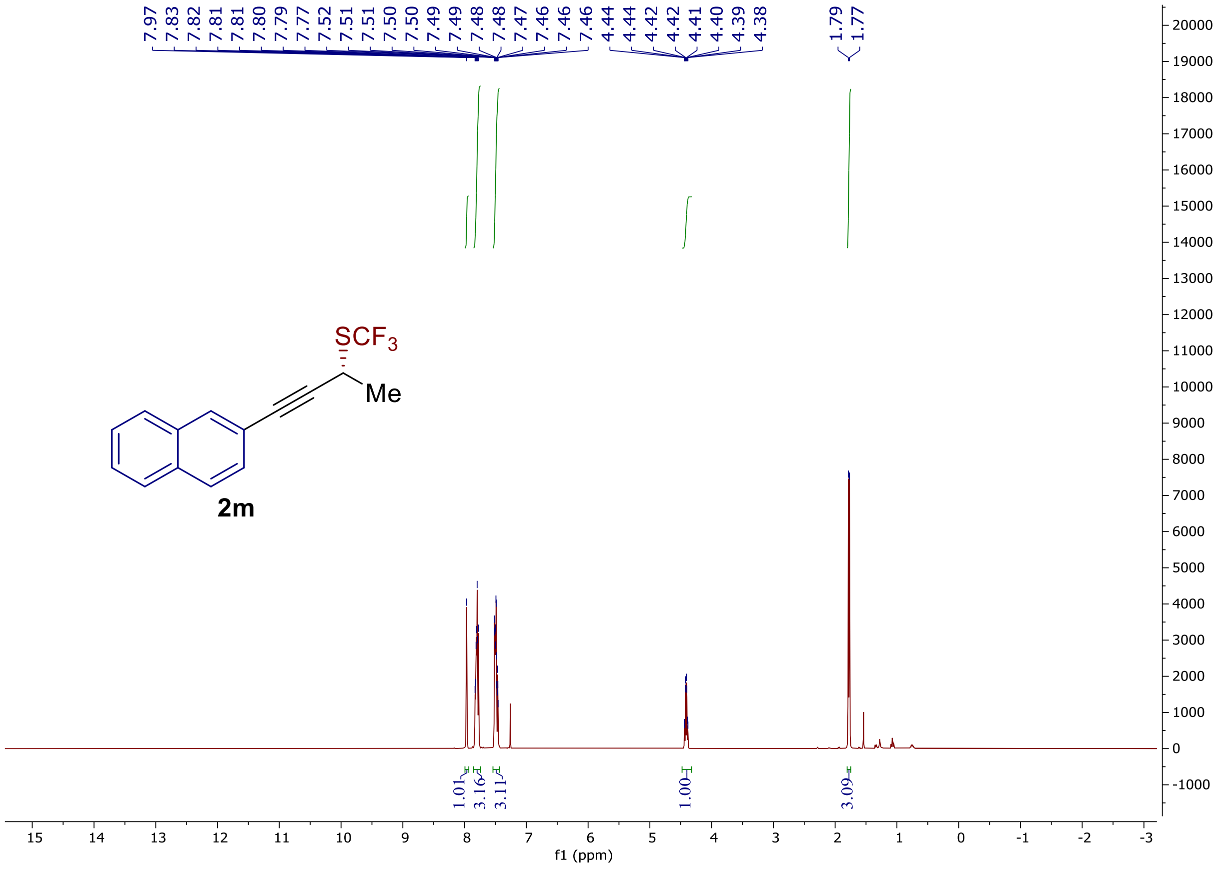

^{19}F NMR:

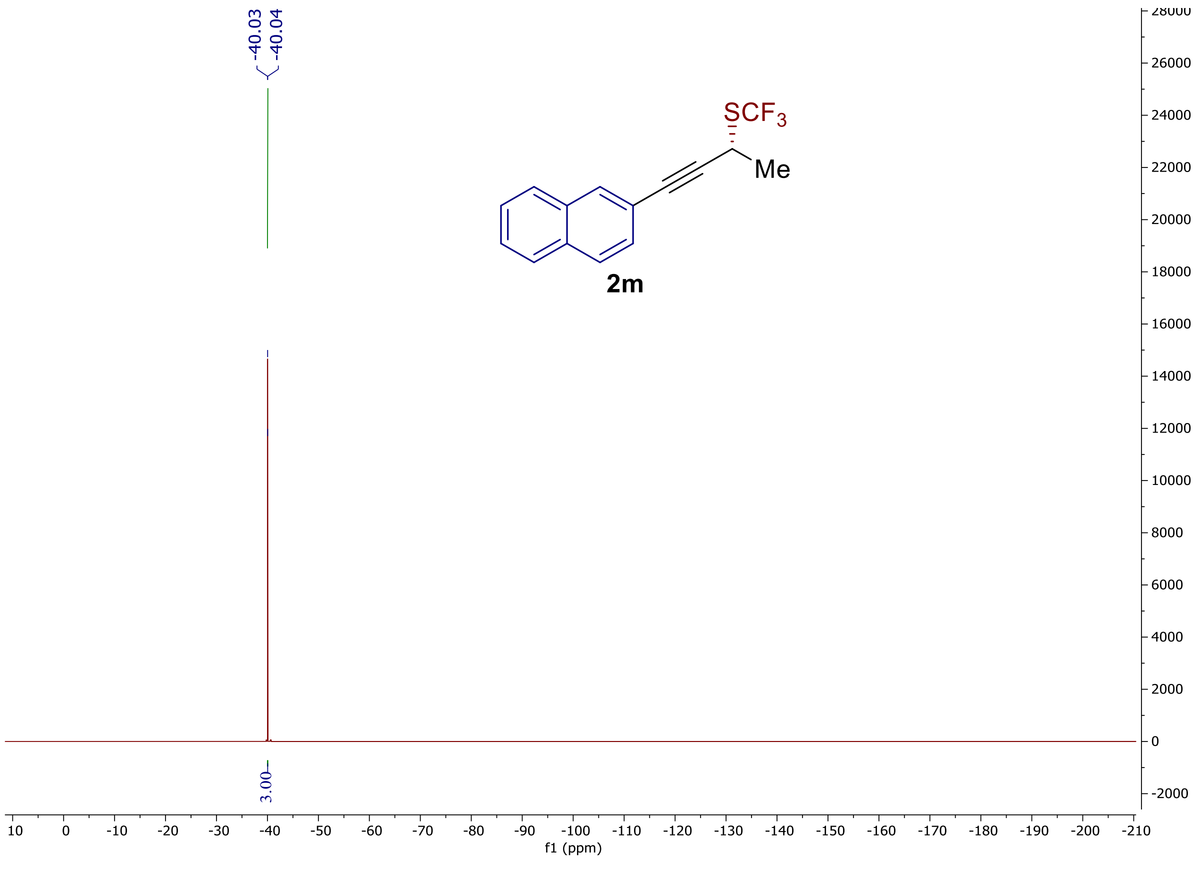

^{13}C NMR:

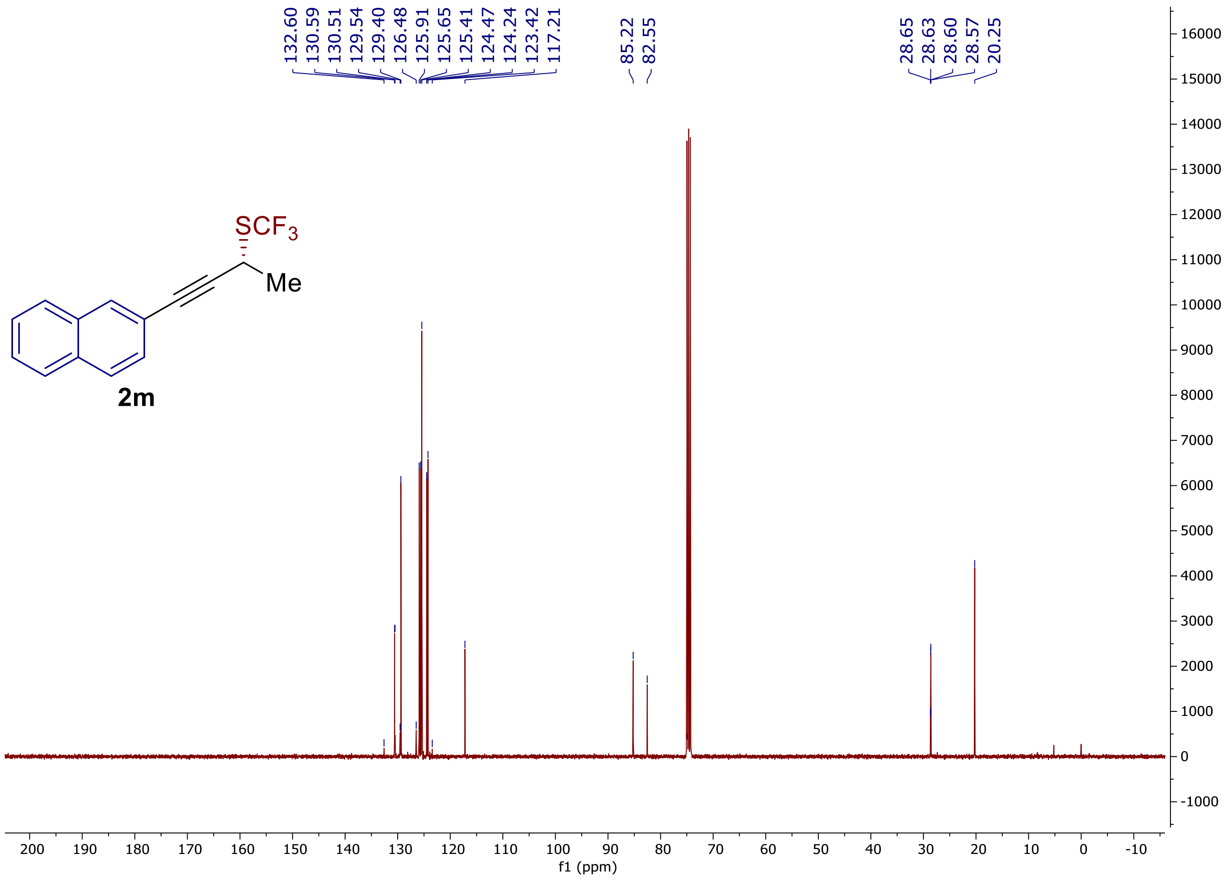

2m
132.60
130.59
130.51
129.54
129.40
126.48
125.91
125.65
125.41
124.47
124.24
123.42
117.21
85.22
82.55
28.65
28.63
28.60
28.57
20.25
f1 (ppm)

^{1}H NMR:

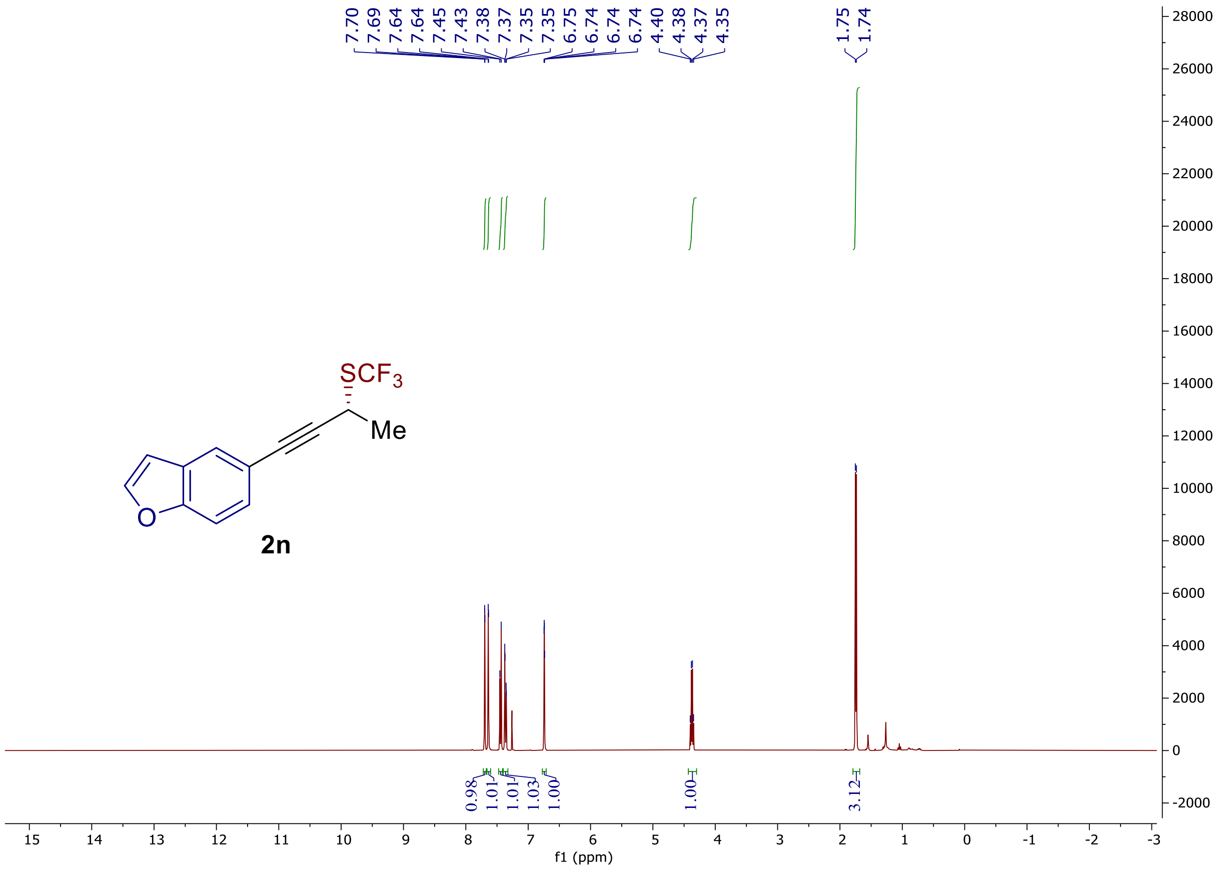

^{19}F NMR:

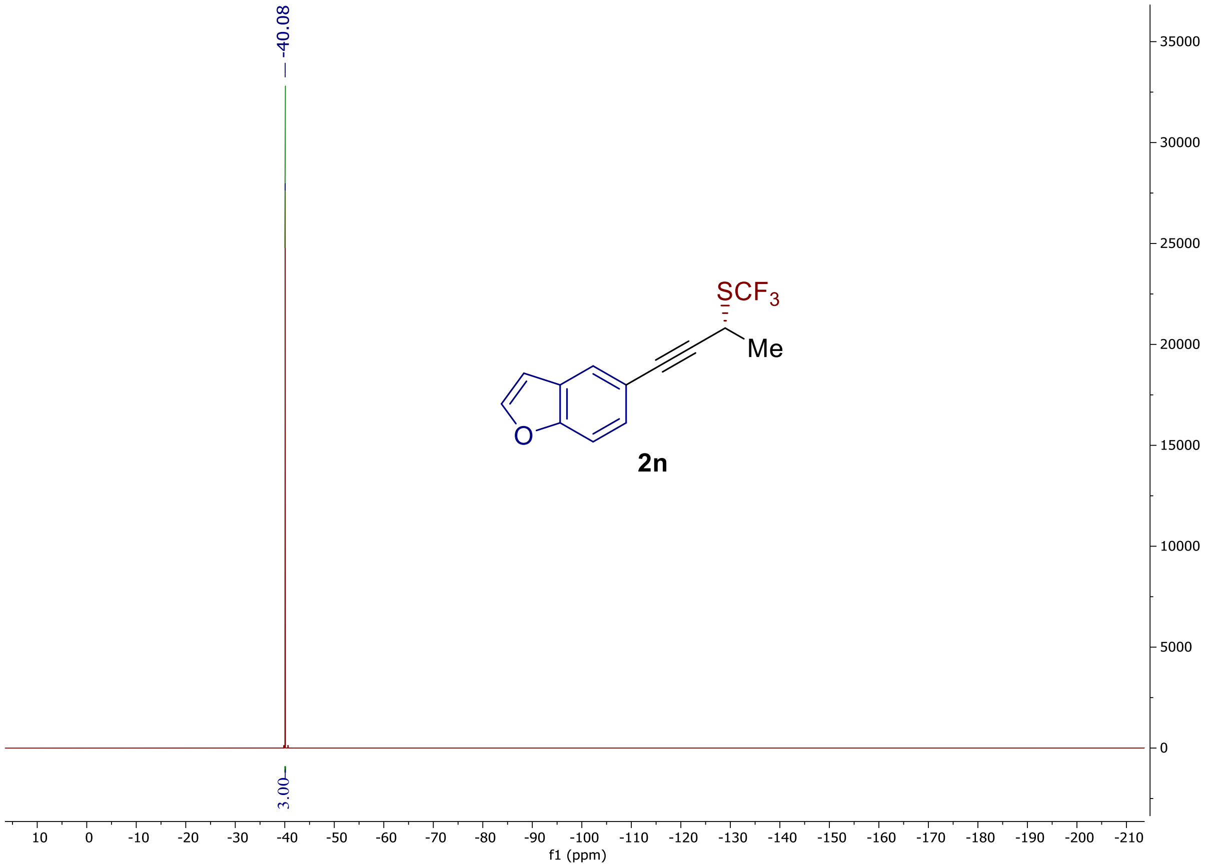

^{13}C NMR:

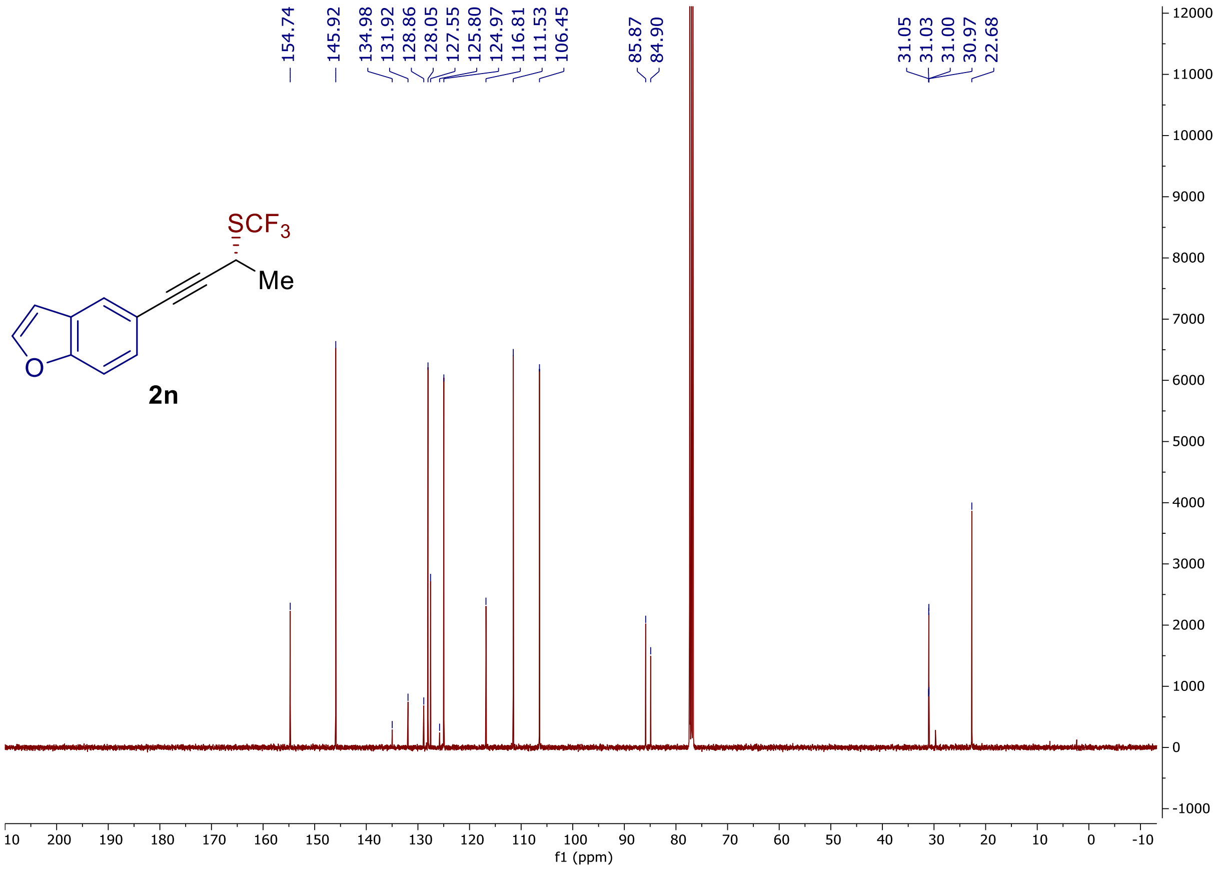
154.74
145.92
134.98
131.92
128.86
128.05
127.55
125.80
124.97
116.81
111.53
106.45
85.87
84.90
31.05
31.03
31.00
30.97
22.68
SCF3
Me
O
2n
f1 (ppm)

^{1}H NMR:

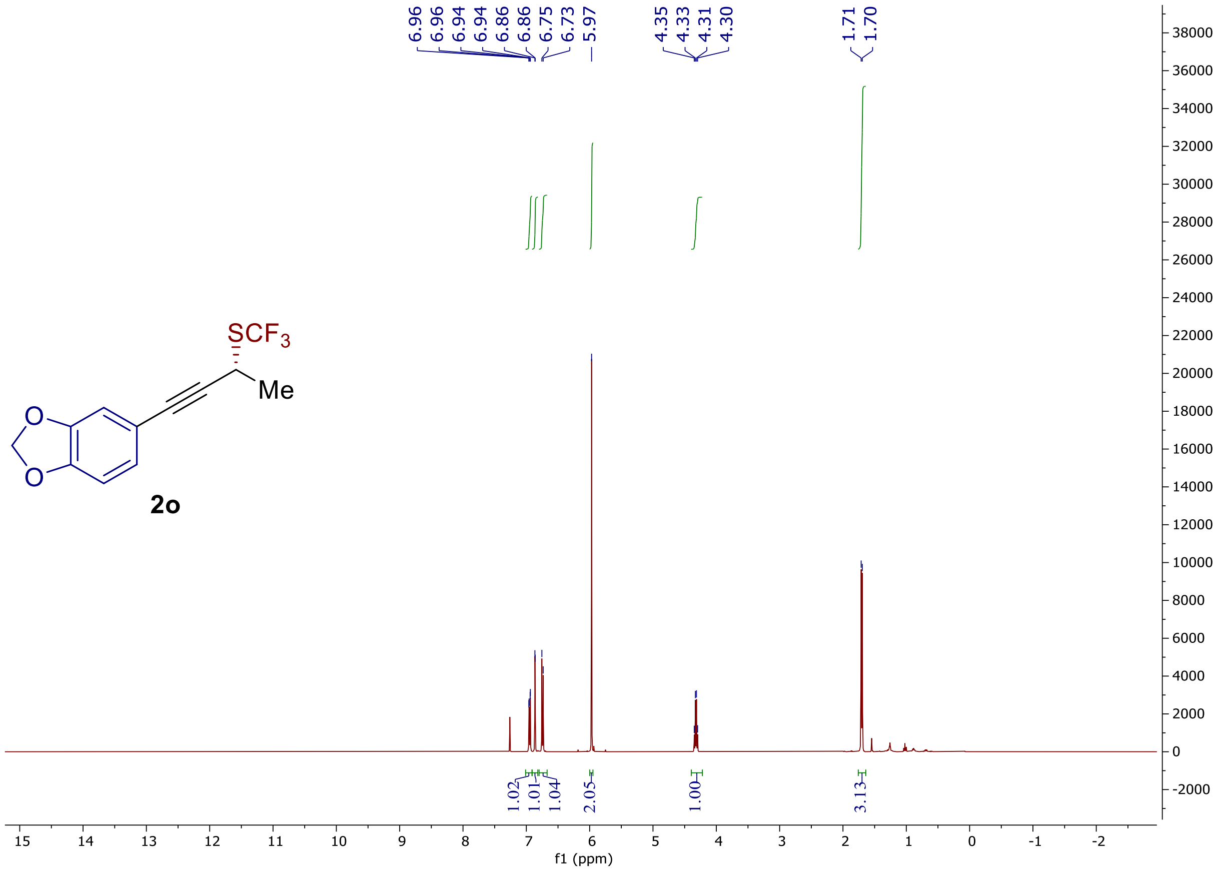

^{19}F NMR:

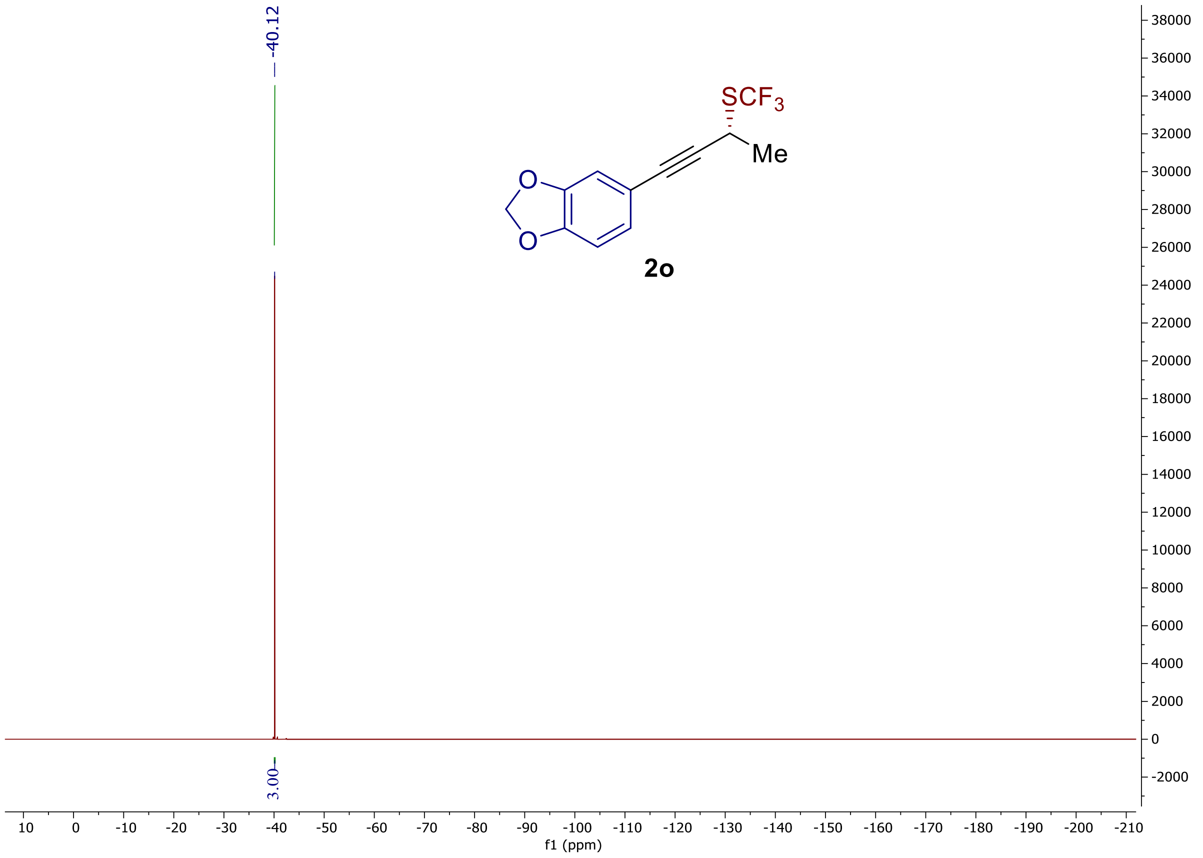

^{13}C NMR:

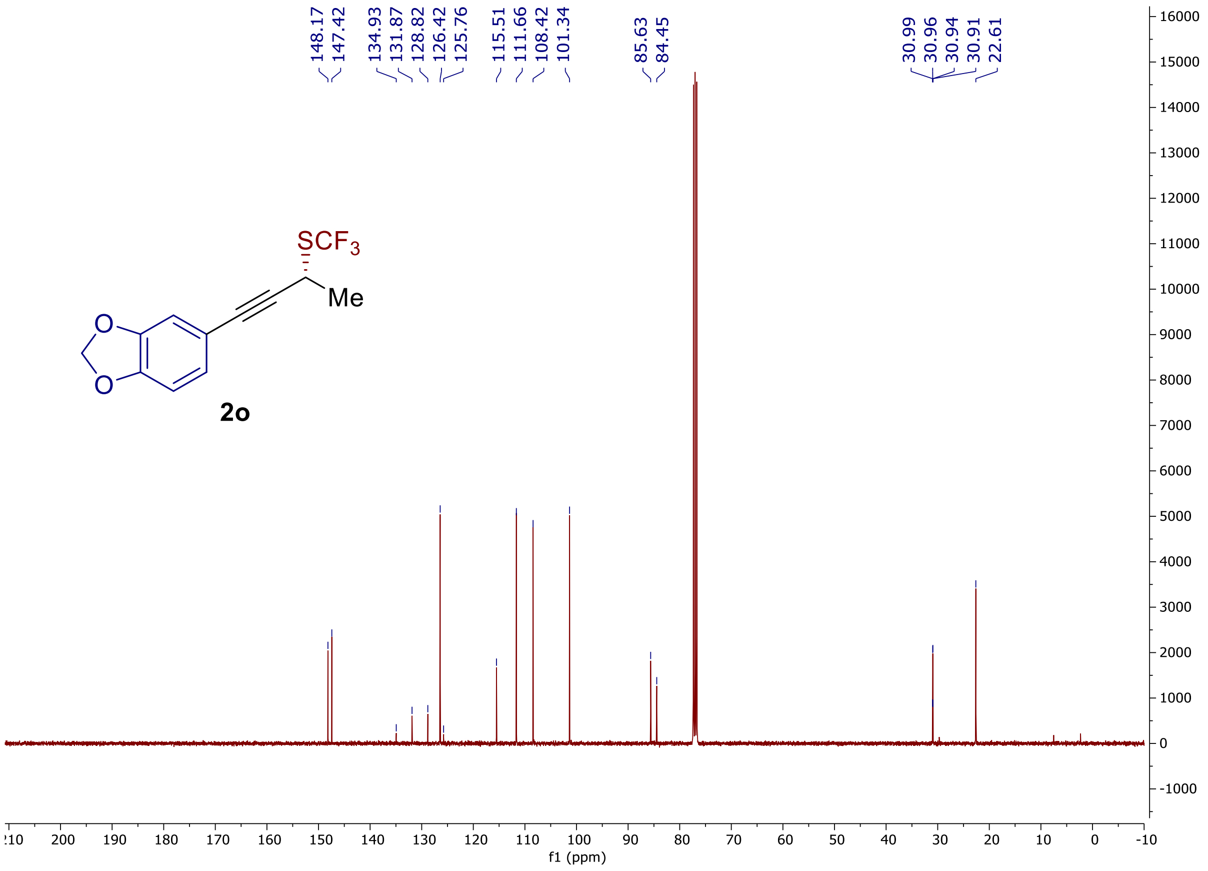

148.17
147.42
134.93
131.87
128.82
126.42
125.76
115.51
111.66
108.42
101.34
85.63
84.45
30.99
30.96
30.94
30.91
22.61
SCF3
Me
O
O
2o
f1 (ppm)

^{1}H NMR:

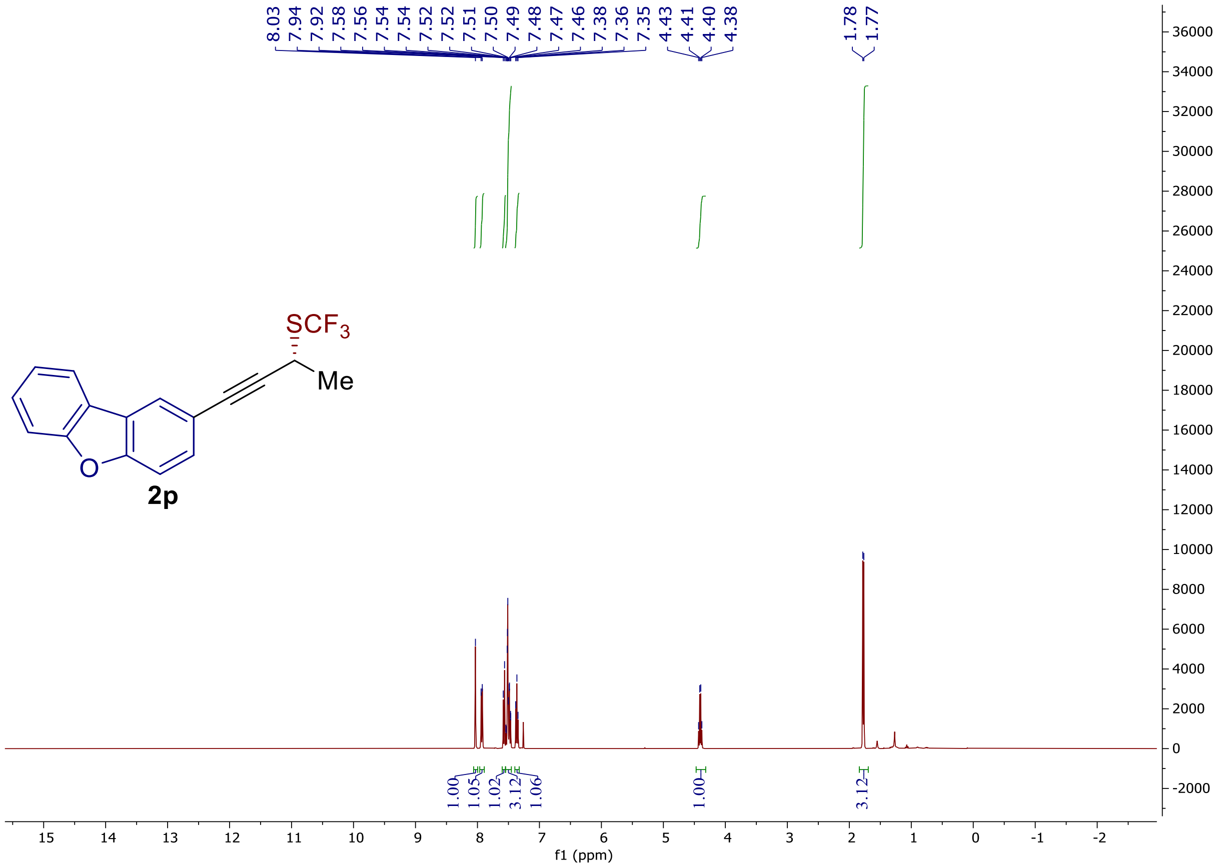

^{19}F NMR:

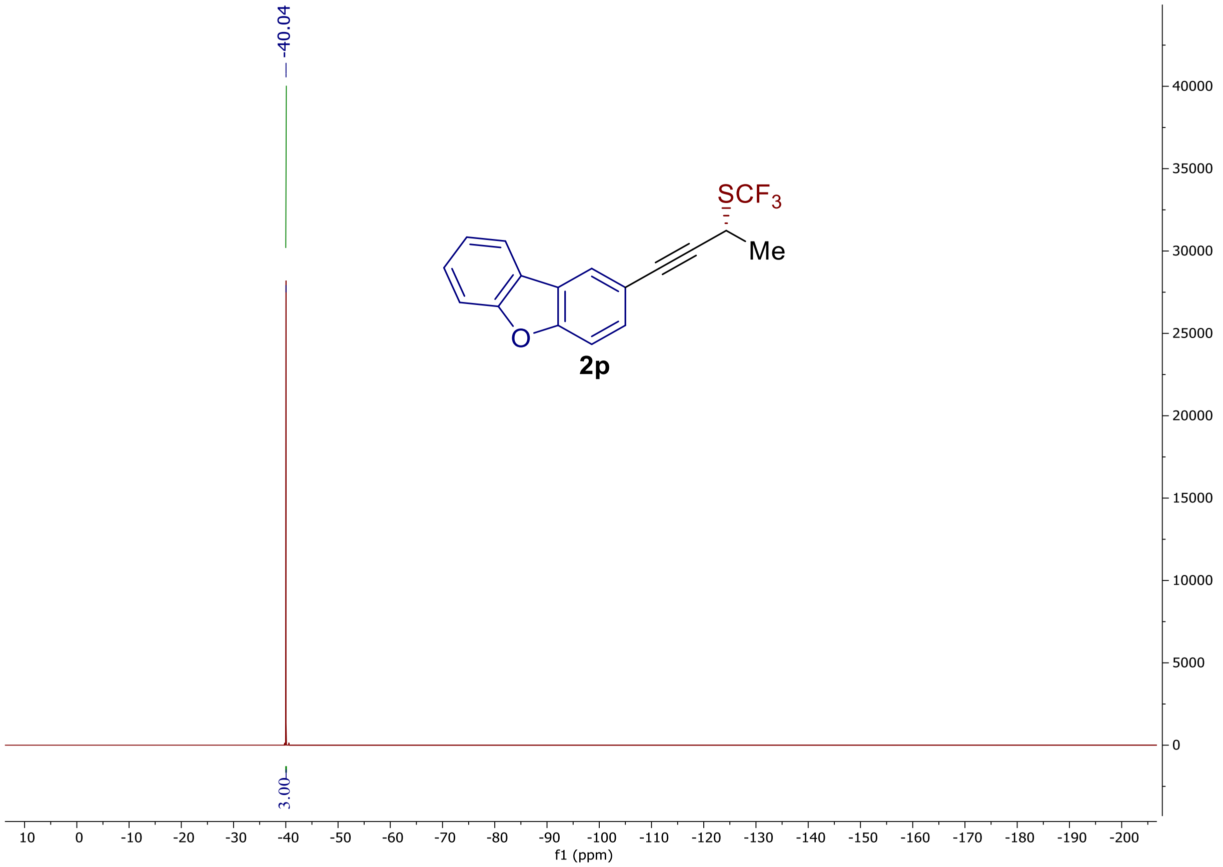

^{13}C NMR:

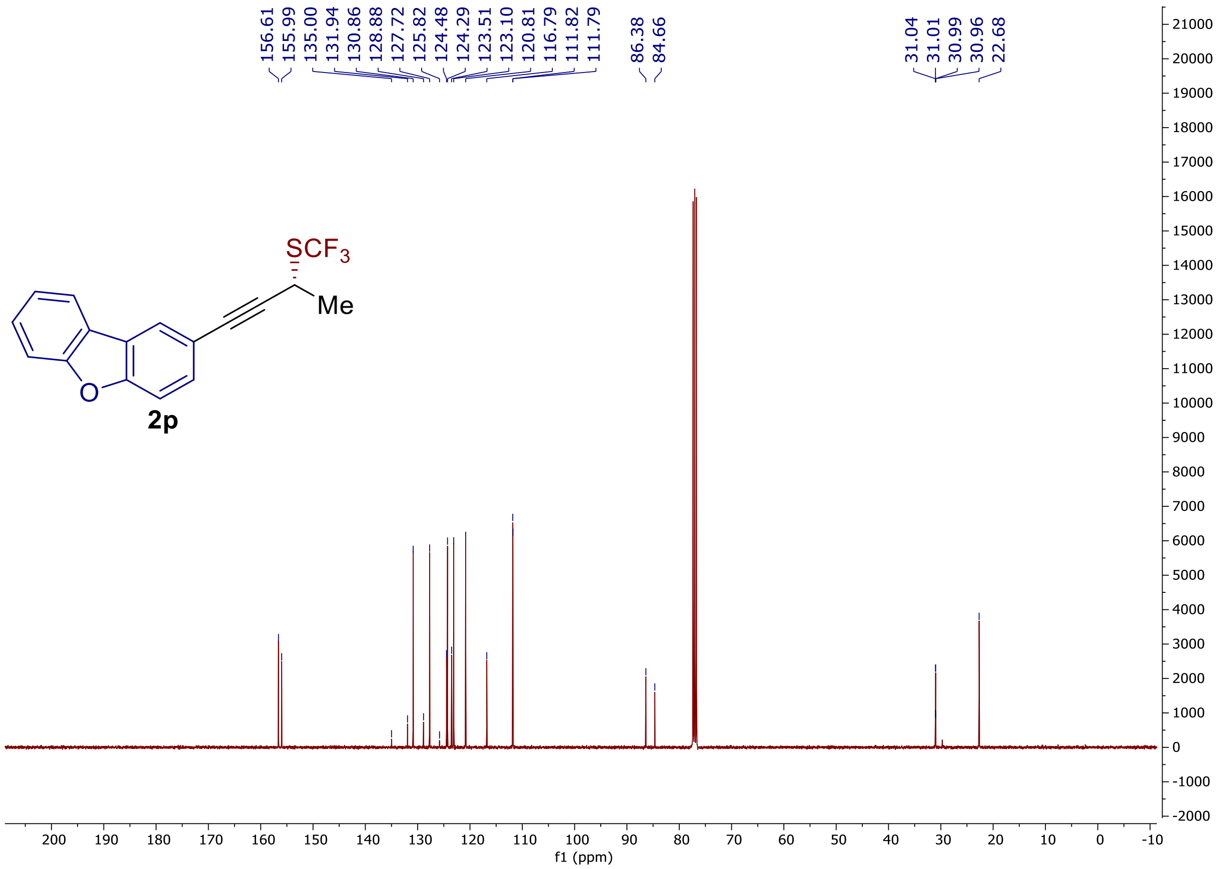

156.61
155.99
135.00
131.94
130.86
128.88
127.72
125.82
124.48
124.29
123.51
123.10
120.81
116.79
111.82
111.79
86.38
84.66
31.04
31.01
30.99
30.96
22.68
SCF3
Me
O
2p
f1 (ppm)

^{1}H NMR:

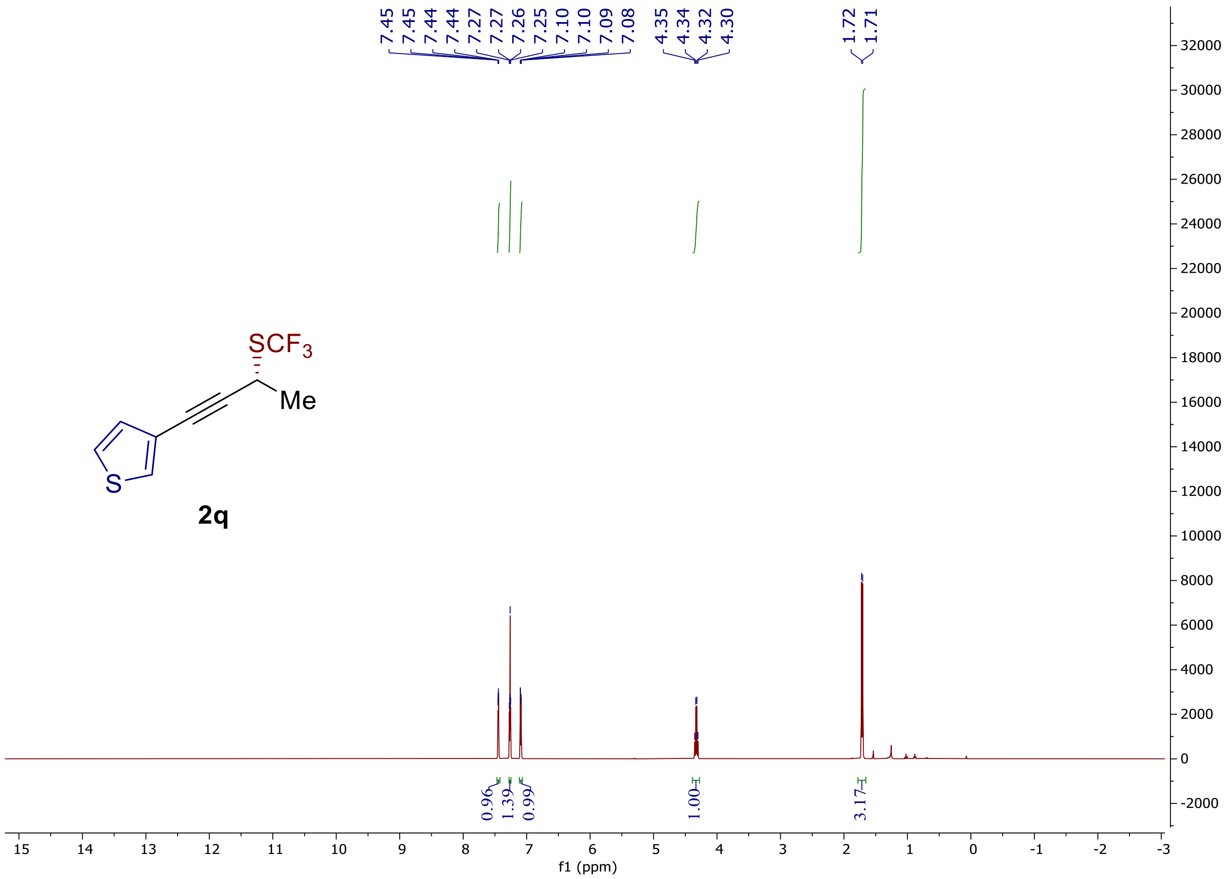

^{19}F NMR:

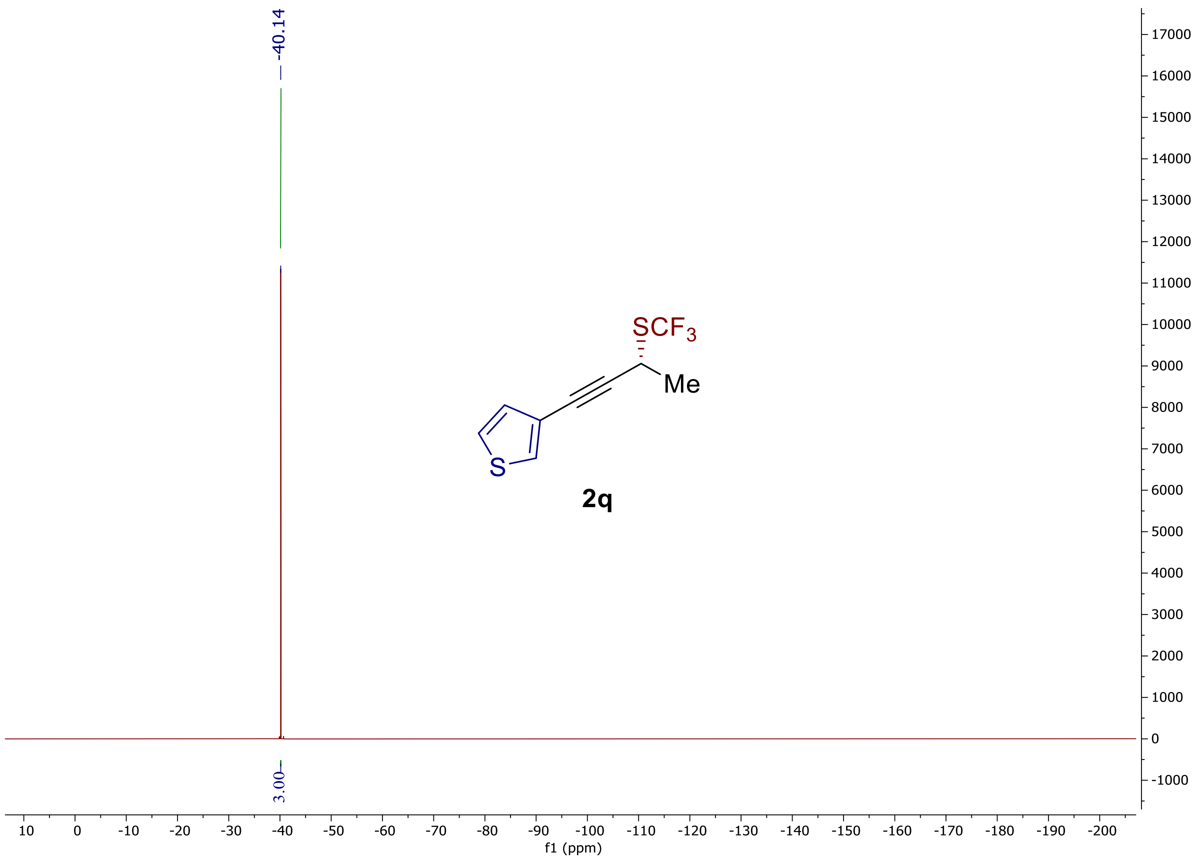

^{13}C NMR:

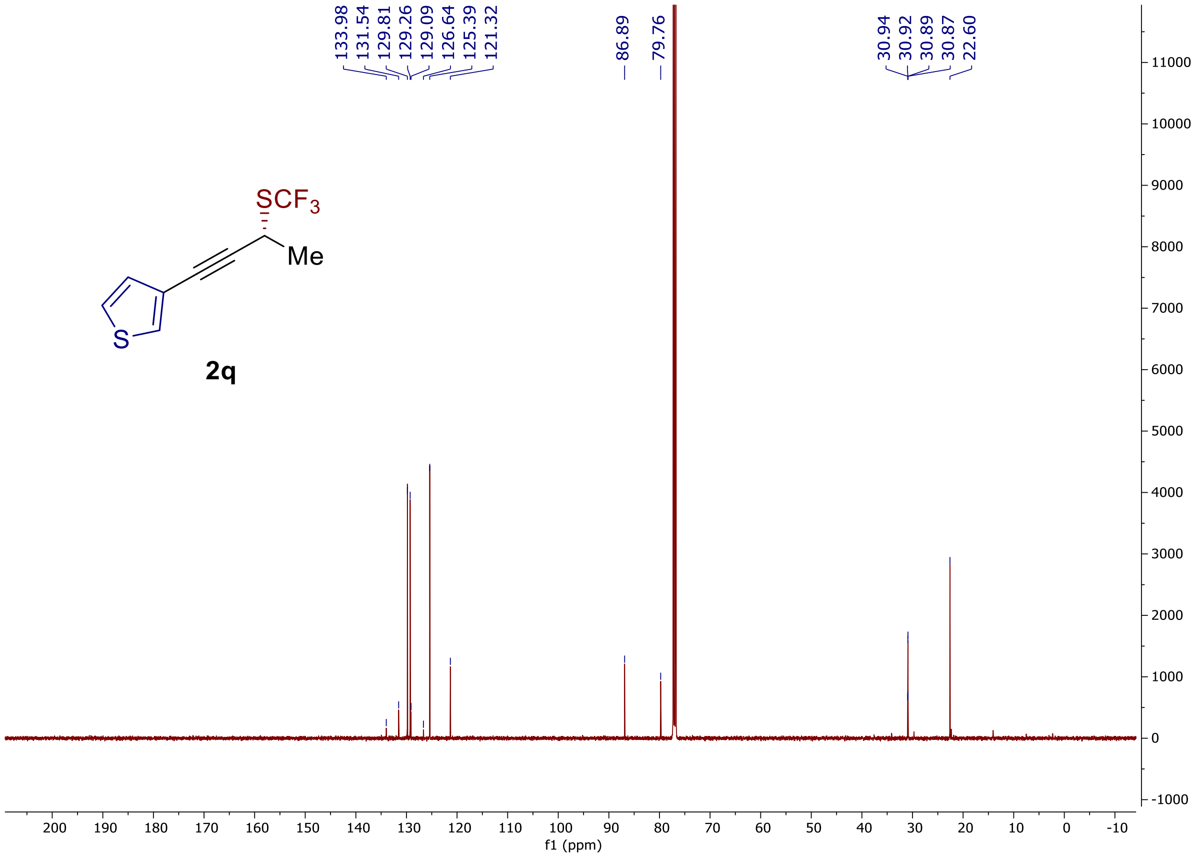

133.98
131.54
129.81
129.26
129.09
126.64
125.39
121.32
86.89
79.76
30.94
30.92
30.89
30.87
22.60
SCF3
Me
S
2q
f1 (ppm)

^{1}H NMR:

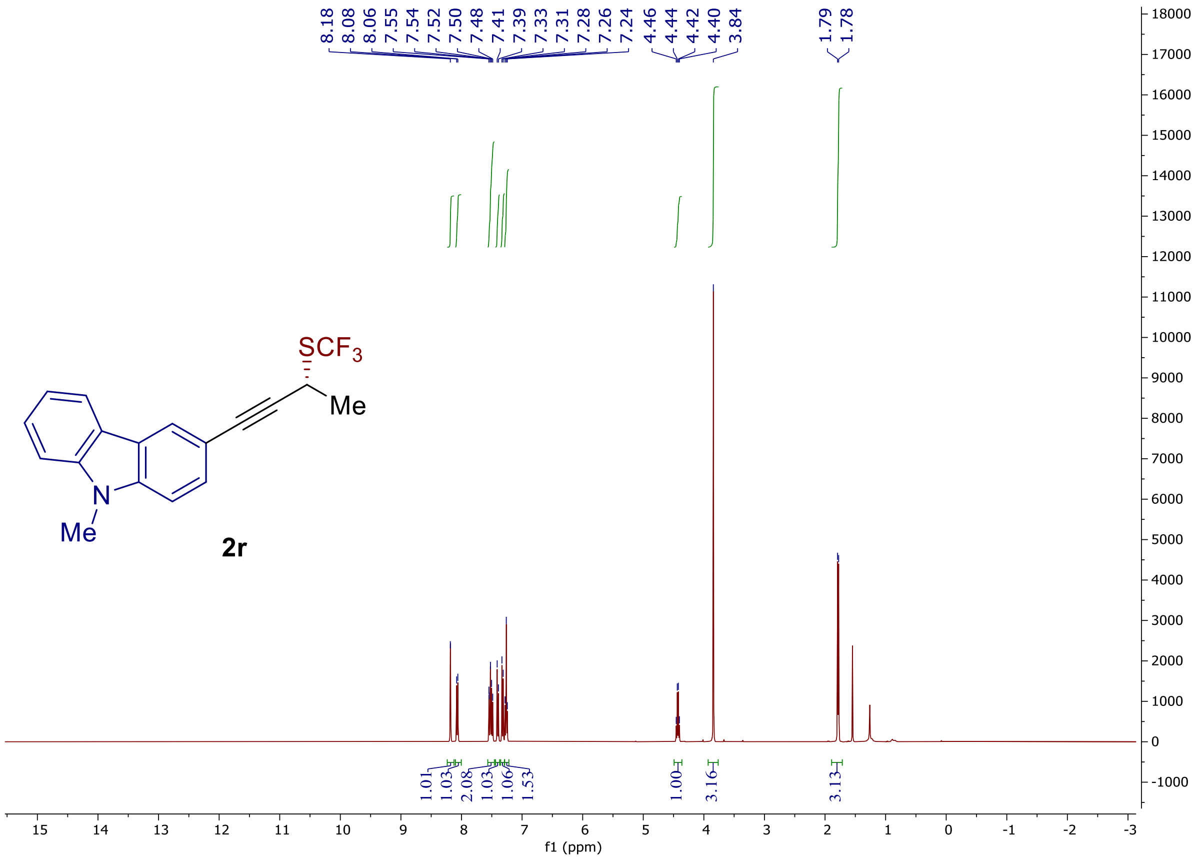

^{19}F NMR:

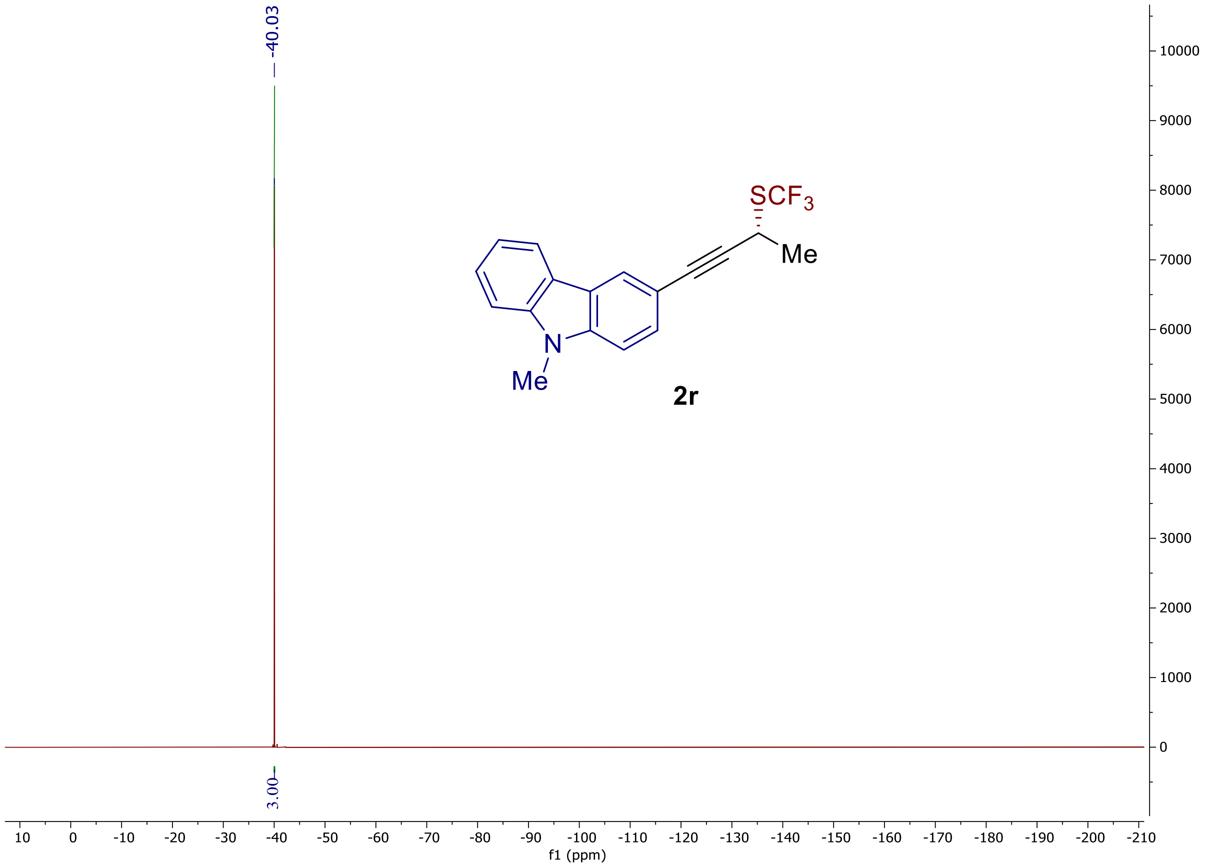

^{13}C NMR:

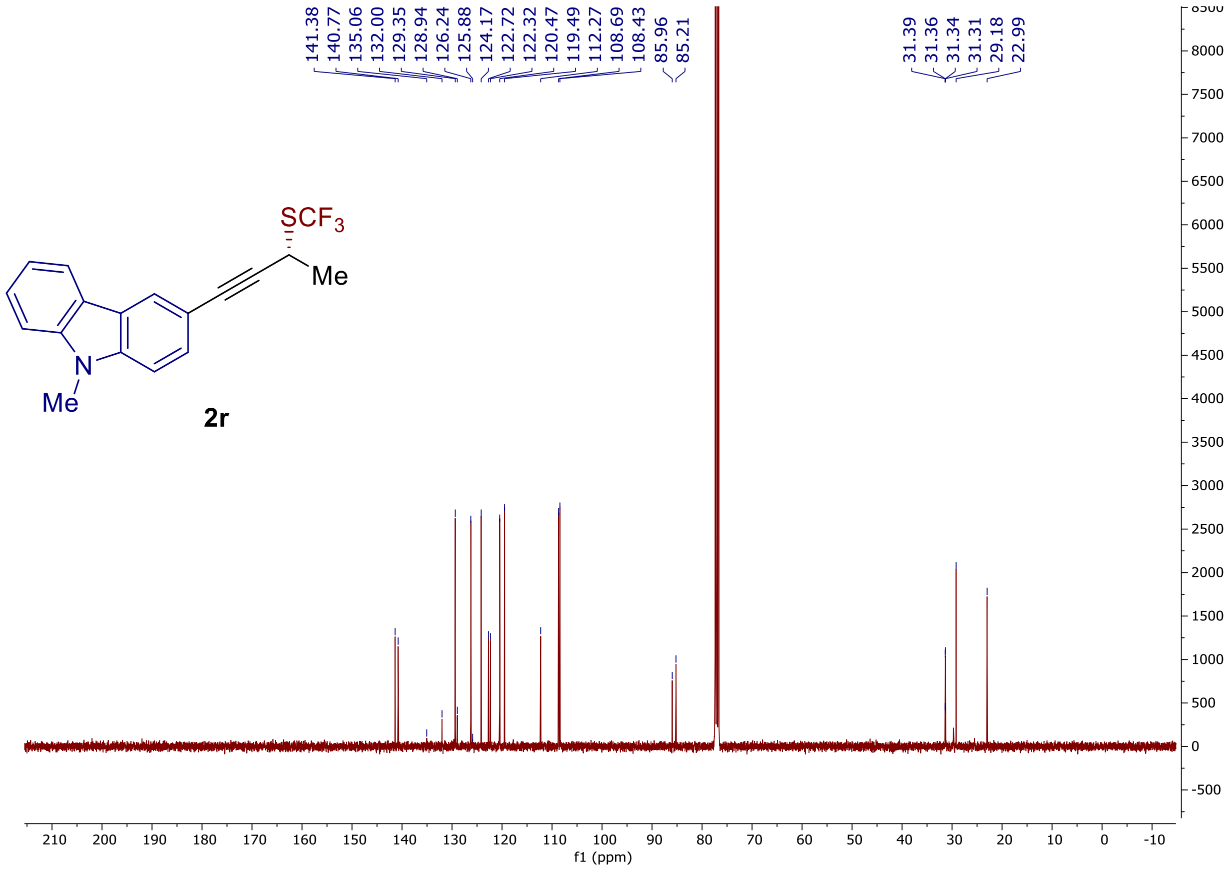

141.38
140.77
135.06
132.00
129.35
128.94
126.24
125.88
124.17
122.72
122.32
120.47
119.49
112.27
108.69
108.43
85.96
85.21
31.39
31.36
31.34
31.31
29.18
22.99
SCF3
Me
N
Me
2r
f1 (ppm)

^{1}H NMR:

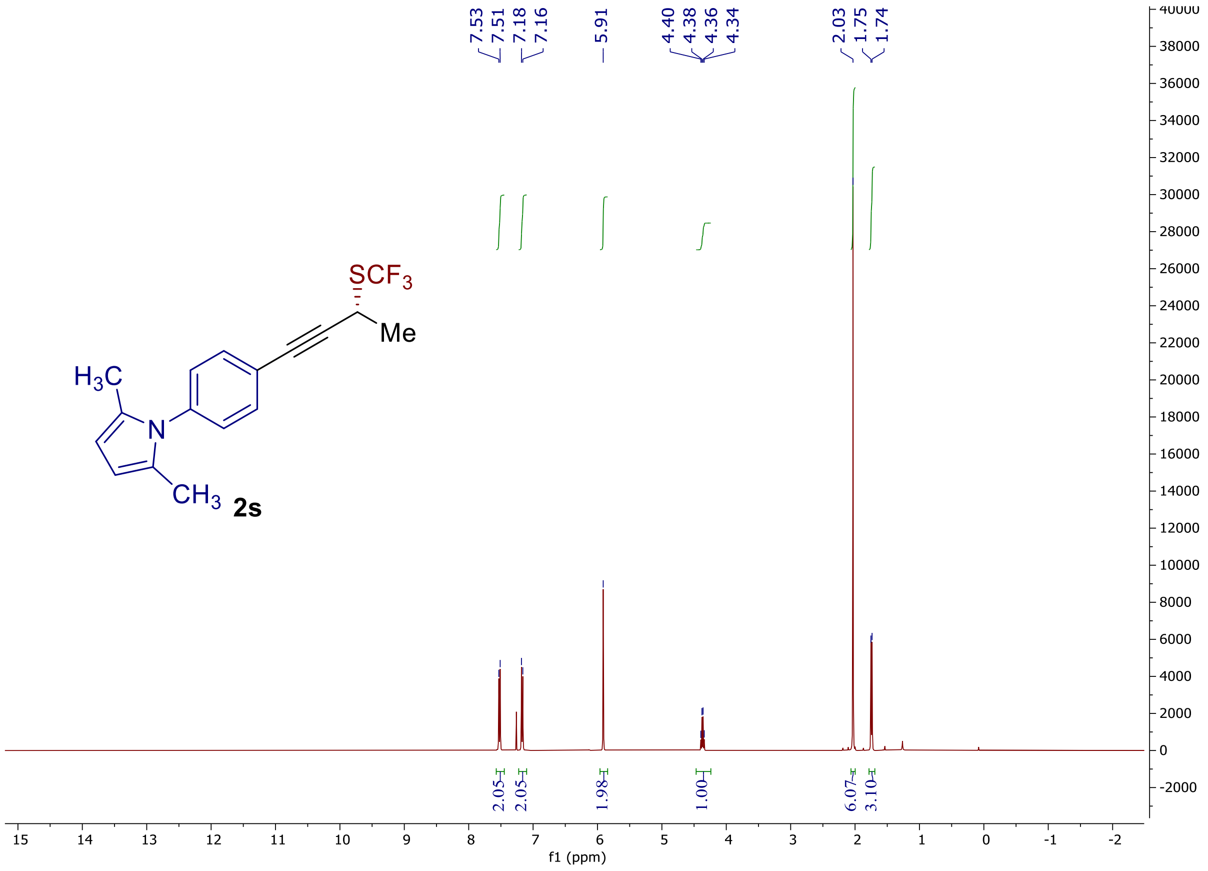

^{19}F NMR:

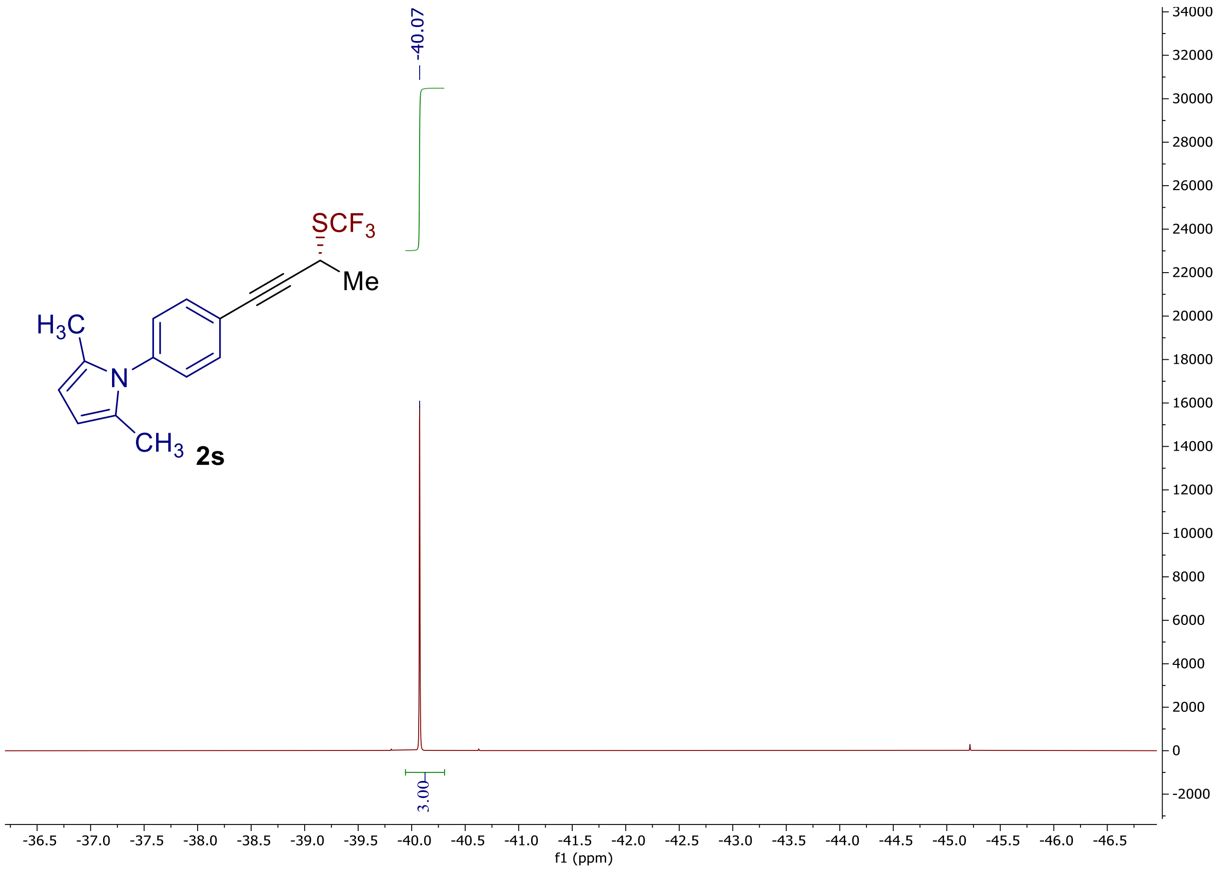

^{13}C NMR:

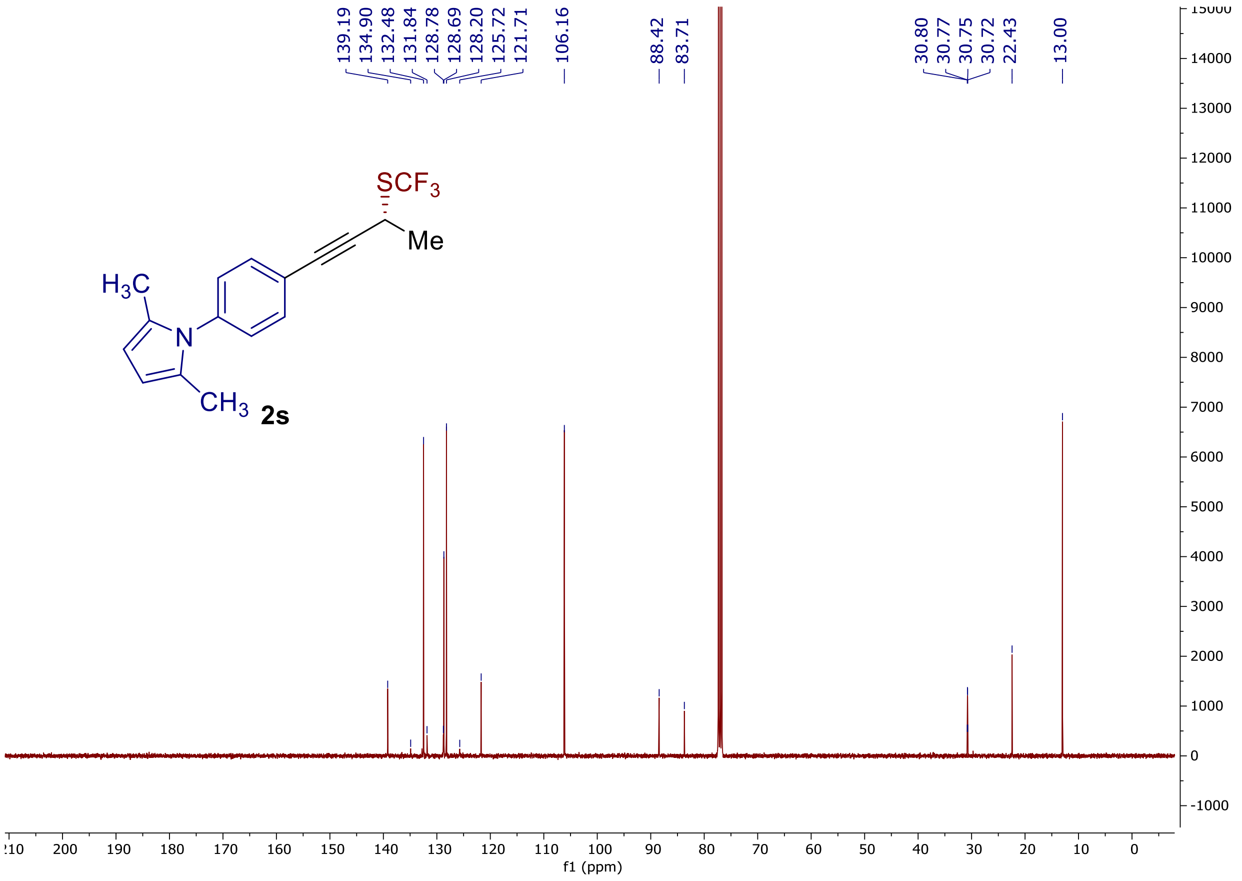
139.19
134.90
132.48
131.84
128.78
128.69
128.20
125.72
121.71
106.16
88.42
83.71
30.80
30.77
30.75
30.72
22.43
13.00
SCF3
Me
H3C
N
CH3
2s
f1 (ppm)

^{1}H NMR:

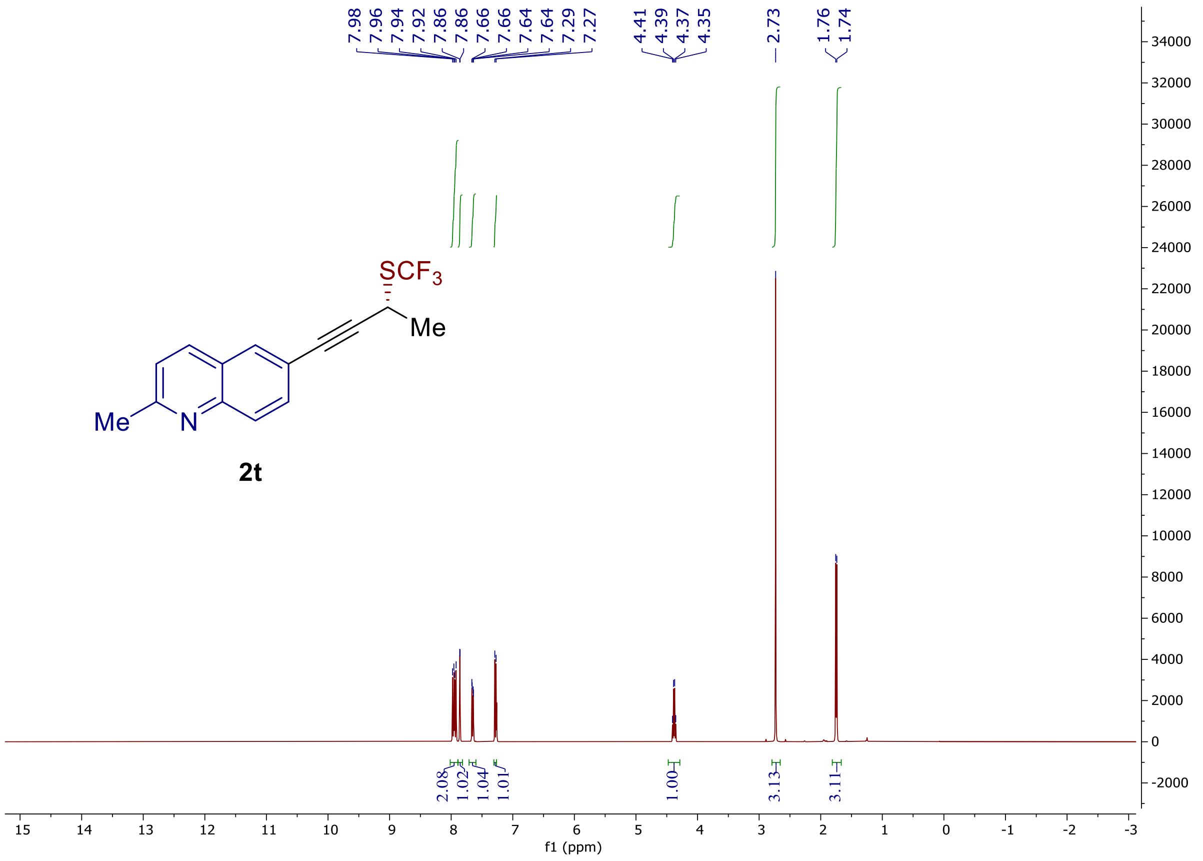

^{19}F NMR:

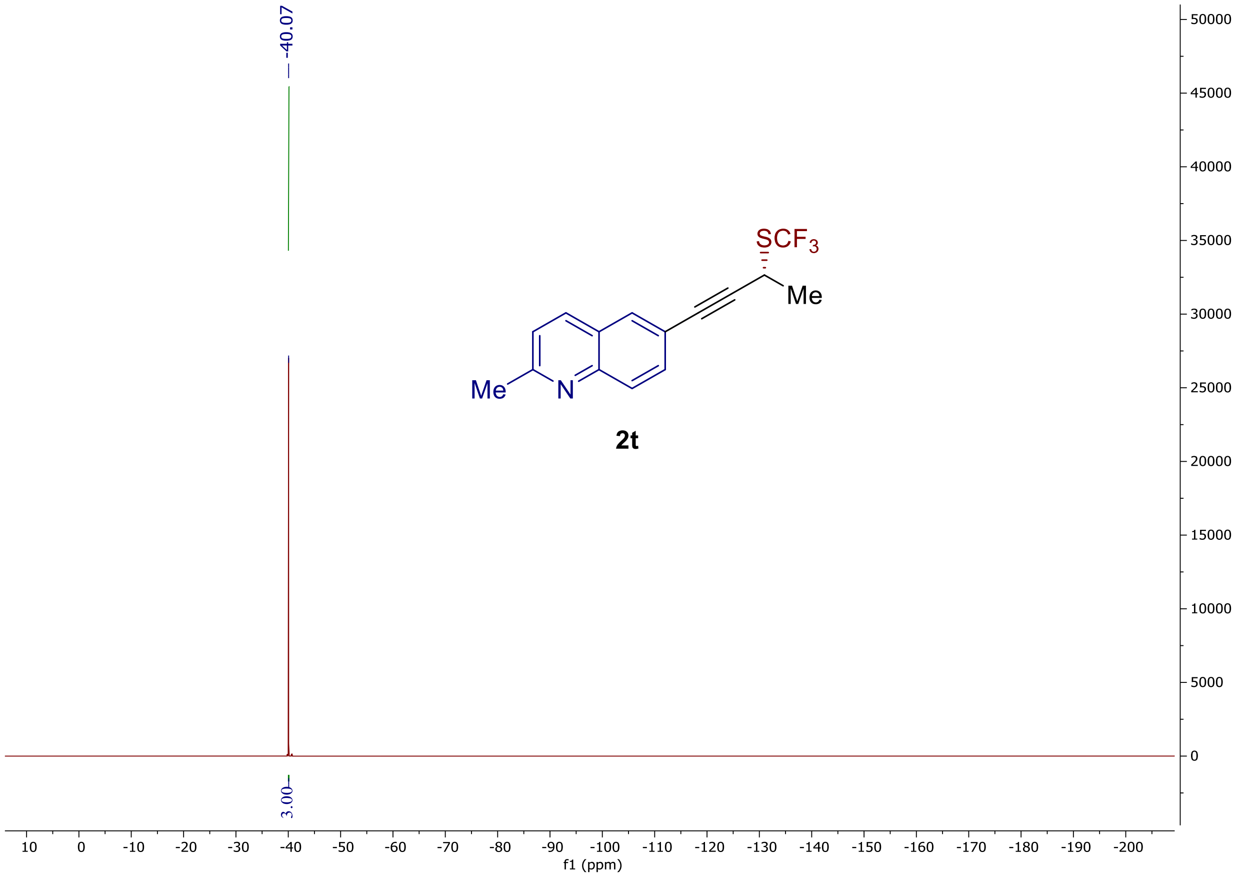

^{13}C NMR:

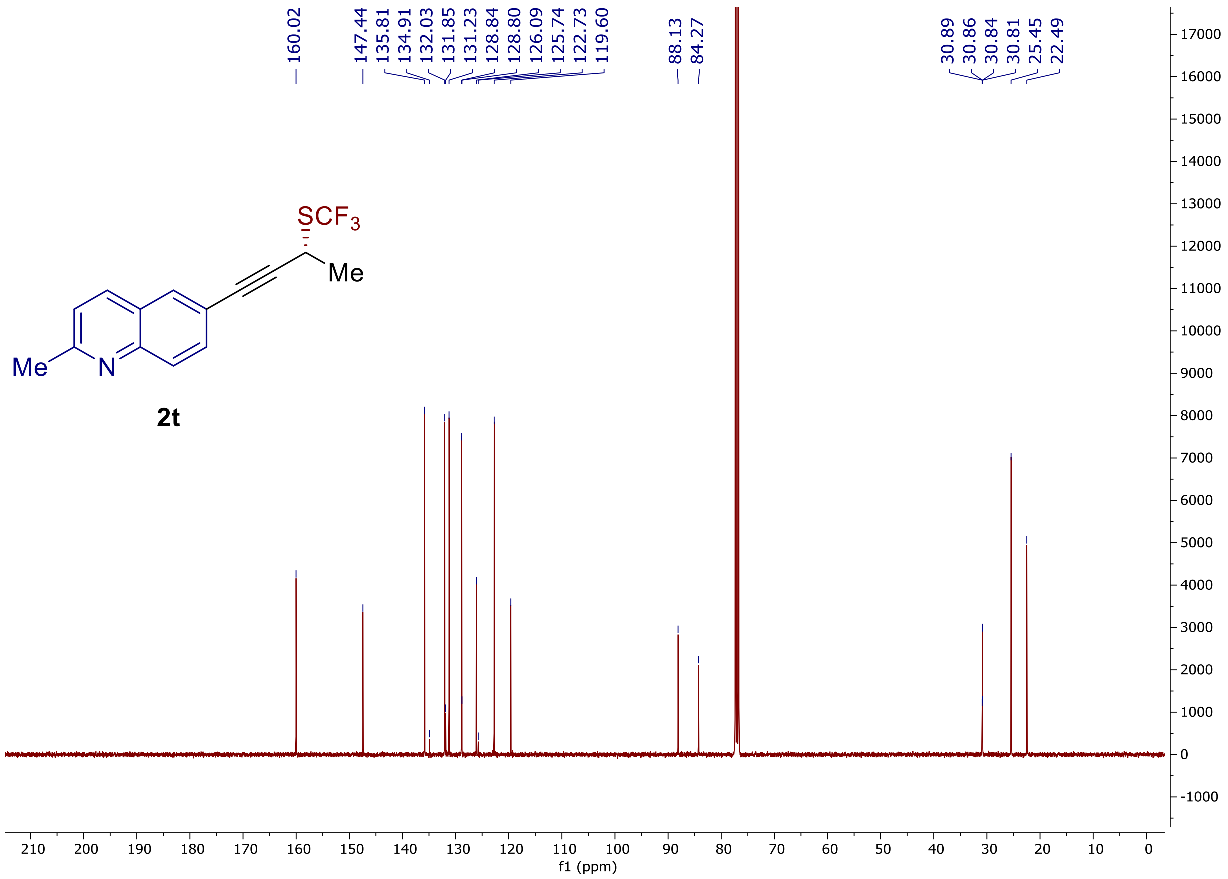

160.02
147.44
135.81
134.91
132.03
131.85
131.23
128.84
128.80
126.09
125.74
122.73
119.60
88.13
84.27
30.89
30.86
30.84
30.81
25.45
22.49
SCF3
Me
Me
N
2t
f1 (ppm)

^{1}H NMR:

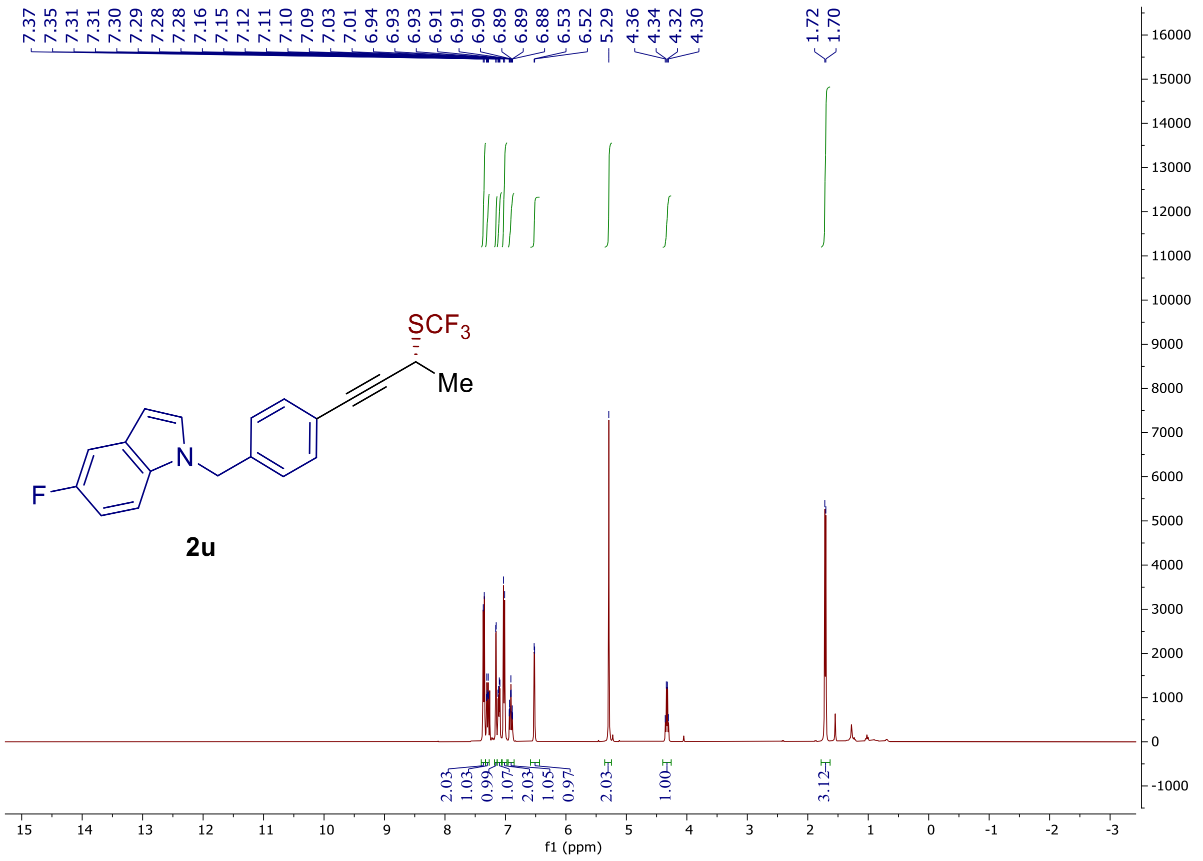

^{19}F NMR:

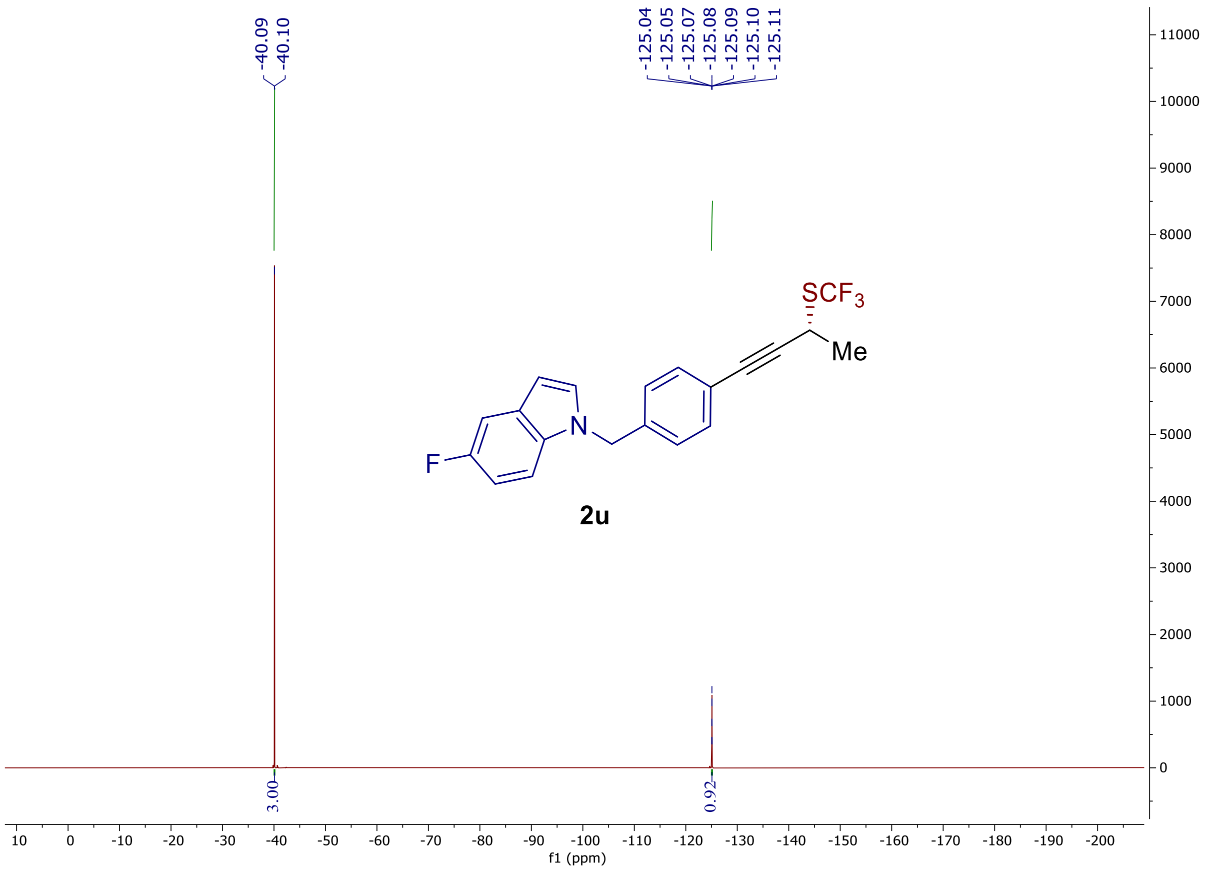

^{13}C NMR:

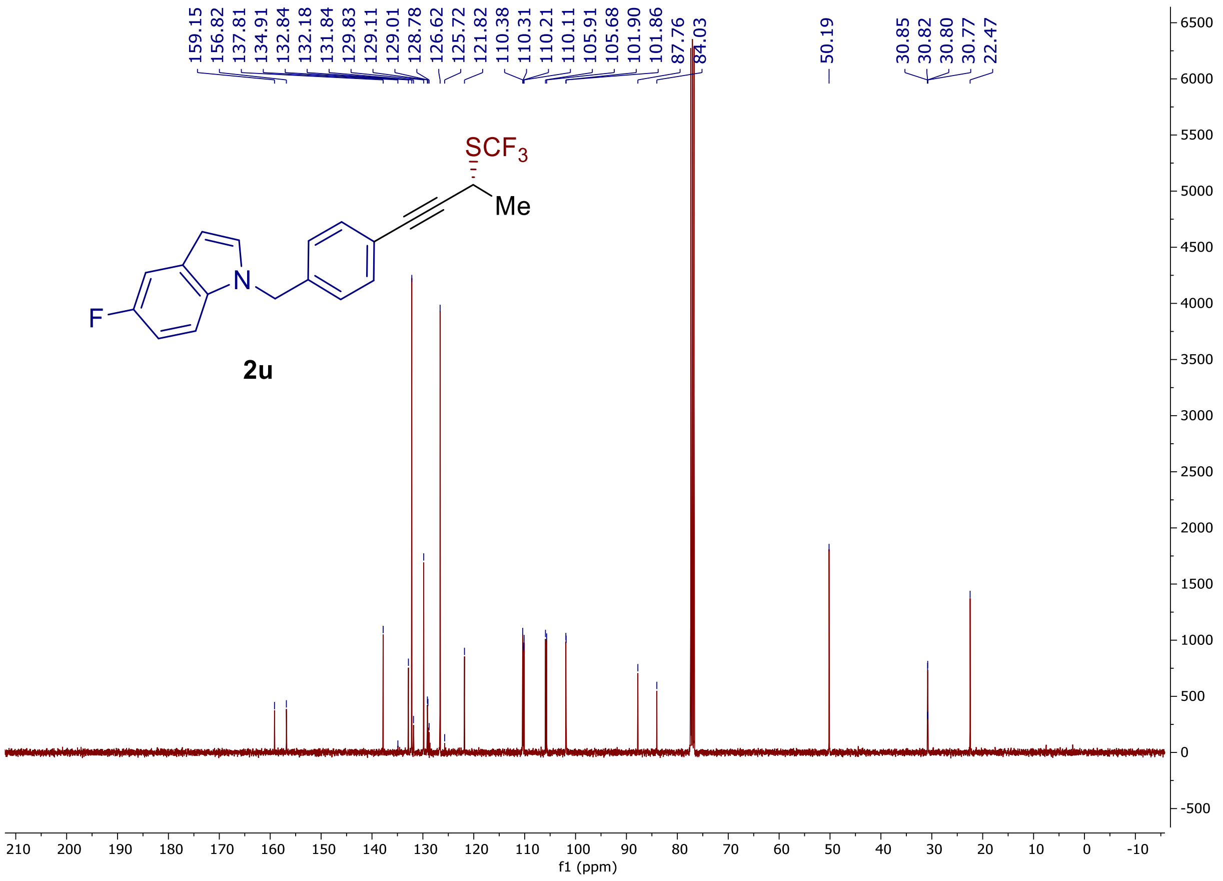

159.15
156.82
137.81
134.91
132.84
132.18
131.84
129.83
129.11
129.01
128.78
126.62
125.72
121.82
110.38
110.31
110.21
110.11
105.91
105.68
101.90
101.86
87.76
84.03
50.19
30.85
30.82
30.80
30.77
22.47
SCF3
Me
N
F
2u
f1 (ppm)

^{1}H NMR:

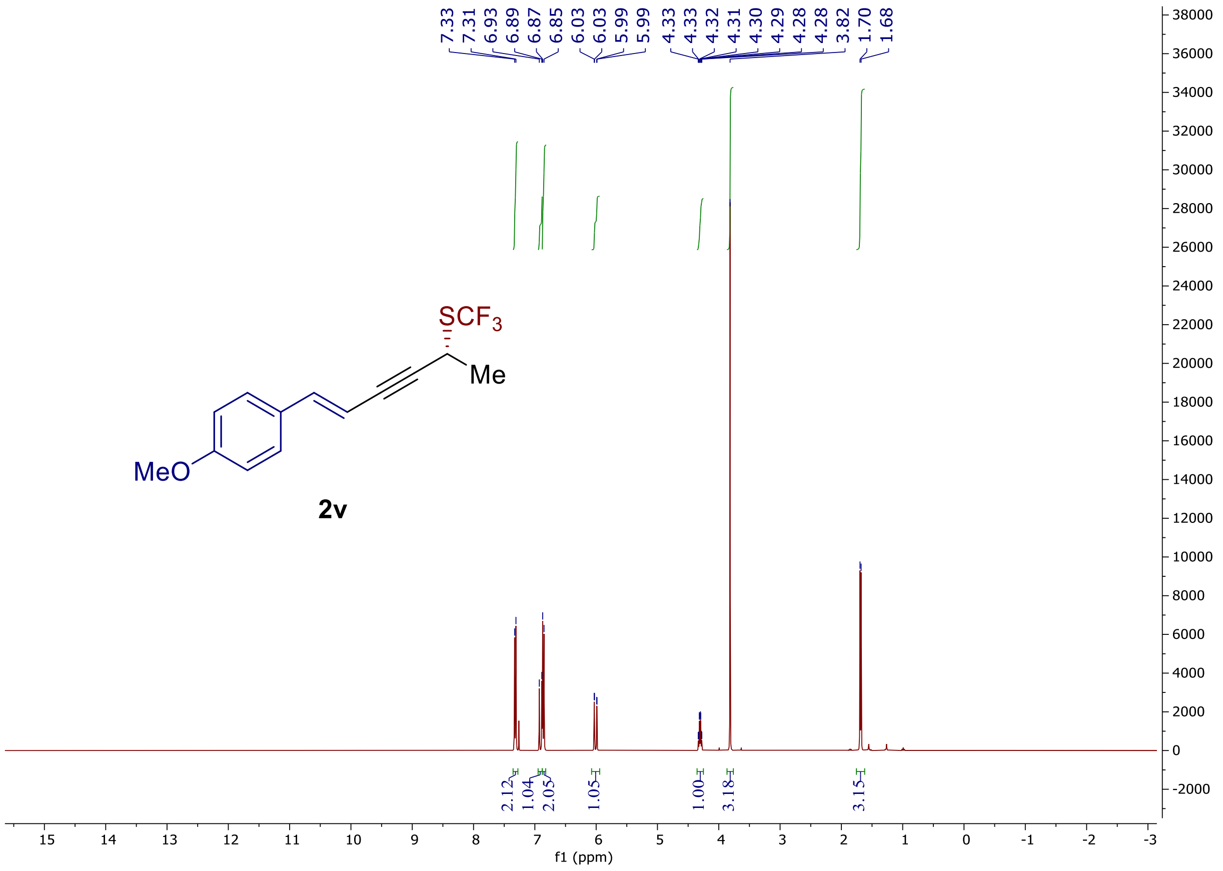

^{19}F NMR:

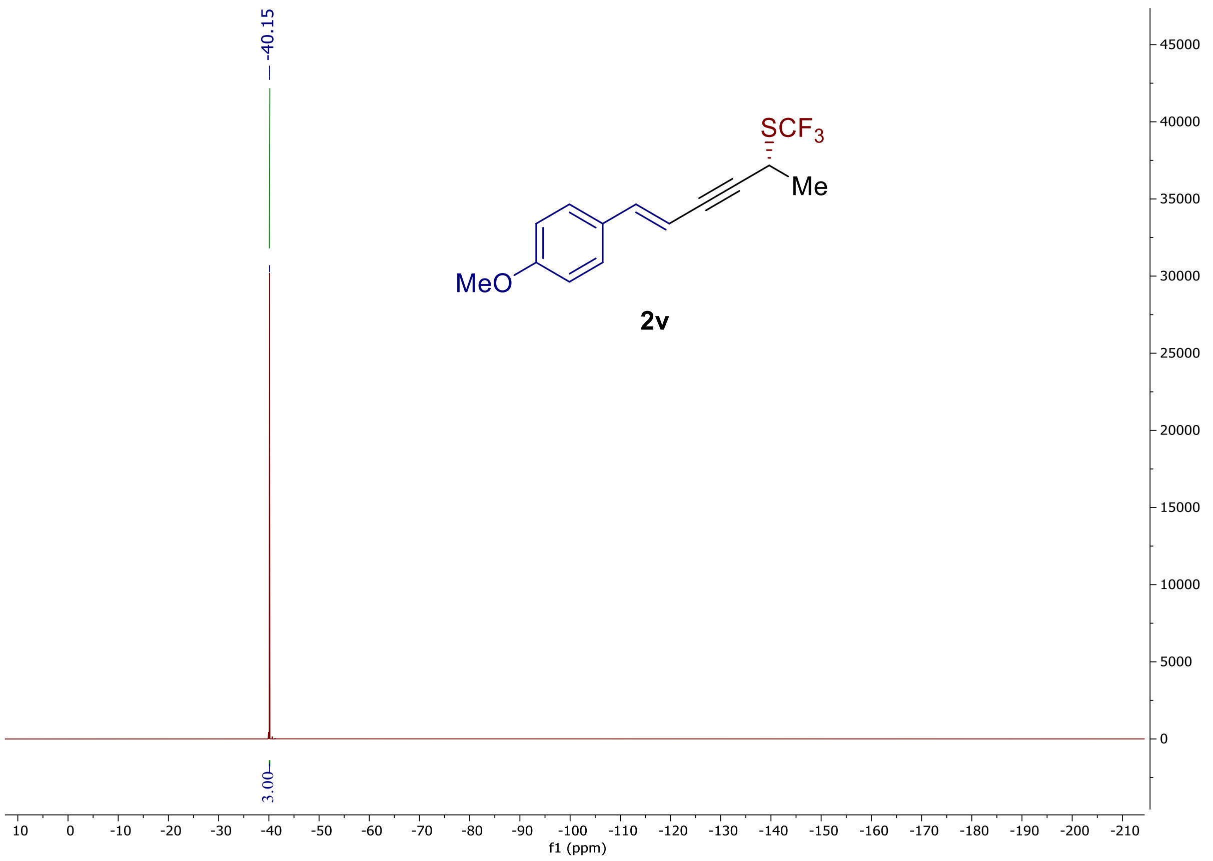

^{13}C NMR:

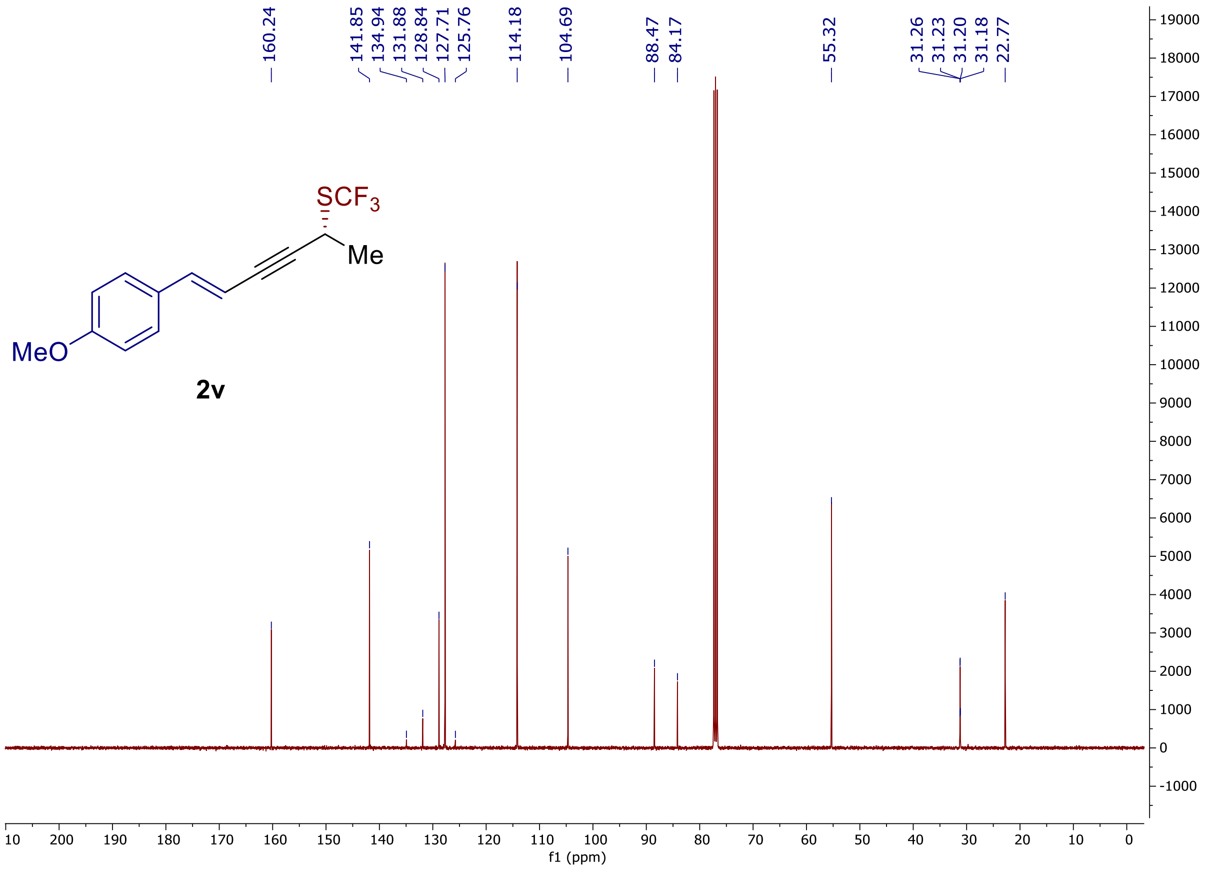
160.24
141.85
134.94
131.88
128.84
127.71
125.76
114.18
104.69
88.47
84.17
55.32
31.26
31.23
31.20
31.18
22.77
SCF3
Me
MeO
2v
f1 (ppm)

^{1}H NMR:

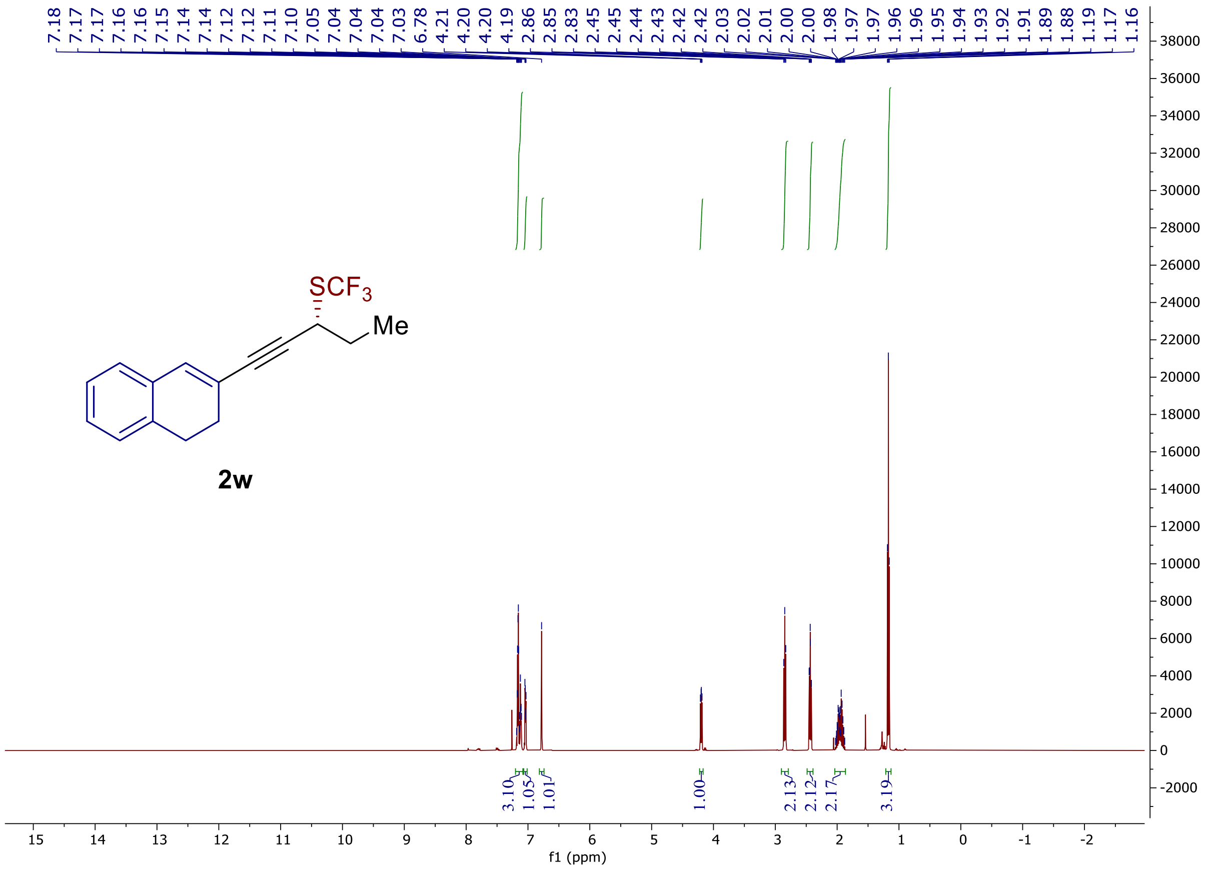

^{19}F NMR:

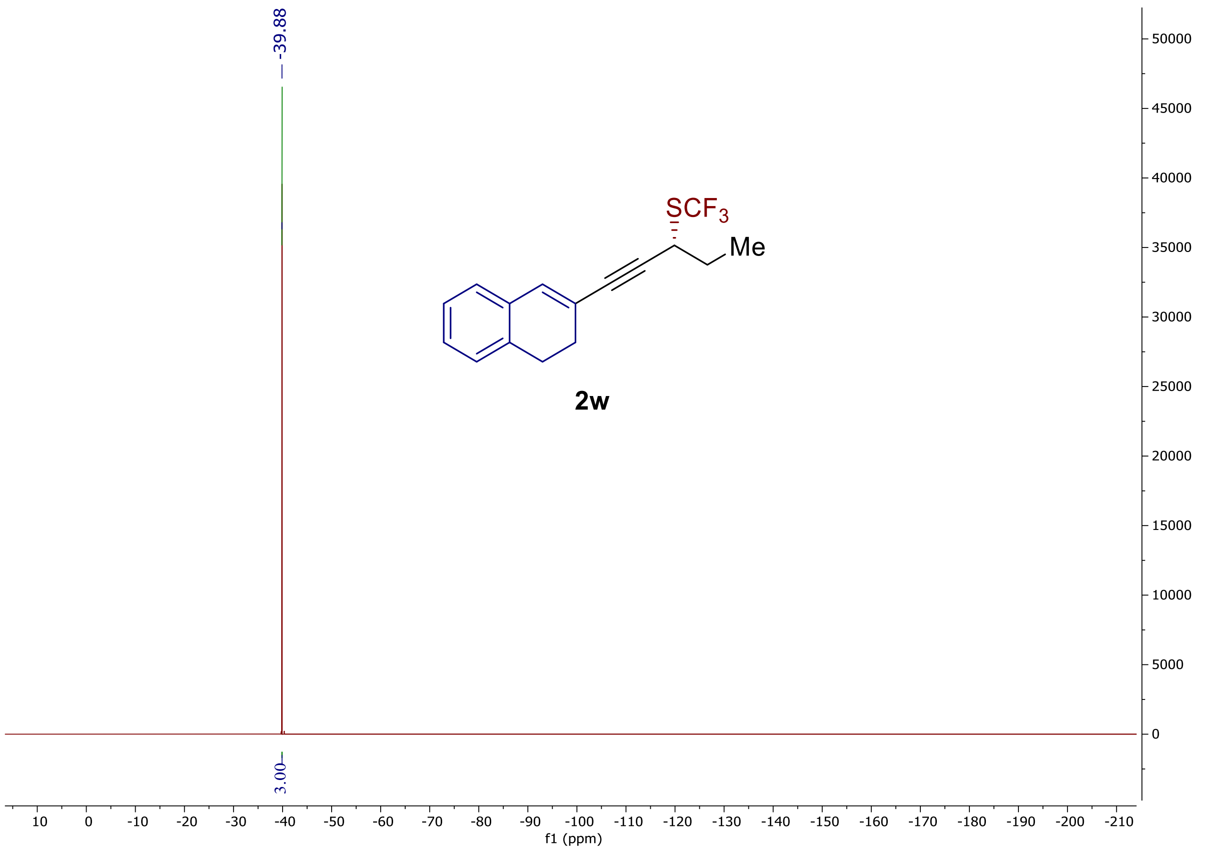

^{13}C NMR:

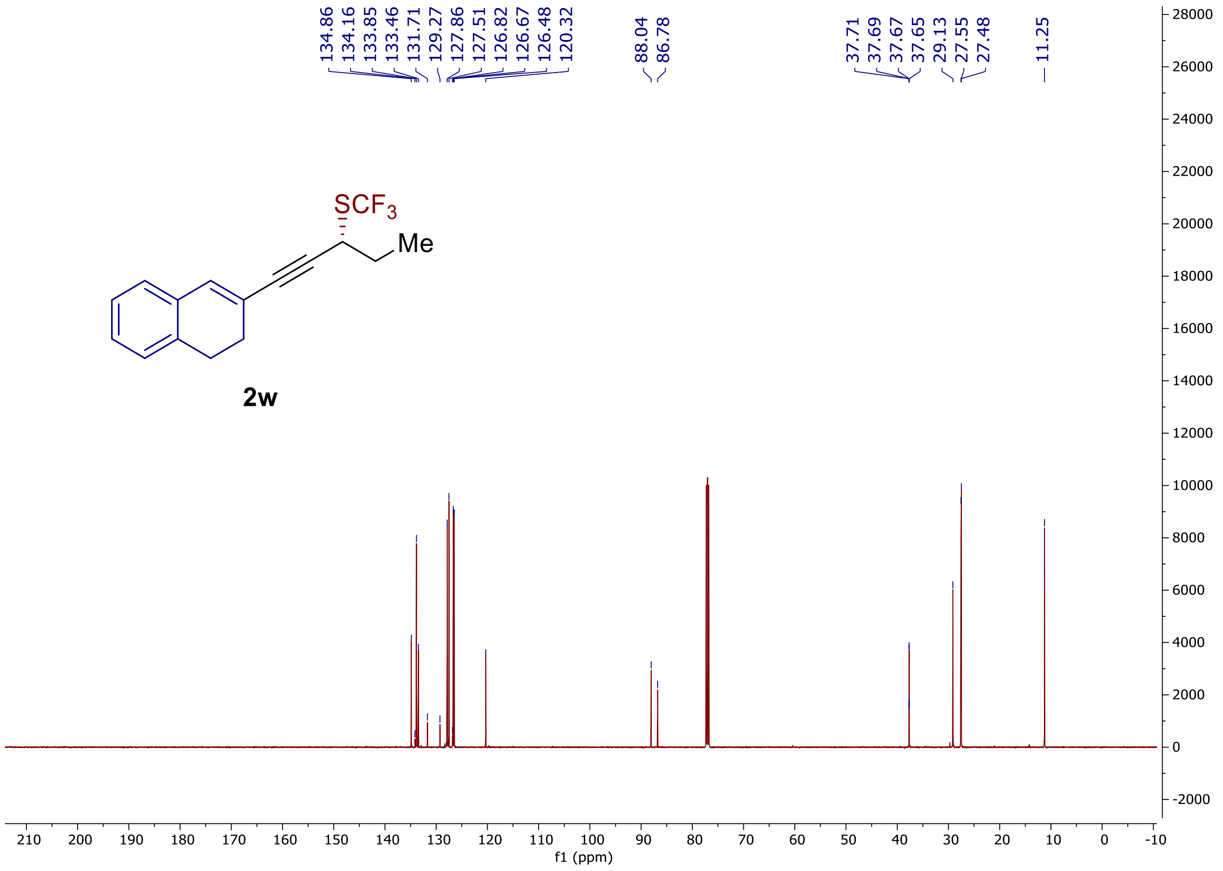
134.86
134.16
133.85
133.46
131.71
129.27
127.86
127.51
126.82
126.67
126.48
120.32
88.04
86.78
37.71
37.69
37.67
37.65
29.13
27.55
27.48
11.25
SCF3
Me
2w
f1 (ppm)

^{1}H NMR:

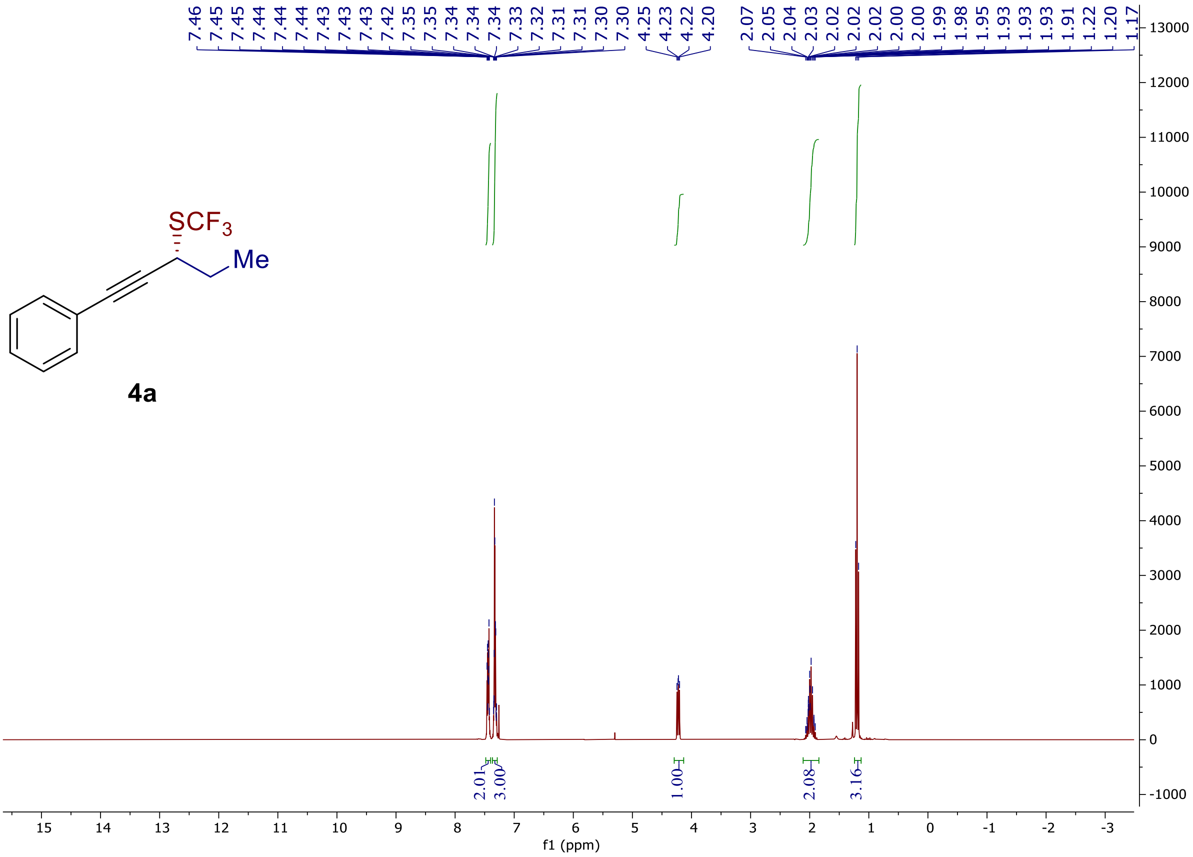

4a

^{19}F NMR:

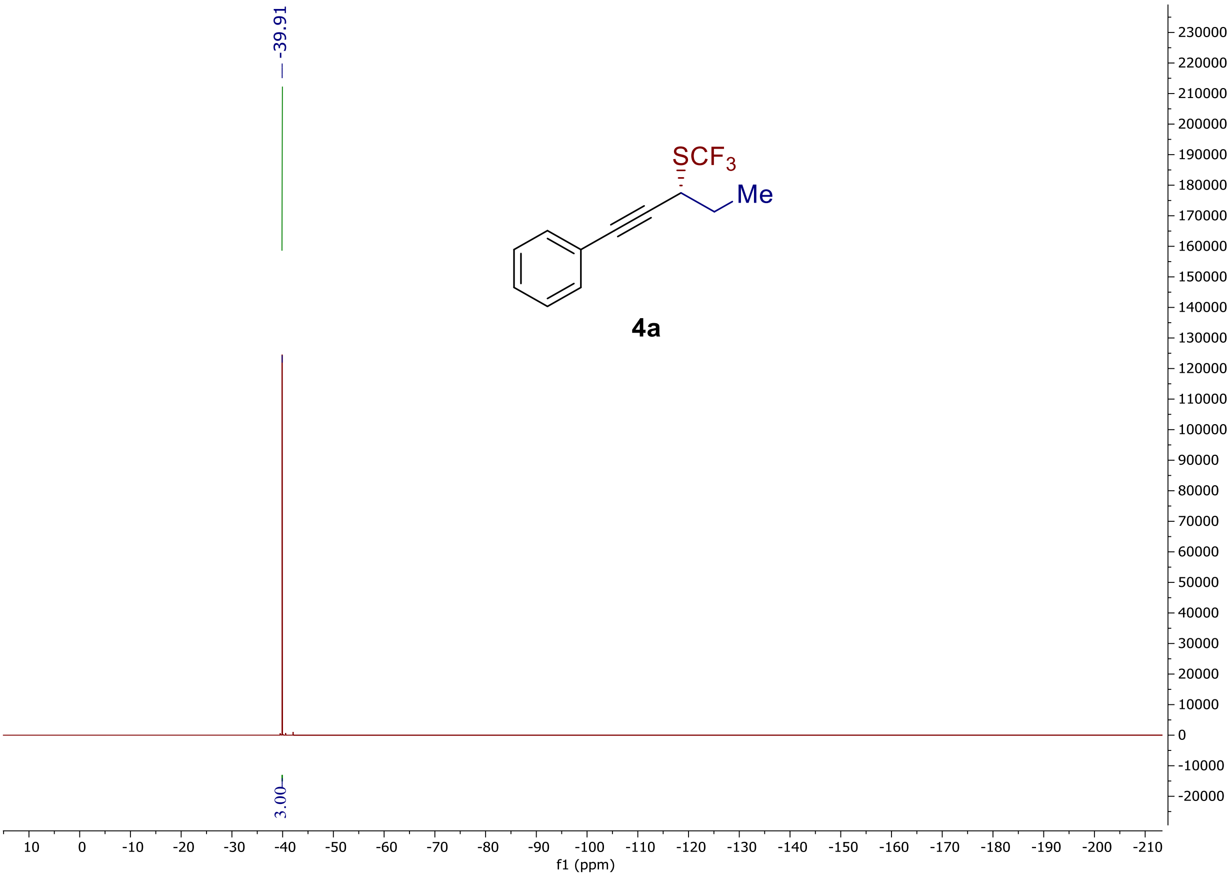

4a

^{13}C NMR:

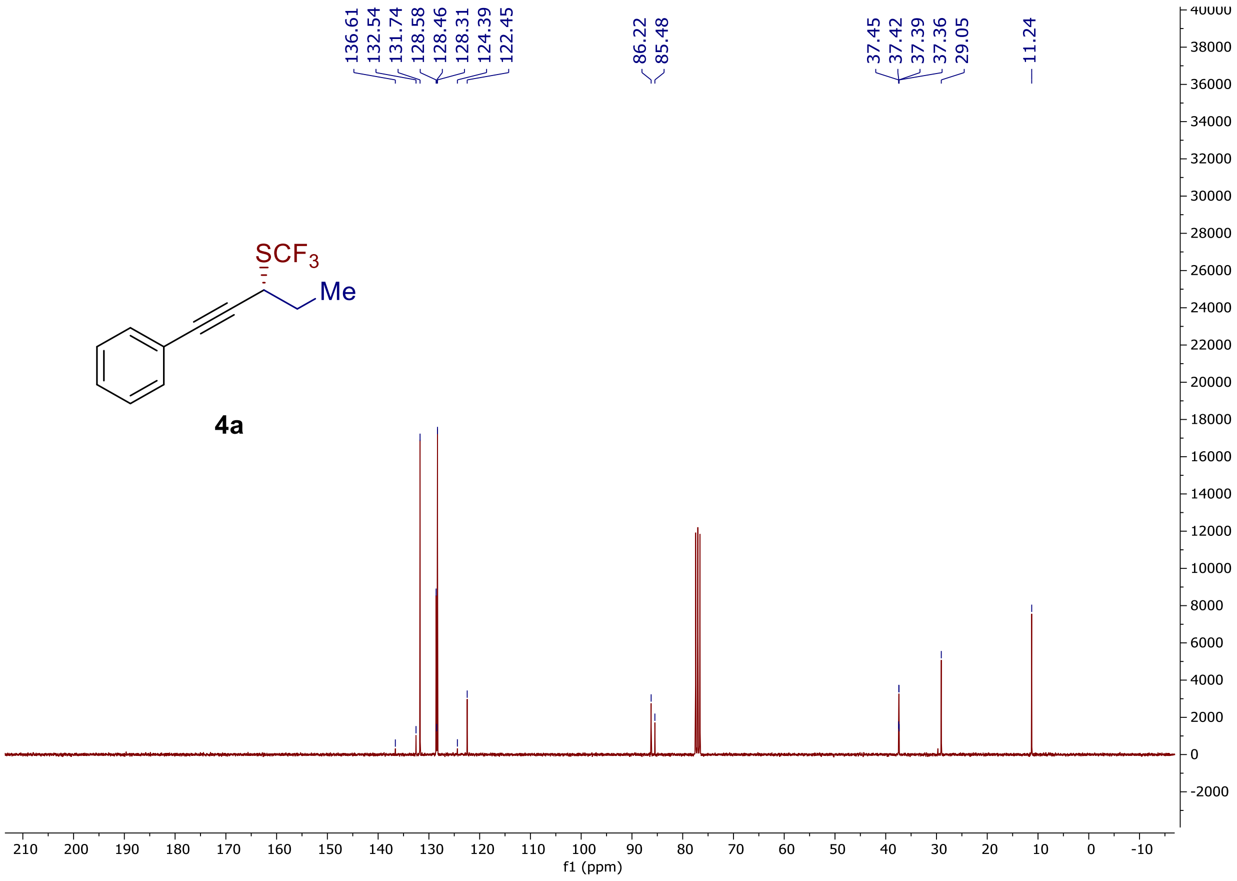

136.61
132.54
131.74
128.58
128.46
128.31
124.39
122.45
86.22
85.48
37.45
37.42
37.39
37.36
29.05
11.24
SCF3
Me
4a
f1 (ppm)

^{1}H NMR:

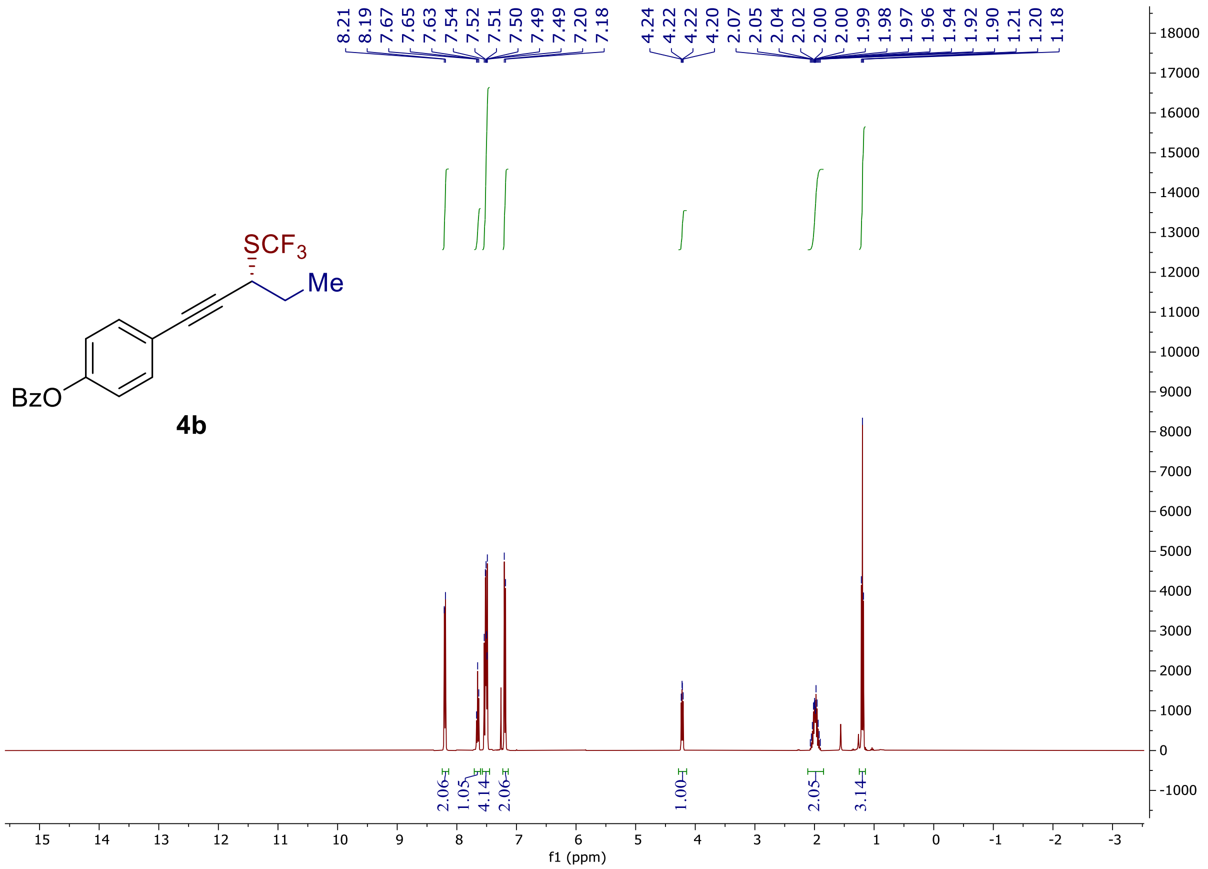

^{19}F NMR:

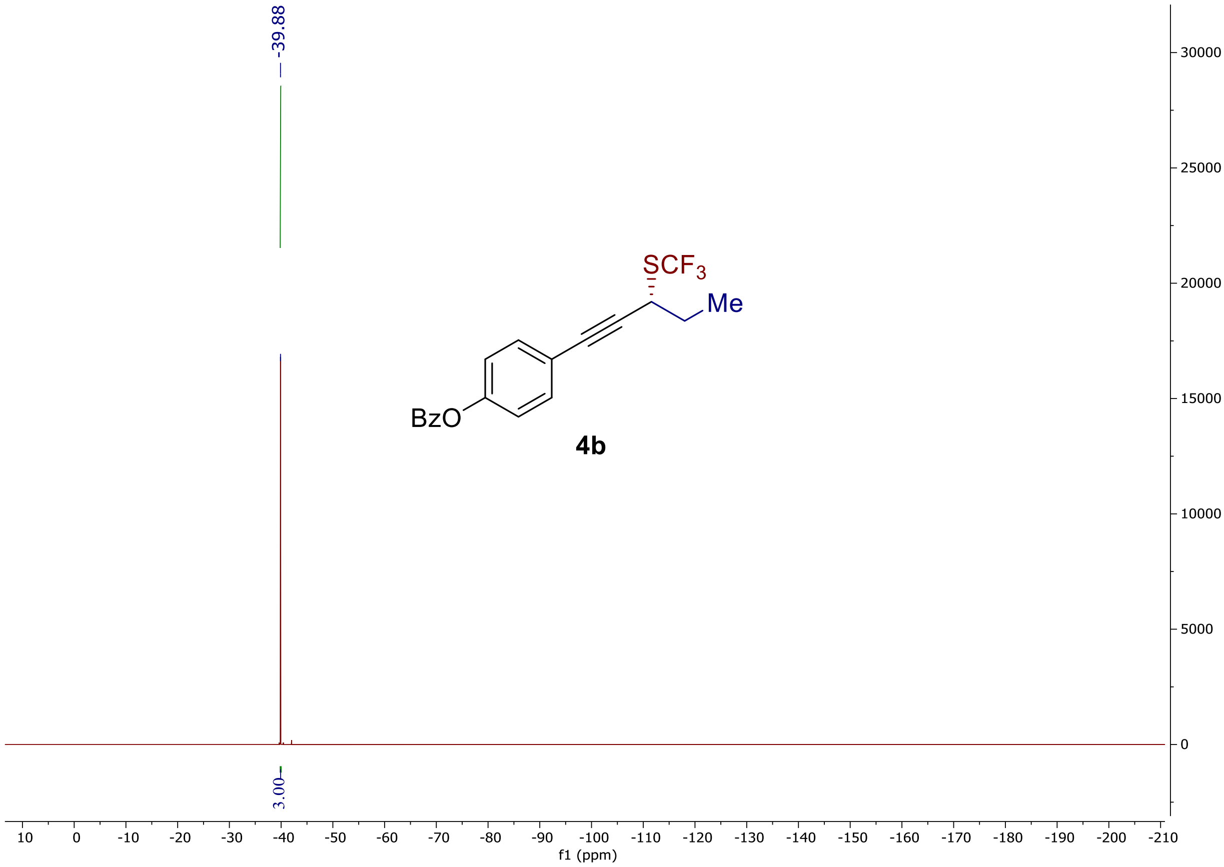

^{13}C NMR:

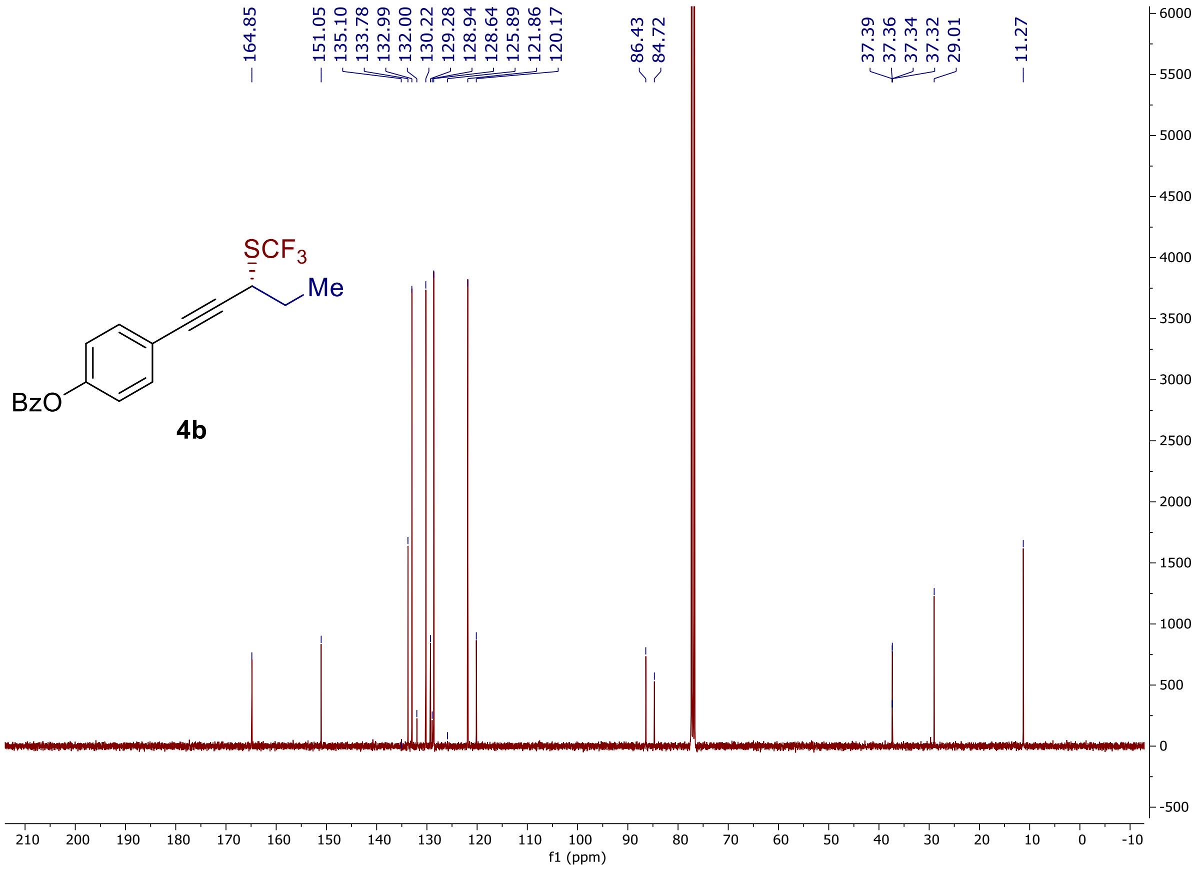
164.85
151.05
135.10
133.78
132.99
132.00
130.22
129.28
128.94
128.64
125.89
121.86
120.17
86.43
84.72
37.39
37.36
37.34
37.32
29.01
11.27
SCF3
Me
BzO
4b
f1 (ppm)

^{1}H NMR:

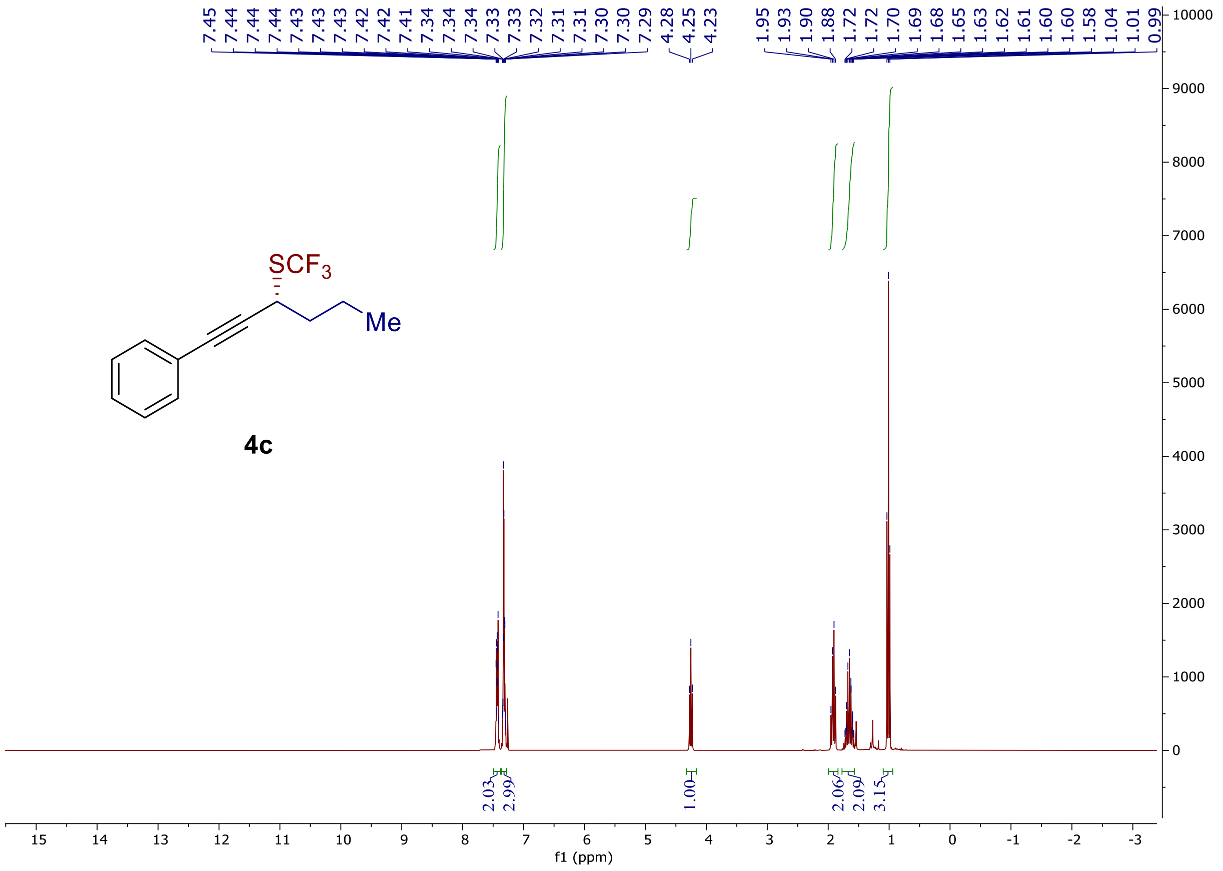

^{19}F NMR:

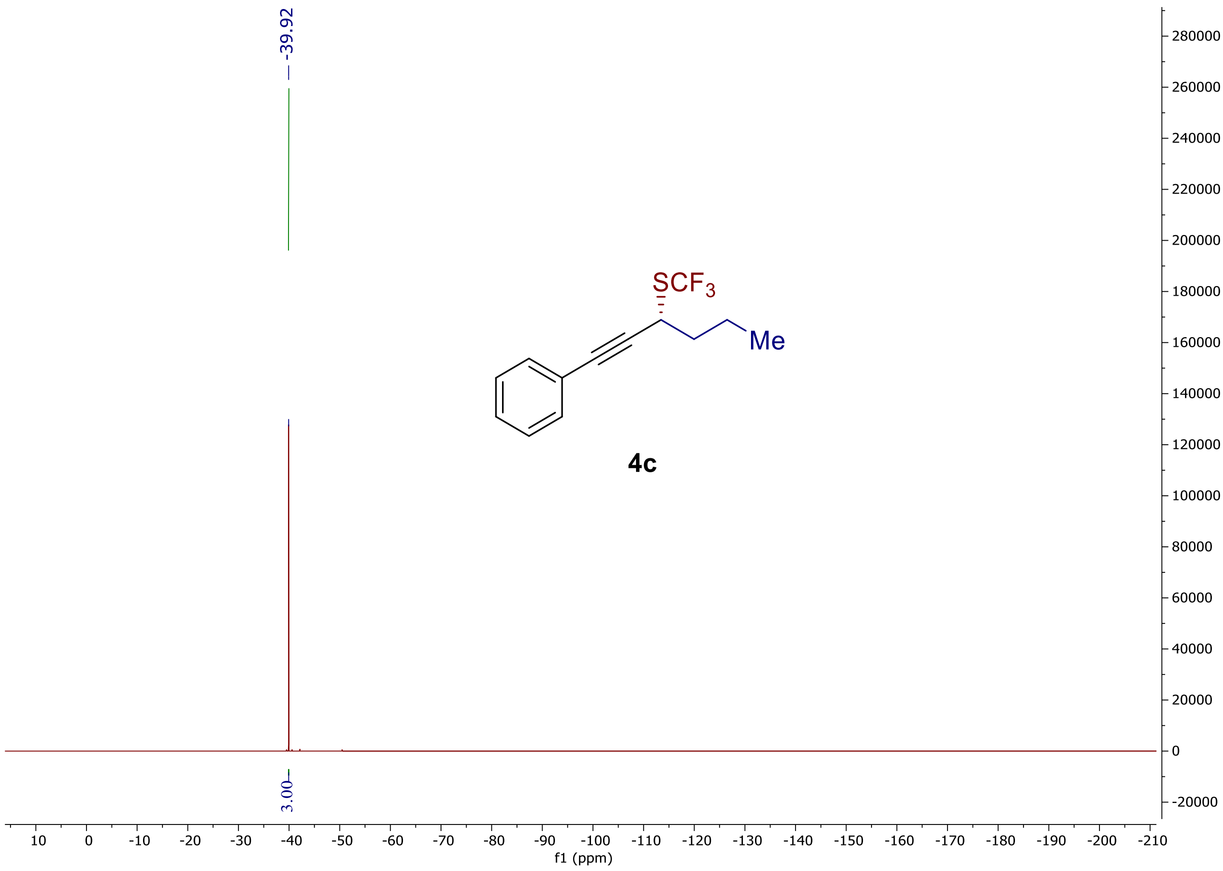

^{13}C NMR:

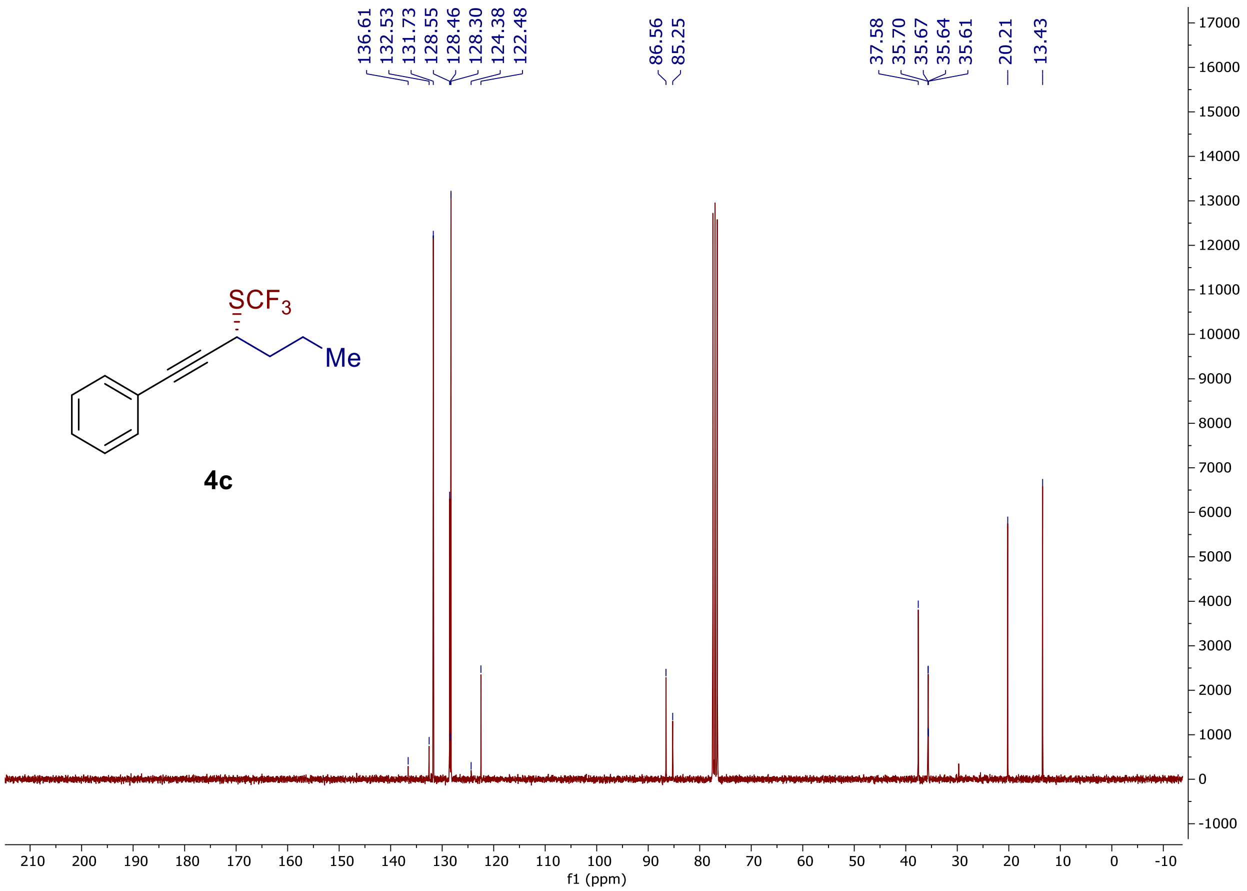
136.61
132.53
131.73
128.55
128.46
128.30
124.38
122.48
86.56
85.25
37.58
35.70
35.67
35.64
35.61
20.21
13.43
SCF3
Me
4c
f1 (ppm)

^{1}H NMR:

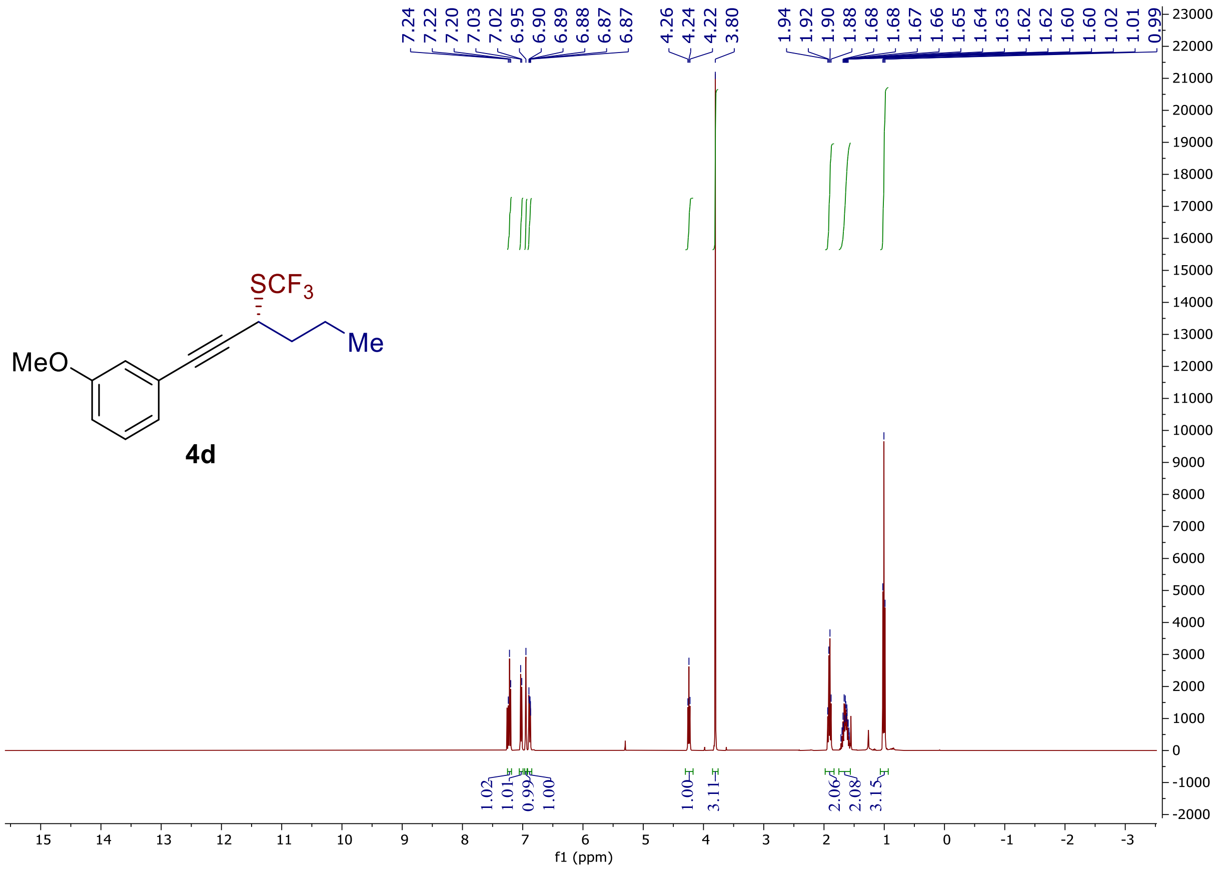

^{19}F NMR:

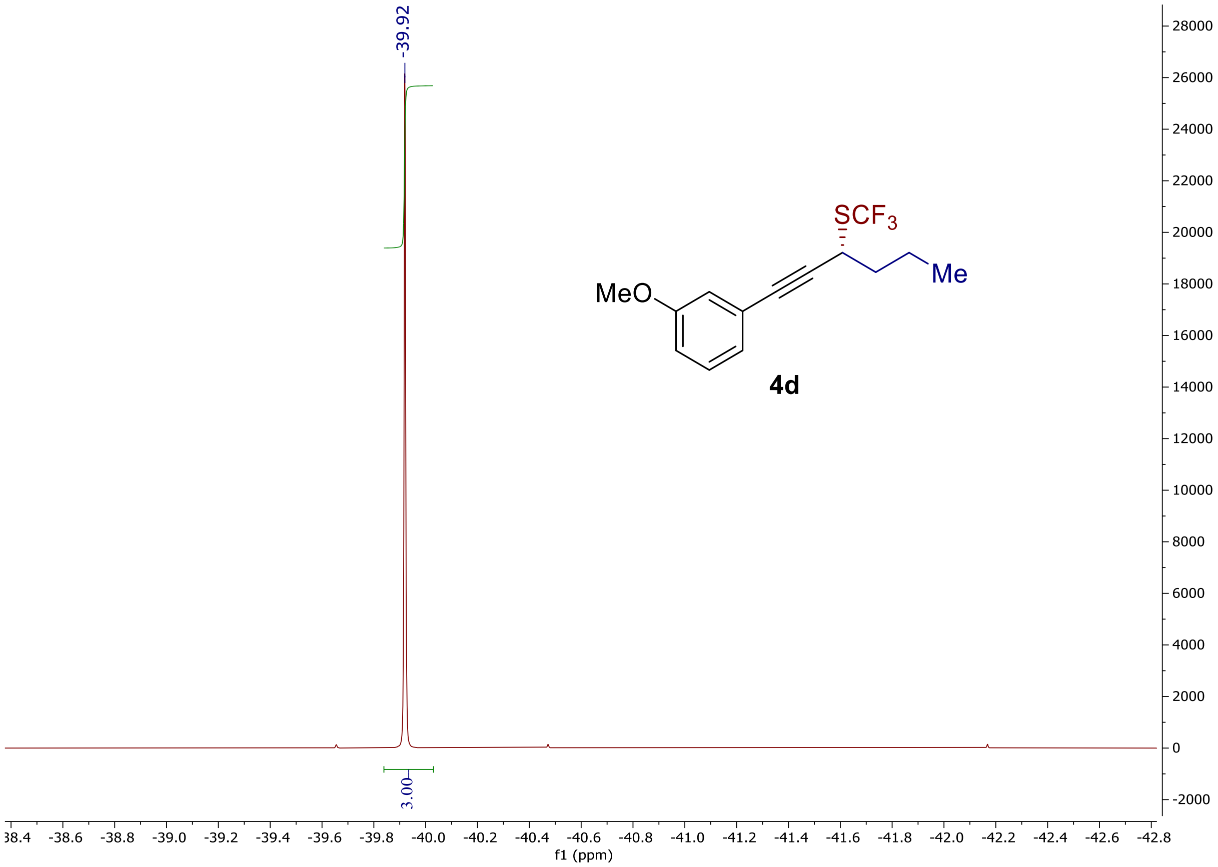

^{13}C NMR:

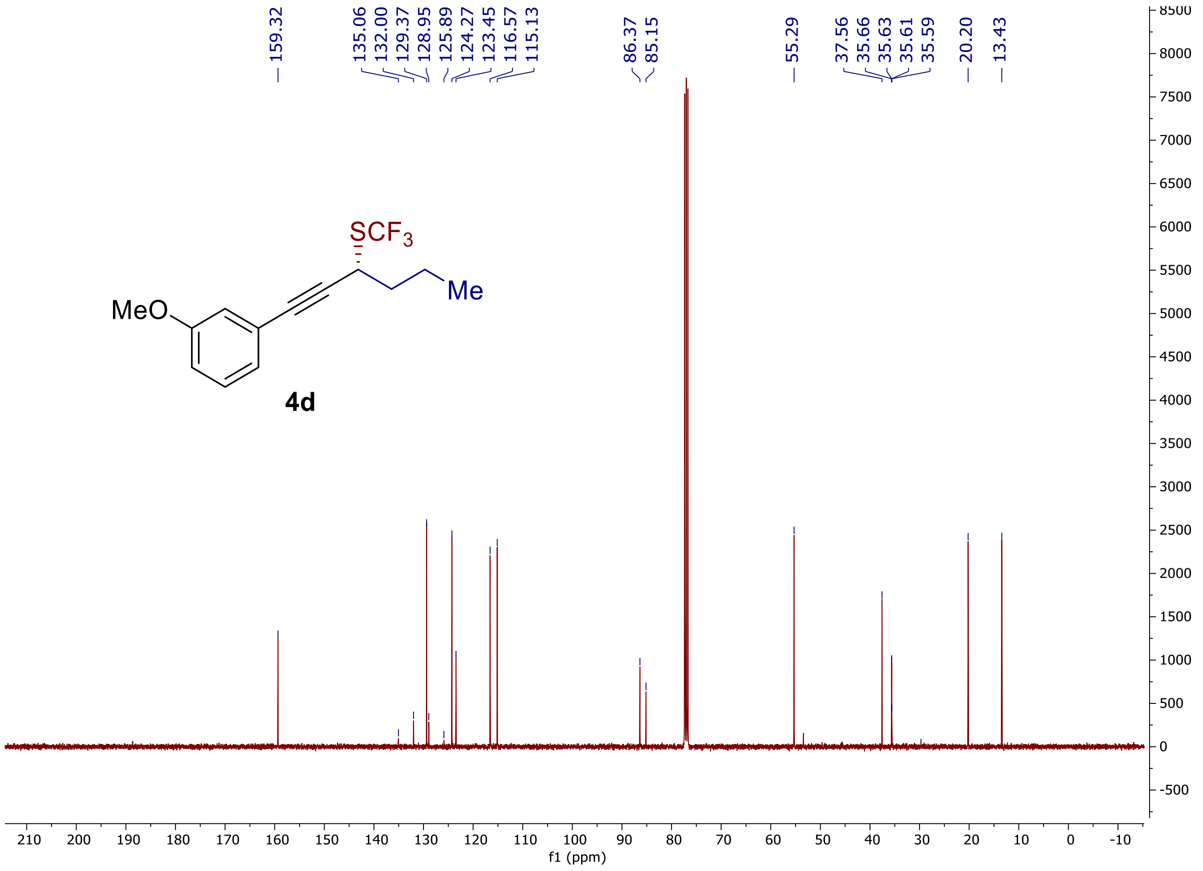
159.32
135.06
132.00
129.37
128.95
125.89
124.27
123.45
116.57
115.13
86.37
85.15
55.29
37.56
35.66
35.63
35.61
35.59
20.20
13.43
SCF3
MeO
Me
4d
f1 (ppm)

^{1}H NMR:

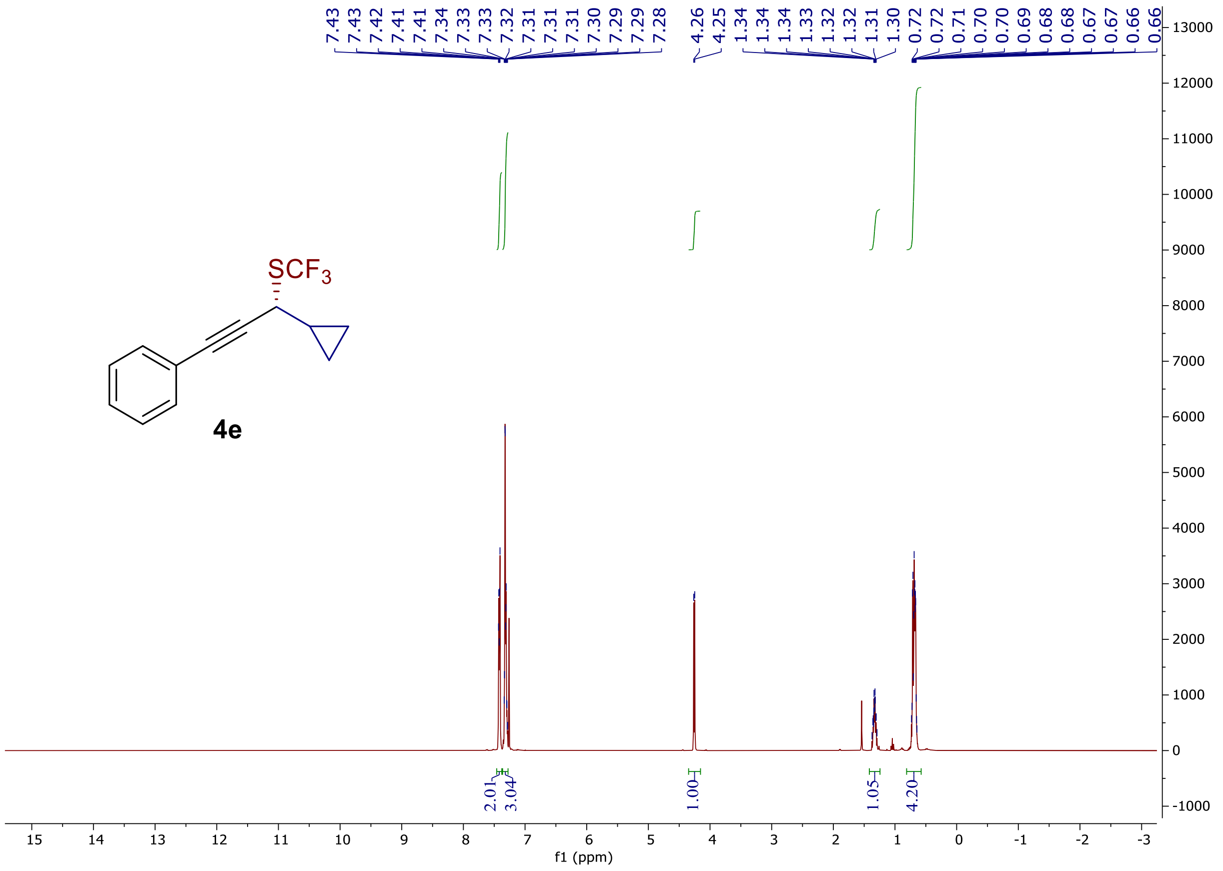

^{19}F NMR:

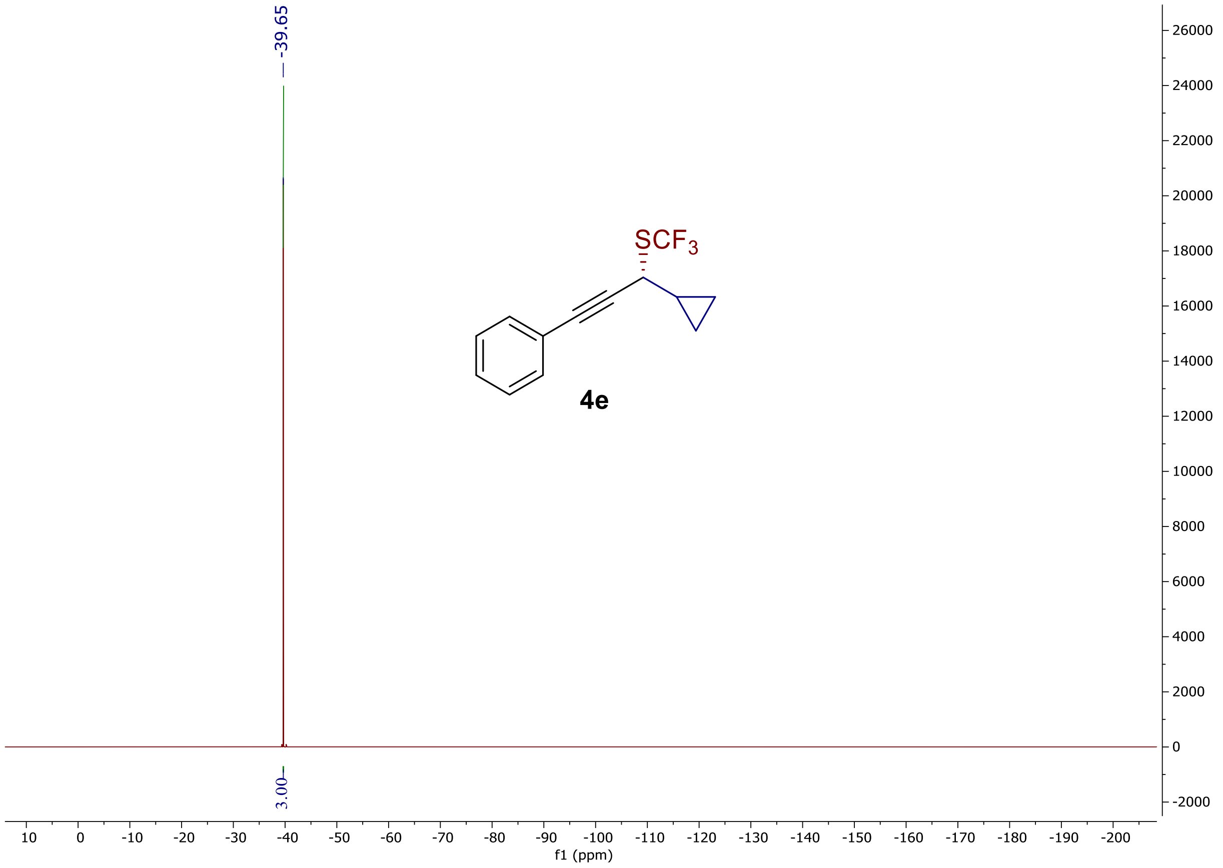

^{13}C NMR:

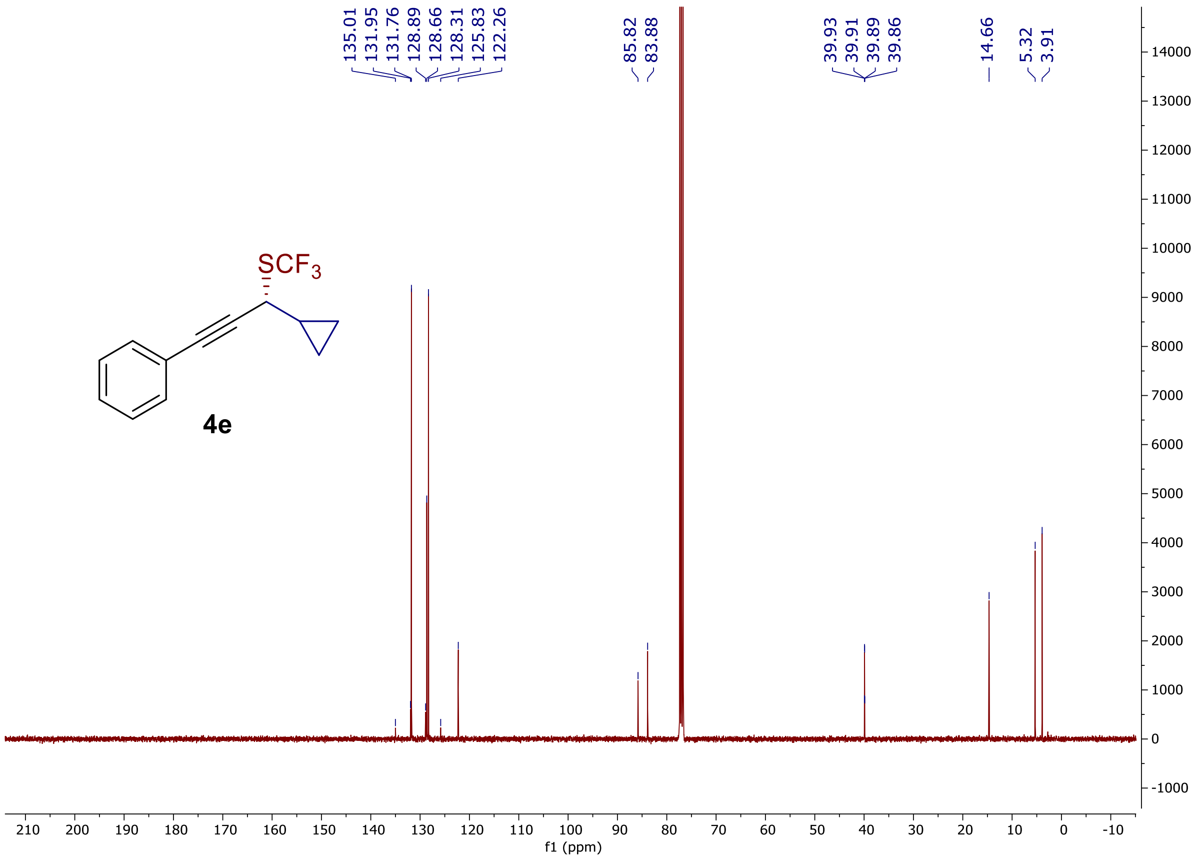

135.01
131.95
131.76
128.89
128.66
128.31
125.83
122.26
85.82
83.88
39.93
39.91
39.89
39.86
14.66
5.32
3.91
SCF3
4e
f1 (ppm)

^{1}H NMR:

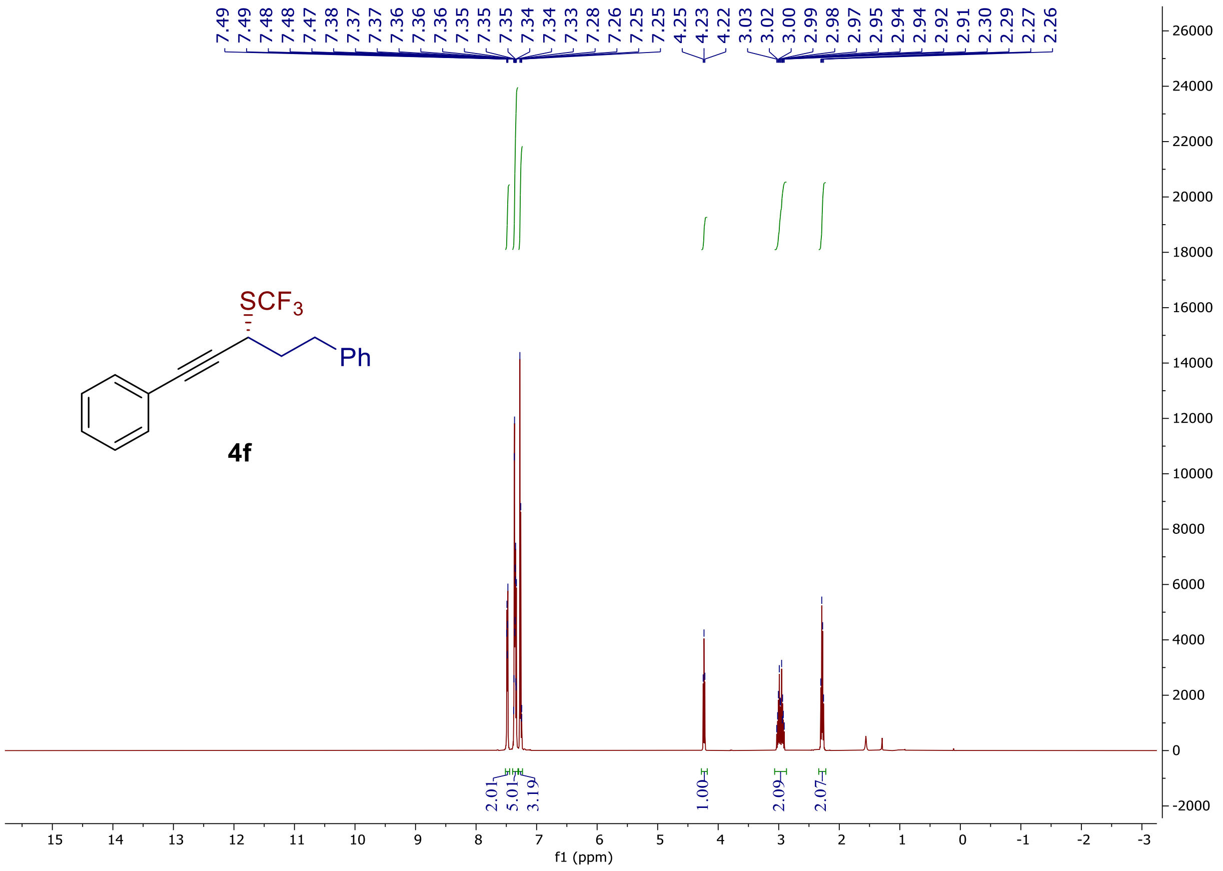

^{19}F NMR:

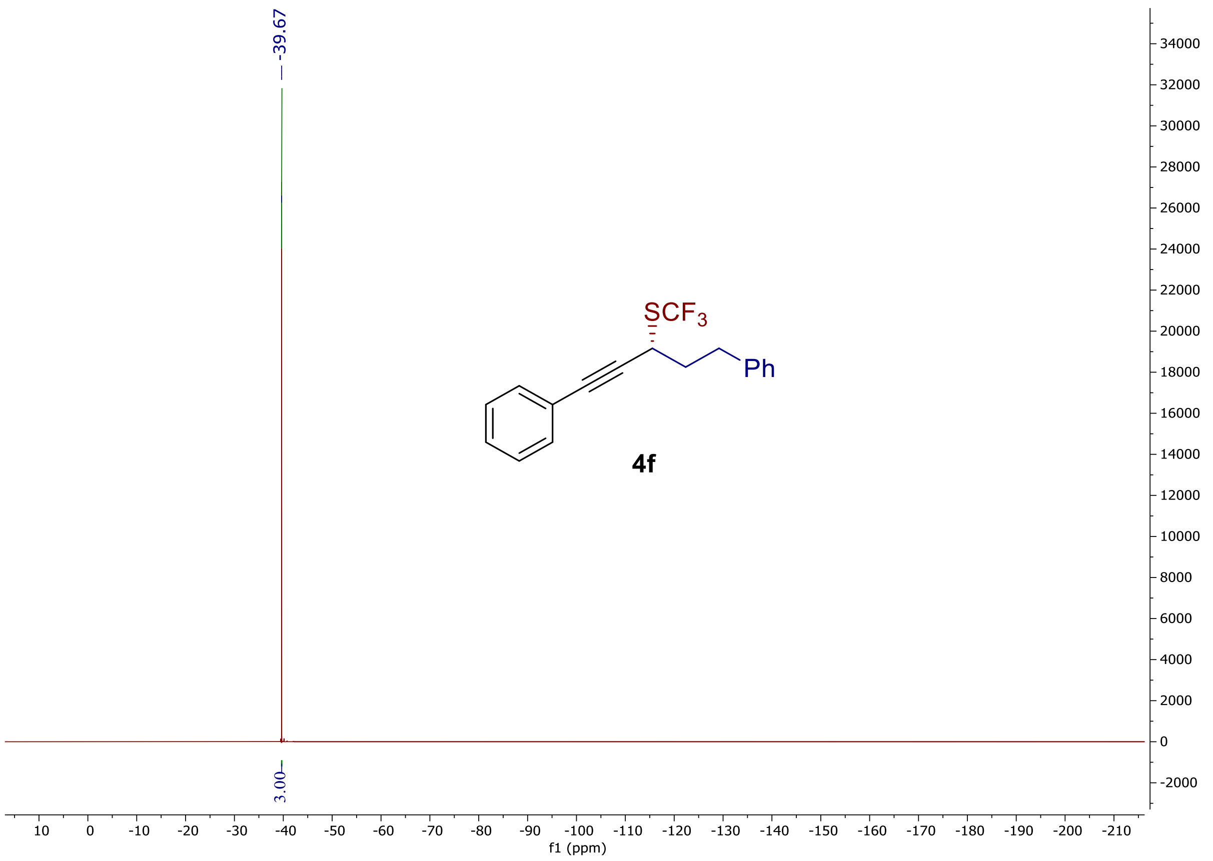

^{13}C NMR:

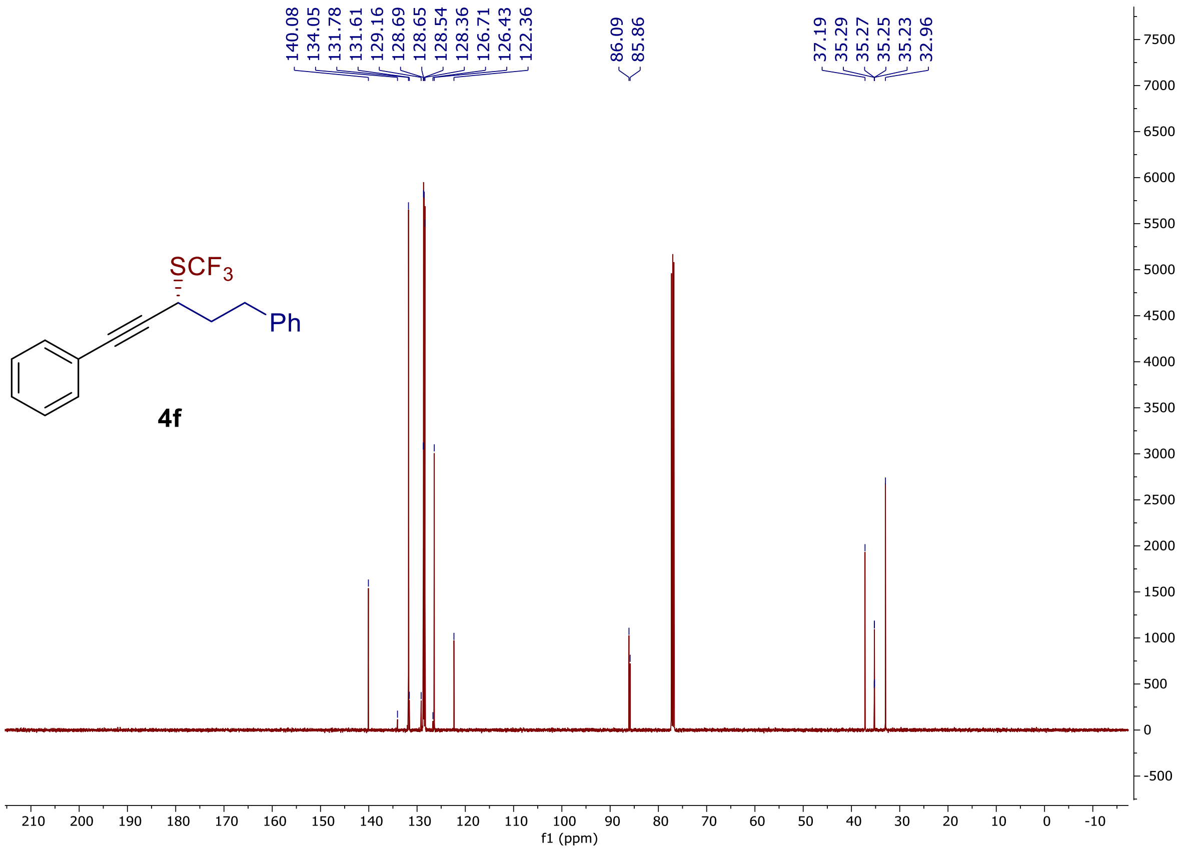
140.08
134.05
131.78
131.61
129.16
128.69
128.65
128.54
128.36
126.71
126.43
122.36
86.09
85.86
37.19
35.29
35.27
35.25
35.23
32.96
SCF3
Ph
4f
f1 (ppm)

^{1}H NMR:

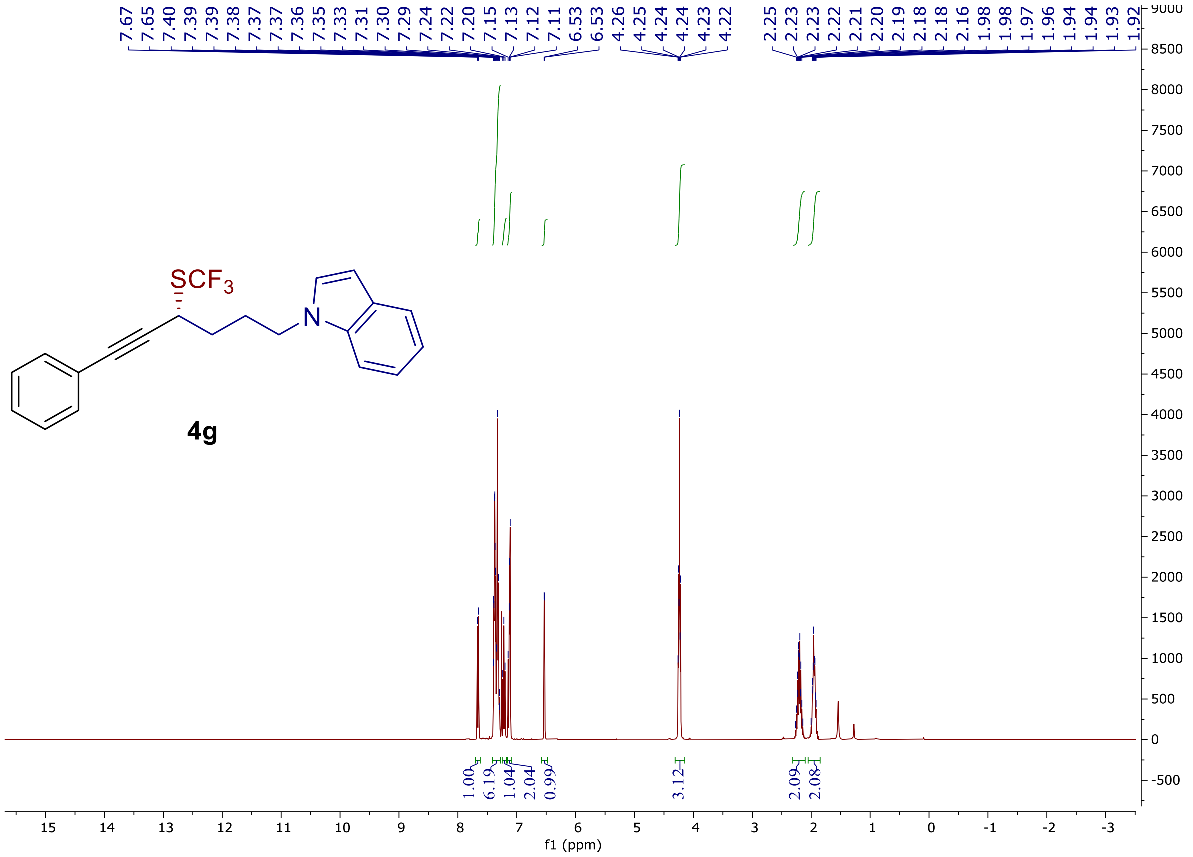

^{19}F NMR:

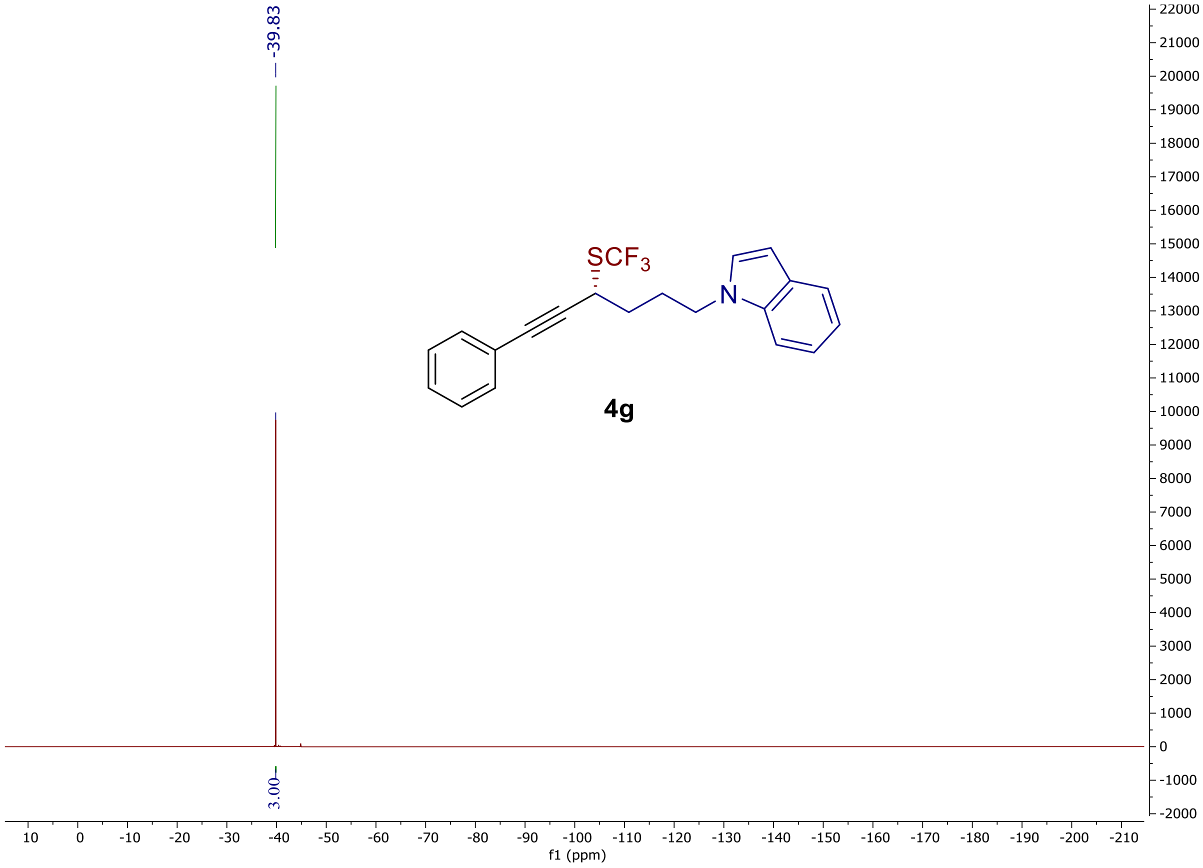

^{13}C NMR:

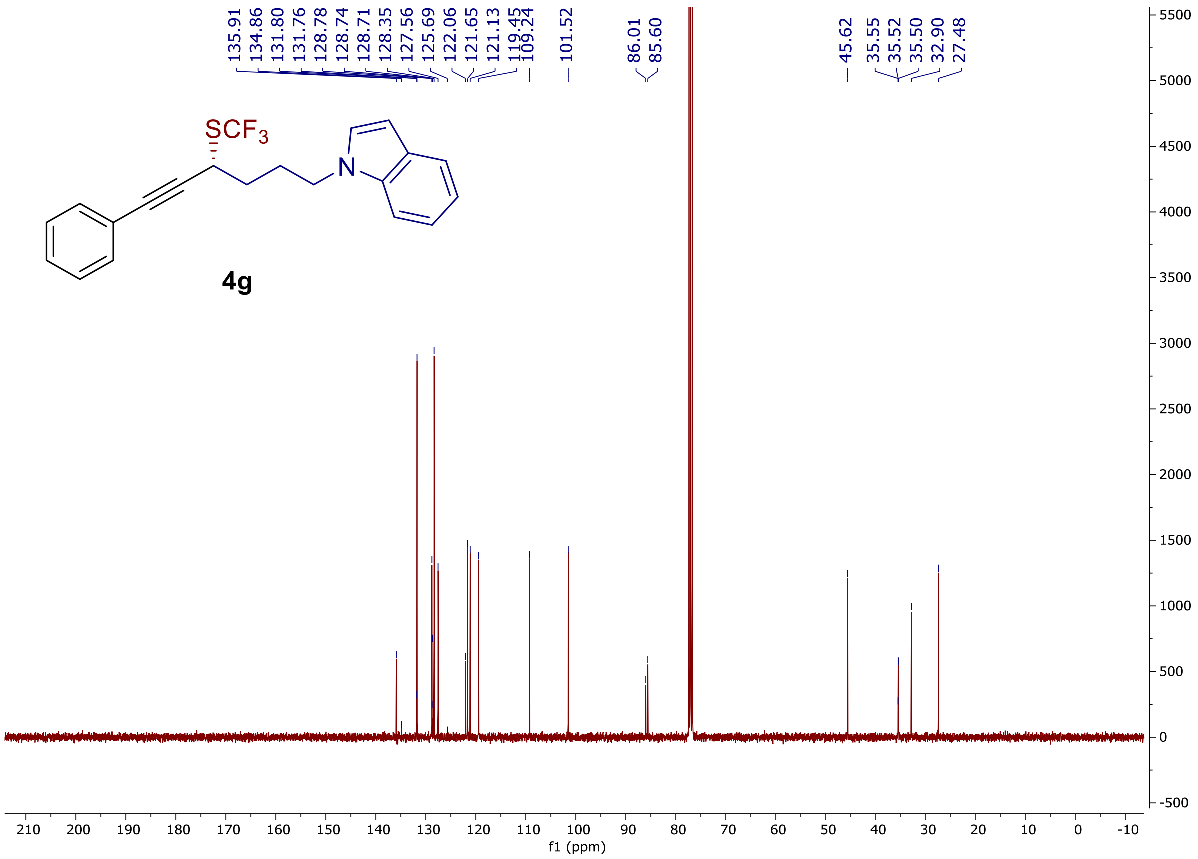

135.91
134.86
131.80
131.76
128.78
128.74
128.71
128.35
127.56
125.69
122.06
121.65
121.13
119.45
109.24
101.52
86.01
85.60
45.62
35.55
35.52
35.50
32.90
27.48
SCF3
N
4g
f1 (ppm)

^{1}H NMR:

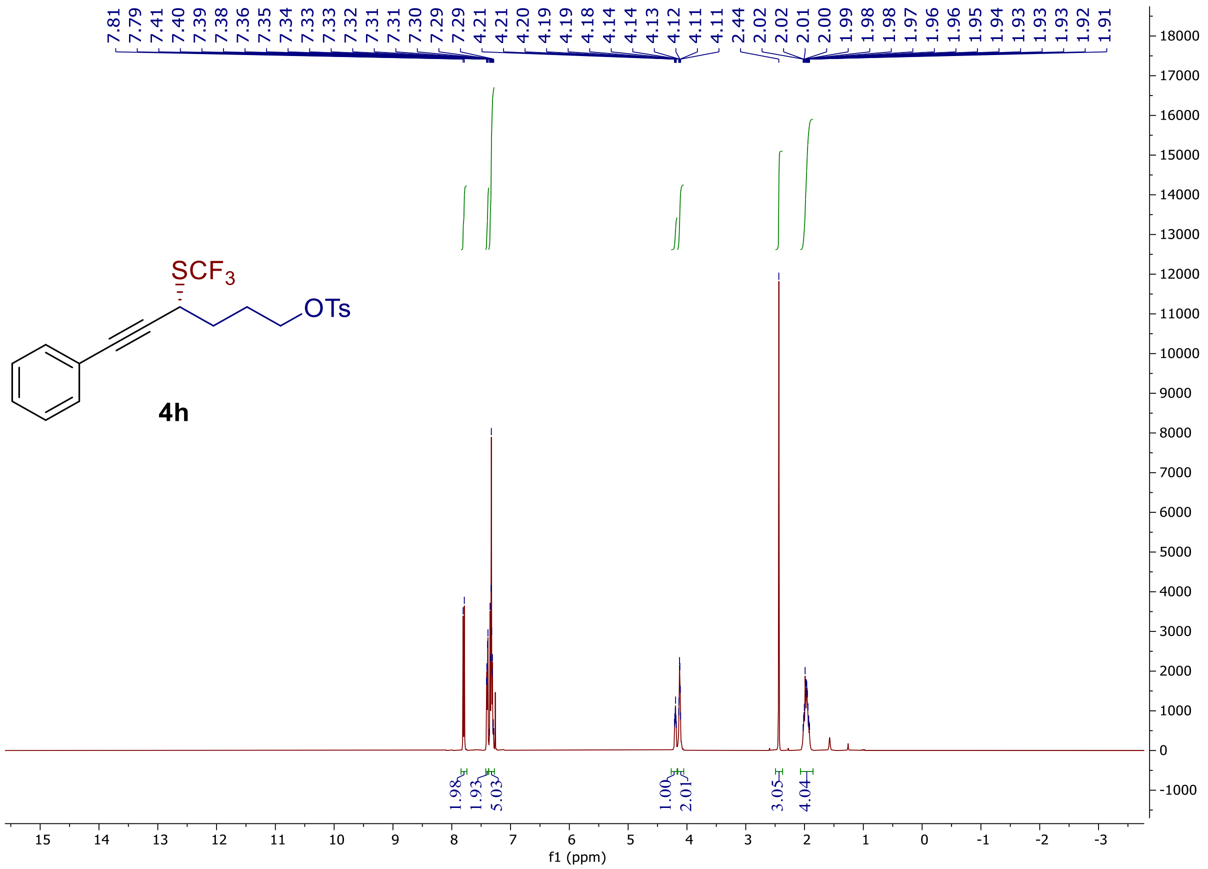

^{19}F NMR:

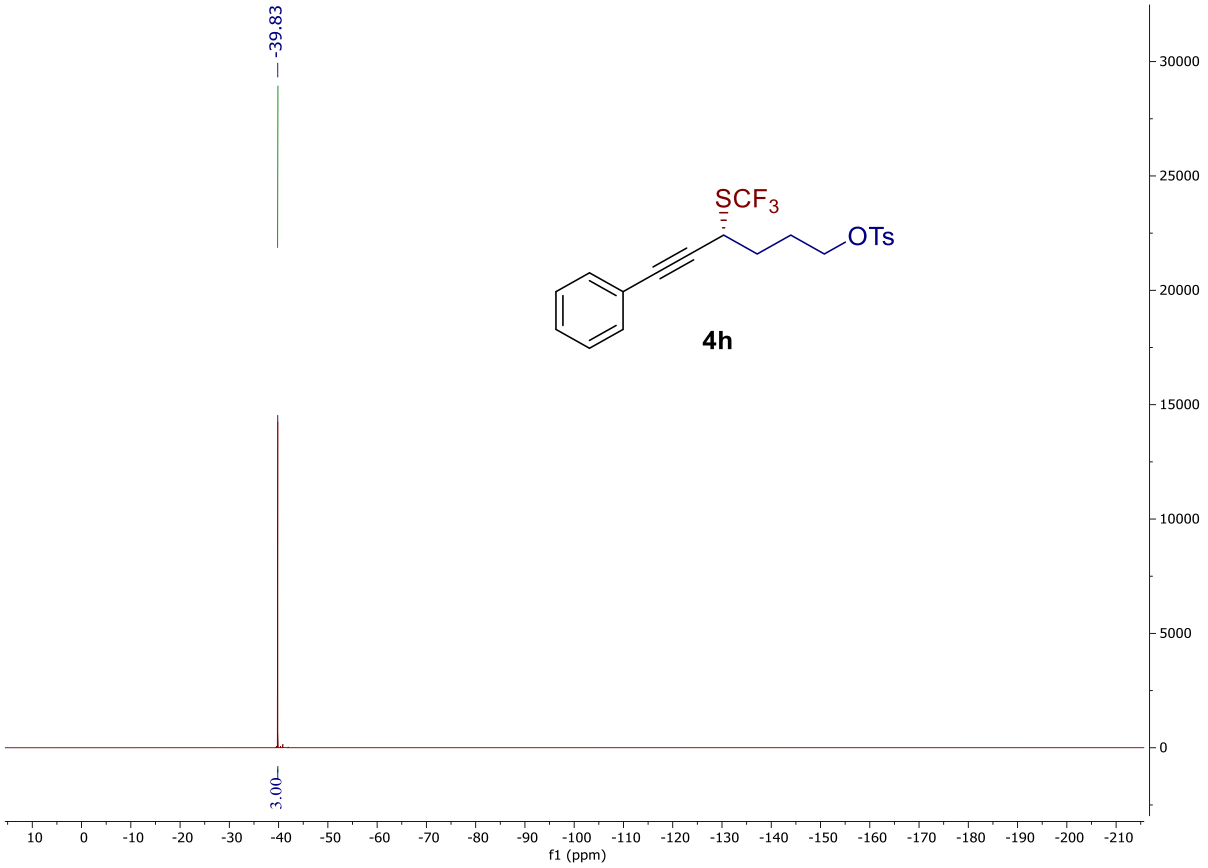

^{13}C NMR:

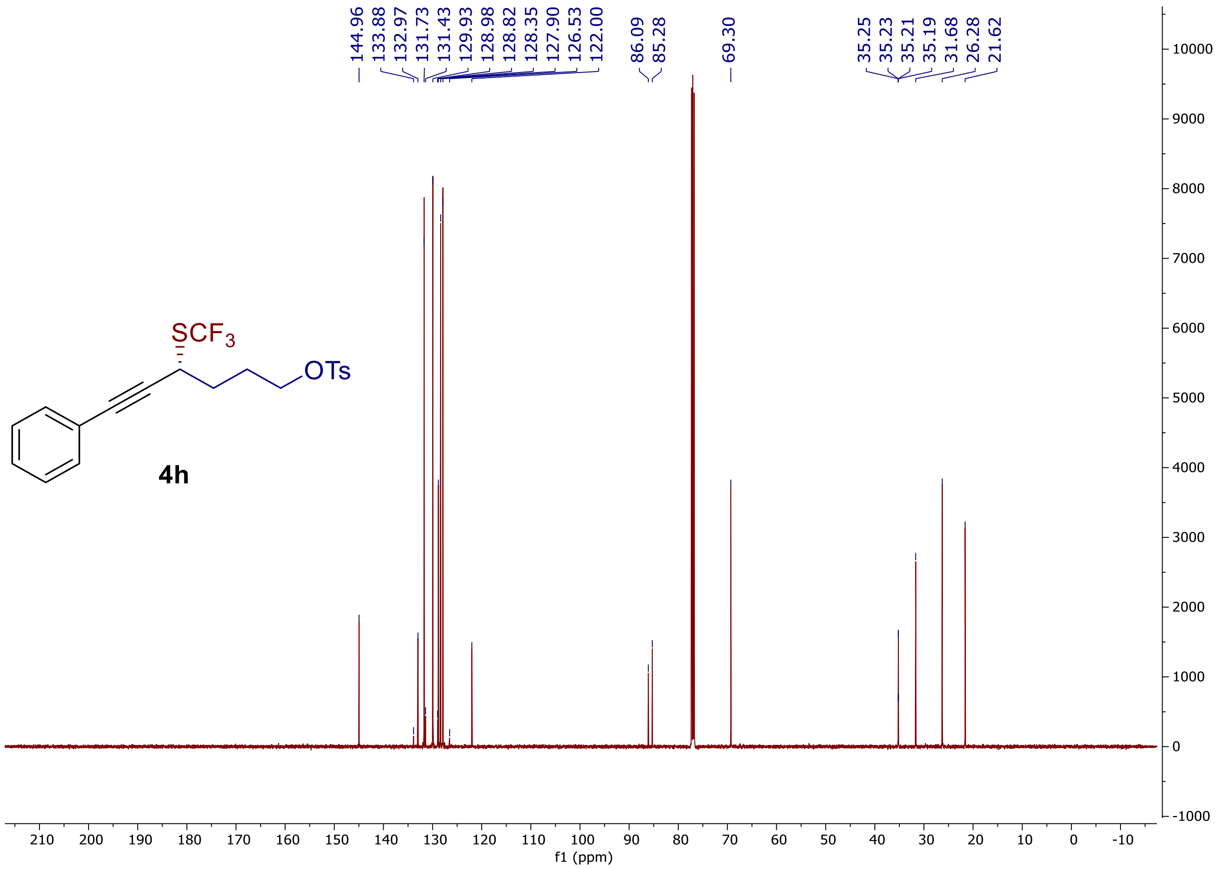
144.96
133.88
132.97
131.73
131.43
129.93
128.98
128.82
128.35
127.90
126.53
122.00
86.09
85.28
69.30
35.25
35.23
35.21
35.19
31.68
26.28
21.62
SCF3
OTs
4h
f1 (ppm)

^{1}H NMR:

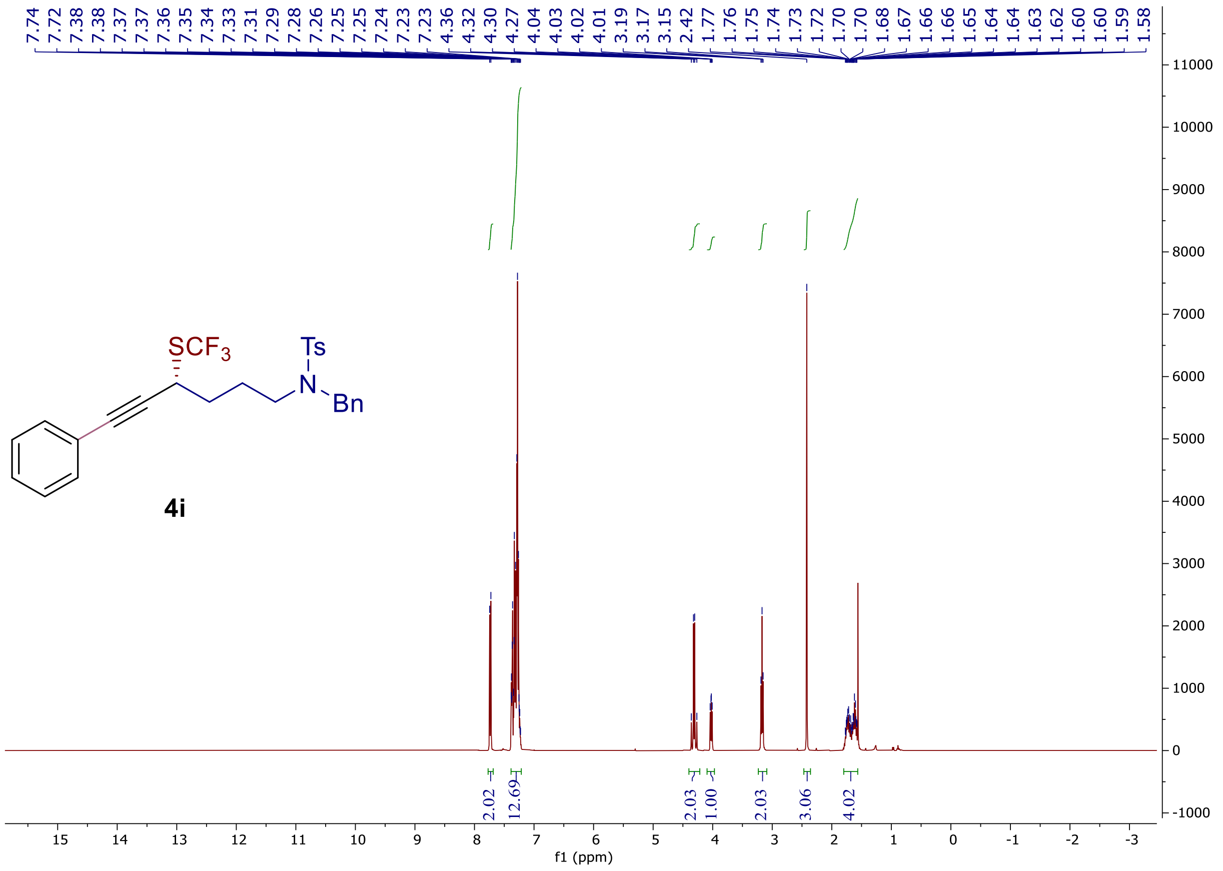

^{19}F NMR:

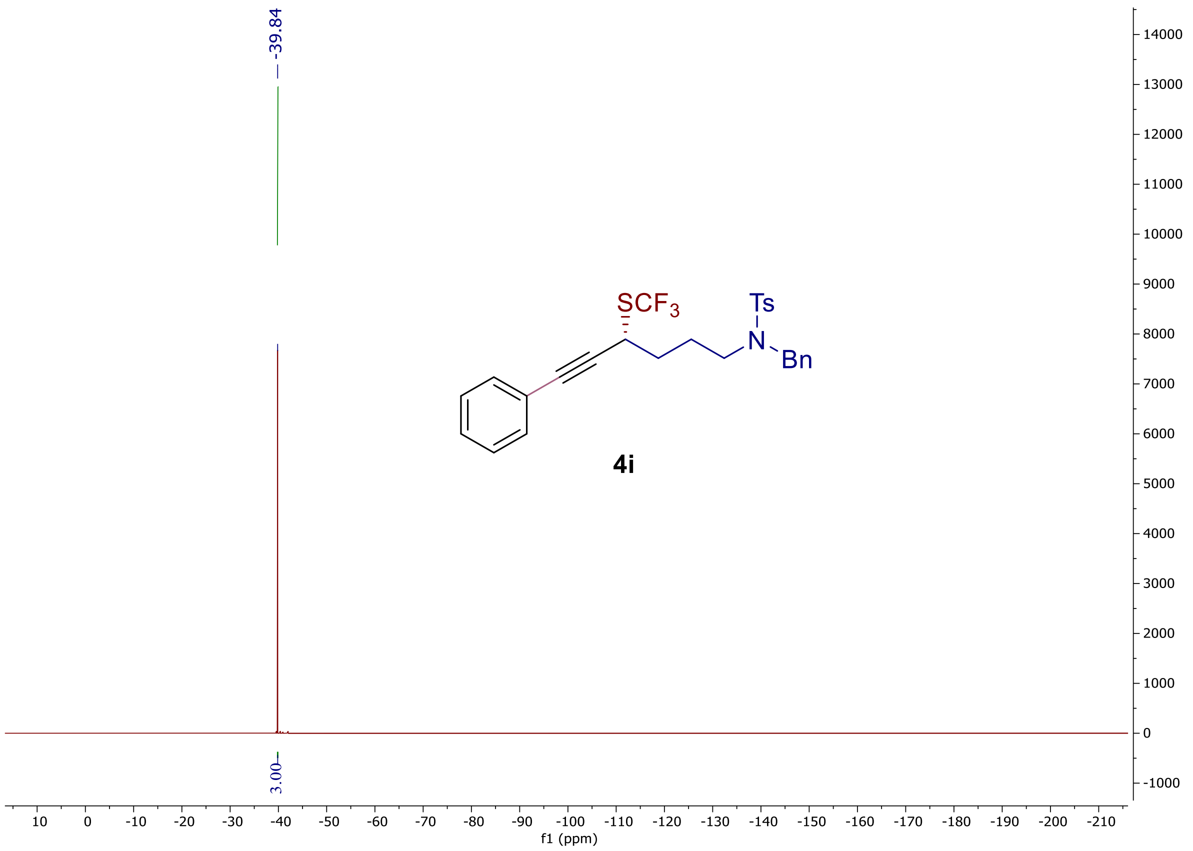

^{13}C NMR:

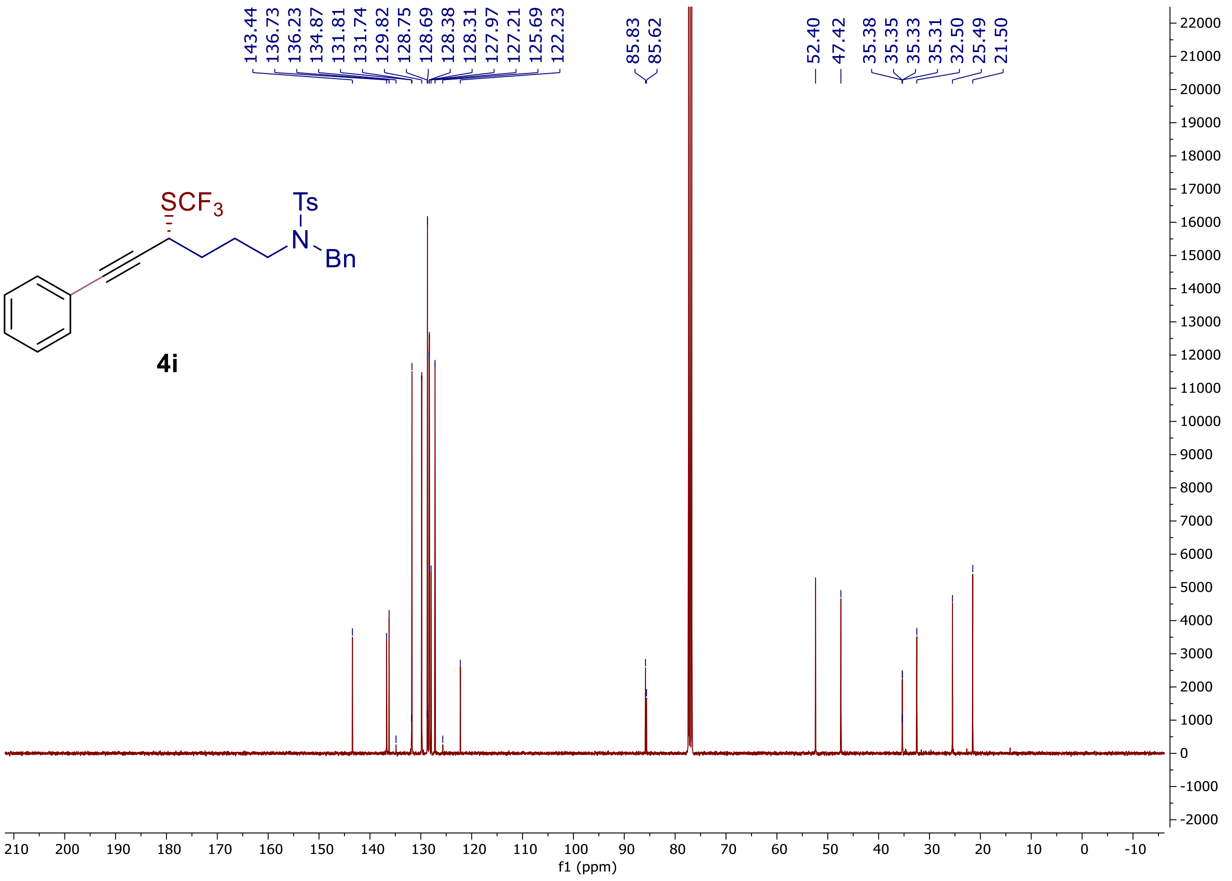
143.44
136.73
136.23
134.87
131.81
131.74
129.82
128.75
128.69
128.38
128.31
127.97
127.21
125.69
122.23
85.83
85.62
52.40
47.42
35.38
35.35
35.33
35.31
32.50
25.49
21.50
SCF3
Ts
N
Bn
4i
f1 (ppm)

^{1}H NMR:

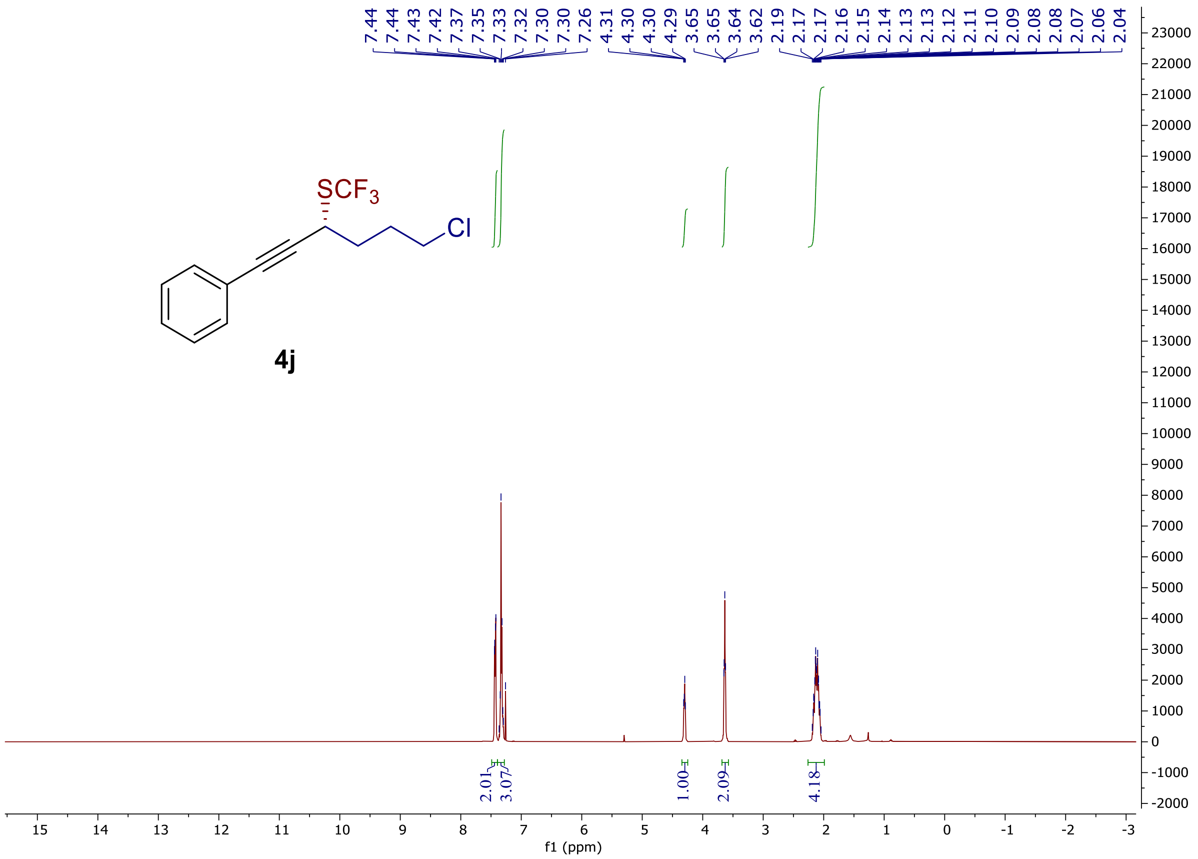

^{19}F NMR:

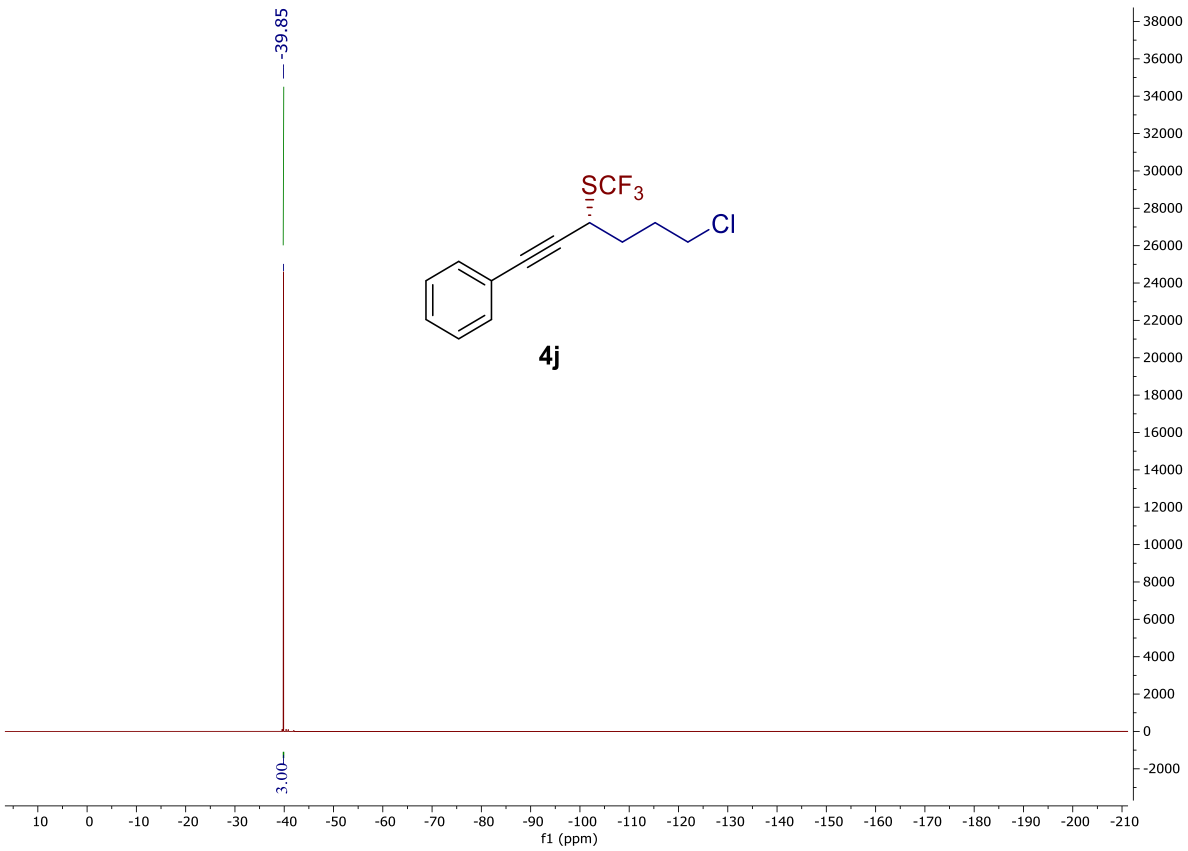

^{13}C NMR:

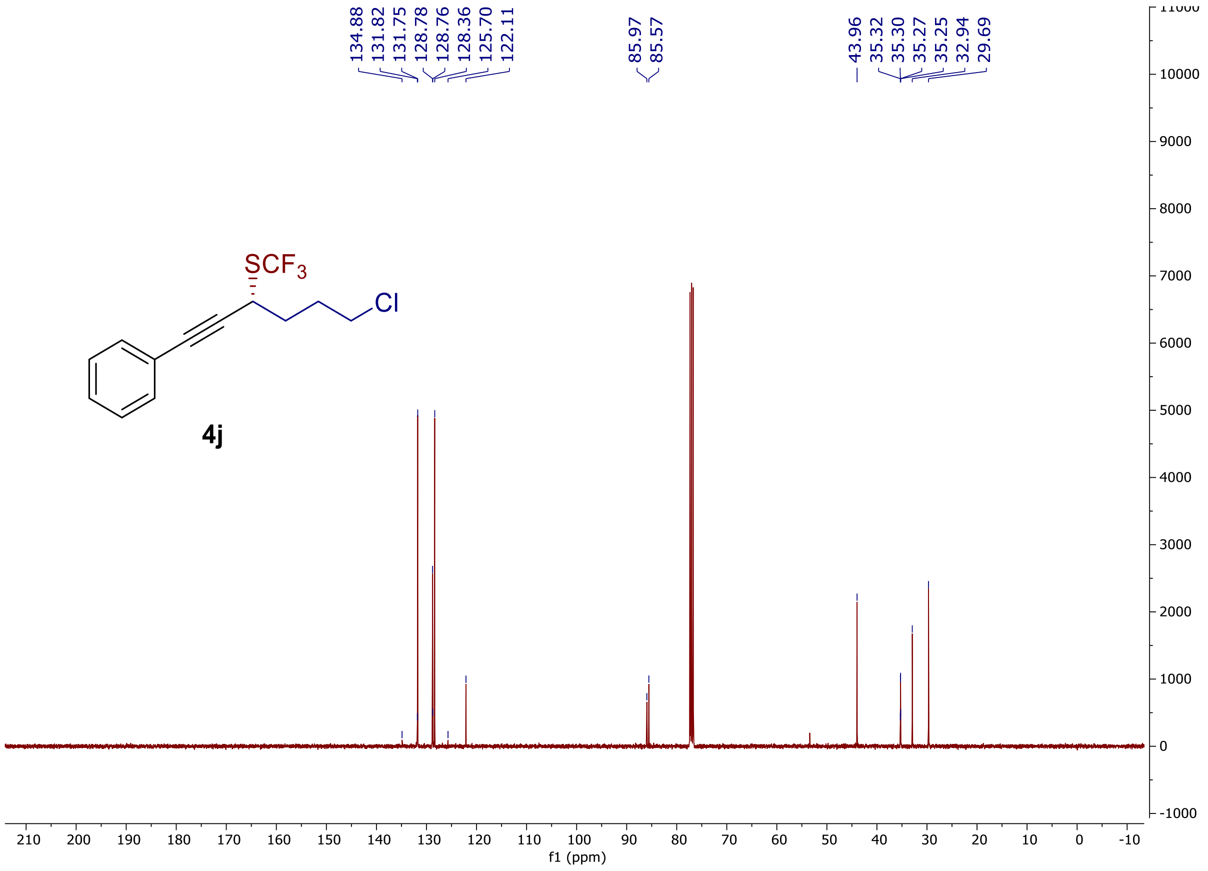

134.88
131.82
131.75
128.78
128.76
128.36
125.70
122.11
85.97
85.57
43.96
35.32
35.30
35.27
35.25
32.94
29.69
SCF3
Cl
4j
f1 (ppm)

^{1}H NMR:

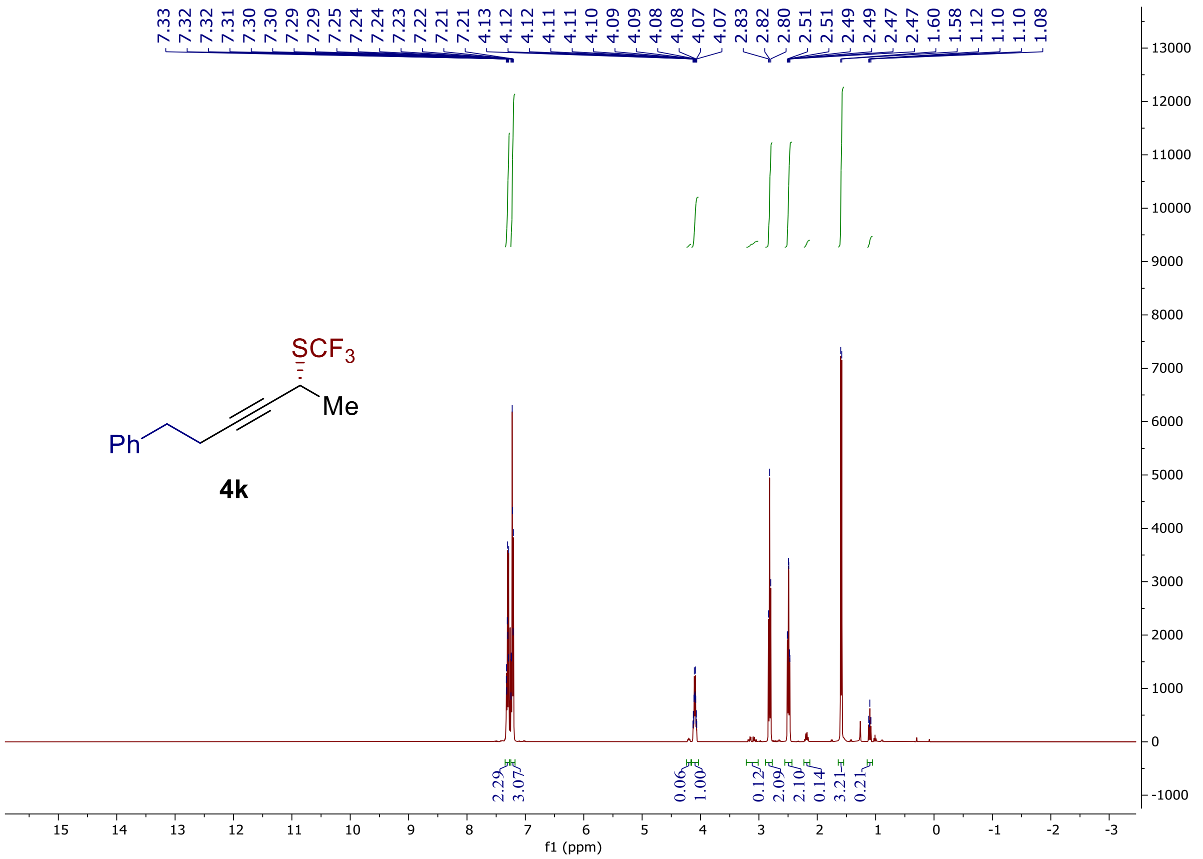

^{19}F NMR:

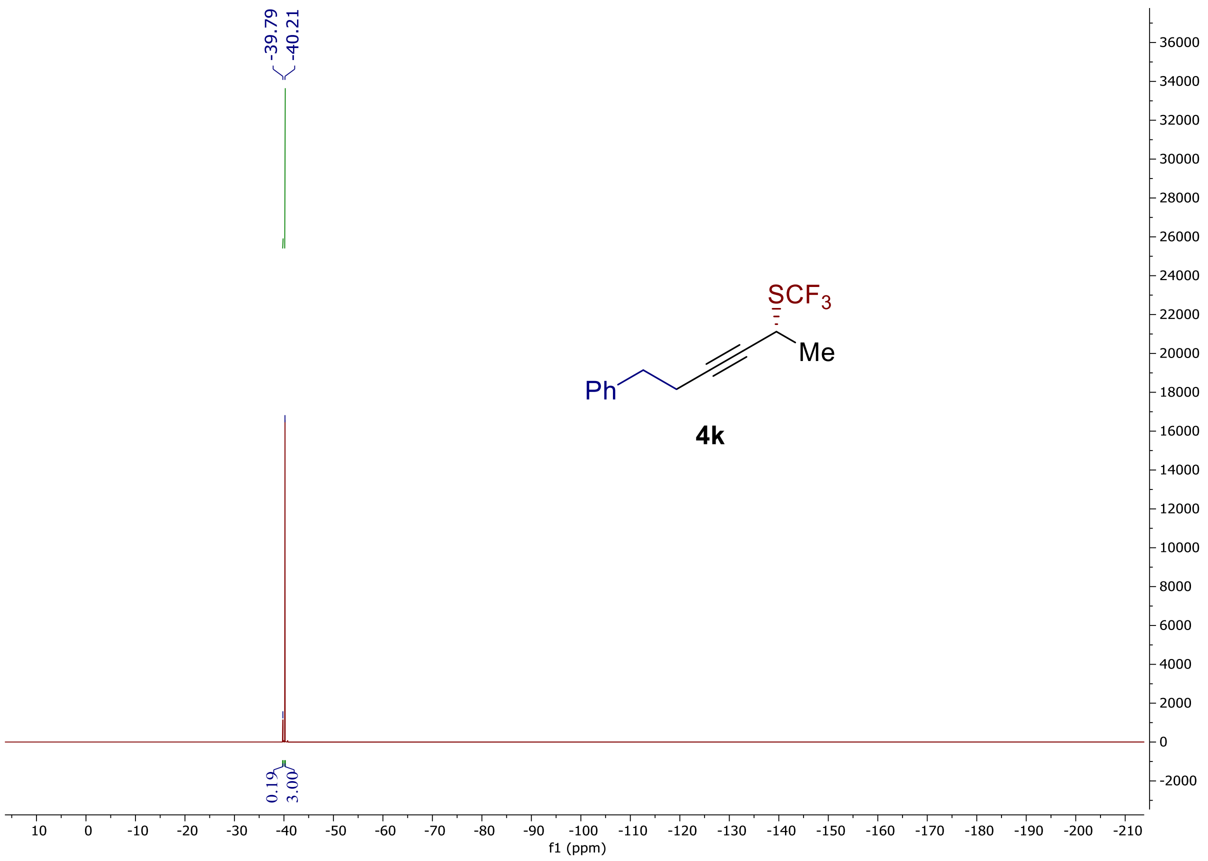

^{13}C NMR:

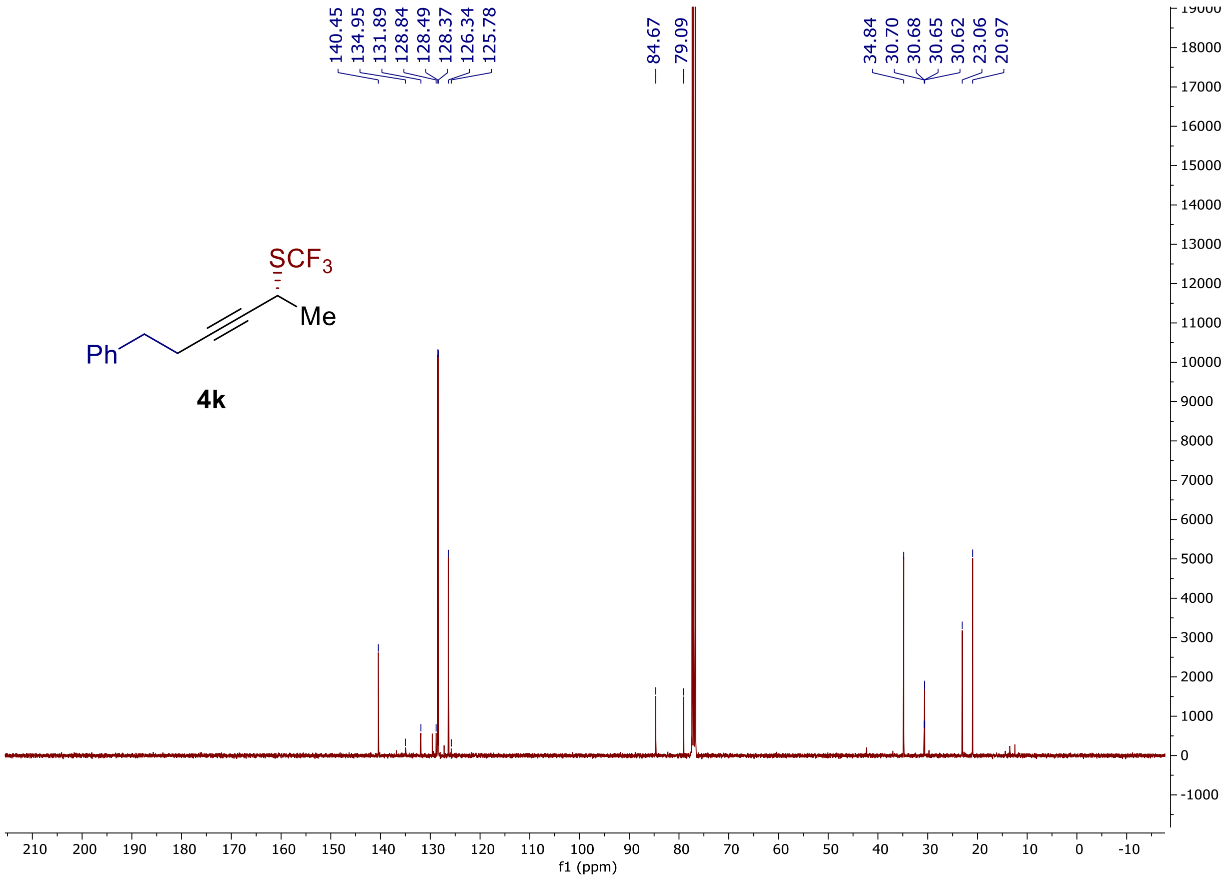
140.45
134.95
131.89
128.84
128.49
128.37
126.34
125.78
84.67
79.09
34.84
30.70
30.68
30.65
30.62
23.06
20.97
SCF3
Me
Ph
4k
f1 (ppm)

^{1}H NMR:

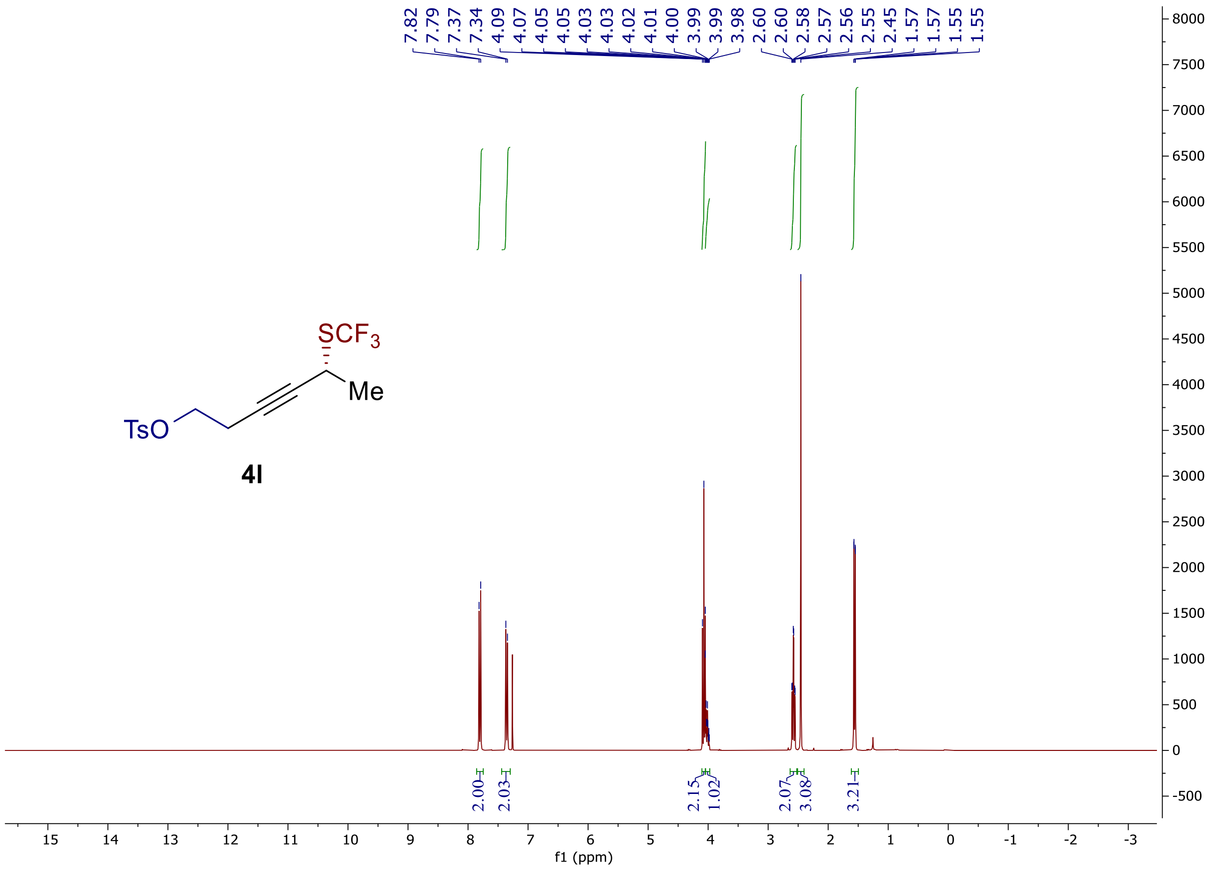

^{19}F NMR:

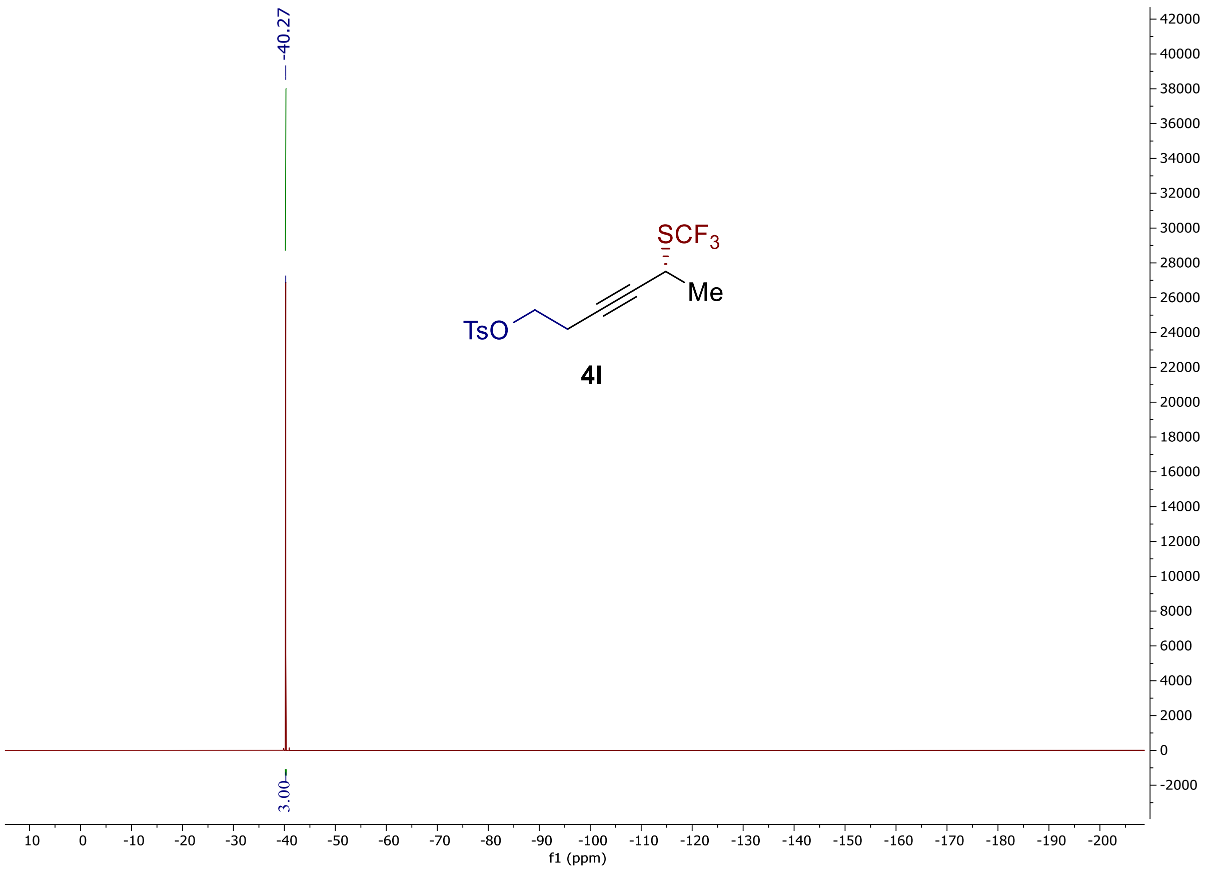

^{13}C NMR:

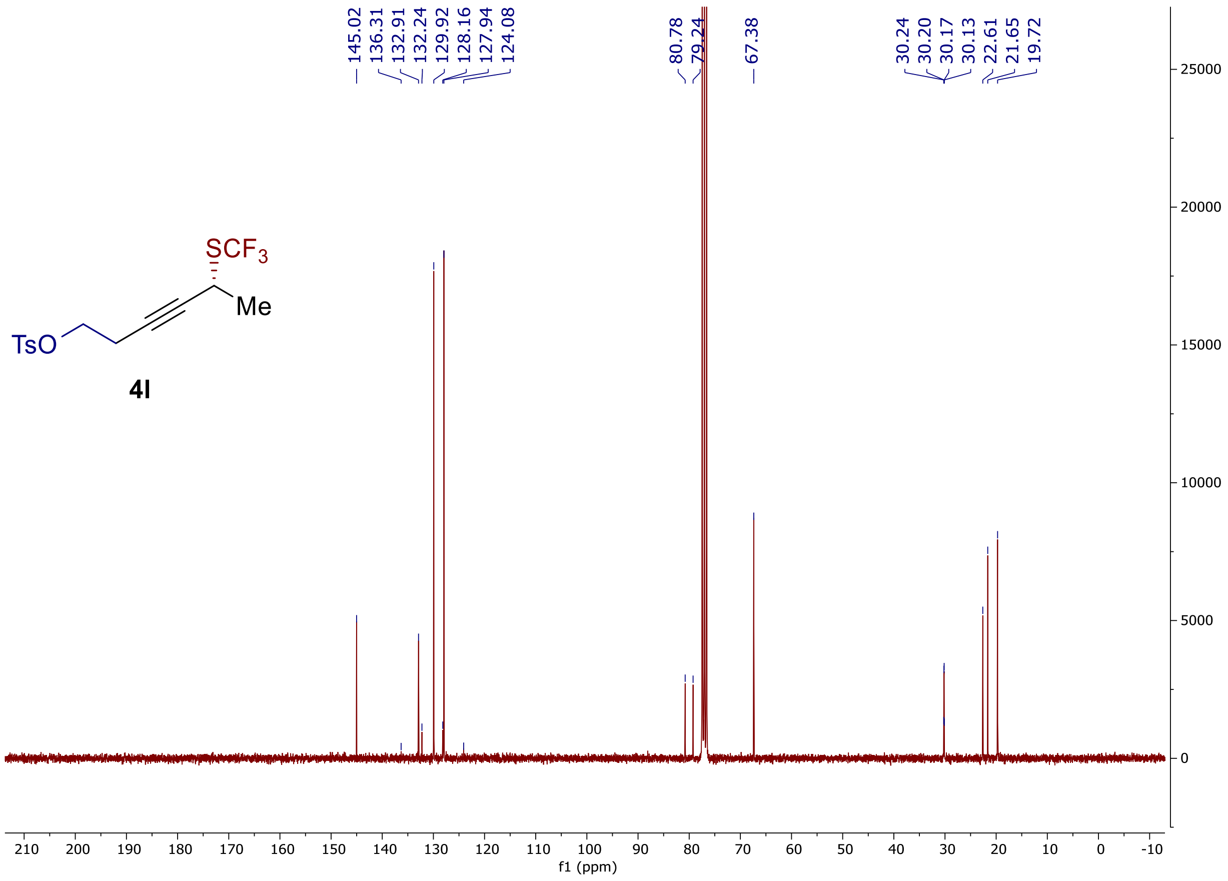
SCF3
Me
TsO
4l
145.02
136.31
132.91
132.24
129.92
128.16
127.94
124.08
80.78
79.24
67.38
30.24
30.20
30.17
30.13
22.61
21.65
19.72
25000
20000
15000
10000
5000
0
210
200
190
180
170
160
150
140
130
120
110
100
90
80
70
60
50
40
30
20
10
0
-10
f1 (ppm)

^{1}H NMR:

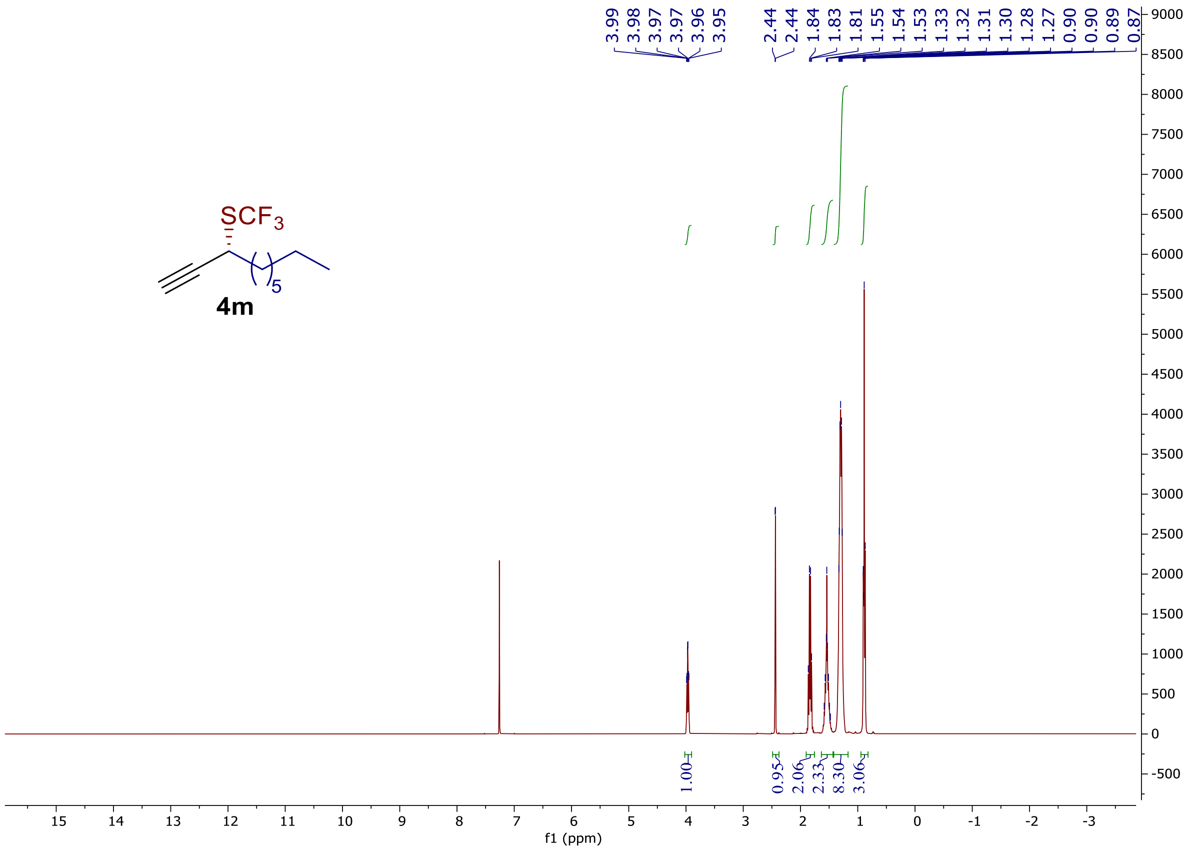

^{19}F NMR:

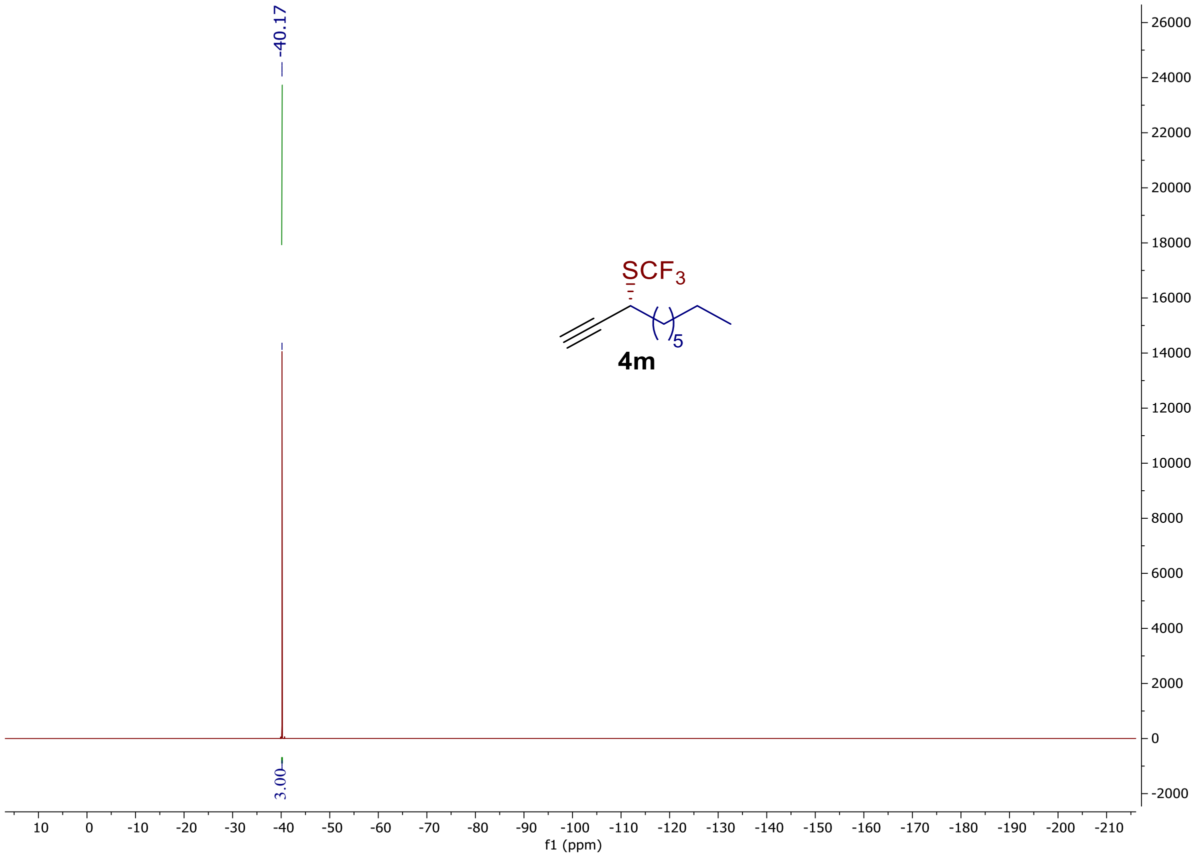

^{13}C NMR:

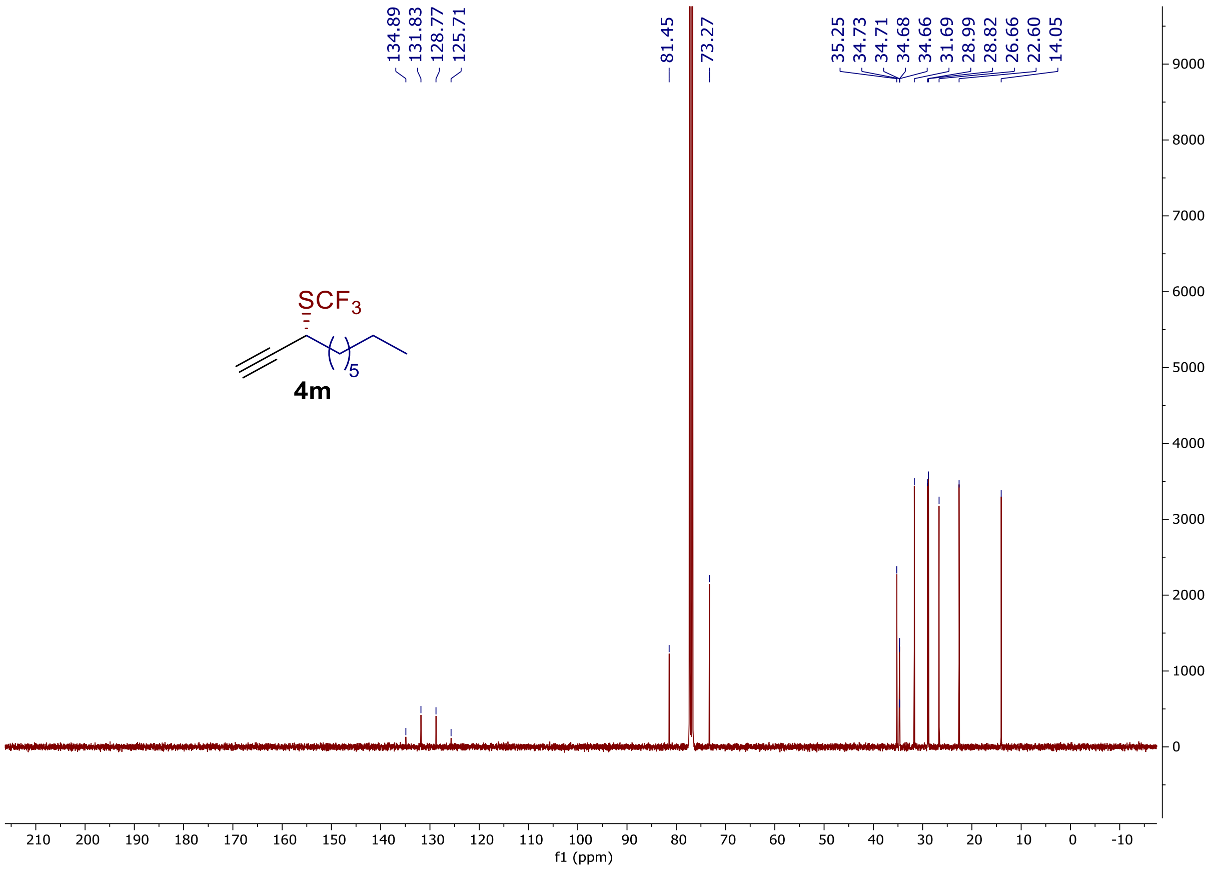
134.89
131.83
128.77
125.71
81.45
73.27
35.25
34.73
34.71
34.68
34.66
31.69
28.99
28.82
26.66
22.60
14.05
SCF3
5
4m
9000
8000
7000
6000
5000
4000
3000
2000
1000
0
210 200 190 180 170 160 150 140 130 120 110 100 90 80 70 60 50 40 30 20 10 0 -10
f1 (ppm)

^{1}H NMR:

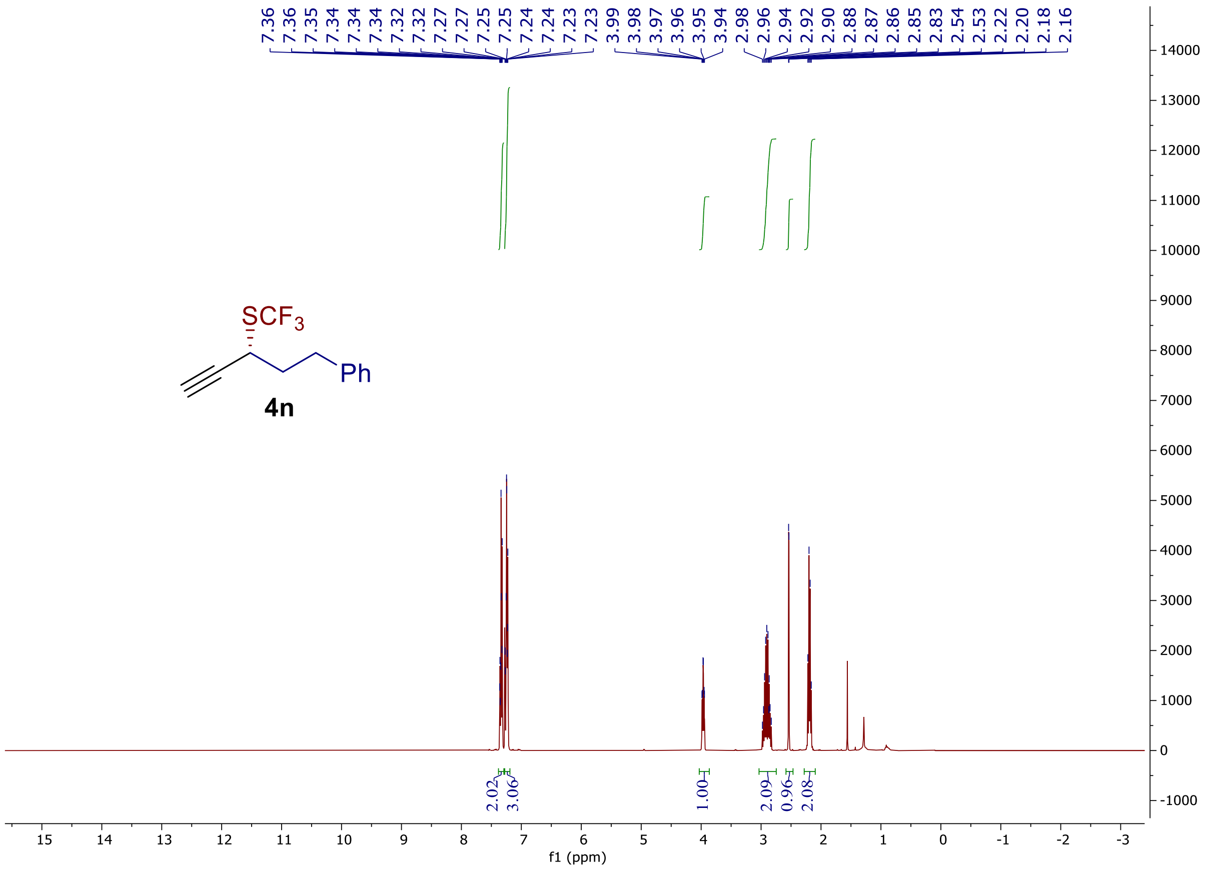

^{19}F NMR:

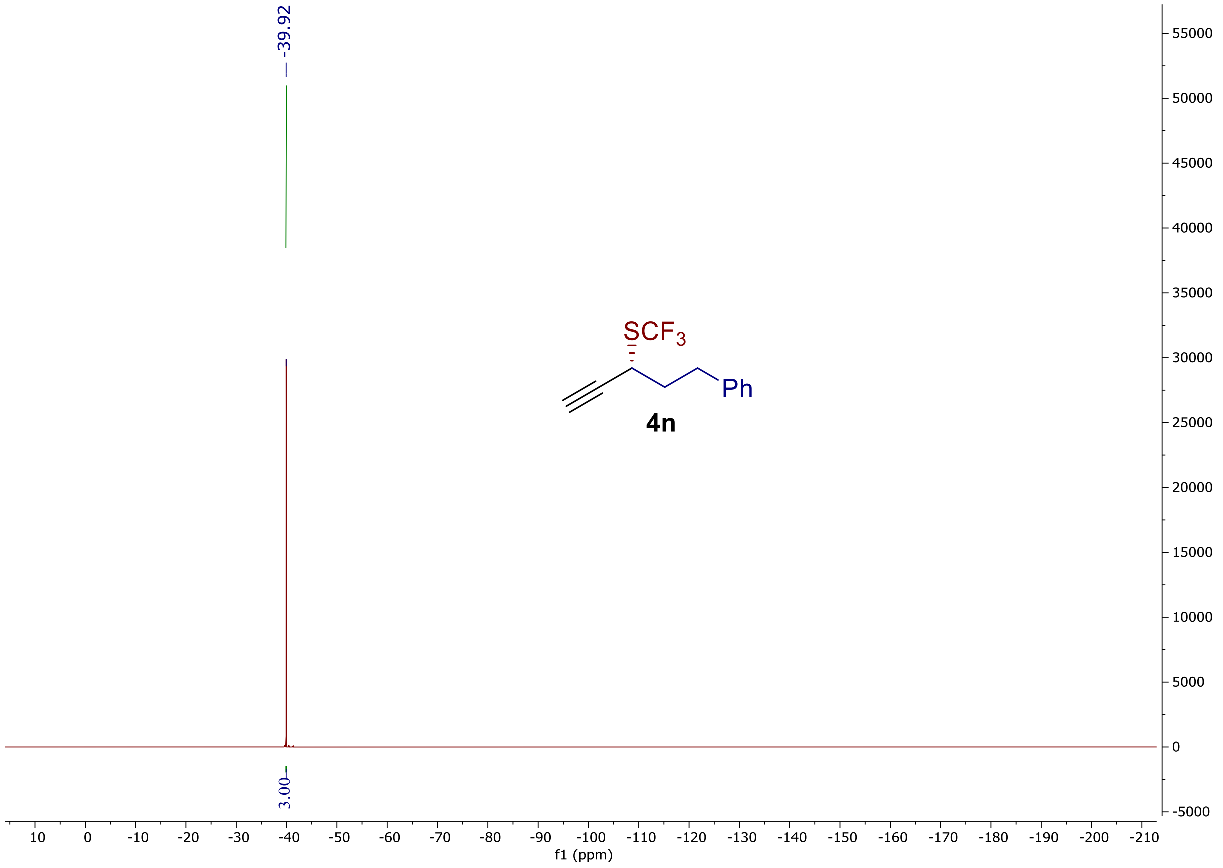

^{13}C NMR:

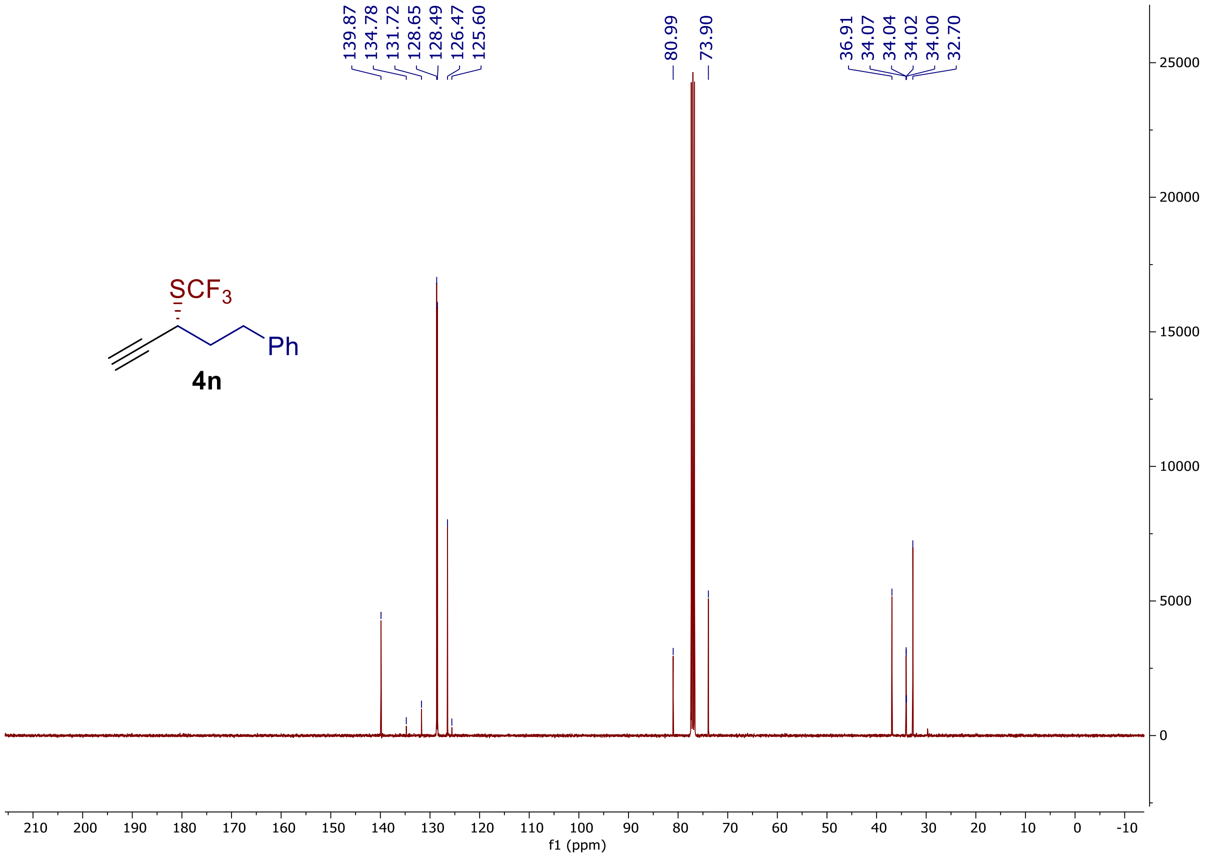
139.87
134.78
131.72
128.65
128.49
126.47
125.60
80.99
73.90
36.91
34.07
34.04
34.02
34.00
32.70
SCF3
Ph
4n
25000
20000
15000
10000
5000
0
210 200 190 180 170 160 150 140 130 120 110 100 90 80 70 60 50 40 30 20 10 0 -10
f1 (ppm)

^{1}H NMR:

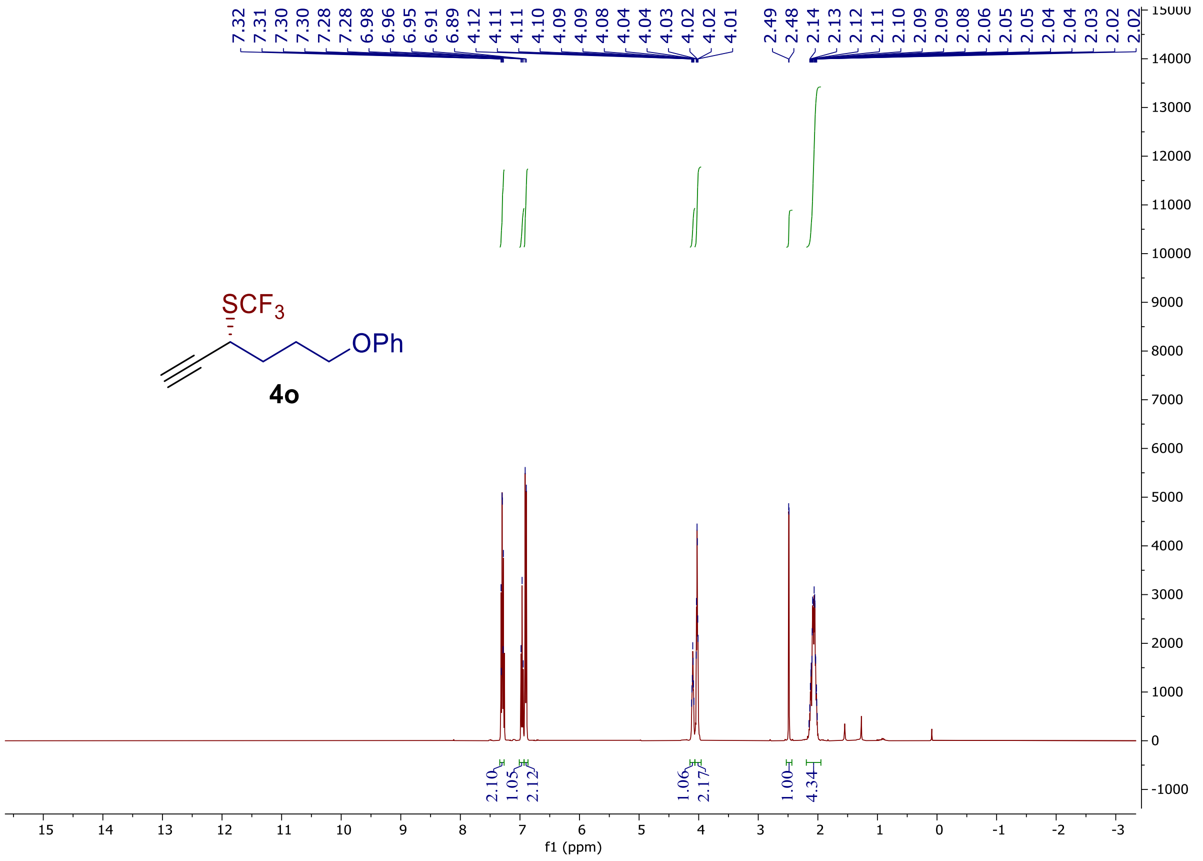

^{19}F NMR:

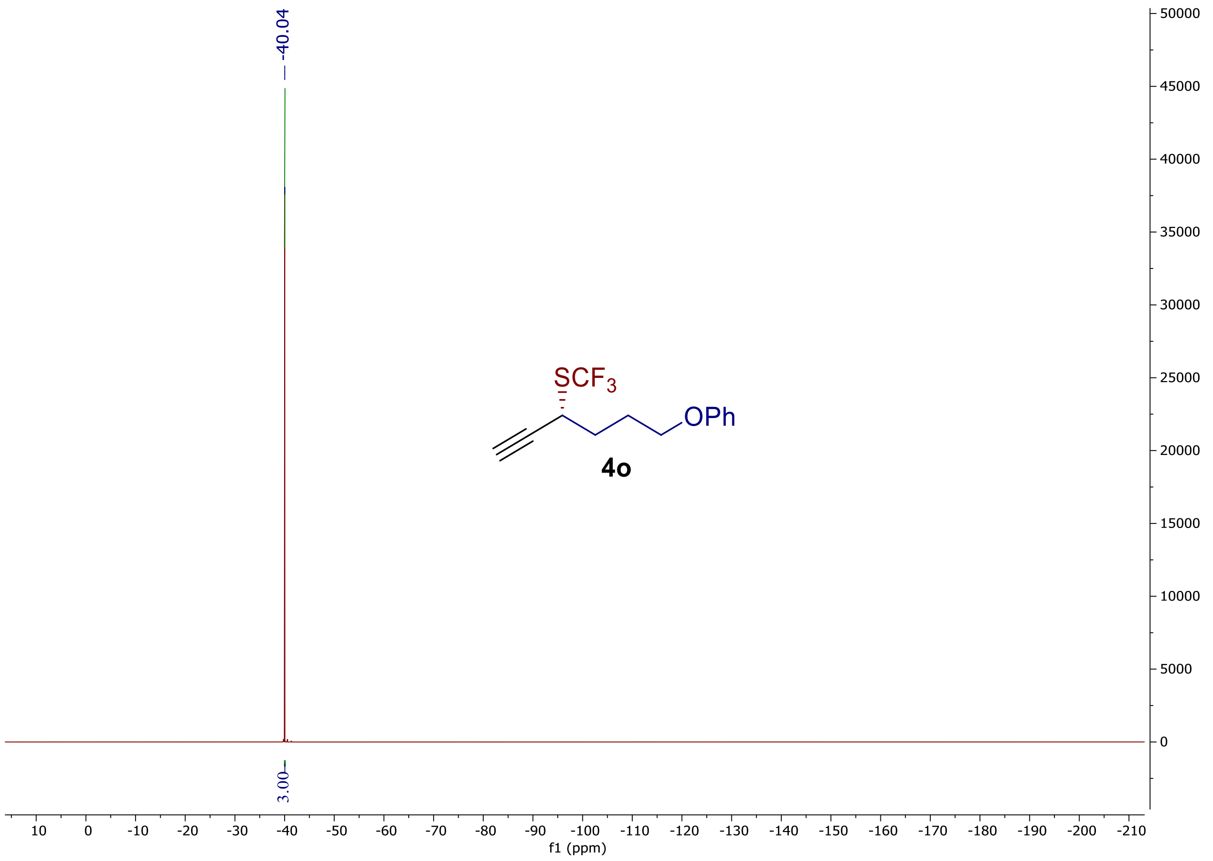

^{13}C NMR:

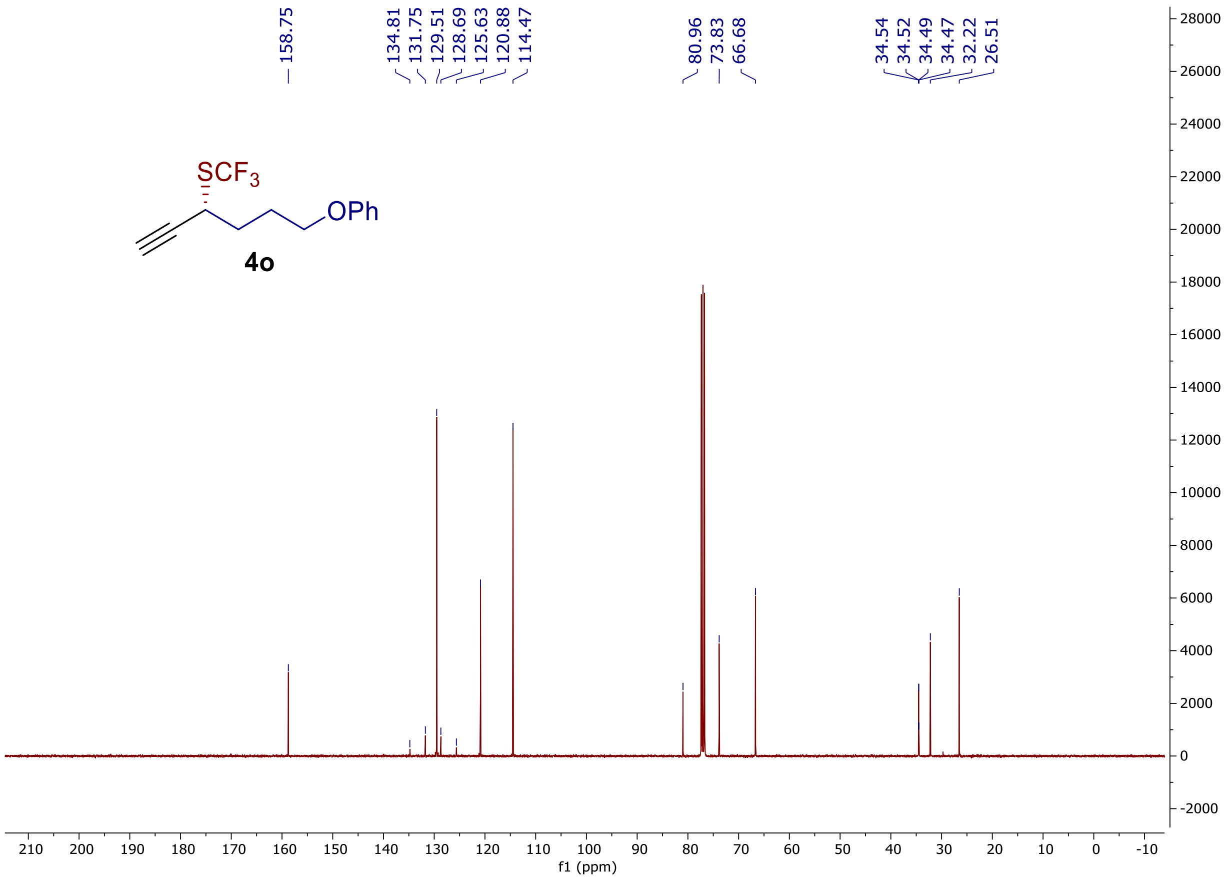
158.75
134.81
131.75
129.51
128.69
125.63
120.88
114.47
80.96
73.83
66.68
34.54
34.52
34.49
34.47
32.22
26.51
SCF3
OPh
4o
f1 (ppm)

^{1}H NMR:

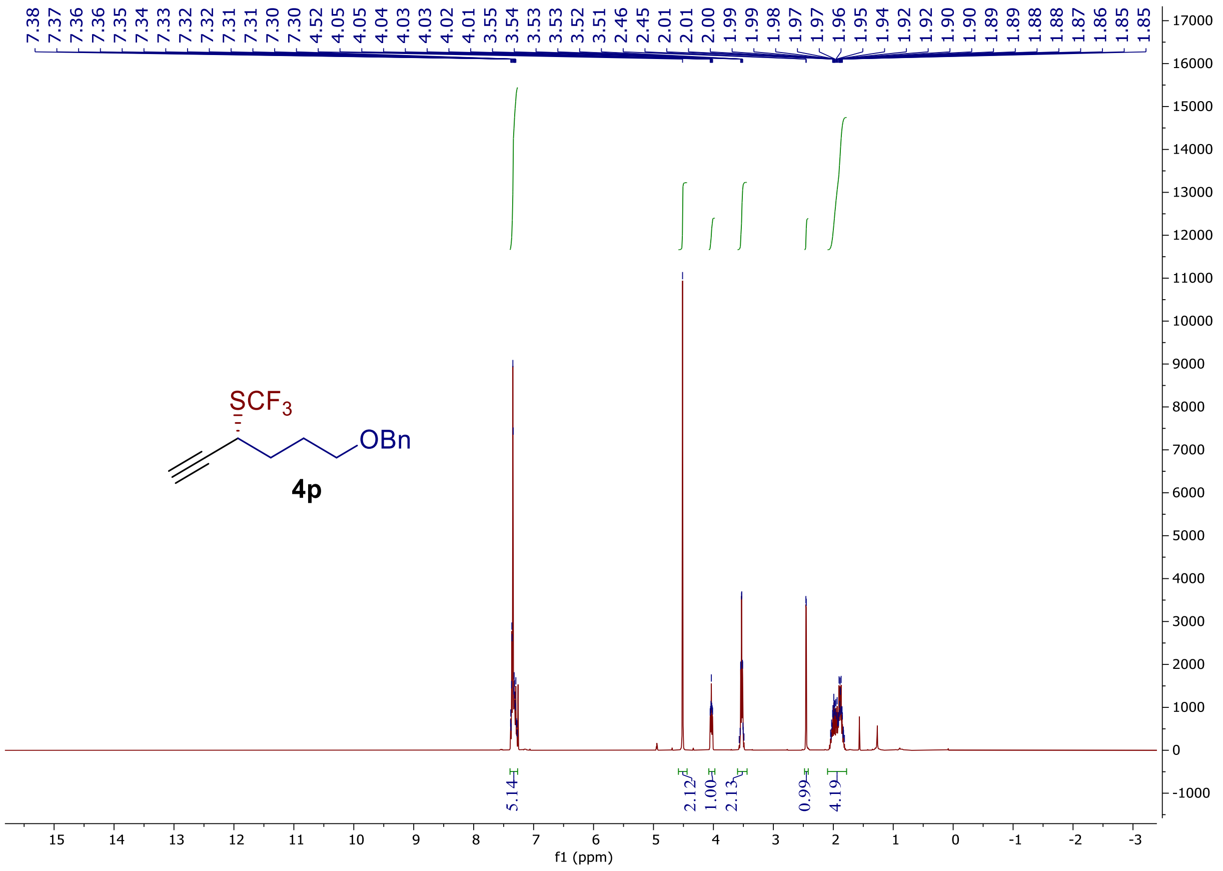

^{19}F NMR:

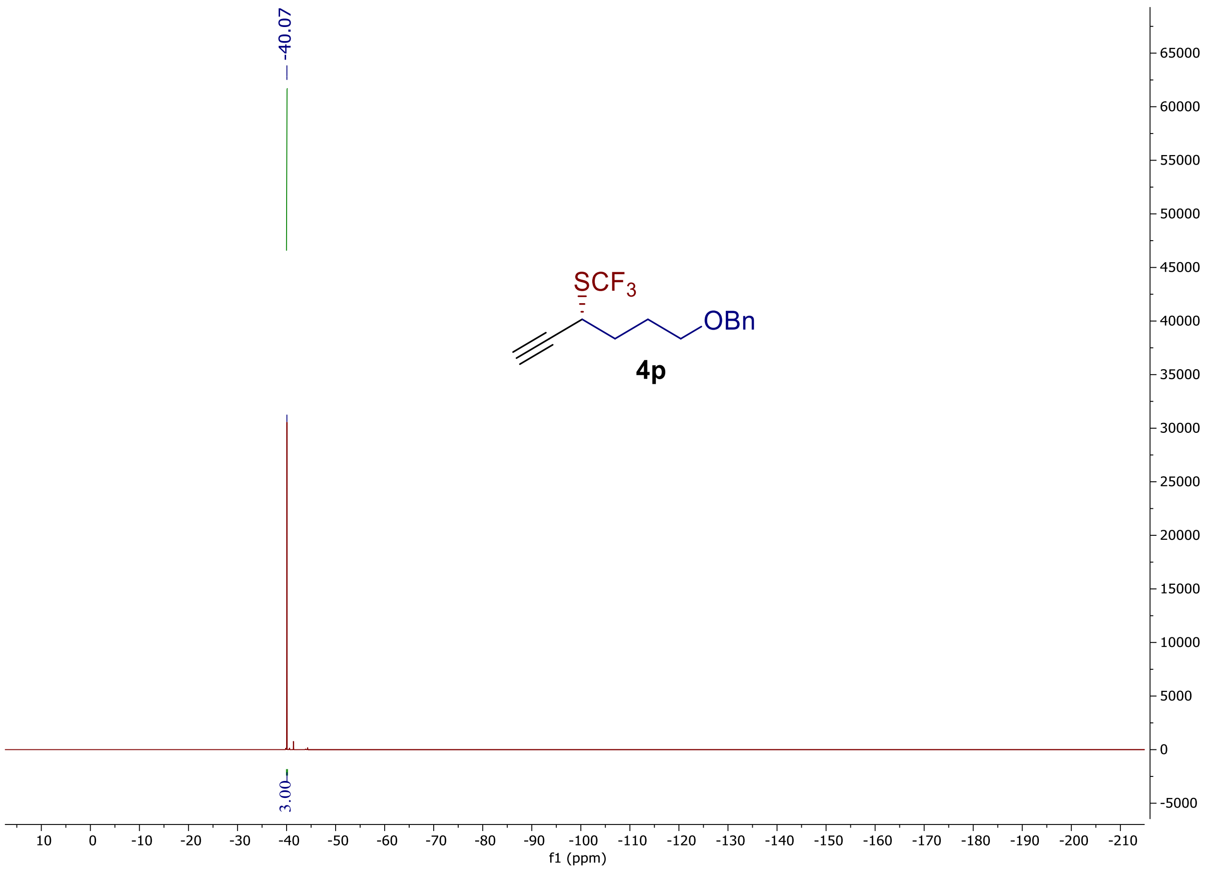

^{13}C NMR:

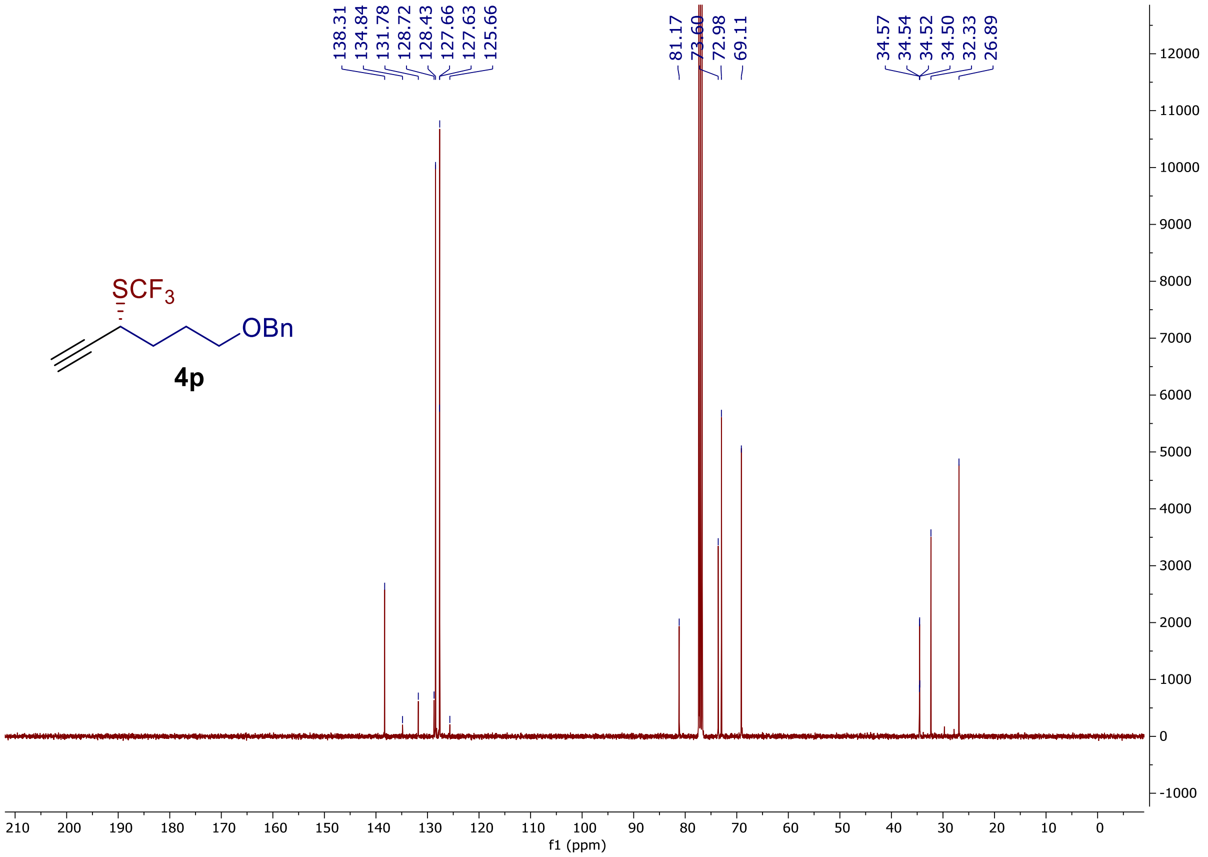
138.31
134.84
131.78
128.72
128.43
127.66
127.63
125.66
81.17
73.60
72.98
69.11
34.57
34.54
34.52
34.50
32.33
26.89
SCF3
OBn
4p
f1 (ppm)

^{1}H NMR:

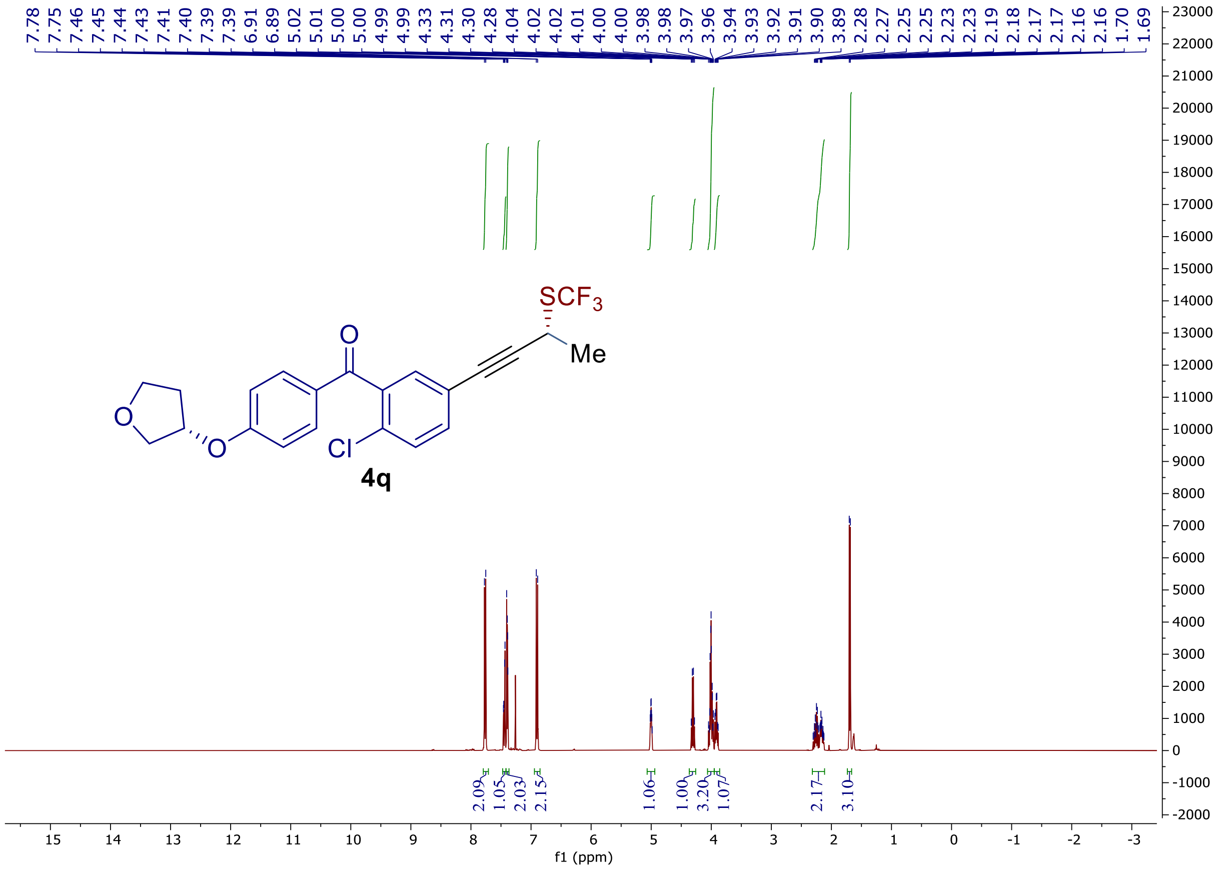

^{19}F NMR:

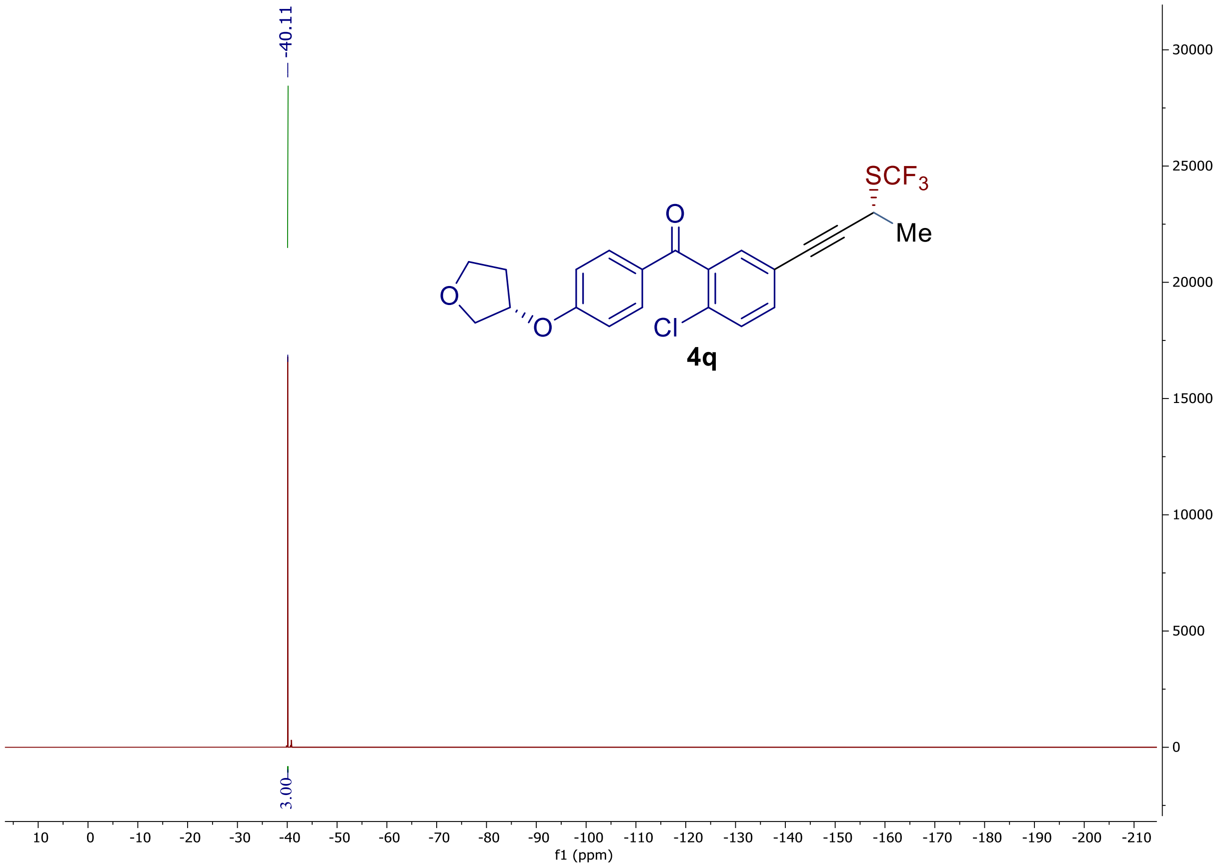

^{13}C NMR:

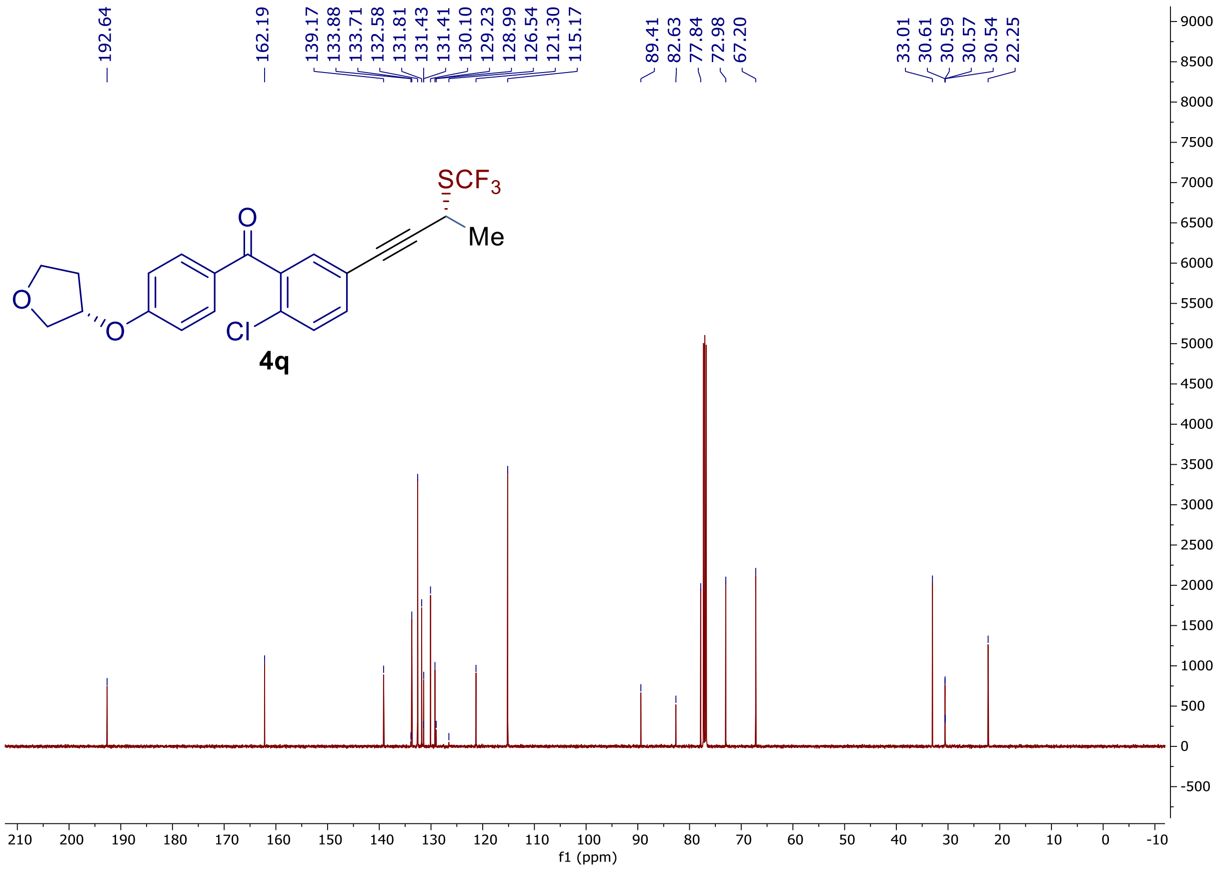

192.64
162.19
139.17
133.88
133.71
132.58
131.81
131.43
131.41
130.10
129.23
128.99
126.54
121.30
115.17
89.41
82.63
77.84
72.98
67.20
33.01
30.61
30.59
30.57
30.54
22.25
SCF3
O
Me
O
O
Cl
4q
f1 (ppm)

^{1}H NMR:

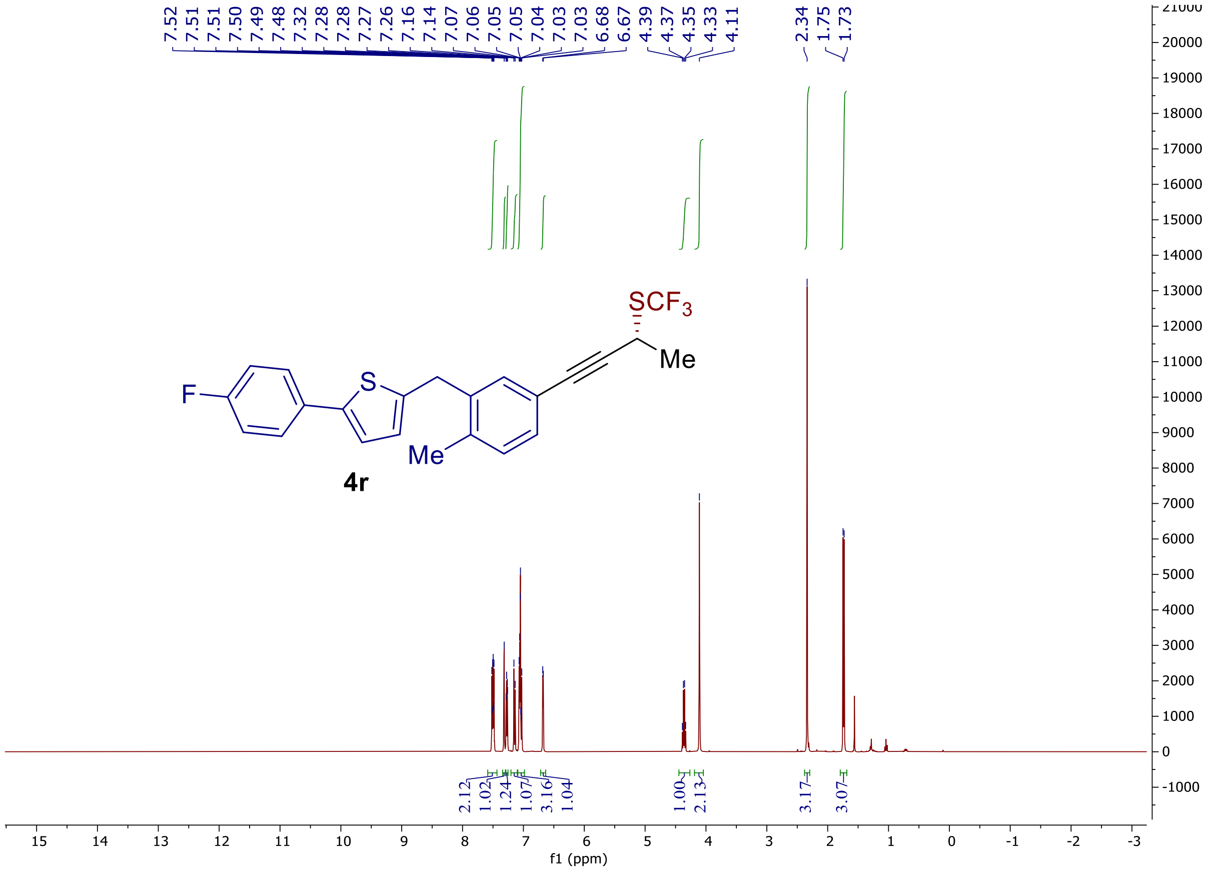

^{19}F NMR:

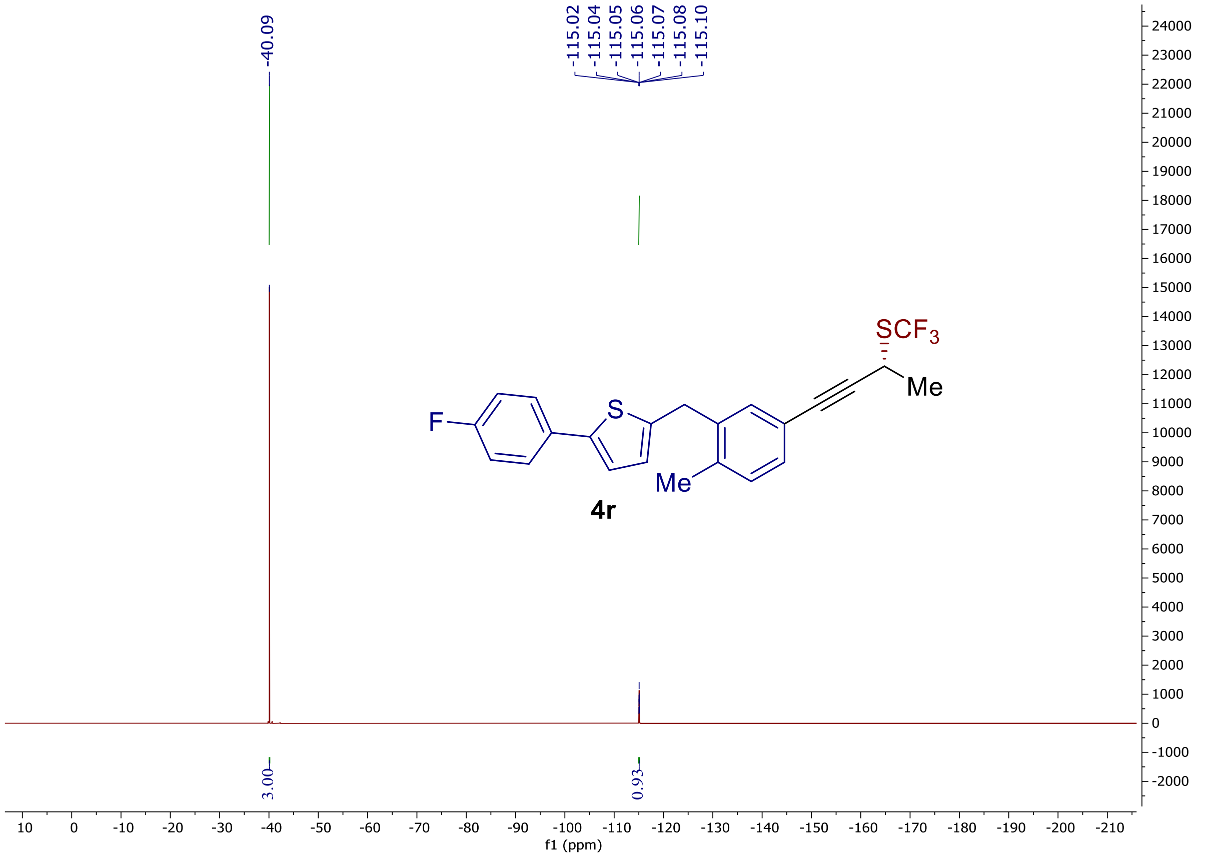

^{13}C NMR:

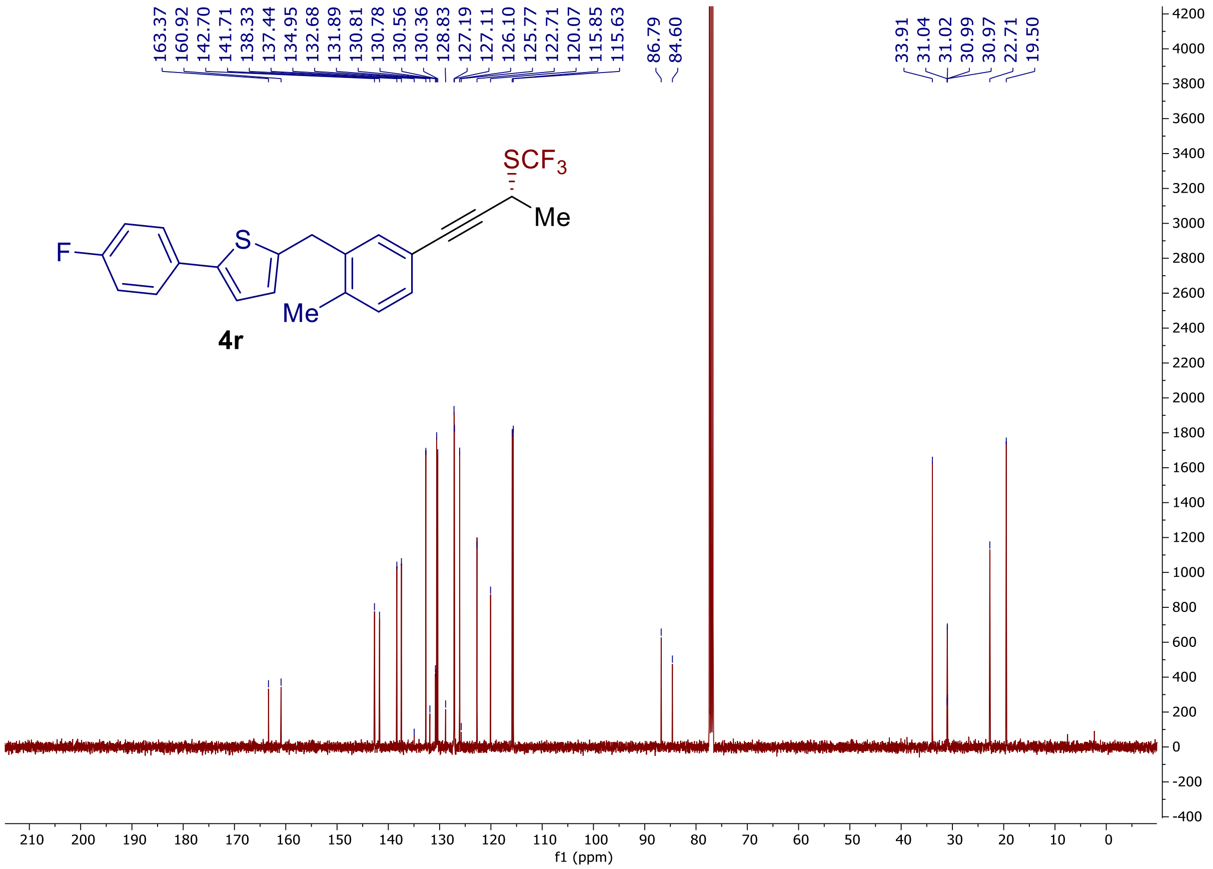
163.37
160.92
142.70
141.71
138.33
137.44
134.95
132.68
131.89
130.81
130.78
130.56
130.36
128.83
127.19
127.11
126.10
125.77
122.71
120.07
115.85
115.63
86.79
84.60
33.91
31.04
31.02
30.99
30.97
22.71
19.50
SCF3
Me
F
S
Me
4r
f1 (ppm)

^{1}H NMR:

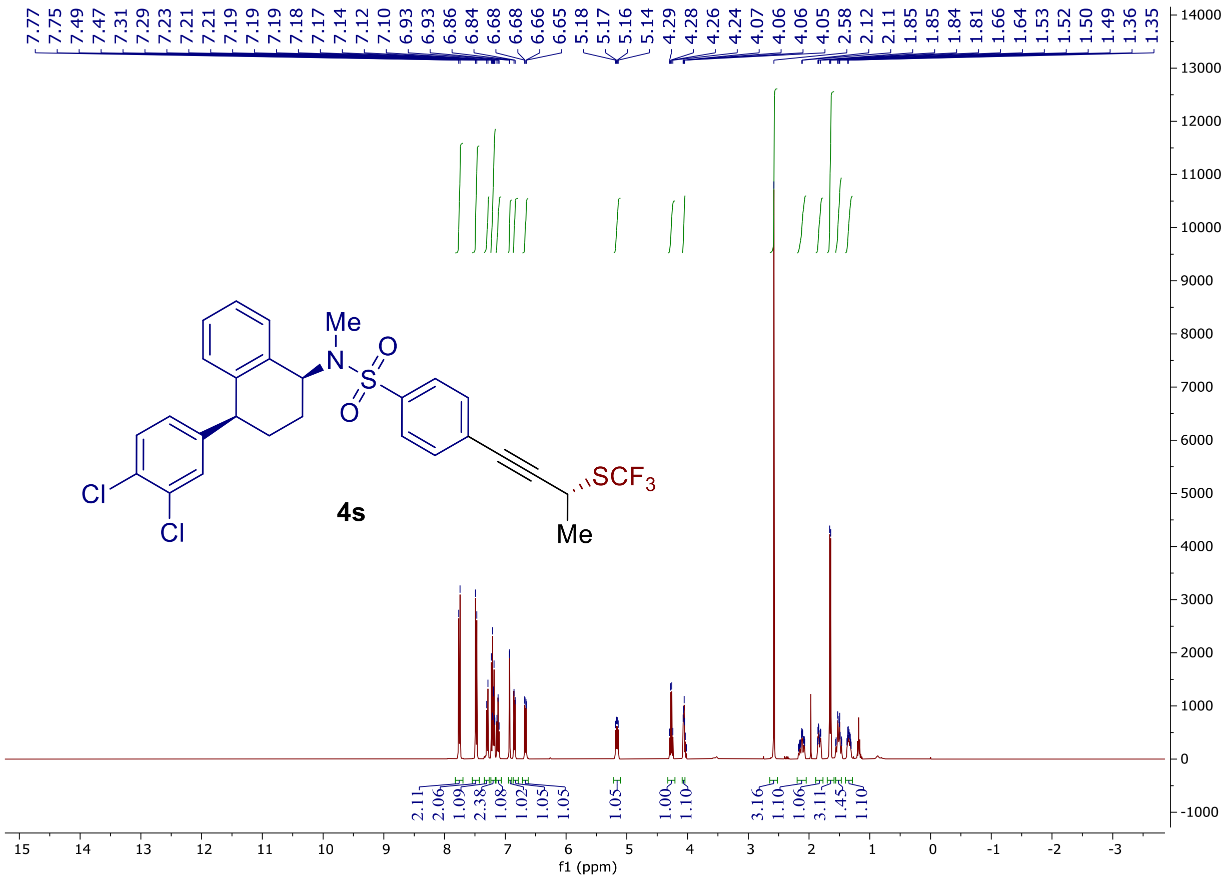

^{19}F NMR:

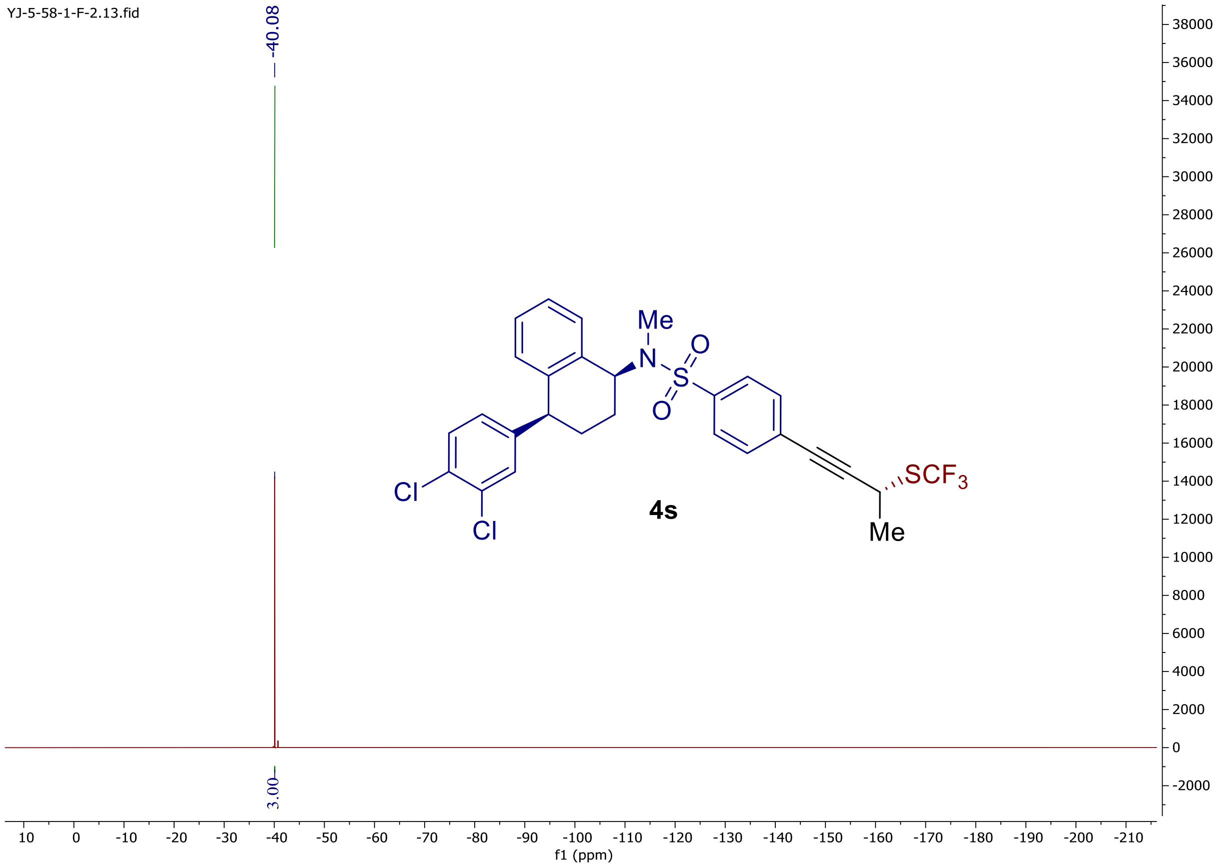

^{13}C NMR:

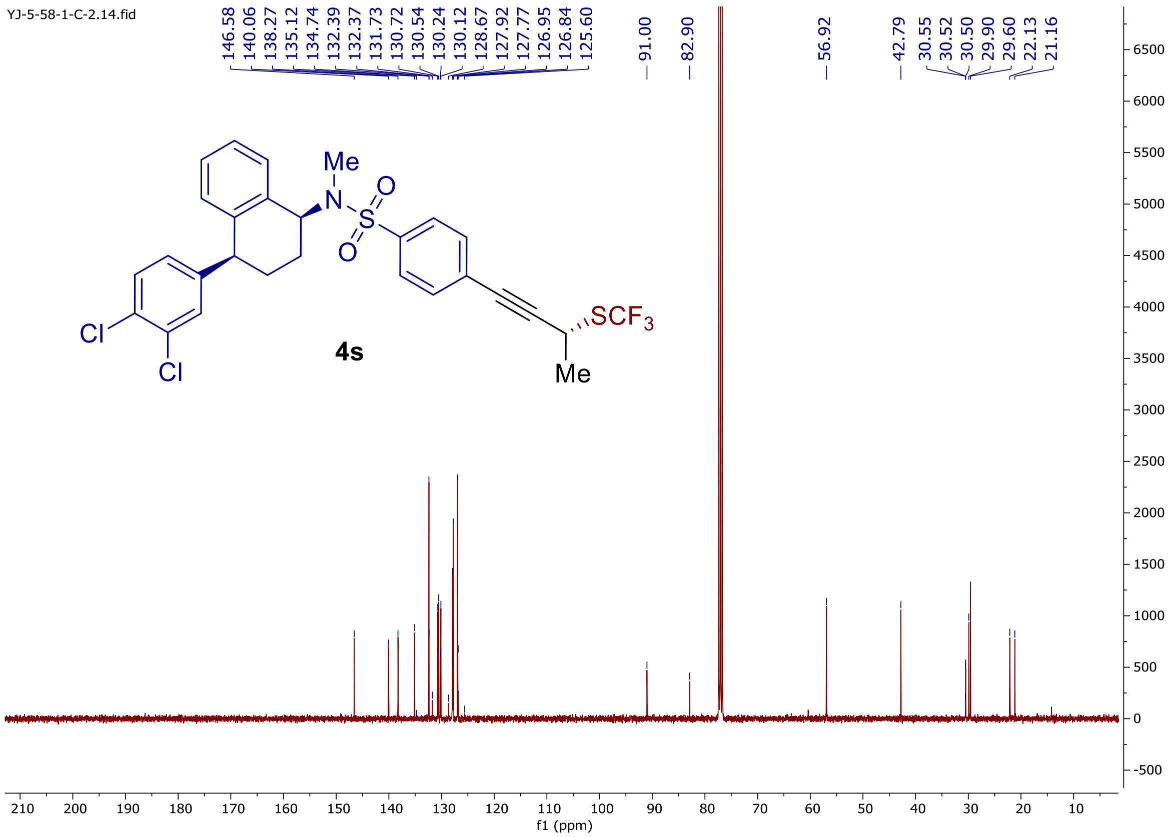

YJ-5-58-1-C-2.14.fid
146.58
140.06
138.27
135.12
134.74
132.39
132.37
131.73
130.72
130.54
130.24
130.12
128.67
127.92
127.77
126.95
126.84
125.60
91.00
82.90
56.92
42.79
30.55
30.52
30.50
29.90
29.60
22.13
21.16
Me
N
O
S
O
SCF3
Cl
Cl
4s
Me
f1 (ppm)

^{1}H NMR:

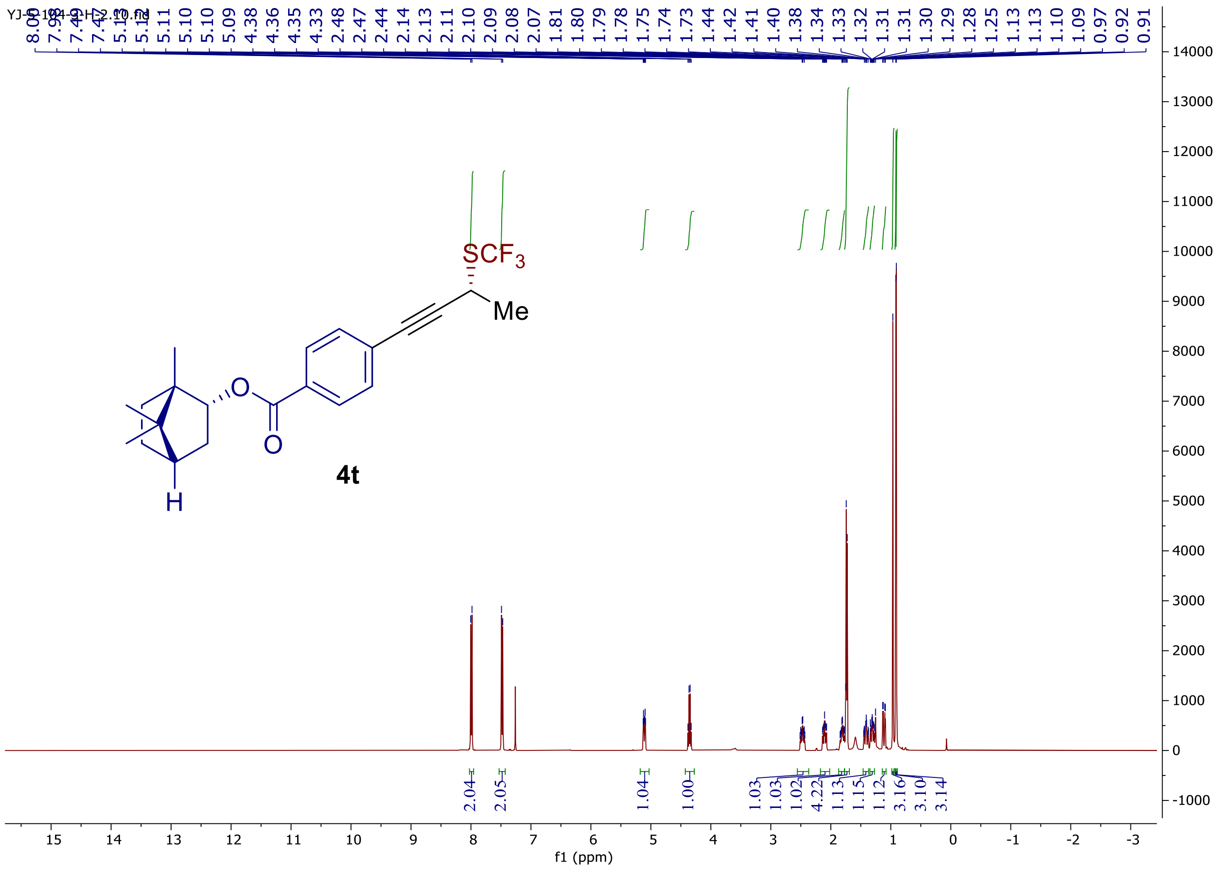

^{19}F NMR:

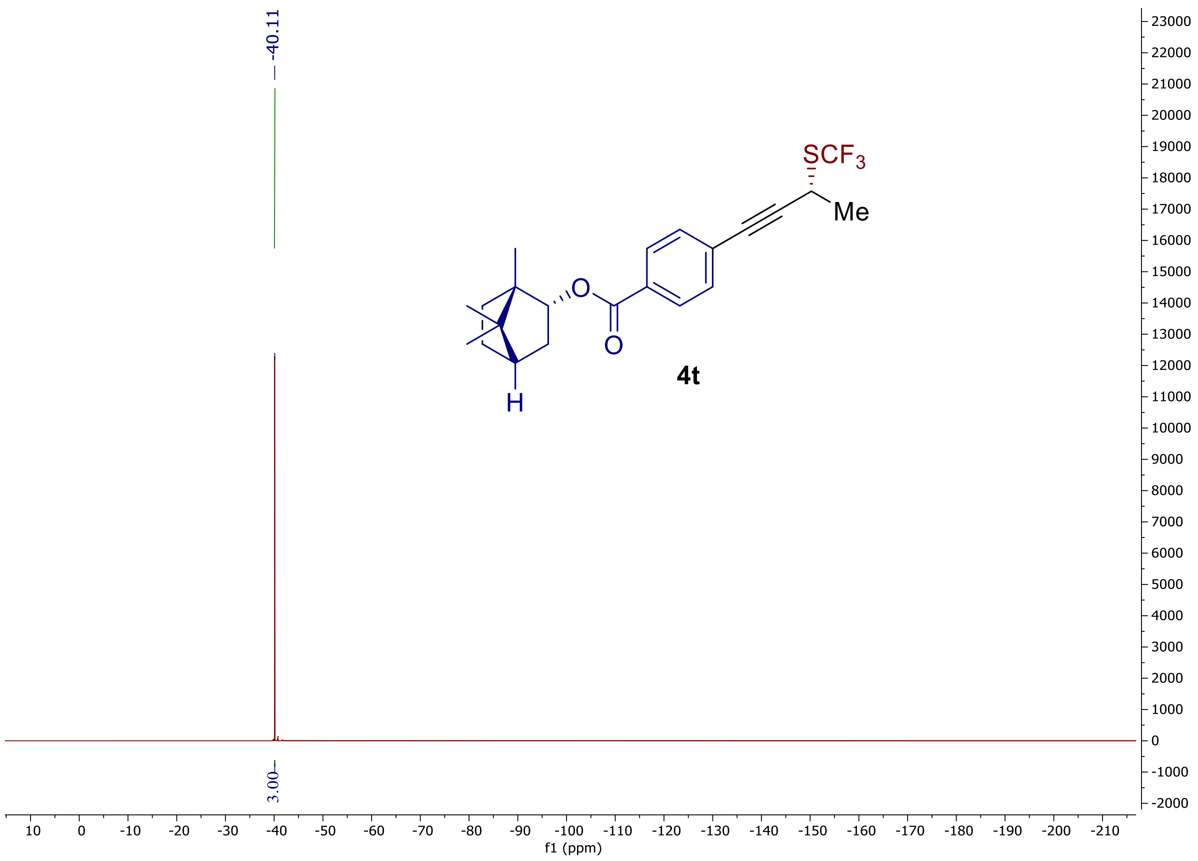

^{13}C NMR:

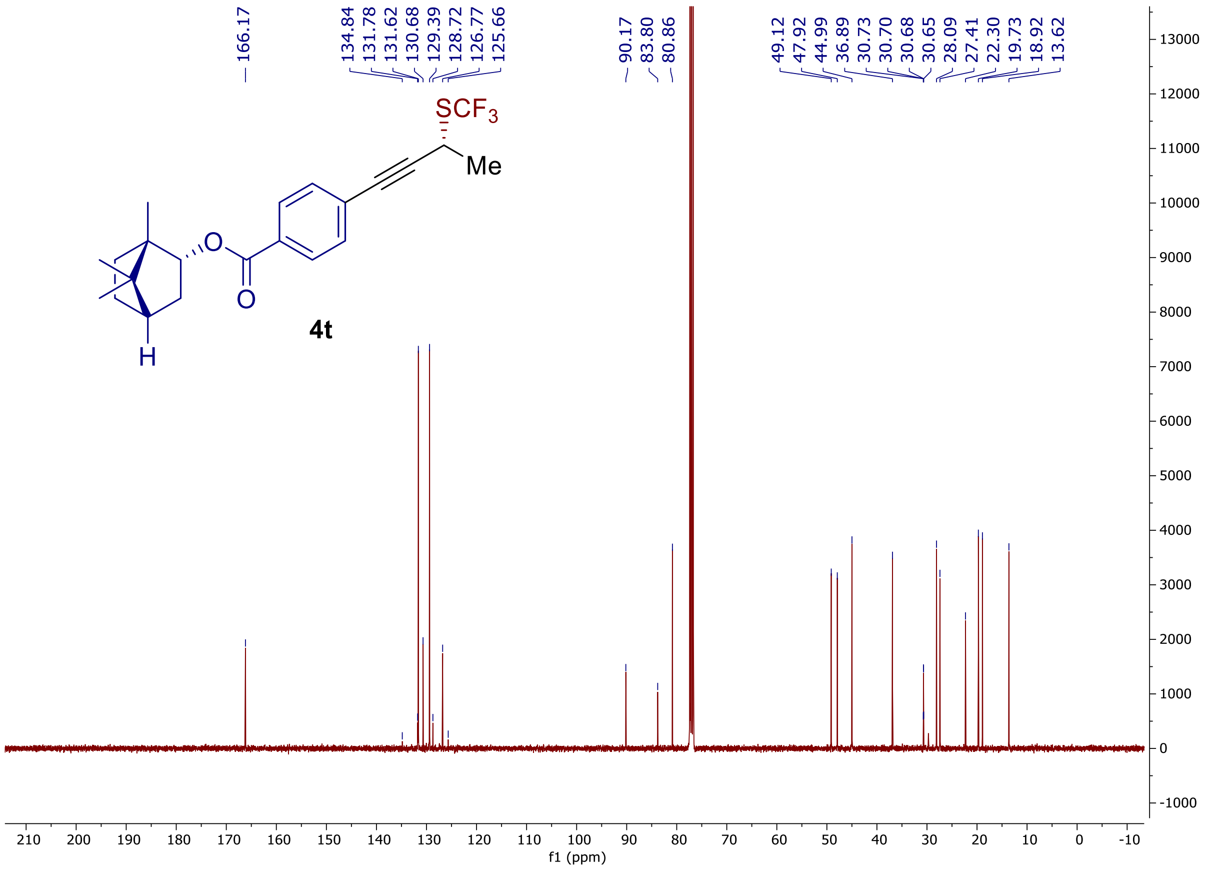

166.17
134.84
131.78
131.62
130.68
129.39
128.72
126.77
125.66
90.17
83.80
80.86
49.12
47.92
44.99
36.89
30.73
30.70
30.68
30.65
28.09
27.41
22.30
19.73
18.92
13.62
SCF3
Me
O
O
H
4t
f1 (ppm)

^{1}H NMR:

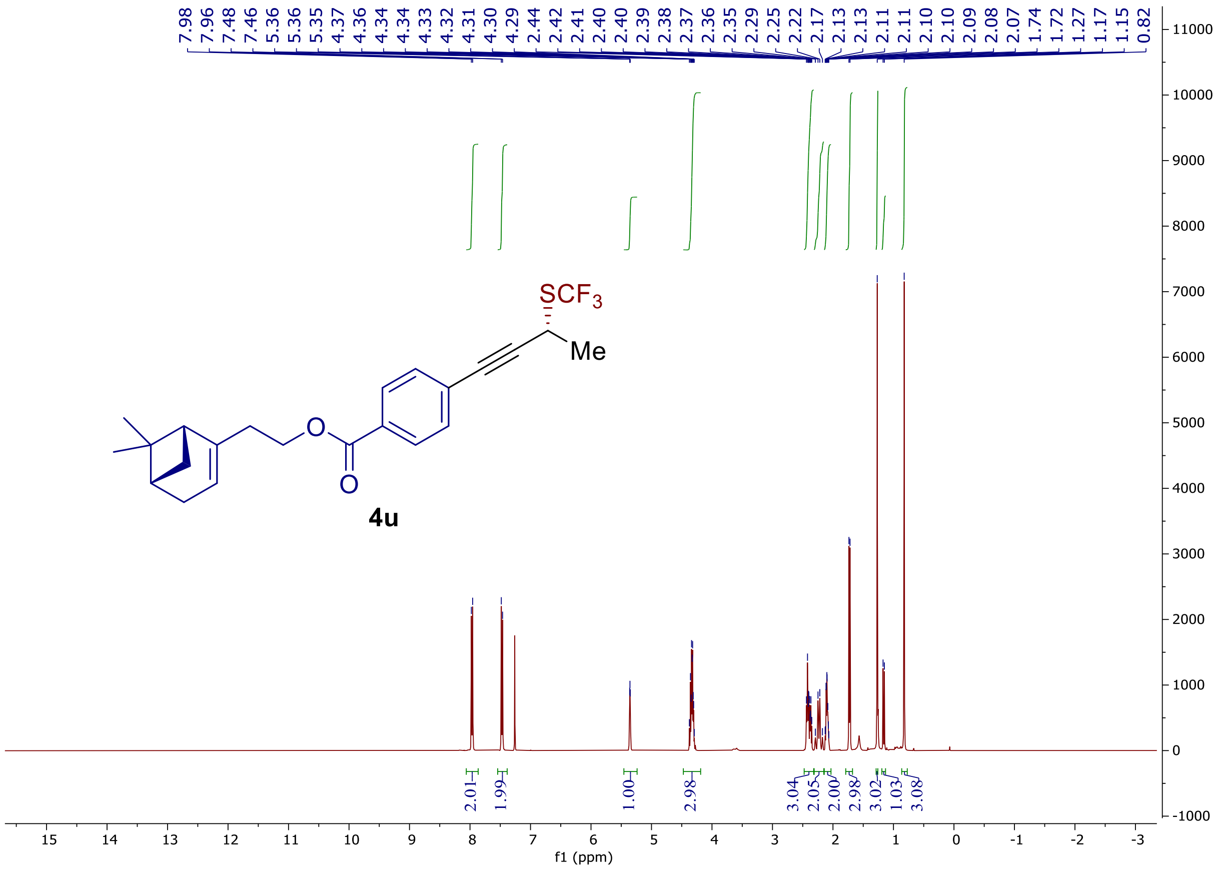

^{19}F NMR:

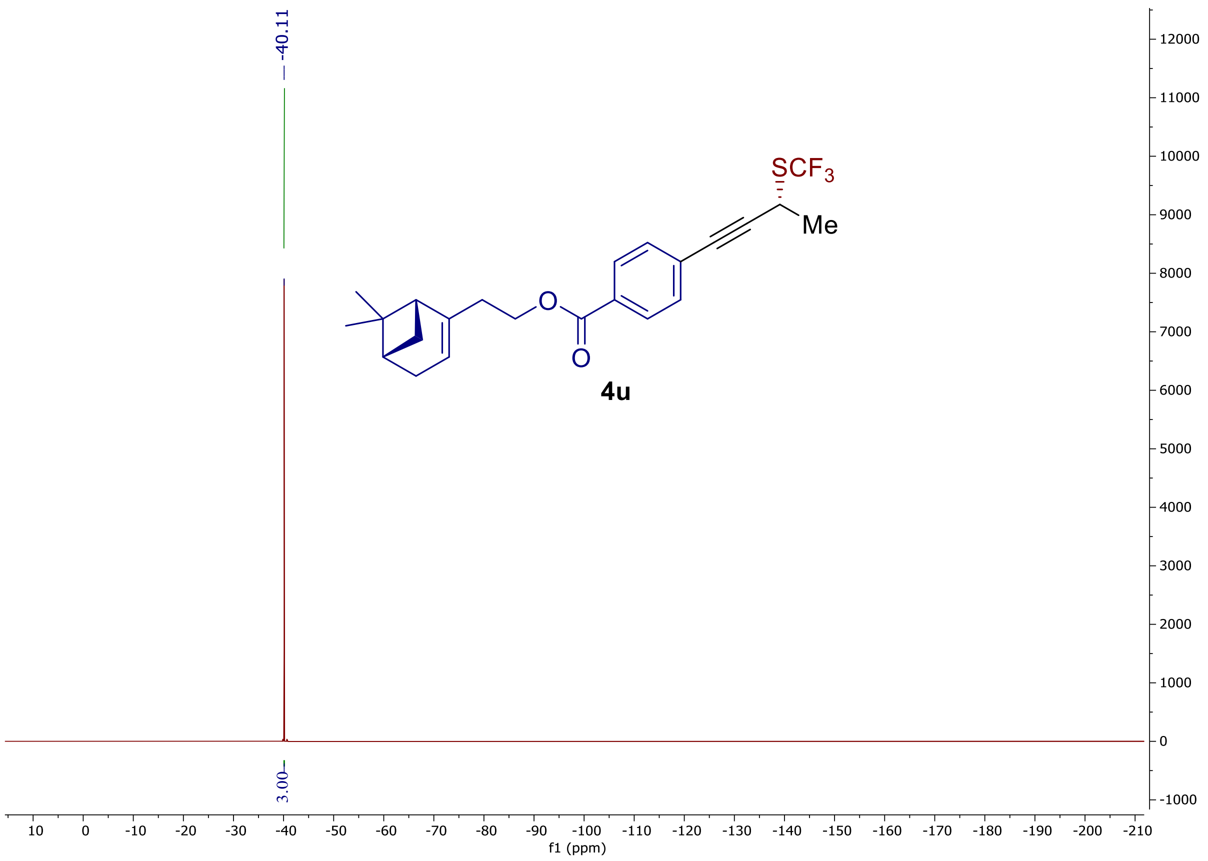

^{13}C NMR:

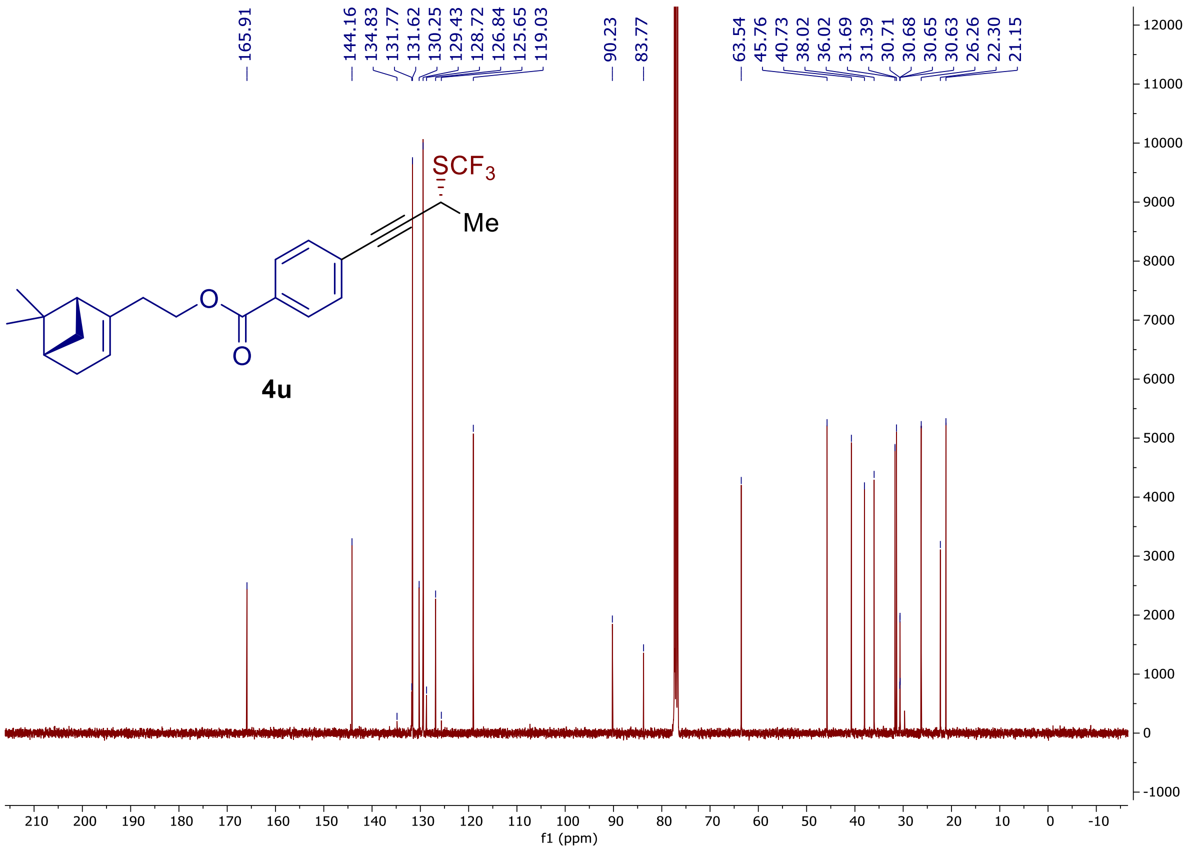

165.91
144.16
134.83
131.77
131.62
130.25
129.43
128.72
126.84
125.65
119.03
90.23
83.77
63.54
45.76
40.73
38.02
36.02
31.69
31.39
30.71
30.68
30.65
30.63
26.26
22.30
21.15
SCF3
Me
O
O
4u
f1 (ppm)

^{1}H NMR:

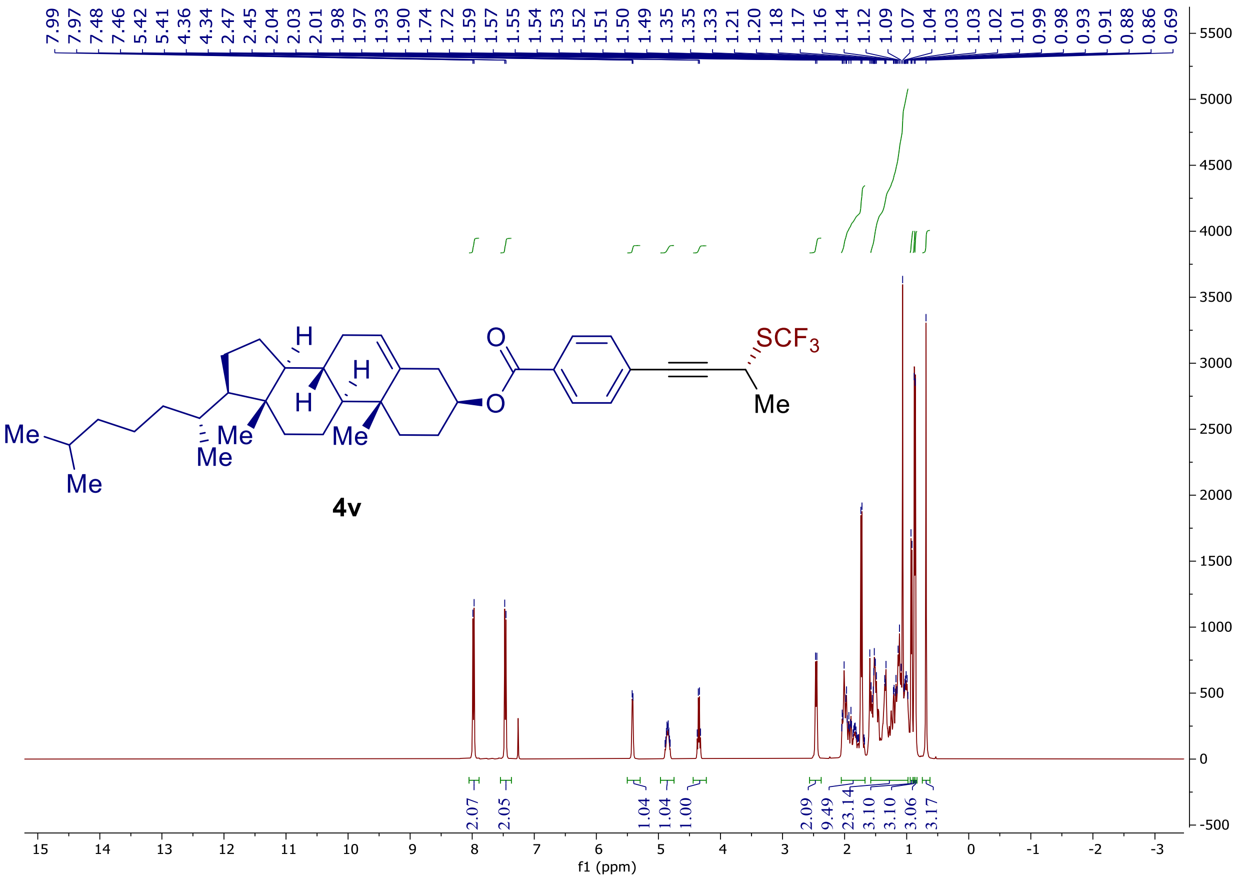

^{19}F NMR:

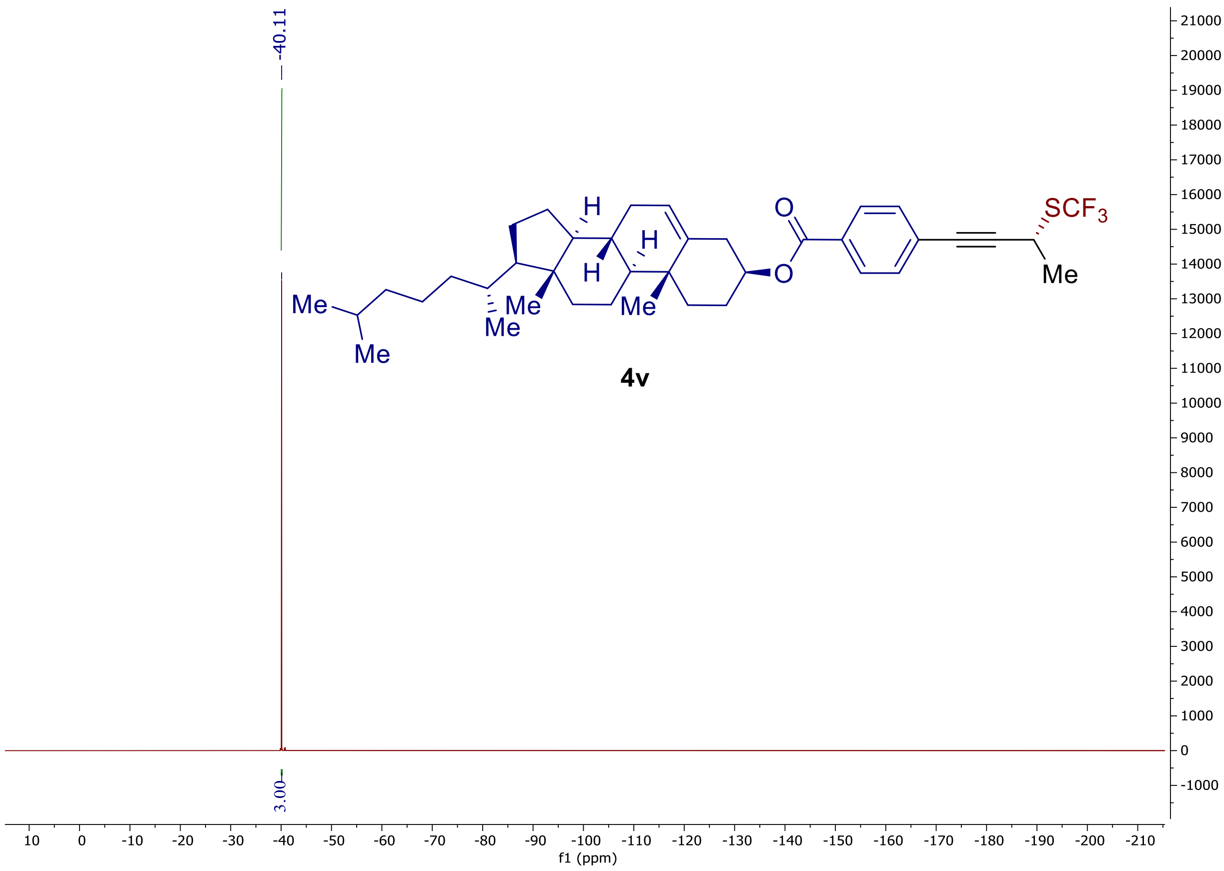

^{13}C NMR:

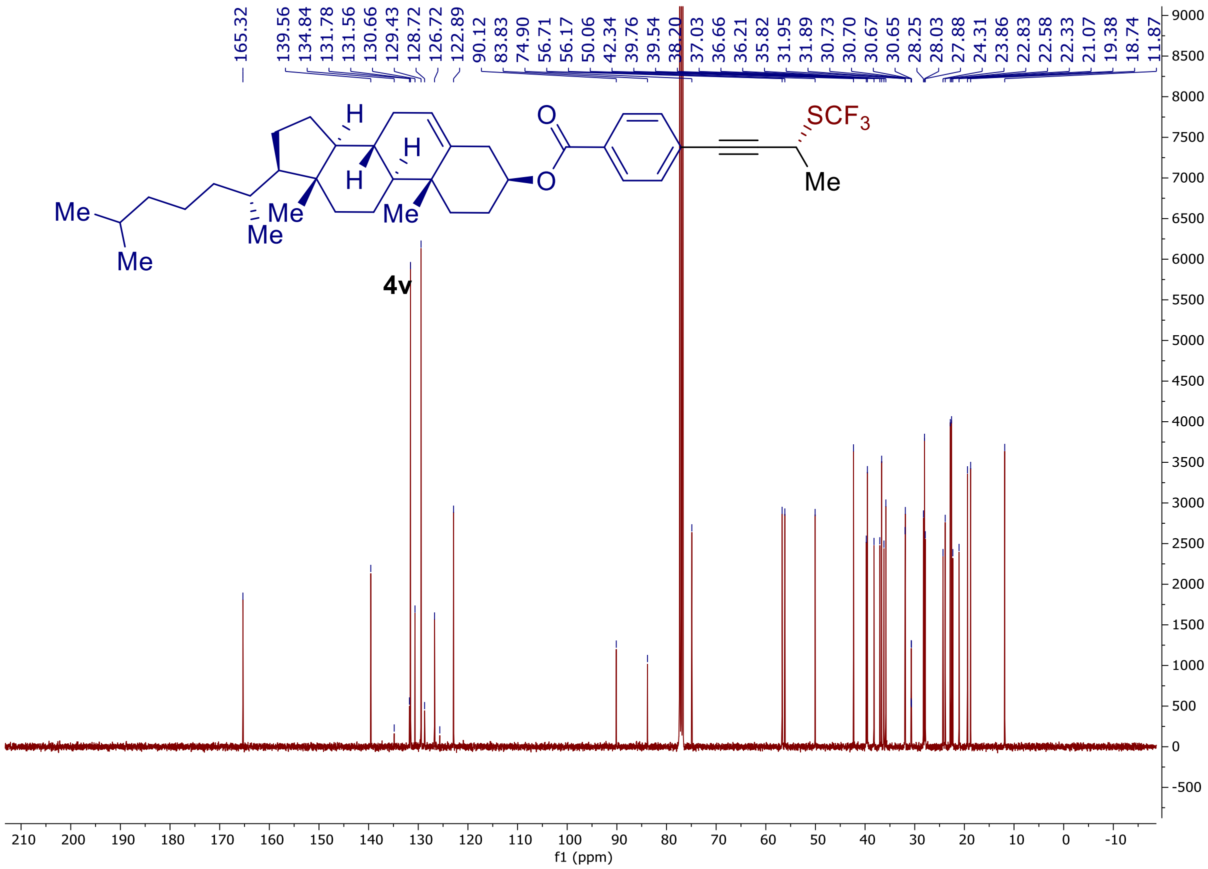

165.32
139.56
134.84
131.78
131.56
130.66
129.43
128.72
126.72
122.89
90.12
83.83
74.90
56.71
56.17
50.06
42.34
39.76
39.54
38.20
37.03
36.66
36.21
35.82
31.95
31.89
30.73
30.70
30.67
30.65
28.25
28.03
27.88
24.31
23.86
22.83
22.58
22.33
21.07
19.38
18.74
11.87
4v
f1 (ppm)

^{1}H NMR:

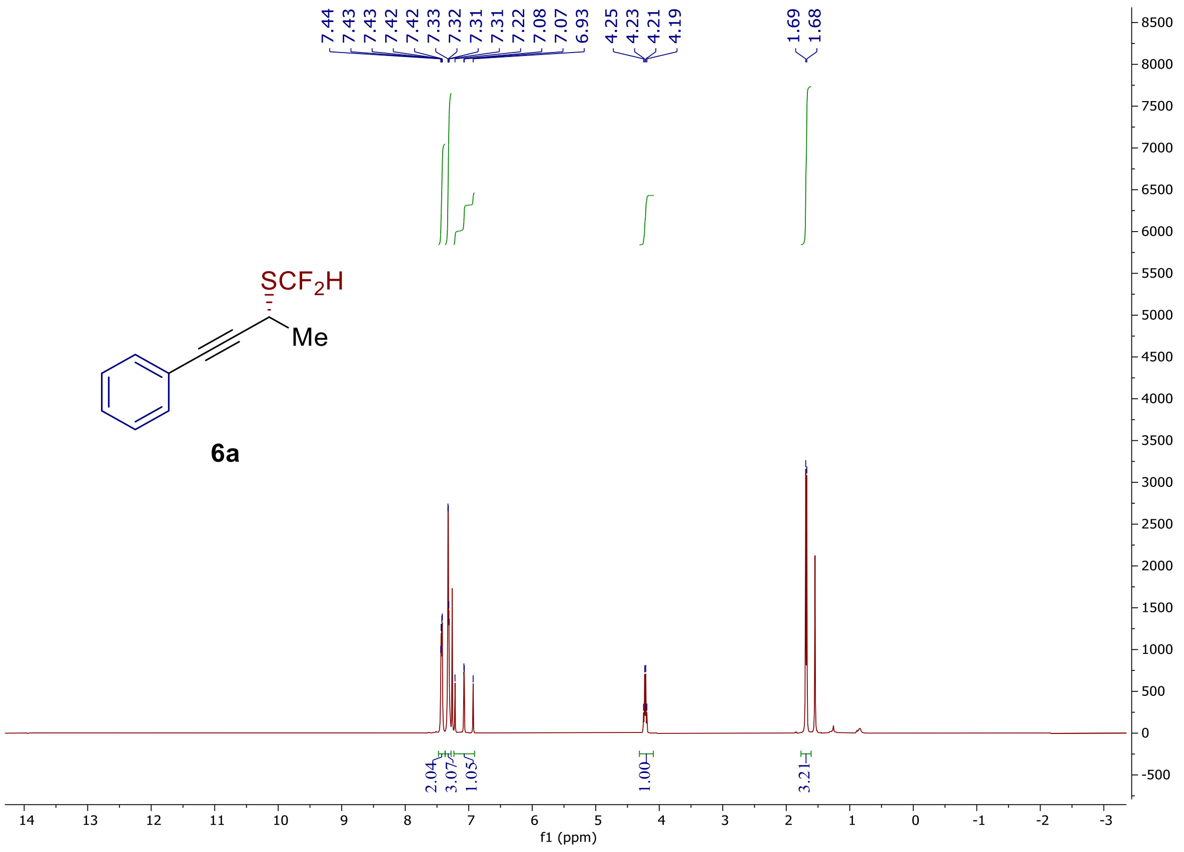

^{19}F NMR:

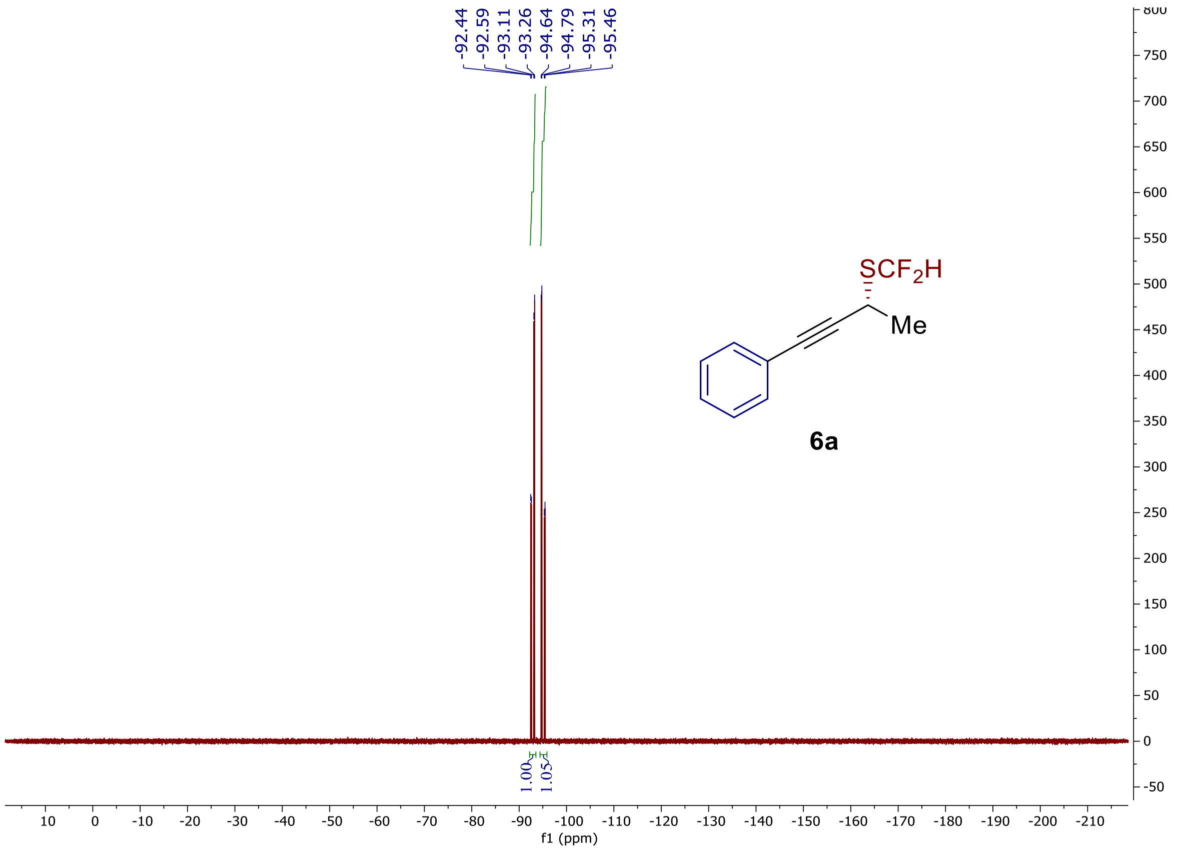

^{13}C NMR:

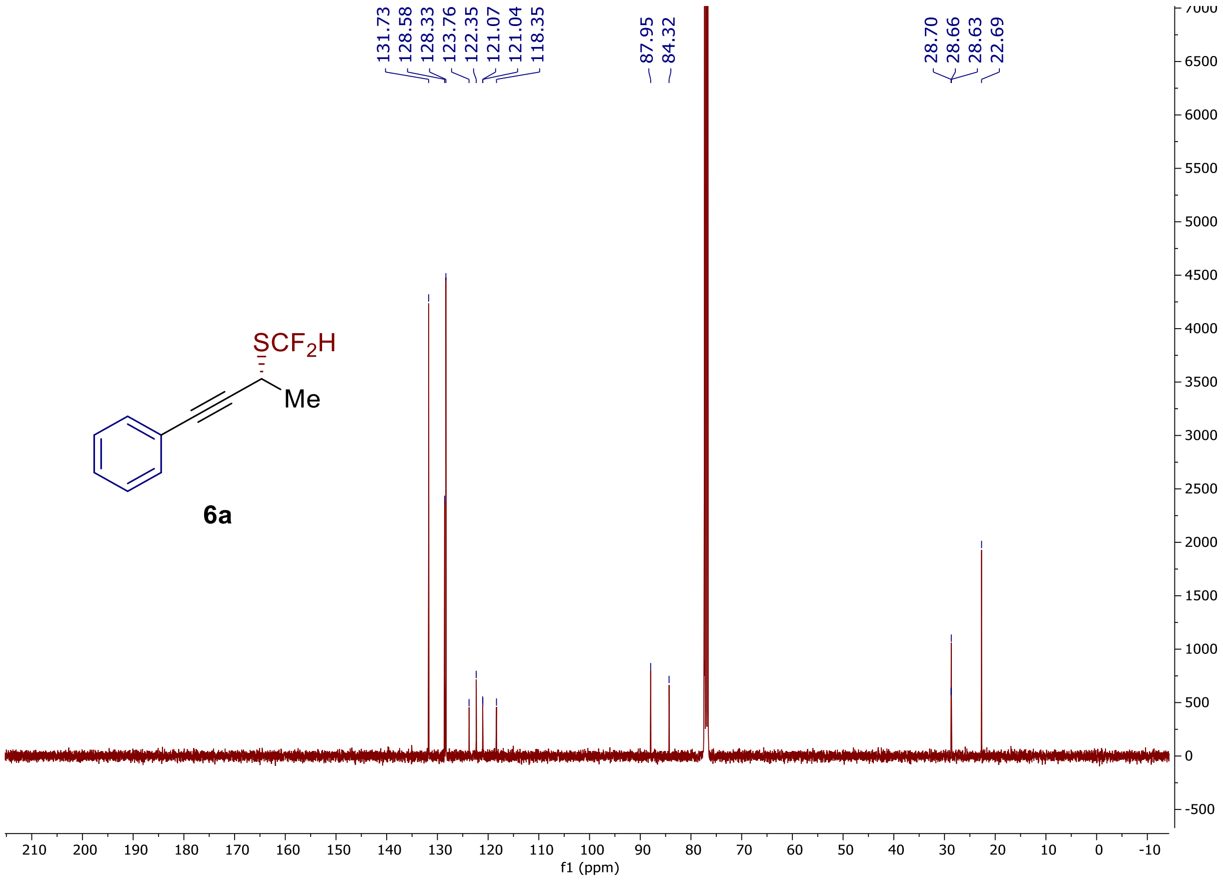
131.73
128.58
128.33
123.76
122.35
121.07
121.04
118.35
87.95
84.32
28.70
28.66
28.63
22.69
SCF2H
Me
6a
f1 (ppm)

^{1}H NMR:

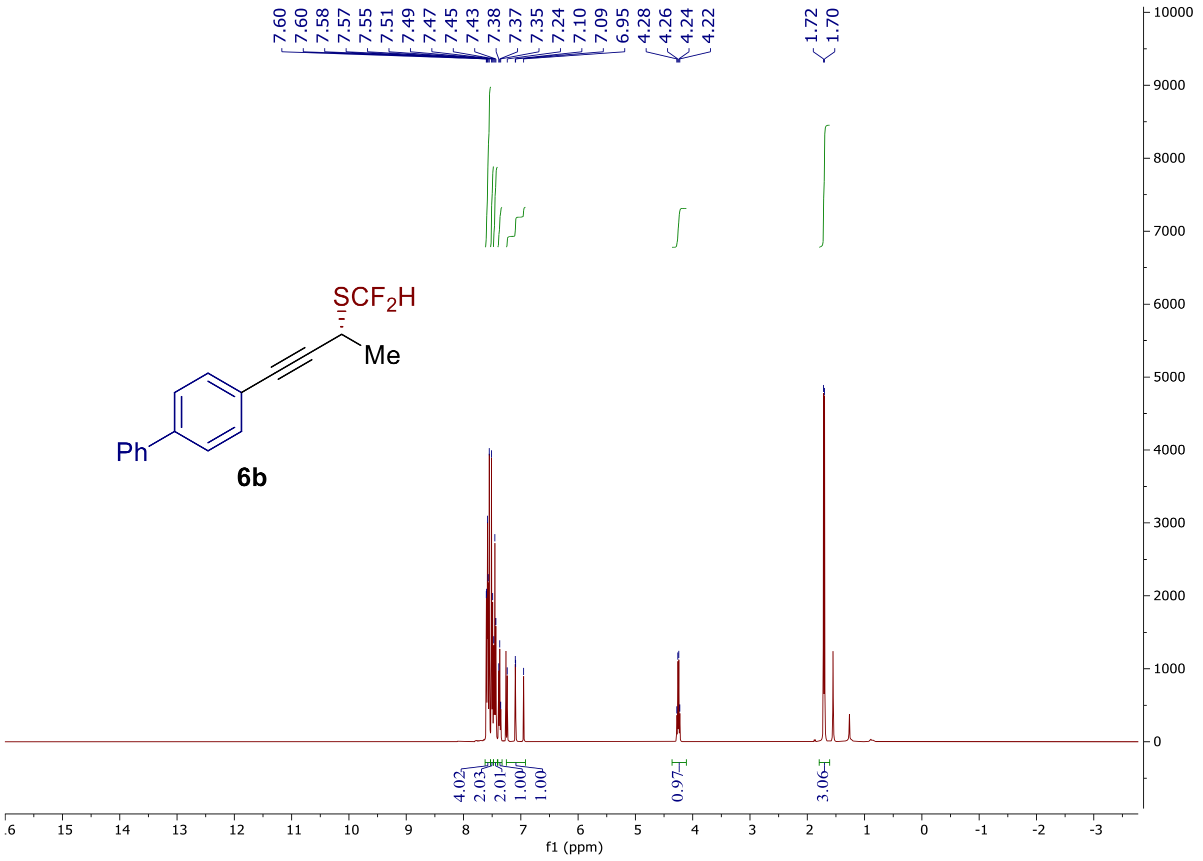

^{19}F NMR:

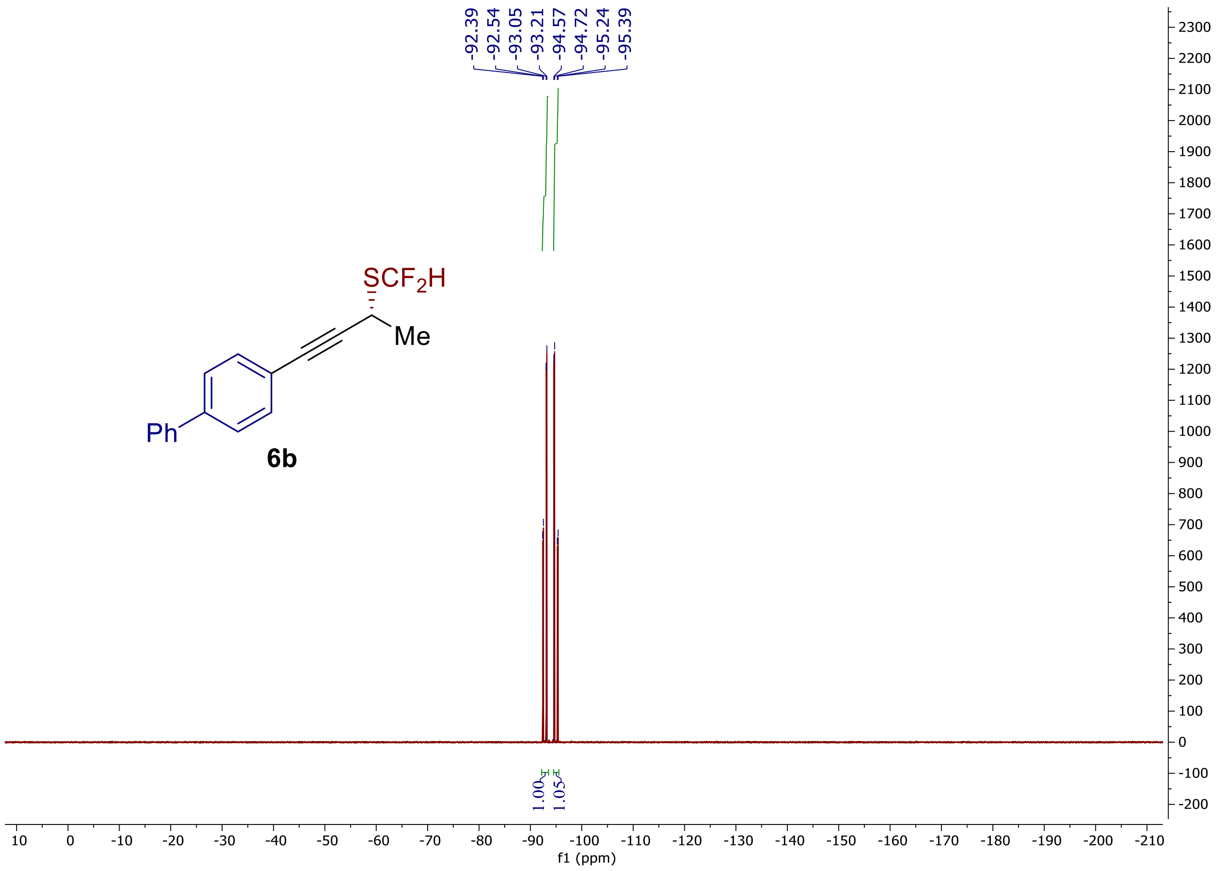

^{13}C NMR:

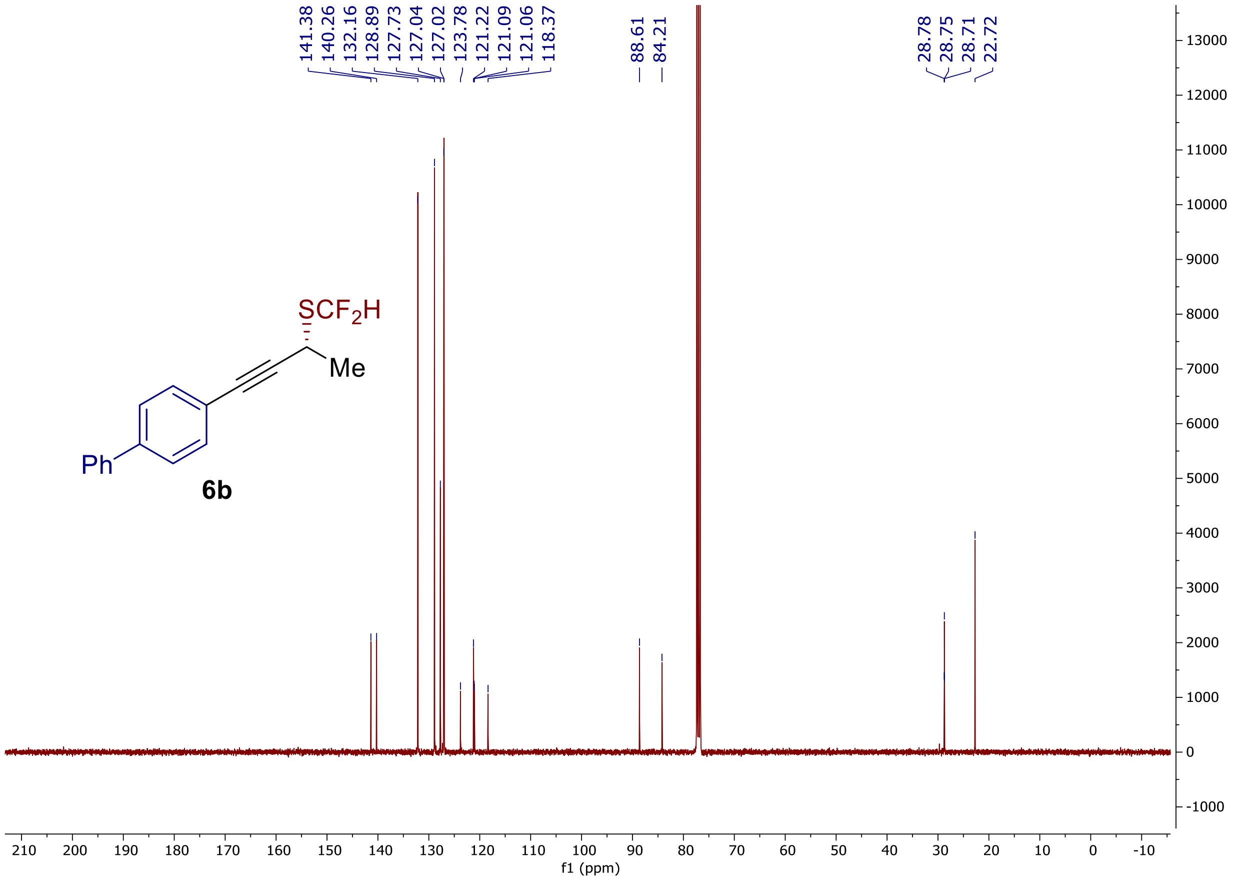

141.38
140.26
132.16
128.89
127.73
127.04
127.02
123.78
121.22
121.09
121.06
118.37
88.61
84.21
28.78
28.75
28.71
22.72
SCF2H
Me
Ph
6b
f1 (ppm)

^{1}H NMR:

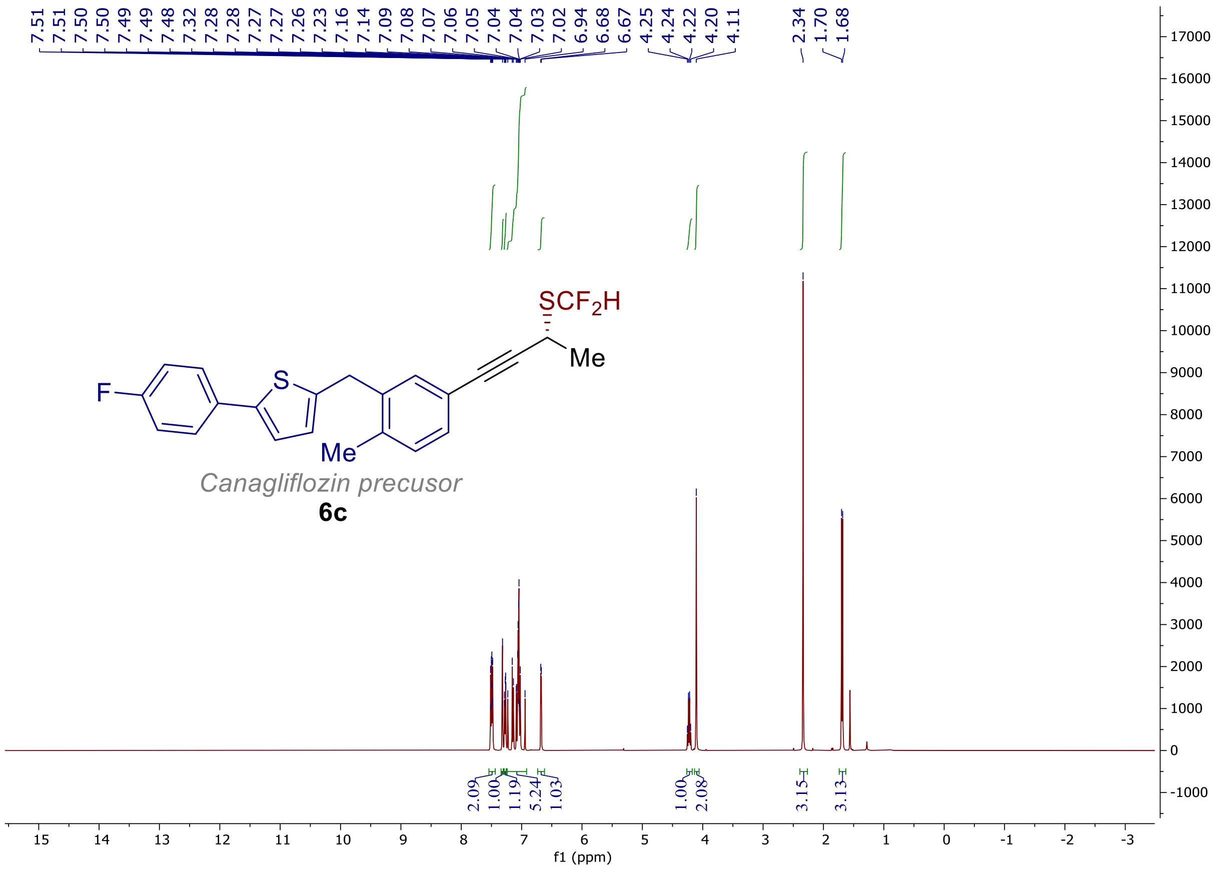

^{19}F NMR:

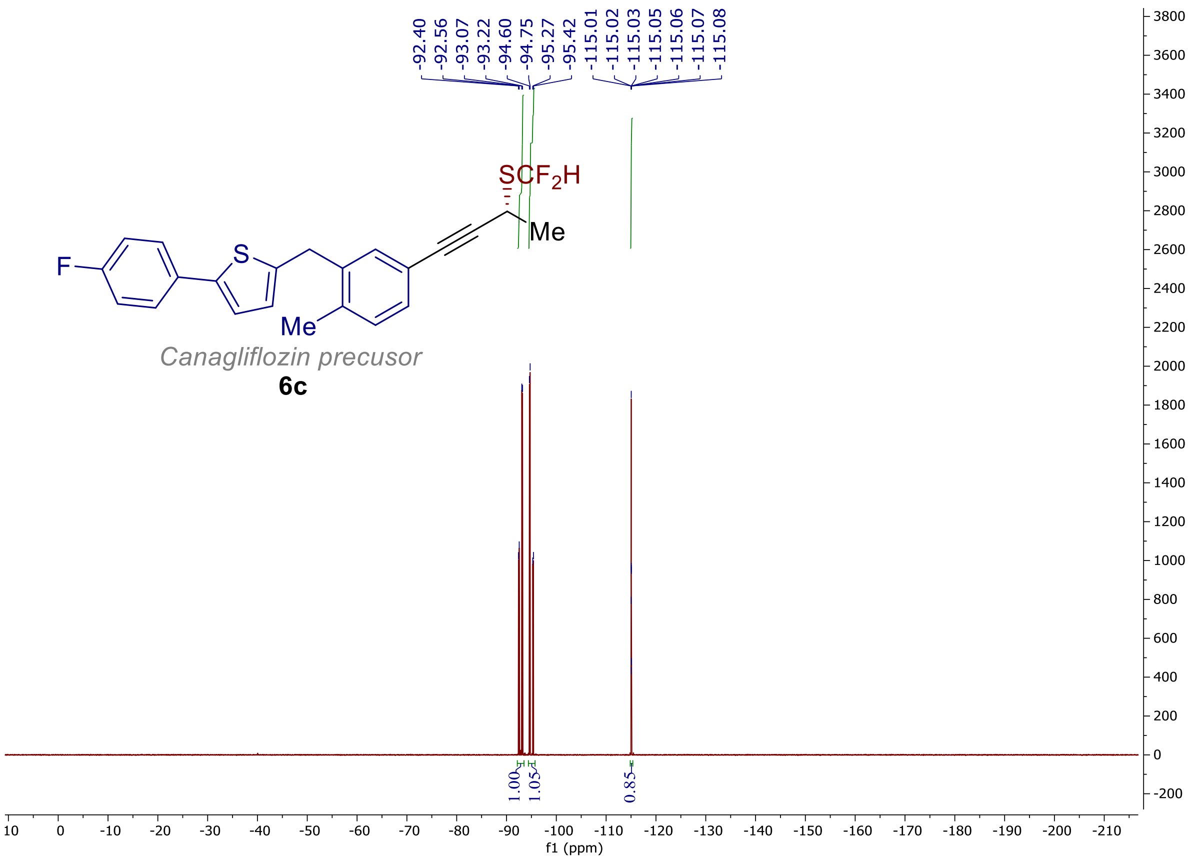

^{13}C NMR:

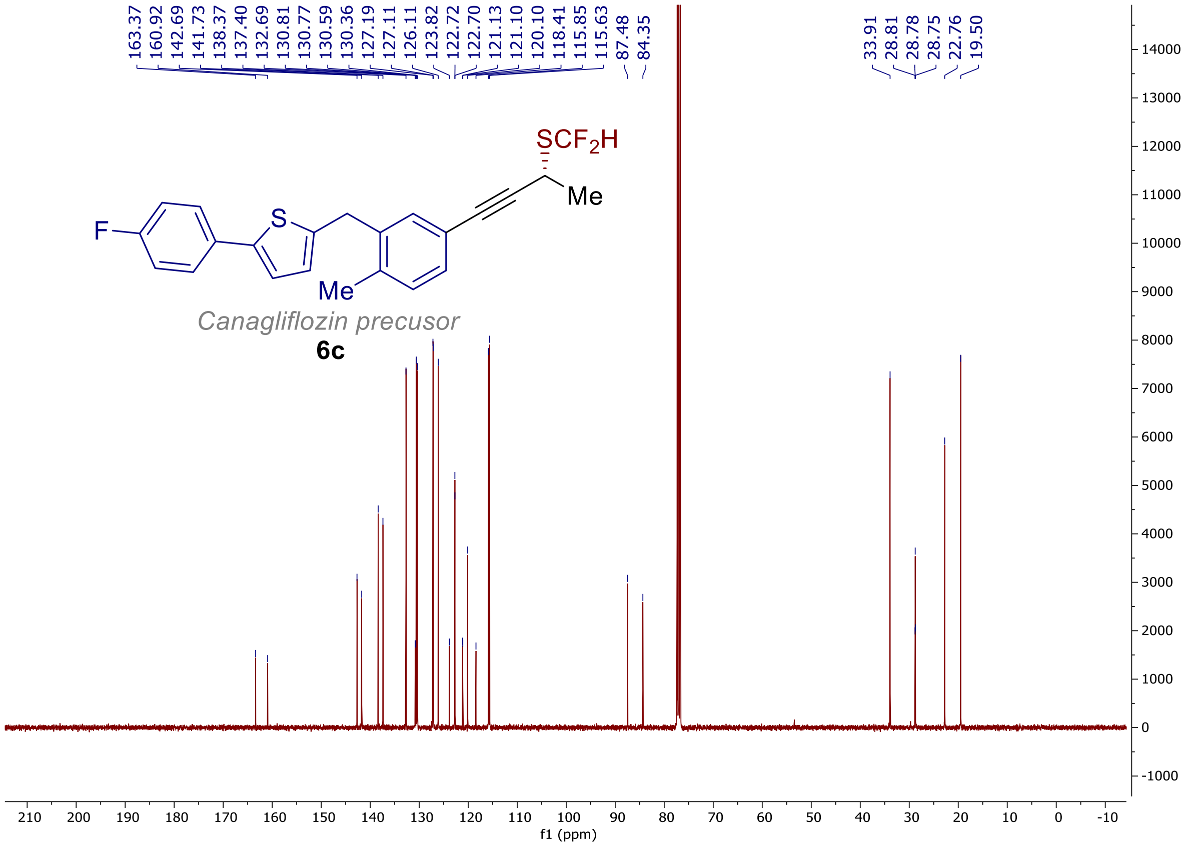
163.37
160.92
142.69
141.73
138.37
137.40
132.69
130.81
130.77
130.59
130.36
127.19
127.11
126.11
123.82
122.72
122.70
121.13
121.10
120.10
118.41
115.85
115.63
87.48
84.35
33.91
28.81
28.78
28.75
22.76
19.50
SCF2H
Me
F
S
Me
Canagliflozin precusor
6c
f1 (ppm)

^{1}H NMR:

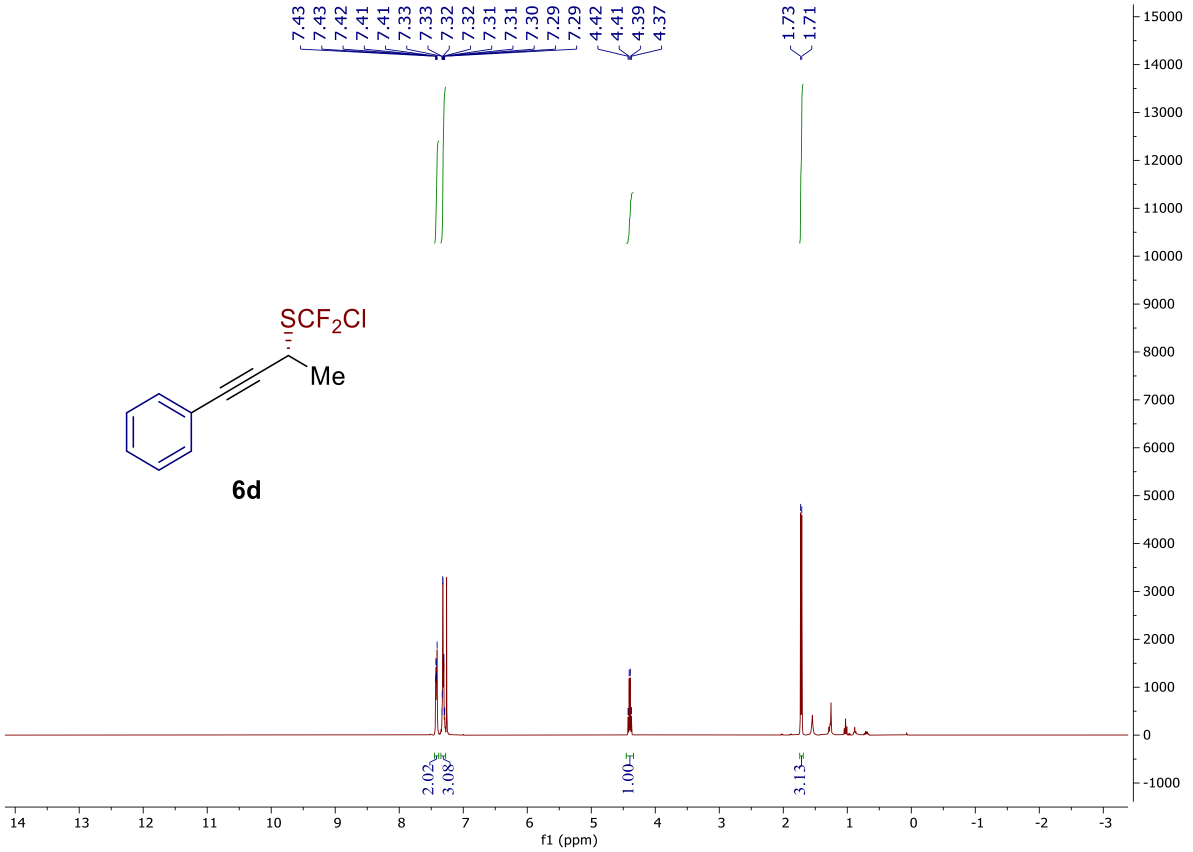

^{19}F NMR:

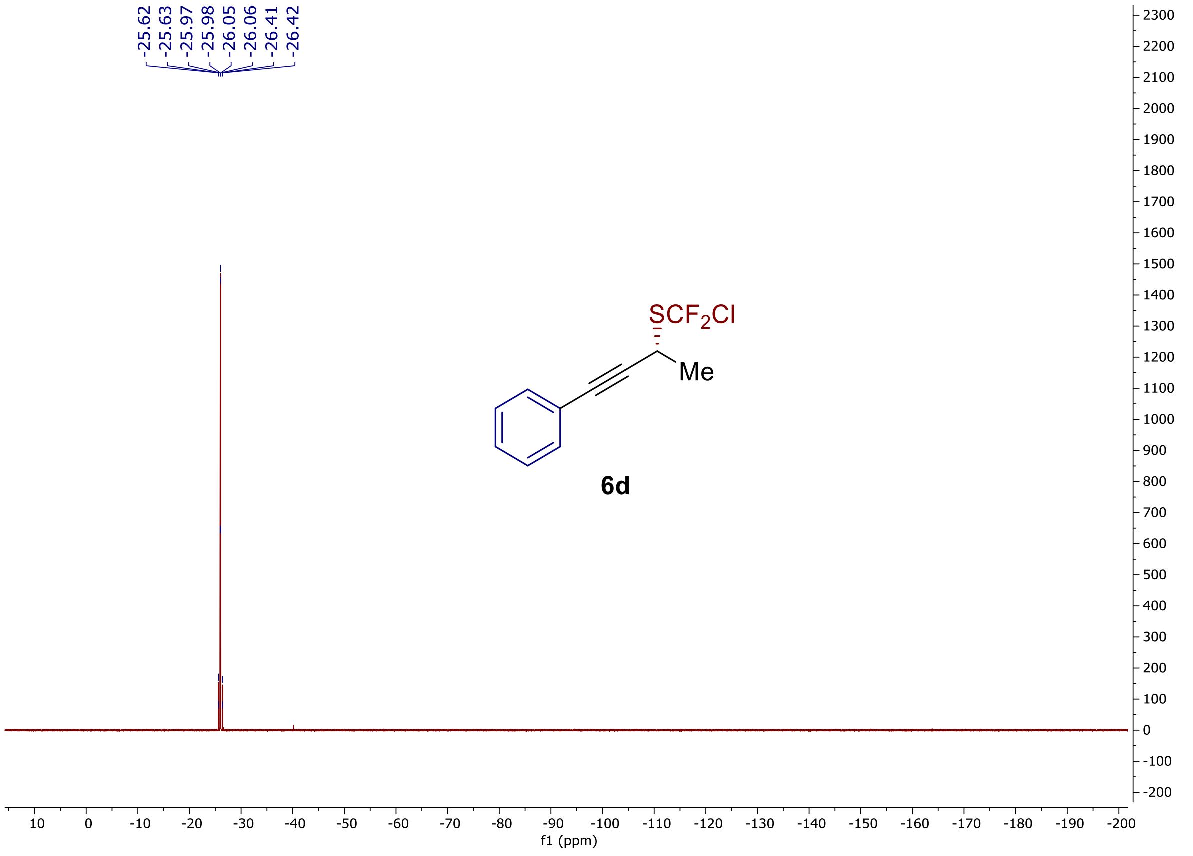

^{13}C NMR:

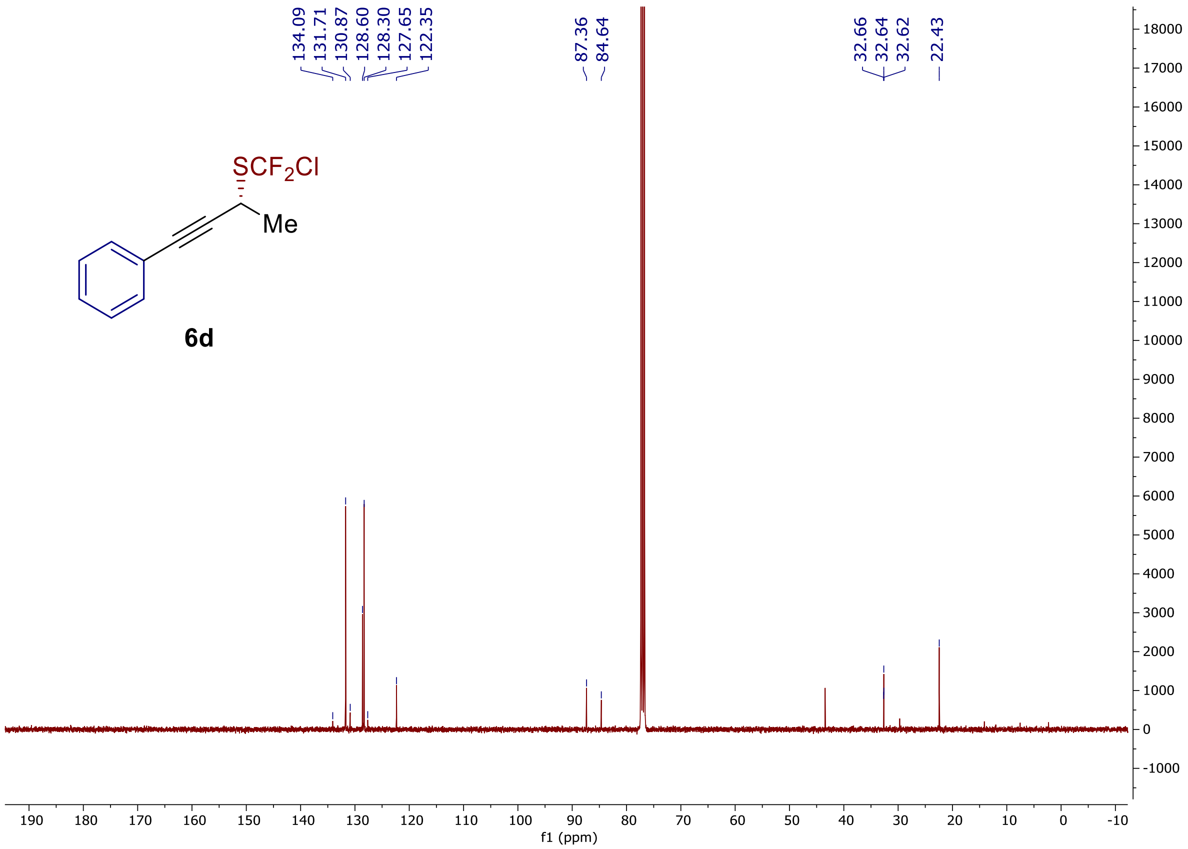
134.09
131.71
130.87
128.60
128.30
127.65
122.35
87.36
84.64
32.66
32.64
32.62
22.43
SCF2Cl
Me
6d
f1 (ppm)

^{1}H NMR:

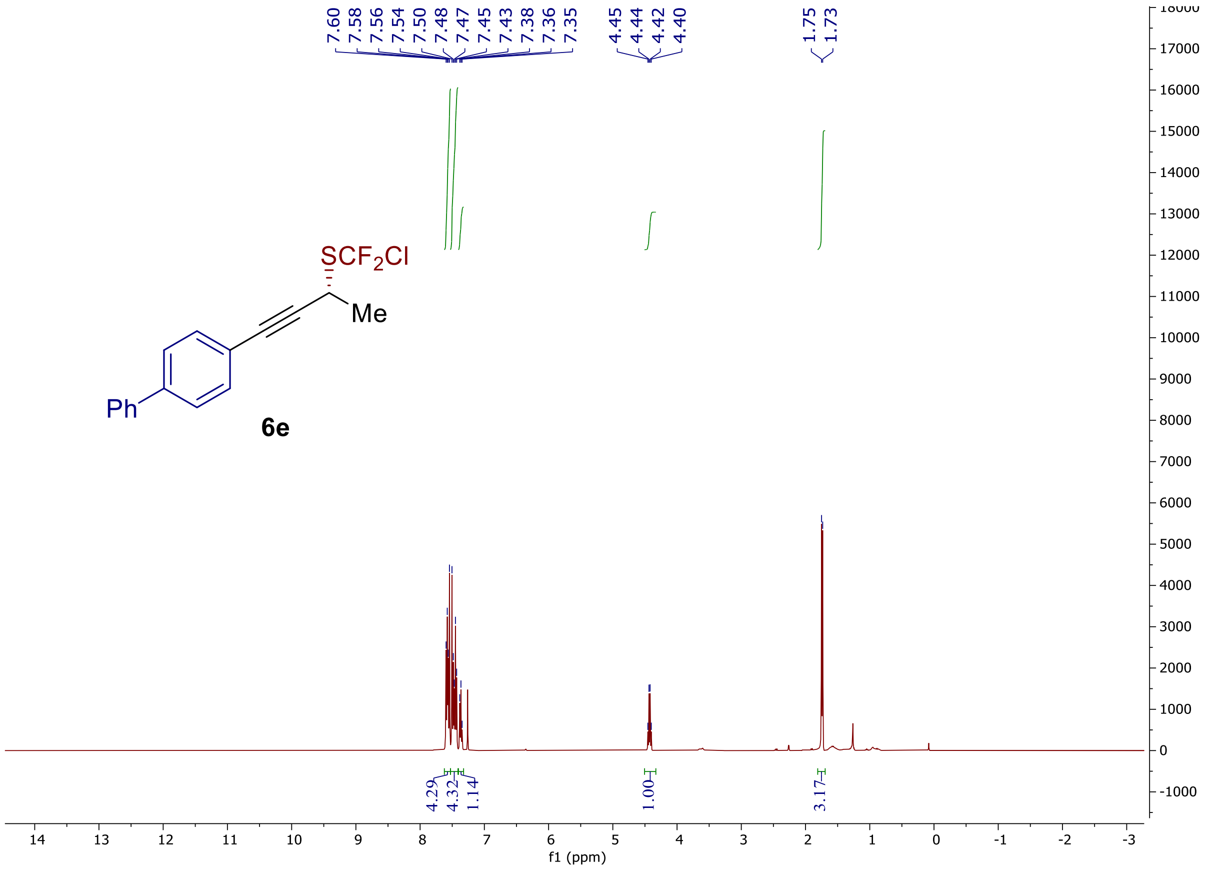

^{19}F NMR:

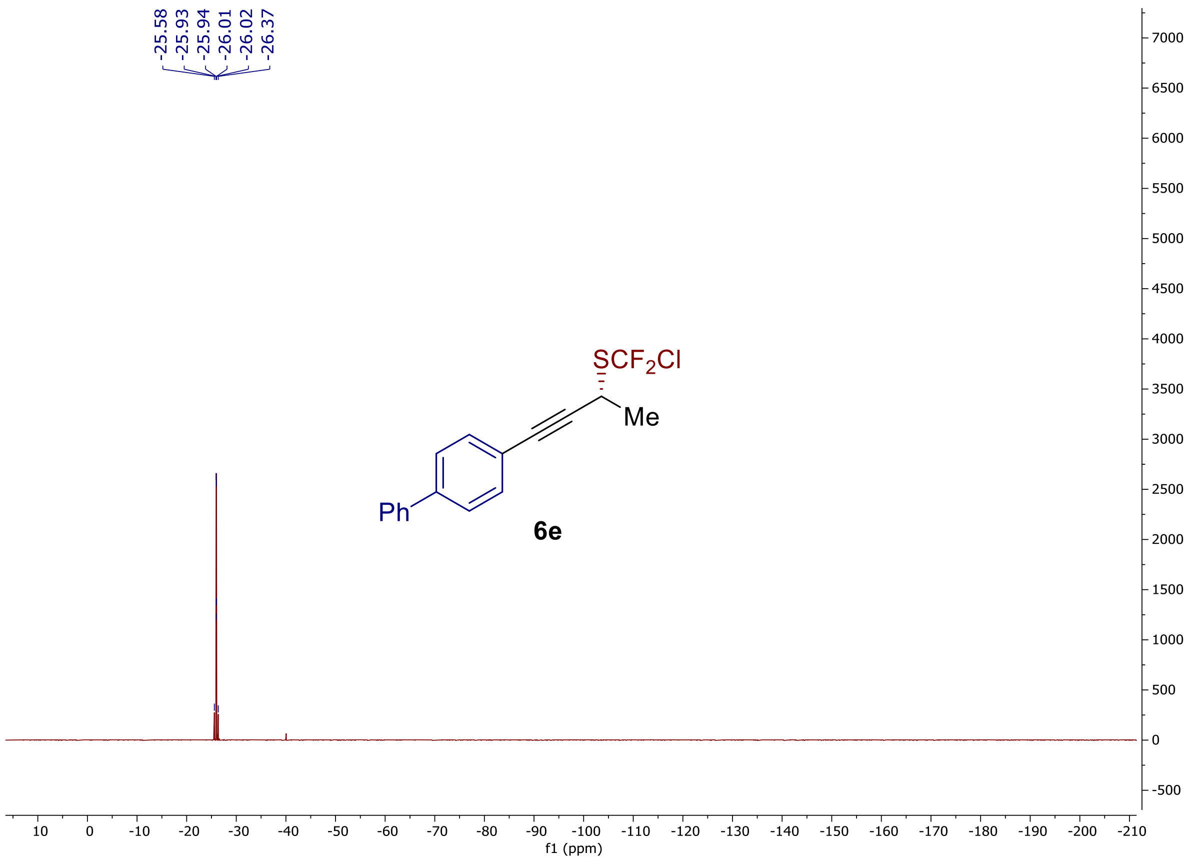

^{13}C NMR:

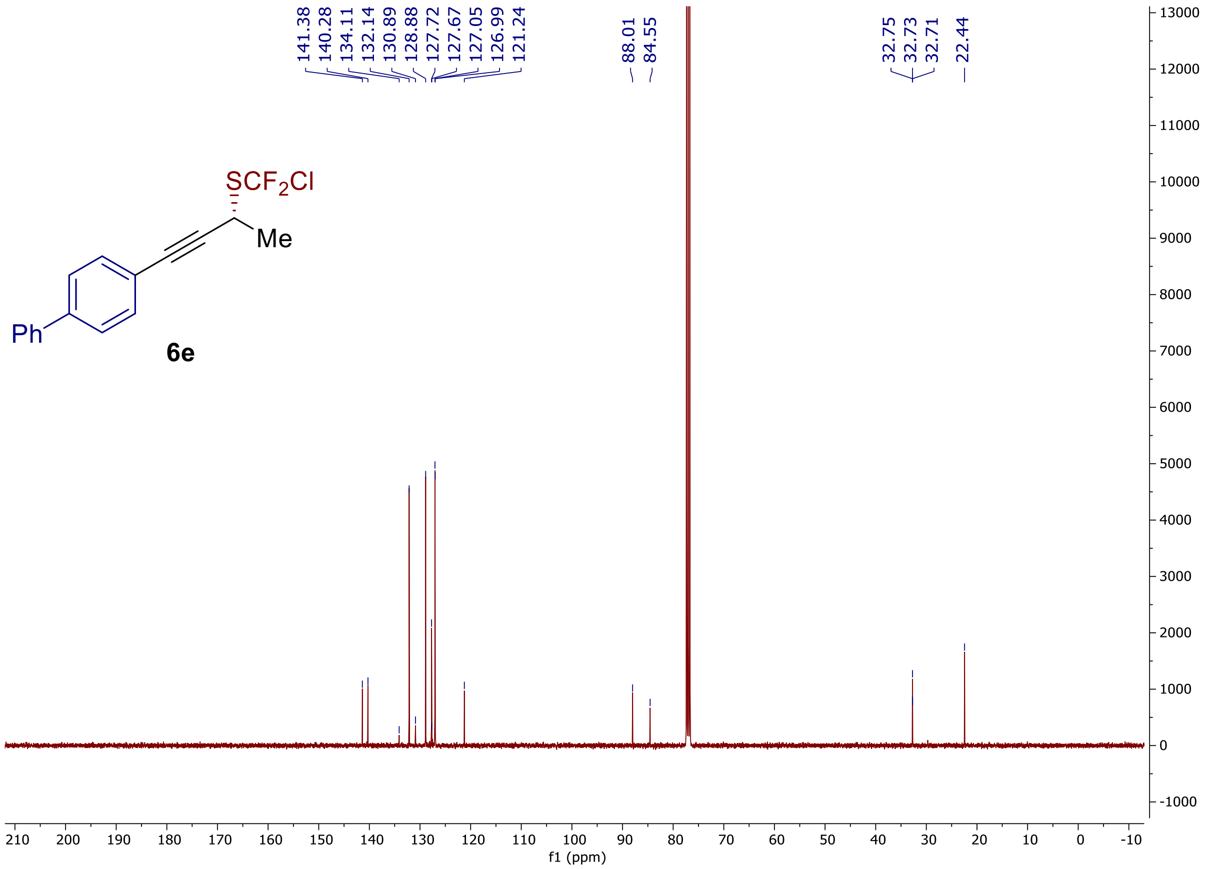
SCF2Cl
Me
Ph
141.38
140.28
134.11
132.14
130.89
128.88
127.72
127.67
127.05
126.99
121.24
88.01
84.55
32.75
32.73
32.71
22.44
f1 (ppm)

6e

^{1}H NMR:

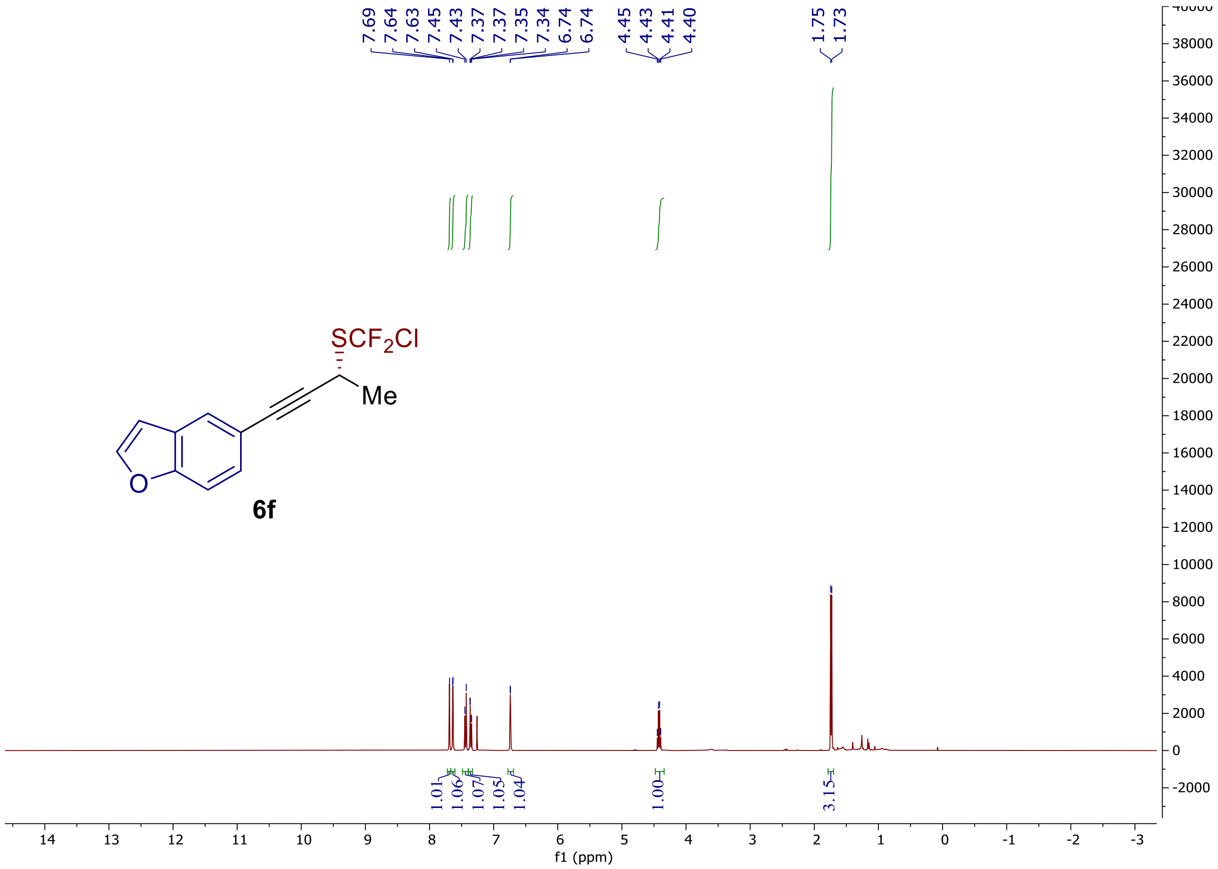

^{19}F NMR:

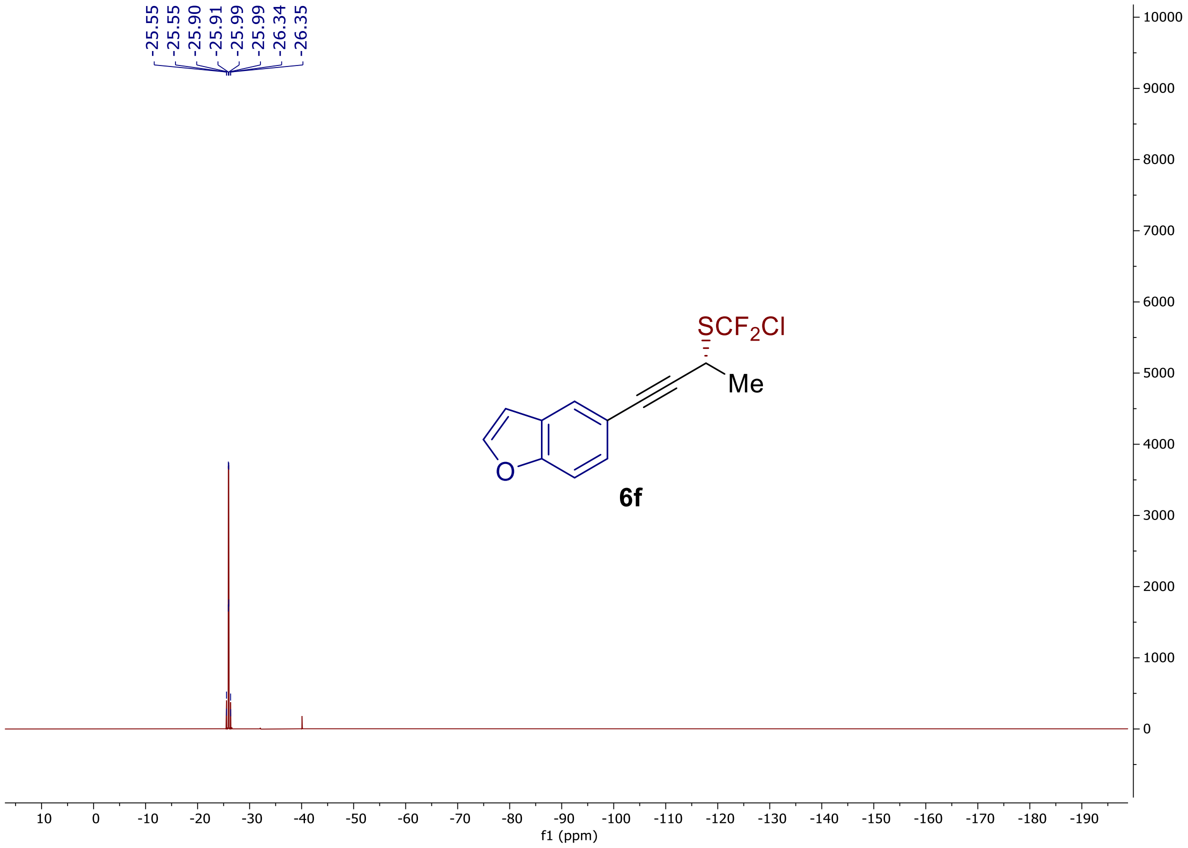

^{13}C NMR:

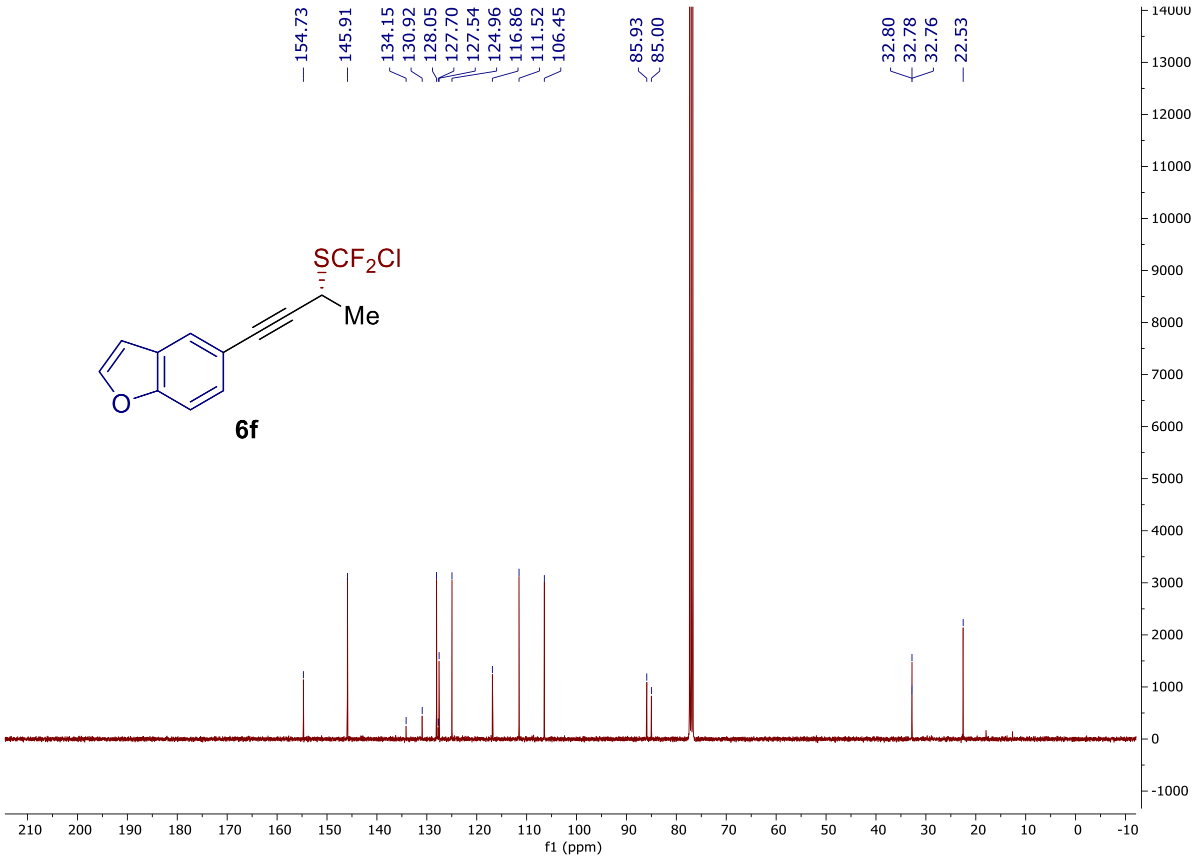
154.73
145.91
134.15
130.92
128.05
127.70
127.54
124.96
116.86
111.52
106.45
85.93
85.00
32.80
32.78
32.76
22.53
SCF2Cl
Me
O
6f
f1 (ppm)

^{1}H NMR:

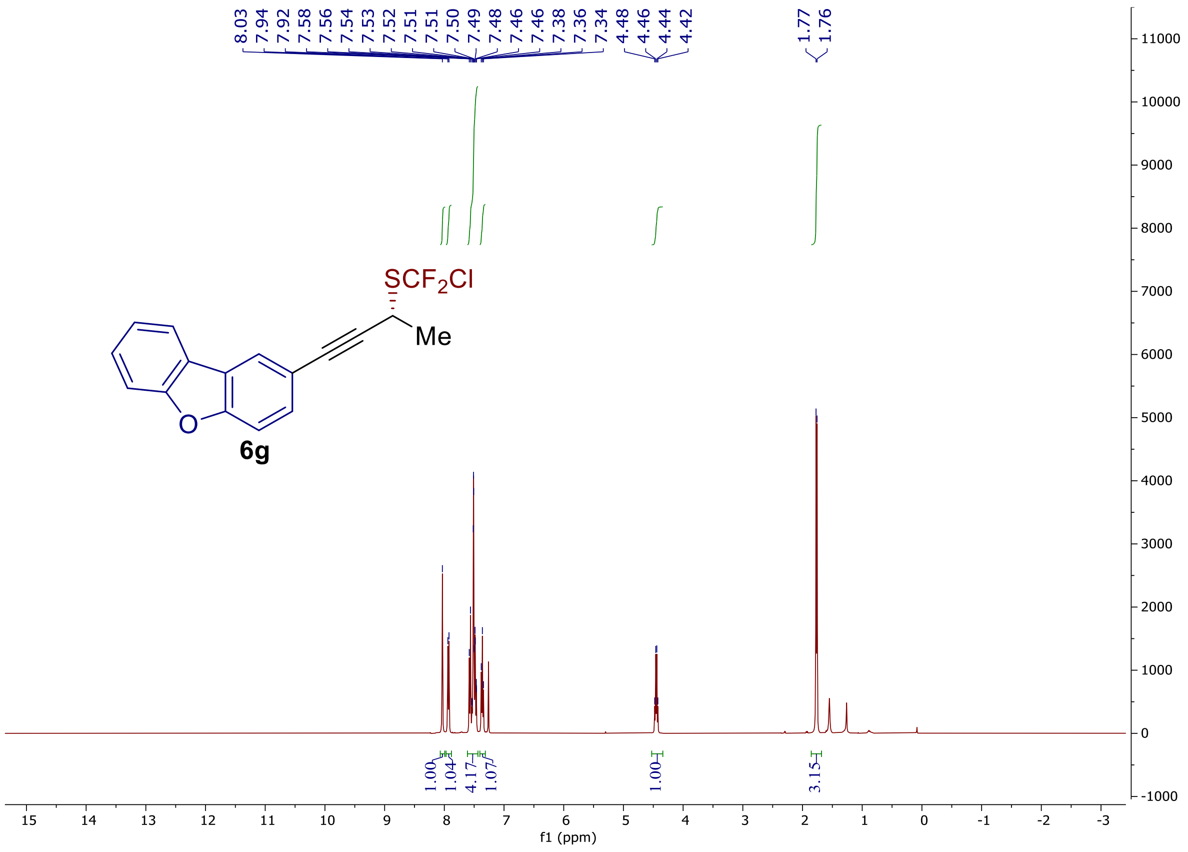

^{19}F NMR:

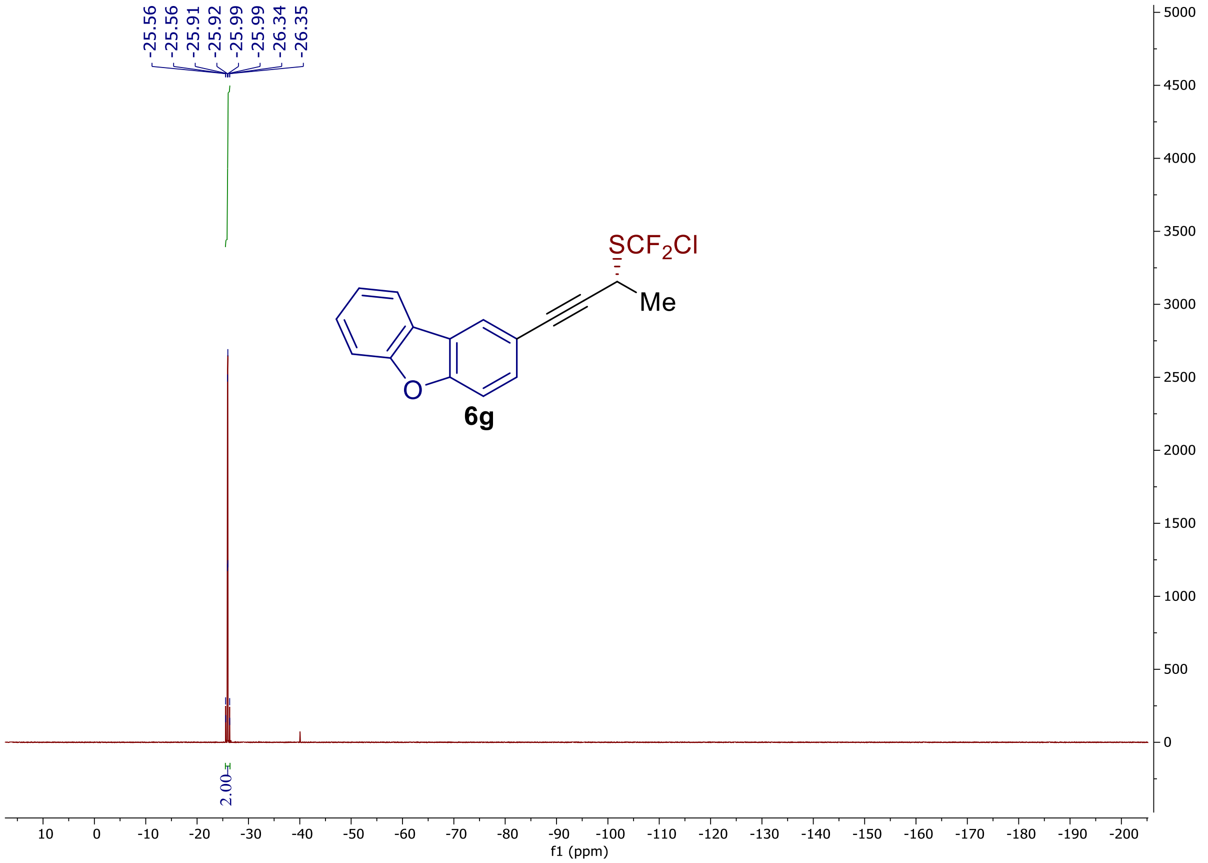

^{13}C NMR:

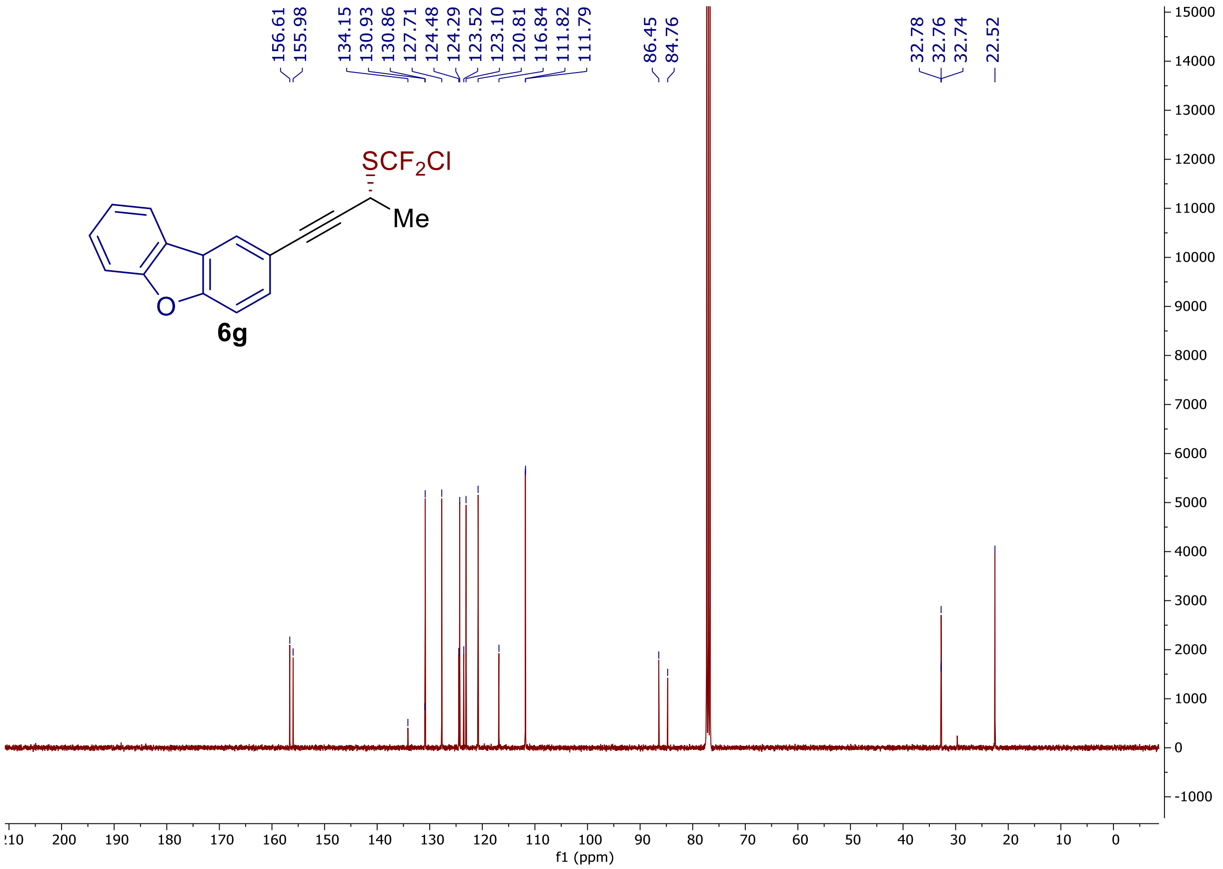

156.61
155.98
134.15
130.93
130.86
127.71
124.48
124.29
123.52
123.10
120.81
116.84
111.82
111.79
86.45
84.76
32.78
32.76
32.74
22.52
SCF2Cl
Me
O
6g
f1 (ppm)

^{1}H NMR:

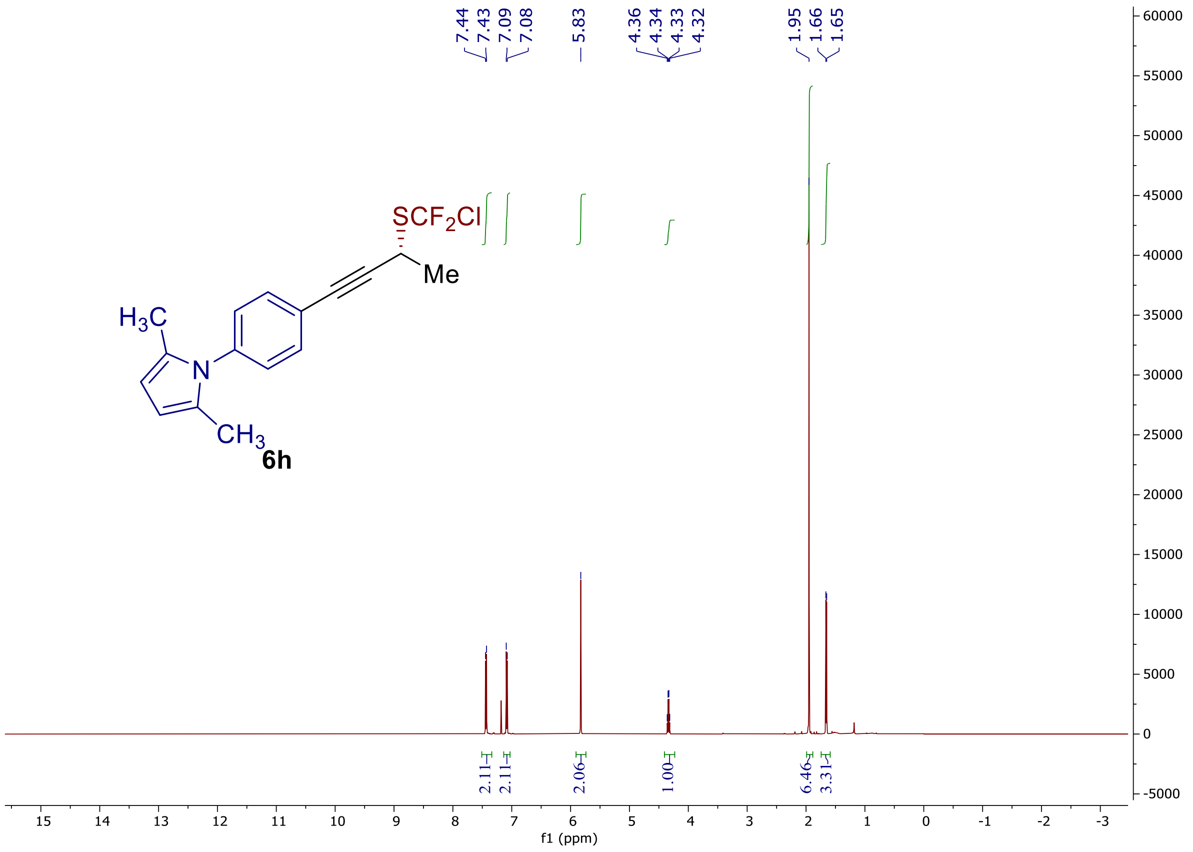

^{19}F NMR:

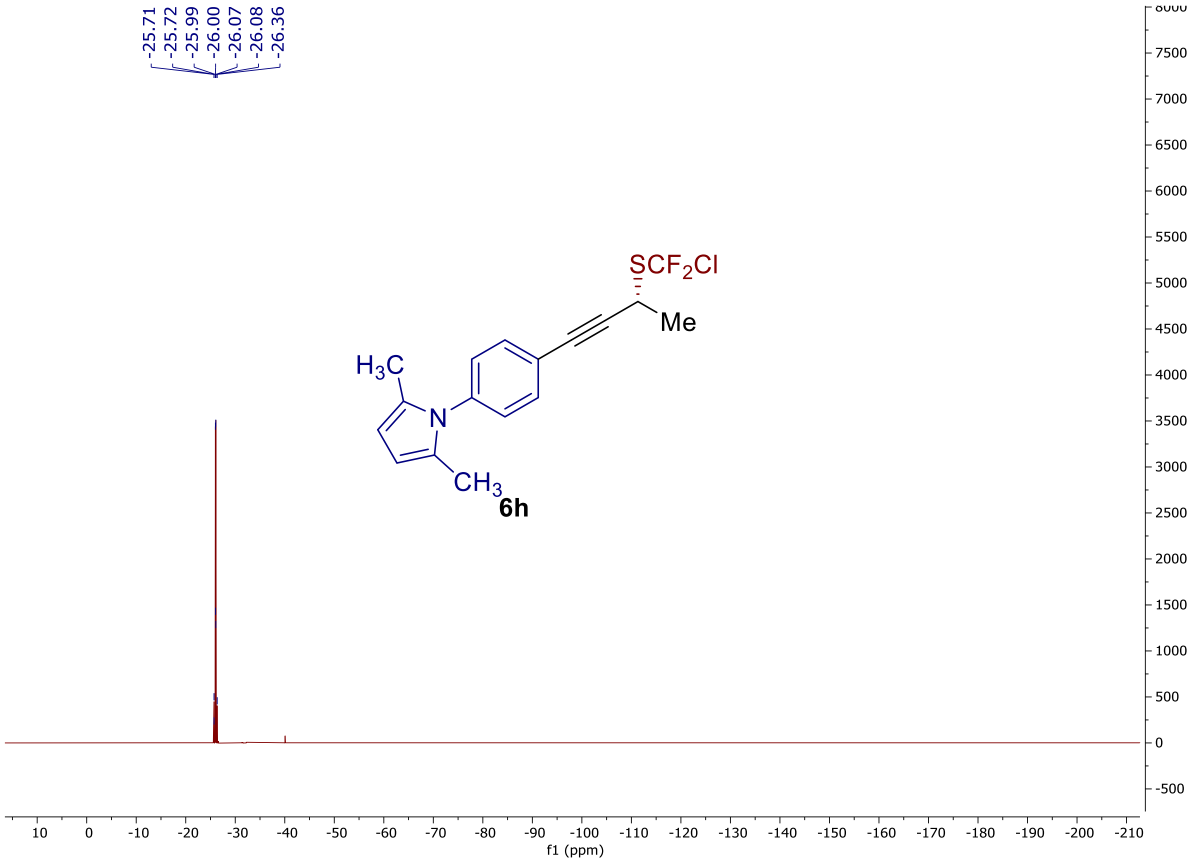

^{13}C NMR:

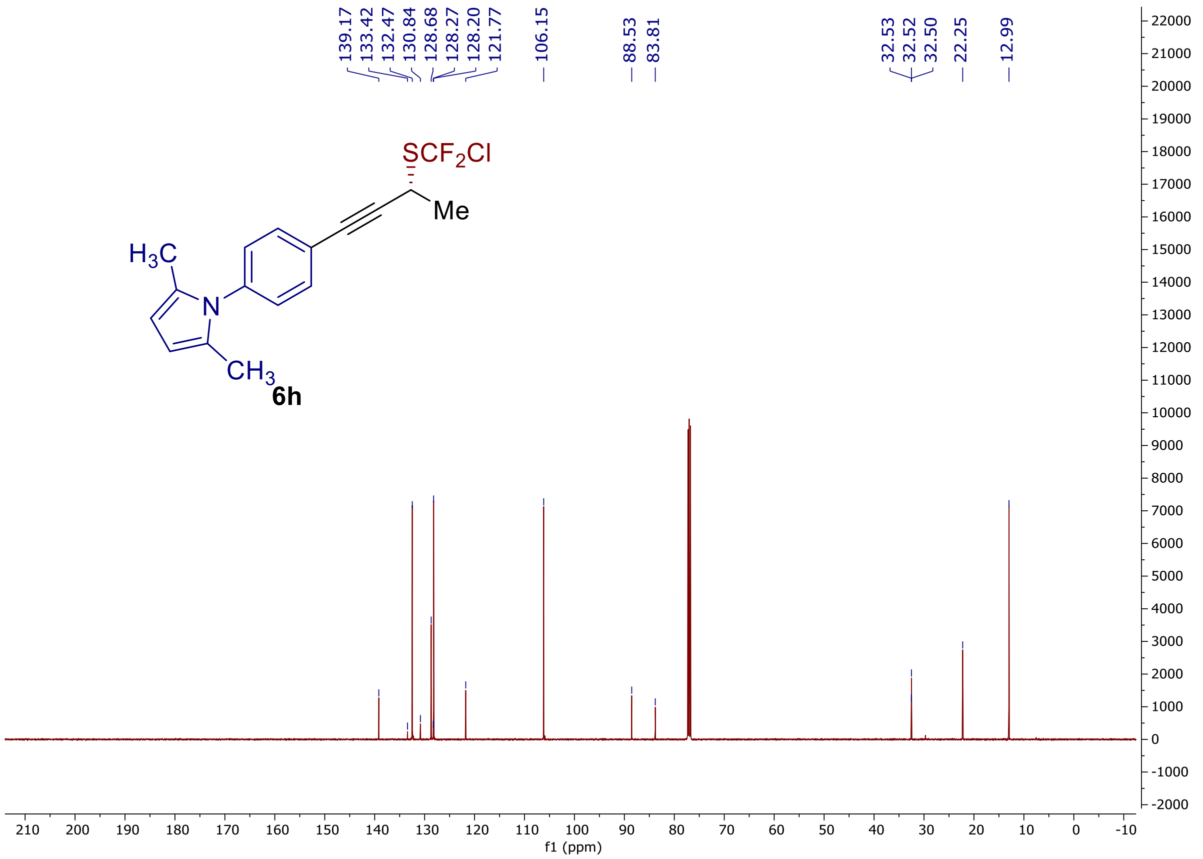
139.17
133.42
132.47
130.84
128.68
128.27
128.20
121.77
106.15
88.53
83.81
32.53
32.52
32.50
22.25
12.99
SCF2Cl
Me
H3C
N
CH3
6h
f1 (ppm)

^{1}H NMR:

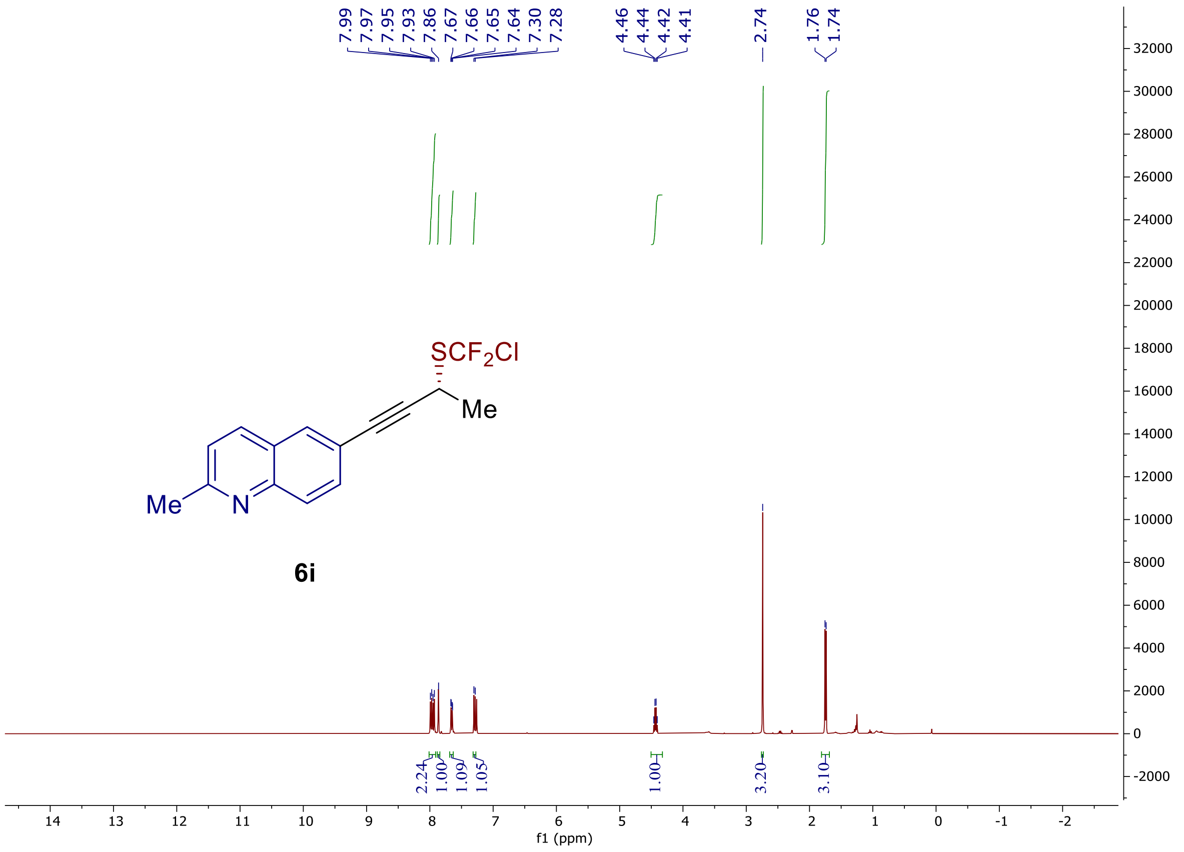

^{19}F NMR:

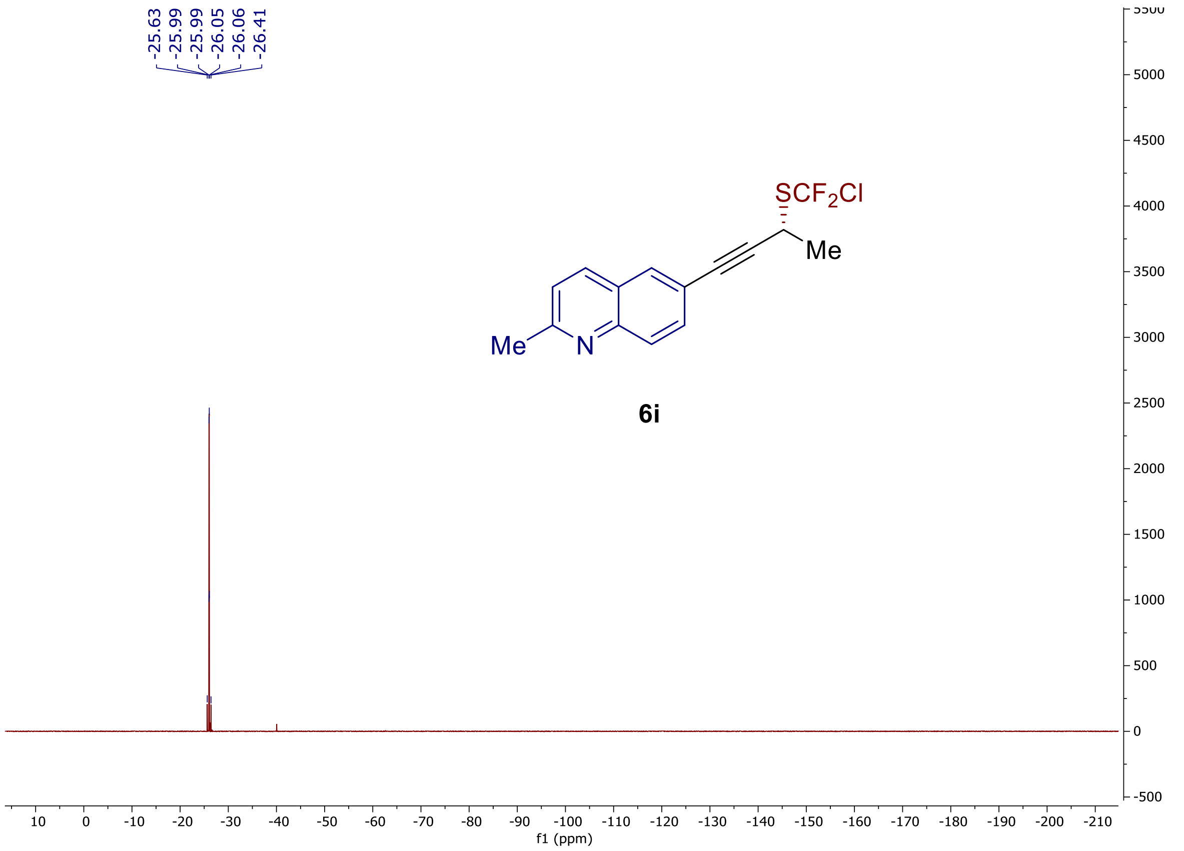

^{13}C NMR:

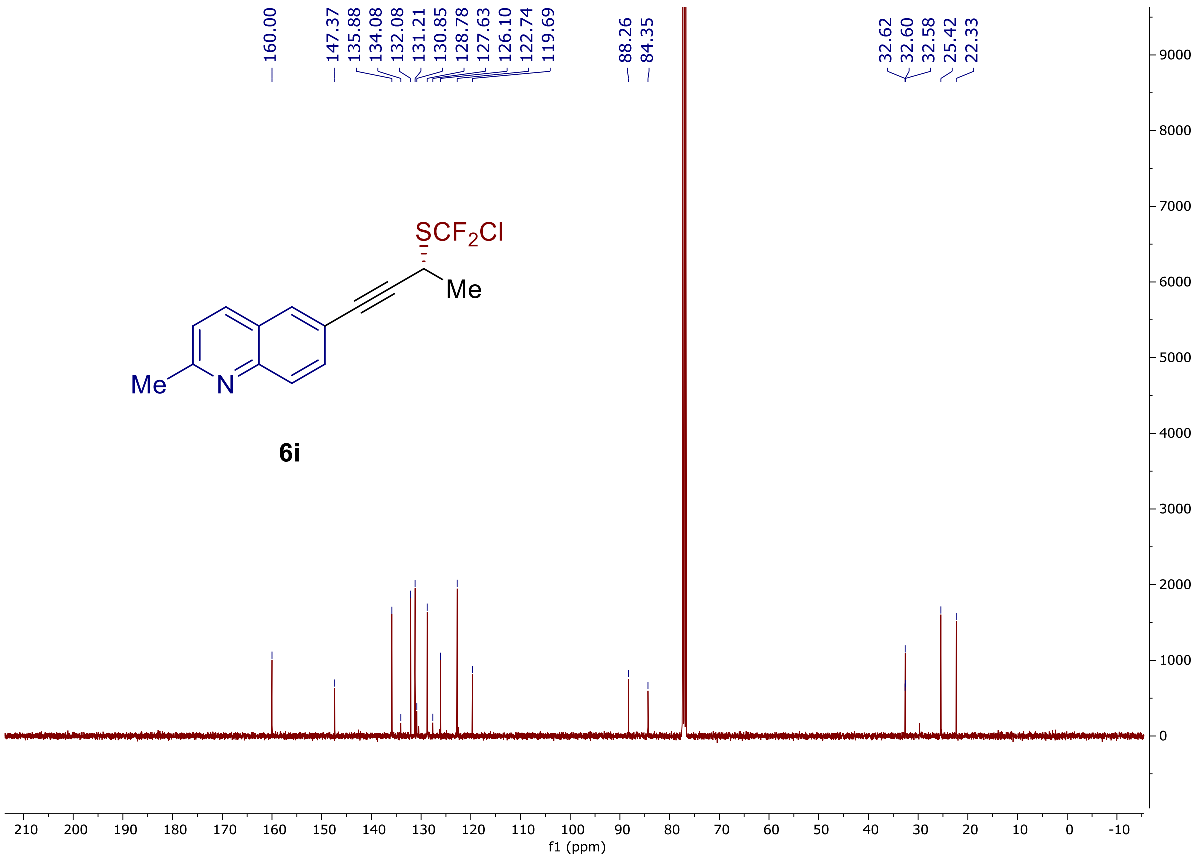

160.00
147.37
135.88
134.08
132.08
131.21
130.85
128.78
127.63
126.10
122.74
119.69
88.26
84.35
32.62
32.60
32.58
25.42
22.33
SCF2Cl
Me
Me
N
6i
9000
8000
7000
6000
5000
4000
3000
2000
1000
0
210 200 190 180 170 160 150 140 130 120 110 100 90 80 70 60 50 40 30 20 10 0 -10
f1 (ppm)

^{1}H NMR:

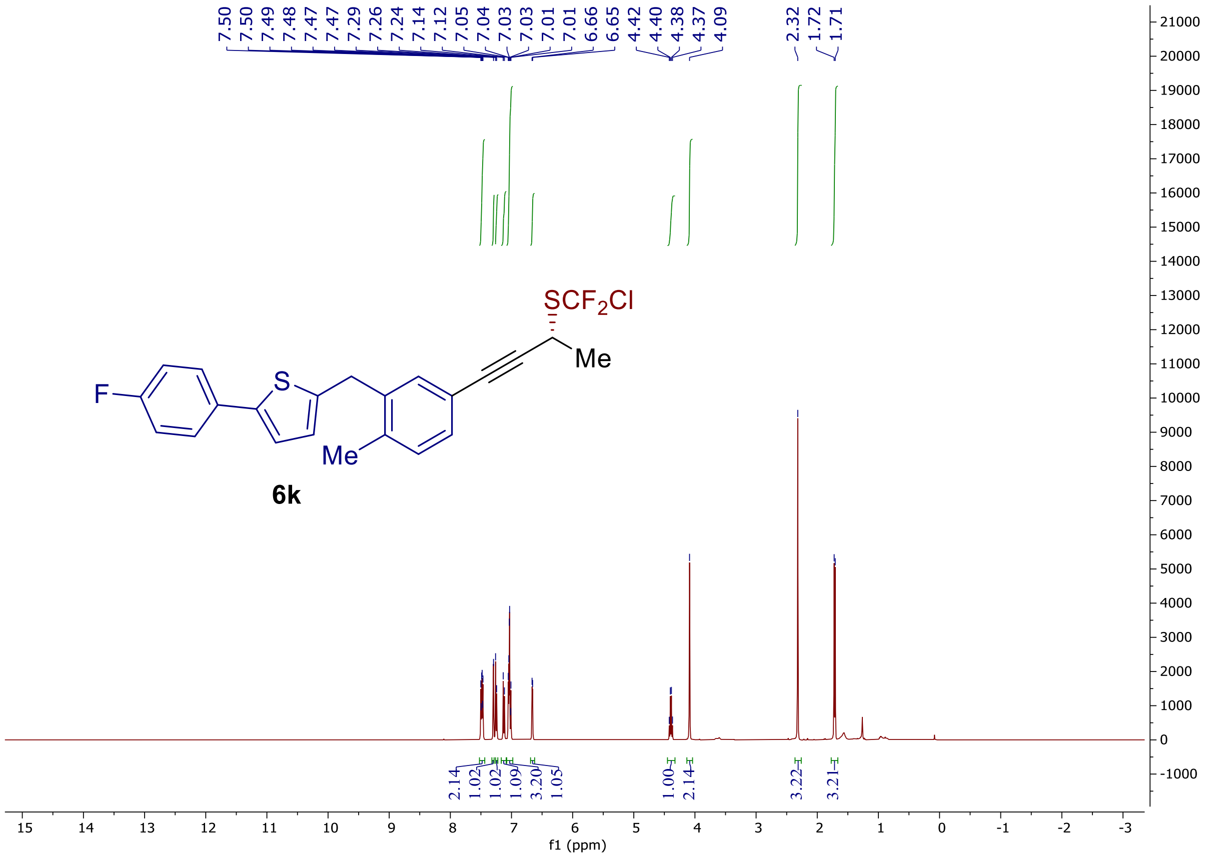

^{19}F NMR:

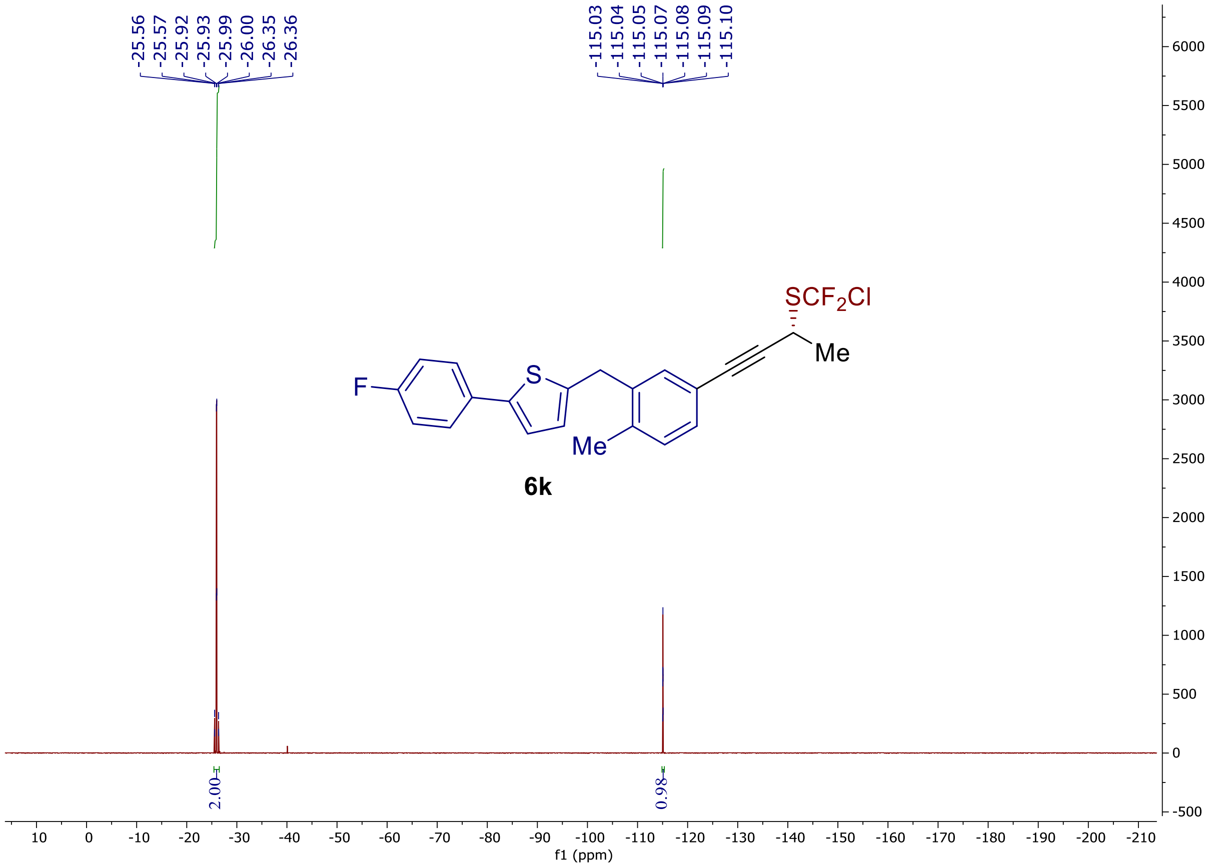

^{13}C NMR:

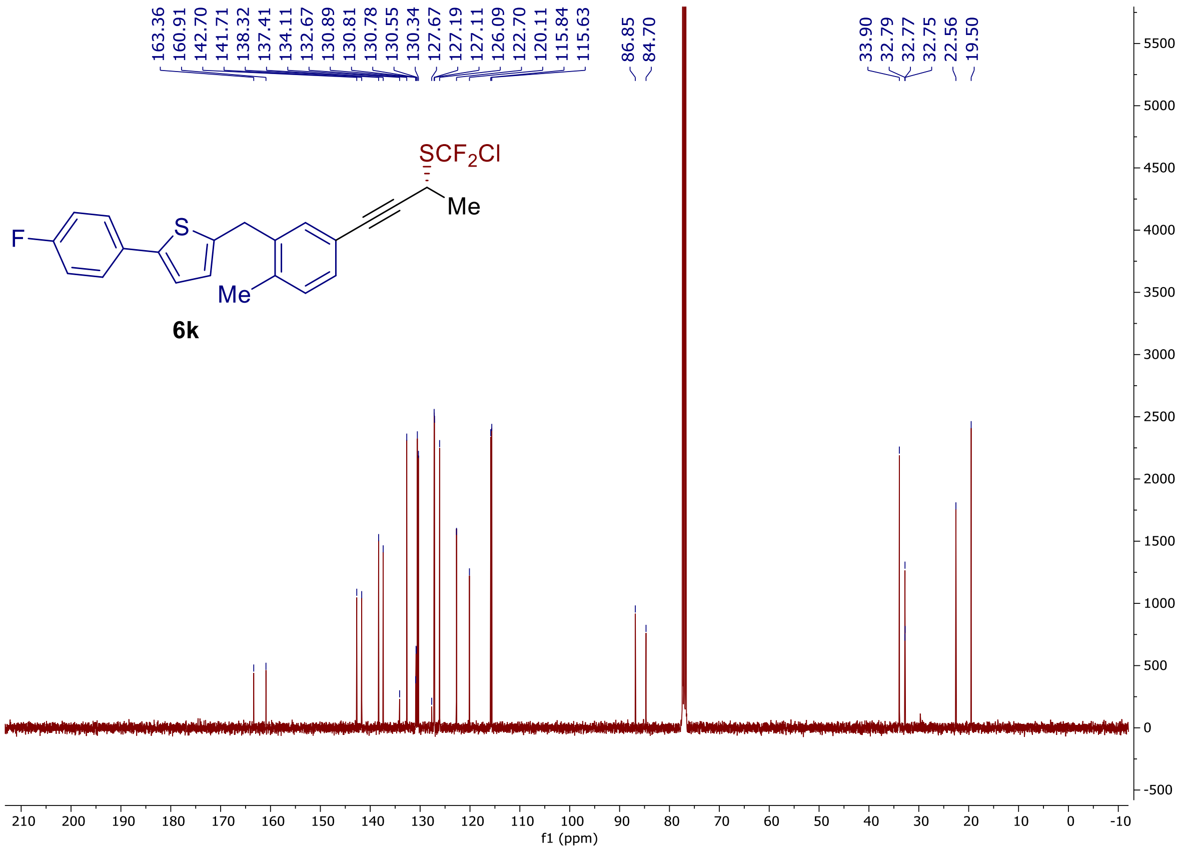

163.36
160.91
142.70
141.71
138.32
137.41
134.11
132.67
130.89
130.81
130.78
130.55
130.34
127.67
127.19
127.11
126.09
122.70
120.11
115.84
115.63
86.85
84.70
33.90
32.79
32.77
32.75
22.56
19.50
SCF2Cl
Me
F
S
Me
6k
f1 (ppm)

^{1}H NMR:

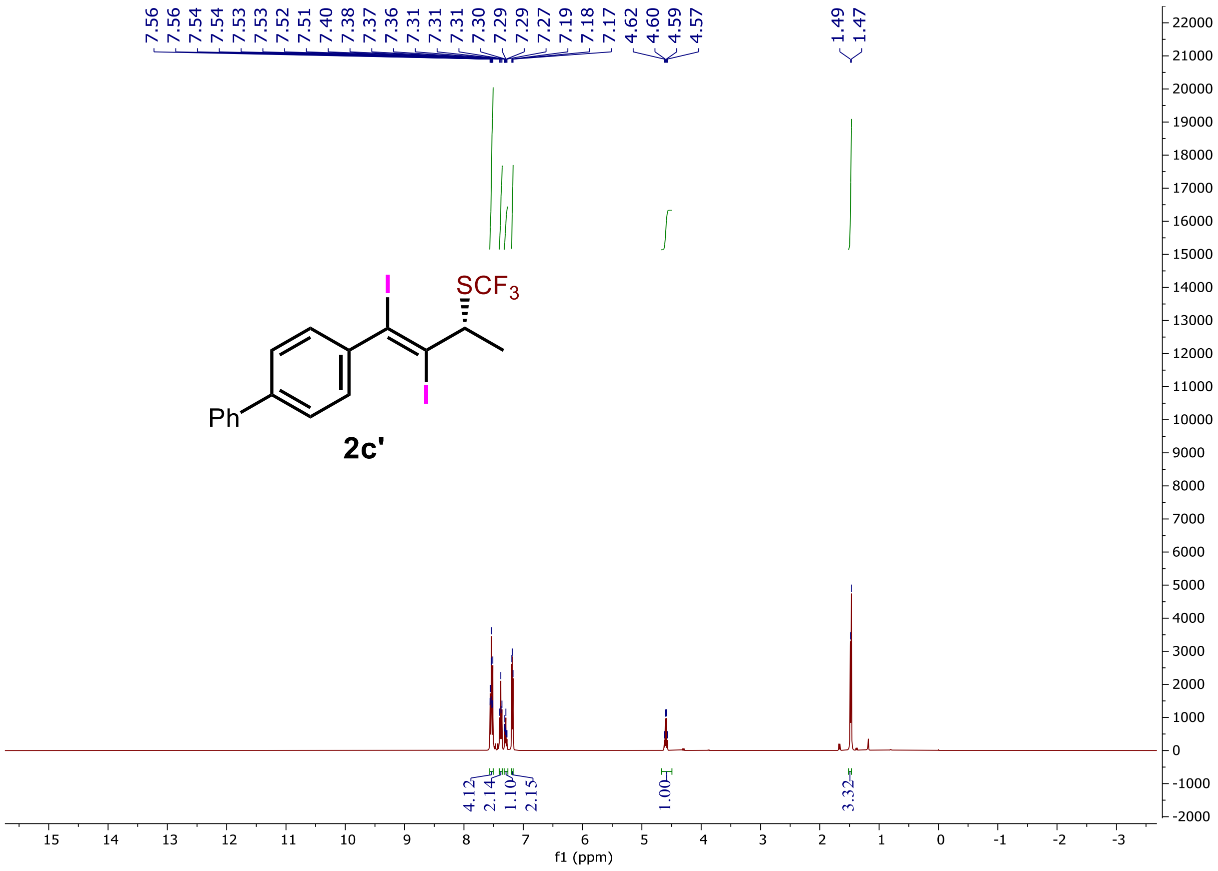

^{19}F NMR:

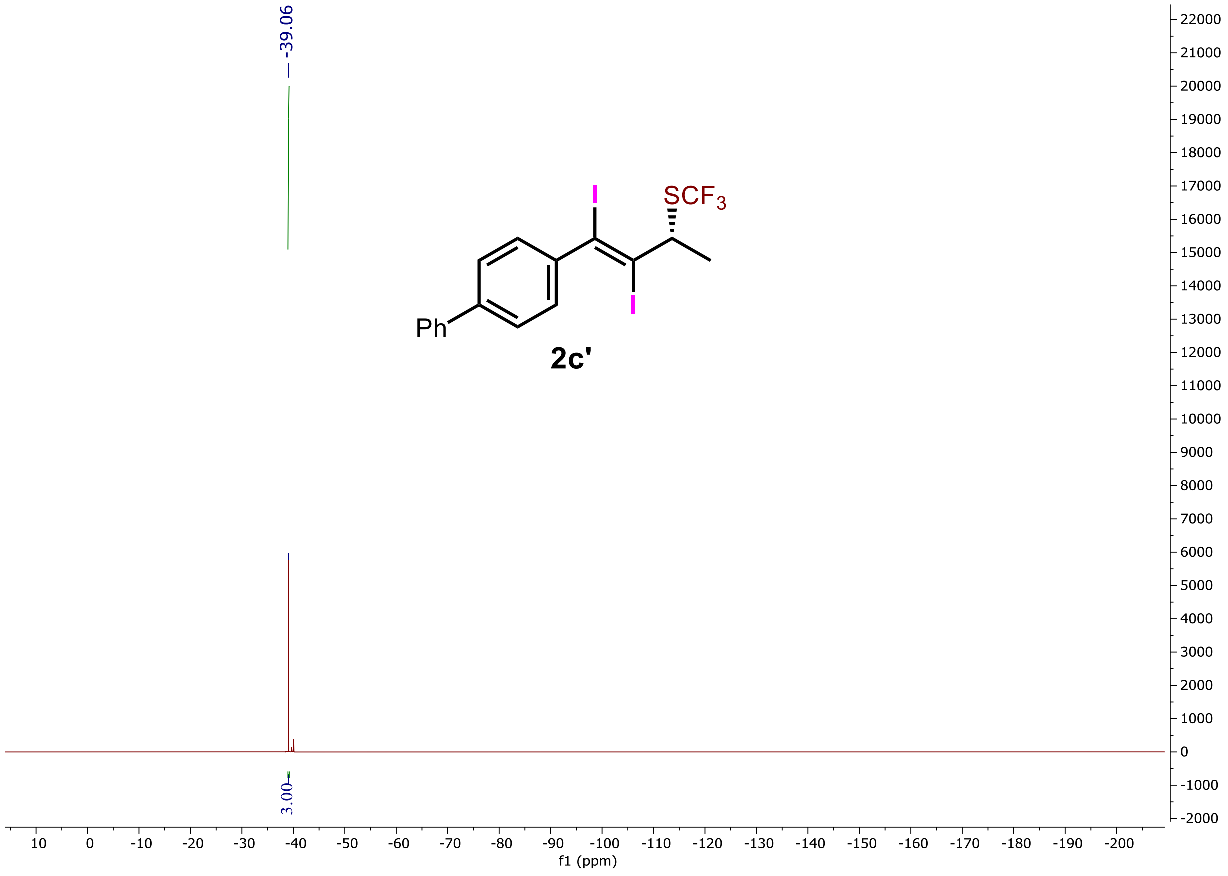

^{13}C NMR:

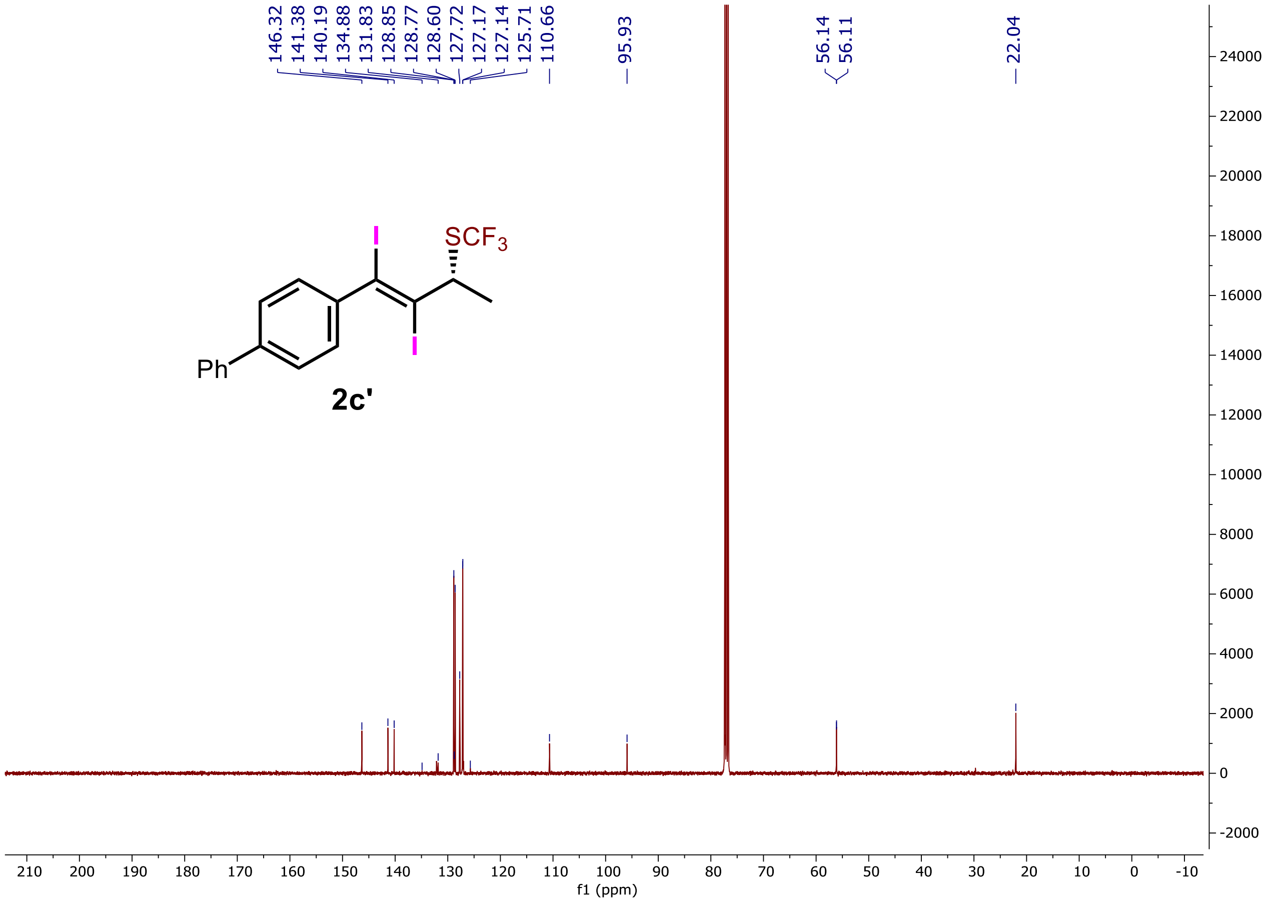
146.32
141.38
140.19
134.88
131.83
128.85
128.77
128.60
127.72
127.17
127.14
125.71
110.66
95.93
56.14
56.11
22.04
SCF3
I
I
Ph
2c'
f1 (ppm)
210 200 190 180 170 160 150 140 130 120 110 100 90 80 70 60 50 40 30 20 10 0 -10
24000 22000 20000 18000 16000 14000 12000 10000 8000 6000 4000 2000 0 -2000

^{1}H NMR:

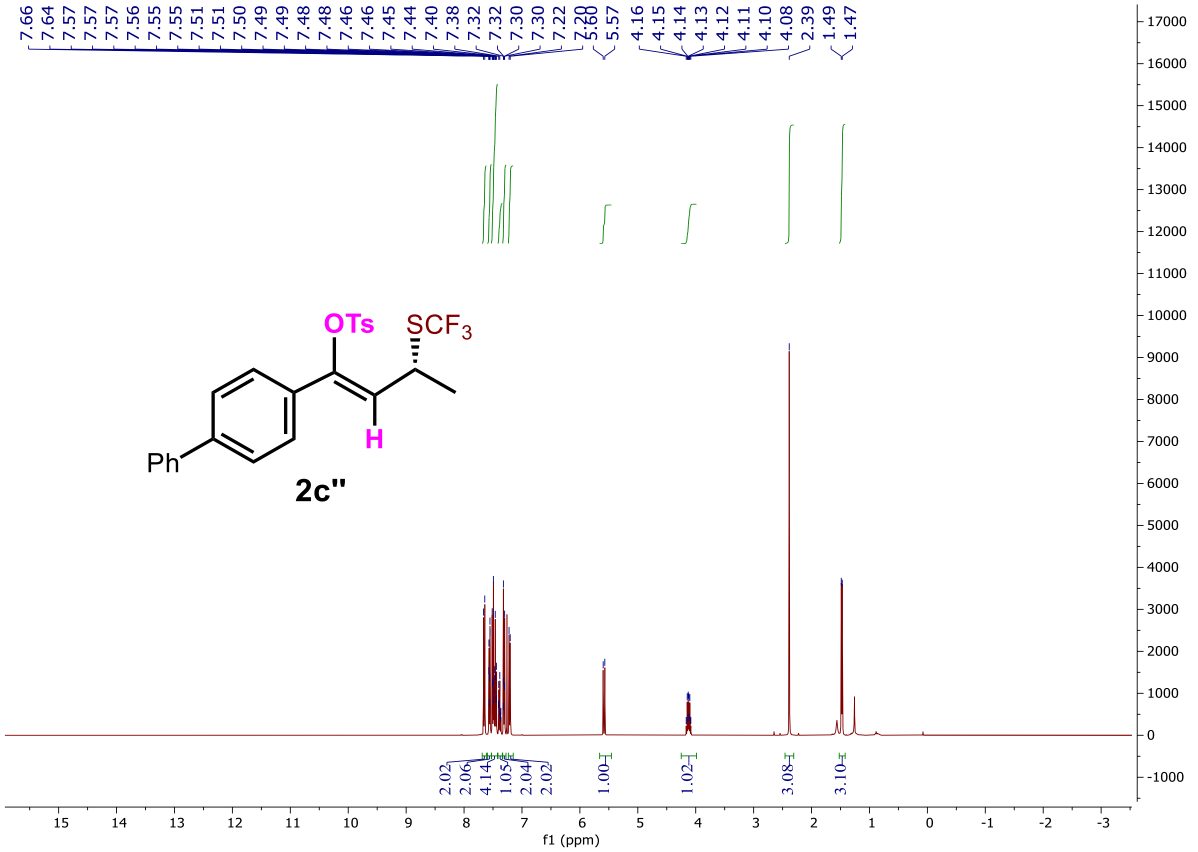

^{19}F NMR:

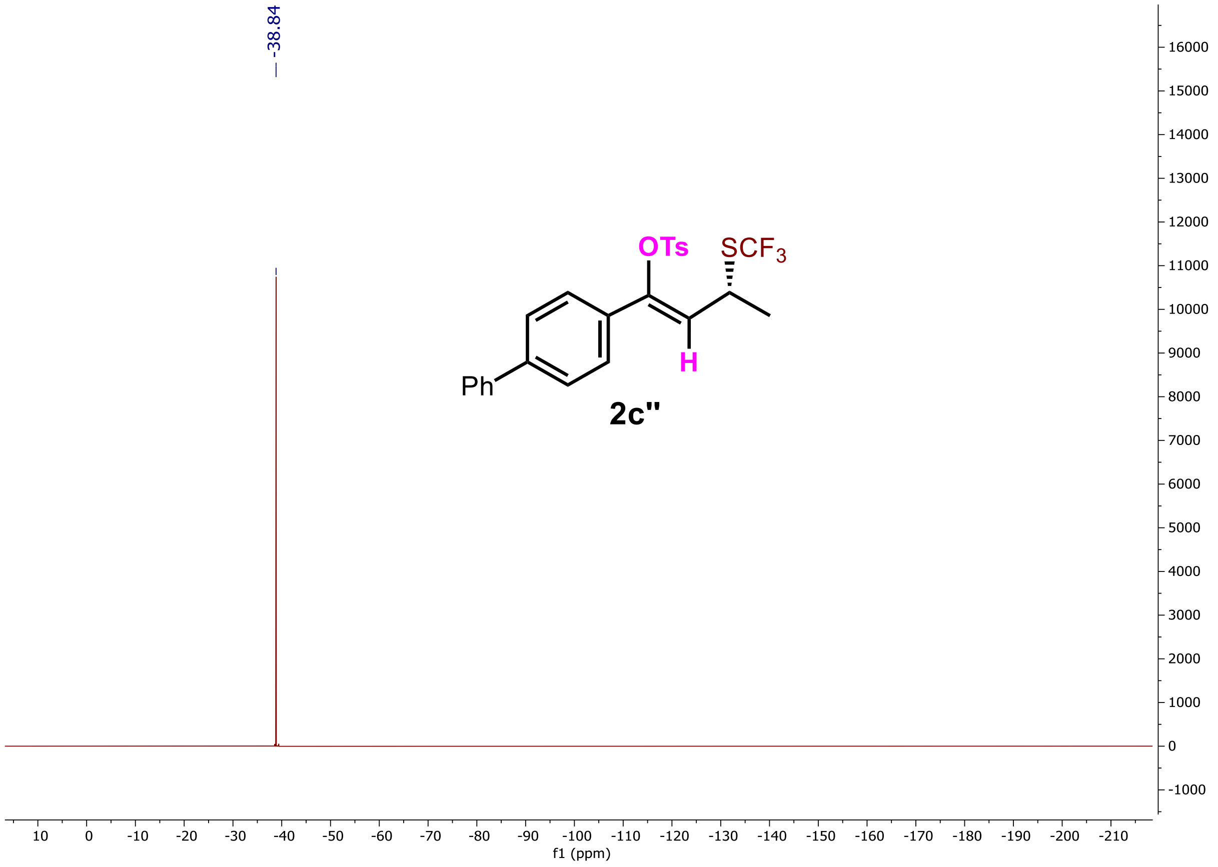

^{13}C NMR:

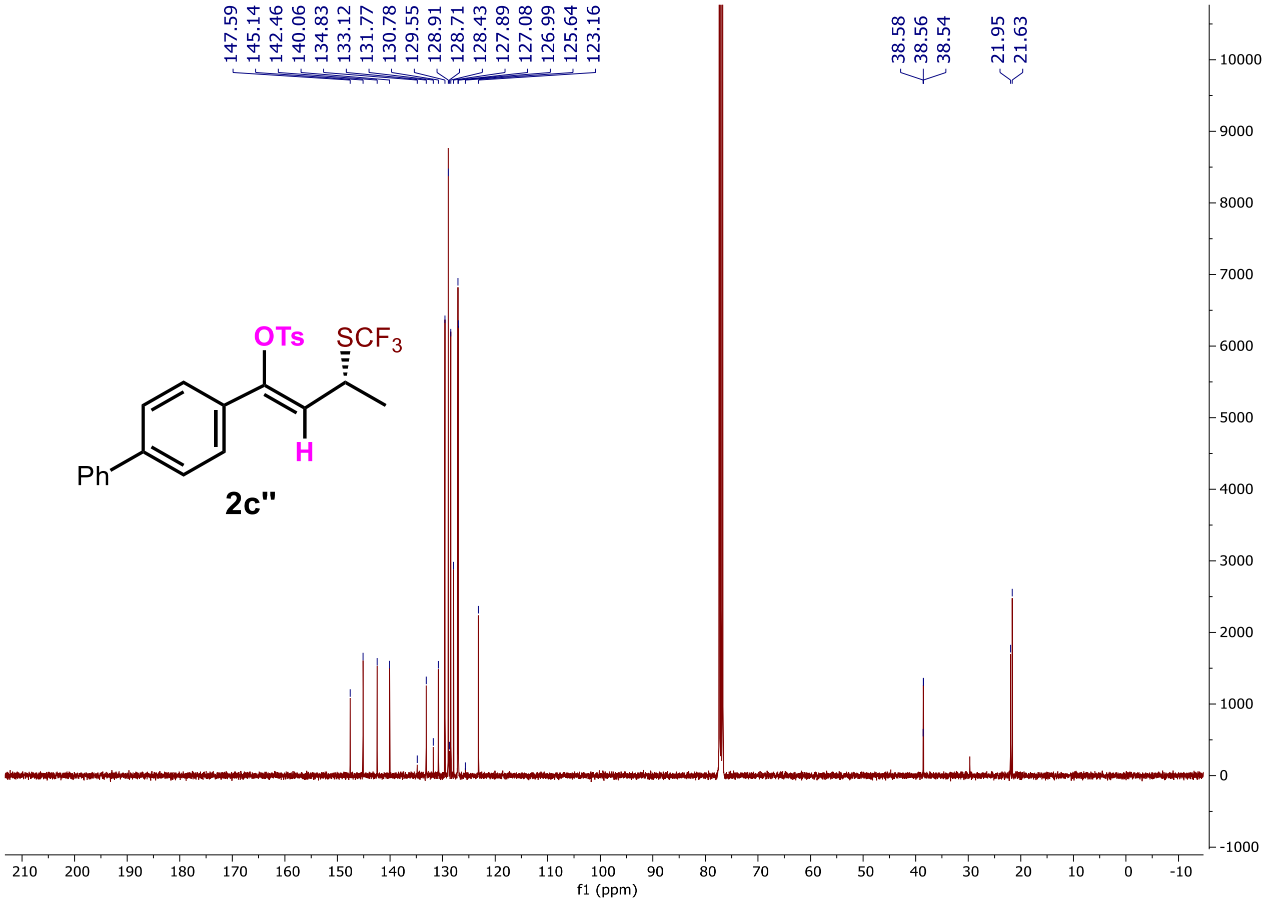

147.59
145.14
142.46
140.06
134.83
133.12
131.77
130.78
129.55
128.91
128.71
128.43
127.89
127.08
126.99
125.64
123.16
38.58
38.56
38.54
21.95
21.63
OTs
SCF3
H
Ph
2c''
f1 (ppm)

^{1}H NMR:

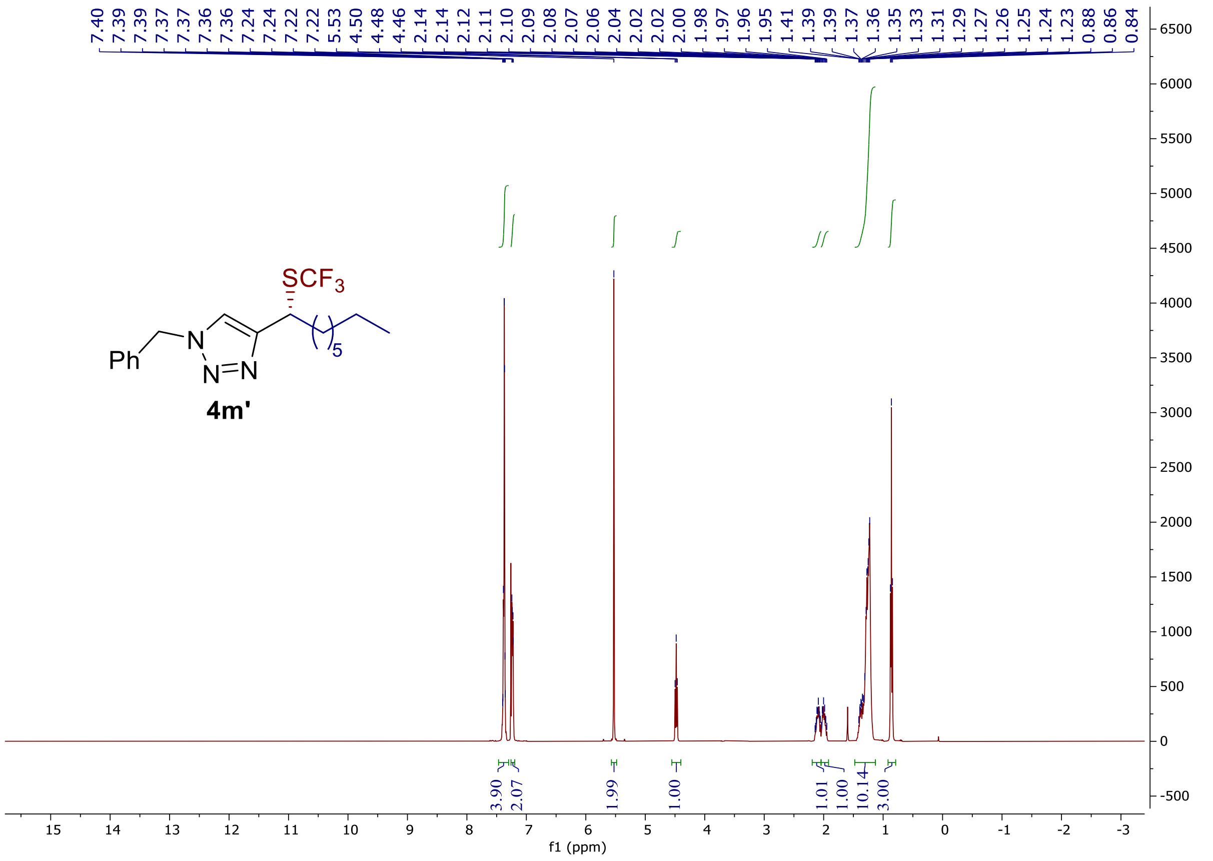

^{19}F NMR:

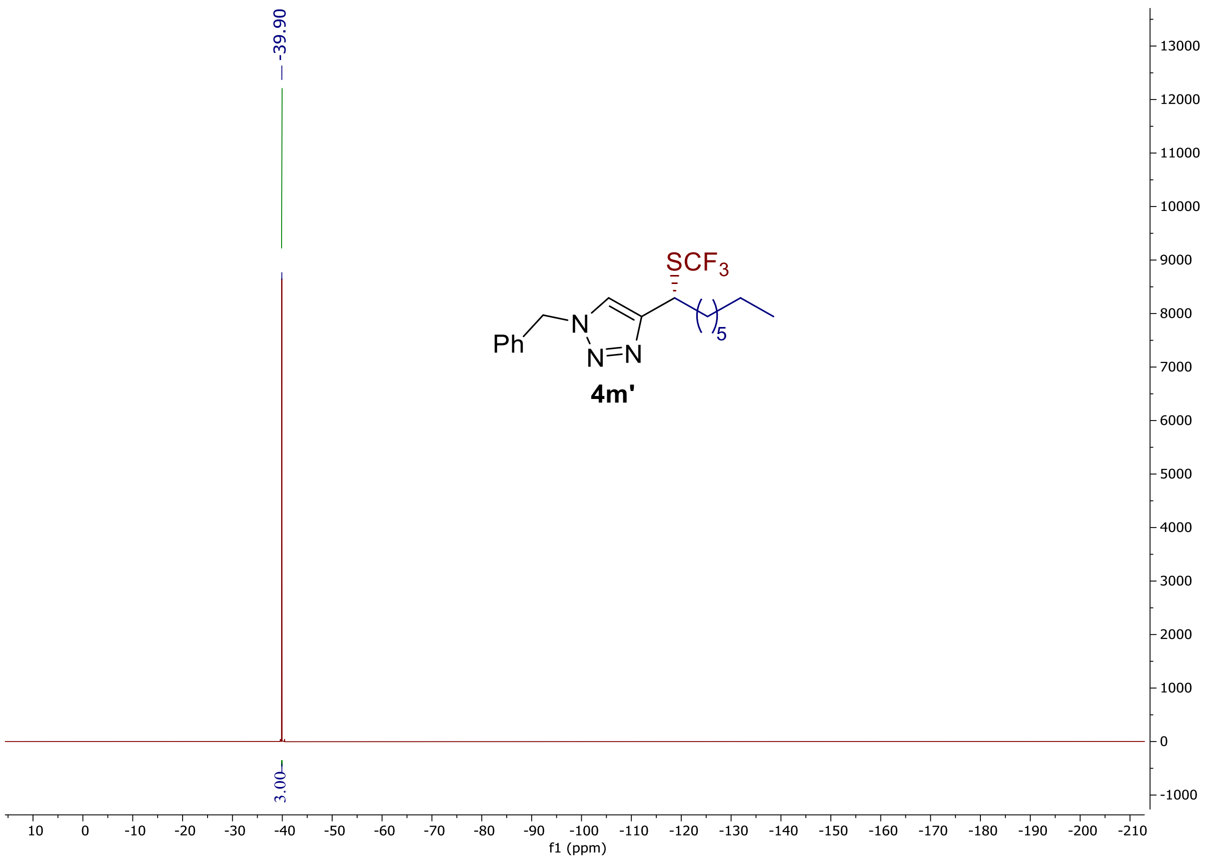

^{13}C NMR:

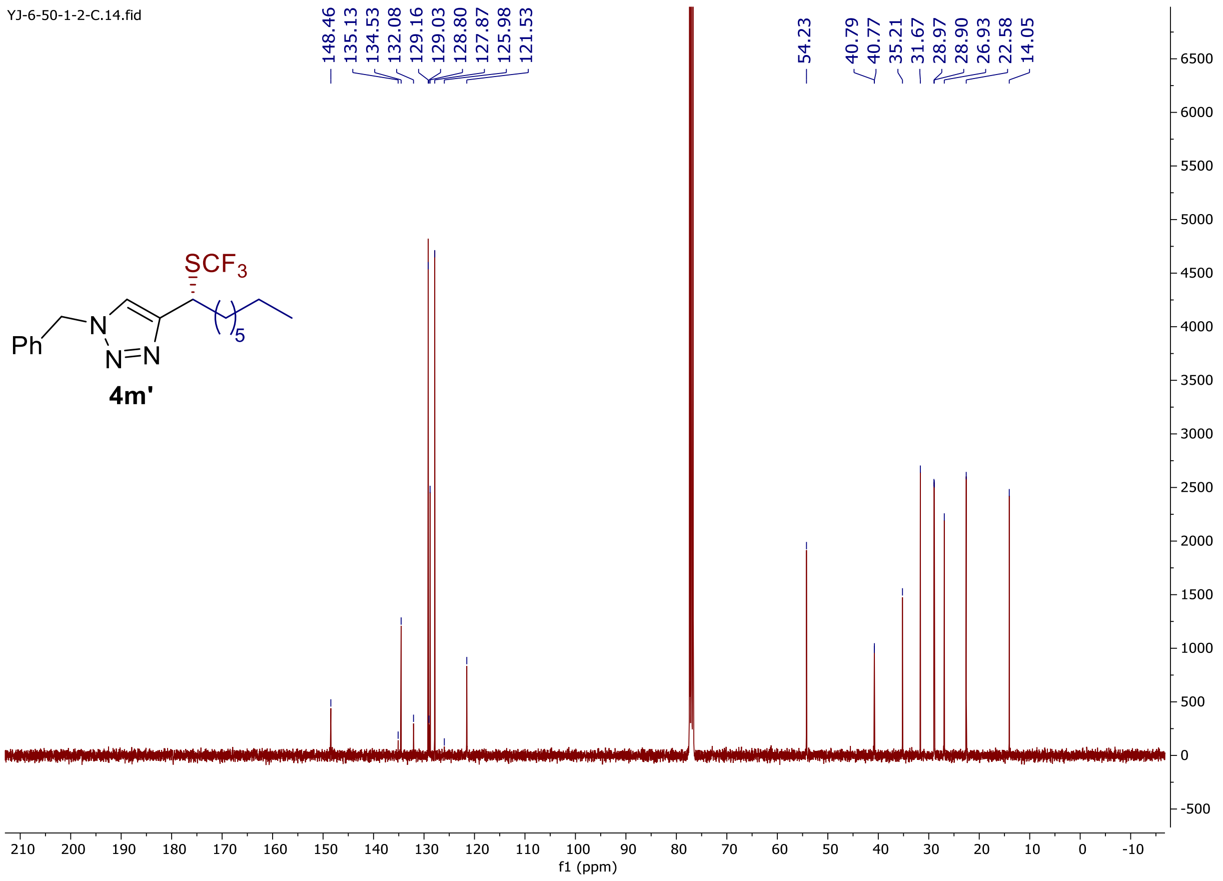

YJ-6-50-1-2-C.14.fid
148.46
135.13
134.53
132.08
129.16
129.03
128.80
127.87
125.98
121.53
54.23
40.79
40.77
35.21
31.67
28.97
28.90
26.93
22.58
14.05
f1 (ppm)

4m'

10. Copies of HPLC traces

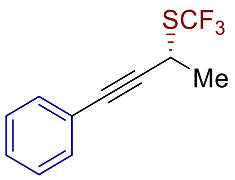

2a

Racemic trace:

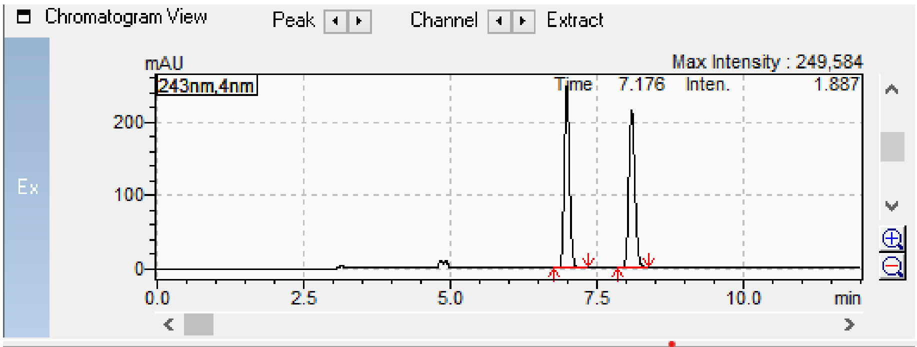

Results View - Peak Table

Peak Table | Compound | Group | Calibration Curve

Peak#	Ret. Time	Mark	Area	Height	Area%
1	6.986	M	1578662	247685	50.069
2	8.094	M	1574293	213469	49.931
Total			3152955	461154	100.000

Extracted 243nm | Ch1 254nm

Enantioenriched trace:

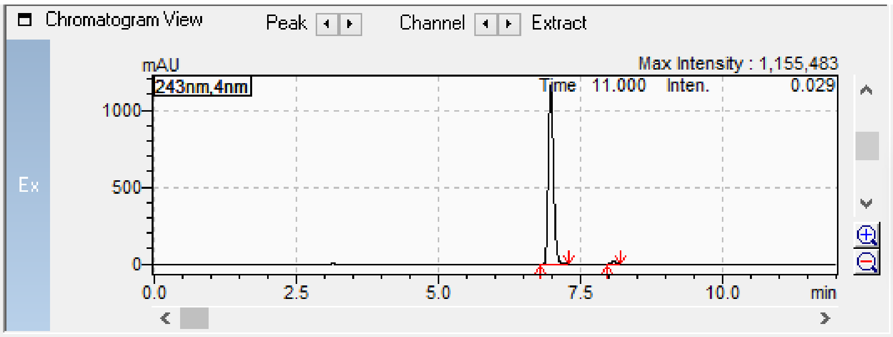

Results View - Peak Table

Peak Table | Compound | Group | Calibration Curve

Peak#	Ret. Time	Mark	Area	Height	Area%
1	6.977	M	7426565	1151561	98.845
2	8.090	M	86802	12814	1.155
Total			7513367	1164374	100.000

Extracted 243nm | Ch1 254nm

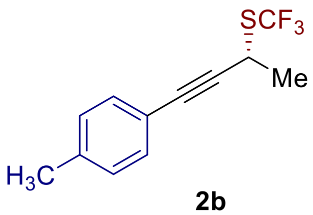

2b

Racemic trace:

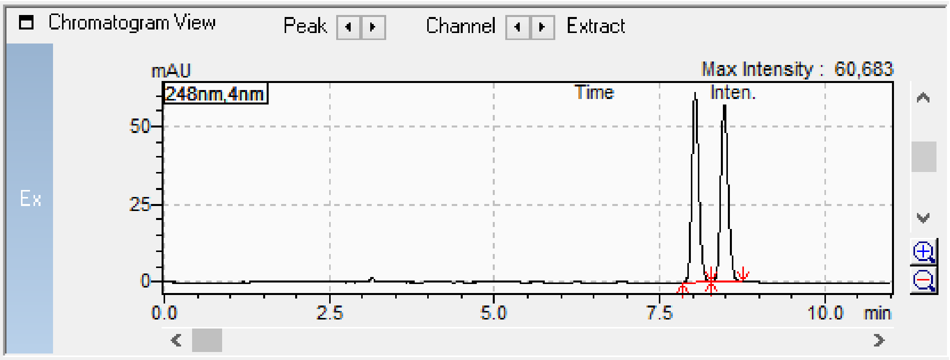

Results View - Peak Table

Peak Table | Compound | Group | Calibration Curve

Peak#	Ret. Time	Mark	Area	Height	Area%
1	8.035	M	449398	60596	50.128
2	8.476	V M	447094	56817	49.872
Total			896492	117413	100.000

Extracted 248nm

Enantioenriched trace:

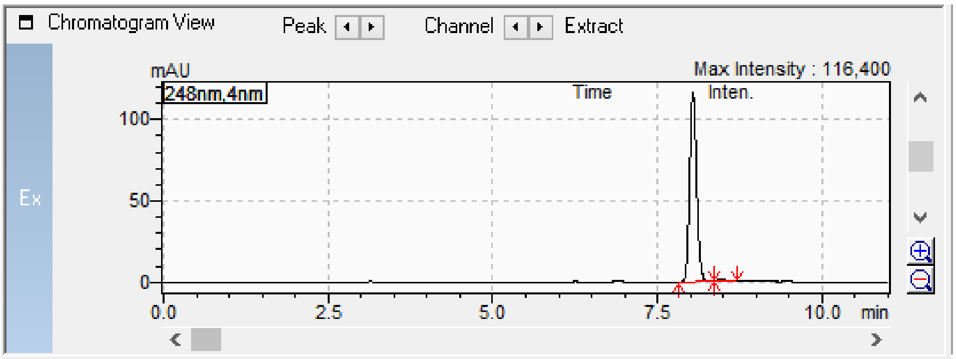

Results View - Peak Table

Peak Table | Compound | Group | Calibration Curve

Peak#	Ret. Time	Mark	Area	Height	Area%
1	8.037	M	862676	116019	98.682
2	8.471	M	11526	1552	1.318
Total			874202	117571	100.000

Extracted 248nm

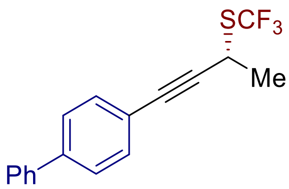

2c

Racemic trace:

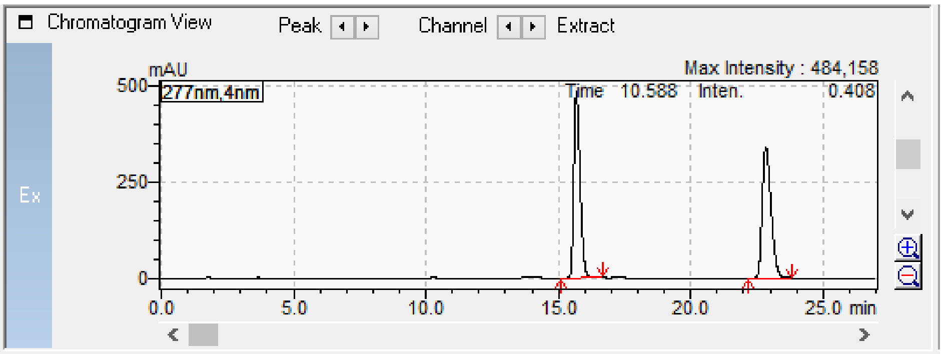

Results View - Peak Table

Peak Table | Compound | Group | Calibration Curve

Peak#	Ret. Time	Mark	Area	Height	Area%
1	15.676	M	7952724	483418	50.056
2	22.837	M	7934791	340287	49.944
Total			15887515	823705	100.000

Extracted 277nm | Ch1 254nm

Enantioenriched trace:

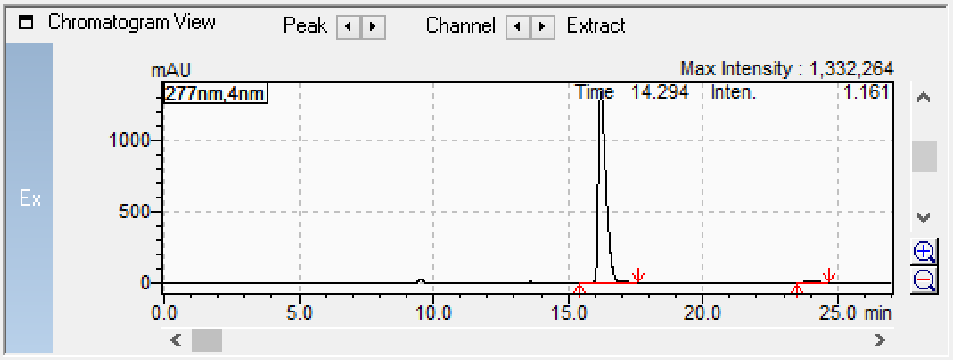

Results View - Peak Table

Peak Table | Compound | Group | Calibration Curve

Peak#	Ret. Time	Mark	Area	Height	Area%
1	16.210	M	25832305	1331164	98.716
2	24.009		335984	14648	1.284
Total			26168288	1345812	100.000

Extracted 277nm | Ch1 254nm

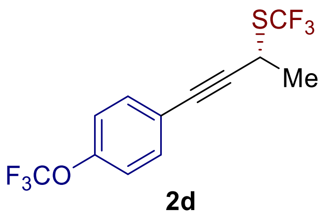

2d

Racemic trace:

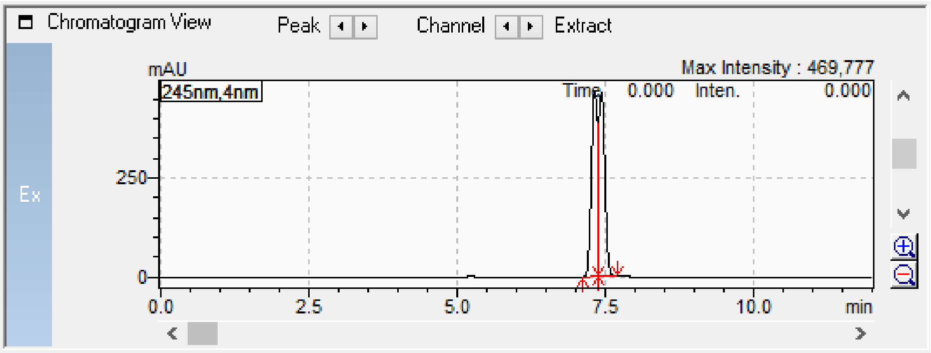

Results View - Peak Table

Peak Table | Compound | Group | Calibration Curve

Peak#	Ret. Time	Mark	Area	Height	Area%
1	7.321	M	3426729	468501	51.526
2	7.436	V M	3223749	465051	48.474
Total			6650478	933552	100.000

Extracted 245nm

Enantioenriched trace:

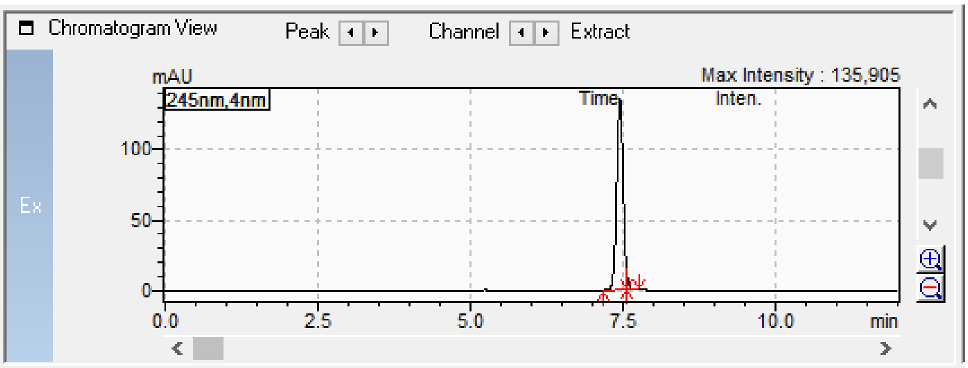

Results View - Peak Table

Peak Table | Compound | Group | Calibration Curve

Peak#	Ret. Time	Mark	Area	Height	Area%
1	7.450	M	1010895	135256	97.806
2	7.570	V M	22682	11898	2.194
Total			1033576	147153	100.000

Extracted 245nm

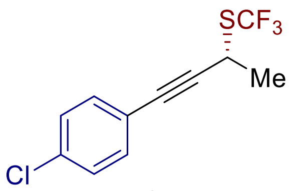

2e

Racemic trace:

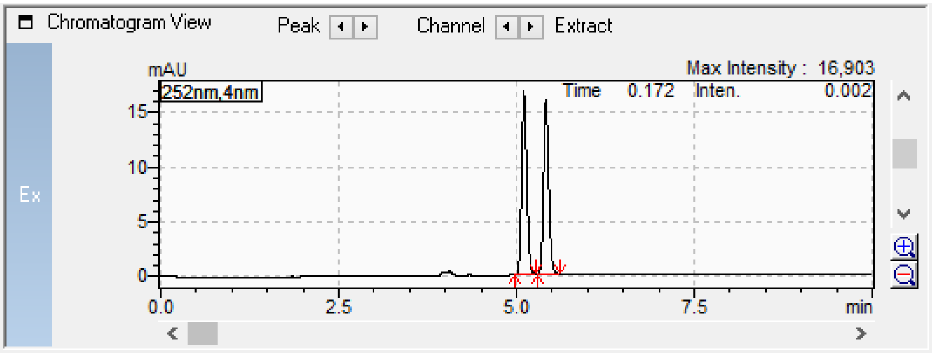

Results View - Peak Table

Peak Table | Compound | Group | Calibration Curve

Peak#	Ret. Time	Mark	Area	Height	Area%
1	5.105	M	82043	16617	49.947
2	5.411	M	82218	15771	50.053
Total			164262	32388	100.000

Extracted 252nm / Ch1 254nm

Enantioenriched trace:

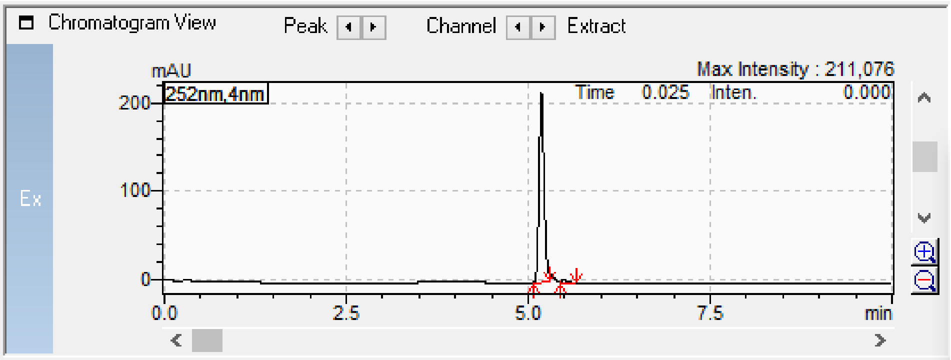

Results View - Peak Table

Peak Table | Compound | Group | Calibration Curve

Peak#	Ret. Time	Mark	Area	Height	Area%
1	5.181	M	1071474	214262	98.790
2	5.493	M	13128	2481	1.210
Total			1084601	216743	100.000

Extracted 252nm / Ch1 254nm

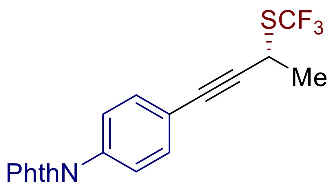

2f

Racemic trace:

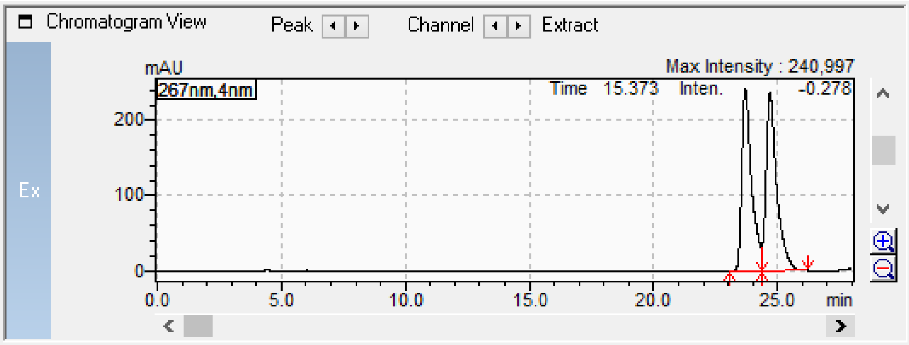

Results View - Peak Table

Peak Table | Compound | Group | Calibration Curve

Peak#	Ret. Time	Mark	Area	Height	Area%
1	23.694	M	6615163	241232	48.195
2	24.690	V M	7110561	236399	51.805
Total			13725724	477631	100.000

Extracted 267nm

Enantioenriched trace:

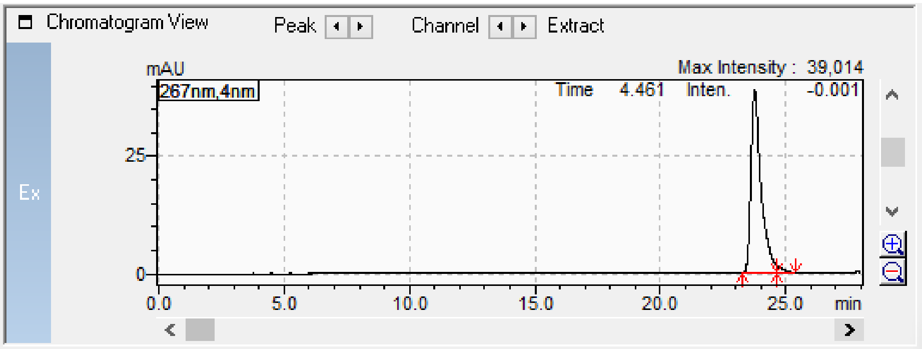

Results View - Peak Table

Peak Table | Compound | Group | Calibration Curve

Peak#	Ret. Time	Mark	Area	Height	Area%
1	23.759	M	1080898	38780	97.942
2	24.642	V M	22711	1513	2.058
Total			1103609	40293	100.000

Extracted 267nm

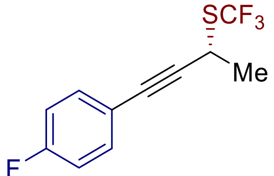

2g

Racemic trace:

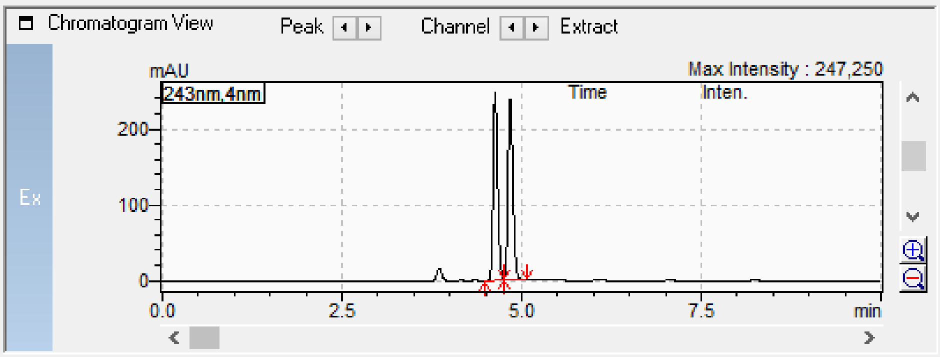

Results View - Peak Table

Peak Table | Compound | Group | Calibration Curve

Peak#	Ret. Time	Mark	Area	Height	Area%
1	4.632	M	1117818	245789	49.799
2	4.842	V M	1126861	236052	50.201
Total			2244679	481841	100.000

Extracted 243nm | Ch1 254nm

Enantioenriched trace:

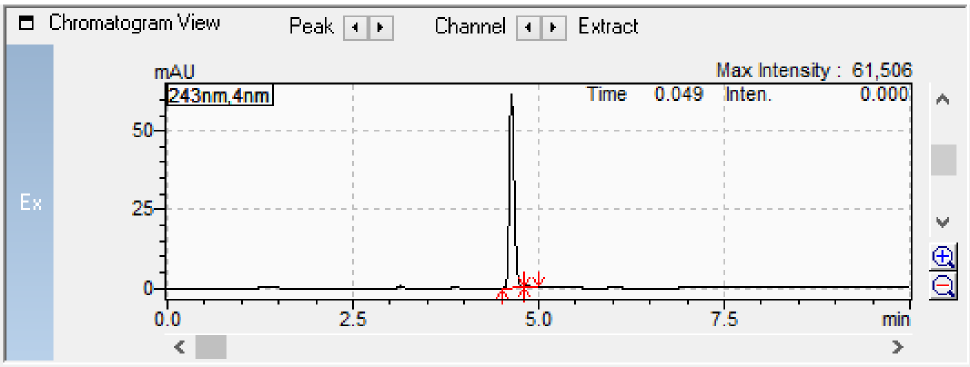

Results View - Peak Table

Peak Table | Compound | Group | Calibration Curve

Peak#	Ret. Time	Mark	Area	Height	Area%
1	4.636	M	280710	61135	98.627
2	4.843	V M	3908	770	1.373
Total			284617	61905	100.000

Extracted 243nm | Ch1 254nm

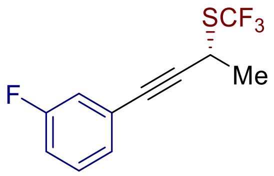

2h

Racemic trace:

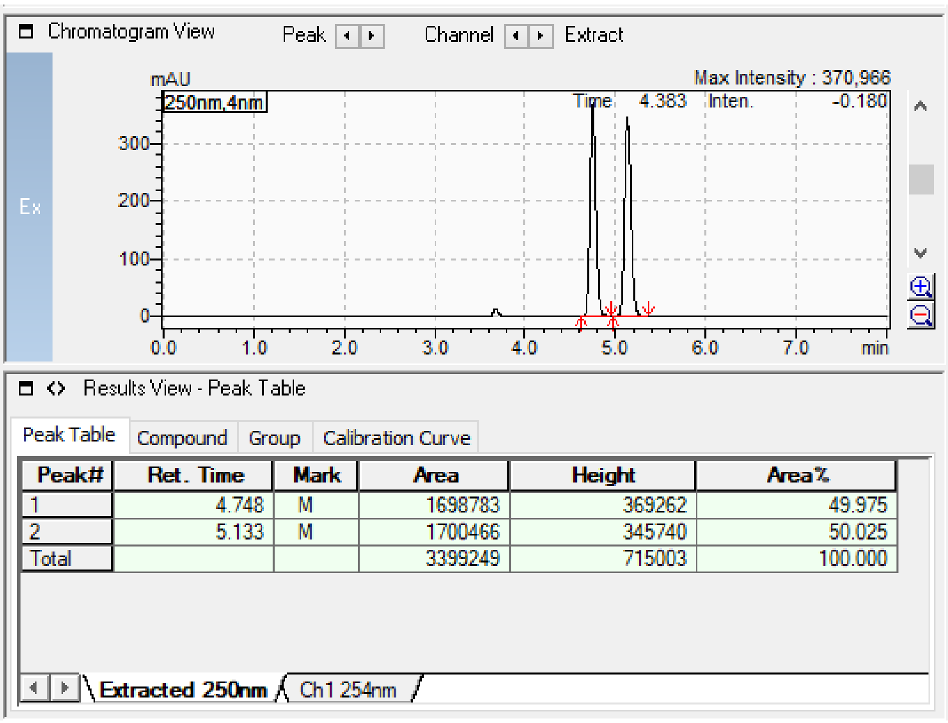

Peak#	Ret. Time	Mark	Area	Height	Area%
1	4.748	M	1698783	369262	49.975
2	5.133	M	1700466	345740	50.025
Total			3399249	715003	100.000

Enantioenriched trace:

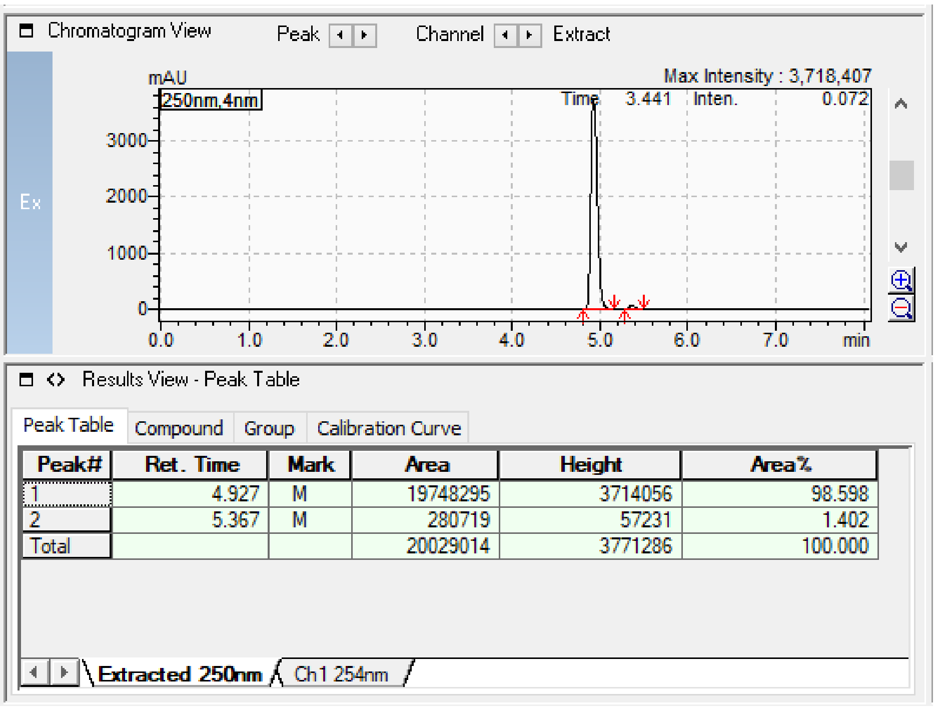

Peak#	Ret. Time	Mark	Area	Height	Area%
1	4.927	M	19748295	3714056	98.598
2	5.367	M	280719	57231	1.402
Total			20029014	3771286	100.000

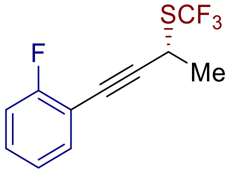

2i

Racemic trace:

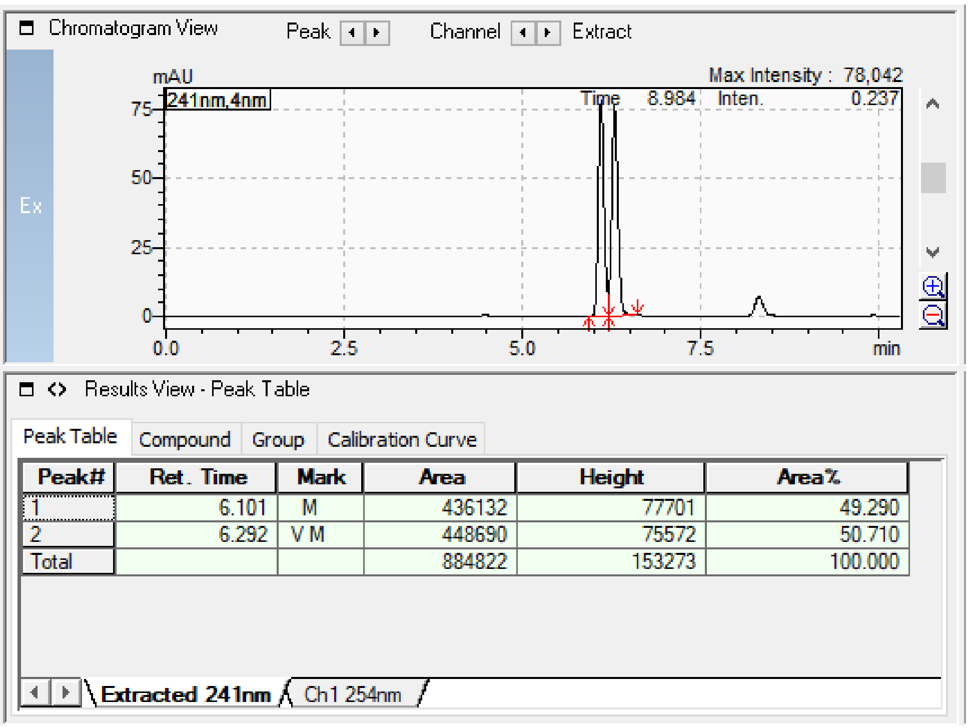

Peak#	Ret. Time	Mark	Area	Height	Area%
1	6.101	M	436132	77701	49.290
2	6.292	V M	448690	75572	50.710
Total			884822	153273	100.000

Enantioenriched trace:

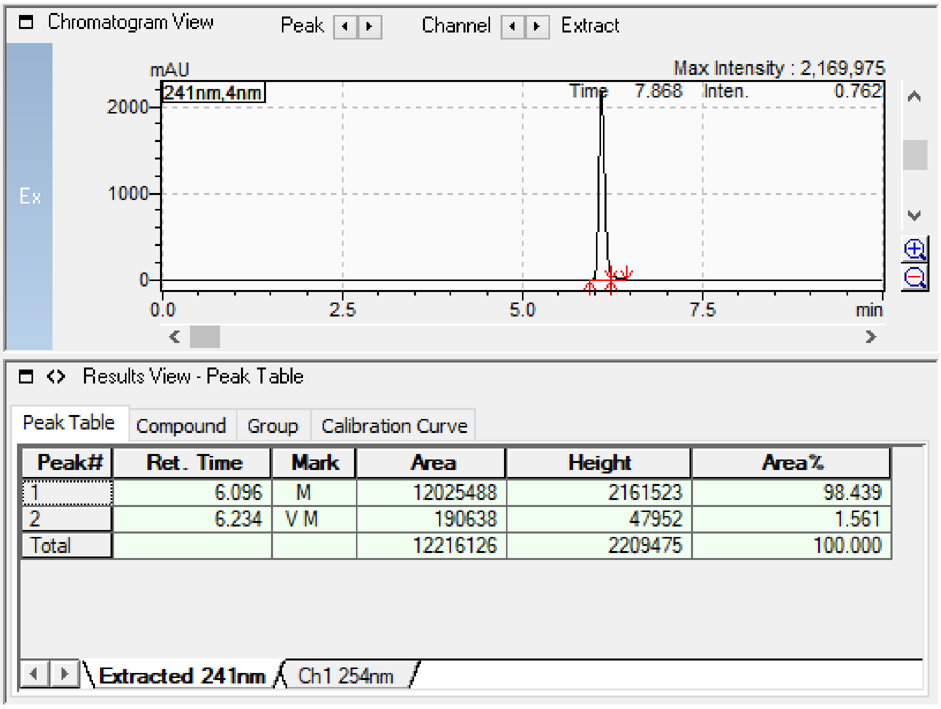

Peak#	Ret. Time	Mark	Area	Height	Area%
1	6.096	M	12025488	2161523	98.439
2	6.234	V M	190638	47952	1.561
Total			12216126	2209475	100.000

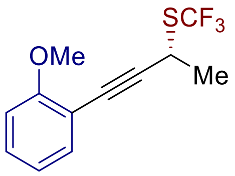

2j

Racemic trace:

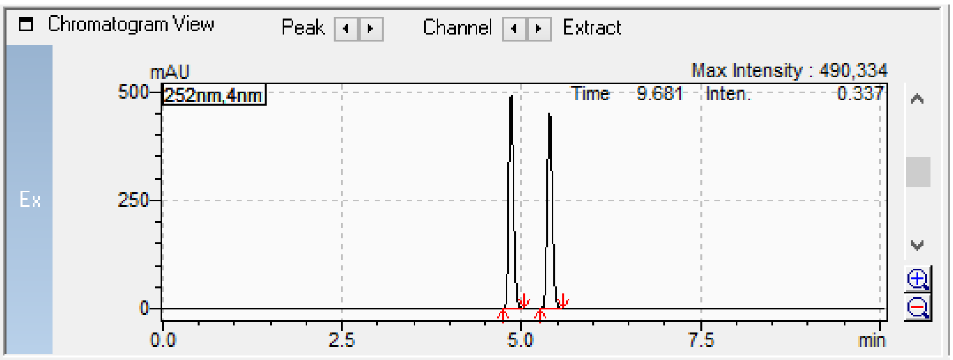

Results View - Peak Table

Peak Table | Compound | Group | Calibration Curve

Peak#	Ret. Time	Mark	Area	Height	Area%
1	4.855	M	2268508	488666	50.041
2	5.393	M	2264788	445548	49.959
Total			4533296	934214	100.000

Extracted 252nm | Ch1 254nm

Enantioenriched trace:

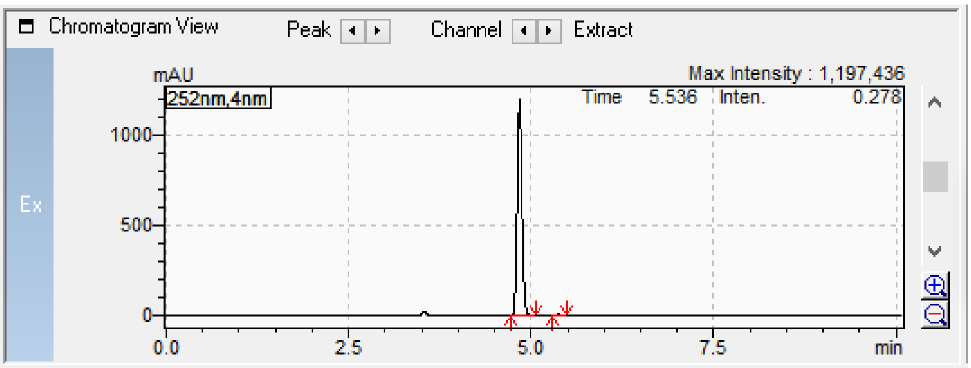

Results View - Peak Table

Peak Table | Compound | Group | Calibration Curve

Peak#	Ret. Time	Mark	Area	Height	Area%
1	4.841	M	5511295	1189342	99.573
2	5.378	M	23644	4864	0.427
Total			5534939	1194206	100.000

Extracted 252nm | Ch1 254nm

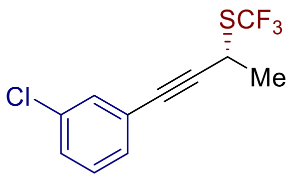

2k

Racemic trace:

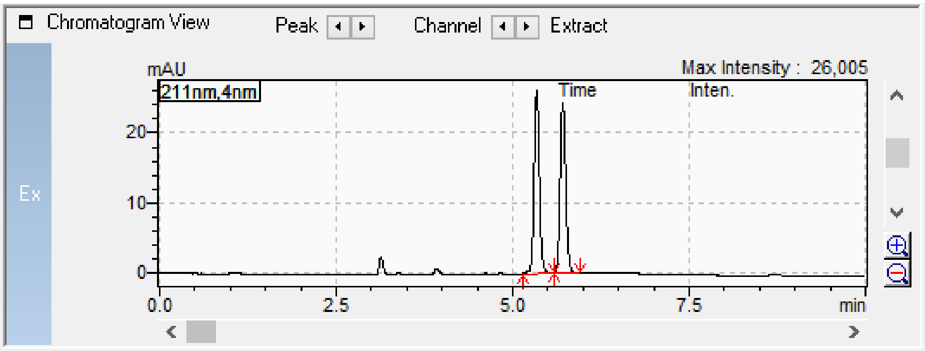

Results View - Peak Table

Peak Table | Compound | Group | Calibration Curve

Peak#	Ret. Time	Mark	Area	Height	Area%
1	5.338	M	138803	25947	50.512
2	5.711	V M	135989	24454	49.488
Total			274792	50401	100.000

Extracted 211nm | Ch1 254nm

Enantioenriched trace:

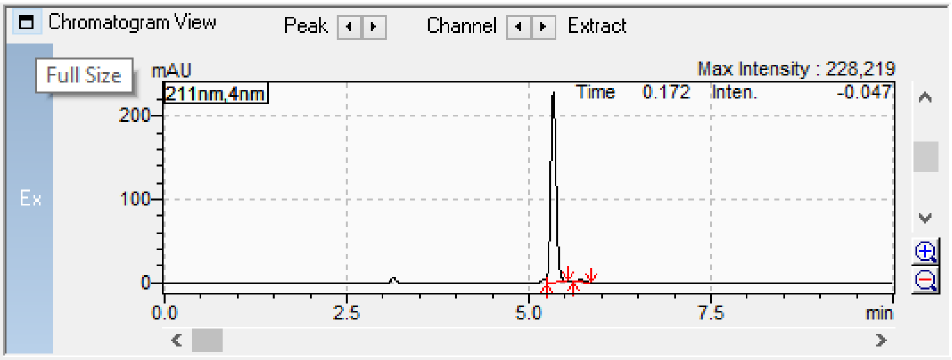

Results View - Peak Table

Peak Table | Compound | Group | Calibration Curve

Peak#	Ret. Time	Mark	Area	Height	Area%
1	5.336	M	1178264	226977	98.450
2	5.709	M	18553	3492	1.550
Total			1196817	230469	100.000

Extracted 211nm | Ch1 254nm

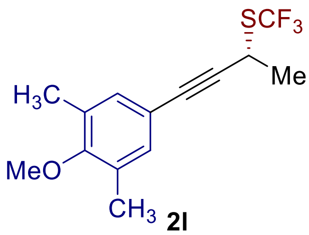

2l

Racemic trace:

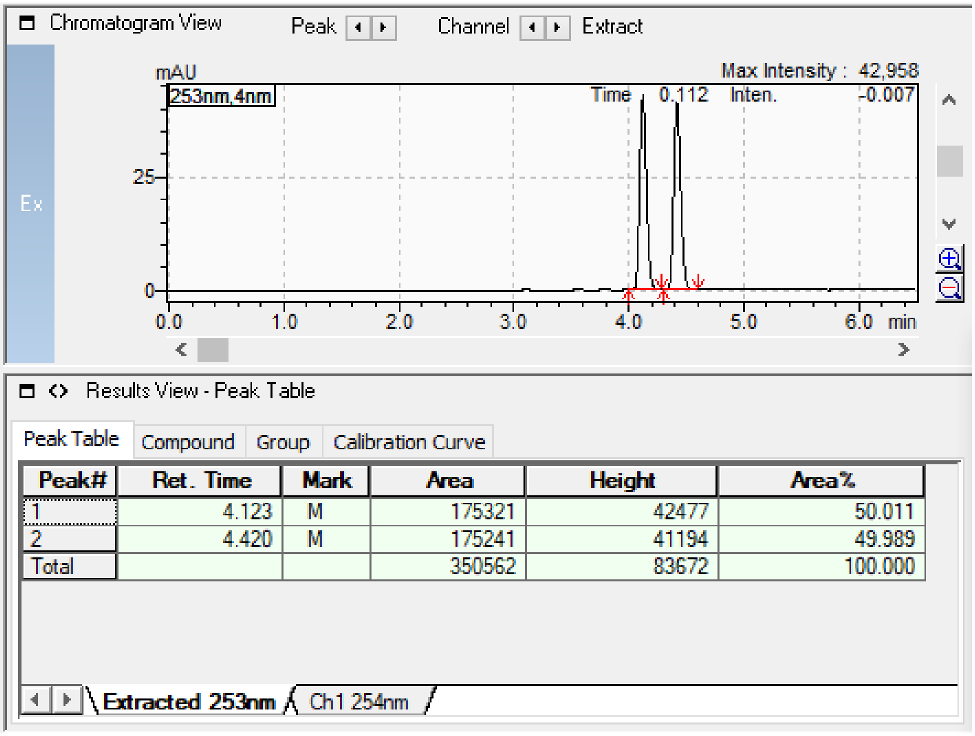

Peak#	Ret. Time	Mark	Area	Height	Area%
1	4.123	M	175321	42477	50.011
2	4.420	M	175241	41194	49.989
Total			350562	83672	100.000

Enantioenriched trace:

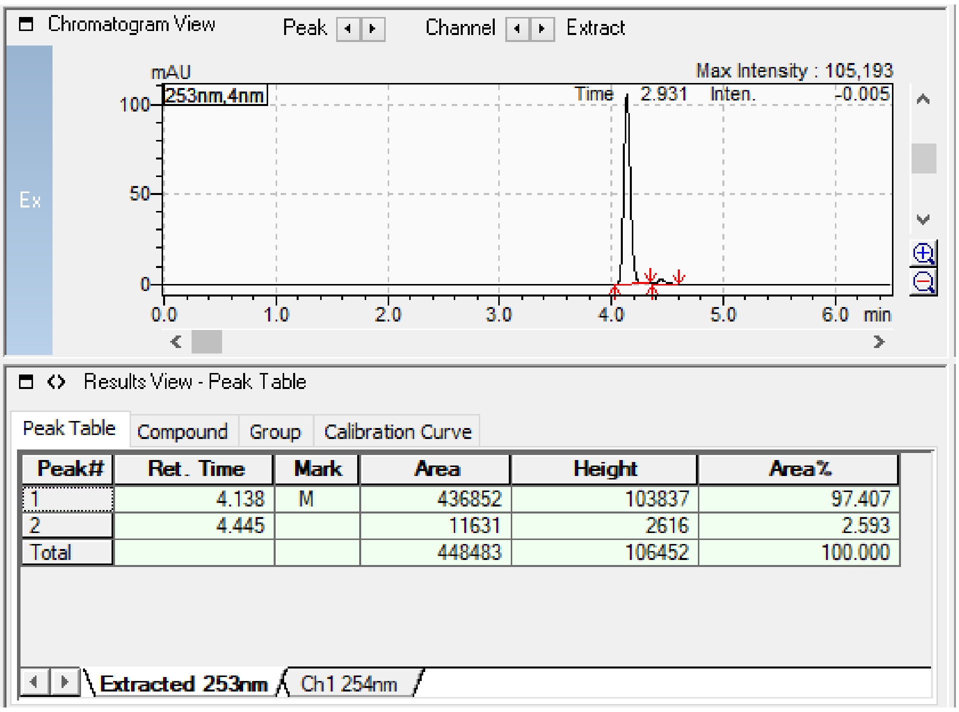

Peak#	Ret. Time	Mark	Area	Height	Area%
1	4.138	M	436852	103837	97.407
2	4.445		11631	2616	2.593
Total			448483	106452	100.000

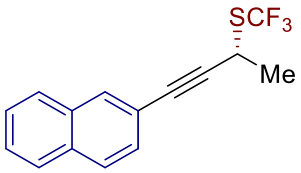

2m

Racemic trace:

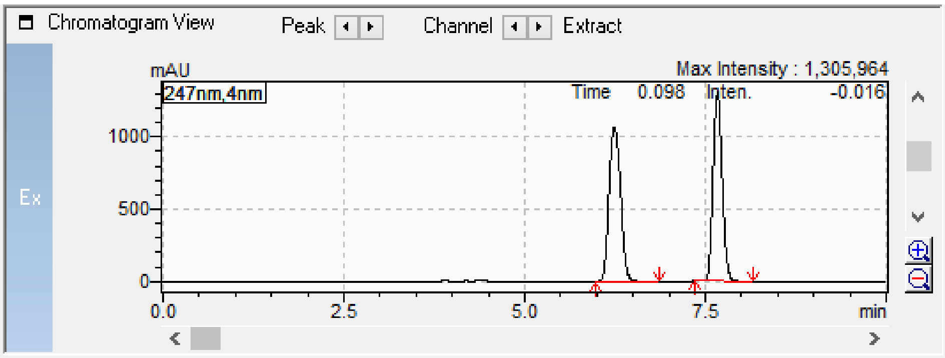

Results View - Peak Table

Peak Table | Compound | Group | Calibration Curve

Peak#	Ret. Time	Mark	Area	Height	Area%
1	6.242	M	11847264	1060676	50.693
2	7.666	M	11523334	1300863	49.307
Total			23370597	2361539	100.000

Extracted 247nm | Ch1 254nm

Enantioenriched trace:

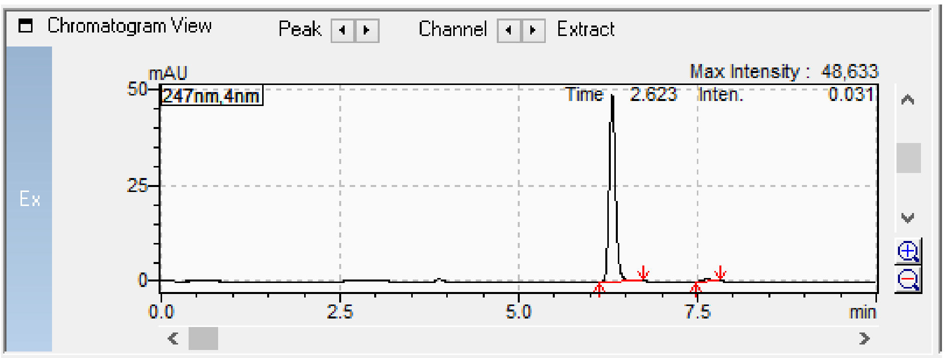

Results View - Peak Table

Peak Table | Compound | Group | Calibration Curve

Peak#	Ret. Time	Mark	Area	Height	Area%
1	6.307	M	301758	48537	98.458
2	7.629		4726	648	1.542
Total			306484	49185	100.000

Extracted 247nm | Ch1 254nm

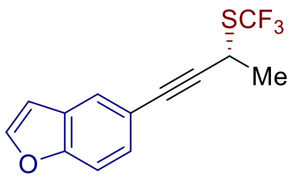

2n

Racemic trace:

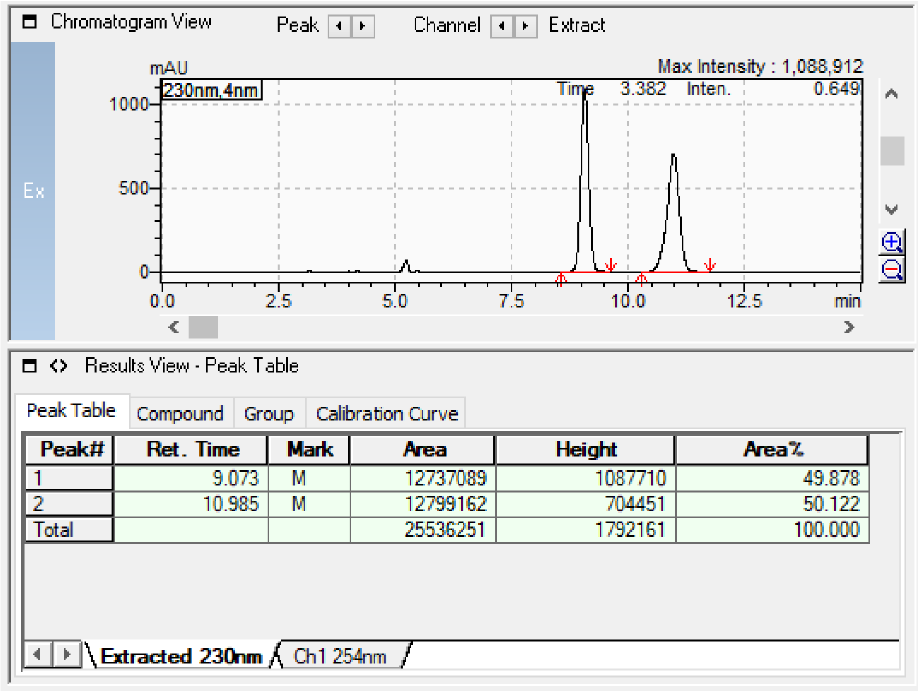

Peak#	Ret. Time	Mark	Area	Height	Area%
1	9.073	M	12737089	1087710	49.878
2	10.985	M	12799162	704451	50.122
Total			25536251	1792161	100.000

Enantioenriched trace:

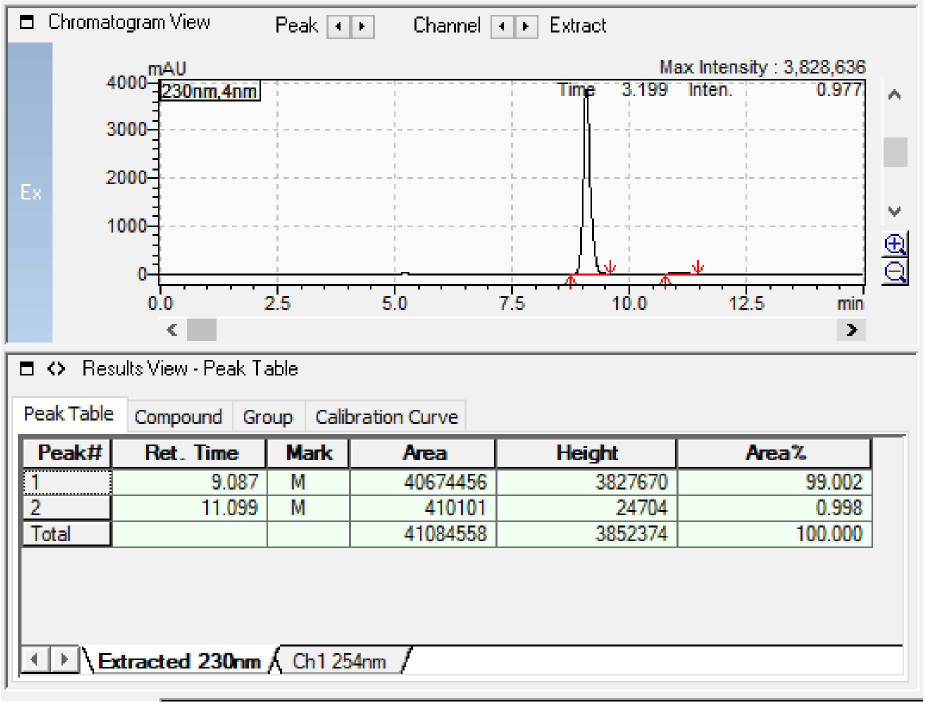

Peak#	Ret. Time	Mark	Area	Height	Area%
1	9.087	M	40674456	3827670	99.002
2	11.099	M	410101	24704	0.998
Total			41084558	3852374	100.000

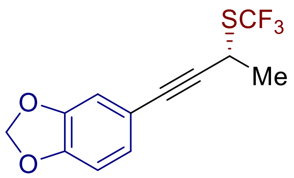

2o

Racemic trace:

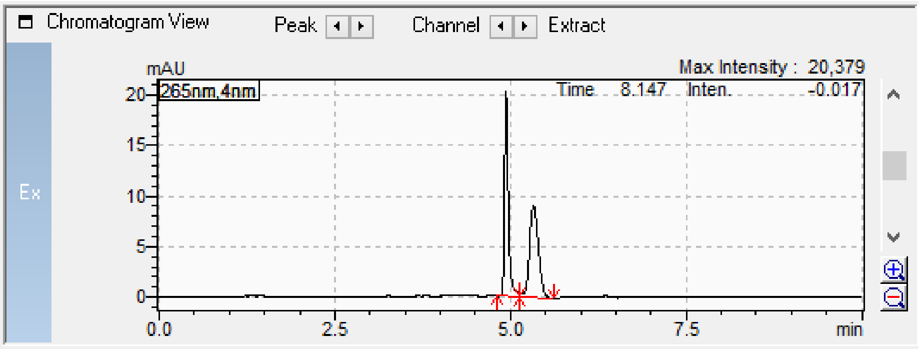

Results View - Peak Table

Peak Table | Compound | Group | Calibration Curve

Peak#	Ret. Time	Mark	Area	Height	Area%
1	4.941	M	79786	20238	49.874
2	5.330	V M	80189	9086	50.126
Total			159975	29324	100.000

Extracted 265nm | Ch1 254nm

Enantioenriched trace:

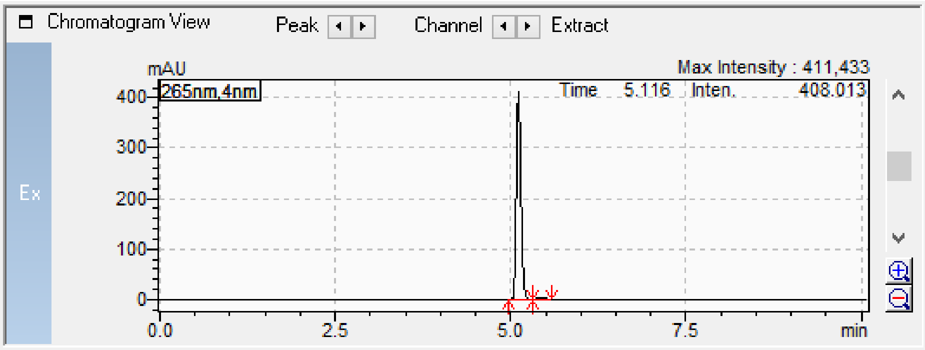

Results View - Peak Table

Peak Table | Compound | Group | Calibration Curve

Peak#	Ret. Time	Mark	Area	Height	Area%
1	5.112	M	1908300	409193	98.521
2	5.425	M	28657	4503	1.479
Total			1936957	413696	100.000

Extracted 265nm | Ch1 254nm

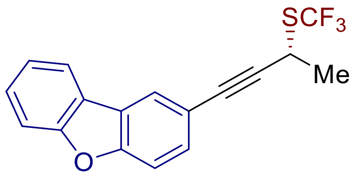

2p

Racemic trace:

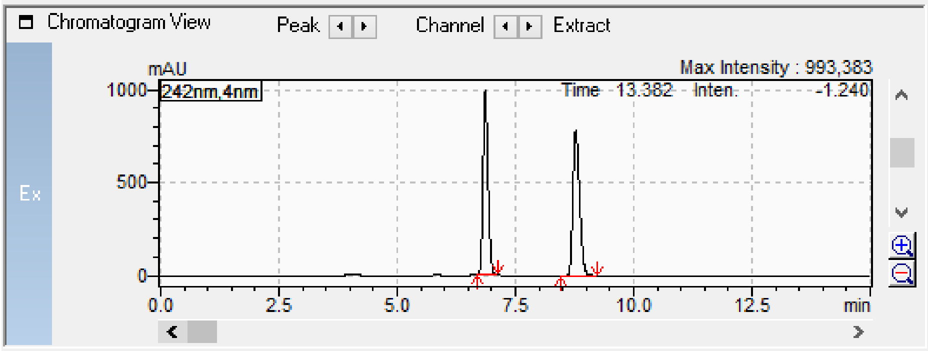

Results View - Peak Table

Peak Table | Compound | Group | Calibration Curve

Peak#	Ret. Time	Mark	Area	Height	Area%
1	6.862	M	7572919	990242	49.781
2	8.778	M	7639629	782514	50.219
Total			15212548	1772756	100.000

Extracted 242nm | Ch1 254nm

Enantioenriched trace:

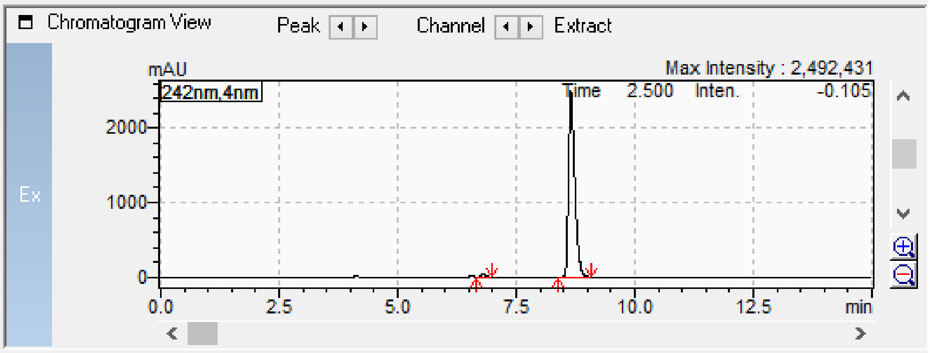

Results View - Peak Table

Peak Table | Compound | Group | Calibration Curve

Peak#	Ret. Time	Mark	Area	Height	Area%
1	6.808	M	258466	37366	1.139
2	8.655	M	22434759	2489693	98.861
Total			22693225	2527060	100.000

Extracted 242nm | Ch1 254nm

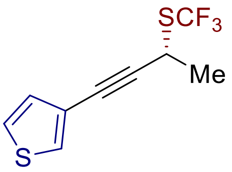

2q

Racemic trace:

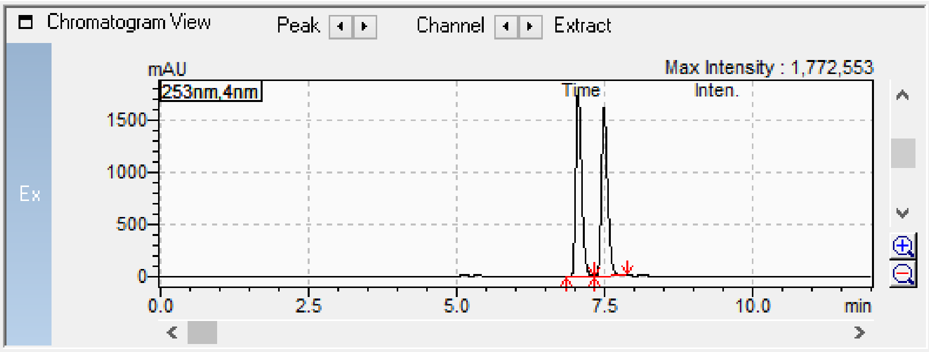

Results View - Peak Table

Peak Table | Compound | Group | Calibration Curve

Peak#	Ret. Time	Mark	Area	Height	Area%
1	7.055	M	12118491	1772062	49.954
2	7.489	V M	12141027	1613900	50.046
Total			24259518	3385963	100.000

Extracted 253nm | Ch1 254nm

Enantioenriched trace:

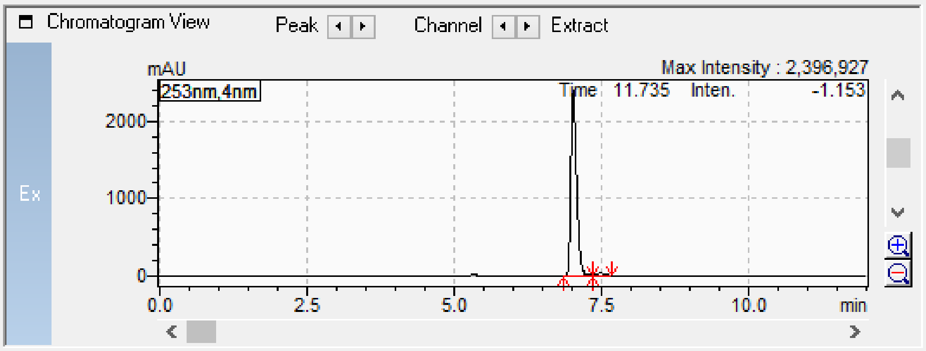

Results View - Peak Table

Peak Table | Compound | Group | Calibration Curve

Peak#	Ret. Time	Mark	Area	Height	Area%
1	7.020	M	16085620	2390065	98.537
2	7.483	V M	238766	30067	1.463
Total			16324386	2420131	100.000

Extracted 253nm | Ch1 254nm

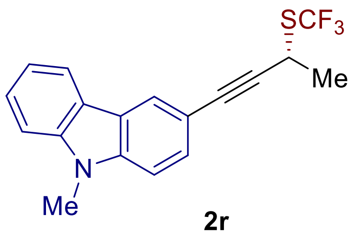

2r

Racemic trace:

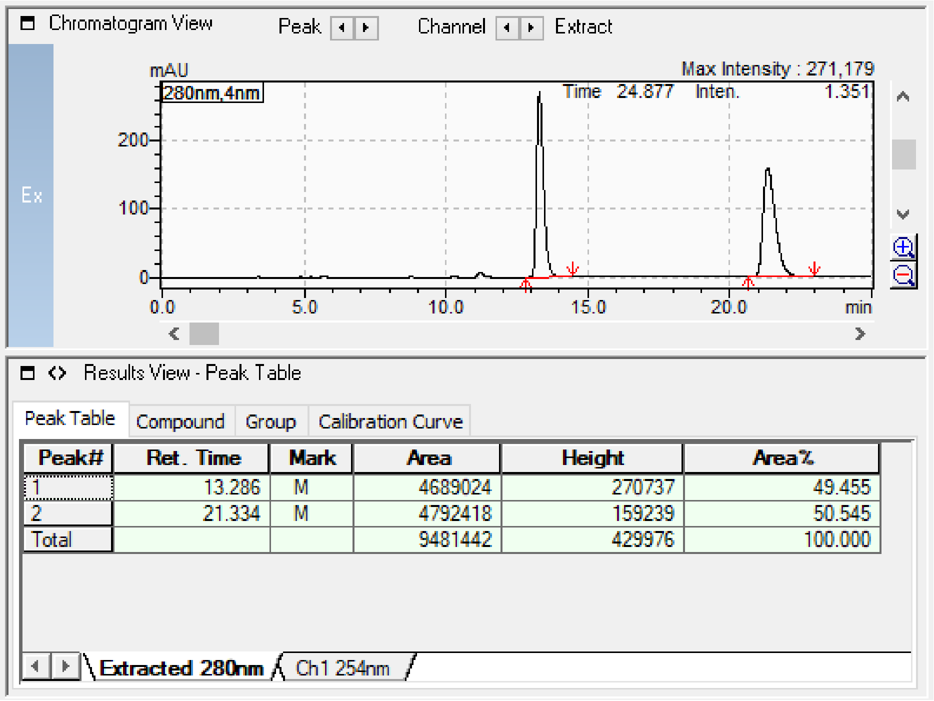

Peak#	Ret. Time	Mark	Area	Height	Area%
1	13.286	M	4689024	270737	49.455
2	21.334	M	4792418	159239	50.545
Total			9481442	429976	100.000

Enantioenriched trace:

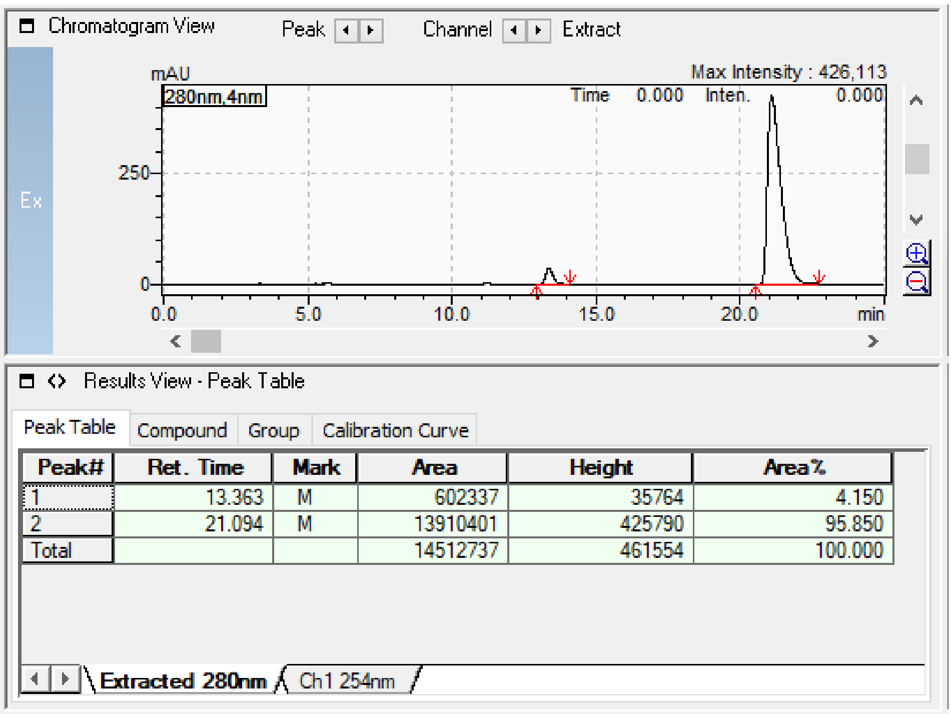

Peak#	Ret. Time	Mark	Area	Height	Area%
1	13.363	M	602337	35764	4.150
2	21.094	M	13910401	425790	95.850
Total			14512737	461554	100.000

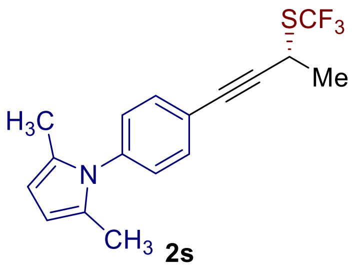

2s

Racemic trace:

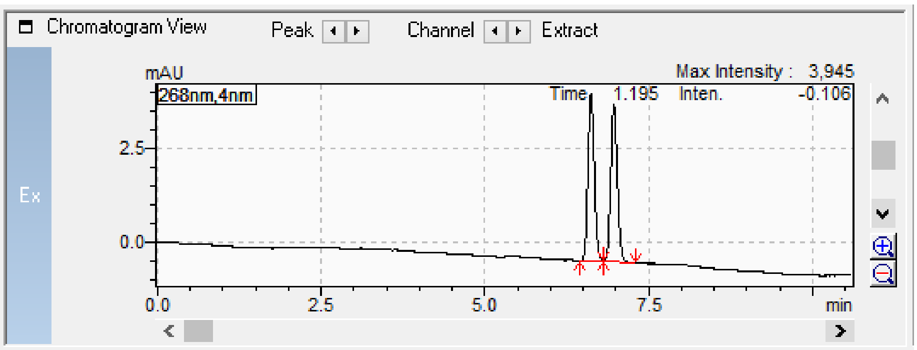

Results View - Peak Table

Peak Table | Compound | Group | Calibration Curve

Peak#	Ret. Time	Mark	Area	Height	Area%
1	6.616	M	29599	4412	50.160
2	6.966	V M	29410	4165	49.840
Total			59009	8576	100.000

Extracted 268nm | Ch1 254nm

Enantioenriched trace:

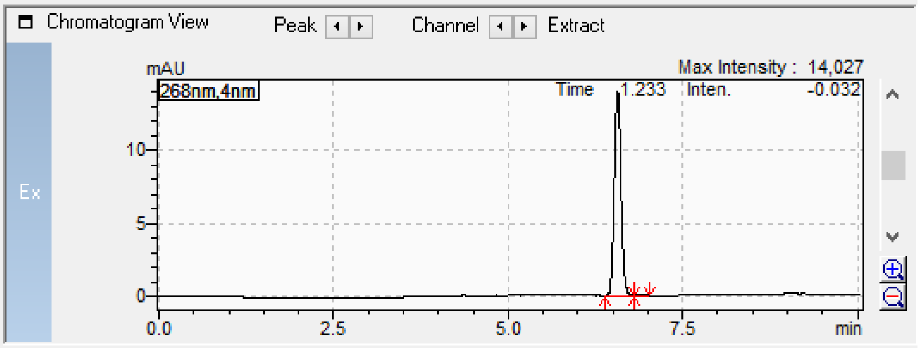

Results View - Peak Table

Peak Table | Compound | Group | Calibration Curve

Peak#	Ret. Time	Mark	Area	Height	Area%
1	6.566		92774	13949	99.332
2	6.914	M	624	104	0.668
Total			93398	14053	100.000

Extracted 268nm | Ch1 254nm

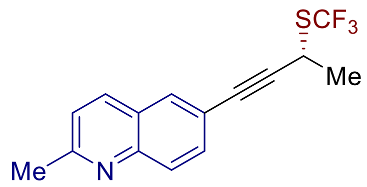

2t

Racemic trace:

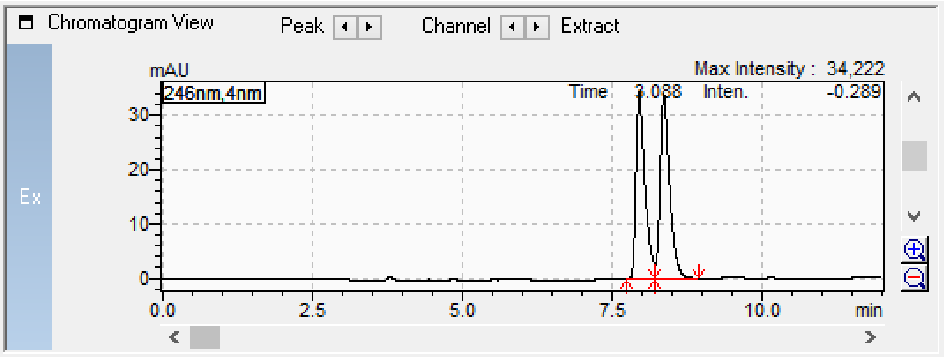

Results View - Peak Table

Peak Table | Compound | Group | Calibration Curve

Peak#	Ret. Time	Mark	Area	Height	Area%
1	7.953	M	360915	34362	49.004
2	8.361	V M	375579	34181	50.996
Total			736494	68543	100.000

Extracted 246nm | Ch1 254nm

Enantioenriched trace:

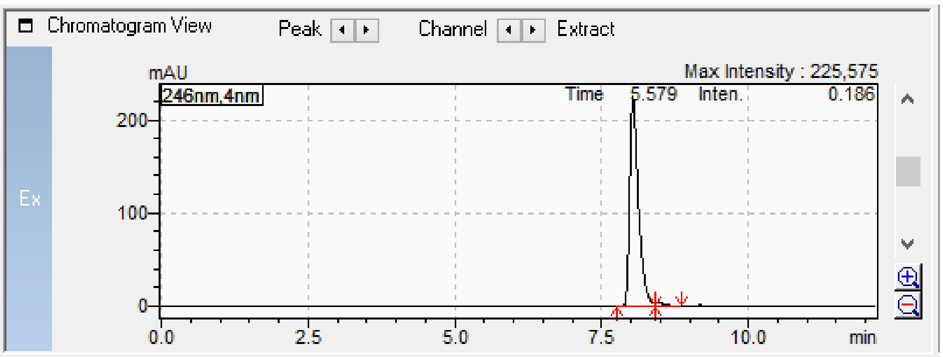

Results View - Peak Table

Peak Table | Compound | Group | Calibration Curve

Peak#	Ret. Time	Mark	Area	Height	Area%
1	8.033	M	2309131	224988	98.441
2	8.454	V M	36569	3854	1.559
Total			2345699	228843	100.000

Extracted 246nm | Ch1 254nm

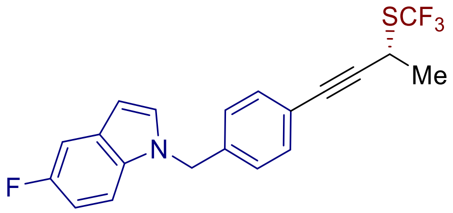

2u

Racemic trace:

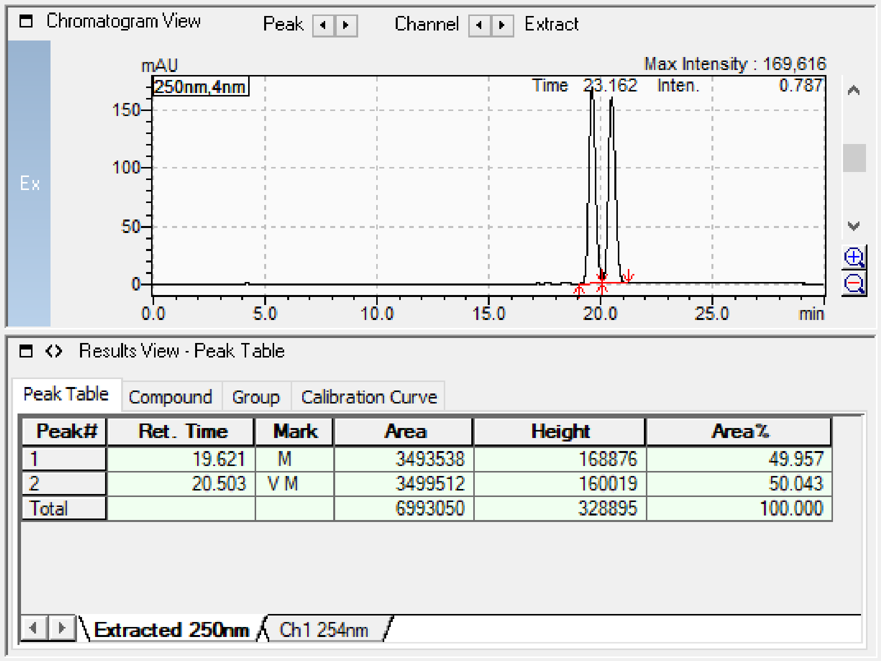

Peak#	Ret. Time	Mark	Area	Height	Area%
1	19.621	M	3493538	168876	49.957
2	20.503	V M	3499512	160019	50.043
Total			6993050	328895	100.000

Enantioenriched trace:

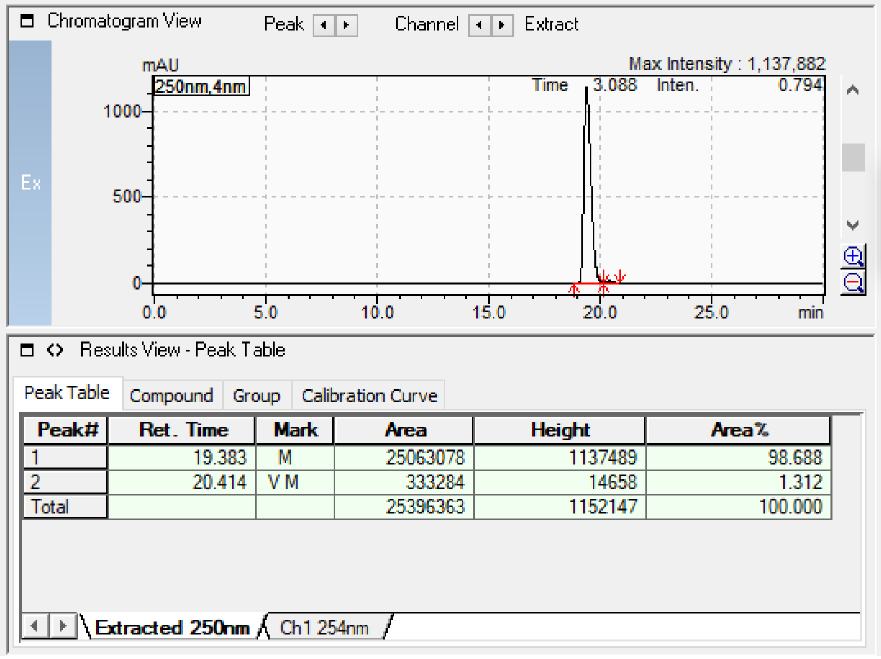

Peak#	Ret. Time	Mark	Area	Height	Area%
1	19.383	M	25063078	1137489	98.688
2	20.414	V M	333284	14658	1.312
Total			25396363	1152147	100.000

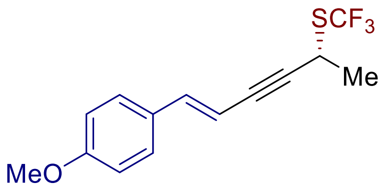

2v

Racemic trace:

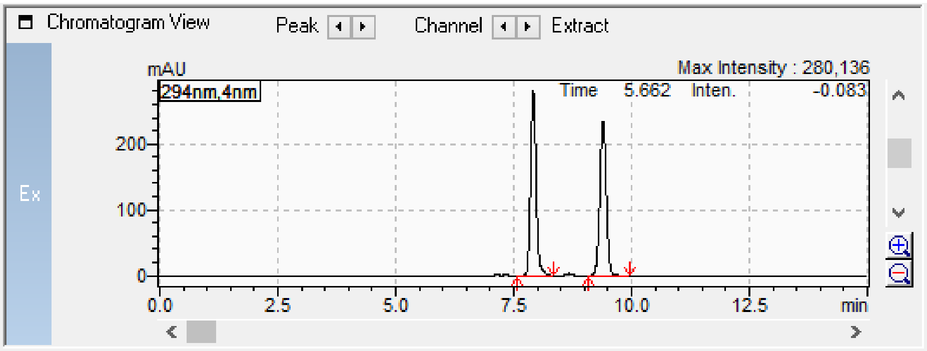

Results View - Peak Table

Peak Table | Compound | Group | Calibration Curve

Peak#	Ret. Time	Mark	Area	Height	Area%
1	7.916	M	2263012	279731	50.623
2	9.396	M	2207342	234010	49.377
Total			4470354	513741	100.000

Extracted 294nm

Enantioenriched trace:

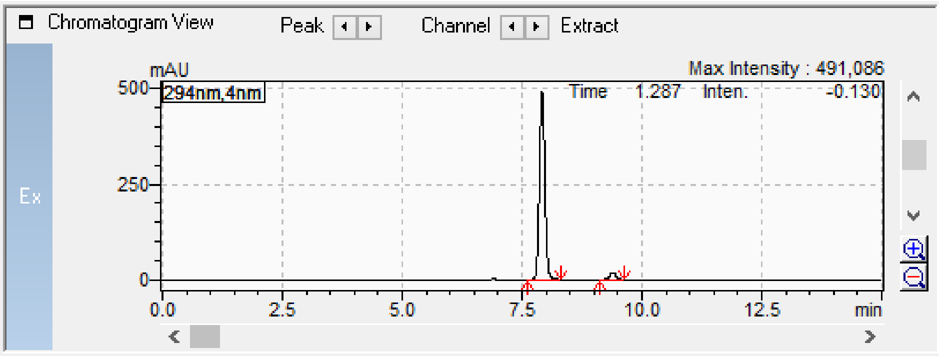

Results View - Peak Table

Peak Table | Compound | Group | Calibration Curve

Peak#	Ret. Time	Mark	Area	Height	Area%
1	7.918	M	3892662	490898	95.702
2	9.390	M	174811	18739	4.298
Total			4067473	509637	100.000

Extracted 294nm

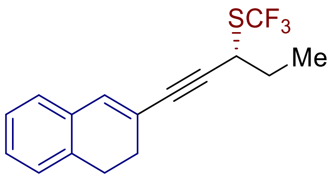

2w

Racemic trace:

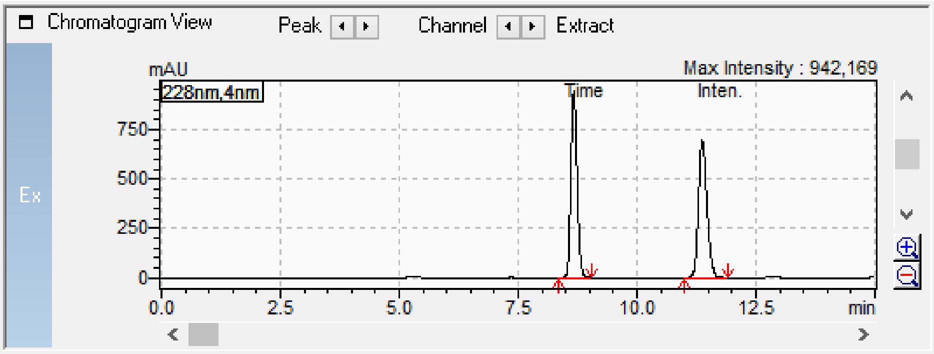

Results View - Peak Table

Peak Table | Compound | Group | Calibration Curve

Peak#	Ret. Time	Mark	Area	Height	Area%
1	8.667	M	8553250	939500	49.724
2	11.372	M	8648222	693409	50.276
Total			17201472	1632908	100.000

Extracted 228nm | Ch1 254nm

Enantioenriched trace:

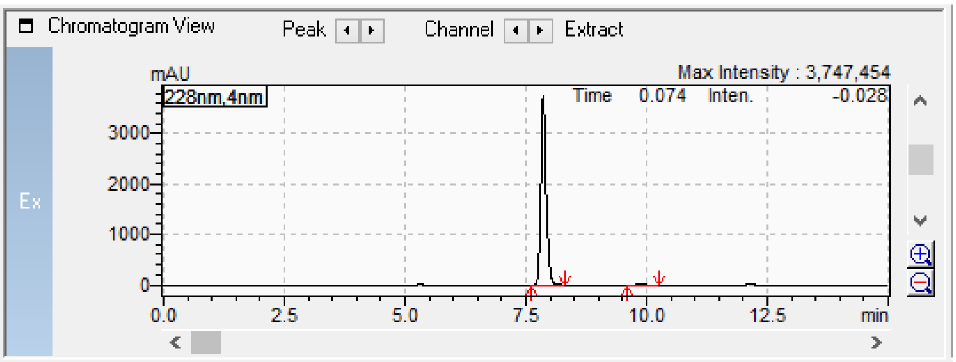

Results View - Peak Table

Peak Table | Compound | Group | Calibration Curve

Peak#	Ret. Time	Mark	Area	Height	Area%
1	7.859	M	28730562	3746924	99.417
2	9.893	M	168594	14327	0.583
Total			28899155	3761251	100.000

Extracted 228nm | Ch1 254nm

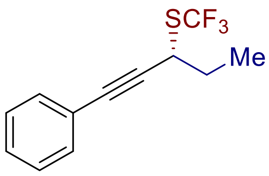

4a

Racemic trace:

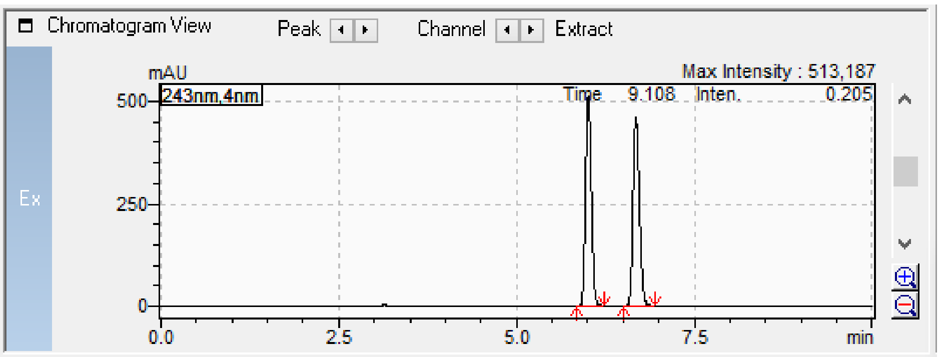

Results View - Peak Table

Peak Table | Compound | Group | Calibration Curve

Peak#	Ret. Time	Mark	Area	Height	Area%
1	6.007	M	2873441	511968	50.023
2	6.680	M	2870744	459028	49.977
Total			5744185	970996	100.000

Extracted 243nm | Ch1 254nm

Enantioenriched trace:

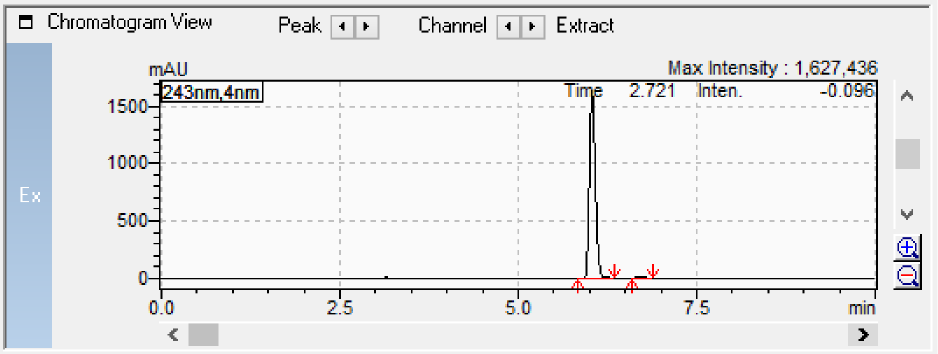

Results View - Peak Table

Peak Table | Compound | Group | Calibration Curve

Peak#	Ret. Time	Mark	Area	Height	Area%
1	6.035	M	9172658	1619442	99.335
2	6.716	M	61447	10006	0.665
Total			9234104	1629448	100.000

Extracted 243nm | Ch1 254nm

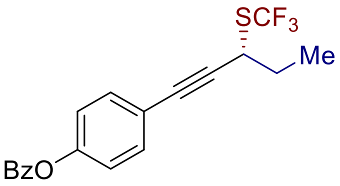

4b

Racemic trace:

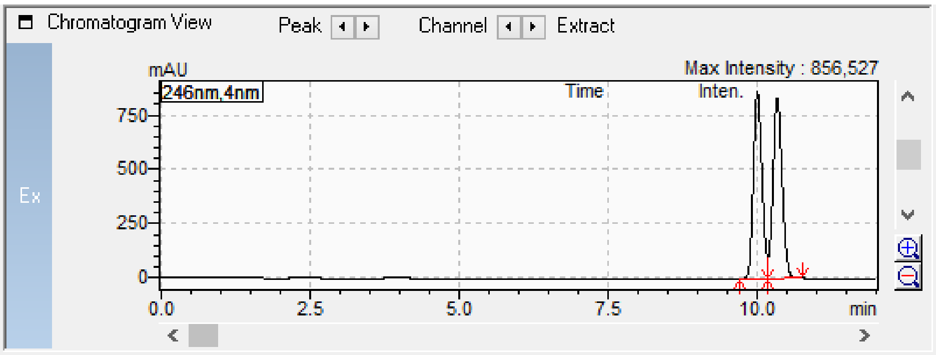

Results View - Peak Table

Peak Table | Compound | Group | Calibration Curve

Peak#	Ret. Time	Mark	Area	Height	Area%
1	10.005	M	8705736	857779	49.654
2	10.339	V M	8826918	827772	50.346
Total			17532655	1685551	100.000

Extracted 246nm | Ch1 254nm

Enantioenriched trace:

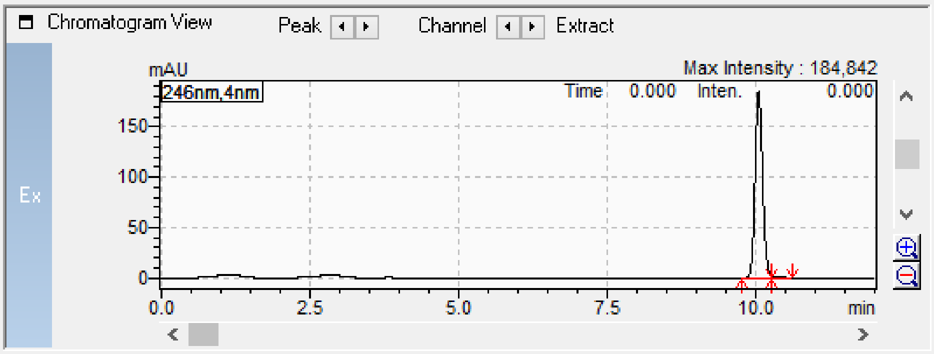

Results View - Peak Table

Peak Table | Compound | Group | Calibration Curve

Peak#	Ret. Time	Mark	Area	Height	Area%
1	10.050	M	1628751	184389	98.920
2	10.266	V M	17779	2286	1.080
Total			1646530	186675	100.000

Extracted 246nm | Ch1 254nm

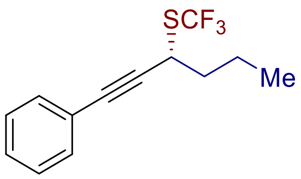

4c

Racemic trace:

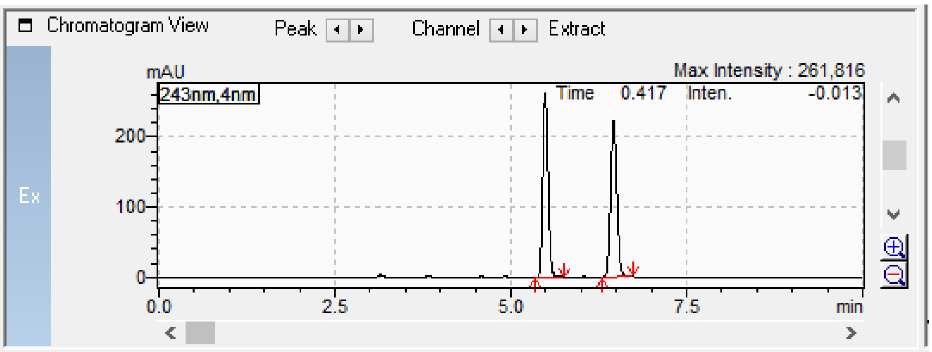

Results View - Peak Table

Peak Table | Compound | Group | Calibration Curve

Peak#	Ret. Time	Mark	Area	Height	Area%
1	5.489	M	1347942	260319	50.000
2	6.459	M	1347929	222118	50.000
Total			2695871	482437	100.000

Extracted 243nm | Ch1 254nm

Enantioenriched trace:

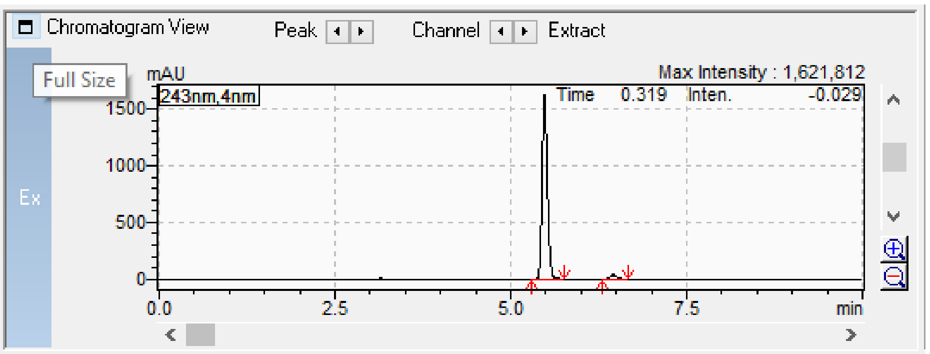

Results View - Peak Table

Peak Table | Compound | Group | Calibration Curve

Peak#	Ret. Time	Mark	Area	Height	Area%
1	5.478	M	8399991	1621163	97.445
2	6.455	M	220229	36481	2.555
Total			8620220	1657644	100.000

Extracted 243nm | Ch1 254nm

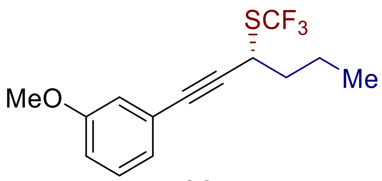

4d

Racemic trace:

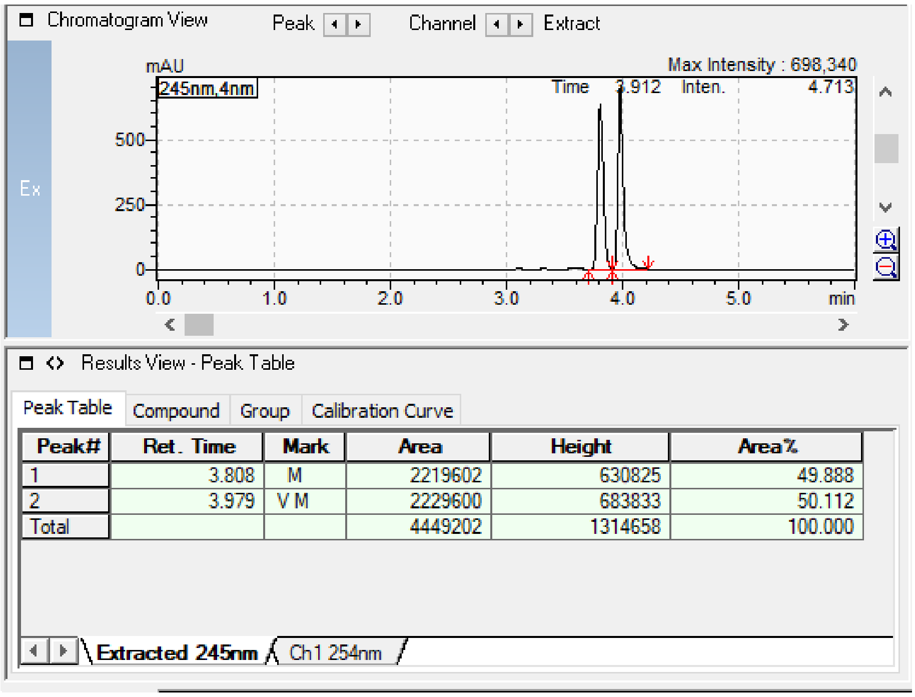

Peak#	Ret. Time	Mark	Area	Height	Area%
1	3.808	M	2219602	630825	49.888
2	3.979	V M	2229600	683833	50.112
Total			4449202	1314658	100.000

Enantioenriched trace:

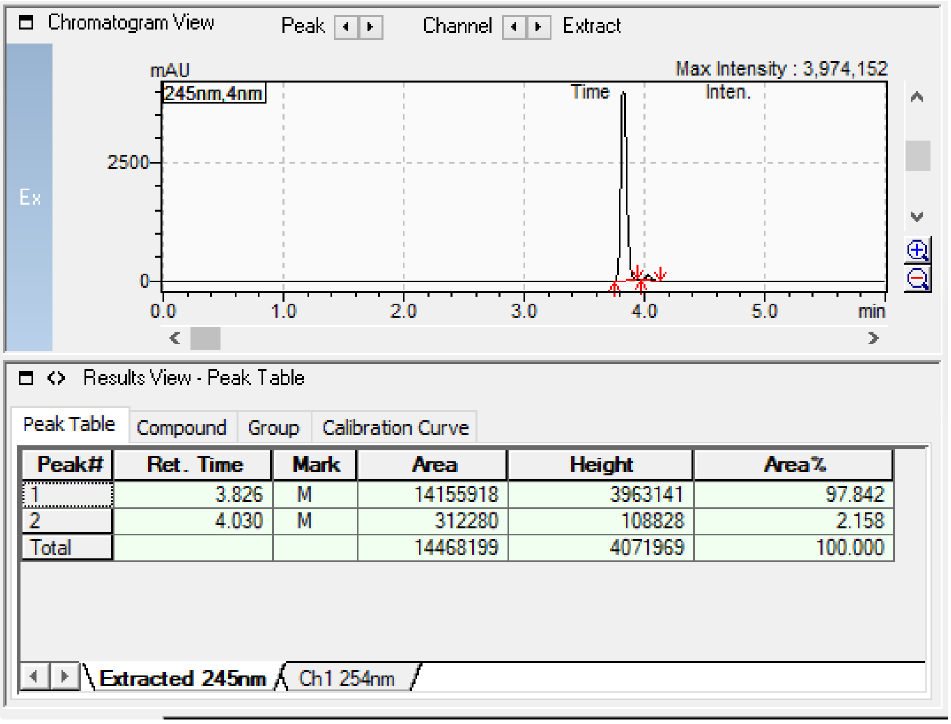

Peak#	Ret. Time	Mark	Area	Height	Area%
1	3.826	M	14155918	3963141	97.842
2	4.030	M	312280	108828	2.158
Total			14468199	4071969	100.000

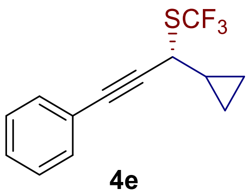

4e

Racemic trace:

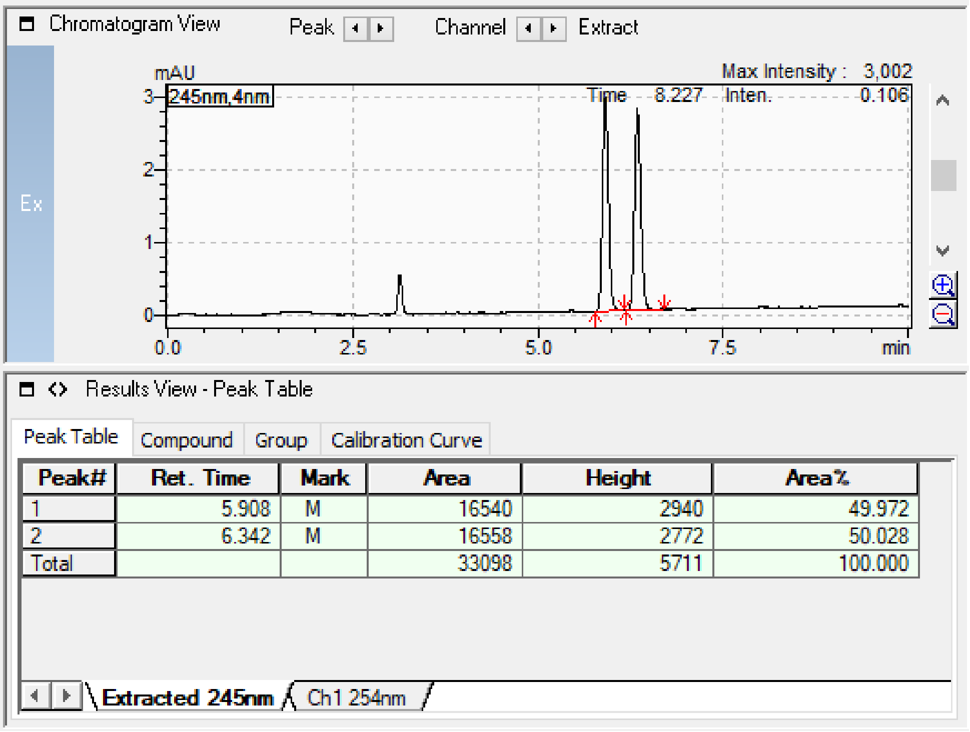

Peak#	Ret. Time	Mark	Area	Height	Area%
1	5.908	M	16540	2940	49.972
2	6.342	M	16558	2772	50.028
Total			33098	5711	100.000

Enantioenriched trace:

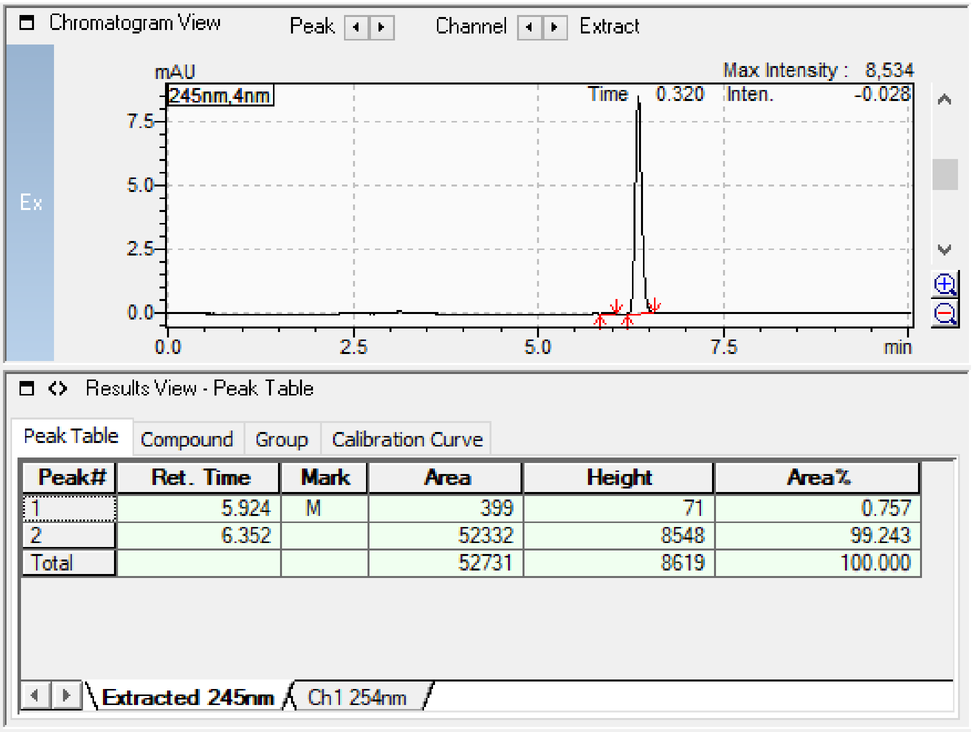

Peak#	Ret. Time	Mark	Area	Height	Area%
1	5.924	M	399	71	0.757
2	6.352		52332	8548	99.243
Total			52731	8619	100.000

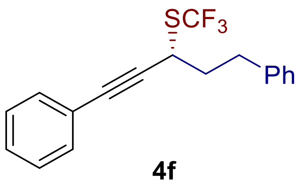

4f

Racemic trace:

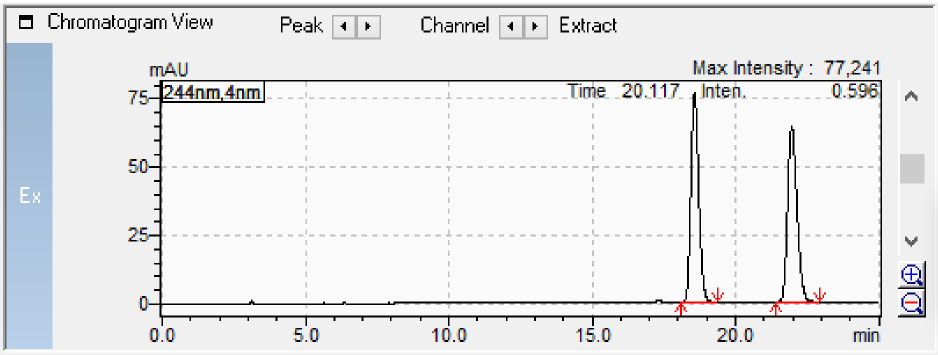

Results View - Peak Table

Peak Table | Compound | Group | Calibration Curve

Peak#	Ret. Time	Mark	Area	Height	Area%
1	18.566	M	1467718	76656	49.983
2	21.956	M	1468728	64261	50.017
Total			2936446	140917	100.000

Extracted 244nm | Ch1 254nm

Enantioenriched trace:

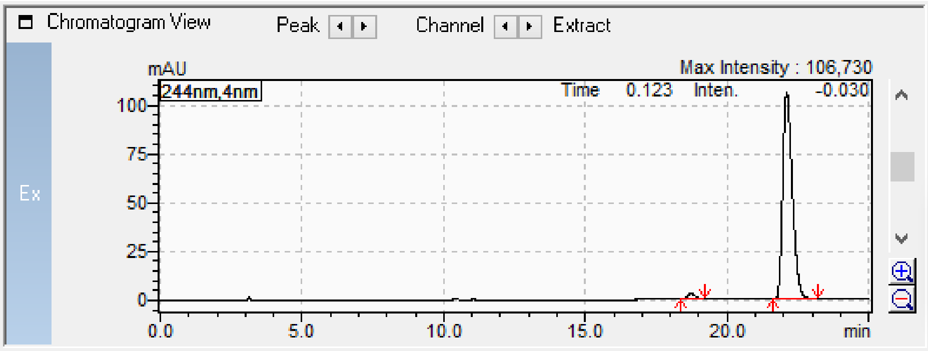

Results View - Peak Table

Peak Table | Compound | Group | Calibration Curve

Peak#	Ret. Time	Mark	Area	Height	Area%
1	18.747		53825	2899	2.119
2	22.103		2486683	106106	97.881
Total			2540508	109005	100.000

Extracted 244nm | Ch1 254nm

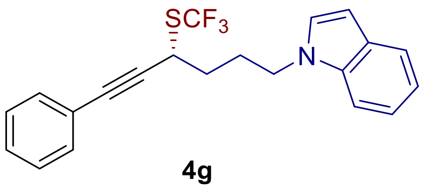

4g

Racemic trace:

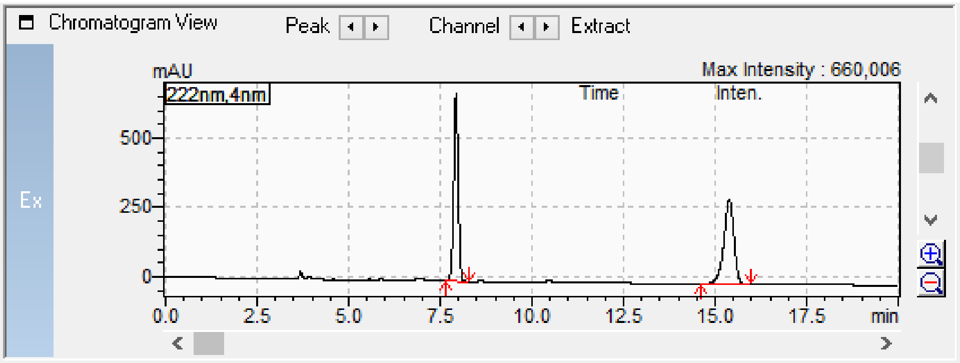

Results View - Peak Table

Peak Table | Compound | Group | Calibration Curve

Peak#	Ret. Time	Mark	Area	Height	Area%
1	7.929	M	5781439	675019	49.813
2	15.387	M	5824787	306124	50.187
Total			11606226	981143	100.000

Extracted 222nm | Ch1 254nm

Enantioenriched trace:

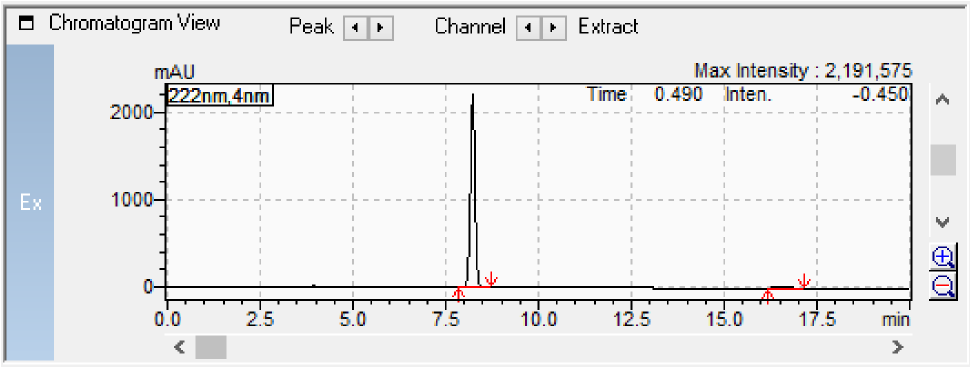

Results View - Peak Table

Peak Table | Compound | Group | Calibration Curve

Peak#	Ret. Time	Mark	Area	Height	Area%
1	8.226	M	18690766	2189064	98.080
2	16.635	M	365978	18613	1.920
Total			19056743	2207676	100.000

Extracted 222nm | Ch1 254nm

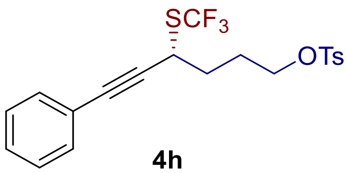

4h

Racemic trace:

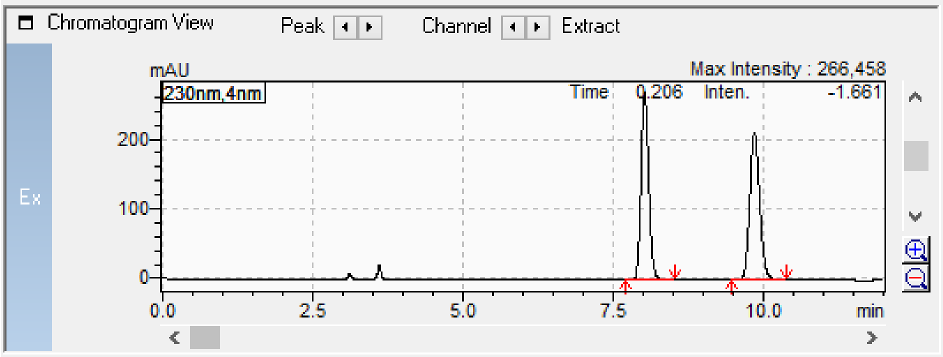

Results View - Peak Table

Peak Table | Compound | Group | Calibration Curve

Peak#	Ret. Time	Mark	Area	Height	Area%
1	8.025	M	2403481	267610	49.775
2	9.853	M	2425223	210826	50.225
Total			4828704	478436	100.000

Extracted 230nm | Ch1 254nm

Enantioenriched trace:

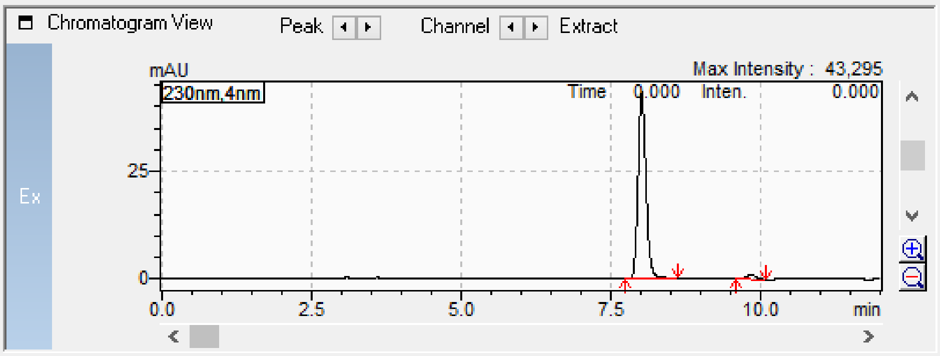

Results View - Peak Table

Peak Table | Compound | Group | Calibration Curve

Peak#	Ret. Time	Mark	Area	Height	Area%
1	8.016	M	402230	43218	97.592
2	9.846	M	9926	944	2.408
Total			412156	44162	100.000

Extracted 230nm | Ch1 254nm

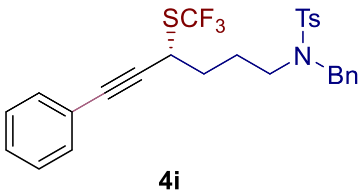

4i

Racemic trace:

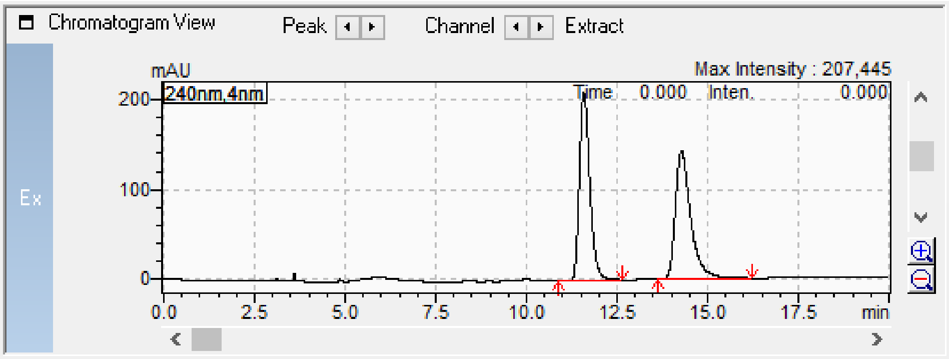

Results View - Peak Table

Peak Table | Compound | Group | Calibration Curve

Peak#	Ret. Time	Mark	Area	Height	Area%
1	11.588	M	4193709	208378	49.863
2	14.277	M	4216832	142788	50.137
Total			8410541	351166	100.000

Extracted 240nm | Ch1 254nm

Enantioenriched trace:

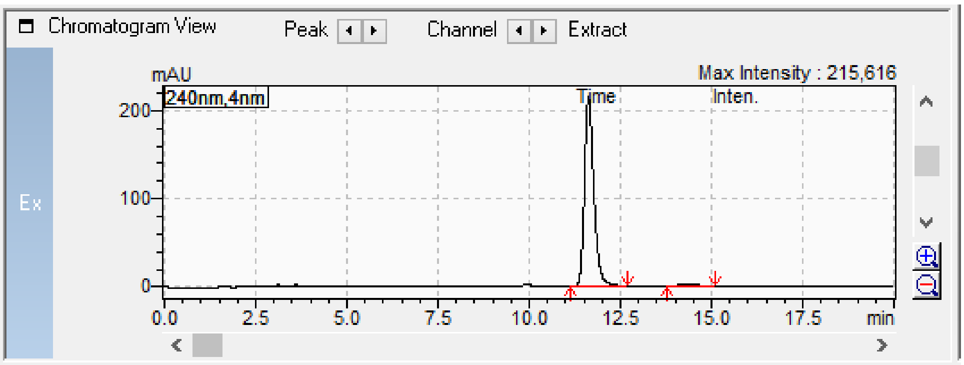

Results View - Peak Table

Peak Table | Compound | Group | Calibration Curve

Peak#	Ret. Time	Mark	Area	Height	Area%
1	11.624	M	3800967	214579	98.401
2	14.317	M	61779	2024	1.599
Total			3862747	216603	100.000

Extracted 240nm | Ch1 254nm

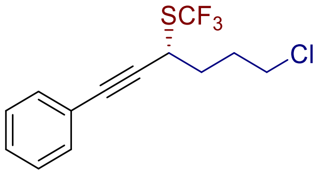

4j

Racemic trace:

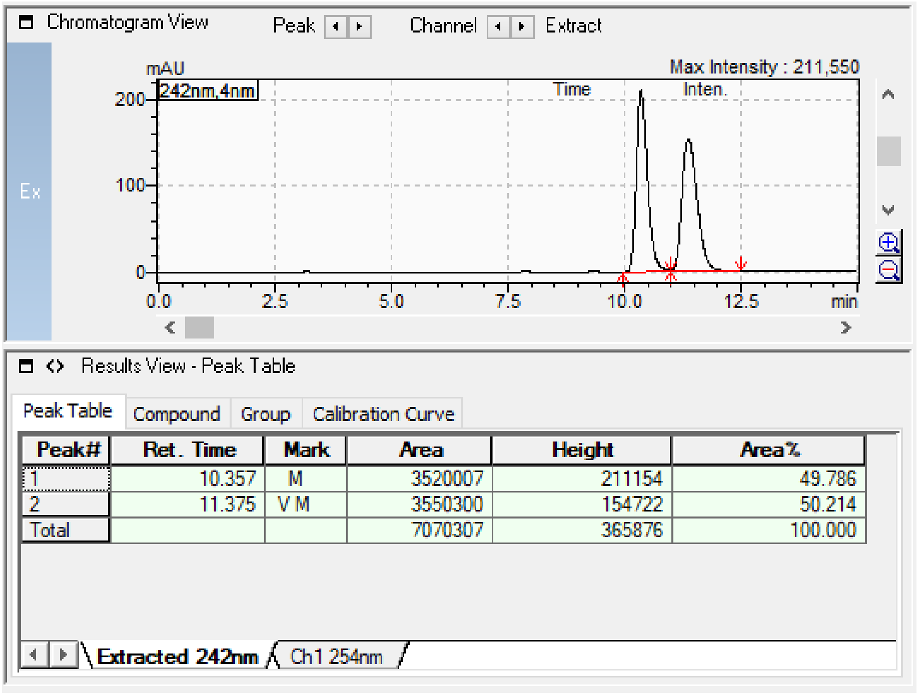

Peak#	Ret. Time	Mark	Area	Height	Area%
1	10.357	M	3520007	211154	49.786
2	11.375	V M	3550300	154722	50.214
Total			7070307	365876	100.000

Enantioenriched trace:

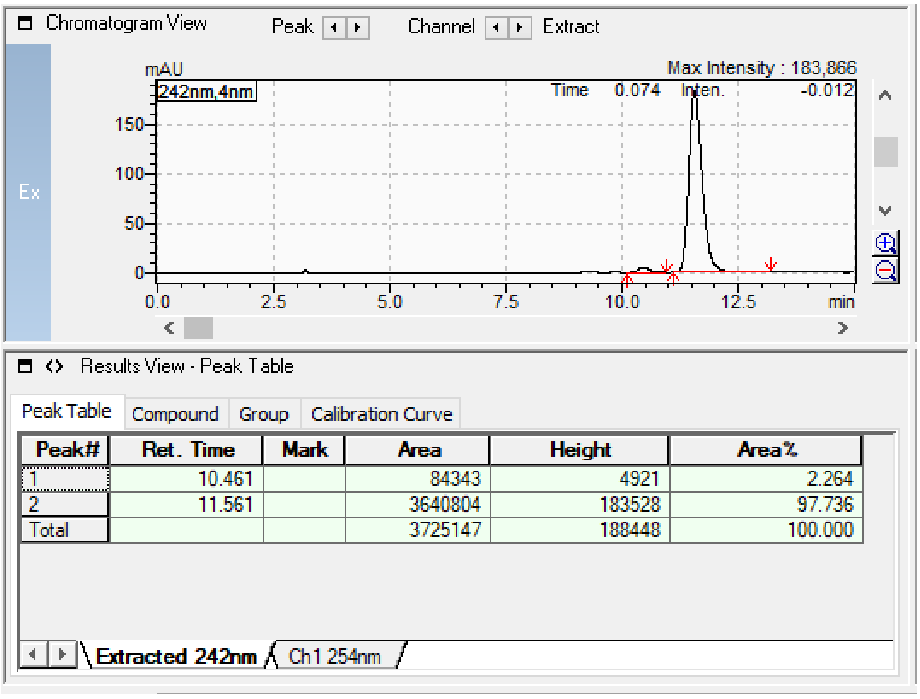

Peak#	Ret. Time	Mark	Area	Height	Area%
1	10.461		84343	4921	2.264
2	11.561		3640804	183528	97.736
Total			3725147	188448	100.000

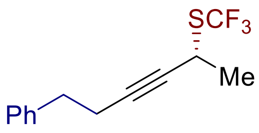

4k

Racemic trace:

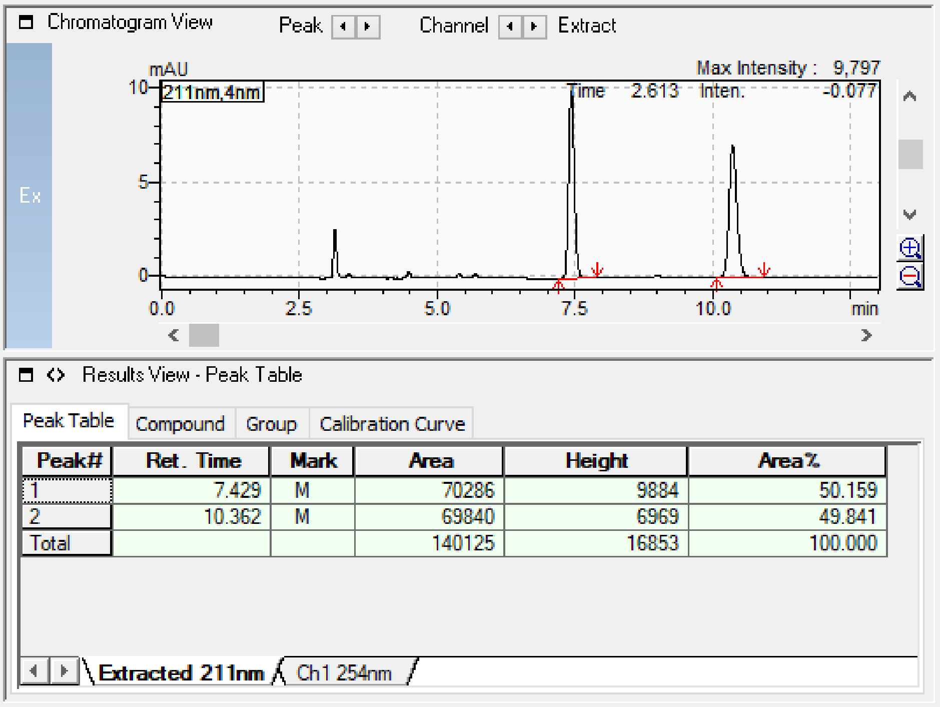

Peak#	Ret. Time	Mark	Area	Height	Area%
1	7.429	M	70286	9884	50.159
2	10.362	M	69840	6969	49.841
Total			140125	16853	100.000

Enantioenriched trace:

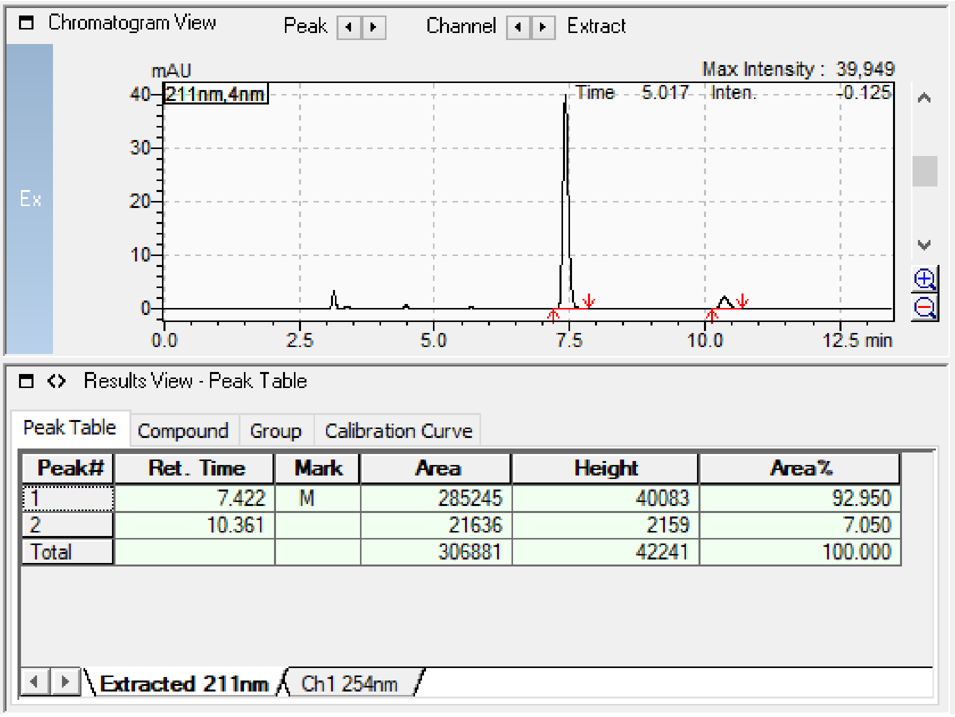

Peak#	Ret. Time	Mark	Area	Height	Area%
1	7.422	M	285245	40083	92.950
2	10.361		21636	2159	7.050
Total			306881	42241	100.000

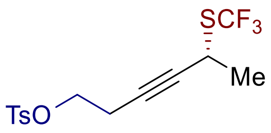

4l

Racemic trace:

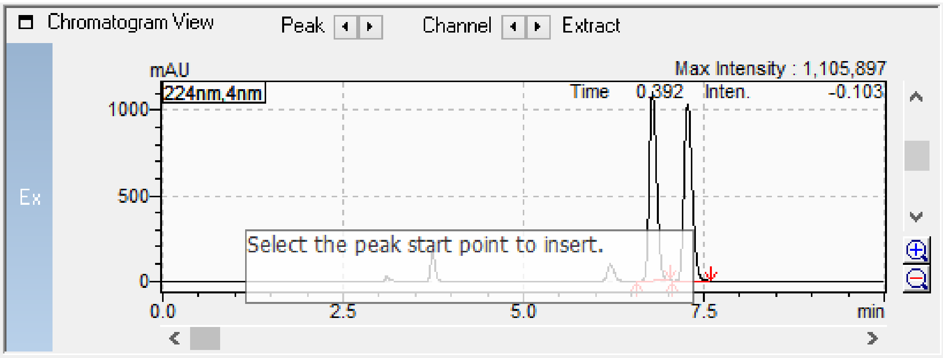

Results View - Peak Table

Peak Table | Compound | Group | Calibration Curve

Peak#	Ret. Time	Mark	Area	Height	Area%
1	6.789	M	8081515	1104363	49.708
2	7.276	M	8176479	1036233	50.292
Total			16257993	2140596	100.000

Extracted 224nm

Enantioenriched trace:

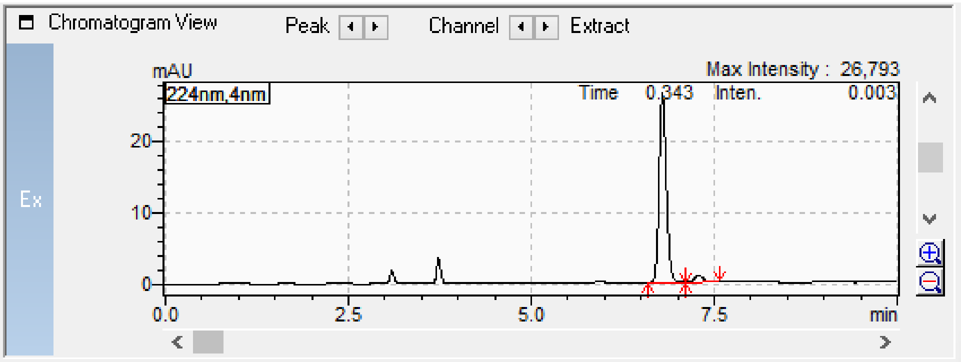

Results View - Peak Table

Peak Table | Compound | Group | Calibration Curve

Peak#	Ret. Time	Mark	Area	Height	Area%
1	6.793		183343	26479	95.894
2	7.283	V	7850	960	4.106
Total			191193	27439	100.000

Extracted 224nm | Ch1 254nm

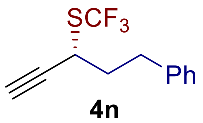

4n

Racemic trace:

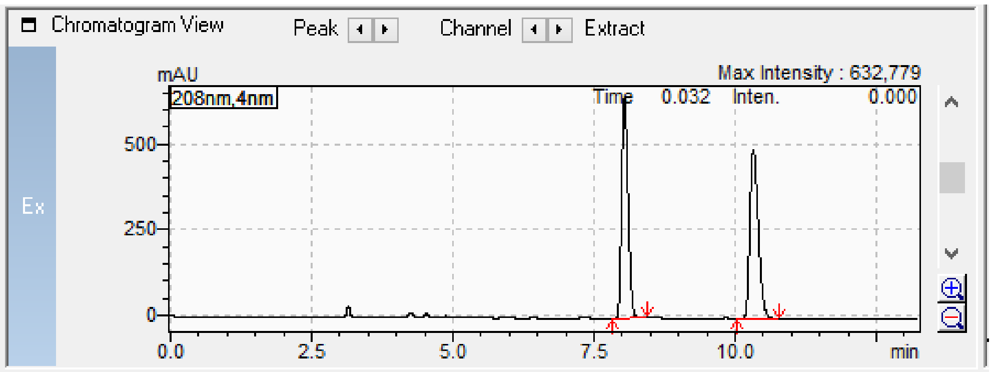

Results View - Peak Table

Peak Table | Compound | Group | Calibration Curve

Peak#	Ret. Time	Mark	Area	Height	Area%
1	8.037	M	4994925	638607	49.364
2	10.320	M	5123696	493450	50.636
Total			10118621	1132057	100.000

Extracted 208nm | Ch1 254nm

Enantioenriched trace:

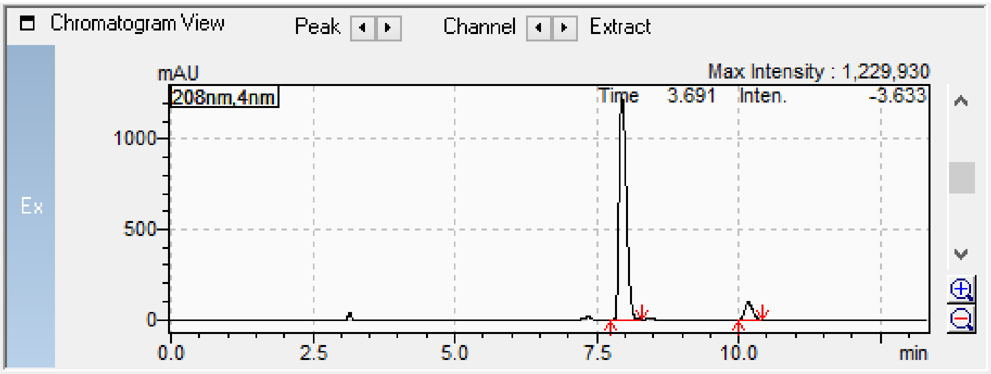

Results View - Peak Table

Peak Table | Compound | Group | Calibration Curve

Peak#	Ret. Time	Mark	Area	Height	Area%
1	7.945	M	11033507	1230700	92.089
2	10.173	M	947781	99463	7.911
Total			11981288	1330164	100.000

Extracted 208nm | Ch1 254nm

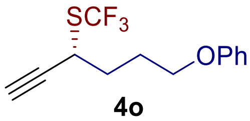

4o

Racemic trace:

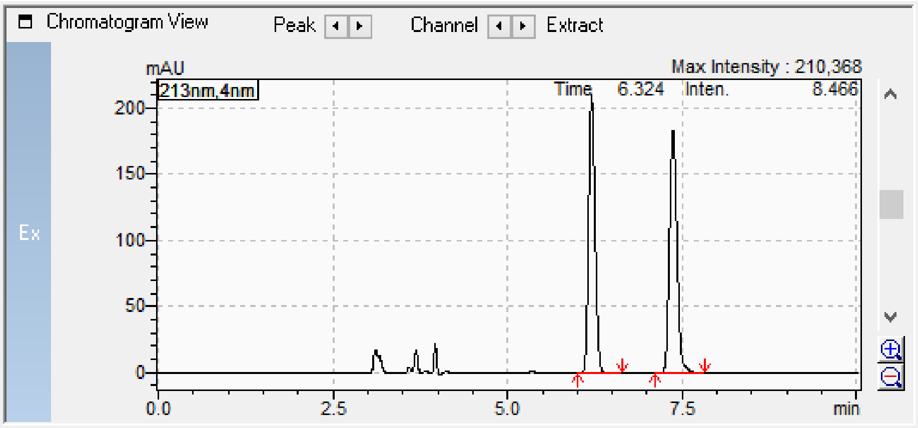

Results View - Peak Table

Peak Table | Compound | Group | Calibration Curve

Peak#	Ret. Time	Mark	Area	Height	Area%
1	6.205		1357919	210877	49.445
2	7.370		1388412	183289	50.555
Total			2746331	394165	100.000

Extracted 213nm | Ch1 254nm

Enantioenriched trace:

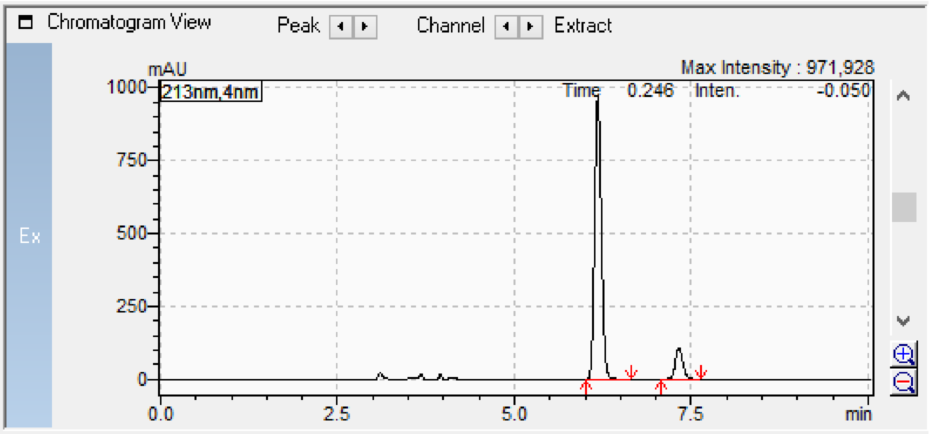

Results View - Peak Table

Peak Table | Compound | Group | Calibration Curve

Peak#	Ret. Time	Mark	Area	Height	Area%
1	6.179		6478234	968170	88.983
2	7.325		802040	106965	11.017
Total			7280274	1075135	100.000

Extracted 213nm | Ch1 254nm

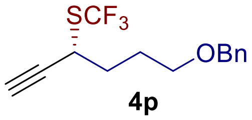

4p

Racemic trace:

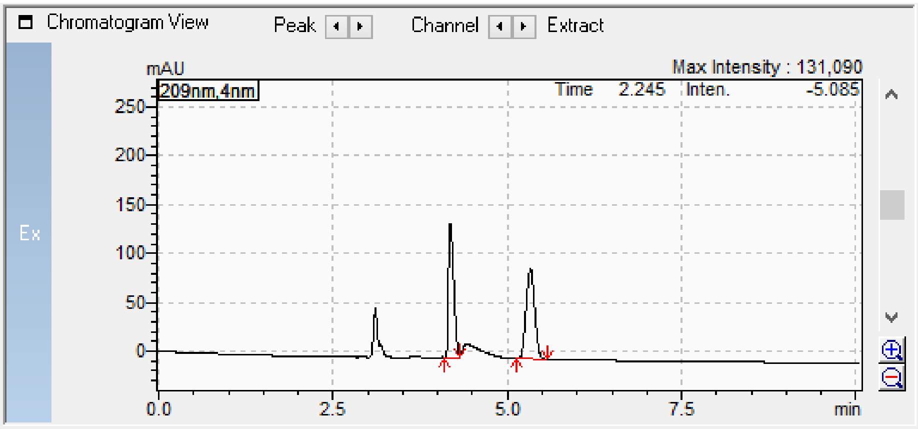

Results View - Peak Table

Peak Table | Compound | Group | Calibration Curve

Peak#	Ret. Time	Mark	Area	Height	Area%
1	4.184	M	735547	137597	48.430
2	5.331	M	783230	92601	51.570
Total			1518777	230198	100.000

Extracted 209nm | Ch1 254nm

Enantioenriched trace:

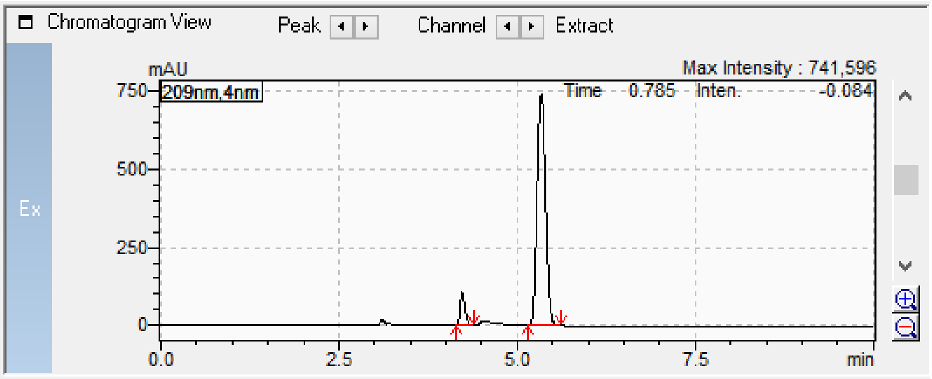

Results View - Peak Table

Peak Table | Compound | Group | Calibration Curve

Peak#	Ret. Time	Mark	Area	Height	Area%
1	4.226	M	510181	105332	7.627
2	5.340	M	6178644	741294	92.373
Total			6688825	846627	100.000

Extracted 209nm | Ch1 254nm

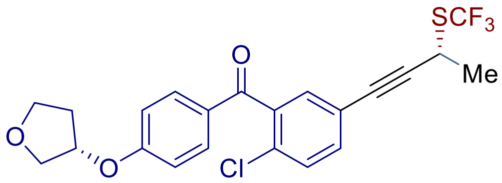

4q

Racemic trace:

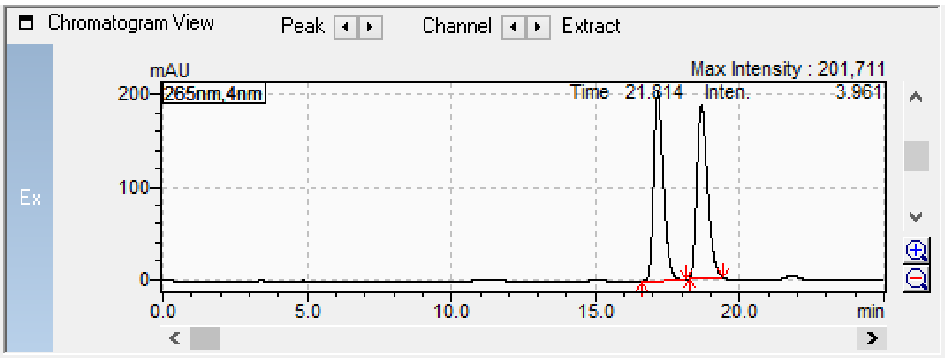

Results View - Peak Table

Peak Table | Compound | Group | Calibration Curve

Peak#	Ret. Time	Mark	Area	Height	Area%
1	17.161	M	4773084	202724	49.746
2	18.670	M	4821834	186229	50.254
Total			9594918	388953	100.000

Extracted 265nm | Ch1 254nm

Enantioenriched trace:

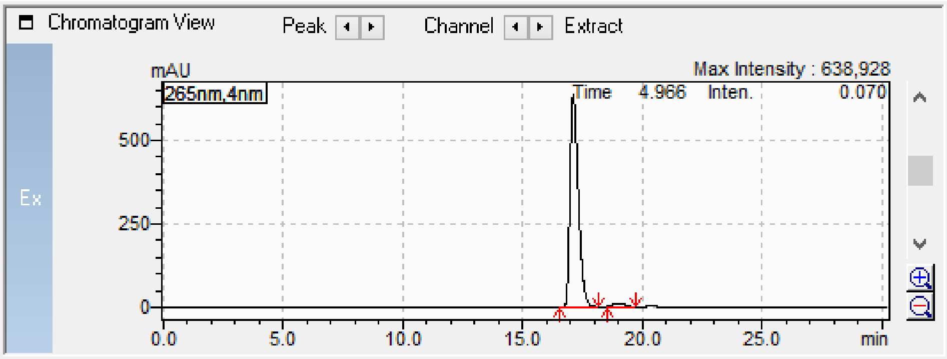

Results View - Peak Table

Peak Table | Compound | Group | Calibration Curve

Peak#	Ret. Time	Mark	Area	Height	Area%
1	17.109	M	15333453	638399	97.562
2	19.040	M	383174	11169	2.438
Total			15716627	649569	100.000

Extracted 265nm | Ch1 254nm

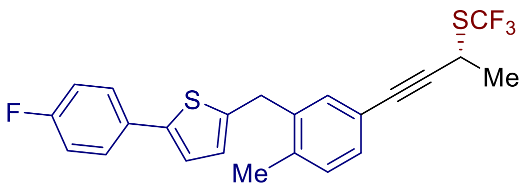

4r

Racemic trace:

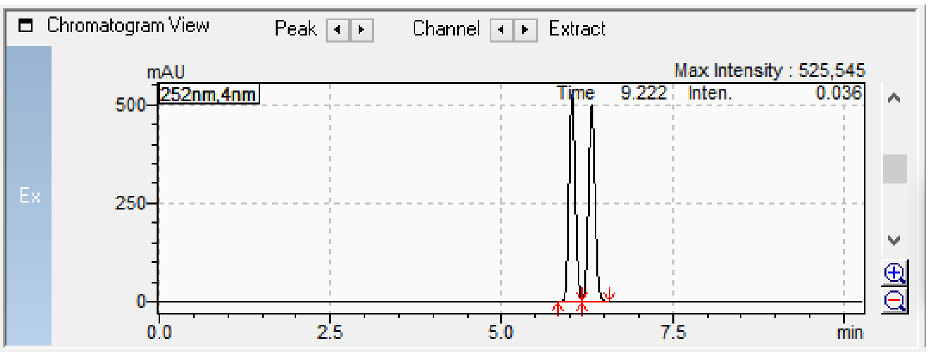

Results View - Peak Table

Peak Table | Compound | Group | Calibration Curve

Peak#	Ret. Time	Mark	Area	Height	Area%
1	6.028	M	3319524	524302	49.925
2	6.316	V M	3329454	500048	50.075
Total			6648978	1024350	100.000

Extracted 252nm | Ch1 254nm

Enantioenriched trace:

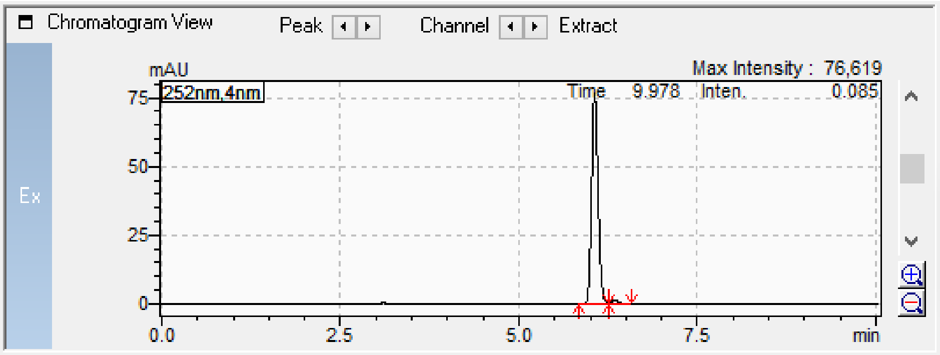

Results View - Peak Table

Peak Table | Compound | Group | Calibration Curve

Peak#	Ret. Time	Mark	Area	Height	Area%
1	6.062	M	489897	76572	98.170
2	6.352	V M	9133	1259	1.830
Total			499031	77832	100.000

Extracted 252nm | Ch1 254nm

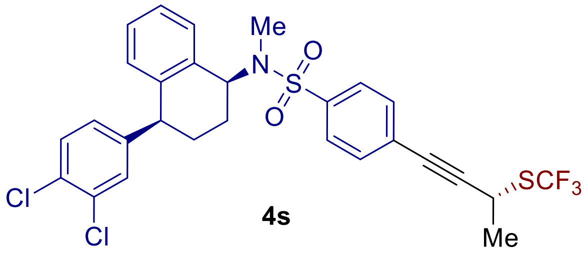

4s

Racemic trace:

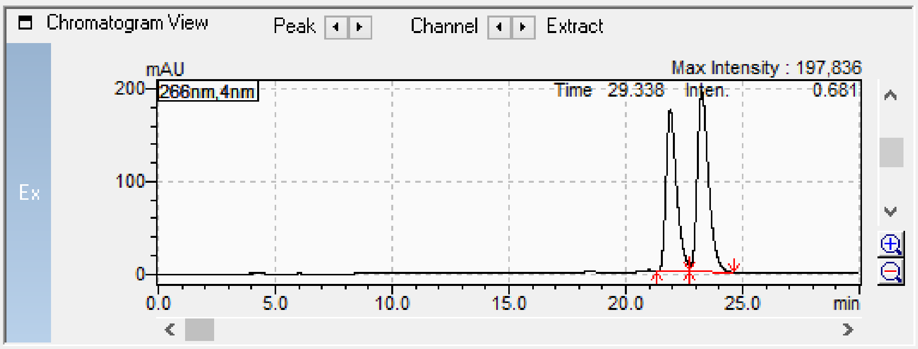

Results View - Peak Table

Peak Table | Compound | Group | Calibration Curve

Peak#	Ret. Time	Mark	Area	Height	Area%
1	21.904	M	5604551	175989	46.130
2	23.257	V M	6544997	195780	53.870
Total			12149548	371770	100.000

Extracted 266nm | Ch1 254nm

Enantioenriched trace:

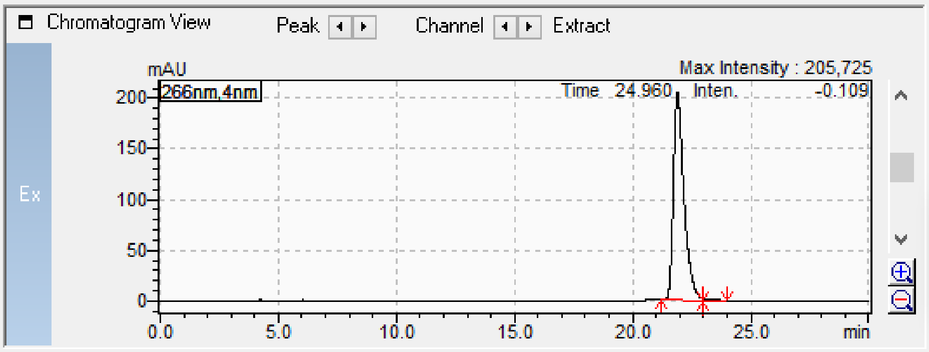

Results View - Peak Table

Peak Table | Compound | Group | Calibration Curve

Peak#	Ret. Time	Mark	Area	Height	Area%
1	21.900	M	6485788	205290	99.069
2	23.018	V M	60968	1872	0.931
Total			6546756	207162	100.000

Extracted 266nm | Ch1 254nm

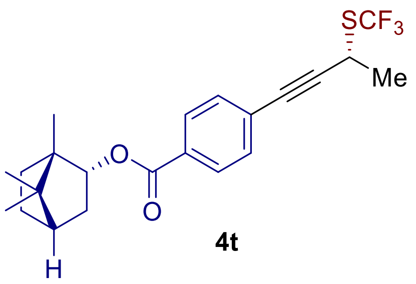

4t

Racemic trace:

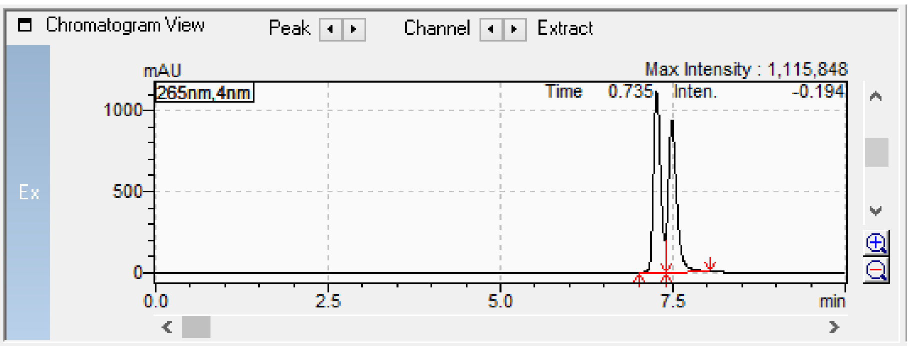

Results View - Peak Table

Peak Table | Compound | Group | Calibration Curve

Peak#	Ret. Time	Mark	Area	Height	Area%
1	7.265	M	8072393	1112336	50.331
2	7.485	V M	7966103	935761	49.669
Total			16038496	2048097	100.000

Extracted 265nm | Ch1 254nm

Enantioenriched trace:

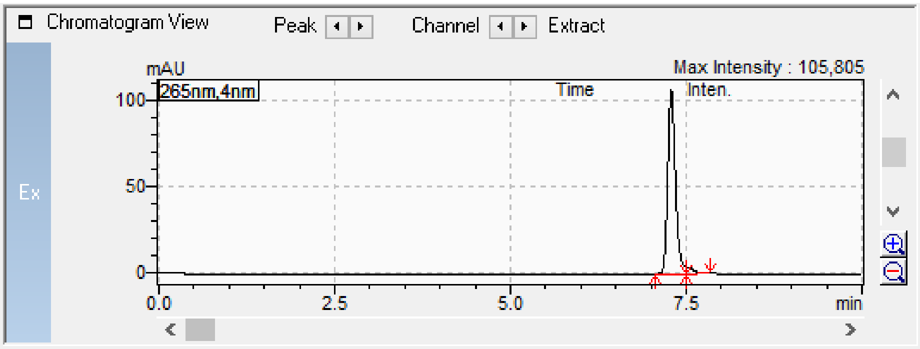

Results View - Peak Table

Peak Table | Compound | Group | Calibration Curve

Peak#	Ret. Time	Mark	Area	Height	Area%
1	7.287	M	806920	105866	96.663
2	7.506	V M	27853	3982	3.337
Total			834773	109848	100.000

Extracted 265nm | Ch1 254nm

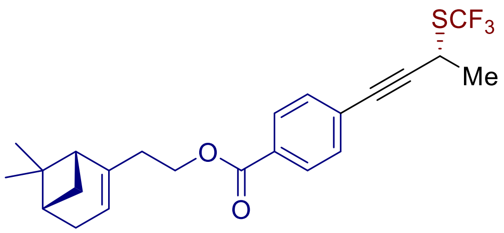

4u

Racemic trace:

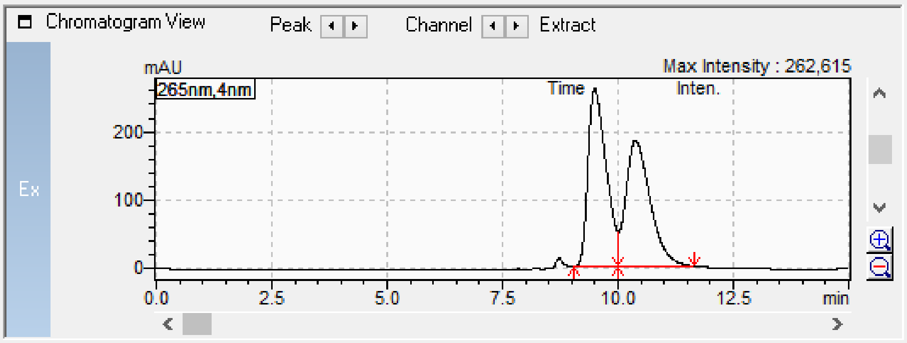

Results View - Peak Table

Peak Table | Compound | Group | Calibration Curve

Peak#	Ret. Time	Mark	Area	Height	Area%
1	9.497	M	7223847	259948	51.501
2	10.381	V M	6802820	184324	48.499
Total			14026667	444272	100.000

Extracted 265nm | Ch1 254nm

Enantioenriched trace:

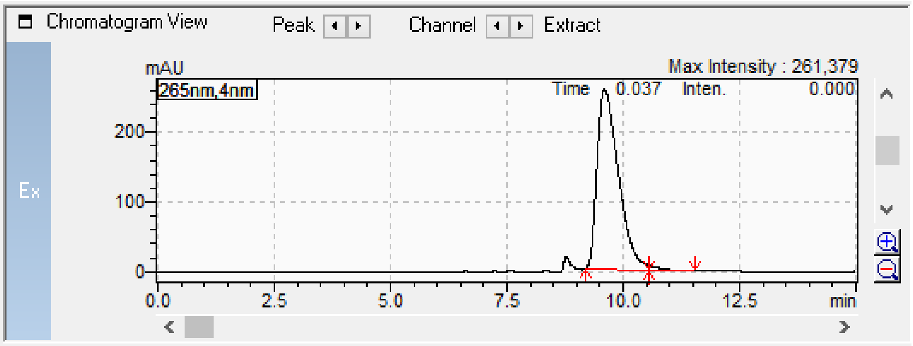

Results View - Peak Table

Peak Table | Compound | Group | Calibration Curve

Peak#	Ret. Time	Mark	Area	Height	Area%
1	9.602	M	8318536	258561	98.610
2	10.570	V M	117287	6887	1.390
Total			8435822	265448	100.000

Extracted 265nm

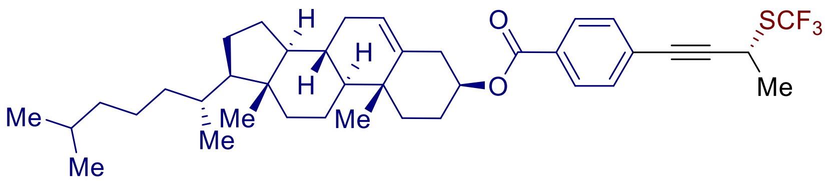

4v

Racemic trace:

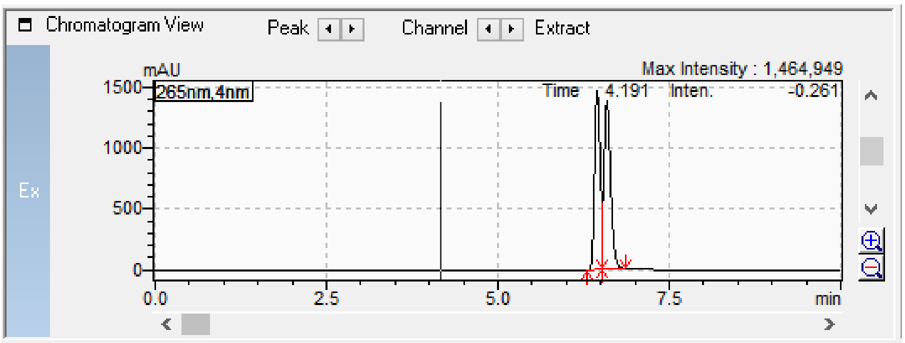

Results View - Peak Table

Peak Table | Compound | Group | Calibration Curve

Peak#	Ret. Time	Mark	Area	Height	Area%
1	6.449	M	8348830	1454737	48.296
2	6.592	V M	8938076	1376190	51.704
Total			17286906	2830927	100.000

Extracted 265nm | Ch1 254nm

Enantioenriched trace:

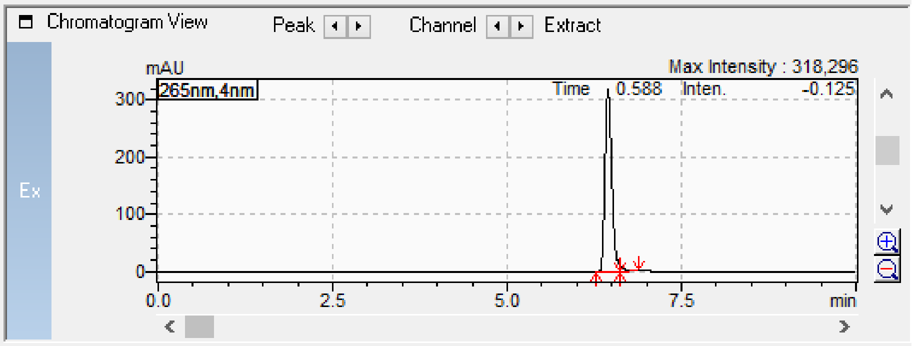

Results View - Peak Table

Peak Table | Compound | Group | Calibration Curve

Peak#	Ret. Time	Mark	Area	Height	Area%
1	6.447	M	2115822	317987	98.312
2	6.634	V M	36326	9170	1.688
Total			2152147	327157	100.000

Extracted 265nm | Ch1 254nm

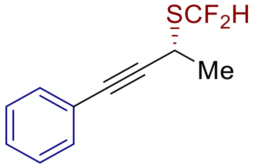

6a

Racemic trace:

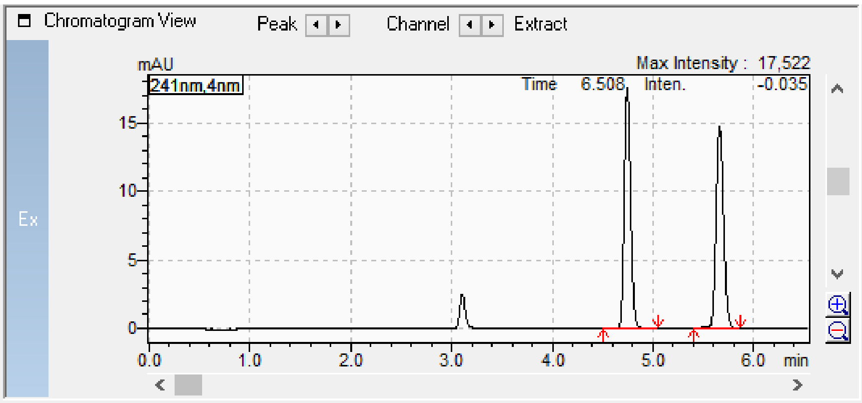

Results View - Peak Table

Peak Table | Compound | Group | Calibration Curve

Peak#	Ret. Time	Mark	Area	Height	Area%
1	4.745	M	77000	17421	49.936
2	5.661	M	77197	14747	50.064
Total			154196	32168	100.000

Extracted 241nm | Ch1 254nm

Enantioenriched trace:

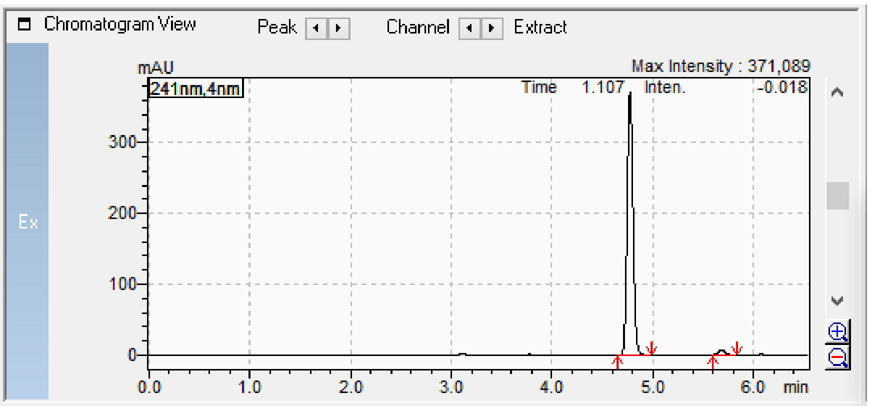

Results View - Peak Table

Peak Table | Compound | Group | Calibration Curve

Peak#	Ret. Time	Mark	Area	Height	Area%
1	4.773	M	1476526	369508	97.640
2	5.683	M	35692	7243	2.360
Total			1512219	376751	100.000

Extracted 241nm | Ch1 254nm

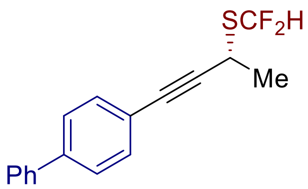

6b

Racemic trace:

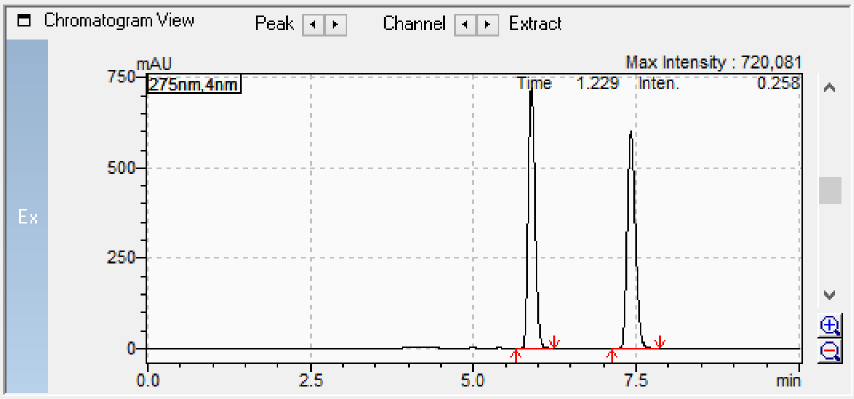

Results View - Peak Table

Peak Table | Compound | Group | Calibration Curve

Peak#	Ret. Time	Mark	Area	Height	Area%
1	5.892	M	5250903	718044	50.031
2	7.420	M	5244445	601072	49.969
Total			10495348	1319116	100.000

Extracted 275nm | Ch1 254nm

Enantioenriched trace:

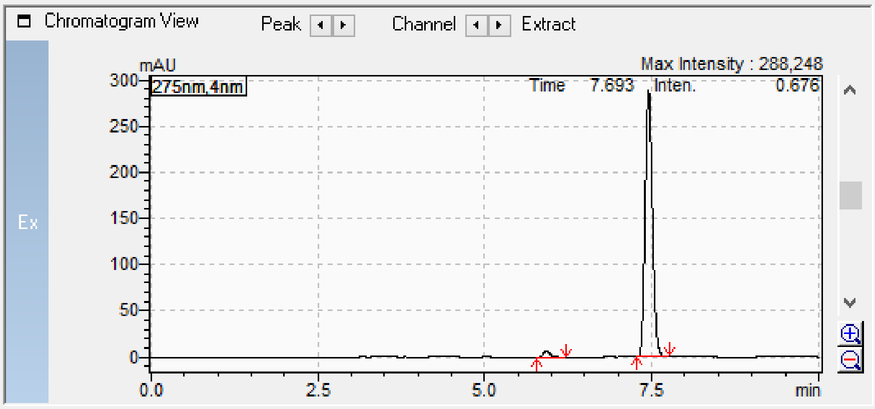

Results View - Peak Table

Peak Table | Compound | Group | Calibration Curve

Peak#	Ret. Time	Mark	Area	Height	Area%
1	5.924	M	39755	6433	1.789
2	7.453	M	2182125	287670	98.211
Total			2221881	294102	100.000

Extracted 275nm | Ch1 254nm

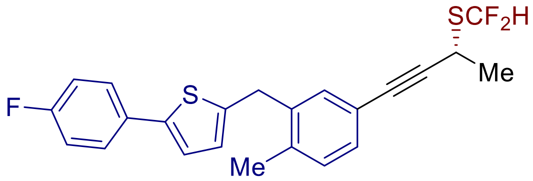

Canagliflozin precusor

6c

Racemic trace:

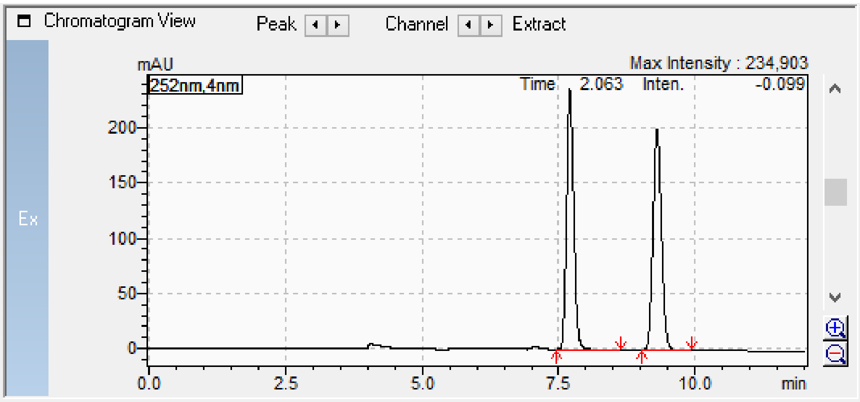

Results View - Peak Table

Peak Table | Compound | Group | Calibration Curve

Peak#	Ret. Time	Mark	Area	Height	Area%
1	7.704		2150262	235383	50.095
2	9.299		2142115	199115	49.905
Total			4292377	434497	100.000

Extracted 252nm | Ch1 254nm

Enantioenriched trace:

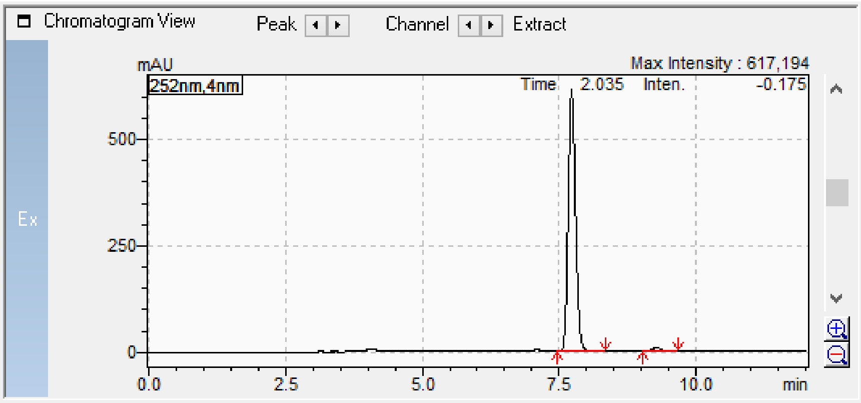

Results View - Peak Table

Peak Table | Compound | Group | Calibration Curve

Peak#	Ret. Time	Mark	Area	Height	Area%
1	7.726		5773602	615477	98.633
2	9.277	M	80039	7868	1.367
Total			5853641	623345	100.000

Extracted 252nm | Ch1 254nm

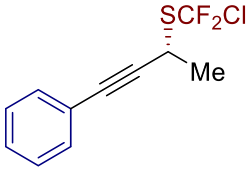

6d

Racemic trace:

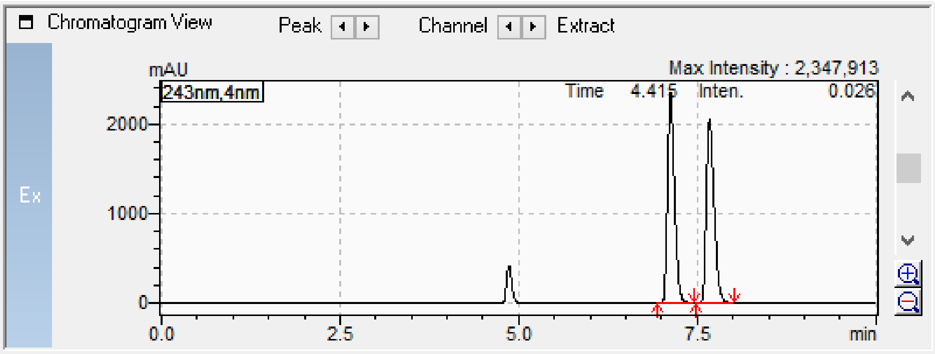

Results View - Peak Table

Peak Table | Compound | Group | Calibration Curve

Peak#	Ret. Time	Mark	Area	Height	Area%
1	7.126	M	15593495	2346100	50.359
2	7.673	M	15371327	2037146	49.641
Total			30964822	4383246	100.000

Extracted 243nm | Ch1 254nm

Enantioenriched trace:

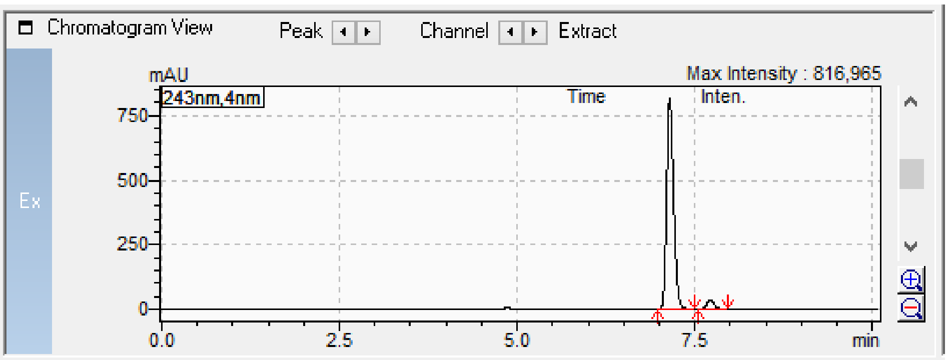

Results View - Peak Table

Peak Table | Compound | Group | Calibration Curve

Peak#	Ret. Time	Mark	Area	Height	Area%
1	7.147	M	5571834	814154	95.580
2	7.715	M	257657	35679	4.420
Total			5829490	849833	100.000

Extracted 243nm | Ch1 254nm

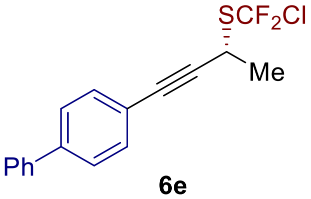

6e

Racemic trace:

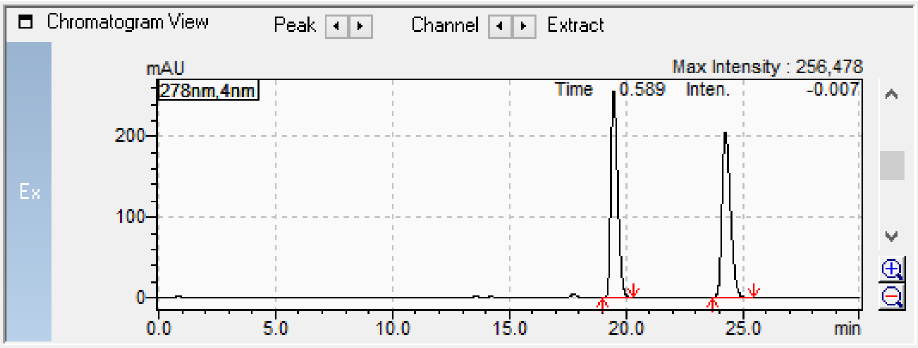

Results View - Peak Table

Peak Table | Compound | Group | Calibration Curve

Peak#	Ret. Time	Mark	Area	Height	Area%
1	19.476		5170271	256367	49.879
2	24.268		5195435	205543	50.121
Total			10365706	461909	100.000

Extracted 278nm | Ch1 254nm

Enantioenriched trace:

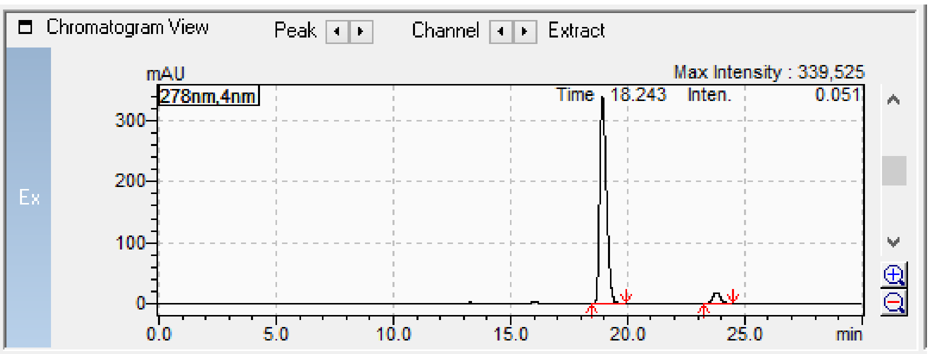

Results View - Peak Table

Peak Table | Compound | Group | Calibration Curve

Peak#	Ret. Time	Mark	Area	Height	Area%
1	18.924		6717980	339257	94.260
2	23.786		409113	17578	5.740
Total			7127093	356835	100.000

Extracted 278nm | Ch1 254nm

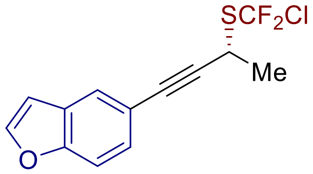

6f

Racemic trace:

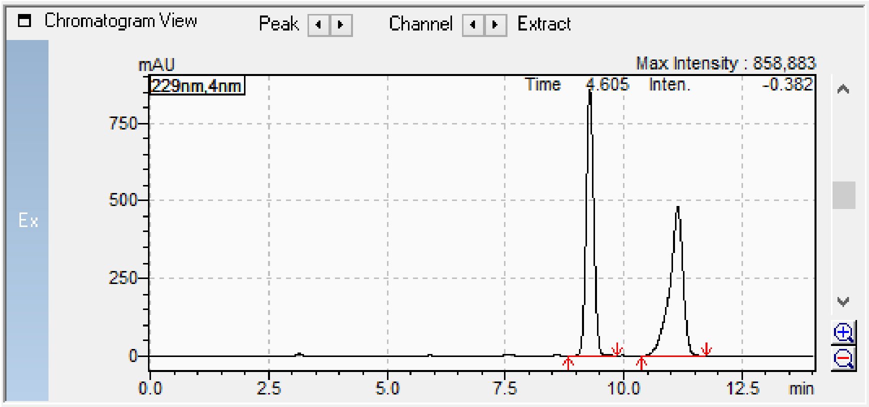

Results View - Peak Table

Peak Table | Compound | Group | Calibration Curve

Peak#	Ret. Time	Mark	Area	Height	Area%
1	9.286	M	9480772	859199	49.996
2	11.145	M	9482430	480623	50.004
Total			18963202	1339821	100.000

Extracted 229nm | Ch1 254nm

Enantioenriched trace:

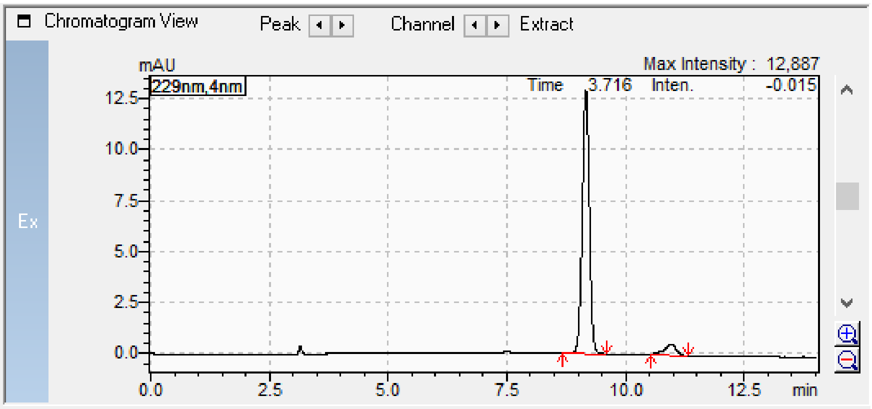

Results View - Peak Table

Peak Table | Compound | Group | Calibration Curve

Peak#	Ret. Time	Mark	Area	Height	Area%
1	9.165	M	135331	12901	94.004
2	10.961	M	8631	507	5.996
Total			143962	13409	100.000

Extracted 229nm | Ch1 254nm

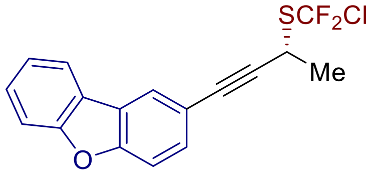

6g

Racemic trace:

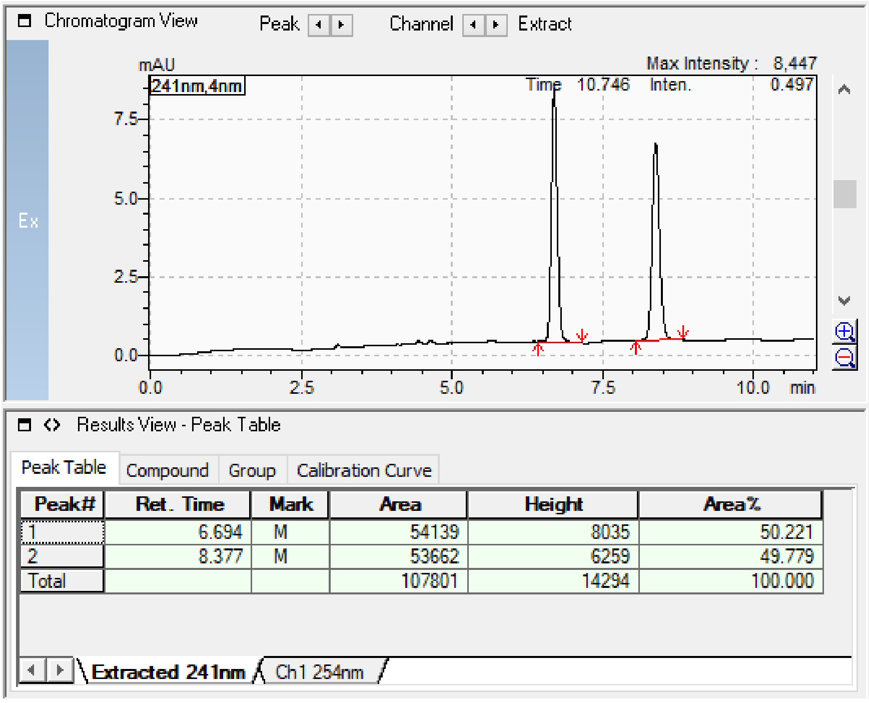

Peak#	Ret. Time	Mark	Area	Height	Area%
1	6.694	M	54139	8035	50.221
2	8.377	M	53662	6259	49.779
Total			107801	14294	100.000

Enantioenriched trace:

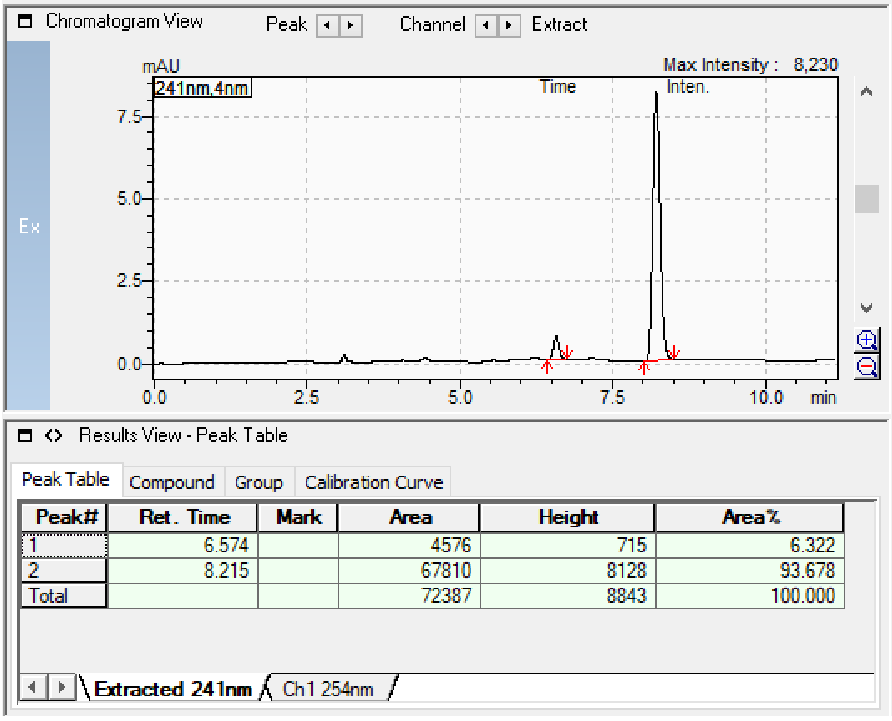

Peak#	Ret. Time	Mark	Area	Height	Area%
1	6.574		4576	715	6.322
2	8.215		67810	8128	93.678
Total			72387	8843	100.000

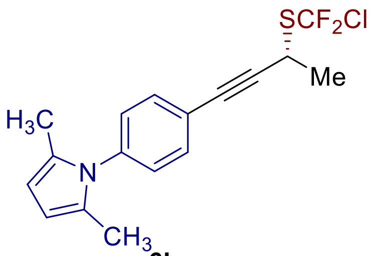

6h

Racemic trace:

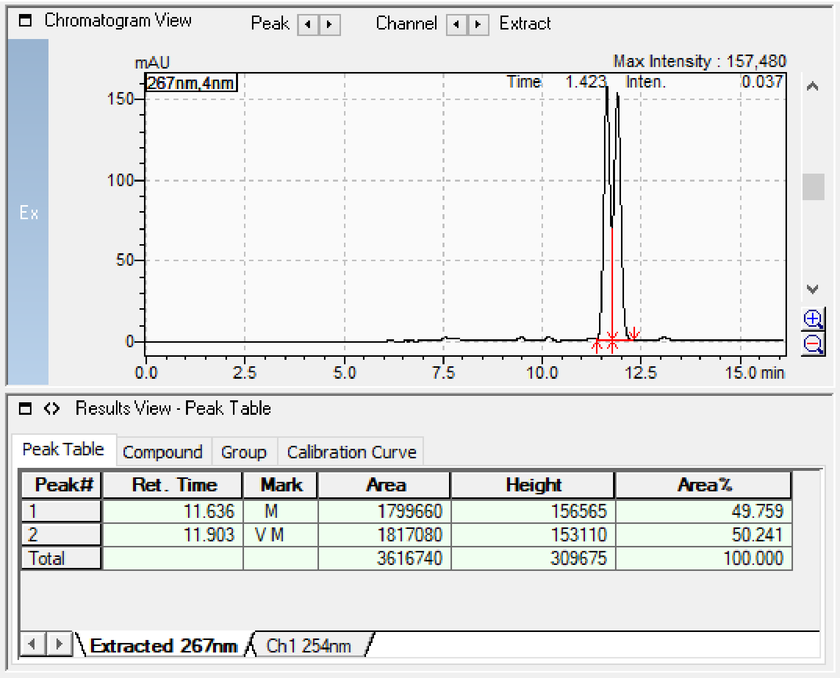

Peak#	Ret. Time	Mark	Area	Height	Area%
1	11.636	M	1799660	156565	49.759
2	11.903	V M	1817080	153110	50.241
Total			3616740	309675	100.000

Enantioenriched trace:

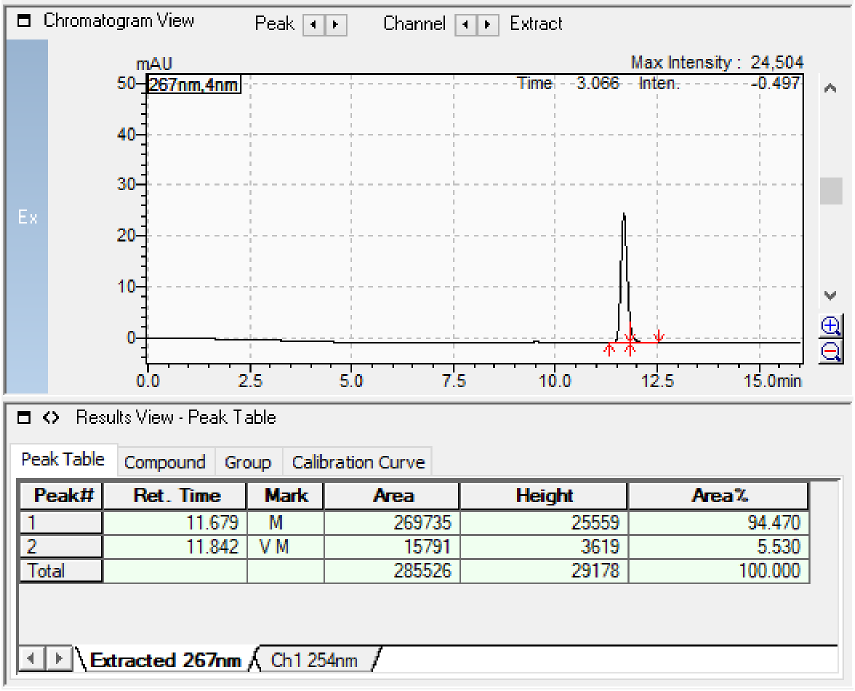

Peak#	Ret. Time	Mark	Area	Height	Area%
1	11.679	M	269735	25559	94.470
2	11.842	V M	15791	3619	5.530
Total			285526	29178	100.000

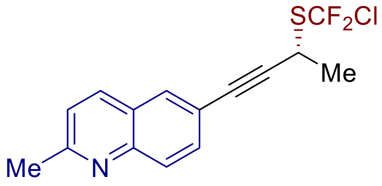

6i

Racemic trace:

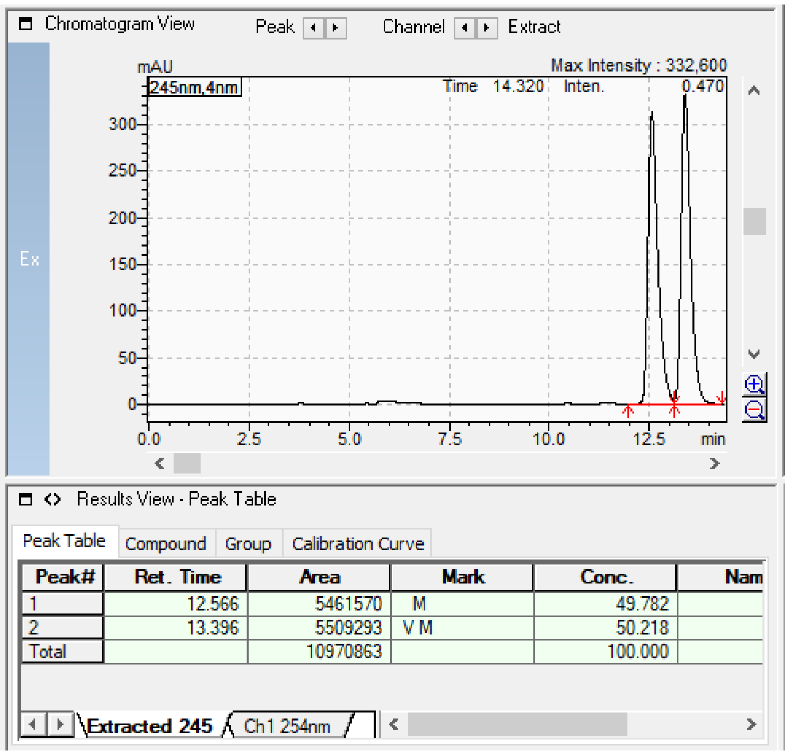

Peak#	Ret. Time	Area	Mark	Conc.	Nam
1	12.566	5461570	M	49.782	
2	13.396	5509293	V M	50.218	
Total		10970863		100.000	

Enantioenriched trace:

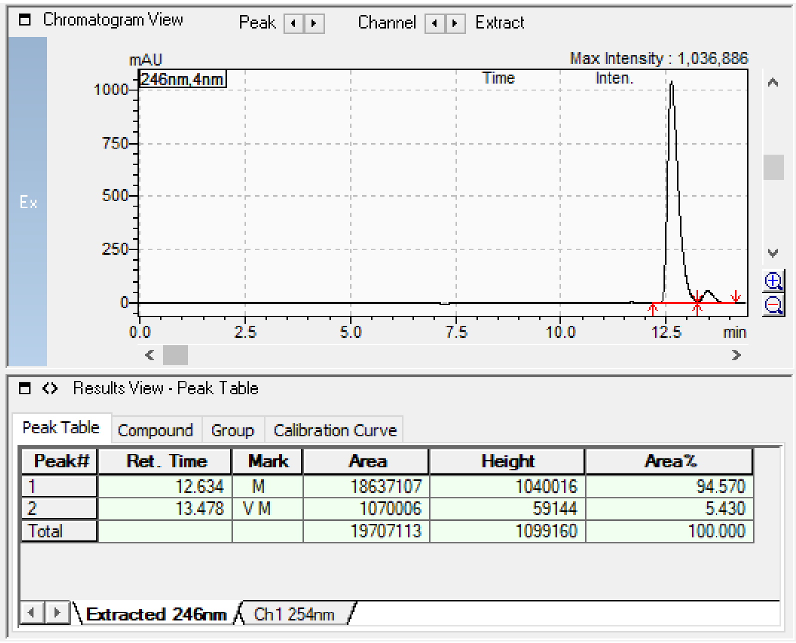

Peak#	Ret. Time	Mark	Area	Height	Area%
1	12.634	M	18637107	1040016	94.570
2	13.478	V M	1070006	59144	5.430
Total			19707113	1099160	100.000

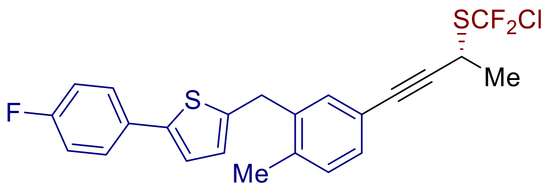

Canagliflozin precusor

6k

Racemic trace:

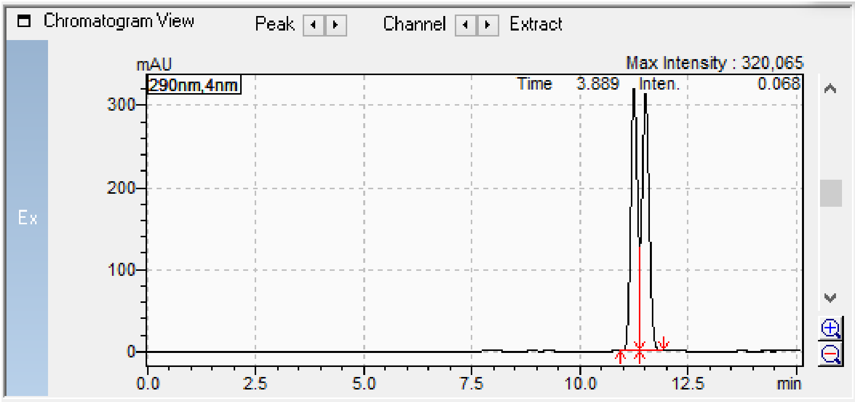

Results View - Peak Table

Peak Table | Compound | Group | Calibration Curve

Peak#	Ret. Time	Mark	Area	Height	Area%
1	11.243	M	3418257	317595	48.839
2	11.507	V M	3580706	313302	51.161
Total			6998963	630896	100.000

Extracted 290nm | Ch1 254nm

Enantioenriched trace:

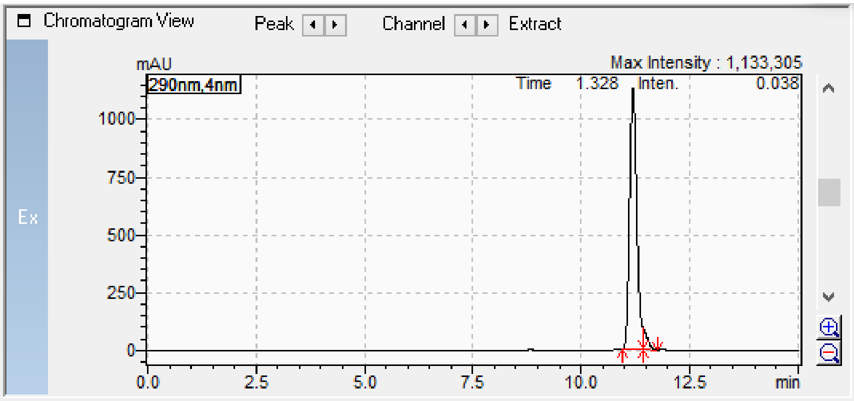

Results View - Peak Table

Peak Table | Compound | Group | Calibration Curve

Peak#	Ret. Time	Mark	Area	Height	Area%
1	11.196	M	12960707	1127949	95.341
2	11.434	V M	633351	89327	4.659
Total			13594057	1217276	100.000

Extracted 290nm | Ch1 254nm

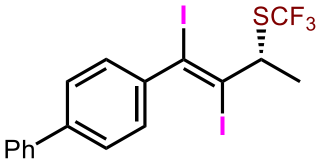

2c'

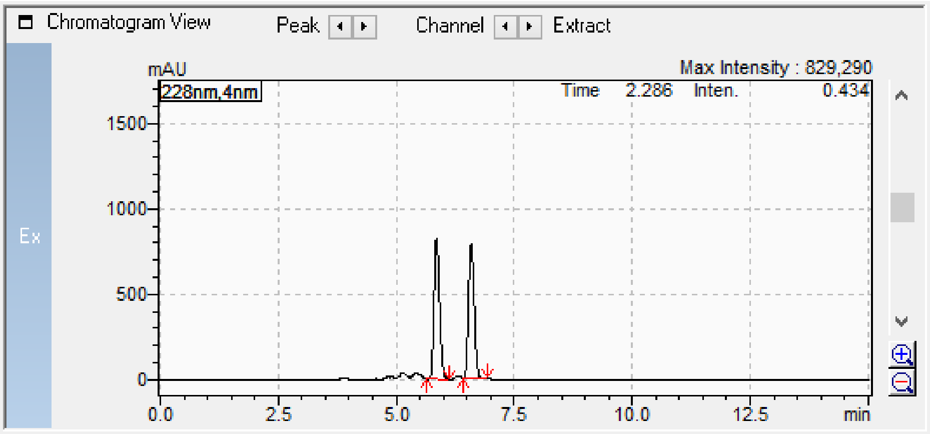

Results View - Peak Table

Peak Table | Compound | Group | Calibration Curve

Peak#	Ret. Time	Mark	Area	Height	Area%
1	5.853	M	6667528	825313	50.485
2	6.588	M	6539535	780980	49.515
Total			13207063	1606294	100.000

Extracted 228nm | Ch1 254nm

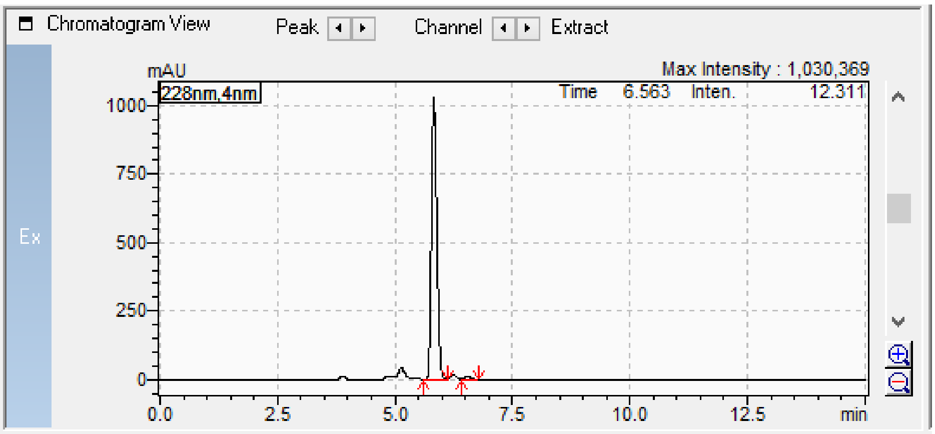

Results View - Peak Table

Peak Table | Compound | Group | Calibration Curve

Peak#	Ret. Time	Mark	Area	Height	Area%
1	5.830	M	8177487	1029386	98.588
2	6.538	M	117155	13593	1.412
Total			8294642	1042979	100.000

Extracted 228nm | Ch1 254nm

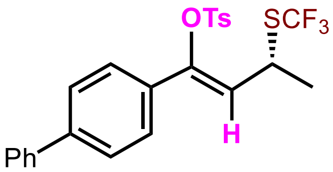

2c''

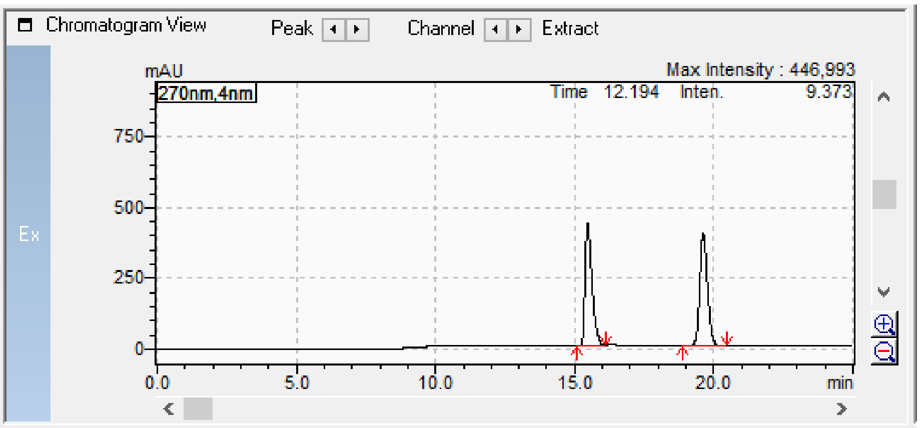

Results View - Peak Table

Peak Table | Compound | Group | Calibration Curve

Peak#	Ret. Time	Mark	Area	Height	Area%
1	15.467	M	7673938	437324	49.970
2	19.618	M	7683133	397064	50.030
Total			15357071	834388	100.000

Extracted 270nm | Ch1 254nm

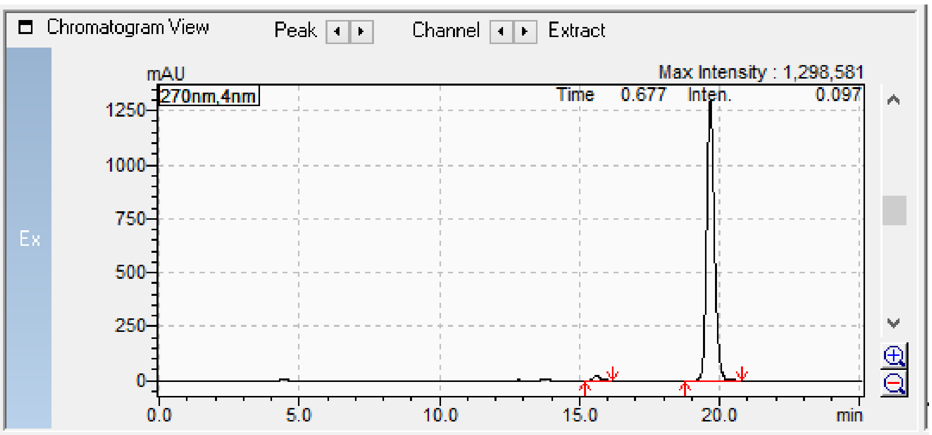

Results View - Peak Table

Peak Table | Compound | Group | Calibration Curve

Peak#	Ret. Time	Mark	Area	Height	Area%
1	15.605	M	375256	21993	1.476
2	19.661	M	25047090	1298155	98.524
Total			25422346	1320147	100.000

Extracted 270nm | Ch1 254nm

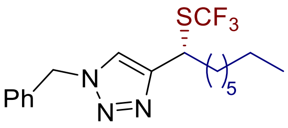

4m'

Racemic trace:

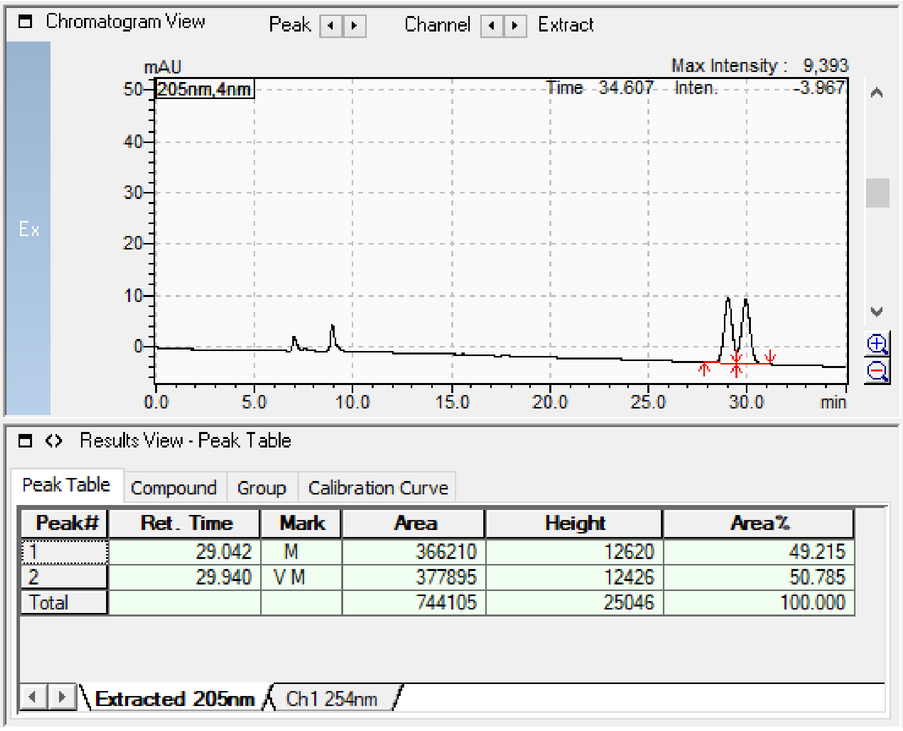

Peak#	Ret. Time	Mark	Area	Height	Area%
1	29.042	M	366210	12620	49.215
2	29.940	V M	377895	12426	50.785
Total			744105	25046	100.000

Enantioenriched trace:

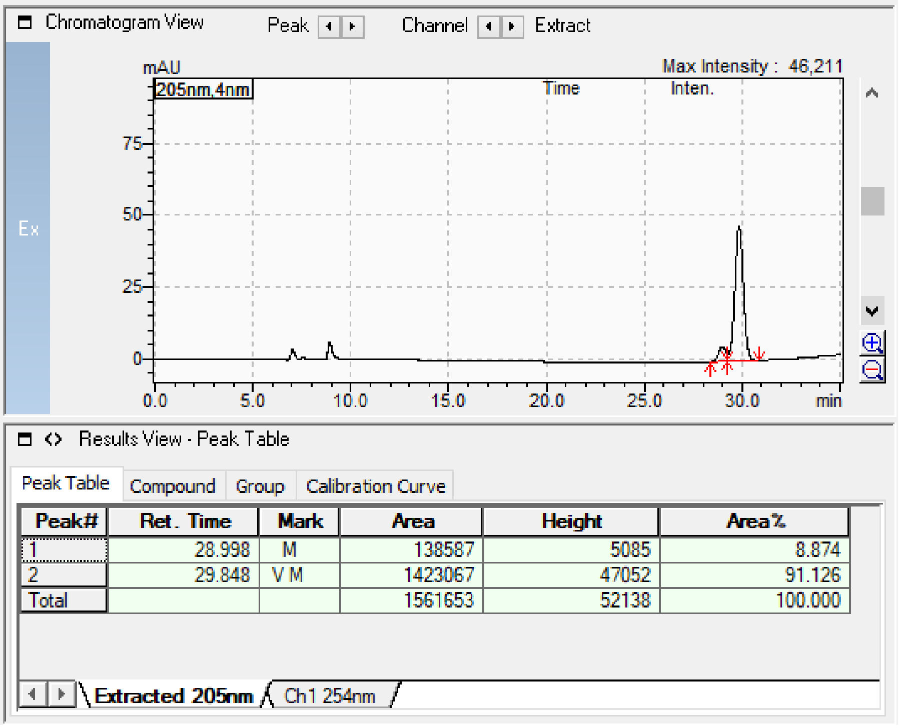

Peak#	Ret. Time	Mark	Area	Height	Area%
1	28.998	M	138587	5085	8.874
2	29.848	V M	1423067	47052	91.126
Total			1561653	52138	100.000

11. References

[1] Rössler, S. L.; Krautwald, S.; Carreira, E. M., *J. Am. Chem. Soc.* **2017,** *139* (10), 3603-3606.

[2] Zhu, J.; Wang, Y.; Charlack, A. D.; Wang, Y.-M., *J. Am. Chem. Soc.* **2022,** *144* (34), 15480-15487.

[3] Liu, Z.; Qin, Z.-Y.; Zhu, L.; Athavale, S. V.; Sengupta, A.; Jia, Z.-J.; Garcia-Borràs, M.; Houk, K. N.; Arnold, F. H., *J. Am. Chem. Soc.* **2022,** *144* (1), 80-85.

[4] Zhu, J.; Xiang, H.; Chang, H.; Corcoran, J. C.; Ding, R.; Xia, Y.; Liu, P.; Wang, Y.-M., *Angew. Chem. Int. Ed.* **2024,** *63* (16), e202318040

[5] Ramazanov, I. R.; Yaroslavova, A. V.; Dzhemilev, U. M.; Nefedov, O. M., *Russian Chemical Bulletin* **2011,** *60* (11), 2275-2278.

[6] Zuo, H.; Klare, H. F. T.; Oestreich, M., *J. Org. Chem.* **2023,** *88* (6), 4024-4027.

[7] Chenniappan, V. K.; Rahaim, R. J., *Org. Lett.* **2016,** *18* (19), 5090-5093.

[8] Ramazanov, I. R.; Yaroslavova, A. V.; Dzhemilev, U. M.; Nefedov, O. M., *Russian Chemical Bulletin* **2011,** *60* (11), 2275-2278.

[9] Nishihara, Y.; Okada, Y.; Jiao, J.; Suetsugu, M.; Lan, M.-T.; Kinoshita, M.; Iwasaki, M.; Takagi, K., *Angew. Chem. Int. Ed.* **2011,** *50* (37), 8660-8664.

[10] Parsons, A. T.; Senecal, T. D.; Buchwald, S. L., *Angew. Chem. Int. Ed.* **2012,** *51* (12), 2947-2950.

[11] Zhu, F.; Miller, E.; Powell, W. C.; Johnson, K.; Beggs, A.; Evenson, G. E.; Walczak, M. A., *Angew. Chem. Int. Ed.* **2022,** *61* (31), e202207153.

[12] Fu, L.; Zhou, S.; Wan, X.; Chen, P.; Liu, G., *J. Am. Chem. Soc.* **2018,** *140* (35), 10965-10969.
